# Supplementary material for: Integrative microbiome- and metatranscriptome-based analyses reveal diagnostic biomarkers for peri-implantitis
Source: NPJ Biofilms Microbiomes. 2025 Aug 26;11:175. doi: 10.1038/s41522-025-00807-6 (PMC12381052; doi:10.1038/s41522-025-00807-6)
Supplement: Supplementary file 1 — Supplementary Information [file 41522_2025_807_MOESM1_ESM.pdf]

Supplementary Information for "Integrative microbiome- and metatranscriptome-based analyses reveal diagnostic biomarkers for peri-implantitis"

Short title: Biomarkers for peri-implantitis

Amruta Joshi[1,2,*], Szymon P. Szafrański[1,2,3,*], Matthias Steglich[1,2,], Ines Yang[1,2], Taoran Qu[1,2], Paula Schaefer-Dreyer[1], Xing Xiao[1,2], Wiebke Behrens[1,2], Jasmin Grischke[1], Susanne Häussler[3,4,5,6], and Meike Stiesch[1,2,3,$]

[1]Department of Prosthetic Dentistry and Biomedical Materials Science, Hannover Medical School, Hannover, Germany

[2]Lower Saxony Centre for Biomedical Engineering, Implant Research and Development (NIFE), Hannover, Germany

[3]Cluster of Excellence RESIST (EXC 2155), Hannover Medical School, Hannover, Germany

[4]Department of Molecular Bacteriology, Helmholtz Centre for Infection Research, Braunschweig, Germany

[5]Institute for Molecular Bacteriology, Twincore, Centre for Clinical and Experimental Infection Research, Hannover, Germany.

[6]Department of Clinical Microbiology, Copenhagen University Hospital - Rigshospitalet, Copenhagen, Denmark

[*]Amruta Joshi and Szymon P. Szafrański contributed equally

[$]correspondence to: Prof. Dr. Meike Stiesch, Department of Prosthetic Dentistry and Biomedical Materials Science, Hannover Medical School Carl-Neuberg-Str.1 30625 Hannover, Germany; stiesch.meike@mh-hannover.de

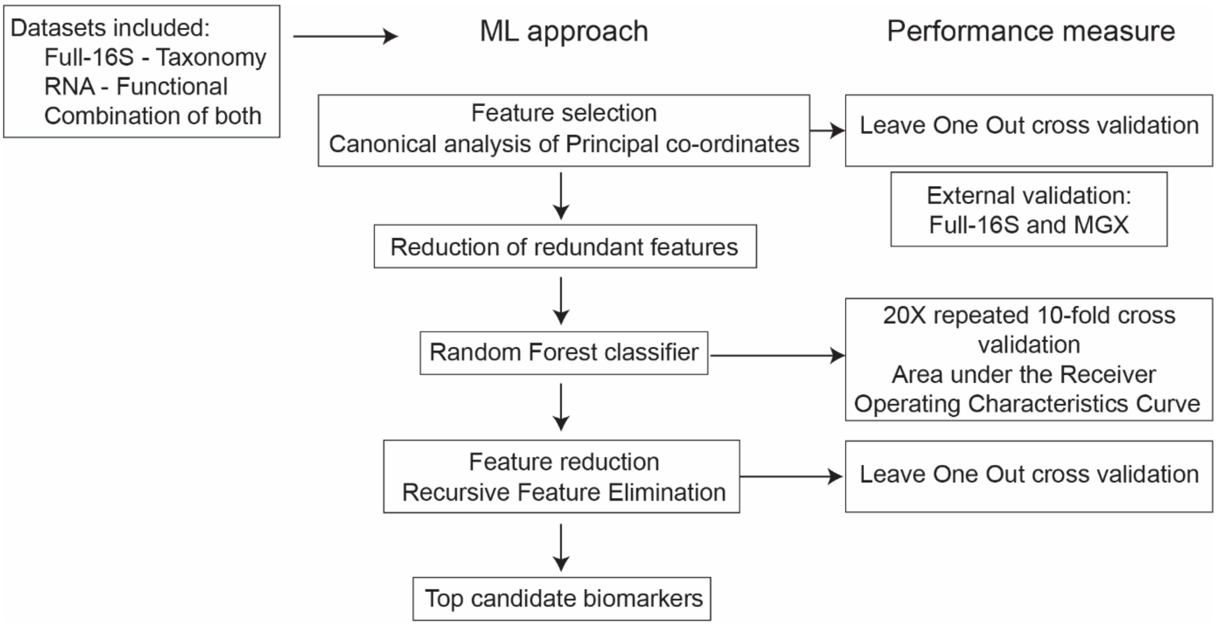

Supplementary Fig. 1 Machine learning workflow for biomarker identification and validation.

Datasets: Full-16S – full length 16S rRNA gene amplicons sequencing; RNA – metatranscriptomics; MGX – metagenomics.

a

Step	Challenges addressed	Comparison to standard protocol	Advantages	Disadvantages
**Sampling technique**	Maximizing input mass; addressing difficulties in collecting sufficient biofilm material due to low biomass in healthy patients.	Both paper points and curette were used	Input mass increased, input for cDNA synthesis increased if applicable	Both attached and non-attached cells analyzed
**Sample preservation**	Preventing degradation of nucleic acids during handling and transport	Storage in RNAprotect at -80°C	Improved sample handling in clinics, long term storage (*e.g.* , multiple yrs) possible	Potential batch effects
**Cell lysis**	Releasing of both DNA and RNA from low-biomass samples while avoiding degradation of RNA and ensuring effective lysis of biofilm cells	Our combined moderate mechanical-enzymatic lysis outperformed a TRIzol-based approach by 50%	Extraction of DNA and RNA suitable from low input and same starting material for downstream application, minimizing degradation of RNA, which is more fragile.	Underrepresentation of hard-to-lyse taxa representing classes Actinobacteria, Clostridia and Coriobacteriia
**Separation**	Preventing co-purification of unwanted substances which may interfere with isolation of nucleic acids	QIAshredder columns were used for separation	Paper points debris removed	Potential batch effects
**Co-isolation**	Achieving simultaneous purification of DNA and RNA which avoids batch effects introduced by multiple sampling	Washing DNA from RNA isolation column in first RNA purification step	Co-isolation of DNA and RNA suitable for downstream application, minimaizing batch effects introduced by independant sampling	This protocol is favoring RNA,low DNA yields suitable for amplicons sequencing but not for metagenomics potential batch effects
**Quality control**	Detecting and quantifying contamination in small biofilm samples. Ensuring consistent RNA quality for transcriptomic applications.	Bioanalyzer profiles generated for all the samples at multiple steps (RNA, mRNA, cDNA, pools), with additional technical replicas is applicable, introduction of blank samples and correlation analysis for identification of contaminating taxa	Improved design of pooled cDNA samples, identifcation and removal of contaminating taxa	Higher resource consumption, potential removal of false negative contaminating taxa
**mRNA sample pooling**	Maximizing input mass addressing difficulties in extracting sufficient mRNA material due to low biofilm biomass	mRNA pooling by patient and diagnosis in low biomass healthy samples	Higher yield of cDNA	Averaging profiles for multiple implants but presnece of individual DNA profiles allows controling
**Re-sequencing**	Maintaining sufficient sequencing depth for low-biomass samples.	Re-sequencing of samples with lowest sequencing depth	Increased sequencing depth	Increased costs
**Summary**	Co-isolation of DNA and RNA from dental implants samples can be challenging especially from healthy patients with little biofilms	We created tailored protocol addressing multiple challenges	Our protocol co-isolates DNA and RNA samples suitable for downstream amplicons and RNA sequencing	Our protocol is time and cost consuming and may introduce some batch effects

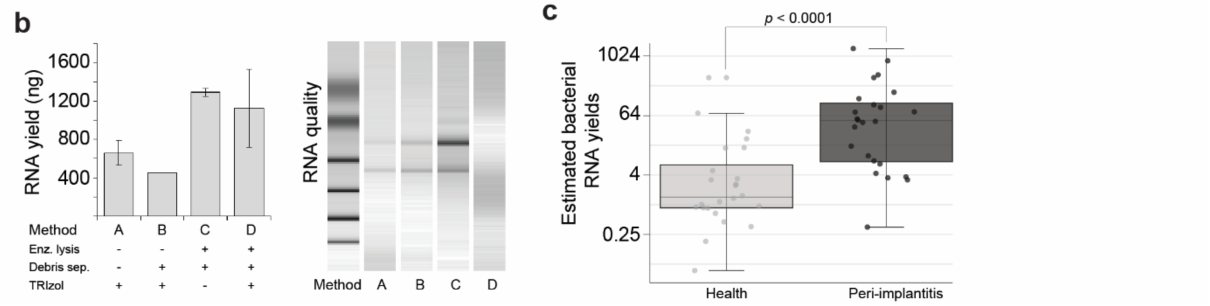

Supplementary Fig 2. Optimized co-isolation of DNA and RNA from peri-implant biofilm samples.

a Table summarizing the key challenges in extracting DNA and RNA from low-biomass biofilm samples and comparison of the optimized protocol with standard extraction approaches. **b** Bar plot and accompanying gels from gel electrophoresis represent the RNA yield obtained from standard samples using different extraction methods. **c** Box and Whisker plot comparing bacterial RNA quantity (calculated as total mRNA multiplied by fraction of reads mapped to bacteria) between healthy and peri-implantitis biofilm samples.

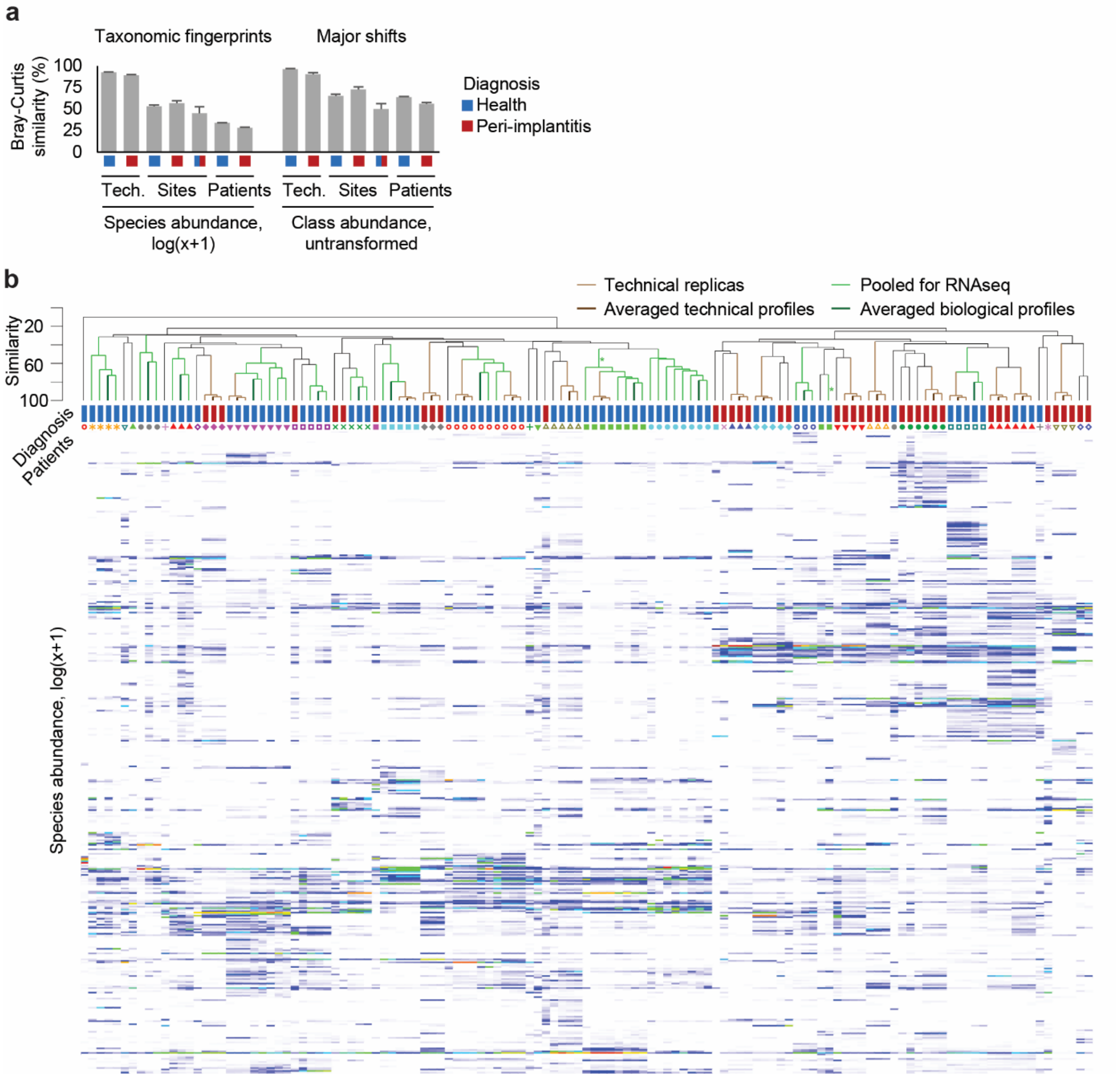

Supplementary Fig. 3 Inter- and Intra-individual variability in the microbiome across samples.

a Bar graph depicting Bray-Curtis similarity at the species and class levels, showing variability within and between technical replicates, different sites within the same patient, and between different patients. **b** Heatmap illustrating the log (x + 1) normalized relative abundances of species (based on full 16S data, with an abundance cut-off of 0.1% across all samples). Samples are coloured according to their clinical diagnosis group and labelled by the patient of origin. The samples are clustered using the Bray-Curtis similarity matrix, and the colours on the dendrogram represent the group of samples that underwent pooling of RNA counterparts.

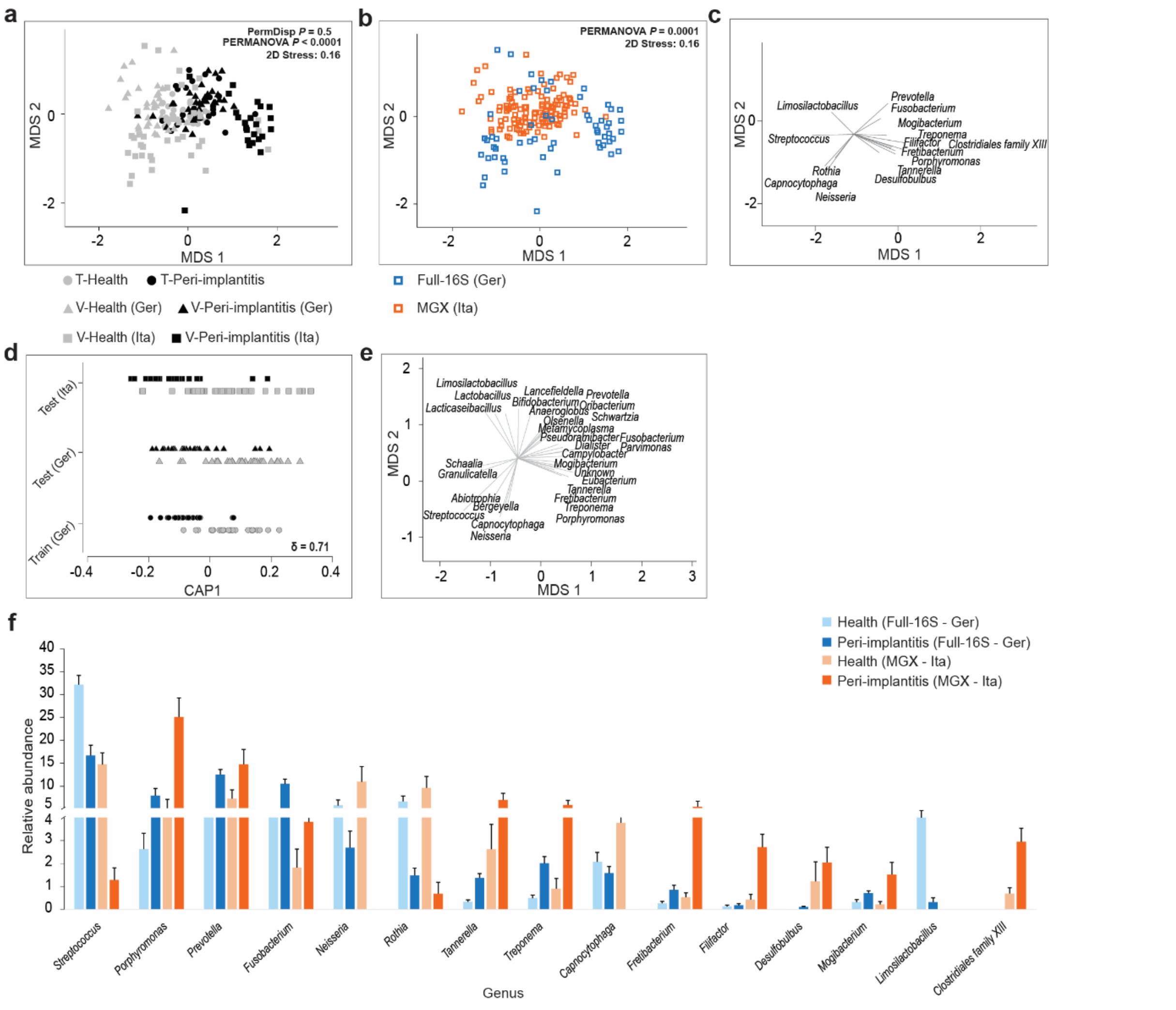

Supplementary Fig 4. Separation between diagnosis groups based on genus-level taxa in full-16S training and test (Germany) and metagenomic (Italy) datasets.

a Non-metric multi-dimensional scaling (nMDS) plot based on Bray-Curtis dissimilarity matrices of 16S rRNA gene amplicon data, aggregated to genus level features of the training and two validation set samples. **b** nMDS plot from figure a, samples denoted according to sequencing method (full-16S and MGX) and population (Germany and Italy) **c** genera present in both full-16S and MGX datasets and that showed highest pearson correlation (cut-off ±0.4) with MDS1 axis. **d** Canonical analysis of principal co-ordinates (CAP) constrained ordination plot of genus level, illustrating the CAP axis that maximizes the separation of the two 'a priori' groups based on diagnosis. The performance of the CAP model on the two validation sets shows the successful allocation of validation samples to their respective diagnosis groups. **e** Vector overlays on the nMDS plot show the genera with highest correlation to the CAP1 axis that best separates the diagnosis groups as seen in (d). **f** Relative abundances of the highly correlated genera shown in (c) across diagnosis groups for both full-16S (Germany) and MGX (Italy) sets. T - training dataset, V - validation dataset, Ger – primary study population based on full-16S dataset from Germany; Ita – External validation population based on shotgun metagenomics (MGX) from Italy.

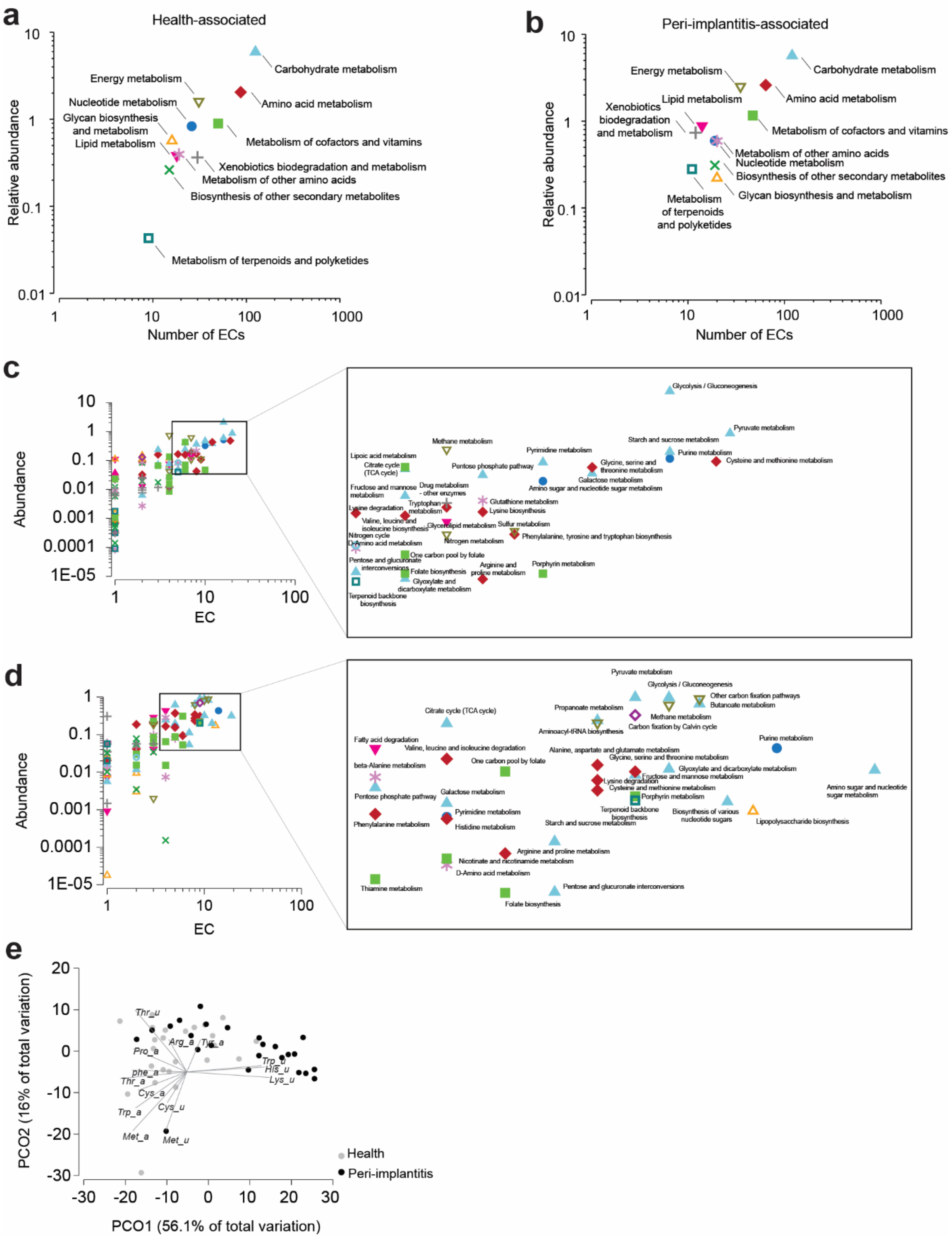

Supplementary Fig 5 Abundances of KEGG functional categories based on EC across diagnosis groups

a Scatterplot for the metabolic categories in the metatranscriptome dataset as classified according to the KEGG Level 1 SEED subsystem, with numbers of health-associated ECs and their relative abundances shown on the x and y axis respectively. **b** Same as figure (a) but for peri-implantitis-associated ECs. Scatterplot shown at the resolution of specific metabolic pathways identified using BRITE classification. **c** health-associated and **d** peri-implantitis-associated ECs. The most prominent pathways

are labelled and shown on the left. **e** PCoA ordination plot based on a Bray-Curtis matrix of normalized EC counts associated with different amino acid anabolic and utilization pathways. (PERMANOVA p value ≤ 0.0001). Same input as Fig. 5c.

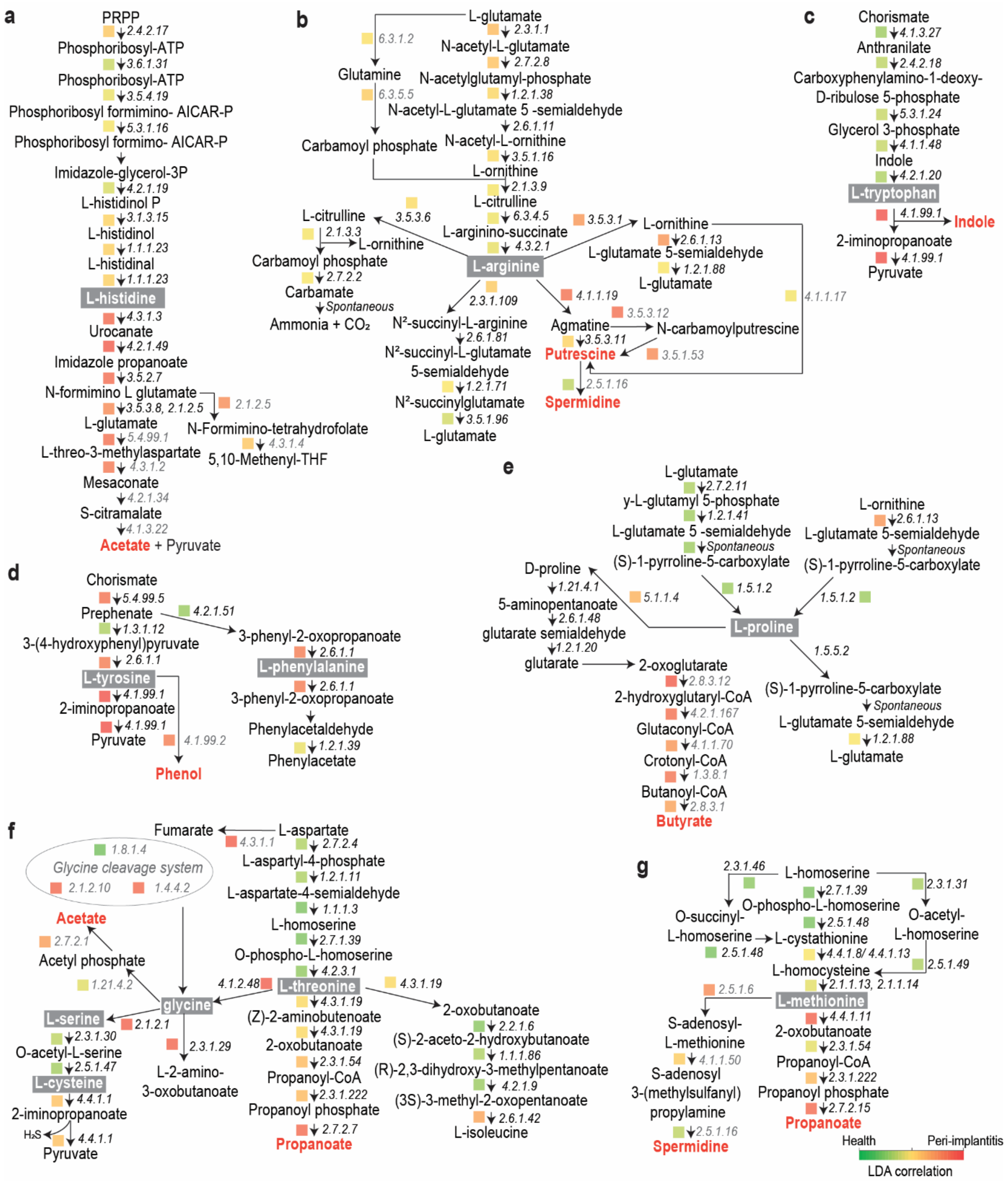

Supplementary Fig. 6. High-resolution view of the curated pathways for selected amino acids based on RNAseq ECs. Anabolic and utilization pathways for **a.** histidine, **b.** arginine, **c.** tryptophan, **d.** tyrosine and phenylalanine, **e.** proline, **f.** threonine, glycine, serine and cysteine, and **g.** methionine were identified using metatranscriptomic data. Colour scale indicates the 'canonical analysis of principal co-ordinates (CAP)'-based linear discriminant analysis (LDA) correlation of each EC to the CAP1 axis that best separates the diagnosis groups.

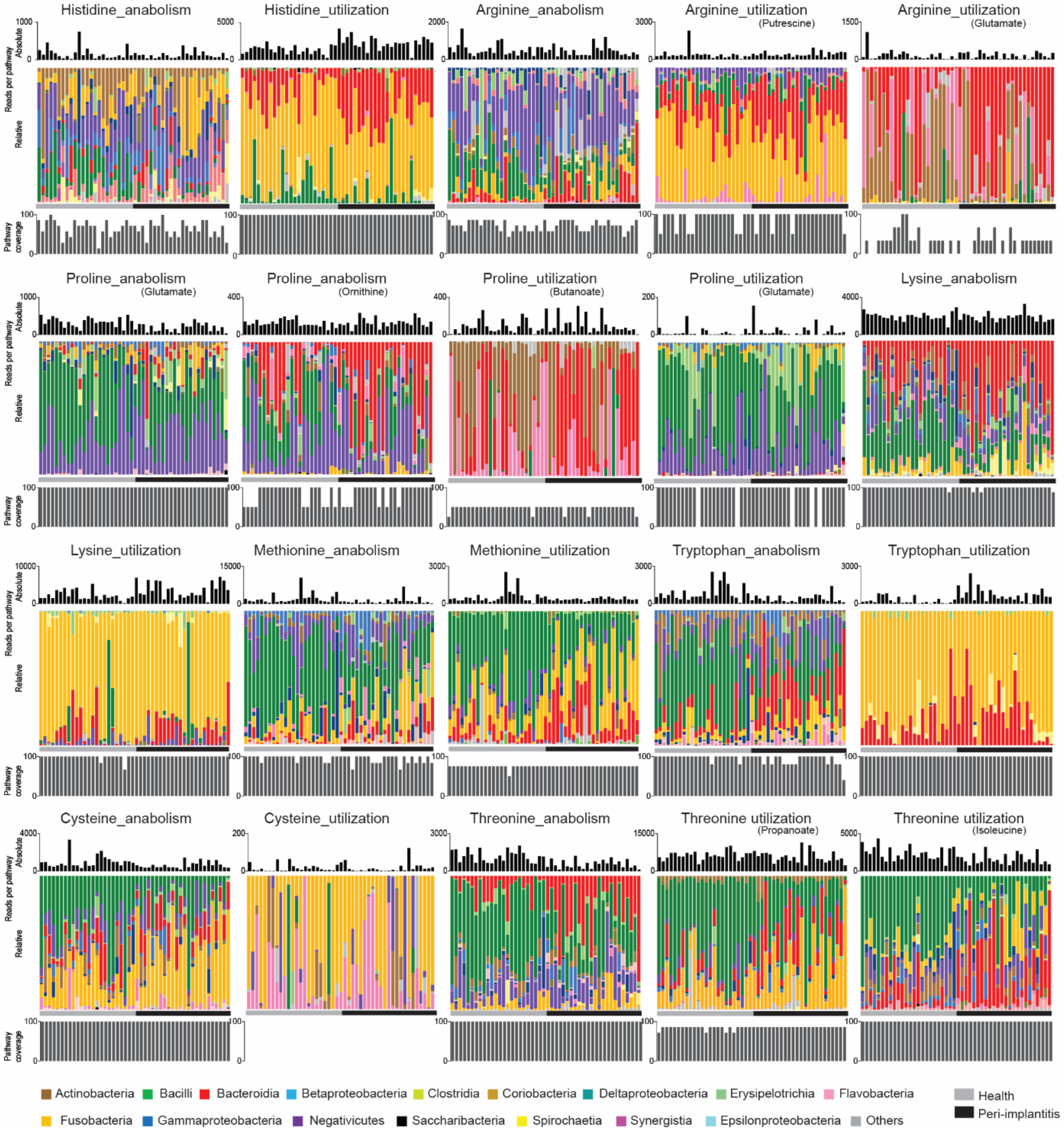

Supplementary Fig. 7. Pathway abundance and coverage of specific amino acid anabolic and utilization pathways in each sample from healthy and peri-implantitis groups. Absolute reads of all ECs per pathway per sample are shown. Relative contributions of bacterial taxonomic classes contributing to EC activites within each amino acid related pathway are indicated with specific colour for each class. Mean relative abundances of the contributing bacterial classes to selected pathways are shown in Fig. 6 of the manuscript. For each pathway, pathway coverage is shown as percentage of ECs whose abundances meet the threshold of median abundance across all samples. The sole EC assigned to cysteine utilization pathway did not meet the strict median threshold criteria. See Methods section.

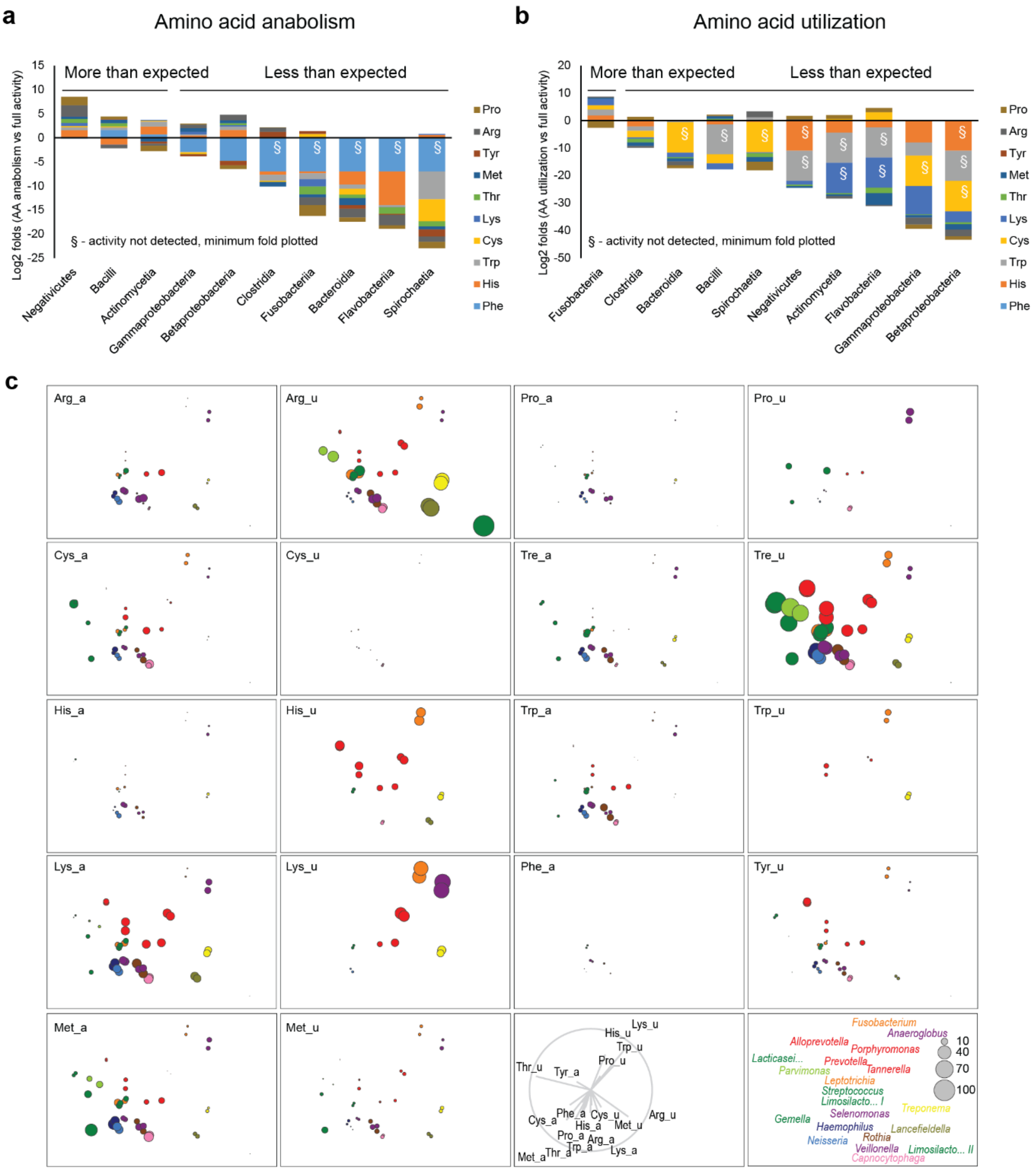

Supplementary Fig. 8. Taxon-specific strategies for amino acids metabolism.

a Log 2-fold change plotted for transcriptional anabolic activities of specific bacterial classes against their full amino acid activity. **b** Log 2-fold change plotted for transcriptional catabolic activities of specific bacterial classes against their full amino acid activity. **c** Non-metric multi-dimensional scaling (nMDS) plots depicting amino acid metabolism for 22 genera for specific anabolic and utilization pathways. Vector overlays displaying Pearson's correlations (cut-off > 0.5 or < -0.5) between the amino acid-related pathways and genera plotted in two separate graphs at the bottom right, with longer vectors indicating higher dissimilarity. Bubble sizes reflect % of reads (EC-based) assigned to specific amino acid-related activities. Genera were split by health status (see **Fig. 7b**).

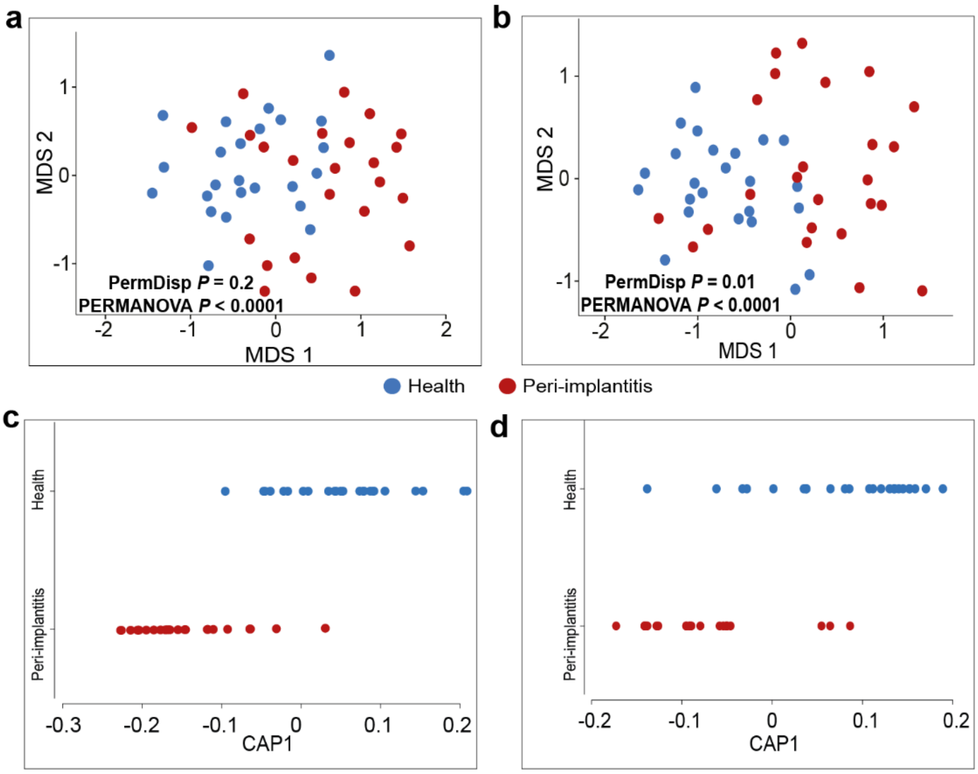

Supplementary Fig 9. Separation between diagnosis groups based on combined datasets.

Non-metric multi-dimensional scaling (nMDS) plots **a** species + EC4 combination and **b** genus + EC4 combination. Canonical analysis of principal co-ordinates (CAP) plots of **c** species + EC4 combination and **d** genus + EC4 combination, illustrating the CAP1 axis that maximizes the separation of the two ‘a priori’ groups based on diagnosis.

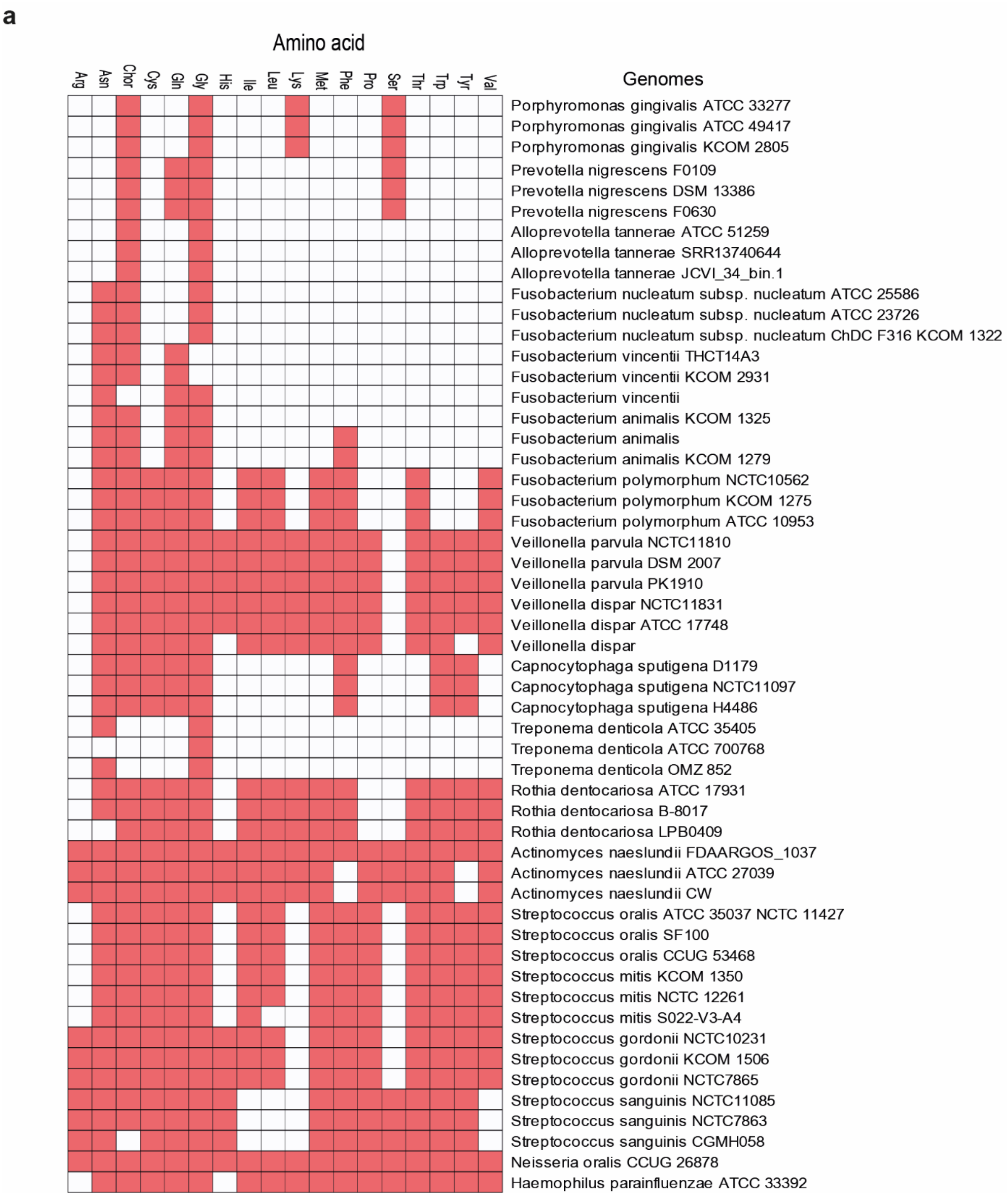

Supplementary Fig. 10. GapMind based cross-validation of the curated amino acid biosynthetic activities of the representative species from the 20 most highly abundant genera detected in our study.

Supplementary Files

A. Demographic and clinical characteristics of the study cohort.

B. Demographic and clinical characteristics of the validation cohort.

C. Full-16S based species level composition. Statistical significance, CAP-based LDA correlation, species-level mean abundances in healthy and peri-implantitis groups

D. Full-16S based genus level composition. Statistical significance, genus-level mean abundances in healthy and peri-implantitis groups

E. Full-16S based class level composition. Statistical significance, class-level mean abundances in healthy and peri-implantitis groups.

F. Metatranscriptome-based features (Enzyme Commission numbers - EC). Statistical significance, CAP-based LDA correlation, Mean 4-digit level EC abundances in healthy and peri-implantitis groups.

G. Metatranscriptome-based class level taxonomic features. Statistical significance, class level mean abundances in healthy and peri-implantitis groups.

H. Curated amino acid metabolism dataset. Mean abundances of ECs aggregated to anabolic and catabolic pathways and their transcribing active taxa.

I. Representative 16S rRNA gene sequences for full-16S taxa designated with OTU numbers.

J. Full-16S and metatranscriptomic sequencing reads per sample

A. Demographic and clinical characteristics of the study cohort

	Health, Mean ± SD/ %distribution	Peri-implantitis, Mean ± SD/ % distribution	P values
**Age**	68 ± 9.42	69 ± 7.52	>0.05
**Sex**	Male:43%; Female:57%	Male:37%; Female:69%	>0.05
**BOP**	Yes:0%; No:100%	Yes:83%; No:17%	<0.0001*
**PD**	3.17 ± 1.62	6.88 ± 1.99	<0.0001*
**GI**	0.3 ± 0.46	2.26 ± 0.53	<0.0001*
**PI**	0.35 ± 0.56	2 ± 0.93	<0.0001*
**Suppuration**	Yes:0%; No:100%	Yes:54%; No:46%	<0.0001*
**Implants in function (years)**	8.48 ± 1.5	9.17 ± 0.8 years	>0.05
**PICF volume**	54.45 ± 0.56	122.12 ± 44.01	<0.0001*
**Total Bacterial RNA**	23.02 ± 74.76	168.71 ± 324.44	<0.0001*
**Smoking**	Yes:5%; No:95%	Yes:13%; No:87%	>0.05

B. Demographic and clinical characteristics of the validation cohort

	Health, Mean ± SD/ %distribution	Peri-implantitis, Mean ± SD/ % distribution	P values
**Age**	56.82 ±14.89	71.41 ± 10.92	0.0002
**Sex**	Male:38%; Female:62%	Male:52%; Female:48%	0.7518
**BOP**	Yes:0%; No:100%	Yes:96%; No:4%	<0.0001
**PD**	2.22 ± 0.38	7.26 ± 2.59	<0.0001
**GI**	0.2 ± 0.12	2.08 ± 1.05	<0.0001
**PI**	0.73 ± 0.41	1.88 ± 1.2	<0.0001

C. Full-16S based species level composition

Species_OTUs	Mean abundance	Std_deviation	Mean abundance - health	Mean abundance - peri-implantitis	P value	FDR corrected P value	LDA correlation (CAP)
Abiotrophia defectiva	0.721220539	1.717509468	0.824976731	0.617464347	0.289502698	0.600118256	0.221826226
Absconditabacteria G-1 sp. HMT-345	0.009691717	0.027249979	0.003095663	0.01628777	0.515046337	0.765935419	-0.125453316
Absconditabacteria G-1 sp. HMT-874	0.00763197	0.034236036	0.000416686	0.014847253	0.323720698	0.600118256	-0.002644887
Absconditabacteria G-1 sp. HMT-875	0.000629407	0.002740209	0.000337765	0.00092105	0.572000957	0.787351719	-0.138663577
Actinomyces cardiffensis	0.013303359	0.036682082	0.019917694	0.006689023	0.391102978	0.654264335	0.106102813
Actinomyces dentalis	0.035614236	0.097459693	0.053873737	0.017354736	0.030059852	0.325353415	0.316728091
Actinomyces georgiae	0.008185342	0.026521457	0.013412489	0.002958194	0.424430938	0.675867614	0.075080745
Actinomyces gerencseriae	0.128840579	0.491825183	0.213808022	0.043873135	0.030926562	0.325353415	0.285337788
Actinomyces graevenitzii	0.017261614	0.040000379	0.025867487	0.008655741	0.023001242	0.280880546	0.392375915
Actinomyces israelii	0.09673679	0.230312564	0.065196322	0.128277258	0.458567743	0.71421291	-0.098817934
Actinomyces johnsonii	0.009022968	0.029032221	0.010493342	0.007552593	0.340189426	0.600118256	0.213127171
Actinomyces massiliensis	0.011896965	0.032749836	0.020550677	0.003243253	0.006984909	0.192529246	0.345329298
Actinomyces meyeri	0.041402177	0.085137953	0.070723575	0.01208078	0.010218587	0.192529246	0.282329513
Actinomyces naeslundii	0.123700144	0.295642344	0.210339223	0.037061065	0.047287548	0.360813321	0.197729391
Actinomyces odontolyticus	0.172036823	0.401279059	0.269974791	0.074098856	0.00536992	0.192529246	0.399263229
Actinomyces oris	0.53356549	2.255897331	0.966081953	0.101049028	0.170608667	0.56290201	0.208897414
Actinomyces sp. HMT-169	0.162340412	0.494864812	0.248546649	0.076134175	0.053187927	0.368944353	0.37843975
Actinomyces sp. HMT-170	0.008942134	0.050538437	0.016512848	0.00137142	0.322518942	0.600118256	0.066914478
Actinomyces sp. HMT-171	0.019110367	0.041330174	0.01868043	0.019540304	0.661705972	0.827132464	0.084565262
Actinomyces sp. HMT-172	0.171839457	0.49872585	0.200810849	0.142868064	0.010852663	0.194229447	0.538809106
Actinomyces sp. HMT-175	0.085788328	0.248440413	0.096963944	0.074612712	0.004953567	0.192529246	0.273397797
Actinomyces sp. HMT-178	0.006748662	0.026795052	0.012918389	0.000578935	0.184615205	0.577490913	0.229866948
Actinomyces sp. HMT-180	0.10917342	0.19203197	0.077613452	0.140733388	0.666667386	0.831697034	-0.167304511
Actinomyces sp. HMT-414	0.00193931	0.013295237	0.003878619	0	0.3378947	0.600118256	0.184260724
Actinomyces sp. HMT-448	0.001547183	0.005499558	0.002367919	0.000726446	0.399060512	0.658991323	0.220470243
Actinomyces sp. HMT-525	0.013418583	0.034730047	0.020358048	0.006479117	0.118181897	0.555892625	0.043791308
Actinomyces sp. HMT-877	0.001053201	0.005641133	0.002106402	0	0.041041673	0.35218192	0.267318243
Actinomyces sp. HMT-896	4.76875E-05	0.000253379	9.5375E-05	0	0.161691678	0.561061286	0.300178068
Actinomyces sp. HMT-897	0.043261761	0.120960632	0.017008896	0.069514625	0.394164891	0.656941485	-0.096264972
Actinomyces timonensis	0.000840539	0.005762442	0.001681077	0	0.3378947	0.600118256	0.10002725
Actinomyces turicensis	0.00149275	0.009207992	0.002683376	0.000302124	0.605877247	0.791629736	-0.295694905
Aggregatibacter actinomycetemcomitans	0.900916117	3.740730236	1.717484239	0.084347994	0.012580651	0.202666974	0.438919097
Aggregatibacter aphrophilus	0.183258506	0.599354564	0.144540081	0.221976931	0.421626231	0.674389564	-0.03745159
Aggregatibacter sp. HMT-458	0.011230313	0.048617831	0.00701459	0.015446036	0.322518942	0.600118256	0.071103709
Aggregatibacter sp. HMT-513	0.01572192	0.105641592	0.030965816	0.000478025	0.572000957	0.787351719	-0.167120655
Aggregatibacter sp. HMT-898	0.000197598	0.001082344	0.000395196	0	0.161691678	0.561061286	0.176815071
Algoriella xinjiangensis	0	0	0	0	NA	NA	0
Alloprevotella rava	0.043002012	0.088409871	0.051156391	0.034847633	0.296234892	0.600118256	0.042698781
Alloprevotella sp. HMT-308	0.052802394	0.089325051	0.068584013	0.037020776	0.263711386	0.600118256	0.072065413
Alloprevotella sp. HMT-473	0.063783218	0.346359018	0.021328118	0.106238318	0.942784758	1	-0.181363696
Alloprevotella sp. HMT-912	0.017090807	0.047136191	0.009841128	0.024340486	0.400583702	0.658991323	-0.211986469
Alloprevotella sp. HMT-913	0.025094589	0.072797849	0.00908722	0.041101958	0.220512914	0.600118256	-0.223045024
Alloprevotella sp. HMT-914	0.008536381	0.038223627	0.014244342	0.00282842	0.053453355	0.368944353	0.149413516
Alloprevotella tannerae	1.550027871	2.806900287	0.784779987	2.315275755	0.386226002	0.652269976	-0.141137072
Alloscardovia omnicolens	0.020267517	0.080036685	0.033680311	0.006854724	0.911934721	1	-0.014168232
Anaeroglobus geminatus	0.657067711	1.761425482	0.242234865	1.071900558	0.124140254	0.561061286	-0.316464436
Anaerolineae G-1 sp. HMT-439	0.046656691	0.138162071	0.010465458	0.082847923	0.033603109	0.338697999	-0.291895859
Atopobium deltae	0	0	0	0	NA	NA	0
Atopobium parvulum	0.179212412	0.311277816	0.197188624	0.1612362	0.266217857	0.600118256	0.155247898
Atopobium rimae	1.211031672	2.386858825	0.389537229	2.032526115	0.063770853	0.404944918	-0.321078405
Atopobium sp. HMT-199	0.058711782	0.156096984	0.02752389	0.089899674	0.82826783	0.9615175	0.039899137
Atopobium sp. HMT-810	6.89298E-05	0.000472559	0.00013786	0	0.3378947	0.600118256	-0.152673171
Bacillus rigiliprofundi	4.72368E-05	0.000323839	9.44737E-05	0	0.3378947	0.600118256	0.10002725
Bacteroidales G-2 sp. HMT-274	0.261496915	0.477729118	0.247375837	0.275617993	0.558807139	0.787351719	-0.080736121
Bacteroidetes G-3 sp. HMT-365	0.080685673	0.345038404	0.044377373	0.116993974	0.961998777	1	-0.029180364
Bacteroidetes G-3 sp. HMT-436	0.00020203	0.001385048	0	0.00040406	0.3378947	0.600118256	-0.057910513

Species_OTUs	Mean abundance	Std_deviation	Mean abundance - health	Mean abundance - peri-implantitis	P value	FDR corrected P value	LDA correlation (CAP)
Bacteroidetes G-3 sp. HMT-899	0.001037291	0.004225435	0.000897646	0.001176936	1	1	-0.028645284
Bacteroidetes G-5 sp. HMT-505	0.020343845	0.139470372	0.040687689	0	0.3378947	0.600118256	0.10002725
Bacteroidetes G-5 sp. HMT-511	0.104430351	0.334584563	0.118236977	0.090623725	0.238190199	0.600118256	-0.183079223
Bacteroidetes G-6 sp. HMT-516	0.008602247	0.055784707	0.016267005	0.000937488	1	1	0.039814741
Bergeyella sp. HMT-322	0.085034018	0.117801094	0.12657976	0.043488277	0.047831418	0.360813321	0.307466067
Bergeyella sp. HMT-900	0.004265011	0.018547376	0.004929514	0.003600508	0.654727339	0.82327101	-0.048724805
Bergeyella sp. HMT-907	0.003364806	0.016271977	0.000377138	0.006352473	0.572844873	0.787351719	-0.227041214
Bergeyella sp. HMT-931	0.000817769	0.004378248	0.001215046	0.000420493	1	1	-0.159258964
Bifidobacterium dentium	1.131052987	2.152758702	0.943545097	1.318560876	0.908805571	1	0.01414389
Bifidobacterium longum	0.000235249	0.001083733	0.000470498	0	0.041041673	0.35218192	0.275017371
Bulleidia extructa	0.025091245	0.090190792	0.024726891	0.025455599	0.484676702	0.741613748	0.124831757
Campylobacter concisus	0.100971008	0.141804615	0.086668434	0.115273581	0.274224109	0.600118256	-0.112931835
Campylobacter curvus	0.020645263	0.090782148	0.002427865	0.038862661	0.356974132	0.615974385	-0.336117657
Campylobacter gracilis	0.605416573	0.70939058	0.638228717	0.57260443	0.844682202	0.97345408	-0.079470199
Campylobacter rectus	0.124605175	0.2742799	0.071408045	0.177802305	0.487946708	0.742824985	-0.104992911
Campylobacter showae	0.163147108	0.328178752	0.11178392	0.214510297	0.567338827	0.787351719	-0.058662944
Campylobacter sp. HMT-044	0.040720946	0.20108264	0.000924722	0.080517169	0.324784491	0.600118256	-0.192473345
Campylobacter ureolyticus	0.00104968	0.006092068	0.001766484	0.000332876	0.605877247	0.791629736	-0.301903721
Capnocytophaga gingivalis	0.371518193	0.590387017	0.419095281	0.323941106	0.580061008	0.791629736	0.021379408
Capnocytophaga granulosa	0.108279092	0.366120328	0.065289939	0.151268244	0.853828802	0.978666588	0.086808211
Capnocytophaga haemolytica	0.020125069	0.085722642	0.00758689	0.032663247	0.53905226	0.77618636	0.017074246
Capnocytophaga leadbetteri	0.290141053	0.70494225	0.277649551	0.302632555	0.48900893	0.742824985	0.212385625
Capnocytophaga sp. HMT-323	0.020271626	0.138224305	0.040328177	0.000215076	1	1	0.085272516
Capnocytophaga sp. HMT-324	0.002040169	0.011217993	0.004080338	0	0.081078758	0.443836303	0.191438521
Capnocytophaga sp. HMT-326	0.060056373	0.18078579	0.030637697	0.08947505	0.403955579	0.66282117	-0.006286386
Capnocytophaga sp. HMT-335	0.00332273	0.011335756	0.005588117	0.001057342	0.131356584	0.561061286	0.089464152
Capnocytophaga sp. HMT-336	0.041721138	0.121428443	0.062068566	0.021373709	0.678841491	0.840964017	0.107931919
Capnocytophaga sp. HMT-338	0.019090261	0.056297692	0.013779624	0.024400898	0.494875601	0.746427568	-0.103063996
Capnocytophaga sp. HMT-380	0.000252613	0.001731826	0.000505226	0	0.3378947	0.600118256	0.10002725
Capnocytophaga sp. HMT-412	0.051221047	0.319371441	0.099422895	0.003019199	0.043520648	0.354302708	0.301087548
Capnocytophaga sp. HMT-864	0.002573601	0.009453651	0.000828709	0.004318493	0.572844873	0.787351719	0.128567973
Capnocytophaga sp. HMT-902	0.0013822	0.00714061	0.002578429	0.00018597	0.53905226	0.77618636	0.021472158
Capnocytophaga sp. HMT-903	0.001514802	0.010384961	0.003029604	0	0.3378947	0.600118256	0.142143987
Capnocytophaga sputigena	0.527668452	1.405529273	0.304375102	0.750961802	0.762082941	0.898929696	0.112722794
Cardiobacterium hominis	0.172751312	0.358758275	0.132828314	0.212674311	0.734795853	0.880368616	0.110570557
Cardiobacterium valvarum	0.305370283	1.140351759	0.217750617	0.39298995	0.356305122	0.615974385	0.198551358
Carnobacterium iners	0	0	0	0	NA	NA	0
Catonella morbi	0.233177094	0.434958179	0.137298058	0.329056129	0.568368072	0.787351719	-0.290384183
Catonella sp. HMT-164	0.007341176	0.040534658	0.000701288	0.013981065	0.53905226	0.77618636	-0.111758704
Catonella sp. HMT-451	0.009619075	0.048923335	0.014793489	0.00444466	0.322518942	0.600118256	0.178891497
Centipeda periodontii	0.021064492	0.063881412	0.033570107	0.008558877	0.88144709	1	-0.058528251
Clostridiales F-1G-1 sp. HMT-093	0.026377699	0.071525277	0.019467214	0.033288185	0.34961243	0.606568013	-0.009293543
Corynebacterium accolens	0.000483596	0.00331537	0.000967193	0	0.3378947	0.600118256	0.194789908
Corynebacterium amycolatum	0	0	0	0	NA	NA	0
Corynebacterium bovis	0.000266207	0.001825021	0.000532413	0	0.3378947	0.600118256	0.152673171
Corynebacterium callunae	0.001471682	0.00654803	0.002660032	0.000283331	0.53905226	0.77618636	0.11745012
Corynebacterium durum	0.039537638	0.093771521	0.066418682	0.012656594	0.017705319	0.244410386	0.367209513
Corynebacterium kroppenstedtii	5.43242E-05	0.000372428	0.000108648	0	0.3378947	0.600118256	0.068439698
Corynebacterium matruchotii	0.095396564	0.258129546	0.123716398	0.06707673	0.420435818	0.674183698	0.354318894
Corynebacterium striatum	0.000105304	0.000721928	0.000210608	0	0.3378947	0.600118256	0.089498066
Corynebacterium testudinoris	3.09909E-05	0.000212463	6.19818E-05	0	0.3378947	0.600118256	0.194789908
Corynebacterium tuberculostearicum	0.001486119	0.006135314	0.002698601	0.000273637	0.282231305	0.600118256	0.231766388
Cryptobacterium curtum	0.065549254	0.284870268	0.01315841	0.117940097	0.596192422	0.791629736	-0.021201132
Desulfobulbus sp. HMT-041	0.080109069	0.217448888	0.026209812	0.134008326	0.100769381	0.499910603	-0.327547149
Desulfovibrio sp. HMT-040	0.005831665	0.031515313	0.00021774	0.011445591	0.53905226	0.77618636	-0.068555687
Dialister invisus	1.102042648	1.331121885	0.544792726	1.65929257	0.009652171	0.192529246	-0.439148846
Dialister micraerophilus	0.009798788	0.047487331	0.002890862	0.016706714	0.399060512	0.658991323	-0.216030611

Species_OTUs	Mean abundance	Std_deviation	Mean abundance - health	Mean abundance - peri-implantitis	P value	FDR corrected P value	LDA correlation (CAP)
Dialister pneumosintes	0.367718325	0.60151291	0.19402162	0.54141503	0.268470706	0.600118256	-0.31189754
Dialister propionicifaciens	0	0	0	0	NA	NA	0
Dialister sp. HMT-119	0.001617341	0.008006433	0	0.003234683	0.161691678	0.561061286	-0.144210873
Eikenella corrodens	0.224958553	0.259545553	0.20906042	0.240856687	0.983533417	1	0.101974892
Erysipelotrichaceae G-1 sp. HMT-904	0.003907622	0.016279559	0.001384295	0.006430949	0.861315265	0.983696391	-0.100584463
Erysipelotrichaceae G-1 sp. HMT-905	0.008224314	0.02715761	0.005077425	0.011371204	0.705381447	0.856438277	-0.1972672
Eubacterium brachy	0.49905917	0.589028724	0.271291793	0.726826547	0.042373243	0.354302708	-0.325003969
Eubacterium infirmum	0.203724699	1.206763708	0.376612201	0.030837197	0.924427728	1	-0.115483463
Eubacterium minutum	0.005712265	0.014457426	0.007779402	0.003645128	0.458895854	0.71421291	0.157577815
Eubacterium saphenum	0.01790968	0.048639505	0.025492822	0.010326538	0.408886257	0.665750701	0.1774233
Eubacterium sulci	0.006056797	0.022196859	0.011476609	0.000636986	0.007565433	0.192529246	0.488425389
Eubacterium yurii	0.063544215	0.203947954	0.044757296	0.082331135	0.790203354	0.925791752	-0.128120476
Eubacterium yurii subsp. margaretiae	0.001639625	0.008001531	0.002238641	0.001040609	1	1	-0.236521732
Eubacterium yurii subsp. schtitka	0.00253323	0.016621453	0.00021774	0.00484872	1	1	0.013010329
Fretibacterium fastidiosum	0.182985331	0.363925967	0.105838785	0.260131878	0.343459653	0.600118256	-0.200281325
Fretibacterium sp. HMT-358	0.000870587	0.005968445	0.001741174	0	0.3378947	0.600118256	0.163202356
Fretibacterium sp. HMT-359	0.019670102	0.05866461	0.03032885	0.009011354	0.043520648	0.354302708	0.401781378
Fretibacterium sp. HMT-360	0.467045515	1.044652273	0.347425319	0.586665711	0.607924327	0.792307849	0.008819392
Fretibacterium sp. HMT-361	0.014413486	0.091093729	0.000514324	0.028312648	0.579454317	0.791629736	0.045442927
Fretibacterium sp. HMT-362	0.046175491	0.147194495	0.009922587	0.082428396	0.022196784	0.280880546	-0.361702732
Fusobacterium canifelinum	0.003069646	0.009055169	0.00070486	0.005434432	0.198142767	0.600118256	-0.190331969
Fusobacterium nucleatum	1.778436092	3.358301837	0.754373686	2.802498499	0.005543224	0.192529246	-0.457713606
Fusobacterium nucleatum subsp. animalis	0.002325537	0.0058031	0.001043612	0.003607463	0.328986907	0.600118256	-0.245747277
Fusobacterium nucleatum subsp. nucleatum	0.281929648	0.730966175	0.261394406	0.302464891	0.555285334	0.787351719	0.089279268
Fusobacterium nucleatum subsp. vincentii	2.233084312	2.991934103	1.750059216	2.716109409	0.407384924	0.665750701	-0.183465586
Fusobacterium periodonticum	0.024851067	0.050047083	0.044382632	0.005319501	0.001016321	0.107560621	0.421527983
Fusobacterium sp. HMT-203	0.541945177	1.355174728	0.786984057	0.296906297	0.168332904	0.562586284	0.20353131
Fusobacterium sp. HMT-204	0.106216991	0.330396804	0.132753537	0.079680446	0.382935975	0.64893819	0.017467683
Fusobacterium sp. HMT-205	0.000222115	0.00129362	7.2289E-05	0.000371941	1	1	-0.13041679
Fusobacterium sp. HMT-370	0.00121218	0.008310287	0	0.00242436	0.3378947	0.600118256	-0.057910513
Gardnerella vaginalis	0.001299957	0.008912053	0	0.002599913	0.3378947	0.600118256	-0.236906645
Gemella haemolysans	0.100948204	0.258329056	0.116367837	0.085528572	0.630052704	0.806619893	0.082720327
Gemella morbillorum	0.648971441	1.325062047	0.503152374	0.794790507	0.950288296	1	-0.110117695
Gemella sanguinis	0.251591243	0.431486175	0.432820141	0.070362346	0.000909816	0.107560621	0.679416418
Granulicatella adiacens	0.838554135	1.177414433	0.968153579	0.70895469	0.061959539	0.401472521	0.456919502
Granulicatella elegans	0.052570576	0.172434675	0.09239036	0.012750792	0.3286285	0.600118256	0.032635037
Haemophilus parainfluenzae	1.237265605	2.959754947	1.858512787	0.616018424	0.008808283	0.192529246	0.521580995
Haemophilus paraphrohaemolyticus	0.000544143	0.003730456	0.001088286	0	0.3378947	0.600118256	0.10002725
Haemophilus pittmaniae	0.00626733	0.041791417	0	0.01253466	0.161691678	0.561061286	-0.178852833
Haemophilus sp. HMT-036	0.001306764	0.008289456	0.002613528	0	0.161691678	0.561061286	0.15032416
Haemophilus sputorum	0.00352893	0.012835558	0.006689785	0.000368075	0.173746132	0.56290201	0.246841738
Johnsonella ignava	0.014380217	0.097778971	0.028760434	0	0.161691678	0.561061286	0.227445627
Johnsonella sp. HMT-166	0.019027658	0.098029961	0.035242852	0.002812465	0.301928049	0.600118256	0.170513034
Kingella denitrificans	0.181993728	0.513993687	0.100855406	0.263132049	0.913565578	1	0.078730439
Kingella oralis	0.175683892	0.222203709	0.199953888	0.151413896	0.280842772	0.600118256	0.242643533
Kingella sp. HMT-012	0.000364943	0.001760666	0.000325826	0.00040406	1	1	0.101417864
Kingella sp. HMT-459	0.002102466	0.014413779	0	0.004204932	0.3378947	0.600118256	-0.068439698
Kingella sp. HMT-932	0.000845925	0.00302131	0.001351492	0.000340358	0.301928049	0.600118256	0.110391905
Kocuria kristinae	0.000124916	0.000856384	0.000249833	0	0.3378947	0.600118256	0.110556435
Lachnoanaerobaculum orale	5.1835E-05	0.000355363	0.00010367	0	0.3378947	0.600118256	0.236906645
Lachnoanaerobaculum saburreum	0.012503823	0.034901633	0.020407945	0.0045997	0.170895935	0.56290201	0.174799617
Lachnoanaerobaculum sp. HMT-083	0.002477711	0.012672062	0.004955421	0	0.161691678	0.561061286	0.106120393
Lachnoanaerobaculum sp. HMT-089	0.000492176	0.002061581	0.000769275	0.000215076	0.53905226	0.77618636	0.129609053
Lachnoanaerobaculum sp. HMT-496	0.003953591	0.016732676	0.006432268	0.001474914	0.53905226	0.77618636	-0.016039444
Lachnospiraceae G-2 sp. HMT-088	0.005361288	0.036755137	0	0.010722575	0.3378947	0.600118256	-0.068439698
Lachnospiraceae G-2 sp. HMT-096	0.00019803	0.000954616	0.000396061	0	0.161691678	0.561061286	0.18653363
Lachnospiraceae G-3 sp. HMT-100	0.018411726	0.067159635	0.023877016	0.012946436	0.741894531	0.885532006	-0.159516031

Species_OTUs	Mean abundance	Std_deviation	Mean abundance - health	Mean abundance - peri-implantitis	P value	FDR corrected P value	LDA correlation (CAP)
Lachnospiraceae G-7 sp. HMT-086	0.005909063	0.011364797	0.005346229	0.006471897	0.850718256	0.976864544	-0.21467487
Lachnospiraceae G-7 sp. HMT-163	0.003925339	0.014623281	0.002845432	0.005005246	0.461946801	0.717203469	0.045152004
Lachnospiraceae G-8 sp. HMT-500	0.018874605	0.067772348	0.028227331	0.009521879	0.095655351	0.482072601	0.196677462
Lactobacillus crispatus	1.67352E-05	0.000114731	3.34704E-05	0	0.3378947	0.600118256	0.236906645
Lactobacillus delbrueckii	0.338632113	2.302698456	0.005400516	0.67186371	0.605877247	0.791629736	-0.018885151
Lactobacillus delbrueckii subsp. bulgaricus	0.001088253	0.006384173	9.12381E-05	0.002085267	0.53905226	0.77618636	-0.157807429
Lactobacillus delbrueckii subsp. delbrueckii	0.000115799	0.000793875	0	0.000231597	0.3378947	0.600118256	-0.24743583
Lactobacillus delbrueckii subsp. lactis	0.006113353	0.040474907	0.000415244	0.011811461	0.605877247	0.791629736	-0.018885151
Lactobacillus delbrueckii subsp. sunkii	0.00173698	0.011908132	0	0.003473959	0.3378947	0.600118256	-0.24743583
Lactobacillus fermentum	0.025812535	0.089220052	0.031986959	0.019638112	0.989182828	1	0.015702701
Lactobacillus frumenti	0.000254391	0.001318795	0.000508781	0	0.161691678	0.561061286	0.330587752
Lactobacillus gasseri	0.801402866	3.070603864	0.386711532	1.2160942	0.429265794	0.679760048	0.196311912
Lactobacillus iners	0.000100387	0.000688219	0	0.000200774	0.3378947	0.600118256	-0.121085619
Lactobacillus oris	0.0743656	0.478405777	0.006935878	0.141795322	1	1	-0.046421211
Lactobacillus paracasei	0.0209605	0.130010039	0.038090086	0.003830914	0.322518942	0.600118256	0.15058588
Lactobacillus paracasei subsp. paracasei	0.001030944	0.005321959	0.001651433	0.000410455	0.572000957	0.787351719	0.071918796
Lactobacillus rhamnosus	0.031723394	0.128518358	0.016809766	0.046637021	0.62625238	0.804207575	-0.128371849
Lactobacillus salivarius	0.050904686	0.329500054	0.096945829	0.004863543	0.572000957	0.787351719	0.019143852
Lactobacillus ultunensis	0.003848517	0.024325723	0.000289004	0.00740803	0.53905226	0.77618636	-0.107619493
Lactobacillus vaginalis	0.175012298	1.102314749	0.009584426	0.34044017	0.74741	0.887507422	0.105268147
Lautropia mirabilis	0.481821482	1.148523722	0.487988421	0.475654542	0.150077946	0.561061286	0.297000075
Leptotrichia buccalis	0.051739895	0.161681332	0.050874485	0.052605304	0.644825294	0.820569263	0.13804102
Leptotrichia goodfellowii	0.018268224	0.098421417	0.034984689	0.001551759	0.59959757	0.791629736	0.111370983
Leptotrichia hofstadii	0.008376535	0.028963472	0.009446522	0.007306549	0.727682909	0.875148953	0.133423667
Leptotrichia hongkongensis	0.128071987	0.456405465	0.178013983	0.07812999	0.474166448	0.73259293	0.249370127
Leptotrichia shahii	0.002968441	0.018118389	0	0.005936881	0.161691678	0.561061286	-0.178852833
Leptotrichia sp. HMT-212	0.550577818	1.704592922	0.596481631	0.504674005	0.013021268	0.202666974	0.427801583
Leptotrichia sp. HMT-215	0.094143315	0.269904975	0.118351224	0.069935406	0.010308653	0.192529246	0.348671071
Leptotrichia sp. HMT-218	0.00020203	0.001385048	0	0.00040406	0.3378947	0.600118256	-0.057910513
Leptotrichia sp. HMT-219	0.040504542	0.150372216	0.058231446	0.022777637	0.811848786	0.947654373	0.038185504
Leptotrichia sp. HMT-221	0.01150644	0.028600221	0.013974857	0.009038024	0.092569838	0.477901196	0.260716031
Leptotrichia sp. HMT-223	0.085974412	0.310986735	0.047676782	0.124272043	0.697312448	0.852329638	-0.000660538
Leptotrichia sp. HMT-225	0.007338635	0.034434924	0.013741642	0.000935629	0.04567182	0.360813321	0.239768909
Leptotrichia sp. HMT-392	0.017823567	0.052302425	0.016378983	0.019268152	0.684690389	0.840964017	-0.070621068
Leptotrichia sp. HMT-417	0.198187218	1.003216477	0.060110381	0.336264055	0.59769524	0.791629736	0.018597056
Leptotrichia sp. HMT-463	0.001906824	0.007941442	0	0.003813647	0.081078758	0.443836303	-0.292849197
Leptotrichia sp. HMT-498	0.01790959	0.064706588	0.026592283	0.009226897	0.699312979	0.852329638	0.076740196
Leptotrichia trevisanii	0.196359294	0.735736618	0.236076913	0.156641675	0.682710228	0.840964017	-0.099310567
Leptotrichia wadei	0.276098444	1.13012531	0.239485002	0.312711885	0.110146684	0.530251708	0.409390732
Megasphaera micronuciformis	0.307260159	0.727980308	0.164317324	0.450202994	0.991709497	1	-0.070348413
Megasphaera sp. HMT-123	0.099375023	0.400967589	0	0.198750045	0.010294231	0.192529246	-0.4019284
Mogibacterium neglectum	0.000220463	0.001511419	0.000440926	0	0.3378947	0.600118256	0.236906645
Mogibacterium timidum	0.596183147	1.103511862	0.447686612	0.744679681	0.063371383	0.404944918	-0.281865956
Mollicutes G-2 sp. HMT-906	9.77173E-05	0.000669916	0.000195435	0	0.3378947	0.600118256	0.10002725
Mycoplasma faucium	0.011201391	0.033377293	0.006646545	0.015756238	0.660627721	0.827132464	0.050213208
Mycoplasma orale	0.000506562	0.003472812	0	0.001013123	0.3378947	0.600118256	-0.236906645
Mycoplasma salivarium	0.084213385	0.183655823	0.027576927	0.140849844	0.060645333	0.397008109	-0.245781581
Neisseria bacilliformis	0.062575965	0.14494785	0.059300501	0.06585143	0.850718256	0.976864544	0.030516858
Neisseria cinerea	0.033179484	0.150004371	0.033991441	0.032367528	0.654727339	0.82327101	-0.26368718
Neisseria elongata	0.204222588	0.485250237	0.298970107	0.109475068	0.05897057	0.394171705	0.197925147
Neisseria elongata subsp. glycolytica	0.006471618	0.025539188	0.004823605	0.00811963	0.290062846	0.600118256	0.21819678
Neisseria oralis	1.667744057	5.21212114	2.260625189	1.074862925	0.593067314	0.791629736	0.158267536
Neisseria polysaccharea	0.000160293	0.001098916	0.000320587	0	0.3378947	0.600118256	-0.152673171
Neisseria sicca	0.004795311	0.013422593	0.004687198	0.004903424	0.187439418	0.583451129	0.239044871
Neisseria sp. HMT-018	0.001526228	0.005388346	0.001840276	0.00121218	0.344004795	0.600118256	0.129639724
Neisseria subflava	0.005605011	0.031462736	0.010796354	0.000413668	0.091680174	0.477187792	0.362916313
Fusobacterium sp. OTU_10	3.685639079	4.01769415	2.877169508	4.49410865	0.230034676	0.600118256	-0.440946591

Species_OTUs	Mean abundance	Std_deviation	Mean abundance - health	Mean abundance - peri-implantitis	P value	FDR corrected P value	LDA correlation (CAP)
Capnocytophaga sp. OTU_1010	7.89141E-05	0.000541008	0	0.000157828	0.3378947	0.600118256	-0.236906645
Granulicatella sp. OTU_1014	0.004644457	0.010357068	0.006932989	0.002355925	0.137965362	0.561061286	0.29981059
Peptostreptococcus sp. OTU_1016	0.00316562	0.01239793	0.000946017	0.005385222	0.360154597	0.61810316	-0.228404905
Prevotella sp. OTU_102	0.011444036	0.032639556	0.020522236	0.002365836	0.012488769	0.202666974	0.383373857
Fusobacterium sp. OTU_103	0.877618796	1.239887911	1.064521713	0.690715879	0.183481379	0.57687949	0.187710347
Porphyromonas sp. OTU_1037	0.003947683	0.021608758	0.000512436	0.007382929	0.373797556	0.638068408	-0.242807382
Treponema sp. OTU_105	0.013307077	0.064082119	0.014492354	0.0121218	1	1	0.017085853
Gemella sp. OTU_1053	0.000157828	0.001082016	0	0.000315657	0.3378947	0.600118256	-0.236906645
Sphaerochaeta sp. OTU_106	0.011107477	0.05903799	0.002984492	0.019230463	0.388162871	0.653802182	0.058661779
Streptococcus sp. OTU_1061	0.000432969	0.00161689	0.000865939	0	0.041041673	0.35218192	0.27773471
Streptococcus sp. OTU_1063	0.534009012	0.592046727	0.553948191	0.514069834	0.219746523	0.600118256	0.336988991
Bifidobacterium sp. OTU_1065	6.84092E-05	0.00046899	0	0.000136818	0.3378947	0.600118256	-0.078968882
Streptococcus sp. OTU_1070	0.01106132	0.021890645	0.011548538	0.010574102	0.832600302	0.964783197	0.204280248
Limosilactobacillus sp. OTU_1071	6.19818E-05	0.000424926	0.000123964	0	0.3378947	0.600118256	0.194789908
Neisseriaceae gen. sp. OTU_1079	0.000424031	0.001303313	0.000672697	0.000175365	0.179721247	0.573482372	0.295214995
Selenomonadaceae gen. sp. OTU_108	0.030297259	0.092021217	0.026199397	0.034395122	0.217722456	0.600118256	-0.160877405
Prevotella sp. OTU_1082	0.001528459	0.007387638	0	0.003056919	0.041041673	0.35218192	-0.275130593
Saccharibacteria sp. OTU_1088	0	0	0	0	NA	NA	0
Leptotrichia sp. OTU_1089	0	0	0	0	NA	NA	0
Leptotrichia sp. OTU_1090	0	0	0	0	NA	NA	0
Leptotrichia sp. OTU_1091	0	0	0	0	NA	NA	0
Cardiobacterium sp. OTU_1092	0.000107538	0.000737243	0	0.000215076	0.3378947	0.600118256	-0.026322961
Fusobacterium sp. OTU_1093	0.000107538	0.000737243	0	0.000215076	0.3378947	0.600118256	-0.026322961
Lautropia sp. OTU_1094	3.1076E-05	0.000213046	6.21519E-05	0	0.3378947	0.600118256	0.184260724
Capnocytophaga sp. OTU_1095	0.00069458	0.002503556	0.000451026	0.000938134	0.344004795	0.600118256	-0.059441795
Gemella sp. OTU_1097	0.00568241	0.024786237	0.010128969	0.001235852	0.070235111	0.433002868	0.223808861
Peptostreptococcus sp. OTU_1098	0.001008538	0.004514075	0	0.002017075	0.081078758	0.443836303	-0.300610218
Cardiobacterium sp. OTU_1099	0.000107538	0.000737243	0	0.000215076	0.3378947	0.600118256	-0.026322961
Streptococcus sp. OTU_11	0.2500777	0.5572442	0.181156001	0.318999399	0.480610421	0.739382515	-0.090219837
Porphyromonas sp. OTU_1101	0.000445227	0.001747787	0.000185334	0.00070512	0.9485568	1	-0.042005535
Bacteroidales gen. sp. OTU_111	0.00756106	0.040610572	0.011779409	0.00334271	0.572000957	0.787351719	0.066486081
Fusobacterium sp. OTU_1122	0.000553104	0.003139661	0.000895961	0.000210247	1	1	-0.028685424
Abiotrophia sp. OTU_1127	0.002035018	0.006208575	0.003265917	0.000804119	0.427142164	0.678088185	0.229513176
Haemophilus sp. OTU_113	0.022393986	0.115563006	0.041616591	0.00317138	0.838849019	0.968489321	-0.083652573
Neisseria sp. OTU_1132	0.130091398	0.342096318	0.132900474	0.127282322	0.488080958	0.742824985	0.068581023
Streptococcus sp. OTU_1133	1.565202429	2.469667498	2.160419784	0.969985074	0.064825243	0.407564647	0.368849843
Gemella sp. OTU_1135	0.000900389	0.00248573	0.000562197	0.001238581	0.62625238	0.804207575	-0.064009104
Porphyromonas sp. OTU_1136	0.004561915	0.01547046	0.001173655	0.007950175	0.589109676	0.791629736	-0.300028848
Streptococcus sp. OTU_1137	8.55157E-05	0.000586266	0.000171031	0	0.3378947	0.600118256	0.078968882
Prevotella sp. OTU_1139	0.000561708	0.003606328	7.2289E-05	0.001051127	1	1	-0.161139976
Neisseria sp. OTU_1144	0.000512476	0.002729222	0.000818118	0.000206834	0.572000957	0.787351719	0.244989567
Streptococcus sanguinis OTU_1147	2.24691E-05	0.00015404	4.49382E-05	0	0.3378947	0.600118256	0.184260724
Neisseriaceae gen. sp. OTU_1149	0.000370272	0.001472721	0.000325865	0.000414679	0.605877247	0.791629736	-0.020696056
Prevotella sp. OTU_1152	0.000326609	0.001568188	0.000342063	0.000311154	1	1	-0.041852503
Fudania sp. OTU_1153	0.000218377	0.001047425	0.000221678	0.000215076	1	1	0.085272516
Streptococcus sp. OTU_1155	0.000279973	0.001379829	0.00034487	0.000215076	1	1	-0.022258635
Pseudoramibacter sp. OTU_1156	0.001051127	0.007206162	0	0.002102254	0.3378947	0.600118256	-0.215848277
Saccharibacteria sp. OTU_1157	0.014511344	0.097245254	0.000171031	0.028851658	0.301928049	0.600118256	-0.272073586
Prevotella sp. OTU_1158	0.002719422	0.014523824	0	0.005438844	0.041041673	0.35218192	-0.366954013
Streptococcus sp. OTU_1159	0.000185382	0.001124197	0.000370764	0	0.161691678	0.561061286	0.278703188
Lachnospiraceae gen. sp. OTU_116	0.002134625	0.007457788	0.000524226	0.003745023	0.282231305	0.600118256	-0.219764807
Neisseriaceae gen. sp. OTU_1160	0.000480696	0.002282011	0.000961392	0	0.081078758	0.443836303	0.130902556
Porphyromonas sp. OTU_1164	4.54301E-05	0.000311453	9.08602E-05	0	0.3378947	0.600118256	-0.005264592
Dialister sp. OTU_1165	0.001824294	0.004251083	0.000338633	0.003309955	0.048342471	0.360813321	-0.290003424
Asteroleplasma sp. OTU_1166	0	0	0	0	NA	NA	0
Porphyromonas sp. OTU_1168	0.000103417	0.000708991	0	0.000206834	0.3378947	0.600118256	0.057910513
Lachnoanaerobaculum sp. OTU_117	0.026461358	0.100824186	0.041524375	0.011398341	0.179344637	0.573482372	0.208632603

Species_OTUs	Mean abundance	Std_deviation	Mean abundance - health	Mean abundance - peri-implantitis	P value	FDR corrected P value	LDA correlation (CAP)
Lactobacillus sp. OTU_118	0.001455047	0.007466018	0.002078484	0.00083161	0.605877247	0.791629736	-0.122624134
Porphyromonadaceae gen. sp. OTU_119	0.011964789	0.061890741	0.019244989	0.004684588	0.982841692	1	0.026267612
Bacteroidales gen. sp. OTU_120	0.001083369	0.003586778	0.000163149	0.002003589	0.144764517	0.561061286	-0.202883537
Schaalia sp. OTU_122	0.019579932	0.092194145	0.030248838	0.008911026	0.333959585	0.600118256	-0.017986994
Catonella sp. OTU_126	0.003052667	0.013962754	0.000441336	0.005663999	0.572844873	0.787351719	-0.008803221
Erysipelotrichaceae gen. sp. OTU_127	0.006910927	0.028828078	0.011915892	0.001905962	0.280949564	0.600118256	0.240465473
Lachnospiraceae gen. sp. OTU_128	0.001425553	0.008344373	0.000210958	0.002640147	0.53905226	0.77618636	-0.122365433
Streptococcus sp. OTU_13	0.713170629	1.577928152	0.521961482	0.904379775	0.074704208	0.443836303	0.320130948
Propionivibrio sp. OTU_130	0.006416144	0.01583414	0.009886999	0.002945288	0.154666319	0.561061286	0.182885072
Campylobacter sp. OTU_134	0.005834128	0.038389433	0.011199513	0.000468744	1	1	-0.013950834
Schaalia sp. OTU_14	0.22069017	0.393911474	0.24718792	0.19419242	0.053082778	0.368944353	0.479854445
Actinomyces sp. OTU_141	0.19473449	0.317738473	0.250469827	0.138999153	0.173692104	0.56290201	0.214753123
Brachymonas sp. OTU_142	0.018382409	0.039912379	0.030673937	0.006090881	0.056260228	0.383388493	0.272977936
Centipeda sp. OTU_143	0.004049707	0.009688732	0.001436466	0.006662949	0.202584403	0.600118256	-0.273048471
Lacticaseibacillus sp. OTU_15	0.345895777	1.493740219	0.239990168	0.451801386	0.464253805	0.719027235	-0.030958761
Selenomonas sp. OTU_155	0.006201124	0.03216233	0.002771387	0.009630861	0.68304078	0.840964017	0.021496238
Arachnia sp. OTU_16	0.256707096	0.766549435	0.220217874	0.293196318	0.339071074	0.600118256	0.183398929
Selenomonadaceae gen. sp. OTU_160	0.037568614	0.155774454	0.014823143	0.060314086	0.608891701	0.792307849	-0.068239406
Aggregatibacter sp. OTU_165	0.00611304	0.034729522	0.01222608	0	0.02067882	0.27356356	0.186300725
Prevotella sp. OTU_166	0.000720653	0.004303074	0.001441307	0	0.161691678	0.561061286	0.0907588
Bacteroidales gen. sp. OTU_167	0.005516275	0.019885368	0.003473593	0.007558958	0.699312979	0.852329638	0.013084734
Saccharibacteria sp. OTU_168	0.003257242	0.013975966	0.002877944	0.00363654	0.344004795	0.600118256	0.147755318
Schwartzia sp. OTU_172	0.008083776	0.028800282	0.000786559	0.015380992	0.009629385	0.192529246	-0.328219032
Dialister sp. OTU_173	0.000390317	0.00267588	0	0.000780634	0.3378947	0.600118256	-0.236906645
Lachnoanaerobaculum sp. OTU_175	0.002867583	0.015609157	0.005187893	0.000547273	0.322518942	0.600118256	0.177419605
Peptoanaerobacter sp. OTU_178	0.007669495	0.051235744	0.000388771	0.01495022	1	1	0.013010329
Alloprevotella sp. OTU_183	0.002222441	0.010598261	0.001616463	0.00282842	0.605877247	0.791629736	0.127022046
Lachnoanaerobaculum sp. OTU_184	0.011027914	0.072087624	0.021723532	0.000332297	0.301928049	0.600118256	0.152170995
Granulicatella sp. OTU_19	0.093847521	0.150531196	0.15118354	0.036511501	0.013085584	0.202666974	0.449678769
Prevotella sp. OTU_194	0.000771036	0.004235138	0.001542072	0	0.161691678	0.561061286	0.187160633
Neisseria sp. OTU_2	1.336458126	2.181997916	1.67925996	0.993656292	0.022255779	0.280880546	0.561091506
Prevotella sp. OTU_20	0.183728534	0.616754859	0.012053403	0.355403665	0.120068522	0.55652198	-0.164280903
Filifactor sp. OTU_21	0.167601748	0.430504877	0.273074267	0.062129229	0.019448687	0.262764177	0.325810544
Lachnospiraceae gen. sp. OTU_211	0.007317852	0.037665213	0.010109179	0.004526525	1	1	0.054235828
Eggerthia sp. OTU_22	0.037588829	0.094476352	0.060104565	0.015073093	0.165538514	0.562537401	0.175748426
Selenomonas sp. OTU_225	0.002806508	0.018936867	8.73955E-05	0.00552562	1	1	0.033544703
Caviibacter sp. OTU_23	0.069513195	0.476558456	0.13902639	0	0.3378947	0.600118256	0.10002725
Desulfovibrio sp. OTU_24	0.087303492	0.377868257	0.004308183	0.170298801	0.195979497	0.600118256	-0.091946779
Prevotella sp. OTU_242	0.001801833	0.012175987	0.003603665	0	0.161691678	0.561061286	0.200327713
Prevotella sp. OTU_243	0.016874789	0.115687725	0	0.033749578	0.3378947	0.600118256	-0.047381329
Capnocytophaga sp. OTU_249	0.009300779	0.032059696	0.009210131	0.009391428	0.684204921	0.840964017	0.061740167
Limosilactobacillus sp. OTU_259	0.001131546	0.006475595	0.000410313	0.001852778	1	1	-0.014734589
Actinomyces sp. OTU_26	0.177335469	0.437064718	0.29054945	0.064121488	0.015733615	0.227099741	0.425952937
Prevotella sp. OTU_269	0.002565227	0.015580022	0.000613858	0.004516595	1	1	0.028685424
Haemophilus sp. OTU_27	0.007649187	0.019246815	0.009829574	0.005468801	0.400551954	0.658991323	-0.042142328
Fretibacterium sp. OTU_282	0.000868641	0.005268579	0	0.001737281	0.161691678	0.561061286	-0.166939761
Porphyromonas sp. OTU_283	0	0	0	0	NA	NA	0
Saccharibacteria sp. OTU_288	0.000431614	0.002288299	0	0.000863229	0.161691678	0.561061286	-0.215689306
Rothia sp. OTU_289	0.002755545	0.018767931	0.00551109	0	0.161691678	0.561061286	0.201581721
Campylobacter sp. OTU_29	0.161853791	0.399610364	0.129417343	0.19429024	0.339213183	0.600118256	0.182830702
Lachnoanaerobaculum sp. OTU_292	0.003719852	0.016350557	0.002322354	0.005117349	0.098900014	0.494500071	0.236719409
Streptococcus sp. OTU_3	1.121744181	1.746056978	1.581473232	0.66201513	0.048908253	0.360813321	0.391271308
Limosilactobacillus sp. OTU_30	0.194963379	1.331921744	0.388587692	0.001339066	1	1	-0.004075525
Saccharibacteria sp. OTU_302	0.000971507	0.004806054	0.000730834	0.00121218	1	1	0.013010329
Peptostreptococcus sp. OTU_303	0.001047526	0.004075075	0.001314417	0.000780634	0.605877247	0.791629736	-0.173070771
Schaalia sp. OTU_304	0.000756508	0.004614044	0.001513017	0	0.161691678	0.561061286	0.140135349
Streptococcus sp. OTU_315	5.77357E-05	0.000395816	0.000115471	0	0.3378947	0.600118256	0.110556435

Species_OTUs	Mean abundance	Std_deviation	Mean abundance - health	Mean abundance - peri-implantitis	P value	FDR corrected P value	LDA correlation (CAP)
Kingella sp. OTU_325	0.011260537	0.072148729	0.021308894	0.00121218	0.322518942	0.600118256	0.211839234
Veillonellaceae gen. sp. OTU_33	0.191970214	0.797253109	0.000647198	0.38329323	0.050819512	0.366708982	-0.318973425
Anaerocella sp. OTU_337	0.00101015	0.006925239	0	0.0020203	0.3378947	0.600118256	-0.057910513
Haemophilus sp. OTU_34	0.023517397	0.119852929	0.007516749	0.039518045	0.961998777	1	-0.022073398
Corynebacterium sp. OTU_346	0.000103417	0.000708991	0	0.000206834	0.3378947	0.600118256	0.057910513
Treponema sp. OTU_357	0.001169758	0.006799628	0.002339516	0	0.081078758	0.443836303	0.225328313
Capnocytophaga sp. OTU_36	0.019409815	0.111572729	0.037915798	0.000903832	0.626901968	0.804207575	-0.067150152
Bacteroidaceae gen. sp. OTU_37	0.026792278	0.122764509	0.046954866	0.00662969	0.167432392	0.562537401	0.146379141
Streptococcus sp. OTU_38	0.472056958	0.792384741	0.885632376	0.05848154	3.22289E-05	0.013416213	0.63744986
Erysipelotrichaceae gen. sp. OTU_388	0.000631824	0.004331565	0.001263647	0	0.3378947	0.600118256	0.089498066
Prevotella sp. OTU_39	0.125223476	0.286257582	0.190988513	0.059458439	1	1	0.015993036
Veillonellaceae gen. sp. OTU_391	0	0	0	0	NA	NA	0
Oribacterium sp. OTU_394	0.000255078	0.000998552	0.000510157	0	0.081078758	0.443836303	0.218602095
Streptococcus sp. OTU_398	0.001655594	0.009604325	0.003096112	0.000215076	0.322518942	0.600118256	0.190326966
Tannerella sp. OTU_399	0.002762109	0.008367611	0.000360926	0.005163292	0.047336187	0.360813321	-0.477423992
Streptococcus sp. OTU_4	0.957204321	1.992051511	1.817331553	0.097077089	0.000135813	0.028747119	0.672620688
Phocaeicola sp. OTU_40	0.021223788	0.054741488	0.028296664	0.014150913	0.763028513	0.898929696	0.073143117
Lautropia sp. OTU_400	0.001006433	0.004531397	0.001806032	0.000206834	0.301928049	0.600118256	0.130885171
Acidimicrobiales gen. sp. OTU_403	0.000842432	0.00577542	0.001684863	0	0.3378947	0.600118256	0.089498066
Eikenella sp. OTU_434	0.003022553	0.006657529	0.002044045	0.00400106	0.644738522	0.820569263	-0.190170851
Campylobacter sp. OTU_44	0.027473555	0.138432883	0	0.054947109	0.081078758	0.443836303	-0.291555693
Saccharibacteria sp. OTU_448	0.000315912	0.002165783	0.000631824	0	0.3378947	0.600118256	0.089498066
Prevotella sp. OTU_45	0.04572016	0.151799323	0	0.09144032	0.041041673	0.35218192	-0.368312683
Neisseria sp. OTU_457	0.002637791	0.007259902	0.00303415	0.002241432	0.820351346	0.95582221	0.043199299
Porphyromonas sp. OTU_46	0.035093804	0.109281833	0.035473827	0.034713782	0.726292725	0.875134498	0.031698392
Catonella sp. OTU_460	0.000284442	0.0016614	9.08602E-05	0.000478025	1	1	-0.099693604
Treponema sp. OTU_47	0.058411649	0.174730511	0.079507478	0.03731582	0.247857501	0.600118256	0.13681339
Mogibacterium sp. OTU_48	0.040465731	0.061589487	0.042216294	0.038715169	0.424679021	0.675867614	0.042701279
Sphingobacteriaceae gen. sp. OTU_496	0.000266207	0.001825021	0.000532413	0	0.3378947	0.600118256	0.152673171
Rothia sp. OTU_5	1.24923843	3.105510168	2.333142886	0.165333974	0.009231595	0.192529246	0.477675975
Lactobacillus sp. OTU_50	0.000234372	0.001606774	0	0.000468744	0.3378947	0.600118256	-0.047381329
Capnocytophaga sp. OTU_52	0.122058346	0.493878723	0.175071103	0.069045589	0.7297626	0.875991022	0.235812809
Oribacterium sp. OTU_527	0.000158693	0.000809639	0.00010231	0.000215076	1	1	0.028685424
Limosilactobacillus sp. OTU_528	3.09909E-05	0.000212463	6.19818E-05	0	0.3378947	0.600118256	0.194789908
Cryptobacterium sp. OTU_53	0.048263131	0.185545965	0.01186629	0.084659971	0.245152863	0.600118256	-0.030666351
Neisseriaceae gen. sp. OTU_531	0.000384859	0.001176457	0.000619188	0.00015053	0.167432392	0.562537401	0.127544343
Fudania sp. OTU_532	5.63154E-05	0.000386079	0	0.000112631	0.3378947	0.600118256	-0.089498066
Porphyromonas sp. OTU_538	0.00205282	0.007676224	0.000579095	0.003526546	0.391102978	0.654264335	-0.21621897
Peptococcus sp. OTU_54	0.141370496	0.304562251	0.089843476	0.192897517	0.887683342	1	-0.172098266
Selenomonas sp. OTU_58	0.090136124	0.230004812	0.043877704	0.136394544	0.067161479	0.418113127	-0.398182923
Saccharibacteria sp. OTU_591	0.000315912	0.002165783	0.000631824	0	0.3378947	0.600118256	0.089498066
Streptococcus sp. OTU_598	0.007219876	0.032193808	0.011999017	0.002440734	0.494875601	0.746427568	0.062771172
Prevotella sp. OTU_599	0.000415157	0.002197965	0	0.000830313	0.161691678	0.561061286	-0.30879937
Rothia sp. OTU_6	0.607619852	1.881687861	0.535371555	0.679868149	0.256322456	0.600118256	0.232656942
Corynebacterium sp. OTU_60	0.030227116	0.089950367	0.033619502	0.02683473	0.227460026	0.600118256	0.348657839
Eubacterium sp. OTU_600	0.0022451	0.005503696	0.000654252	0.003835948	0.02266284	0.280880546	-0.171583765
Rothia sp. OTU_601	0.001160736	0.003290936	0.002321471	0	0.005039521	0.192529246	0.444880955
Eubacterium sp. OTU_61	0.059909797	0.14643137	0.019118234	0.100701361	1	1	0.118456768
Schwartzia sp. OTU_63	0.040161976	0.087649319	0.039804788	0.040519163	0.540634305	0.776703131	0.13154055
Rothia sp. OTU_633	0.00318895	0.00620869	0.005382368	0.000995531	0.007691626	0.192529246	0.398350623
Saccharibacteria sp. OTU_643	4.73438E-05	0.000324573	9.46876E-05	0	0.3378947	0.600118256	0.236906645
Treponema sp. OTU_65	0.044021491	0.154778073	0.01723348	0.070809503	0.972789109	1	-0.154795706
Abiotrophia sp. OTU_650	0.004570143	0.014619807	0.006601604	0.002538682	0.310416155	0.600118256	0.338580487
Desulfovibrio sp. OTU_654	0.000304423	0.002087019	0.000608846	0	0.3378947	0.600118256	0.110556435
Selenomonas sp. OTU_669	0.000375144	0.001545475	0.000189303	0.000560985	0.9485568	1	0.086049074
Lachnoanaerobaculum sp. OTU_67	0.047123503	0.112552127	0.062364939	0.031882068	0.725389404	0.875134498	0.081255714
Parvimonas sp. OTU_69	0.000610384	0.002955919	0.000308725	0.000912042	0.572000957	0.787351719	-0.148752905

Species_OTUs	Mean abundance	Std_deviation	Mean abundance - health	Mean abundance - peri-implantitis	P value	FDR corrected P value	LDA correlation (CAP)
Centipeda sp. OTU_695	0.000320597	0.001015031	0.000192515	0.00044868	0.626901968	0.804207575	-0.027842746
Veillonella sp. OTU_7	2.867555137	4.122754359	3.917618351	1.817491923	0.1195118	0.55652198	0.280348468
Porphyromonas sp. OTU_70	0.023618827	0.127779366	0.000593093	0.046644562	0.301928049	0.600118256	-0.204479774
Bifidobacterium sp. OTU_700	6.65517E-05	0.000456255	0.000133103	0	0.3378947	0.600118256	0.152673171
Saccharibacteria sp. OTU_701	0.000604478	0.00363207	0	0.001208955	0.161691678	0.561061286	-0.323220457
Streptococcus sp. OTU_702	0	0	0	0	NA	NA	0
Cardiobacterium sp. OTU_706	0	0	0	0	NA	NA	0
Tannerella sp. OTU_716	0.002861158	0.008590583	0	0.005722316	0.005039521	0.192529246	-0.421010156
Prevotella sp. OTU_72	0.02457605	0.121926749	0.00281415	0.04633795	0.207848174	0.600118256	-0.222440477
Alloprevotella sp. OTU_722	0.001907289	0.006200603	0.000178256	0.003636323	0.173746132	0.56290201	-0.24259804
Porphyromonas sp. OTU_726	0.002105544	0.007734915	0.000345822	0.003865266	0.173746132	0.56290201	-0.241006654
Bacteroidales gen. sp. OTU_727	0.000403545	0.001959932	0	0.00080709	0.161691678	0.561061286	-0.232618409
Alloprevotella sp. OTU_728	0.000356613	0.002444816	0.000713226	0	0.3378947	0.600118256	0.215848277
Selenomonas sp. OTU_73	0.042760149	0.159285665	0.003431129	0.082089169	0.094976298	0.482072601	-0.286946668
Neisseria sp. OTU_735	0.000806426	0.002926315	0.000992351	0.000620501	0.344004795	0.600118256	0.239352294
Saccharibacteria sp. OTU_737	0.000133103	0.00091251	0.000266207	0	0.3378947	0.600118256	0.152673171
Prevotellaceae gen. sp. OTU_751	0	0	0	0	NA	NA	0
Cardiobacterium sp. OTU_76	0.031950483	0.21509616	0.063900966	0	0.161691678	0.561061286	0.227445627
Neisseria sp. OTU_77	0.019006341	0.040129676	0.031932023	0.00608066	0.026535379	0.30089224	0.436414279
Saccharibacteria sp. OTU_778	0.000315912	0.002165783	0.000631824	0	0.3378947	0.600118256	0.089498066
Lactobacillaceae gen. sp. OTU_789	0	0	0	0	NA	NA	0
Olsenella sp. OTU_79	0.013492979	0.090919814	0.026985957	0	0.161691678	0.561061286	-0.034014956
Clostridiales XIII gen. sp. OTU_798	0.001075518	0.004806502	0.001019405	0.001131631	0.605877247	0.791629736	0.105549887
Streptococcus sp. OTU_8	1.239551124	3.197491359	1.223008358	1.256093889	0.448959137	0.702189783	0.249758792
Selenomonas sp. OTU_80	0.127463544	0.286988218	0.060418644	0.194508445	0.145969514	0.561061286	-0.27850226
Fudania sp. OTU_81	0.008382655	0.030980589	0.009060838	0.007704473	0.275021908	0.600118256	0.073911065
Leptotrichia sp. OTU_83	0.043136548	0.187645306	0.047483336	0.03878976	0.179721247	0.573482372	0.220694705
Ligilactobacillus sp. OTU_86	0.020737891	0.094566044	0.040086199	0.001389584	0.282231305	0.600118256	0.147075984
Capnocytophaga sp. OTU_862	0	0	0	0	NA	NA	0
Capnocytophaga sp. OTU_873	8.01467E-05	0.000549458	0.000160293	0	0.3378947	0.600118256	-0.152673171
Porphyromonas sp. OTU_88	0.011392102	0.061396154	0.005409625	0.01737458	0.605877247	0.791629736	0.127022046
Lancefieldella sp. OTU_889	0.001308101	0.005032077	9.02052E-05	0.002525996	0.144764517	0.561061286	-0.318349042
Selenomonas sp. OTU_89	0.009033606	0.026001071	0.012040133	0.006027078	0.363353466	0.621912266	0.092519298
Prevotella sp. OTU_892	0.001784201	0.00583542	0	0.003568401	0.005039521	0.192529246	-0.478653714
Prevotella sp. OTU_894	0.00437758	0.012013718	0.00018041	0.00857475	0.004033528	0.192529246	-0.545596939
Fusobacterium sp. OTU_895	0.007362018	0.012365151	0.005506863	0.009217173	0.504227262	0.755932436	-0.106873517
Eubacterium sp. OTU_896	0.001500622	0.004534651	0.000127005	0.002874239	0.035801554	0.349753641	-0.337727593
Lactobacillales gen. sp. OTU_897	0.002375083	0.007965045	0.003128133	0.001622034	0.484676702	0.741613748	0.149915164
Leptotrichia sp. OTU_898	0.001230696	0.004254239	0.001210648	0.001250745	0.41942732	0.674183698	0.221981607
Gemella sp. OTU_9	0.571065024	1.901835815	0.912967668	0.229162381	0.060015389	0.396976789	0.373464145
Campylobacter sp. OTU_901	0.000110241	0.000564337	6.99516E-05	0.00015053	1	1	0.048279292
Prevotella sp. OTU_903	0.002390977	0.0094075	0	0.004781955	0.010294231	0.192529246	-0.487226013
Lactobacillales gen. sp. OTU_906	0.000551585	0.003480499	0.001103171	0	0.161691678	0.561061286	0.155810444
Eggerthellaceae gen. sp. OTU_907	0.000105304	0.000721928	0.000210608	0	0.3378947	0.600118256	0.089498066
Streptococcus sp. OTU_908	0.004619985	0.017178524	0.007862075	0.001377894	0.03176679	0.325353415	0.354403008
Porphyromonas sp. OTU_909	0.003398155	0.011694563	0.000430243	0.006366067	0.110150879	0.530251708	-0.292294997
Capnocytophaga sp. OTU_910	0.000103417	0.000708991	0	0.000206834	0.3378947	0.600118256	0.057910513
Lancefieldella sp. OTU_936	0.000654662	0.003208026	0.000520183	0.000789141	1	1	-0.06599215
Porphyromonadaceae gen. sp. OTU_95	0.01351673	0.075418276	0	0.02703346	0.161691678	0.561061286	-0.308172366
Selenomonas sp. OTU_97	0.000275302	0.001358226	0	0.000550605	0.161691678	0.561061286	-0.182301354
Selenomonas sp. OTU_99	0.060707469	0.240785443	0.004850434	0.116564503	0.419069484	0.674183698	-0.148444972
Olsenella profusa	0.002398911	0.010418672	0.002534558	0.002263263	0.605877247	0.791629736	0.034148493
Olsenella scatoligenes	0.00030937	0.002120933	0.00061874	0	0.3378947	0.600118256	-0.152673171
Olsenella sp. HMT-807	0.117256946	0.269649892	0.065694491	0.168819402	0.983098667	1	-0.004405297
Olsenella sp. HMT-809	0.000351644	0.001848019	0.000197379	0.000505909	1	1	0.121481986
Olsenella sp. HMT-939	0.000107538	0.000737243	0	0.000215076	0.3378947	0.600118256	-0.026322961
Olsenella uli	0.046628227	0.114715114	0.03812033	0.055136125	0.025993714	0.30089224	-0.431126174

Species_OTUs	Mean abundance	Std_deviation	Mean abundance - health	Mean abundance - peri-implantitis	P value	FDR corrected P value	LDA correlation (CAP)
Oribacterium asaccharolyticum	0.016880161	0.042532446	0.029209335	0.004550987	0.001823457	0.144736913	0.523855936
Oribacterium parvum	0.000855365	0.002892713	0.001710731	0	0.041041673	0.35218192	0.235389507
Oribacterium sinus	0.020073132	0.052625429	0.036805417	0.003340847	0.015736045	0.227099741	0.497049746
Oribacterium sp. HMT-078	0.259709503	0.460317946	0.205111675	0.31430733	0.436630728	0.689702767	-0.124278752
Oribacterium sp. HMT-102	0.011385037	0.053936551	0.001895689	0.020874385	0.650245177	0.82252129	-0.053223039
Ottowia sp. HMT-894	0.183352336	0.62048793	0.130492156	0.236212515	0.74741	0.887507422	-0.036858595
Parascardovia denticolens	0.000205514	0.001015519	0.000255451	0.000155577	1	1	0.019593869
Parvimonas micra	1.155290198	1.501023598	0.817596668	1.492983728	0.030266661	0.325353415	-0.443443335
Parvimonas sp. HMT-110	0.121799568	0.332654031	0.069672979	0.173926157	0.588711706	0.791629736	-0.176164817
Parvimonas sp. HMT-393	0.004263353	0.013494289	0.005867423	0.002659284	0.14642191	0.561061286	0.060171635
Peptidiphaga sp. HMT-183	0.134958101	0.514348364	0.196257416	0.073658786	0.165351457	0.562537401	0.339146839
Peptoanaerobacter stomatis	0	0	0	0	NA	NA	0
Peptococcus niger	0.005402704	0.033333572	0.001122054	0.009683354	1	1	-0.271962897
Peptococcus sp. HMT-168	0.002043293	0.009140347	0.003442413	0.000644173	0.301928049	0.600118256	0.084690405
Peptoniphilaceae G-1 sp. HMT-113	0.193177137	0.866912996	0.143130148	0.243224127	0.551551039	0.787351719	0.076839255
Peptoniphilus sp. HMT-187	5.43242E-05	0.000372428	0.000108648	0	0.3378947	0.600118256	0.068439698
Peptoniphilus sp. HMT-375	0.000333061	0.001339936	0.000464385	0.000201737	0.572000957	0.787351719	-0.151857313
Peptostreptococcaceae XI G-1 sp. HMT-383	0.003716991	0.010097813	0.000813815	0.006620168	0.123700848	0.561061286	-0.397805615
Peptostreptococcaceae XI G-2 sp. HMT-091	0.044000876	0.107933311	0.027565546	0.060436206	0.648838699	0.82252129	-0.097861846
Peptostreptococcaceae XI G-3 sp. HMT-495	0.000410639	0.001889331	0.000322834	0.000498445	0.605877247	0.791629736	0.008278423
Peptostreptococcaceae XI G-4 sp. HMT-369	0.1736038	0.401809048	0.10275591	0.244451691	0.310630897	0.600118256	-0.194480317
Peptostreptococcaceae XI G-7 sp. HMT-081	0.00020203	0.001385048	0	0.00040406	0.3378947	0.600118256	-0.057910513
Peptostreptococcaceae XI G-7 sp. HMT-922	1.80723E-05	0.000123897	3.61445E-05	0	0.3378947	0.600118256	-0.005264592
Peptostreptococcus anaerobius	0.005541432	0.022930102	0.007885665	0.003197198	0.572000957	0.787351719	-0.171001165
Peptostreptococcus stomatis	0.443651131	0.962390905	0.168579391	0.718722871	0.974525323	1	-0.083497251
Phocaeicola abscessus	0.000328394	0.002251358	0.000656789	0	0.3378947	0.600118256	0.110556435
Porphyromonas asaccharolytica	0.002799467	0.0178334	0	0.005598933	0.161691678	0.561061286	-0.246725994
Porphyromonas bennonis	0.000261162	0.001285876	0.000320587	0.000201737	1	1	-0.269768384
Porphyromonas endodontalis	1.421205589	3.457557484	0.764097982	2.078313195	0.589189115	0.791629736	-0.096717986
Porphyromonas gingivalis	6.37980143	11.76777604	3.642840251	9.116762609	0.166979315	0.562537401	-0.349991562
Porphyromonas pasteri	0.11528604	0.357856417	0.166172738	0.064399342	0.259406241	0.600118256	0.163734781
Porphyromonas sp. HMT-275	0.000325826	0.002233753	0.000651653	0	0.3378947	0.600118256	0.205319093
Porphyromonas sp. HMT-277	0.003507295	0.024044804	0.00701459	0	0.3378947	0.600118256	0.226377461
Porphyromonas sp. HMT-278	0.037175828	0.251341326	0.001013294	0.073338362	1	1	-0.054862832
Porphyromonas sp. HMT-285	0.000473485	0.003246049	0	0.00094697	0.3378947	0.600118256	-0.236906645
Porphyromonas sp. HMT-930	0.000537585	0.00292694	7.63545E-05	0.000998815	0.53905226	0.77618636	-0.119261025
Porphyromonas uenonis	0.012018986	0.045475869	0.000295062	0.023742911	0.072772322	0.443836303	-0.27402921
Prevotella baroniae	0.047888532	0.150267223	0.06880647	0.026970593	0.087123939	0.461030845	0.216634125
Prevotella bivia	0.000705974	0.004839912	0	0.001411948	0.3378947	0.600118256	-0.236906645
Prevotella buccae	0.074081368	0.13916372	0.063972568	0.084190167	0.110755735	0.530251708	-0.326429738
Prevotella buccalis	0.000147844	0.00071072	0.00013786	0.000157828	1	1	-0.279643694
Prevotella colorans	0.001513854	0.007316585	0	0.003027709	0.161691678	0.561061286	-0.254093289
Prevotella dentalis	0.050593901	0.119752338	0.023908288	0.077279514	0.375651897	0.639514623	-0.23976141
Prevotella denticola	0.862073567	1.933107154	0.678121	1.046026135	0.83675999	0.967837147	0.017755062
Prevotella disiens	0.003325342	0.017206609	0	0.006650683	0.081078758	0.443836303	-0.313803954
Prevotella enoeca	0.004120312	0.017355677	0.001209696	0.007030928	0.59959757	0.791629736	0.01248829
Prevotella fusca	0.038259396	0.19064297	0.027696548	0.048822244	1	1	0.109725664
Prevotella histicola	0.265762643	1.357077327	0.475009466	0.056515821	0.031632396	0.325353415	0.22424416
Prevotella intermedia	0.54443413	1.577071949	0.079110737	1.009757522	0.34259973	0.600118256	-0.030905771
Prevotella jejuni	0.061873085	0.153738852	0.047992838	0.075753333	0.145745462	0.561061286	0.307849809
Prevotella loescheii	0.05426603	0.32870498	0.096687244	0.011844817	0.68304078	0.840964017	-0.007165413
Prevotella maculosa	0.040013753	0.062116019	0.031188956	0.04883855	0.260985404	0.600118256	-0.180432025
Prevotella marshii	0.03814976	0.156872309	0.006330166	0.069969353	0.139463325	0.561061286	-0.167019642
Prevotella melaninogenica	0.375666748	0.890274389	0.460590869	0.290742627	0.011011433	0.194229447	0.40534703
Prevotella micans	0.006110532	0.016087974	0.003398355	0.008822709	0.415952213	0.674183698	-0.210558951
Prevotella multiformis	0.024262064	0.088144559	0.033203118	0.015321009	0.699312979	0.852329638	0.054814426
Prevotella nanceiensis	0.014242532	0.036259049	0.025755987	0.002729078	0.095086626	0.482072601	0.27545186

Species_OTUs	Mean abundance	Std_deviation	Mean abundance - health	Mean abundance - peri-implantitis	P value	FDR corrected P value	LDA correlation (CAP)
Prevotella nigrescens	2.733999589	4.593836392	1.242967998	4.225031181	0.112253713	0.531948567	-0.252239545
Prevotella oralis	0.257524308	0.529179615	0.17277468	0.342273937	0.974942147	1	0.026347935
Prevotella oris	1.334962995	2.242535042	0.827717438	1.842208552	0.183511271	0.57687949	-0.380734804
Prevotella oulorum	0.173491451	0.546554076	0.037081455	0.309901447	0.905048715	1	0.037337011
Prevotella pallens	0.106347766	0.242987288	0.152724651	0.05997088	0.056753572	0.383388493	0.371452586
Prevotella pleuritidis	0.611903803	2.468947608	0.324648114	0.899159491	0.342043916	0.600118256	0.183183936
Prevotella saccharolytica	0.012355544	0.033511792	0.007336686	0.017374401	0.585000249	0.791629736	-0.11883211
Prevotella salivae	0.354493981	0.837570515	0.190875944	0.518112017	0.725389404	0.875134498	0.109162849
Prevotella scopos	0.000425277	0.002130158	0.000692726	0.000157828	0.572000957	0.787351719	-0.015780743
Prevotella shahii	0.000163304	0.000784094	0.000171031	0.000155577	1	1	-0.041852503
Prevotella sp. HMT-292	0.00015053	0.001031981	0	0.00030106	0.3378947	0.600118256	-0.110556435
Prevotella sp. HMT-293	7.89141E-05	0.000541008	0	0.000157828	0.3378947	0.600118256	-0.236906645
Prevotella sp. HMT-300	0.109854121	0.352499461	0.100984688	0.118723555	0.332137948	0.600118256	-0.105536902
Prevotella sp. HMT-301	0.003160178	0.012406756	0.002416839	0.003903518	0.440392808	0.693919188	0.106456711
Prevotella sp. HMT-304	0.053714929	0.2814699	0.081825725	0.025604133	1	1	0.123752156
Prevotella sp. HMT-305	0.037410768	0.143348502	0.017479399	0.057342137	0.70506773	0.856438277	-0.103295742
Prevotella sp. HMT-306	0.00882265	0.027505997	0.008716009	0.008929292	0.797338491	0.932430832	-0.020819429
Prevotella sp. HMT-309	0.000720359	0.003336093	0.000174203	0.001266514	0.301928049	0.600118256	-0.129979391
Prevotella sp. HMT-313	0.044384735	0.174251943	0.010145037	0.078624432	0.824430743	0.958815974	-0.031473156
Prevotella sp. HMT-314	0.07081336	0.248380459	0.009079541	0.132547178	0.125516655	0.561061286	-0.33760808
Prevotella sp. HMT-315	0.006562321	0.020196479	0.00699784	0.006126803	0.749670045	0.887507422	0.04367472
Prevotella sp. HMT-317	0.077242087	0.213575947	0.081478341	0.073005833	0.499128103	0.75105769	-0.086019574
Prevotella sp. HMT-376	0.067349637	0.167457409	0.018451593	0.11624768	0.615610318	0.799412172	-0.188224228
Prevotella sp. HMT-443	0.011967142	0.055509238	0.015044964	0.00888932	0.572000957	0.787351719	0.110723901
Prevotella sp. HMT-472	0.076588385	0.174994788	0.071581874	0.081594896	0.226494723	0.600118256	-0.179529967
Prevotella sp. HMT-475	0.019050466	0.05565384	0.012335963	0.025764969	0.644023334	0.820569263	-0.19490825
Prevotella sp. HMT-515	0.001406232	0.009640644	0	0.002812465	0.3378947	0.600118256	-0.047381329
Prevotella sp. HMT-526	0.051701976	0.162396639	0.084476042	0.018927911	0.408886257	0.665750701	0.274245252
Prevotella timonensis	0.008052488	0.037052229	0.00070099	0.015403986	0.9485568	1	-0.20934834
Prevotella veroralis	0.033746009	0.12706082	0.048298834	0.019193184	0.333959585	0.600118256	0.069294158
Pseudopropionibacterium sp. HMT-194	0.027281718	0.162444081	0.051907702	0.002655733	0.650245177	0.82252129	-0.05339986
Pseudoramibacter alactolyticus	0.310821118	0.757128686	0.03095776	0.590684476	0.006485322	0.192529246	-0.367591372
Pyramidobacter piscolens	0.005153572	0.030404133	0.000723096	0.009584048	0.9485568	1	-0.102692777
Rothia aeria	0.342077153	1.464578703	0.621180032	0.062974274	0.034943696	0.346706982	0.308124819
Rothia dentocariosa	2.07376741	4.512485931	3.641930428	0.505604393	4.22558E-05	0.013416213	0.622123317
Rothia mucilaginosa	0.971852481	1.719082872	1.609128199	0.334576762	0.00134265	0.121797566	0.519270441
Ruminococcaceae G-1 sp. HMT-075	0.017109722	0.04696555	0.022562196	0.011657248	0.969974386	1	0.01492816
Ruminococcaceae G-2 sp. HMT-085	0.0054554	0.017181429	0.008693896	0.002216905	0.163258663	0.562537401	0.13601695
Saccharibacteria G-1 sp. HMT-346	0.15901645	0.270946127	0.174203416	0.143829484	0.506435097	0.756673616	0.066846629
Saccharibacteria G-1 sp. HMT-347	0.001610265	0.008624317	0.003220529	0	0.081078758	0.443836303	0.073471
Saccharibacteria G-1 sp. HMT-348	0.004029579	0.022405071	0.00164731	0.006411847	0.344004795	0.600118256	0.310682447
Saccharibacteria G-1 sp. HMT-349	0.687328899	2.499222545	0.106311687	1.268346111	0.086824764	0.461030845	-0.281223781
Saccharibacteria G-1 sp. HMT-352	0.036556346	0.072002668	0.047836714	0.025275978	0.052244963	0.368944353	0.372285393
Saccharibacteria G-1 sp. HMT-488	0.149964852	0.350787394	0.161522565	0.13840714	0.043040454	0.354302708	0.202741921
Saccharibacteria G-1 sp. HMT-869	0.000669201	0.004587809	0.001338401	0	0.3378947	0.600118256	0.005264592
Saccharibacteria G-1 sp. HMT-952	0.040453734	0.076529694	0.036729755	0.044177713	0.748858202	0.887507422	-0.0921995
Saccharibacteria G-2 sp. HMT-350	0.001462661	0.005501392	0.001351059	0.001574263	0.344004795	0.600118256	0.032041958
Saccharibacteria G-3 sp. HMT-351	0.009664344	0.027261008	0.012205215	0.007123473	0.247857501	0.600118256	0.140260763
Saccharibacteria G-5 sp. HMT-356	0.220729784	0.597098749	0.252396928	0.189062641	0.081923866	0.444629527	0.281936495
Saccharibacteria G-6 sp. HMT-870	0.009916753	0.038606956	0.00648884	0.013344665	0.504748588	0.755932436	0.044732076
Saccharibacteria G-8 sp. HMT-955	0.010529433	0.049896436	0.00791387	0.013144996	0.399060512	0.658991323	0.227554762
Scardovia inopinata	0.000115799	0.000793875	0	0.000231597	0.3378947	0.600118256	-0.24743583
Scardovia wiggsiae	0.681833204	2.464705584	0.366544361	0.997122047	0.342834976	0.600118256	0.285525058
Selenomonas artemidis	0.137360629	0.329685397	0.143517822	0.131203436	0.786310493	0.922933758	-0.085521165
Selenomonas infelix	0.109699005	0.256976692	0.072383617	0.147014394	0.48088973	0.739382515	0.066510119
Selenomonas noxia	0.112474166	0.402792039	0.127892096	0.097056237	0.137965362	0.561061286	0.320382092
Selenomonas sp. HMT-126	0.013664571	0.072220591	0.003507518	0.023821625	0.740990061	0.885532006	-0.021086785

Species_OTUs	Mean abundance	Std_deviation	Mean abundance - health	Mean abundance - peri-implantitis	P value	FDR corrected P value	LDA correlation (CAP)
Selenomonas sp. HMT-133	0.001304099	0.007560497	0.000436977	0.002171221	1	1	0.033544703
Selenomonas sp. HMT-134	0.045366179	0.108063159	0.053921616	0.036810742	0.49014751	0.742824985	0.142147791
Selenomonas sp. HMT-136	0.061836397	0.30086839	0.006958859	0.116713935	0.290585133	0.600118256	-0.165274903
Selenomonas sp. HMT-137	0.008717707	0.043393656	0.002044347	0.015391066	0.886340355	1	-0.069731872
Selenomonas sp. HMT-138	0.042458524	0.215411106	0.01052628	0.074390768	0.620974155	0.804207575	-0.191746714
Selenomonas sp. HMT-149	0.037648312	0.137740462	0.014093687	0.061202936	0.084388692	0.454125587	0.007629028
Selenomonas sp. HMT-388	0.005703156	0.030484856	0.002742003	0.00866431	0.605877247	0.791629736	0.012417634
Selenomonas sp. HMT-442	0.000155577	0.001066583	0	0.000311154	0.3378947	0.600118256	-0.142143987
Selenomonas sp. HMT-478	0.004136396	0.025941774	0.000210345	0.008062446	0.572844873	0.787351719	-0.058961109
Selenomonas sp. HMT-501	0.002981427	0.010806764	0.001177984	0.004784869	0.886340355	1	-0.010200586
Selenomonas sp. HMT-892	0.037736263	0.174533688	0.011598649	0.063873878	0.855557727	0.978881364	0.039070111
Selenomonas sp. HMT-919	0.011879226	0.037766091	0.008776223	0.014982229	0.391528263	0.654264335	-0.150237946
Selenomonas sp. HMT-920	0.026924114	0.155366048	0.006068544	0.047779683	0.750537773	0.887507422	0.058024151
Selenomonas sp. HMT-936	0.000684609	0.00333856	0	0.001369219	0.161691678	0.561061286	-0.134335564
Selenomonas sp. HMT-937	0.003781655	0.01327122	0.000462782	0.007100528	0.07868443	0.443836303	-0.104378586
Selenomonas sputigena	0.434528292	0.995314378	0.207909648	0.661146937	0.683026399	0.840964017	-0.11002192
Shuttleworthia satelles	0.036974578	0.126952469	0.019516428	0.054432727	0.768047822	0.903167346	-0.100795
Simonsiella muelleri	0.001498889	0.007628778	0.000977479	0.0020203	1	1	0.101417864
Slackia exigua	0.019597938	0.029104945	0.019319756	0.01987612	0.445682001	0.700515026	-0.267050225
Solobacterium moorei	0.202033308	0.347785968	0.178398738	0.225667879	0.718069595	0.870179757	-0.151406561
Stomatobaculum longum	0.108676147	0.564320476	0.025287233	0.192065061	0.526013397	0.77618636	0.061846314
Stomatobaculum sp. HMT-097	0.0387221	0.096162923	0.069642844	0.007801355	0.007368056	0.192529246	0.394017089
Stomatobaculum sp. HMT-373	0.005362137	0.019603889	0.007297229	0.003427045	0.41942732	0.674183698	0.063004992
Stomatobaculum sp. HMT-910	0.000233366	0.001599875	0	0.000466731	0.3378947	0.600118256	-0.142143987
Streptococcus anginosus	0.449466097	1.110547319	0.327091177	0.571841018	0.990928139	1	0.07757743
Streptococcus australis	0.015795252	0.042030488	0.025826746	0.005763759	0.026260383	0.30089224	0.339473452
Streptococcus constellatus	0.007196538	0.019745064	0.003552249	0.010840826	0.88144709	1	-0.000428255
Streptococcus constellatus subsp. constellatus	0.018277206	0.052191404	0.007669019	0.028885393	0.971138239	1	0.053206538
Streptococcus cristatus	0.120139571	0.342292663	0.130028507	0.110250635	0.447876403	0.702189783	0.212566748
Streptococcus gordonii	1.327958929	3.100628616	2.086425965	0.569491893	0.026304733	0.30089224	0.493527564
Streptococcus intermedius	1.146996547	2.225267306	1.28119756	1.012795533	0.04762171	0.360813321	0.261808479
Streptococcus massiliensis	0.000413579	0.002835352	0.000827157	0	0.3378947	0.600118256	-0.152673171
Streptococcus mitis oralis group	5.3927709	6.293948549	7.61414398	3.171397819	0.00419749	0.192529246	0.490664351
Streptococcus mutans	0.540142897	2.008603554	1.042278064	0.038007731	0.150054517	0.561061286	0.330380413
Streptococcus parasanguinis	0.895792199	1.8348268	1.537443639	0.254140759	0.003733807	0.192529246	0.559693839
Streptococcus peroris	0.000429802	0.001799269	0.000859603	0	0.041041673	0.35218192	0.310682447
Streptococcus rubneri	0.040566288	0.12379217	0.069454991	0.011677584	0.047888718	0.360813321	0.198540322
Streptococcus salivarius	0.17458322	0.668533473	0.325441213	0.023725228	0.013995666	0.211601141	0.412146213
Streptococcus salivarius subsp. salivarius	0.911375533	2.858116986	1.713684135	0.10906693	0.008687822	0.192529246	0.475382606
Streptococcus salivarius subsp. thermophilus	0.008117937	0.029337305	0.003014108	0.013221765	0.652559841	0.82327101	0.151078973
Streptococcus sanguinis	3.25421731	5.908647019	4.694839331	1.81359529	0.010138861	0.192529246	0.5146777
Streptococcus sinensis	0.000127726	0.000875643	0.000255451	0	0.3378947	0.600118256	0.163202356
Streptococcus sp. HMT-064	0.194567618	0.466911103	0.274408217	0.114727019	0.383231214	0.64893819	0.205245303
Streptococcus sp. HMT-066	0.128840694	0.280667886	0.236971921	0.020709467	0.00650944	0.192529246	0.548250472
Streptococcus vestibularis	0.055435721	0.31258181	0.106348096	0.004523347	0.181770275	0.57687949	0.268421923
Tannerella forsythia	0.877114789	1.24853821	0.476768609	1.27746097	0.109497189	0.530251708	-0.30734978
Tannerella sp. HMT-286	0.120064494	0.327668996	0.124657043	0.115471944	0.624901275	0.804207575	0.078654895
Tannerella sp. HMT-808	0.043083437	0.134481533	0.018235145	0.067931728	0.111060594	0.530251708	-0.227357201
Tannerella sp. HMT-916	0.00527527	0.022355245	0.00574233	0.00480821	0.192668169	0.5968014	-0.271057319
Treponema amylovorum	0.0105082	0.05283602	0.0210164	0	0.081078758	0.443836303	0.223258707
Treponema denticola	0.402057781	1.010448154	0.156139632	0.64797593	0.912245667	1	-0.098806676
Treponema lecithinolyticum	0.049100223	0.194963673	0.088306952	0.009893494	0.091680174	0.477187792	0.27412367
Treponema maltophilum	0.103422573	0.199907832	0.117515654	0.089329492	0.32667304	0.600118256	0.022685502
Treponema medium	0.02523973	0.130243798	0.047312752	0.003166708	0.251744487	0.600118256	0.131912822
Treponema parvum	0.021982488	0.049461353	0.009053249	0.034911727	0.882491322	1	-0.150006976
Treponema pectinovorum	0.000952482	0.003643061	0.001070752	0.000834212	1	1	0.050270775
Treponema socranskii	0.326564003	0.491539296	0.162393395	0.49073461	0.199078364	0.600118256	-0.320097015

Species_OTUs	Mean abundance	Std_deviation	Mean abundance - health	Mean abundance - peri-implantitis	P value	FDR corrected P value	LDA correlation (CAP)
Treponema socranskii subsp. buccale	9.05327E-05	0.000434182	0.000181065	0	0.161691678	0.561061286	0.095618079
Treponema socranskii subsp. paredis	0.004265688	0.018957557	0.006196998	0.002334377	0.68304078	0.840964017	-0.099701597
Treponema socranskii subsp. socranskii	0.062175802	0.139772646	0.036330012	0.088021591	0.511209999	0.762014904	-0.295851953
Treponema sp. HMT-231	0.123022557	0.329437399	0.017604751	0.228440364	0.420074608	0.674183698	-0.129716019
Treponema sp. HMT-234	0.000863571	0.003619782	0.001323081	0.00040406	0.53905226	0.77618636	0.068555687
Treponema sp. HMT-236	0.008376975	0.051487634	0.00180373	0.01495022	1	1	0.013010329
Treponema sp. HMT-237	0.269928648	1.063030427	0.098653422	0.441203874	0.349539271	0.606568013	0.086864489
Treponema sp. HMT-258	0.000396416	0.001901511	0.000388771	0.00040406	1	1	0.013010329
Treponema sp. HMT-260	0.000369851	0.002228484	9.44737E-05	0.000645228	1	1	0.050787308
Treponema sp. HMT-262	0.003507295	0.024044804	0.00701459	0	0.3378947	0.600118256	0.226377461
Treponema sp. HMT-270	0.031087927	0.134249836	0.016812453	0.045363402	0.942784758	1	-0.008690344
Treponema sp. HMT-517	0.000598446	0.003048323	0.000388771	0.00080812	1	1	0.013010329
Treponema sp. HMT-951	0.009544952	0.042937062	0.008584345	0.01050556	0.344004795	0.600118256	0.2267846
Veillonella atypica	0.318319824	0.833459387	0.317630225	0.319009422	0.012273536	0.202666974	0.330188976
Veillonella dispar	0.762377351	1.203320169	0.944347182	0.580407521	0.003517504	0.192529246	0.449915864
Veillonella parvula	0.215775721	0.360835133	0.278642665	0.152908777	0.521628783	0.773911862	0.096289968
Veillonella rogosae	0.049769473	0.086130022	0.078169898	0.021369048	0.027583231	0.307286872	0.432165221
Veillonella sp. HMT-780	0.001849173	0.011962988	0.0034881	0.000210247	1	1	-0.159258964
Veillonella sp. HMT-917	0.017822339	0.067419799	0.00867884	0.026965838	0.660627721	0.827132464	-0.049734987
Veillonella tobetsuensis	0.000664709	0.003404948	0.000908925	0.000420493	1	1	0.025080152
Veillonellaceae G-1 sp. HMT-132	0.171691666	0.359111555	0.03012831	0.313255023	0.000666974	0.105882096	-0.559810931
Veillonellaceae G-1 sp. HMT-135	0.008040284	0.047694404	0.015172061	0.000908507	0.360154597	0.61810316	0.15359238
Veillonellaceae G-1 sp. HMT-145	0.009441409	0.029001559	0.002769218	0.0161136	0.049434266	0.360813321	-0.36619573
Veillonellaceae G-1 sp. HMT-148	0.000854794	0.004220203	0.001551759	0.000157828	0.301928049	0.600118256	0.062498801
Veillonellaceae G-1 sp. HMT-150	0.090458362	0.257583713	0.035700468	0.145216256	0.017307244	0.244224442	-0.239040213
Veillonellaceae G-1 sp. HMT-483	0.002368163	0.011138053	0	0.004736326	0.041041673	0.35218192	-0.302190762
Veillonellaceae G-1 sp. HMT-918	0.001861306	0.011104357	0.003722611	0	0.161691678	0.561061286	0.212711038

D. Full-16S based genus level composition

Genus	Mean abundance	Std_deviation	Mean abundance - health	Mean abundance - peri-implantitis	P value	FDR corrected P value
Abiotrophia	0.7278257	1.730280658	0.834844252	0.620807148	0.289502698	0.559062141
Actinomyces	1.665945047	2.601436149	2.533323845	0.798566249	0.00591703	0.098360715
Aggregatibacter	1.117239896	3.742043	1.912230806	0.322248986	0.019075972	0.160893684
Alloprevotella	1.764823616	3.007255859	0.961529144	2.568118088	0.649782871	0.882540914
Alloscardovia	0.020267517	0.080036685	0.033680311	0.006854724	0.911934721	0.999832043
Anaerocella	0.00101015	0.006925239	0	0.0020203	0.3378947	0.559062141
Anaeroglobus	0.657067711	1.761425482	0.242234865	1.071900558	0.124140254	0.387306847
Arachnia	0.283988814	0.777680104	0.272125577	0.295852051	0.279222076	0.559062141
Bacteroidales	0.261496915	0.477729118	0.247375837	0.275617993	0.558807139	0.789035799
Bergeyella	0.093481604	0.122696307	0.133101457	0.053861752	0.101211646	0.354240762
Bifidobacterium	1.131423197	2.152850501	0.944148699	1.318697695	0.908805571	0.999832043
Brachymonas	0.018382409	0.039912379	0.030673937	0.006090881	0.056260228	0.269456882
Bulleidia	0.037223181	0.09847325	0.031188611	0.043257752	0.892472746	0.999832043
Campylobacter	1.251827468	1.086292898	1.053894994	1.449759943	0.281836841	0.559062141
Capnocytophaga	1.672904416	2.311645496	1.559652495	1.786156338	0.718208808	0.920521149
Cardiobacterium	0.510287154	1.200566471	0.414479896	0.606094413	0.127683576	0.387306847
Catonella	0.253474454	0.476788638	0.153325031	0.353623878	0.336073882	0.559062141
Caviibacter	0.069513195	0.476558456	0.13902639	0	0.3378947	0.559062141
Centipeda	0.031137953	0.074290858	0.037941091	0.024334815	0.87935097	0.999832043
Corynebacterium	0.169162958	0.333941334	0.230994059	0.107331857	0.004130665	0.093972637
Cryptobacterium	0.113812384	0.469686835	0.0250247	0.202600069	0.268642742	0.559062141
Desulfobulbus	0.080109069	0.217448888	0.026209812	0.134008326	0.100769381	0.354240762
Desulfovibrio	0.09343958	0.377998674	0.005134769	0.181744392	0.316306015	0.559062141
Dialister	1.483391714	1.460451524	0.74204384	2.224739587	0.000668127	0.020266515
Eggerthia	0.037588829	0.094476352	0.060104565	0.015073093	0.165538514	0.407135264
Eikenella	0.227981106	0.262155159	0.211104465	0.244857747	1	1
Eubacterium	0.799835121	1.39024314	0.713366132	0.88630411	0.109844795	0.370217641
Filifactor	0.167601748	0.430504877	0.273074267	0.062129229	0.019448687	0.160893684
Flexilinea	0.046656691	0.138162071	0.010465458	0.082847923	0.033603109	0.206699361
Fretibacterium	0.732029153	1.363790855	0.495771039	0.968287267	0.39002431	0.62267039
Fudania	0.008657348	0.030934207	0.009282517	0.008032179	0.750537773	0.922958613
Fusobacterium	9.544573301	7.16093581	7.679862338	11.40928426	0.085151945	0.352219409
Gardnerella	0.001299957	0.008912053	0	0.002599913	0.3378947	0.559062141
Gemella	1.57931654	2.570974214	1.975999185	1.182633895	0.06200204	0.282109284
Granulicatella	0.989616688	1.299753963	1.218660469	0.760572907	0.040149021	0.21491535
Haemophilus	1.30267094	2.959335477	1.928262496	0.677079385	0.00805125	0.104666245
Howardella	0.009834402	0.018371071	0.008191662	0.011477143	0.953444711	1
Johnsonella	0.033407875	0.1370297	0.064003285	0.002812465	0.155785777	0.407135264
Kingella	0.372251491	0.600552343	0.323795506	0.420707475	0.296636074	0.559062141
Lachnoanaerobaculum	0.110679345	0.251160774	0.165791673	0.055567018	0.034071323	0.206699361
Lacticaseibacillus	0.399610615	1.60605984	0.296541454	0.502679776	0.70324001	0.920521149
Lactobacillus	1.154744421	5.182759091	0.395019488	1.914469353	0.708502746	0.920521149
Lancefieldella	1.450918629	2.391424382	0.614860131	2.286977127	0.17007846	0.407293154
Lautropia	0.482858991	1.148859661	0.489856605	0.475861376	0.14421664	0.407135264
Leptotrichia	1.752324455	2.5441323	1.749114806	1.755534103	0.288223248	0.559062141
Ligilactobacillus	0.071642577	0.413616357	0.137032028	0.006253127	0.301928049	0.559062141
Limosilactobacillus	0.471632722	2.048031742	0.438199996	0.505065448	0.935757696	1
Megasphaera	0.406635182	0.891487889	0.164317324	0.648953039	0.610647957	0.841954001
Mesobacillus	4.72368E-05	0.000323839	9.44737E-05	0	0.3378947	0.559062141
Metamycoplasma	0.095921339	0.186966795	0.034223472	0.157619205	0.047942235	0.242374635
Mogibacterium	0.636869341	1.091667577	0.490343832	0.78339485	0.100973392	0.354240762
MollicutesG-2	9.77173E-05	0.000669916	0.000195435	0	0.3378947	0.559062141
Neisseria	3.475793114	5.785480114	4.524292335	2.427293893	0.036353482	0.206760428
Odoribacter	0.008602247	0.055784707	0.016267005	0.000937488	1	1
Olsenella	0.180614544	0.337440474	0.134289314	0.226939774	0.123270213	0.387306847
Oribacterium	0.30931697	0.509346187	0.275345314	0.343288625	0.78070875	0.934796003

Genus	Mean abundance	Std_deviation	Mean abundance - health	Mean abundance - peri-implantitis	P value	FDR corrected P value
Ottowia	0.183352336	0.62048793	0.130492156	0.236212515	0.74741	0.922958613
Parascardovia	0.000205514	0.001015519	0.000255451	0.000155577	1	1
Parvimonas	1.281963503	1.599939922	0.893445795	1.670481211	0.031936275	0.206699361
Peptidiphaga	0.134958101	0.514348364	0.196257416	0.073658786	0.165351457	0.407135264
Peptoanaerobacter	0.007871525	0.052620335	0.000388771	0.01535428	1	1
Peptococcus	0.148816493	0.305132086	0.094407942	0.203225044	0.800063272	0.945529321
PeptoniphilaceaeG-1	0.193177137	0.866912996	0.143130148	0.243224127	0.551551039	0.789035799
Peptoniphilus	0.000387385	0.001377659	0.000573033	0.000201737	0.322518942	0.559062141
Peptostreptococcaceae_incertae_sedis	0.067735142	0.207745503	0.04724982	0.088220463	0.811848786	0.947156917
Peptostreptococcus	0.454414246	0.971523835	0.178725491	0.730103001	0.906790003	0.999832043
Phocaeicola	0.021552183	0.054789076	0.028953453	0.014150913	0.744706801	0.922958613
Porphyromonas	8.060157616	12.08143094	4.627276501	11.49303873	0.067726454	0.293481299
Prevotella	9.203466578	7.710821119	5.884472739	12.52246042	0.009969192	0.113399555
Propionivibrio	0.006416144	0.01583414	0.009886999	0.002945288	0.154666319	0.407135264
Pseudoramibacter	0.311872245	0.760645857	0.03095776	0.59278673	0.006485322	0.098360715
Pyramidobacter	0.005153572	0.030404133	0.000723096	0.009584048	0.9485568	1
Rothia	5.251785473	8.060365815	8.754217863	1.749353082	4.22558E-05	0.001922638
RuminococcaceaeG-2	0.0054554	0.017181429	0.008693896	0.002216905	0.163258663	0.407135264
Saccharibacteria	1.352836668	2.789434237	0.819202464	1.886470872	0.563597	0.789035799
Scardovia	0.681949003	2.464673677	0.366544361	0.997353644	0.434030791	0.675951034
Schaalia	0.766261801	0.873992141	0.949110793	0.58341281	0.022979765	0.174263216
Schwartzia	0.332961735	0.597233646	0.129635775	0.536287695	0.016054051	0.160893684
Selenomonas	1.433096118	2.089378127	0.799153123	2.067039113	0.439332478	0.675951034
Shuttleworthia	0.036974578	0.126952469	0.019516428	0.054432727	0.768047822	0.931898024
Simonsiella	0.001498889	0.007628778	0.000977479	0.0020203	1	1
Slackia	0.019597938	0.029104945	0.019319756	0.01987612	0.445682001	0.675951034
Solobacterium	0.202033308	0.347785968	0.178398738	0.225667879	0.718069595	0.920521149
Sphaerochaeta	0.011107477	0.05903799	0.002984492	0.019230463	0.388162871	0.62267039
SR1	0.017953094	0.054765933	0.003850115	0.032056073	0.485044291	0.723590664
Stomatobaculum	0.147631612	0.569829908	0.094930078	0.200333147	0.306325227	0.559062141
Streptococcus	21.56184874	14.41318133	30.44311086	12.68058661	4.17358E-07	3.79796E-05
Tannerella	1.051161257	1.327098207	0.625764054	1.476558461	0.137539044	0.403743646
Treponema	1.570966114	2.186120355	0.911758077	2.230174151	0.563332009	0.789035799
Unknown	0.868380612	1.12672548	0.5942384	1.142522823	0.278964122	0.559062141
Veillonella	4.234133727	4.790473315	5.549484186	2.918783268	0.093046618	0.354240762

E. Full-16S based class level composition

Class	Mean abundance	Std_deviation	Mean abundance - health	Mean abundance - peri-implantitis	P value	FDR corrected P value
Acidimicrobiia	0.000842432	0.00577542	0.001684863	0	0.3378947	0.473052581
Actinobacteria	10.11590473	9.276634219	14.28994089	5.941868567	0.000718634	0.00503044
Anaerolineae	0.046656691	0.138162071	0.010465458	0.082847923	0.033603109	0.093208874
Bacilli	26.9592119	17.23445756	35.74373351	18.17469029	7.10844E-05	0.000746386
Bacteroidia	20.6458078	14.17710477	12.67745442	28.61416117	5.78771E-05	0.000746386
Betaproteobacteria	4.770194336	6.458775142	5.723658624	3.816730048	0.035508142	0.093208874
Campylobacteria	1.251827468	1.086292898	1.053894994	1.449759943	0.281836841	0.455274896
Clostridia	5.298982594	5.05502243	3.899795462	6.698169726	0.009969192	0.034892171
Coriobacteriia	1.7650488	2.795015322	0.793704509	2.73639309	0.129624174	0.247464331
Deltaproteobacteria	0.173548649	0.533984769	0.031344581	0.315752717	0.008963333	0.034892171
Erysipelotrichia	0.28438807	0.387767699	0.282871453	0.285904687	0.439332478	0.542704826
Flavobacteriia	1.766386021	2.374520646	1.692753952	1.840018089	0.613421744	0.613421744
Fusobacteriia	11.36641095	7.95764148	9.568003534	13.16481837	0.093046618	0.195397898
Gammaproteobacteria	2.930197991	5.01213226	4.254973198	1.605422783	0.00757305	0.034892171
Mollicutes	0.096019056	0.18697969	0.034418907	0.157619205	0.050462202	0.117745138
Negativicutes	8.838260228	6.528891902	7.706479942	9.970040514	0.185272618	0.324227082
Saccharibacteria	1.352836668	2.789434237	0.819202464	1.886470872	0.563597	0.59177685
Sphingobacteriia	0.000266207	0.001825021	0.000532413	0	0.3378947	0.473052581
Spirochaetia	1.582073592	2.216086896	0.914742569	2.249404614	0.563332009	0.59177685
SR1	0.017953094	0.054765933	0.003850115	0.032056073	0.485044291	0.565885006
Synergistia	0.737182725	1.364121985	0.496494135	0.977871315	0.39002431	0.511906907

F. Metatranscriptome-based features (Enzyme Commission numbers - EC)

EC	Mean abundance	Std_deviation	Mean abundance - health	Mean abundance - peri-implantitis	P value	FDR corrected P value	LDA correlation (CAP)
1.1.1.1	0.022648022	0.015407621	0.028439996	0.016856048	0.013583315	0.052578624	0.631893183
2.1.1.33	0.018610632	0.005895405	0.02110454	0.016116723	0.003157811	0.018990575	0.458423795
4.1.1.19	0.038208695	0.030754866	0.035683839	0.040733551	0.055961569	0.144949638	-0.659465914
2.7.13.3	0.220649176	0.090036281	0.255263277	0.186035076	0.000129181	0.001781874	0.646330873
3.1.3.97	0.003212616	0.002021798	0.002847736	0.003577495	0.097204238	0.210649583	-0.419887104
6.1.1.7	0.1058436	0.017839672	0.112726066	0.098961134	0.008230024	0.036374662	0.425966131
6.3.1.2	0.067738407	0.024816867	0.068342459	0.067134355	0.846132513	0.915107845	0.081958315
4.6.1.12	0.003815841	0.002627691	0.002583294	0.005048389	0.000189424	0.002334886	-0.806556665
2.7.7.7	0.366572661	0.055261491	0.370836125	0.362309197	0.30070683	0.47991595	0.055145462
3.1.3.18	0.013800473	0.007267465	0.015034374	0.012566571	0.238172101	0.399945036	0.23957881
3.6.3.17	0.067900993	0.023579658	0.066651418	0.069150568	0.630998004	0.750119378	-0.040924881
3.1.3.15	0.005012958	0.004024978	0.004896273	0.005129643	0.690279643	0.800337572	0.071320017
6.3.4.3	0.09244931	0.034997392	0.07542637	0.109472249	1.24541E-05	0.000408399	-0.680416848
4.2.99.18	0.015675488	0.003991513	0.015626145	0.01572483	0.959276437	0.987691024	-0.013352149
5.3.1.26	0.015980874	0.022948744	0.023213102	0.008748647	0.000300628	0.003286107	0.670104212
4.1.2.40	0.027528299	0.035683222	0.037499055	0.017557543	0.00187728	0.012845322	0.670104212
3.1.3.48	0.021922316	0.024745627	0.03118936	0.012655271	1.24541E-05	0.000408399	0.775618758
5.4.99.25	0.017677619	0.004917047	0.017344984	0.018010255	0.751487275	0.845559951	-0.16283109
2.1.1.176	0.020130915	0.007673472	0.024837742	0.015424087	6.09052E-06	0.000252032	0.73501954
4.1.1.49	0.176725306	0.092628143	0.131360265	0.222090347	0.000718634	0.006152642	-0.557967868
6.1.1.12	0.080075828	0.017459816	0.085978039	0.074173618	0.008230024	0.036374662	0.262592271
6.3.5.1	0.01811781	0.01417967	0.010922206	0.025313414	4.69671E-05	0.000927601	-0.869735128
3.1.3.16	0.036780163	0.019828929	0.048886515	0.02467381	4.75872E-06	0.00022974	0.764871906
2.7.1.4	0.015349467	0.007650518	0.015039613	0.015659321	0.79844653	0.883885395	0.138840643
2.1.1.14	0.077853827	0.123105406	0.10688812	0.048819535	0.003157811	0.018990575	0.726877985
5.1.1.3	0.016315913	0.006695965	0.013970737	0.018661089	0.02051487	0.072168252	-0.767151541
3.4.11.18	0.083111944	0.028683912	0.075994731	0.090229158	0.037415704	0.110031291	-0.661311333
1.7.2.3	0.002699843	0.002689203	0.002198935	0.003200752	0.214346279	0.37068043	-0.328158923
6.1.1.17	0.04230695	0.015658502	0.035167096	0.049446804	0.000429499	0.004217336	-0.73849327
2.4.1.227	0.031050729	0.006285521	0.031820863	0.030280596	0.384241116	0.530429754	-0.021168042
3.5.1.53	0.00583162	0.002772391	0.004960449	0.006702791	0.004823307	0.025635801	-0.465696917
2.5.1.55	0.015569766	0.006024268	0.012526414	0.018613118	0.000189424	0.002334886	-0.674772036
6.1.1.22	0.060068504	0.014483537	0.058033152	0.062103857	0.105955089	0.221867402	-0.461680417
2.1.1.198	0.031759888	0.01048769	0.028535223	0.034984553	0.011299384	0.045991404	-0.704407295
2.3.1.179	0.131883322	0.042072803	0.122957626	0.140809018	0.330525182	0.489384158	-0.400781589
2.7.2.1	0.133394596	0.06755072	0.14852298	0.118266212	0.06461309	0.158457409	0.275835866
6.3.5.5	0.109906366	0.03301785	0.110054947	0.109757785	0.602179894	0.731368732	-0.062744247
2.7.1.194	0.014258301	0.012760602	0.017077398	0.011439203	0.06461309	0.158457409	0.533000434
3.5.4.5	0.011943798	0.004619457	0.013007754	0.010879841	0.130510373	0.26012274	0.163482414
5.1.3.2	0.073577554	0.015707654	0.069254865	0.077900243	0.06461309	0.158457409	-0.452778984
2.7.1.24	0.009792126	0.003155101	0.010039638	0.009544614	0.255039588	0.418959172	0.163048198
2.4.2.1	0.024463585	0.007728551	0.025006127	0.023921043	0.862160642	0.924389386	0.038536691
5.4.99.23	0.034363934	0.012450717	0.032030497	0.03669737	0.033682558	0.102522391	-0.523773339
3.6.4.12	0.313451102	0.070382803	0.316001398	0.310900806	0.165425496	0.302323356	-0.092705167
1.20.4.1	0.023210508	0.014700114	0.031284743	0.015136274	3.79844E-05	0.00084637	0.695071646
5.4.99.2	0.107536157	0.092993782	0.102821115	0.112251199	0.153095027	0.293361805	-0.250434216
2.7.9.1	0.110624854	0.061876597	0.079367431	0.141882278	0.000781202	0.006496311	-0.74305254
5.3.3.2	0.007414453	0.004788125	0.010536335	0.00429257	8.55438E-07	7.07977E-05	0.814481112
5.2.1.8	0.407620093	0.135483964	0.347818654	0.467421533	0.004500767	0.024521421	-0.705384281
3.5.1.124	0.024376601	0.007603738	0.024694837	0.024058365	0.573959013	0.706974319	-0.108445506
3.4.16.4	0.113806829	0.032387063	0.105201848	0.12241181	0.373099055	0.52125897	-0.318063396
3.4.21.53	0.09217144	0.031681922	0.078896124	0.105446757	0.000718634	0.006152642	-0.609313938
3.5.1.1	0.028514185	0.009825571	0.026453341	0.030575028	0.085151945	0.189979564	-0.386561007
2.4.1.1	0.123732716	0.076905753	0.154447651	0.093017781	0.005166141	0.026722477	0.617564047
2.5.1.17	0.027353046	0.010053279	0.026778143	0.027927949	0.546368678	0.686615157	-0.405775076
3.2.2.9	0.016387312	0.004975062	0.018936227	0.013838397	0.000468756	0.004501094	0.686712983

EC	Mean abundance	Std_deviation	Mean abundance - health	Mean abundance - peri-implantitis	P value	FDR corrected P value	LDA correlation (CAP)
2.7.7.24	0.029405397	0.008854379	0.027915997	0.030894797	0.430768443	0.577681754	-0.269539731
3.2.1.78	0.002924365	0.004261892	0.001717333	0.004131398	0.027147143	0.088025623	-0.302323057
2.3.3.13	0.016023478	0.010898736	0.019424342	0.012622615	0.010616164	0.043930696	0.535497178
3.1.3.5	0.033633081	0.054054728	0.045103136	0.022163027	0.008230024	0.036374662	0.615610074
6.3.4.5	0.015083034	0.011767012	0.015998831	0.014167236	0.246506703	0.409285723	0.285280069
3.4.22.70	0.023681036	0.01530801	0.033505281	0.013856791	9.83425E-07	7.12164E-05	0.735887972
2.2.1.1	0.122124983	0.033717443	0.124372111	0.119877855	0.467672658	0.616703398	0.104646114
5.3.1.8	0.02001191	0.007296104	0.021211012	0.018812808	0.44288188	0.590282751	0.212657403
4.3.3.7	0.025567434	0.010168534	0.025801861	0.025333006	0.587992654	0.721702848	-0.131567521
1.1.1.25	0.014288269	0.005353963	0.016461491	0.012115047	0.00419749	0.023307469	0.310790274
3.2.1.20	0.004885833	0.016081279	0.007006868	0.002764799	0.91055459	0.956219866	-0.064264003
5.4.99.19	0.011524622	0.003710749	0.013549941	0.009499303	6.41681E-05	0.001115242	0.387863656
3.5.1.88	0.022231548	0.003921422	0.022736271	0.021726826	0.206792441	0.359046216	-0.085974815
1.1.1.94	0.034692051	0.006077598	0.035419303	0.033964799	0.330525182	0.489384158	0.159574468
2.10.1.1	0.007110115	0.003709785	0.008124163	0.006096067	0.048279687	0.129691029	0.463525836
3.6.3.21	0.029448312	0.01516883	0.03779806	0.021098564	0.000129181	0.001781874	0.701259227
2.3.1.12	0.081413612	0.126575417	0.120841198	0.041986025	0.000781202	0.006496311	0.842270951
5.3.1.1	0.099463171	0.028080604	0.102318186	0.096608156	0.532819632	0.676479136	0.198979592
2.7.11.1	0.102100911	0.033886785	0.116199233	0.08800259	0.003393537	0.020061115	0.374402953
2.7.1.33	0.015246825	0.004406032	0.015669954	0.014823696	0.407115953	0.554954923	0.145679548
6.3.3.1	0.024055815	0.009391553	0.021631123	0.026480507	0.085151945	0.189979564	-0.369735128
4.3.2.2	0.048464655	0.013965334	0.045239087	0.051690223	0.081408821	0.184237696	-0.521059488
2.1.1.217	0.004422804	0.002636532	0.005505606	0.003340003	0.000329015	0.00344475	0.636018237
4.1.1.20	0.017616296	0.005799356	0.01903974	0.016192852	0.130510373	0.26012274	0.225249674
5.1.3.8	0.004341618	0.003494237	0.002374508	0.006308729	1.4826E-06	9.54357E-05	-0.766608771
1.4.3.16	0.009526249	0.007285588	0.005929653	0.013122844	0.000848669	0.006860405	-0.805471125
3.5.4.12	0.007627424	0.00200993	0.007027083	0.008227765	0.06461309	0.158457409	-0.413808076
2.3.1.54	0.43401077	0.212109341	0.47991911	0.38810243	0.053297186	0.140349256	0.325770734
3.2.1.21	0.010222076	0.010898239	0.006264298	0.014179854	0.002936794	0.017909288	-0.558402084
2.2.1.7	0.035311859	0.013175781	0.029455348	0.041168369	0.002535725	0.016084268	-0.772687799
4.2.1.51	0.005442803	0.003177595	0.007172426	0.003713181	4.69671E-05	0.000927601	0.62755102
1.17.4.1	0.114234527	0.02816313	0.130548924	0.097920129	2.51875E-06	0.000141213	0.76042119
3.7.1.12	0.008671161	0.008423128	0.010551569	0.006790754	0.467672658	0.616703398	0.109639601
3.4.21.107	0.071536561	0.048761268	0.084888581	0.058184541	0.006327364	0.030632198	0.603452019
2.4.99.17	0.036441474	0.020522084	0.031785419	0.041097529	0.000718634	0.006152642	-0.766500217
2.7.7.6	1.176825153	0.237756125	1.247023569	1.106626737	0.024303877	0.081387551	0.487299175
6.3.2.9	0.036564845	0.007305916	0.03794873	0.03518096	0.30070683	0.47991595	0.343682154
2.5.1.31	0.03014631	0.007877243	0.030014659	0.030277961	0.991851953	1	-0.216782458
4.1.1.17	0.013676045	0.024184347	0.015849941	0.011502149	0.159171691	0.299914766	0.26736865
1.6.99.3	0.024121407	0.023675846	0.031384378	0.016858437	0.031936275	0.099293822	0.701367781
2.5.1.15	0.022587389	0.005606523	0.021942643	0.023232134	0.418845832	0.565922893	-0.502713851
2.7.1.21	0.009437077	0.002993639	0.010185308	0.008688845	0.035508142	0.105854462	0.193877551
3.6.4.13	0.08707838	0.026785596	0.100827928	0.073328832	4.69671E-05	0.000927601	0.562527139
2.6.1.21	0.004125746	0.004543617	0.005690146	0.002561345	0.024303877	0.081387551	0.440729483
1.15.1.1	0.111040509	0.060383016	0.121408994	0.100672025	0.085151945	0.189979564	0.509335649
2.8.1.8	0.01096266	0.004961368	0.010453431	0.011471888	0.281836841	0.455448476	-0.196048632
3.6.3.14	0.368343426	0.143689861	0.408129959	0.328556892	0.07095965	0.169174036	0.565458098
4.4.1.8	0.026665724	0.00835456	0.02839044	0.024941008	0.097204238	0.210649583	0.211897525
2.1.1.297	0.020245246	0.006886099	0.021989853	0.018500639	0.009356868	0.04015367	0.395028224
6.1.1.14	0.107335988	0.019639498	0.102791306	0.111880669	0.085151945	0.189979564	-0.581306991
1.1.1.100	0.088454239	0.029352331	0.079133004	0.097775475	0.033682558	0.102522391	-0.520191055
3.6.3.34	0.037636968	0.011412768	0.040743355	0.034530582	0.045920684	0.125487655	0.252388189
3.2.1.23	0.111223647	0.128545595	0.144057013	0.078390281	0.120214723	0.24493926	0.495549284
4.2.1.18	0.000411151	0.000545246	0.000382748	0.000439555	0.500199462	0.646835316	0.176043363
3.6.5.4	0.073950997	0.019071965	0.067459912	0.080442083	0.043657636	0.122382211	-0.426074685
2.7.1.39	0.017750635	0.011214876	0.024046026	0.011455244	2.74461E-05	0.000691325	0.728940512
2.7.1.130	0.013686899	0.00623418	0.010642518	0.01673128	0.000429499	0.004217336	-0.860508033

EC	Mean abundance	Std_deviation	Mean abundance - health	Mean abundance - peri-implantitis	P value	FDR corrected P value	LDA correlation (CAP)
2.7.8.7	0.006370679	0.001797047	0.006757967	0.005983392	0.17185877	0.312437805	0.225358228
2.6.1.16	0.080946149	0.02761083	0.089041567	0.072850731	0.022979765	0.078774815	0.584020842
5.99.1.3	0.250749059	0.034051326	0.251326839	0.25017128	0.751487275	0.845559951	-0.084997829
1.1.1.267	0.014651435	0.006159427	0.012438445	0.016864426	0.06461309	0.158457409	-0.61202779
3.1.3.45	0.010049974	0.003824809	0.008616935	0.011483013	0.006762554	0.031938366	-0.746634824
1.2.1.12	0.544582588	0.257687998	0.58909037	0.500074805	0.031936275	0.099293822	0.41977855
1.1.1.346	0.00258393	0.003092631	0.003190239	0.001977622	0.009356868	0.04015367	0.557859314
4.1.1.23	0.017229548	0.0051083	0.016675174	0.017783922	0.263771717	0.432078458	-0.158597482
2.5.1.49	0.030917251	0.037773459	0.042532686	0.019301817	0.017242287	0.063355381	0.552974381
3.5.2.3	0.026851847	0.008223736	0.025482681	0.028221012	0.34086786	0.490420812	-0.117238385
3.1.26.4	0.030272105	0.006990032	0.032120677	0.028423533	0.033682558	0.102522391	0.405992184
2.1.2.2	0.010729644	0.004554353	0.010342557	0.011116731	0.506231587	0.649800959	-0.162939644
1.8.1.9	0.119970398	0.050642102	0.115960706	0.123980089	0.30070683	0.47991595	-0.028006947
6.1.1.24	0.022653118	0.017228536	0.033871403	0.011434833	4.17358E-07	5.1812E-05	0.813721233
3.2.1.22	0.009509336	0.012860058	0.010660099	0.008358572	0.6603774	0.775267373	-0.041467651
3.2.2.27	0.014241584	0.004362718	0.013849132	0.014634037	0.147193088	0.284246208	-0.185518888
2.3.1.79	0.003732706	0.002604193	0.004287357	0.003178055	0.101506067	0.216463245	0.240881459
5.3.1.9	0.145068491	0.07413634	0.185210734	0.104926248	0.000117169	0.001682975	0.691815024
2.3.1.8	0.059430718	0.022347357	0.049328793	0.069532643	0.001271988	0.009488051	-0.765414677
2.1.1.45	0.01935239	0.003439245	0.019975937	0.018728843	0.291171026	0.468136211	0.242943986
3.2.1.35	0.004568507	0.003611292	0.003748276	0.005388739	0.192257401	0.338887791	-0.260095528
6.5.1.2	0.040139593	0.010763422	0.042183797	0.038095389	0.045920684	0.125487655	0.385909683
6.3.4.13	0.02622723	0.008616464	0.021440598	0.031013862	0.000117169	0.001682975	-0.674446374
1.6.5.2	0.003691209	0.004581871	0.004911124	0.002471295	0.012781229	0.050257413	0.584455059
2.7.8.43	0.002872038	0.00212811	0.002040747	0.00370333	0.003644805	0.020906504	-0.540924881
5.99.1.2	0.102580675	0.028471094	0.100252105	0.104909246	0.630998004	0.750119378	-0.267477204
1.1.1.6	0.01363597	0.015549687	0.015623804	0.011648137	0.418845832	0.565922893	0.270516717
3.1.1.31	0.013434528	0.009022805	0.017901933	0.008967124	1.3982E-05	0.000433941	0.682262267
1.6.5.3	0.078127916	0.043529788	0.061995167	0.094260665	0.012781229	0.050257413	-0.383195832
2.7.2.4	0.04347509	0.011380169	0.048230821	0.038719359	0.004500767	0.024521421	0.549500651
6.3.4.21	0.045530967	0.009210164	0.044121362	0.046940572	0.238172101	0.399945036	-0.426183239
2.1.1.72	0.066524264	0.032699862	0.074920751	0.058127778	0.002354183	0.015324232	0.273664785
3.1.26.11	0.018981255	0.008151803	0.024397648	0.013564861	6.4466E-07	6.7975E-05	0.79385584
6.2.1.22	0.002323509	0.002195671	0.00151842	0.003128599	0.005166141	0.026722477	-0.254993487
1.6.1.2	0.02788263	0.029211688	0.024502663	0.031262596	0.506231587	0.649800959	-0.286691272
2.7.7.27	0.111931243	0.089361314	0.128111682	0.095750803	0.159171691	0.299914766	0.282132002
2.1.1.13	0.016674584	0.013306258	0.018479444	0.014869724	0.089030196	0.195611925	0.333912288
3.1.5.1	0.01399871	0.006315462	0.010083812	0.017913608	3.70198E-06	0.00018383	-0.677702996
2.1.1.104	0.01005167	0.004709986	0.011573604	0.008529736	0.012781229	0.050257413	0.389709075
3.6.1.13	0.018040683	0.008777039	0.023573561	0.012507804	1.43578E-07	2.77265E-05	0.753690838
2.7.2.3	0.148641379	0.05920251	0.164776585	0.132506172	0.017242287	0.063355381	0.441815024
1.16.1.1	0.013165059	0.018410225	0.01849329	0.007836828	0.001174568	0.008953503	0.647742076
4.1.3.27	0.012061612	0.011200221	0.017066245	0.007056978	0.001083935	0.008447885	0.613438993
1.11.1.9	0.029110145	0.017053495	0.02534634	0.03287395	0.050737544	0.134834635	-0.532457664
2.1.1.37	0.017781717	0.012298786	0.018718279	0.016845154	0.862160642	0.924389386	-0.055688233
2.7.1.30	0.065294078	0.076716596	0.081698843	0.048889314	0.222093193	0.379172858	-0.019865393
2.2.1.9	0.008013862	0.004606326	0.008640137	0.007387588	0.830166612	0.906745422	0.11257056
2.9.1.1	0.003311304	0.003698482	0.003844903	0.002777705	0.814269221	0.893434284	0.1951802
1.1.5.3	0.023749458	0.016379018	0.022863476	0.024635439	0.506231587	0.649800959	-0.380807642
2.7.1.202	0.035008879	0.027662318	0.040824859	0.029192898	0.010616164	0.043930696	0.393399913
2.8.4.4	0.017431198	0.007827915	0.013573936	0.021288459	0.000208058	0.002493826	-0.828593139
1.1.1.23	0.003676231	0.003853963	0.003487223	0.003865239	0.506231587	0.649800959	0.053842814
3.5.1.28	0.074305824	0.027771604	0.084376563	0.064235085	0.002535725	0.016084268	0.375271385
2.4.1.182	0.00996067	0.004358938	0.008584263	0.011337077	0.035508142	0.105854462	-0.492075554
5.4.99.9	0.016902676	0.010230055	0.022102864	0.011702489	0.000250456	0.002863767	0.652844116
2.8.4.5	0.019377037	0.007322043	0.016305305	0.02244877	0.004500767	0.024521421	-0.68182805
1.10.3.14	0.074109989	0.051938443	0.091179125	0.057040853	0.005166141	0.026722477	0.512158055

EC	Mean abundance	Std_deviation	Mean abundance - health	Mean abundance - peri-implantitis	P value	FDR corrected P value	LDA correlation (CAP)
2.3.1.9	0.092343693	0.063346763	0.077162392	0.107524994	0.055961569	0.144949638	-0.550586192
6.3.2.8	0.049076851	0.011744526	0.052427214	0.045726487	0.06461309	0.158457409	0.2927703
2.1.1.190	0.018143467	0.00583824	0.017836241	0.018450693	0.975559196	0.997952845	0.01042119
2.6.1.9	0.006945122	0.004660577	0.006686463	0.007203781	0.767049595	0.857319741	-0.181502388
6.3.1.1	0.023876659	0.013117477	0.02694971	0.020803607	0.493199174	0.639686689	-0.075119409
6.1.1.21	0.04800497	0.006847267	0.047331017	0.048678923	0.943010334	0.976729416	-0.164785063
4.3.2.1	0.010712516	0.007322461	0.01259471	0.008830321	0.230034676	0.388910766	0.325119409
3.5.4.1	0.000391711	0.001208111	0.000594711	0.000188711	0.54207966	0.684085511	0.323795862
2.4.1.129	0.03336742	0.016652906	0.041555768	0.025179071	5.78771E-05	0.001058846	0.771710812
6.1.1.15	0.076708039	0.012498743	0.078971132	0.074444946	0.07095965	0.169174036	-0.011723838
6.3.1.19	0.000916992	0.001030006	0.001117456	0.000716528	0.044364089	0.123763702	0.43122354
3.4.11.1	0.021548932	0.00851517	0.019881998	0.023215866	0.101506067	0.216463245	-0.496200608
2.4.1.18	0.098109995	0.056741055	0.105706439	0.090513551	0.573959013	0.706974319	0.038970908
2.8.4.3	0.037032091	0.013244267	0.033099282	0.040964899	0.067726454	0.164397453	-0.5040165
5.4.2.2	0.126990457	0.046773865	0.122514584	0.131466331	0.602179894	0.731368732	-0.251628311
1.7.99.1	0.039270333	0.027556607	0.041423575	0.03711709	0.373099055	0.52125897	0.241749891
3.4.21.102	0.062739427	0.025394279	0.049337484	0.076141369	4.69671E-05	0.000927601	-0.714937039
1.14.14.12	6.125E-07	4.19909E-06	0	0.000001225	0.3378947	0.489384158	-0.163202356
1.8.1.4	0.146401892	0.135267846	0.195637289	0.097166495	0.001083935	0.008447885	0.726877985
2.3.1.61	0.014167863	0.014832288	0.016795433	0.011540294	0.061616509	0.154307626	0.564155449
2.3.1.128	0.016659763	0.008521076	0.022004073	0.011315454	9.83425E-07	7.12164E-05	0.765414677
6.3.2.6	0.025043288	0.010907751	0.019841089	0.030245488	0.000718634	0.006152642	-0.62299175
4.3.1.19	0.022371624	0.009109994	0.024051068	0.020692179	0.147193088	0.284246208	0.120495007
3.3.1.1	0.007318844	0.00376488	0.005942672	0.008695016	0.002535725	0.016084268	-0.399913157
3.4.22.40	0.082747881	0.039157515	0.069222596	0.096273166	0.012020504	0.048360269	-0.673143726
1.3.98.1	0.007745209	0.008728099	0.00554781	0.009942608	0.033682558	0.102522391	-0.540165002
3.1.2.6	0.008896561	0.0034247	0.0097407	0.008052422	0.07095965	0.169174036	0.27887538
3.4.23.43	0.01186579	0.009976995	0.010870006	0.012861573	0.926767515	0.966239916	-0.138189318
3.2.1.51	0.05220713	0.065645142	0.062712563	0.041701696	0.846132513	0.915107845	0.003256622
3.4.11.4	0.05169229	0.017618958	0.045512207	0.057872374	0.017242287	0.063355381	-0.698762484
6.3.4.4	0.057796184	0.01196609	0.06015205	0.055440318	0.130510373	0.26012274	0.035280069
1.7.2.5	0.024589434	0.027925727	0.025049649	0.024129219	0.074315735	0.175014563	0.587928789
2.1.1.177	0.006320694	0.001747589	0.006526029	0.006115359	0.165425496	0.302323356	0.054928354
2.7.2.8	0.002843017	0.002009001	0.002875841	0.002810193	0.878244653	0.93357138	-0.052323057
2.3.2.6	0.000667264	0.000677118	0.000619388	0.000715139	0.613421744	0.737804146	0.196542459
4.1.99.17	0.01125201	0.010639761	0.007121083	0.015382937	0.006327364	0.030632198	-0.707338254
5.1.3.29	0.002153974	0.004036071	0.001911406	0.002396543	0.163929165	0.302323356	-0.006513951
2.3.1.31	0.002515124	0.002115998	0.002888008	0.00214224	0.141463386	0.276250972	0.568823274
3.1.21.3	0.062816747	0.04393913	0.055384626	0.070248868	0.991851953	1	-0.072079896
2.7.1.15	0.00638332	0.010670431	0.007487139	0.005279501	0.894378126	0.943880803	0.369300912
2.4.1.25	0.113315534	0.061450217	0.12434142	0.102289648	0.272703911	0.444198124	0.224706904
5.1.3.9	0.015711114	0.017592806	0.019271068	0.01215116	0.407115953	0.554954923	0.167173252
6.3.2.13	0.032879809	0.00776282	0.034118239	0.03164138	0.222093193	0.379172858	0.127659574
6.1.1.4	0.090825358	0.014489314	0.096067654	0.085583063	0.003644805	0.020906504	0.38797221
6.2.1.3	0.069410266	0.025994068	0.054940782	0.083879749	7.8682E-05	0.001314898	-0.834129396
2.8.1.13	0.035255266	0.008962232	0.037855758	0.032654774	0.058733676	0.149456998	0.290056448
1.8.1.8	0.038593022	0.023910286	0.025327403	0.051858641	5.38649E-06	0.000246361	-0.862679114
2.7.1.48	0.025105557	0.009955479	0.023702273	0.026508842	0.846132513	0.915107845	-0.287234043
2.4.1.21	0.09040394	0.072777375	0.085241868	0.095566013	0.878244653	0.93357138	-0.03636561
3.2.1.52	0.073068101	0.06789772	0.0840582	0.062078002	0.630998004	0.750119378	-0.031155015
1.11.1.6	0.017050017	0.017220416	0.023352308	0.010747727	0.000228358	0.002681659	0.662722536
6.3.4.19	0.01365421	0.008560443	0.009937135	0.017371286	0.000606942	0.005581297	-0.787234043
6.1.1.11	0.053985194	0.011988122	0.050621562	0.057348825	0.135903357	0.267800492	-0.433456361
2.7.1.2	0.054984975	0.015662516	0.05018542	0.059784531	0.030266661	0.09546907	-0.561115936
5.4.2.9	0.001895556	0.003170153	0.000686265	0.003104847	0.001609529	0.011371391	-0.566000868
2.1.1.170	0.012337221	0.003050974	0.012802437	0.011872006	0.028671131	0.091431973	0.114741641
2.7.7.9	0.034545033	0.022809241	0.04370924	0.025380827	0.001083935	0.008447885	0.735128094

EC	Mean abundance	Std_deviation	Mean abundance - health	Mean abundance - peri-implantitis	P value	FDR corrected P value	LDA correlation (CAP)
1.11.1.15	0.38238136	0.169977867	0.396948457	0.367814263	1	1	-0.102366479
1.97.1.4	0.062439411	0.028764637	0.073186003	0.051692819	0.004500767	0.024521421	0.349652627
1.3.8.7	0.000686983	0.001594683	0.001020046	0.00035392	0.037103577	0.109483898	0.249158002
3.5.3.12	0.010150131	0.007857362	0.006766287	0.013533975	0.000359831	0.003635969	-0.66739036
2.7.6.5	0.066839581	0.019322417	0.076471299	0.057207863	0.000359831	0.003635969	0.487299175
1.1.1.219	0.002958235	0.002495938	0.002632117	0.003284352	0.053297186	0.140349256	-0.142097264
5.1.3.3	0.035924873	0.029694089	0.034047818	0.037801929	0.019369559	0.06912586	-0.430199739
1.11.1.5	0.00155181	0.001588875	0.001559483	0.001544138	0.675265662	0.786603029	0.184107686
2.7.9.2	0.018637846	0.01359651	0.020107626	0.017168067	0.705414169	0.811389693	0.374402953
6.3.4.2	0.048531148	0.010165635	0.046951155	0.050111142	0.30070683	0.47991595	-0.340316978
6.3.4.15	0.014720607	0.007757703	0.015675398	0.013765817	0.110554262	0.229561897	0.254559271
2.5.1.6	0.06769142	0.017069492	0.062250716	0.073132123	0.009969192	0.042259647	-0.526812853
6.3.2.10	0.036079428	0.009749008	0.037224603	0.034934252	0.467672658	0.616703398	0.083043856
2.7.7.72	0.014527298	0.005087445	0.017300154	0.011754442	1.75724E-05	0.000484774	0.524316109
3.1.22.4	0.007785913	0.00313707	0.006262351	0.009309475	0.000781202	0.006496311	-0.636343899
3.5.2.6	0.111160034	0.363953704	0.183792199	0.038527869	0.000189424	0.002334886	0.614198871
2.1.1.35	0.000840963	0.001212551	0.001105749	0.000576177	0.110554262	0.229561897	0.439209726
2.1.2.1	0.086812521	0.042508068	0.066819748	0.106805295	0.000106192	0.001604884	-0.756187581
3.5.2.7	0.015785035	0.007412103	0.012530674	0.019039395	0.001271988	0.009488051	-0.690186713
2.5.1.47	0.061537786	0.044539832	0.074365277	0.048710295	0.018279409	0.066186695	0.594007816
6.2.1.5	0.0504828	0.039714454	0.038452303	0.062513297	0.02051487	0.072168252	-0.130373426
2.7.1.107	0.005587905	0.00576331	0.007292794	0.003883016	0.000660648	0.005888234	0.664025185
1.12.5.1	0.000822137	0.001315295	0.000625122	0.001019153	0.311861368	0.489384158	-0.232146358
1.2.7.1	0.30579601	0.147760691	0.232794347	0.378797673	0.000660648	0.005888234	-0.818931828
1.17.7.4	0.129573027	0.059012938	0.134020889	0.125125165	0.959276437	0.987691024	-0.162396874
1.8.2.2	0.000107857	0.000152632	7.5364E-05	0.00014035	0.008588498	0.037599017	-0.412196351
3.4.21.92	0.058616858	0.014476312	0.063142114	0.054091603	0.035508142	0.105854462	0.473838472
2.1.1.192	0.025707023	0.005598029	0.026907041	0.024507006	0.043657636	0.122382211	0.468736431
4.2.3.5	0.020547881	0.006323125	0.020957713	0.020138049	0.310444458	0.489384158	-0.092705167
5.1.1.7	0.007169197	0.004684954	0.00756078	0.006777614	0.310444458	0.489384158	0.43128528
2.7.8.5	0.011572219	0.004212868	0.013732561	0.009411876	9.61681E-05	0.001505766	0.554711246
3.1.3.7	0.001069972	0.001173465	0.001094184	0.001045761	0.862160642	0.924389386	-0.049066435
3.1.4.46	0.02864351	0.011070078	0.029510596	0.027776424	0.846132513	0.915107845	-0.107359965
6.3.2.45	0.001265913	0.002051063	0.001516083	0.001015743	0.135903357	0.267800492	0.463742944
6.1.1.10	0.075579318	0.013861986	0.079104355	0.072054281	0.06461309	0.158457409	0.19973947
1.3.1.98	0.026167749	0.006156061	0.024141498	0.028194	0.031936275	0.099293822	-0.512592271
3.6.3.8	0.064168872	0.035364128	0.078243684	0.05009406	0.001609529	0.011371391	0.798849327
1.1.1.22	0.016282705	0.009414798	0.012157602	0.020407807	0.001174568	0.008953503	-0.61148502
3.4.13.21	0.007731339	0.004436872	0.007063323	0.008399355	0.384241116	0.530429754	-0.134715588
1.4.1.1	0.024424018	0.027438861	0.021304764	0.027543273	0.751487275	0.845559951	0.244029527
3.2.1.26	0.012558202	0.007607187	0.015192989	0.009923414	0.008230024	0.036374662	0.516717325
3.6.3.54	0.097439055	0.06135577	0.115070258	0.079807852	0.031936275	0.099293822	0.353234911
4.3.1.7	0.023558528	0.023971161	0.025008912	0.022108143	0.48034525	0.628644612	0.001411203
3.1.2.21	0.007303511	0.002657228	0.007927016	0.006680007	0.125281744	0.252891604	0.443009119
2.7.1.180	0.037010226	0.022115949	0.042636276	0.031384176	0.281836841	0.455448476	0.46417716
2.3.1.41	0.01384966	0.008321143	0.01112718	0.016572141	0.006762554	0.031938366	-0.261181068
6.1.1.6	0.095815261	0.017356991	0.089017897	0.102612625	0.011299384	0.045991404	-0.643834129
4.2.1.46	0.033850251	0.009962775	0.035312494	0.032388009	0.310444458	0.489384158	0.000651324
1.17.1.8	0.022893446	0.00844046	0.023810163	0.021976728	0.061616509	0.154307626	0.245223621
2.7.7.71	0.001840701	0.001999103	0.00094062	0.002740783	0.006478355	0.03118942	-0.38091103
2.2.1.6	0.055098308	0.032344322	0.073226259	0.036970358	8.70215E-05	0.001440412	0.694420321
5.1.1.4	0.007513937	0.007807902	0.006614401	0.008413474	0.751487275	0.845559951	-0.174663482
3.6.1.55	0.018846047	0.007523931	0.022913323	0.014778771	5.78771E-05	0.001058846	0.552974381
6.1.1.19	0.067654917	0.013519663	0.066106879	0.069202955	0.878244653	0.93357138	-0.160442901
4.1.1.31	0.030873017	0.019477717	0.040768814	0.02097722	0.000189424	0.002334886	0.684324794
2.3.1.234	0.027031741	0.006654864	0.028381147	0.025682335	0.061616509	0.154307626	0.278441164
1.1.1.88	0.001271951	0.002035421	0.001351457	0.001192445	0.926001519	0.966239916	0.271100006

EC	Mean abundance	Std_deviation	Mean abundance - health	Mean abundance - peri-implantitis	P value	FDR corrected P value	LDA correlation (CAP)
2.4.2.9	0.05063076	0.015344515	0.058721219	0.0425403	0.000106192	0.001604884	0.64893617
2.7.2.11	0.009084072	0.004928056	0.011834795	0.00633335	0.000129181	0.001781874	0.552105949
4.99.1.3	0.069377933	0.035938446	0.058582824	0.080173043	0.031936275	0.099293822	-0.528766826
2.4.2.29	0.031219015	0.007419264	0.029438218	0.032999811	0.246506703	0.409285723	-0.534845853
2.7.1.56	0.017688297	0.016449221	0.021404835	0.01397176	0.022979765	0.078774815	0.379179331
5.4.3.8	0.014957481	0.007450136	0.016063304	0.013851657	0.573959013	0.706974319	0.243161094
2.7.1.148	0.024754506	0.010485593	0.020600697	0.028908316	0.002936794	0.017909288	-0.506621798
5.1.3.15	0.000714915	0.00108351	0.00096538	0.00046445	0.018279409	0.066186695	0.593790708
3.2.1.50	0.000968164	0.00122265	0.00074626	0.001190068	0.288013773	0.463918386	-0.379493919
1.6.5.8	0.089111174	0.042939929	0.067395065	0.110827284	1.96718E-05	0.000525994	-0.530178029
2.8.1.4	0.011014057	0.006480288	0.014789784	0.00723833	1.24541E-05	0.000408399	0.899696049
2.7.7.39	0.005131271	0.005172429	0.003190435	0.007072107	0.007712559	0.034998504	-0.510204082
4.6.1.1	0.013983388	0.016607447	0.009581193	0.018385583	0.024303877	0.081387551	-0.491532783
2.7.7.3	0.011945987	0.003442215	0.012220881	0.011671094	0.48034525	0.628644612	-0.132110291
1.4.1.16	0.015313054	0.008704707	0.014539622	0.016086486	0.230034676	0.388910766	-0.394702562
2.7.4.29	3.6555E-05	0.000126615	6.52642E-05	7.84583E-06	0.93385104	0.973041432	-0.058740507
3.4.23.51	0.000483694	0.000657001	0.000640339	0.000327049	0.056412457	0.145683285	0.240783982
5.4.4.2	0.007759126	0.004809978	0.00792076	0.007597492	0.943010334	0.976729416	-0.021819366
3.1.3.25	0.005824178	0.004279454	0.007843193	0.003805162	4.69671E-05	0.000927601	0.855731654
1.4.4.2	0.052766073	0.04754211	0.030381692	0.075150453	0.000250456	0.002863767	-0.729483283
4.2.1.24	0.010687614	0.004191294	0.011181349	0.010193878	0.493199174	0.639686689	0.063938341
2.7.7.65	0.004540448	0.005797179	0.003753887	0.005327009	0.395580536	0.543063958	-0.133847156
2.6.1.102	0.008707549	0.006284399	0.011349595	0.006065503	0.006327364	0.030632198	0.574142423
3.2.1.11	0.002071255	0.004248285	0.003575553	0.000566957	0.024303877	0.081387551	0.391011724
5.4.2.11	0.072901987	0.033225638	0.087356448	0.058447526	0.002354183	0.015324232	0.611376465
6.1.1.3	0.121448223	0.02762476	0.118116336	0.124780109	0.130510373	0.26012274	-0.251519757
2.7.7.56	0.007819028	0.003952036	0.008533122	0.007104934	0.73602349	0.836084199	-0.101389492
2.2.1.2	0.020068671	0.010266512	0.02227308	0.017864263	0.165425496	0.302323356	0.316435085
1.3.1.9	0.043154797	0.018152922	0.047054805	0.039254789	0.135903357	0.267800492	0.3435736
6.1.1.9	0.081483932	0.012763215	0.085434258	0.077533606	0.024303877	0.081387551	0.167933131
2.7.7.70	0.000139108	0.00029582	0.000100338	0.000177877	0.991407149	1	0.342117801
6.1.1.20	0.130150296	0.017813966	0.126556738	0.133743854	0.30070683	0.47991595	-0.413265306
6.1.1.5	0.128788351	0.023402225	0.123405737	0.134170966	0.214346279	0.37068043	-0.572839774
1.1.1.335	0.004768161	0.005122933	0.003232403	0.006303919	0.043302463	0.121790181	-0.389231145
1.3.99.38	0.000454246	0.000749149	0.000661606	0.000246885	0.010837031	0.044632131	0.37916808
2.7.1.40	0.145446312	0.067707002	0.167656634	0.12323599	0.003912486	0.02200615	0.608445506
5.4.2.10	0.042073695	0.01427619	0.049626651	0.034520738	0.000274495	0.00305816	0.767151541
1.6.3.4	0.027947193	0.017443308	0.026042089	0.029852297	0.045920684	0.125487655	-0.108662614
2.5.1.9	0.015117066	0.006410984	0.013353337	0.016880796	0.005530342	0.027941089	-0.448762484
2.3.1.15	0.044219164	0.011457443	0.048246405	0.040191922	0.027147143	0.088025623	0.440078159
4.2.1.20	0.027468306	0.017936522	0.033867226	0.021069387	0.007712559	0.034998504	0.548523665
2.5.1.75	0.020151663	0.006928363	0.018137881	0.022165444	0.185272618	0.32924725	-0.468519323
2.8.1.7	0.069720986	0.033712323	0.079286876	0.060155095	0.007223852	0.033569663	0.364198871
4.1.2.4	0.023265782	0.006040096	0.024033382	0.022498182	0.48034525	0.628644612	0.036148502
3.1.26.5	0.016971367	0.011884657	0.017553735	0.016389	0.085151945	0.189979564	0.297112462
3.4.21.89	0.037100498	0.009641038	0.035875231	0.038325764	0.395580536	0.543063958	-0.157403387
3.2.1.196	0.001404757	0.002138137	0.001825327	0.000984186	0.380394958	0.52890115	0.330372572
6.6.1.2	0.048558507	0.038554601	0.029044963	0.06807205	5.78771E-05	0.001058846	-0.795918367
2.5.1.19	0.019537937	0.005595282	0.019516435	0.01955944	0.675265662	0.786603029	-0.253148068
2.7.7.8	0.160885686	0.038487547	0.145995082	0.17577629	0.00591703	0.029309765	-0.405449414
4.2.1.2	0.055823539	0.031055466	0.054754781	0.056892296	0.418845832	0.565922893	-0.164025185
2.6.1.42	0.046175913	0.016892678	0.042875238	0.049476588	0.532819632	0.676479136	-0.273773339
2.7.8.6	0.006006443	0.004108436	0.005585495	0.006427391	0.159171691	0.299914766	-0.356057317
2.4.2.14	0.036294389	0.013020413	0.029991057	0.04259772	0.000921373	0.007413637	-0.547221016
5.1.1.1	0.02349483	0.010562274	0.019416545	0.027573115	0.012020504	0.048360269	-0.639383413
3.2.1.17	0.002193356	0.005783708	0.002608099	0.001778612	0.846132513	0.915107845	0.054928354
2.4.2.22	0.008591264	0.004797499	0.011129708	0.00605282	4.69671E-05	0.000927601	0.579353018

EC	Mean abundance	Std_deviation	Mean abundance - health	Mean abundance - peri-implantitis	P value	FDR corrected P value	LDA correlation (CAP)
6.1.1.16	0.049927714	0.006589252	0.048667869	0.051187558	0.130510373	0.26012274	-0.488384716
3.4.11.5	0.008402323	0.005050945	0.008087754	0.008716892	0.310444458	0.489384158	-0.14090317
2.3.1.39	0.053447697	0.014190206	0.052064185	0.054831209	0.56008305	0.699299095	-0.112244898
3.1.31.1	0.001391597	0.00246205	0.001687199	0.001095995	0.73602349	0.836084199	-0.126139818
2.7.3.9	0.244881811	0.16340337	0.334400283	0.15536334	3.41151E-05	0.000796167	0.711137647
2.7.7.59	0.001654009	0.002351627	0.002128538	0.001179481	0.44288188	0.590282751	0.386018237
2.6.1.1	0.032679857	0.01322472	0.028742574	0.036617139	0.058733676	0.149456998	-0.577290491
3.5.4.33	0.005052455	0.001744459	0.005108366	0.004996544	0.878244653	0.93357138	-0.011832393
2.7.6.3	0.010031975	0.009368501	0.009647821	0.010416129	0.089030196	0.195611925	-0.592596613
4.2.1.136	0.000270428	0.000387664	0.000330489	0.000210367	0.150503377	0.28935273	0.422459236
2.4.2.3	0.012001693	0.006716788	0.008934275	0.015069112	0.000329015	0.00344475	-0.786691272
3.4.23.36	0.018210809	0.004195594	0.017072274	0.019349343	0.147193088	0.284246208	-0.457881025
3.5.1.4	0.004285181	0.004895227	0.00559935	0.002971012	0.017242287	0.063355381	0.655775076
2.7.7.87	0.026444253	0.006913667	0.028106067	0.024782438	0.074315735	0.175014563	0.264003474
5.1.3.1	0.023774905	0.004994344	0.022904493	0.024645318	0.159171691	0.299914766	-0.456795484
3.4.17.19	0.007794873	0.003406042	0.006844454	0.008745292	0.030266661	0.09546907	-0.570885801
3.4.13.19	0.012601212	0.007844202	0.008881797	0.016320627	0.000557234	0.005178994	-0.829787234
1.7.2.1	0.005346455	0.006970219	0.005475224	0.005217687	0.546368678	0.686615157	0.519756839
3.5.99.10	0.044123295	0.017855891	0.044042973	0.044203617	1	1	-0.334563613
5.3.1.24	0.004885503	0.004454021	0.00682847	0.002942537	0.000718634	0.006152642	0.450825011
3.6.1.66	0.026153226	0.007120729	0.026109146	0.026197306	0.894378126	0.943880803	-0.403278333
6.3.2.1	0.011061155	0.003955342	0.009842153	0.012280157	0.055961569	0.144949638	-0.455709944
3.5.1.42	0.020540476	0.005883027	0.021501268	0.019579685	0.384241116	0.530429754	-0.114958749
3.1.1.29	0.014890514	0.004164768	0.015594034	0.014186995	0.255039588	0.418959172	0.121037777
2.7.1.165	0.007440866	0.004769087	0.008936772	0.00594496	0.013583315	0.052578624	0.365610074
3.5.1.19	0.007131064	0.005121224	0.006928327	0.0073338	0.943010334	0.976729416	0.144268346
1.5.1.3	0.026067347	0.02450783	0.03656824	0.015566454	3.10079E-07	4.89924E-05	0.750759878
2.7.1.11	0.09668954	0.043073203	0.114981793	0.078397288	0.001083935	0.008447885	0.609313938
6.3.5.2	0.067825131	0.014129773	0.073065949	0.062584314	0.005530342	0.027941089	0.477963526
5.3.1.6	0.036421607	0.013521627	0.033793875	0.03904934	0.165425496	0.302323356	-0.38102475
6.2.1.1	0.002824057	0.003438295	0.003843649	0.001804465	0.074315735	0.175014563	0.43877551
2.1.1.185	0.014110557	0.00329602	0.015283799	0.012937314	0.024303877	0.081387551	0.503799392
2.7.7.18	0.017932231	0.00954313	0.02188077	0.013983692	0.000359831	0.003635969	0.524207555
1.1.1.3	0.027135575	0.016224796	0.035847459	0.018423692	0.000117169	0.001682975	0.732739904
3.1.1.5	0.001331592	0.001881633	0.001864585	0.000798598	0.110554262	0.229561897	0.356165871
3.4.14.5	0.024326191	0.01531468	0.015441207	0.033211175	1.56822E-05	0.000461959	-0.874294399
4.2.1.44	0.000814275	0.000786116	0.000635368	0.000993183	0.219859194	0.378807165	-0.179716123
3.1.3.3	0.023162511	0.014267324	0.017083079	0.029241944	0.003393537	0.020061115	-0.740990013
6.3.2.4	0.03655471	0.010358501	0.039286133	0.033823287	0.085151945	0.189979564	0.497286149
2.5.1.74	0.011321068	0.005614874	0.010060018	0.012582117	0.165425496	0.302323356	-0.502062527
2.4.1.211	0.002205526	0.00285003	0.002607881	0.001803171	0.081408821	0.184237696	0.616912723
1.7.2.2	0.023244804	0.016741699	0.015827533	0.030662076	0.000156664	0.002031961	-0.69973947
3.4.21.105	0.011893428	0.004413381	0.011415646	0.01237121	0.34086786	0.490420812	-0.222535823
1.14.15.20	0.001173952	0.003183678	0.00146319	0.000884713	0.588975588	0.722176129	0.226416861
3.2.2.21	0.001875153	0.002302204	0.002088339	0.001661968	0.991851953	1	0.084672167
1.17.99.6	0.010972062	0.004953087	0.011653718	0.010290405	0.105955089	0.221867402	0.356057317
4.2.1.45	0.00164519	0.001660532	0.001128325	0.002162054	0.007223852	0.033569663	-0.274424663
4.4.1.21	0.008985082	0.003246299	0.0098993	0.008070863	0.048279687	0.129691029	0.57218845
3.6.1.23	0.013576049	0.00272575	0.013571559	0.013580539	0.720663933	0.822924974	-0.141554494
2.5.1.39	0.002146023	0.001910164	0.002191854	0.002100193	0.846132513	0.915107845	0.340316978
5.4.99.12	0.01292855	0.005324438	0.014536138	0.011320963	0.00419749	0.023307469	0.453430308
6.1.1.13	0.02819114	0.021066139	0.038505808	0.017876473	1.56822E-05	0.000461959	0.789622232
2.5.1.129	0.000377107	0.000327693	0.000223724	0.000530491	0.000319313	0.00344475	-0.325859179
2.4.1.11	0.011476639	0.00866595	0.007779068	0.015174209	0.000781202	0.006496311	-0.735236648
3.1.1.83	0.000079598	0.000154973	6.66452E-05	9.25508E-05	0.32706278	0.489384158	0.054657203
6.1.1.1	0.0565862	0.009106948	0.057162338	0.056010062	0.255039588	0.418959172	0.020950934
2.5.1.7	0.068296623	0.019696031	0.08048474	0.056108506	6.09052E-06	0.000252032	0.779526704

EC	Mean abundance	Std_deviation	Mean abundance - health	Mean abundance - peri-implantitis	P value	FDR corrected P value	LDA correlation (CAP)
2.7.1.12	0.00319186	0.010674685	0.005209848	0.001173871	0.120214723	0.24493926	0.502496743
2.3.1.241	0.016341908	0.008521367	0.016498207	0.016185609	0.943010334	0.976729416	-0.074468085
3.5.4.13	0.003465713	0.002378492	0.003705846	0.003225579	0.493199174	0.639686689	0.123100304
3.4.17.14	0.013914297	0.019336442	0.021823625	0.006004969	0.000117169	0.001682975	0.609313938
3.2.1.68	0.002788718	0.003289784	0.002018314	0.003559122	0.190404489	0.337332316	-0.142535349
3.4.17.13	0.012883398	0.010373531	0.012448353	0.013318443	0.943010334	0.976729416	-0.15881459
3.4.21.83	0.004480987	0.003337932	0.003376293	0.005585681	0.085151945	0.189979564	-0.141554494
2.7.1.45	0.005570886	0.003650888	0.003598481	0.007543291	4.69671E-05	0.000927601	-0.563721233
3.5.1.18	0.042611699	0.019564222	0.053286852	0.031936545	1.4826E-06	9.54357E-05	0.710811984
1.2.4.1	0.151093386	0.231515088	0.220151029	0.082035743	0.000557234	0.005178994	0.87299175
1.1.1.31	0.003740489	0.003813844	0.003321476	0.004159501	0.17185877	0.312437805	0.12407729
2.5.1.54	0.026593062	0.014412937	0.034384205	0.018801919	3.06131E-05	0.000760078	0.639274859
4.1.3.36	0.006571113	0.004130523	0.006740987	0.00640124	0.690279643	0.800337572	-0.003365176
2.7.4.22	0.049245499	0.012050809	0.053835531	0.044655468	0.00187728	0.012845322	0.421841077
5.4.2.8	0.029887646	0.016371698	0.02339459	0.036380703	0.003644805	0.020906504	-0.567086409
3.2.1.55	0.00140099	0.002272807	0.001151626	0.001650354	0.814269221	0.893434284	0.003148068
5.1.1.13	0.005345151	0.005861312	0.005799537	0.004890766	0.17185877	0.312437805	0.241424229
4.1.1.70	0.054344252	0.030215275	0.048619907	0.060068597	0.101506067	0.216463245	-0.456035606
2.7.1.50	0.008721115	0.010154095	0.013261935	0.004180296	0.007223852	0.033569663	0.499565784
2.7.6.2	0.00869627	0.004447225	0.010399001	0.006993539	0.009969192	0.042259647	0.297112462
2.1.1.289	0.00496362	0.004577673	0.006131215	0.003796024	0.407115953	0.554954923	0.158706036
2.4.2.8	0.018646084	0.007510965	0.017775115	0.019517054	0.050737544	0.134834635	-0.430525402
2.3.1.191	0.033722272	0.013429566	0.027641241	0.039803302	0.001271988	0.009488051	-0.518671298
6.3.1.20	0.019892992	0.023760698	0.027663896	0.012122088	0.002354183	0.015324232	0.708315241
4.2.3.1	0.02768221	0.01312337	0.034499632	0.020864787	0.000106192	0.001604884	0.664676509
3.5.1.115	0.000654835	0.001188972	0.000984416	0.000325255	0.019083522	0.068527191	0.408132675
2.8.1.6	0.021494415	0.010465062	0.023045971	0.019942858	0.573959013	0.706974319	0.056231003
3.1.13.1	0.001957908	0.002546683	0.0019765	0.001939317	0.828588496	0.906284962	0.101555079
3.4.24.71	0.027099632	0.016148068	0.016985062	0.037214202	7.4311E-07	6.7975E-05	-0.836517586
3.2.1.1	0.03334617	0.015683503	0.028497469	0.038194871	0.07095965	0.169174036	-0.503690838
3.1.3.11	0.050292608	0.020137059	0.042263554	0.058321662	0.008777645	0.038138866	-0.800369084
6.3.5.3	0.074258208	0.027859104	0.058232627	0.090283788	2.74461E-05	0.000691325	-0.736756405
2.7.7.41	0.026150421	0.006868667	0.026791815	0.025509027	0.675265662	0.786603029	0.133195832
3.1.26.8	0.008389342	0.002987811	0.009983742	0.006794943	6.87931E-06	0.000271733	0.527247069
3.4.21.72	0.003472192	0.006334136	0.005145754	0.001798631	0.00591703	0.029309765	0.453864525
2.7.1.23	0.018879853	0.005235066	0.019789177	0.017970528	0.07095965	0.169174036	0.276487191
2.7.4.6	0.009162237	0.006321475	0.010601367	0.007723108	0.192257401	0.338887791	0.514654798
3.6.3.20	0.00022511	0.000309449	0.000234953	0.000215268	1	1	-0.097741197
6.3.4.20	0.006398311	0.002929054	0.006796518	0.006000105	0.418845832	0.565922893	-0.150564481
2.3.1.51	0.013863475	0.00551124	0.014318228	0.013408723	0.384241116	0.530429754	0.457989579
2.7.4.25	0.024599428	0.007212462	0.028095671	0.021103185	0.000429499	0.004217336	0.668258793
3.2.1.86	0.013999791	0.019851557	0.015905186	0.012094395	0.012781229	0.050257413	0.660117238
5.3.1.13	0.016335911	0.0063945	0.013854786	0.018817036	0.009969192	0.042259647	-0.699413808
3.1.3.96	1.83252E-05	5.84483E-05	5.31667E-06	3.13338E-05	0.095655351	0.20781125	0.080941474
2.3.1.265	0.000268942	0.000347536	0.000299044	0.000238841	0.297004532	0.476633312	0.289519796
4.1.2.48	0.006225726	0.003958149	0.004294291	0.008157161	0.000208058	0.002493826	-0.798523665
2.3.1.180	0.115282389	0.039564929	0.101971058	0.12859372	0.031936275	0.099293822	-0.298306557
3.1.11.6	0.029421855	0.006545403	0.030039265	0.028804446	0.130510373	0.26012274	0.066109422
3.2.1.18	0.031876377	0.022649149	0.037541178	0.026211576	0.07095965	0.169174036	0.193334781
2.4.2.10	0.01067552	0.004700959	0.012481851	0.008869189	0.011299384	0.045991404	0.612462006
2.7.1.35	0.013147645	0.008546854	0.015752563	0.010542727	0.010616164	0.043930696	0.29385584
2.7.1.121	0.02638786	0.017675259	0.027429384	0.025346336	0.814269221	0.893434284	-0.385584021
2.7.1.193	0.019244654	0.011801125	0.017245672	0.021243635	0.34086786	0.490420812	-0.299174989
2.7.6.1	0.076076379	0.019904284	0.079886331	0.072266428	0.192257401	0.338887791	0.419344333
1.1.1.27	0.083718702	0.088530502	0.11469411	0.052743293	0.00137664	0.01009536	0.471993053
3.6.1.27	0.028625593	0.008081574	0.028313897	0.028937289	0.56008305	0.699299095	0.033108988
2.1.2.9	0.026521152	0.00697034	0.025653377	0.027388926	0.573959013	0.706974319	-0.224924012

EC	Mean abundance	Std_deviation	Mean abundance - health	Mean abundance - peri-implantitis	P value	FDR corrected P value	LDA correlation (CAP)
3.1.11.2	0.023756671	0.005370633	0.024135546	0.023377795	0.519439475	0.663813094	0.168910117
1.2.1.41	0.010285482	0.005718046	0.013686393	0.006884571	8.74923E-06	0.000316795	0.595093356
3.4.21.88	0.022140673	0.011808159	0.019134846	0.0251465	0.105955089	0.221867402	-0.310464611
5.3.1.23	0.004028597	0.002069501	0.003729795	0.004327398	0.141463386	0.276250972	-0.481654364
1.13.12.16	0.020667367	0.013353878	0.017146852	0.024187881	0.222093193	0.379172858	-0.163482414
3.1.2.20	0.000826715	0.001248324	0.001182154	0.000471277	0.09037306	0.197570287	0.587481339
2.3.1.207	1.52289E-05	2.74485E-05	2.49454E-05	5.5125E-06	0.012609526	0.050257413	0.16167311
4.1.1.65	0.023629723	0.009605275	0.018663842	0.028595604	0.000329015	0.00344475	-0.794832827
3.4.11.2	0.038964073	0.028662992	0.053225071	0.024703075	7.8682E-05	0.001314898	0.804928354
6.3.4.18	0.006422642	0.005654061	0.009039334	0.00380595	0.000156664	0.002031961	0.822297004
2.6.1.57	0.006297922	0.00582617	0.006785963	0.005809882	0.602179894	0.731368732	0.159574468
2.4.2.18	0.002741756	0.002689145	0.003464437	0.002019075	0.035508142	0.105854462	0.494463743
4.2.1.11	0.386888067	0.16504021	0.426647958	0.347128176	0.017242287	0.063355381	0.501845419
3.2.1.96	0.038743175	0.062026574	0.056619489	0.020866862	0.013583315	0.052578624	0.577181937
2.7.8.13	0.030921202	0.007989986	0.030328659	0.031513745	0.720663933	0.822924974	-0.046678246
3.6.3.2	0.015403744	0.01408408	0.020899575	0.009907913	2.74461E-05	0.000691325	0.696482848
2.1.1.182	0.017456157	0.004211635	0.017275021	0.017637294	0.506231587	0.649800959	0.13048198
3.4.21.26	0.011334092	0.008453656	0.006637559	0.016030625	1.3982E-05	0.000433941	-0.66391663
3.1.3.73	0.005834801	0.003502	0.004506405	0.007163197	0.014428574	0.05535731	-0.744897959
2.1.1.266	0.00115747	0.002826328	0.001227144	0.001087795	0.369287882	0.518636598	0.243772691
2.7.7.73	0.001459001	0.001407255	0.000883533	0.002034469	0.002729685	0.017004276	-0.412939644
3.1.13.5	0.002599107	0.002741552	0.003312515	0.0018857	0.048279687	0.129691029	0.401541468
2.4.1.337	0.015201744	0.010286195	0.020705205	0.009698284	9.61681E-05	0.001505766	0.731437256
6.3.2.2	0.009937796	0.013802604	0.015555519	0.004320072	8.74923E-06	0.000316795	0.797980894
3.1.26.12	0.014396797	0.015958052	0.020634623	0.008158972	0.000468756	0.004501094	0.582718194
1.1.1.136	0.010089122	0.008873259	0.00736849	0.012809753	0.003157811	0.018990575	-0.433782023
3.2.1.99	0.001869546	0.003090036	0.001274386	0.002464707	0.024303877	0.081387551	-0.100738168
2.1.1.74	0.012502179	0.006311454	0.016005002	0.008999356	6.09052E-06	0.000252032	0.669669996
6.3.1.5	0.014850888	0.006570689	0.019231098	0.010470677	4.83137E-07	5.59795E-05	0.722427269
3.1.26.3	0.029315275	0.011518906	0.02519955	0.033431	0.009356868	0.04015367	-0.750976987
2.1.1.191	0.019194317	0.006289299	0.018975976	0.019412657	0.894378126	0.943880803	-0.321754234
2.4.1.80	0.00149018	0.001526559	0.001627013	0.001353348	0.56008305	0.699299095	-0.080438558
2.7.1.201	0.007912593	0.013645384	0.010940828	0.004884358	0.024303877	0.081387551	0.482414242
2.1.1.80	0.002981732	0.003371793	0.00130352	0.004659943	0.003644805	0.020906504	-0.545267043
6.1.1.2	0.03539026	0.006263178	0.036564362	0.034216159	0.141463386	0.276250972	0.095093356
2.7.4.9	0.010162772	0.003097232	0.012110029	0.008215514	1.24541E-05	0.000408399	0.632327399
5.4.99.5	0.008861134	0.003422675	0.007330898	0.010391371	0.002354183	0.015324232	-0.645245332
3.4.11.9	0.06810872	0.023577207	0.056854956	0.079362485	0.001609529	0.011371391	-0.710594876
1.1.1.262	0.006281801	0.003312537	0.004036777	0.008526826	1.2248E-07	2.77265E-05	-0.832175423
1.1.1.69	0.003942569	0.003304872	0.002349427	0.005535711	0.000117169	0.001682975	-0.573491099
6.3.1.10	0.00684926	0.004746235	0.006957664	0.006740855	0.720663933	0.822924974	-0.255319149
1.1.1.371	0.000688781	0.0007782	0.000781663	0.000595898	0.483141694	0.631018822	0.23763506
6.3.3.3	0.00601942	0.003519694	0.006323772	0.005715069	0.705414169	0.811389693	-0.140360399
5.4.2.12	0.045519981	0.026807582	0.037781631	0.05325833	0.089030196	0.195611925	-0.246851932
5.3.1.16	0.002256286	0.002146568	0.002697427	0.001815144	0.519439475	0.663813094	0.264654798
3.5.4.16	0.020591712	0.004732263	0.020721311	0.020462113	0.91055459	0.956219866	-0.25749023
3.6.3.25	0.005346834	0.003951152	0.004147988	0.006545681	0.011299384	0.045991404	-0.318931828
2.7.1.211	0.019334378	0.01689773	0.025115066	0.013553691	0.001271988	0.009488051	0.624185845
4.1.2.13	0.164878024	0.098260142	0.119035179	0.210720869	0.000557234	0.005178994	-0.714937039
3.2.1.10	0.006319197	0.008850534	0.008009835	0.004628559	0.048279687	0.129691029	0.526704299
5.1.99.6	0.0002399	0.001096659	0.000376279	0.000103521	0.273401406	0.444917269	0.175209917
2.3.1.181	0.004671095	0.003650451	0.00450384	0.004838349	0.705414169	0.811389693	0.15990013
1.14.13.172	5.04167E-07	3.45639E-06	1.00833E-06	0	0.3378947	0.489384158	0.131614803
2.6.1.66	0.001522799	0.004370475	0.002232902	0.000812696	0.049961179	0.133588508	0.444913562
4.2.1.135	0.000592991	0.000607382	0.000561986	0.000623996	0.570582118	0.706974319	-0.143113095
3.4.15.5	0.012623079	0.009021943	0.006966587	0.018279572	3.21683E-08	2.21769E-05	-0.846396005
3.5.4.3	0.003438213	0.002079882	0.003095507	0.00378092	0.320383949	0.489384158	-0.429548415

EC	Mean abundance	Std_deviation	Mean abundance - health	Mean abundance - peri-implantitis	P value	FDR corrected P value	LDA correlation (CAP)
1.4.1.2	0.009280252	0.017292205	0.013783415	0.004777089	0.017242287	0.063355381	0.512483717
2.1.1.63	0.008212481	0.003651984	0.009382325	0.007042638	0.004823307	0.025635801	0.415110725
3.6.3.28	0.00328021	0.001704313	0.003311078	0.003249342	0.79844653	0.883885395	-0.101280938
2.7.1.191	0.050538536	0.05996339	0.060260911	0.040816161	0.045920684	0.125487655	0.558727746
2.6.1.104	6.11452E-05	0.000216804	9.356E-05	2.87304E-05	0.418573654	0.565922893	0.13803011
2.4.2.7	0.023554136	0.006997884	0.025424004	0.021684269	0.048279687	0.129691029	0.406643508
4.1.1.38	0.000942379	0.001380576	0.001241594	0.000643164	0.008556308	0.037552684	0.341005322
3.5.99.2	0.00577286	0.007521196	0.009069229	0.002476491	0.002729685	0.017004276	0.50694746
3.4.21.96	0.030622271	0.022239574	0.032919082	0.028325461	0.165425496	0.302323356	0.100521059
1.1.1.38	0.017175073	0.016753992	0.02168161	0.012668537	0.030266661	0.09546907	0.524316109
2.1.1.171	0.008561272	0.002167222	0.009310004	0.00781254	0.014428574	0.05535731	0.317629179
2.7.1.6	0.028876651	0.012839115	0.022995399	0.034757903	0.000660648	0.005888234	-0.580330004
3.4.13.9	0.011911209	0.00785524	0.016593883	0.007228535	2.86704E-06	0.000155716	0.67433782
2.1.3.2	0.025292968	0.008128089	0.024739533	0.025846402	0.255039588	0.418959172	-0.329353018
1.7.1.13	0.005014948	0.002278069	0.004848371	0.005181526	0.959276437	0.987691024	-0.130916196
2.5.1.18	0.001048117	0.002081674	0.001346797	0.000749438	0.064941696	0.158457409	0.566713715
3.1.3.23	0.000328015	0.000350028	0.000315292	0.000340739	0.606111969	0.731543474	0.360605909
1.1.5.4	0.004108646	0.007792861	0.005979784	0.002237507	0.006762554	0.031938366	0.509335649
1.1.5.12	0.002695661	0.003233779	0.002710449	0.002680873	0.407115953	0.554954923	0.503690838
1.7.5.1	0.048672896	0.038732715	0.062564792	0.034781	0.006762554	0.031938366	0.643182805
4.1.99.19	0.06944767	0.091109475	0.100894846	0.038000494	0.178473761	0.321104965	0.264112028
6.3.5.4	0.008539623	0.013557468	0.008163135	0.008916111	0.125281744	0.252891604	-0.243378202
4.1.1.32	0.009734218	0.009706476	0.010305424	0.009163013	0.959276437	0.987691024	0.048089449
1.3.5.2	0.001038043	0.00138763	0.001378002	0.000698084	0.34086786	0.490420812	0.135475467
4.2.3.4	0.016055084	0.005536098	0.01729459	0.014815577	0.093046618	0.202650404	0.07218845
2.6.1.85	0.003617718	0.003064261	0.003222908	0.004012527	0.101506067	0.216463245	-0.486539297
6.5.1.3	0.000862768	0.001143733	0.000618527	0.00110701	0.613392853	0.737804146	0.12702204
2.1.1.223	0.008428847	0.004041297	0.0109586	0.005899094	2.51875E-06	0.000141213	0.551780287
2.7.7.4	0.002555476	0.002629234	0.002928148	0.002182804	0.705414169	0.811389693	0.386018237
6.3.3.2	0.012262254	0.007317492	0.014326571	0.010197936	0.101506067	0.216463245	0.379287885
1.6.5.5	0.001923534	0.002687577	0.00254585	0.001301217	0.014428574	0.05535731	0.40295267
2.7.4.16	0.013082252	0.005424812	0.011350439	0.014814065	0.022979765	0.078774815	-0.314915328
3.6.3.35	0.039260127	0.033845297	0.052394238	0.026126016	0.012781229	0.050257413	0.50640469
4.1.1.3	0.063856269	0.025616468	0.054498915	0.073213622	0.005530342	0.027941089	-0.665219279
3.2.1.135	0.005614875	0.005569348	0.002983376	0.008246373	0.000359831	0.003635969	-0.726009553
3.1.3.100	0.021784249	0.005590947	0.022057841	0.021510657	0.675265662	0.786603029	-0.180199739
3.2.1.14	0.004158112	0.005556695	0.003684292	0.004631932	0.705414169	0.811389693	-0.104429006
2.4.1.64	0.000521394	0.001199225	0.000902146	0.000140642	0.000635953	0.005817295	0.55034931
3.2.1.41	0.028102709	0.014800598	0.034606401	0.021599017	0.002025608	0.013698469	0.560898828
1.8.1.7	0.041571159	0.058743173	0.055445263	0.027697056	1.75724E-05	0.000484774	0.739144594
2.1.1.34	0.000631561	0.000575895	0.000703766	0.000559357	0.141463386	0.276250972	0.546352584
2.7.7.85	0.018505129	0.004429463	0.020988192	0.016022066	3.41151E-05	0.000796167	0.45212766
1.5.1.36	0.009590378	0.011502963	0.012971176	0.006209579	0.009356868	0.04015367	0.618106817
2.1.1.193	0.011717457	0.002497268	0.011733877	0.011701037	0.690279643	0.800337572	-0.077507599
2.5.1.3	0.012951933	0.007401994	0.013992008	0.011911859	0.767049595	0.857319741	-0.155775076
2.3.1.29	0.041618579	0.032361791	0.02773282	0.055504337	6.41681E-05	0.001115242	-0.744138081
1.5.1.34	0.000775521	0.001175244	0.000703877	0.000847166	0.79658161	0.883885395	0.358375857
2.7.2.2	0.039091823	0.055340584	0.045142018	0.033041628	0.255039588	0.418959172	0.230894485
2.5.1.48	0.011296449	0.011611971	0.018136121	0.004456777	1.4826E-06	9.54357E-05	0.739253148
2.4.2.21	0.013154447	0.007005557	0.012150821	0.014158073	0.373099055	0.52125897	-0.387103778
3.5.1.25	0.02513835	0.024530531	0.030534101	0.019742599	0.000848669	0.006860405	0.643942683
3.4.22.10	0.003302608	0.006080907	0.002265915	0.004339301	0.598986716	0.731368732	-0.424383891
1.1.98.6	0.130523482	0.051413186	0.117201221	0.143845742	0.06461309	0.158457409	-0.685735997
3.6.3.41	0.000846628	0.001151158	0.001024426	0.00066883	1	1	0.149342913
3.1.11.5	0.024048519	0.013175122	0.028922195	0.019174843	0.007712559	0.034998504	0.513352149
4.2.1.10	0.012076803	0.003915375	0.014060391	0.010093215	0.000274495	0.00305816	0.498697351
4.2.1.126	0.004138249	0.002653219	0.003506835	0.004769664	0.045920684	0.125487655	-0.244029527

EC	Mean abundance	Std_deviation	Mean abundance - health	Mean abundance - peri-implantitis	P value	FDR corrected P value	LDA correlation (CAP)
2.7.7.77	0.006619699	0.004550934	0.007107383	0.006132016	0.720663933	0.822924974	-0.091293964
3.2.1.45	0.000916624	0.001596604	0.000349569	0.00148368	9.85184E-06	0.000349439	-0.690295267
1.4.3.19	0.000649316	0.001227696	0.001045017	0.000253615	0.006692326	0.031938366	0.478666814
4.3.3.6	0.032149396	0.019835572	0.034356851	0.02994194	0.991851953	1	-0.156643508
1.8.4.14	0.004815816	0.002264589	0.005801401	0.003830232	0.00148899	0.010693652	0.502822406
2.3.2.10	0.001721042	0.003011676	0.002913216	0.000528869	9.31217E-05	0.001505766	0.457062211
2.7.4.3	0.059938989	0.016408888	0.065030678	0.0548473	0.015318868	0.057878678	0.335214937
6.2.1.30	0.004428639	0.006777623	0.002146187	0.006711091	0.000129181	0.001781874	-0.520299609
2.7.7.12	0.015761139	0.011741091	0.018867215	0.012655062	0.074315735	0.175014563	0.339122883
4.3.99.3	0.004366913	0.002147458	0.00461946	0.004114366	0.418845832	0.565922893	0.117021277
3.1.3.41	0.006203742	0.003290004	0.008541391	0.003866093	7.52424E-08	2.61542E-05	0.770408163
2.3.1.129	0.034026435	0.01219649	0.028667601	0.039385269	0.000511256	0.00485553	-0.684107686
4.1.1.33	0.006960638	0.004583638	0.009951545	0.003969731	7.4311E-07	6.7975E-05	0.830330004
3.6.1.1	0.107572662	0.027470731	0.101996697	0.113148627	0.351411522	0.499798057	-0.373860182
6.1.3.1	0.001140436	0.002264366	0.001658913	0.000621959	0.33739017	0.489384158	0.163679833
4.2.1.3	0.011910704	0.011027983	0.010745863	0.013075546	0.493199174	0.639686689	-0.046026921
3.5.99.6	0.039205741	0.014015676	0.033289945	0.045121538	0.002354183	0.015324232	-0.391771602
4.1.1.96	0.006669704	0.003516748	0.005121169	0.008218239	0.000329015	0.00344475	-0.551454624
3.1.4.55	0.001791458	0.001662173	0.001136493	0.002446422	0.003912486	0.02200615	-0.498263135
2.1.1.187	0.002331989	0.002482024	0.003476563	0.001187415	4.69671E-05	0.000927601	0.650998697
1.17.1.9	0.01767682	0.017988133	0.021145238	0.014208402	0.185272618	0.32924725	0.436170213
4.4.1.5	0.016049151	0.006145047	0.017406166	0.014692135	0.110554262	0.229561897	0.430416848
1.2.1.11	0.039766365	0.011224063	0.043759858	0.035772872	0.010616164	0.043930696	0.553625706
5.4.99.22	0.042998107	0.020420658	0.035361517	0.050634696	0.000718634	0.006152642	-0.824142423
2.7.7.60	0.00938371	0.003425363	0.008490787	0.010276633	0.081408821	0.184237696	-0.603994789
4.3.1.17	0.030097308	0.00740491	0.029786683	0.030407934	0.602179894	0.731368732	0.001736865
3.2.1.122	0.081480333	0.24546572	0.104630915	0.058329751	0.089030196	0.195611925	0.215262701
2.1.1.342	0.019441064	0.014381998	0.016464295	0.022417834	0.018279409	0.066186695	-0.354754668
3.4.19.3	0.005262054	0.002812487	0.005660739	0.00486337	0.00148899	0.010693652	0.509009987
2.3.3.10	0.011839847	0.008096362	0.017043693	0.006636002	1.69573E-06	0.000105256	0.849218411
3.6.1.41	0.000667326	0.001129343	0.000950072	0.00038458	0.008545536	0.037552684	0.457625295
5.3.1.4	0.001282096	0.002260265	0.000990732	0.00157346	0.532819632	0.676479136	-0.133738602
6.2.1.16	1.82917E-06	7.47822E-06	2.98333E-06	0.000000675	0.53905226	0.68086688	0.112534806
3.2.1.24	0.001096398	0.001582164	0.001421047	0.00077175	0.23575573	0.397809183	0.152142644
2.7.7.92	7.04891E-05	0.000161352	6.97209E-05	7.12574E-05	0.775970853	0.864511117	-0.010986535
3.2.1.70	0.010674013	0.011725459	0.015860866	0.00548716	0.000468756	0.004501094	0.679656969
3.5.1.97	3.62875E-06	1.20352E-05	3.5125E-06	0.000003745	0.246560502	0.409285723	-0.226860995
1.2.7.8	0.0111377	0.015803076	0.007411628	0.014863771	0.006327364	0.030632198	-0.661528441
1.1.1.40	0.003985842	0.006086973	0.004478094	0.003493591	0.085151945	0.189979564	0.512266609
2.7.8.8	0.011558144	0.003982381	0.010405057	0.012711231	0.048279687	0.129691029	-0.599001303
1.16.3.1	0.016479801	0.027789641	0.019790135	0.013169467	0.001738778	0.012087982	0.601172384
3.2.1.25	0.002093408	0.004918257	0.000976697	0.003210119	0.039407961	0.114533506	-0.434867564
5.1.3.14	0.019545431	0.007868973	0.016114695	0.022976167	0.001174568	0.008953503	-0.4532132
1.3.5.4	0.012678121	0.010773286	0.014739627	0.010616615	0.79844653	0.883885395	0.164133739
2.5.1.78	0.014824646	0.006127074	0.012657038	0.016992255	0.008777645	0.038138866	-0.52887538
4.99.1.12	0.003278926	0.00735075	0.004382471	0.002175381	0.943010334	0.976729416	-0.200825011
2.3.1.57	0.007371719	0.003393011	0.008045257	0.006698182	0.027147143	0.088025623	0.243486756
2.1.1.10	0.002933229	0.003492605	0.004440842	0.001425616	0.000718634	0.006152642	0.56578376
4.1.1.98	0.001176854	0.001018508	0.000859192	0.001494516	0.045920684	0.125487655	-0.089557099
3.6.3.32	0.002311021	0.004376026	0.004038325	0.000583718	6.41681E-05	0.001115242	0.547980894
3.6.1.54	0.0072615	0.004639529	0.004934694	0.009588306	0.000156664	0.002031961	-0.820343031
2.4.1.250	0.000407932	0.000604525	0.000557895	0.000257968	0.020725824	0.072187338	0.248775919
1.15.1.2	0.015587794	0.012785573	0.017079788	0.0140958	0.878244653	0.93357138	-0.183456361
2.4.1.230	6.40922E-05	0.000173404	0.000033325	9.48595E-05	0.882491322	0.936939473	-0.282175932
2.7.7.19	0.00172994	0.002070331	0.001460219	0.001999661	0.675265662	0.786603029	0.099218411
3.4.13.22	0.002492778	0.001812148	0.001642729	0.003342827	9.61681E-05	0.001505766	-0.650347373
5.1.2.1	0.003737545	0.007057183	0.004737499	0.002737591	0.384241116	0.530429754	-0.006187581

EC	Mean abundance	Std_deviation	Mean abundance - health	Mean abundance - peri-implantitis	P value	FDR corrected P value	LDA correlation (CAP)
2.3.1.193	0.00073059	0.00174883	0.001235668	0.000225512	0.024469267	0.081783818	0.481351914
1.5.1.20	0.017162672	0.019000945	0.022501138	0.011824207	0.039407961	0.114533506	0.59465914
4.2.1.113	0.002816802	0.003770865	0.00422109	0.001412513	0.002936794	0.017909288	0.650673035
3.1.21.4	0.007198401	0.004531546	0.007883638	0.006513164	0.159171691	0.299914766	0.048632219
4.1.1.37	0.00574834	0.006550738	0.007612007	0.003884674	0.06461309	0.158457409	0.567629179
5.1.3.22	0.005982938	0.005476116	0.007271078	0.004694798	0.033682558	0.102522391	0.650564481
2.4.1.281	0.000410959	0.000733926	0.000232788	0.00058913	0.063371383	0.158019317	-0.471560324
1.5.1.2	0.008301145	0.00411531	0.010169592	0.006432698	0.002184365	0.014545696	0.611702128
3.5.1.44	0.000866733	0.000972656	0.000454541	0.001278925	0.003522871	0.020561799	-0.349690589
2.7.8.12	0.005631461	0.004687799	0.005933929	0.005328994	0.587992654	0.721702848	0.113981763
4.1.99.22	0.010223858	0.00614354	0.012196722	0.008250994	0.005166141	0.026722477	0.523230569
2.7.7.13	0.023172203	0.013612406	0.016144636	0.03019977	4.22558E-05	0.000927601	-0.716131133
2.7.1.53	0.000822569	0.001727683	0.001062103	0.000583035	0.844348139	0.915107845	0.002665658
3.1.1.3	0.007184499	0.009648574	0.008622633	0.005746364	0.846132513	0.915107845	-0.431936604
3.6.3.40	0.006560762	0.005516483	0.00954332	0.003578205	5.78771E-05	0.001058846	0.651432914
5.3.1.22	0.000421745	0.000597236	0.000194501	0.000648989	0.012564885	0.050201772	-0.489714084
2.1.1.79	0.002222342	0.003635383	0.00225944	0.002185245	0.61651648	0.739990085	0.004776379
3.1.1.53	0.008843467	0.009256367	0.004891969	0.012794965	0.000781202	0.006496311	-0.707772471
1.3.1.14	0.006431932	0.00352672	0.005573424	0.007290439	0.206792441	0.359046216	-0.27540165
1.1.1.37	0.064960447	0.038073342	0.042061864	0.087859031	6.09052E-06	0.000252032	-0.741641337
2.7.1.59	0.000641844	0.001871129	0.001033612	0.000250075	0.097782656	0.211639174	0.259195008
2.3.1.30	0.01693258	0.012242428	0.019735257	0.014129903	0.053297186	0.140349256	0.493161094
3.6.3.30	0.028205943	0.013659262	0.024400898	0.032010988	0.033682558	0.102522391	-0.581089883
1.4.3.5	0.00214472	0.001746981	0.001770156	0.002519283	0.157807343	0.299420483	-0.231606372
5.4.2.6	0.009213821	0.034867503	0.011809277	0.006618365	0.025692206	0.085215753	0.514654798
2.1.1.113	0.002163713	0.002028136	0.001882194	0.002445231	0.893377762	0.943880803	0.136673271
4.1.2.17	0.004652827	0.004256232	0.004406297	0.004899357	0.602179894	0.731368732	-0.051997395
4.2.1.1	0.011825713	0.006427721	0.013590384	0.010061042	0.012781229	0.050257413	0.402192792
2.6.1.62	0.006916435	0.003954999	0.007654792	0.006178078	0.44288188	0.590282751	-0.036257056
3.4.21.50	0.026932961	0.03422115	0.012729453	0.041136469	0.019369559	0.06912586	-0.538319583
6.6.1.1	0.003560739	0.003271314	0.003797363	0.003324116	0.230034676	0.388910766	0.204624403
1.1.1.380	0.00017236	0.000416523	0.0002676	7.71195E-05	0.017494199	0.064145396	0.450566578
3.2.1.93	0.007458978	0.012238118	0.010648051	0.004269905	0.027147143	0.088025623	0.453321754
3.1.6.1	0.000618582	0.000999854	0.000477719	0.000759446	0.860854853	0.924389386	0.01150798
1.1.1.28	0.044401305	0.030750069	0.036801116	0.052001494	0.002535725	0.016084268	-0.623100304
2.3.1.47	0.003815429	0.002302074	0.003727317	0.00390354	0.48034525	0.628644612	-0.462657403
4.1.3.38	0.002168053	0.002163072	0.002025754	0.002310351	0.630998004	0.750119378	-0.120603561
2.7.1.170	0.000830064	0.000808841	0.00100301	0.000657118	0.369691028	0.518636598	0.323200523
2.7.1.207	0.012905608	0.020836249	0.013544224	0.012266992	0.178473761	0.321104965	0.491749891
1.11.1.21	0.000141446	0.000561007	0.000231359	5.15325E-05	0.001775691	0.01229542	0.541288462
2.1.1.265	0.006478366	0.006056472	0.009400684	0.003556048	7.8682E-05	0.001314898	0.753148068
3.6.3.12	0.001376142	0.003225495	0.002108217	0.000644067	0.00137664	0.01009536	0.4959835
3.4.11.21	0.010669007	0.004873656	0.008865962	0.012472052	0.001271988	0.009488051	-0.637646548
1.1.1.14	0.008171167	0.017594279	0.010117827	0.006224507	0.165425496	0.302323356	0.383521494
3.6.1.22	0.002739615	0.001982029	0.00253177	0.00294746	0.630998004	0.750119378	-0.290707772
2.1.1.199	0.045149971	0.021063333	0.047400666	0.042899275	0.165425496	0.302323356	0.332283977
1.1.1.86	0.051281351	0.037316614	0.063898954	0.038663748	0.004823307	0.025635801	0.550694746
5.4.2.7	0.014749664	0.008768921	0.018942055	0.010557273	0.000660648	0.005888234	0.71309162
2.1.1.186	0.001029069	0.001900344	0.001303665	0.000754474	0.893323067	0.943880803	0.083306192
2.7.7.53	0.000552191	0.000480504	0.000707069	0.000397313	0.015318868	0.057878678	0.39090317
4.2.1.17	0.056790053	0.043054722	0.044605333	0.068974773	0.024303877	0.081387551	-0.589882762
3.1.12.1	0.00249877	0.002486908	0.002047153	0.002950386	0.320383949	0.489384158	-0.246960486
1.3.8.1	0.212744535	0.150199395	0.155353441	0.270135629	0.008230024	0.036374662	-0.675206253
4.1.1.15	0.014176232	0.018813978	0.009213497	0.019138968	0.506231587	0.649800959	-0.258901433
3.1.21.5	0.00968086	0.008014448	0.011779907	0.007581812	0.061616509	0.154307626	0.186061659
3.6.3.29	0.003893666	0.002253156	0.004293547	0.003493785	0.310444458	0.489384158	0.199413808
1.1.1.42	0.017150201	0.024148546	0.012045915	0.022254486	0.720663933	0.822924974	-0.00749023

EC	Mean abundance	Std_deviation	Mean abundance - health	Mean abundance - peri-implantitis	P value	FDR corrected P value	LDA correlation (CAP)
2.3.3.1	0.010075615	0.009933989	0.009630052	0.010521179	0.79844653	0.883885395	0.210594876
2.4.1.4	0.001046305	0.001124912	0.000865792	0.001226818	0.751487275	0.845559951	0.263677812
1.1.1.17	0.004984524	0.009908546	0.007281633	0.002687414	0.00591703	0.029309765	0.56871472
1.3.1.12	0.009522607	0.005569188	0.012413798	0.006631416	0.000208058	0.002493826	0.554168476
2.7.4.8	0.036013317	0.013790897	0.043079194	0.02894744	0.000156664	0.002031961	0.624728615
3.1.1.41	0.001955893	0.001836462	0.002562223	0.001349563	0.07095965	0.169174036	0.628745115
2.7.1.208	0.139845917	0.12952497	0.198069069	0.081622765	0.000156664	0.002031961	0.76443769
4.2.2.3	7.93209E-05	0.000146196	0.000112305	4.63365E-05	0.52771354	0.672902518	0.215703959
3.5.5.1	0.000294307	0.000280617	0.000283933	0.000304682	0.463927331	0.614562272	0.145107005
2.6.1.52	0.052255137	0.038610714	0.030930688	0.073579585	7.76212E-06	0.00029979	-0.858988276
3.1.1.85	0.002103621	0.001825795	0.00180569	0.002401552	0.320383949	0.489384158	-0.371037777
3.4.24.13	0.015010586	0.018093811	0.025034441	0.004986732	2.45847E-05	0.000647397	0.614633087
3.1.1.22	6.4375E-07	4.41333E-06	1.2875E-06	0	0.3378947	0.489384158	0.194789908
1.2.4.4	0.001284794	0.002897171	0.00174855	0.000821038	0.244001999	0.406592018	0.300757185
2.4.1.288	0.005800167	0.007920781	0.007268424	0.004331911	0.050737544	0.134834635	0.380916196
2.4.2.46	8.08746E-05	0.000240259	0.000110583	5.11661E-05	0.660561442	0.775267373	0.155358552
2.5.1.30	0.009291168	0.006315765	0.006888278	0.011694057	0.007712559	0.034998504	-0.621363439
2.4.1.342	0.000922727	0.001390983	0.000839252	0.001006202	0.105514701	0.221867402	0.308898961
2.1.1.207	0.007043912	0.002803862	0.007941529	0.006146294	0.015318868	0.057878678	0.54385584
2.4.1.348	0.0004594	0.00043338	0.000299221	0.000619579	0.008023848	0.036221943	-0.018464215
2.5.1.16	0.005989648	0.002844923	0.006470449	0.005508847	0.230034676	0.388910766	0.437147199
3.4.25.2	0.004808451	0.010007662	0.006023272	0.003593631	0.30070683	0.47991595	-0.008575771
2.6.1.98	0.000205934	0.000370857	0.000155897	0.000255971	0.715512904	0.821374786	0.110410056
3.5.1.104	0.017859841	0.009129308	0.021731609	0.013988072	0.002729685	0.017004276	0.548415111
4.2.1.19	0.001941987	0.002127081	0.002129137	0.001754836	0.230034676	0.388910766	0.377333912
1.17.1.1	0.001852956	0.002027174	0.001563376	0.002142536	0.281836841	0.455448476	0.077724707
2.1.1.189	0.000797707	0.001216689	0.00122599	0.000369423	0.00252057	0.016084268	0.614106983
4.1.1.81	0.008217203	0.008656637	0.008456465	0.007977941	0.48034525	0.628644612	-0.27138515
3.2.1.80	0.009421108	0.010378834	0.011377855	0.007464361	0.630998004	0.750119378	0.000868432
1.7.2.4	0.023967927	0.022720058	0.021844921	0.026090933	0.56008305	0.699299095	0.285062961
3.2.2.20	0.00456882	0.002624927	0.005747184	0.003390455	0.000848669	0.006860405	0.726009553
1.1.1.26	0.004484177	0.003280936	0.00448759	0.004480764	0.91055459	0.956219866	-0.164242293
3.5.1.47	0.005078043	0.003898072	0.007672445	0.00248364	1.04306E-07	2.77265E-05	0.779526704
2.7.1.36	0.00526022	0.003402008	0.007223426	0.003297013	6.87931E-06	0.000271733	0.843465046
4.1.1.11	0.004378166	0.002014552	0.003669568	0.005086765	0.004823307	0.025635801	-0.469821971
2.4.99.16	0.001622793	0.001973051	0.00147122	0.001774366	0.407115953	0.554954923	0.312852801
3.2.1.177	0.001740908	0.002529734	0.001371579	0.002110237	0.675265662	0.786603029	0.012266609
4.1.1.48	0.005775022	0.00513118	0.00747606	0.004073984	0.067726454	0.164397453	0.457121146
2.7.8.20	0.015114713	0.020142899	0.022961066	0.007268361	2.51875E-06	0.000141213	0.646547981
2.7.4.2	0.00867858	0.006645249	0.012742908	0.004614252	8.55438E-07	7.07977E-05	0.809270517
3.1.21.2	0.003253535	0.001395711	0.003235356	0.003271715	0.6603774	0.775267373	0.018671298
4.2.1.9	0.024756312	0.015116871	0.03314104	0.016371584	6.41681E-05	0.001115242	0.625597047
2.4.1.109	0.001505222	0.001552111	0.001871125	0.00113932	0.0477552	0.129482899	0.530192417
3.1.2.12	0.002056566	0.002957108	0.002082771	0.002030361	0.467672658	0.616703398	0.041576205
5.3.1.28	0.010275106	0.004926005	0.008731686	0.011818526	0.007712559	0.034998504	-0.560138949
5.3.4.1	0.004758032	0.005323649	0.00585077	0.003665295	0.222093193	0.379172858	0.542010421
6.2.1.26	0.003753505	0.003050674	0.003507555	0.003999455	0.532819632	0.676479136	0.022796353
2.7.1.177	0.002531553	0.003672266	0.003415538	0.001647569	0.141463386	0.276250972	0.331415545
2.5.1.90	0.01253357	0.00608478	0.008782545	0.016284596	9.83425E-07	7.12164E-05	-0.803951368
1.14.19.3	6.6676E-05	0.000161357	0.000119937	1.34145E-05	0.003200791	0.019182672	0.190650716
3.1.3.1	0.016762692	0.021771004	0.01463872	0.018886664	0.037415704	0.110031291	-0.484693878
2.7.1.200	0.003611877	0.005076077	0.004108309	0.003115444	0.153095027	0.293361805	0.517042987
3.6.3.44	0.001584635	0.003845667	0.001978973	0.001190297	0.077823119	0.181066372	0.255279883
4.6.1.17	0.003973731	0.002894883	0.004263932	0.003683531	0.310444458	0.489384158	0.389926183
2.7.1.1	0.001431982	0.002149679	0.000473794	0.00239017	0.002984688	0.018137719	-0.615843895
4.3.1.14	0.007736707	0.006961138	0.004287465	0.01118595	0.000250456	0.002863767	-0.852583587
4.2.1.167	0.051873081	0.029077178	0.038804737	0.064941425	0.005530342	0.027941089	-0.711897525

EC	Mean abundance	Std_deviation	Mean abundance - health	Mean abundance - peri-implantitis	P value	FDR corrected P value	LDA correlation (CAP)
2.7.1.167	0.000292521	0.000510005	0.000336534	0.000248508	0.654320761	0.77042391	0.302613461
2.5.1.72	0.005938855	0.003979382	0.004264154	0.007613556	0.002025608	0.013698469	-0.776378637
3.2.2.10	0.007814954	0.002907932	0.007166762	0.008463147	0.125281744	0.252891604	-0.27941815
4.3.1.12	0.001481791	0.002498276	0.001886809	0.001076773	0.120214723	0.24493926	0.200825011
1.5.1.7	0.020465768	0.012513075	0.012880717	0.028050819	3.70198E-06	0.00018383	-0.902627008
2.3.1.183	0.002637066	0.002527578	0.003302424	0.001971708	0.125281744	0.252891604	0.508033
2.1.1.228	0.038884703	0.023882663	0.042204693	0.035564713	0.395580536	0.543063958	0.217542336
2.3.1.117	0.003954492	0.004385382	0.004548236	0.003360749	0.362155525	0.511996853	0.479374729
1.8.4.11	0.001432801	0.002440578	0.001925693	0.00093991	0.720663933	0.822924974	0.212440295
4.2.1.59	0.023784483	0.010664617	0.025852862	0.021716104	0.192257401	0.338887791	0.267585758
5.1.3.20	0.010918855	0.004875504	0.010120217	0.011717493	0.185272618	0.32924725	-0.479049066
3.1.3.10	0.005135096	0.003705393	0.00528678	0.004983411	0.878244653	0.93357138	-0.08423795
1.5.5.1	0.000721894	0.000860174	0.000644633	0.000799155	1	1	0.309517358
3.4.11.7	0.01014903	0.009523169	0.014977282	0.005320779	1.75724E-05	0.000484774	0.712765957
2.3.1.46	0.018557859	0.015588687	0.025006598	0.012109119	0.000274495	0.00305816	0.676183239
2.4.2.2	0.016138593	0.01113966	0.0214736	0.010803587	0.000557234	0.005178994	0.67433782
2.8.1.1	0.000109454	0.000206563	0.000164215	5.46918E-05	0.047201628	0.12832471	0.406996253
1.2.1.70	0.010010783	0.006248422	0.011640271	0.008381296	0.089030196	0.195611925	0.454950065
4.2.99.20	0.001861827	0.001838414	0.002681663	0.001041991	0.006097564	0.030021434	0.506581269
1.1.1.133	0.022885862	0.005962151	0.023760373	0.02201135	0.105955089	0.221867402	0.092379505
1.3.99.23	0.000465239	0.001095656	0.000599672	0.000330807	0.572934393	0.706974319	0.227589024
3.1.11.1	0.000591146	0.001053891	0.000863727	0.000318565	0.044329477	0.123763702	0.406536743
3.5.4.2	0.002936493	0.002630408	0.002627489	0.003245497	0.222093193	0.379172858	-0.235345202
5.5.1.4	0.005617504	0.005021711	0.003170388	0.00806462	0.000836354	0.006860405	-0.576329145
4.3.1.4	0.007627051	0.003667472	0.007418425	0.007835677	0.630998004	0.750119378	-0.052323057
2.7.7.1	0.00024611	0.000795624	0.000147317	0.000344904	0.367538241	0.517231954	-0.041699408
1.11.1.19	0.000747678	0.001065151	0.001035263	0.000460092	0.039900204	0.115577592	0.351924739
4.1.1.50	0.003865597	0.002499689	0.003787093	0.003944101	0.720663933	0.822924974	-0.098024316
2.1.1.172	0.007700897	0.004655475	0.010954206	0.004447587	7.52424E-08	2.61542E-05	0.793095962
3.4.19.1	0.00683378	0.004010852	0.005978228	0.007689332	0.230034676	0.388910766	-0.543530178
6.1.1.18	0.031938128	0.014007584	0.025617748	0.038258509	0.000781202	0.006496311	-0.72166739
1.1.3.15	0.054232072	0.04563547	0.036190736	0.072273408	0.000848669	0.006860405	-0.773230569
1.8.5.7	0.001657788	0.001675708	0.002103895	0.001211682	0.004288369	0.023660906	0.617413965
2.4.1.5	0.029170058	0.041429309	0.047282393	0.011057722	5.78771E-05	0.001058846	0.730894485
3.1.1.75	4.52515E-05	0.000157662	4.10486E-05	4.94544E-05	0.607406489	0.73259714	0.128445082
4.3.1.3	0.037716448	0.020095878	0.027326535	0.048106362	0.000117169	0.001682975	-0.719170647
2.6.1.13	0.004910263	0.005664382	0.003026246	0.006794279	0.147193088	0.284246208	-0.382327399
5.3.1.12	0.003708668	0.002650138	0.002713039	0.004704297	0.013583315	0.052578624	-0.598892749
4.1.1.5	0.008519228	0.007922285	0.013434628	0.003603827	2.69768E-08	2.21769E-05	0.838363005
4.2.1.75	0.013546252	0.00670351	0.010462799	0.016629705	0.002729685	0.017004276	-0.761832393
3.5.1.3	0.004195028	0.004439566	0.003941767	0.004448289	0.081408821	0.184237696	-0.447351281
3.2.2.28	4.14322E-05	7.16527E-05	2.09151E-05	6.19493E-05	0.00733952	0.034016231	-0.383493998
2.4.1.153	1.25417E-05	2.35507E-05	1.26125E-05	1.24708E-05	0.634739042	0.754050891	-0.188118411
2.7.8.36	0.000833455	0.001363374	0.000647554	0.001019356	0.627200981	0.750119378	-0.211022202
1.1.1.169	0.010571317	0.006291675	0.009904856	0.011237778	0.130510373	0.26012274	-0.391771602
2.7.7.38	0.008507102	0.004127054	0.00675749	0.010256714	0.002025608	0.013698469	-0.751628311
3.6.3.31	0.009964562	0.00946832	0.011318791	0.008610332	0.943010334	0.976729416	0.060247503
2.8.1.12	0.001822318	0.001822709	0.002502239	0.001142396	0.00137664	0.01009536	0.623968736
2.1.2.11	0.008795126	0.004284928	0.007510887	0.010079365	0.028671131	0.091431973	-0.347155884
1.6.99.1	0.001215702	0.004241237	0.00164758	0.000783823	0.269909419	0.440471897	-0.195418522
1.1.1.290	0.001671388	0.00253311	0.000865151	0.002477625	0.064963533	0.158457409	-0.478899232
3.8.1.2	0.002437741	0.001897762	0.003562072	0.001313409	4.17358E-07	5.1812E-05	0.800586192
4.2.1.49	0.074210052	0.035648153	0.054697878	0.093722227	5.21595E-05	0.001007257	-0.821211463
4.3.1.1	0.04251955	0.024632409	0.030825882	0.054213218	0.000329015	0.00344475	-0.799283543
5.4.3.2	0.029193059	0.024066843	0.020186947	0.038199171	0.007223852	0.033569663	-0.778115502
3.6.1.3	0.000874923	0.001566257	0.001364706	0.000385141	0.003635619	0.020906504	0.267386947
3.5.2.10	0.000433922	0.000512429	0.000200698	0.000667146	0.001063398	0.008439203	-0.614916998

EC	Mean abundance	Std_deviation	Mean abundance - health	Mean abundance - peri-implantitis	P value	FDR corrected P value	LDA correlation (CAP)
3.1.3.90	0.016580242	0.01399689	0.023376041	0.009784444	0.000129181	0.001781874	0.720039079
2.3.1.81	0.00029389	0.000374592	0.000308468	0.000279311	0.47617898	0.6257154	0.05370739
2.1.3.3	0.073528997	0.082089241	0.081603171	0.065454823	0.206792441	0.359046216	0.257056014
2.4.2.17	0.002603014	0.002424671	0.002803542	0.002402487	0.830166612	0.906745422	-0.005210595
2.7.1.17	0.003277697	0.003364906	0.002163824	0.00439157	0.000106192	0.001604884	-0.623100304
2.1.1.298	0.000872619	0.001386434	0.001200582	0.000544656	0.059173368	0.149751441	0.554988604
3.6.3.27	0.013725165	0.009841408	0.020396488	0.007053842	1.43578E-07	2.77265E-05	0.857902736
3.6.3.10	0.028515365	0.024781228	0.037492177	0.019538553	0.003393537	0.020061115	0.592705167
2.6.1.33	0.002409786	0.00324315	0.001509547	0.003310025	0.017226921	0.063355381	-0.349582024
4.1.99.3	0.000751197	0.00170032	0.001189446	0.000312949	0.032737058	0.101240227	0.523297568
1.1.1.271	0.008373872	0.005027373	0.006039695	0.01070805	0.000359831	0.003635969	-0.819474598
1.8.1.2	0.003380868	0.005146112	0.0046639	0.002097836	0.199430058	0.349052811	0.49359531
2.1.2.10	0.03230619	0.025802161	0.020052693	0.044559688	0.000300628	0.003286107	-0.7540165
3.6.1.9	0.00087148	0.001255559	0.001087573	0.000655386	0.380796111	0.529035685	0.14895234
2.1.1.131	0.007741625	0.007003767	0.008993945	0.006489306	0.630998004	0.750119378	0.047980894
1.21.4.2	0.075149205	0.081479554	0.050316932	0.099981479	0.058733676	0.149456998	-0.375054277
1.1.1.381	0.00093737	0.001418199	0.001146678	0.000728062	0.519439475	0.663813094	0.267694312
1.1.2.3	0.007182695	0.022344004	0.010780607	0.003584783	0.11530651	0.23772564	0.471233174
2.8.3.10	0.029531532	0.01332221	0.025872819	0.033190245	0.081408821	0.184237696	-0.284628745
2.4.1.346	0.008438551	0.00593985	0.004572324	0.012304778	3.70198E-06	0.00018383	-0.898610508
5.4.99.27	0.000888927	0.001689528	0.001311319	0.000466535	0.072819801	0.172897287	0.390262437
3.4.11.23	0.001639451	0.003800848	0.002313716	0.000965186	0.026567331	0.087008193	0.444010058
1.4.5.1	0.003736245	0.002828721	0.00511734	0.002355149	0.000274495	0.00305816	0.761940947
1.5.1.38	0.000884833	0.001897534	0.0015864	0.000183265	5.07379E-05	0.000990814	0.702312457
2.4.1.173	0.00013254	0.000307405	0.000152274	0.000112805	0.844805773	0.915107845	0.038433244
1.14.18.1	4.47849E-05	8.64744E-05	5.68332E-05	3.27367E-05	0.721124704	0.822924974	0.158097636
5.4.99.24	0.004132513	0.002286584	0.00351732	0.004747707	0.06461309	0.158457409	-0.439426835
2.7.1.100	0.001279358	0.001478294	0.00101373	0.001544985	0.430768443	0.577681754	-0.559921841
3.2.1.58	0.00032688	0.000603273	0.000149528	0.000504233	0.193995798	0.340570401	-0.283800598
3.7.1.2	3.3839E-05	0.000101962	6.41864E-05	3.49167E-06	0.040842135	0.116743743	0.232113075
2.4.1.315	0.004876743	0.002885064	0.00526639	0.004487096	0.384241116	0.530429754	0.103560573
3.2.1.97	0.022561659	0.034065655	0.032237784	0.012885533	0.061616509	0.154307626	0.451868538
1.18.6.1	0.001475109	0.001619393	0.000872874	0.002077344	0.005041832	0.026393688	-0.675278856
6.3.2.14	0.001774995	0.002248449	0.002589199	0.000960791	0.002184365	0.014545696	0.471558836
2.1.1.86	1.37083E-07	9.39796E-07	0	2.74167E-07	0.3378947	0.489384158	-0.184260724
1.14.14.28	0.003938796	0.014482994	0.00668064	0.001196952	0.014495012	0.055489716	0.35331818
3.6.1.31	0.000679951	0.000764132	0.000959562	0.00040034	0.010616164	0.043930696	0.445071646
2.7.4.1	0.021362192	0.010906103	0.020815809	0.021908576	0.926767515	0.966239916	0.094333478
2.8.1.10	0.002639275	0.002770078	0.002319098	0.002959451	0.407115953	0.554954923	-0.367998263
1.1.1.205	0.088206852	0.015725693	0.090363913	0.08604979	0.165425496	0.302323356	0.268562744
1.8.5.2	0.001669773	0.001957717	0.002188091	0.001151456	0.005205424	0.026766353	0.559177138
1.14.11.47	0.001296402	0.002523985	0.002003289	0.000589515	0.018279409	0.066186695	0.475032566
3.1.3.2	0.016268625	0.022796552	0.017567116	0.014970134	0.814269221	0.893434284	0.019865393
4.2.1.47	0.012374094	0.007252074	0.008051495	0.016696693	1.56822E-05	0.000461959	-0.886995224
6.3.2.3	0.000557203	0.000888579	0.000529675	0.000584731	0.288013773	0.463918386	0.468013537
1.4.1.4	0.156739484	0.11246892	0.104378064	0.209100905	0.000511256	0.00485553	-0.86550152
2.1.1.107	0.003846625	0.006304045	0.005280215	0.002413034	0.028671131	0.091431973	0.539513678
1.7.1.15	0.002475638	0.003960755	0.003933622	0.001017653	0.000250456	0.002863767	0.513135041
2.7.1.71	0.01390907	0.004351437	0.014339848	0.013478293	0.645619886	0.764364688	0.076530612
2.6.1.34	0.000558976	0.000804582	0.000508896	0.000609056	0.269701316	0.440471897	-0.148256783
4.1.3.3	0.016009587	0.013758549	0.019318719	0.012700454	0.081408821	0.184237696	0.286257056
3.5.4.40	0.001369427	0.002527662	0.001305353	0.001433502	0.269909419	0.440471897	-0.050048855
4.1.1.44	0.003071413	0.002851067	0.003485976	0.002656851	0.630998004	0.750119378	0.22166739
1.21.98.3	0.00039635	0.000813147	0.000308613	0.000484087	0.032286895	0.100026067	-0.576746495
3.1.4.58	0.000283157	0.000567977	0.000487215	7.90995E-05	0.05500858	0.1439833	0.522888104
2.7.7.76	0.001264445	0.001273841	0.001478086	0.001050803	0.206792441	0.359046216	0.372340426
2.1.1.166	0.001972216	0.002161363	0.002127407	0.001817024	0.153095027	0.293361805	0.441597916

EC	Mean abundance	Std_deviation	Mean abundance - health	Mean abundance - peri-implantitis	P value	FDR corrected P value	LDA correlation (CAP)
4.2.1.55	0.000742602	0.000959311	0.000708598	0.000776607	0.432254762	0.579228046	-0.431401409
2.7.1.5	0.003491645	0.006236797	0.003597025	0.003386265	0.630998004	0.750119378	0.140034737
2.7.2.7	0.00601869	0.005526367	0.003673163	0.008364218	0.005530342	0.027941089	-0.69973947
1.5.1.39	0.000715699	0.001225562	0.000626507	0.000804891	0.780406509	0.868339637	0.079256452
3.2.2.4	0.002613265	0.003049361	0.00175802	0.00346851	0.101506067	0.216463245	-0.544507165
1.2.7.4	0.007368235	0.014731358	0.006676455	0.008060016	0.055961569	0.144949638	0.48501954
2.8.3.18	0.022633388	0.022591324	0.022383761	0.022883015	0.782704652	0.87033953	0.040816327
2.5.1.61	0.007650324	0.004848056	0.008791623	0.006509026	0.105955089	0.221867402	0.427920104
5.3.1.30	0.00068011	0.000787416	0.000537453	0.000822766	0.026633118	0.087008193	-0.364346979
2.6.1.17	0.000260975	0.000354403	0.000302563	0.000219387	0.109558256	0.228586132	0.458741676
1.1.1.85	0.008235392	0.00568593	0.010074008	0.006396777	0.028671131	0.091431973	0.487950499
1.7.7.1	0.003966889	0.004688008	0.005677354	0.002256424	0.025692206	0.085215753	0.456904038
3.5.2.2	0.007665792	0.008775549	0.008557077	0.006774507	0.373099055	0.52125897	0.194528875
3.4.14.4	0.014358036	0.010335835	0.008104602	0.020611471	5.38649E-06	0.000246361	-0.845527573
4.3.2.3	5.43006E-05	9.90383E-05	6.26128E-05	4.59884E-05	0.529888244	0.675180182	-0.155317365
2.4.2.19	0.008183283	0.005079486	0.005223637	0.01114293	9.61681E-05	0.001505766	-0.800911854
1.1.1.29	0.00721127	0.007394598	0.007053417	0.007369123	0.07095965	0.169174036	-0.313612679
6.4.1.1	0.049592402	0.02885508	0.044157937	0.055026866	0.028671131	0.091431973	-0.697459835
2.6.99.2	0.003446021	0.002945311	0.002189973	0.004702069	0.013583315	0.052578624	-0.362244898
2.1.1.77	0.000775592	0.000686425	0.000692115	0.000859069	0.672503686	0.786603029	0.27302087
2.7.1.166	0.00036414	0.001270286	0.000581733	0.000146546	0.857716109	0.923612514	0.128696417
2.3.1.1	0.001151416	0.000830979	0.000902616	0.001400215	0.07095965	0.169174036	-0.11550152
1.1.1.77	0.006618299	0.010441539	0.005473785	0.007762814	0.751487275	0.845559951	0.191923578
4.1.99.12	0.000684485	0.001017882	0.000824323	0.000544647	0.804132991	0.888482606	-0.019149218
1.17.1.4	0.00265418	0.002649122	0.001783264	0.003525097	0.008777645	0.038138866	-0.414459401
2.7.7.43	0.003193586	0.005991561	0.003985211	0.002401961	0.243963602	0.406592018	0.305612856
3.2.2.1	0.003151736	0.01205798	0.004856352	0.001447121	0.089030196	0.195611925	0.414242293
2.8.3.16	0.001218097	0.005204561	0.000837317	0.001598876	0.745502422	0.844093296	0.08389447
1.2.4.2	0.014827267	0.01855902	0.016769809	0.012884724	0.185272618	0.32924725	0.538862353
5.4.99.18	0.011350215	0.004396286	0.00949652	0.013203909	0.003912486	0.02200615	-0.551997395
2.3.1.201	0.001044838	0.001512424	0.000514996	0.001574681	0.026608441	0.087008193	-0.539062944
1.2.5.1	0.009102607	0.011041935	0.012367408	0.005837807	0.028671131	0.091431973	0.457446809
1.3.98.3	0.00133141	0.002747011	0.002077897	0.000584924	0.010245682	0.043215876	0.487569214
3.4.14.11	0.012419013	0.010570184	0.017497122	0.007340903	9.61681E-05	0.001505766	0.771710812
2.7.9.3	0.006109028	0.003574194	0.006061932	0.006156124	0.975559196	0.997952845	-0.268128528
3.5.1.5	0.023313602	0.038693096	0.028764382	0.017862822	0.055961569	0.144949638	0.511940947
3.1.1.1	0.002363456	0.002780296	0.002853603	0.001873309	0.814269221	0.893434284	0.179548415
3.5.1.103	0.000550429	0.00081264	0.000762184	0.000338673	0.077451949	0.181066372	0.383996966
3.7.1.5	0.000242135	0.000367384	0.000294096	0.000190174	0.763160626	0.8551729	0.269413636
1.3.1.34	0.000173673	0.000392954	0.000277924	6.94218E-05	0.103394859	0.219600359	0.320645089
3.5.1.9	0.003954955	0.011107062	0.006696501	0.00121341	7.10844E-05	0.001223214	0.697676943
2.7.14.1	0.006866038	0.007393958	0.00649125	0.007240826	0.751487275	0.845559951	0.109422492
3.5.1.24	0.002075836	0.007709244	0.00300622	0.001145452	0.219821642	0.378807165	0.306915646
1.2.1.87	0.008532687	0.034715231	0.013790012	0.003275363	1	1	-0.025727312
4.2.1.115	0.000927973	0.001726015	0.000966252	0.000889695	0.274007131	0.445485868	-0.268166258
6.3.1.12	0.003017917	0.007756919	0.00320693	0.002828904	0.720663933	0.822924974	0.079244464
3.6.1.25	0.000436546	0.000785396	0.000683816	0.000189276	0.011690486	0.047472116	0.497390048
4.2.1.8	0.001487611	0.001299686	0.001705926	0.001269296	0.384241116	0.530429754	0.182045158
2.7.7.61	0.001188806	0.001086141	0.001352712	0.0010249	0.421288251	0.566717478	0.407848672
3.6.1.7	0.001284263	0.000952625	0.001631478	0.000937049	0.004823307	0.025635801	0.57674772
2.4.1.291	0.000721906	0.00126622	0.00040975	0.001034061	0.204070294	0.356098565	-0.263427137
3.4.24.55	0.000168353	0.000522936	0.000251787	8.49193E-05	0.916008331	0.959049686	0.24257576
2.7.7.75	0.001830114	0.001493221	0.00220258	0.001457648	0.147193088	0.284246208	0.272253582
4.1.3.34	0.014805964	0.006804846	0.012890914	0.016721015	0.058733676	0.149456998	-0.170646982
1.1.1.157	0.072732645	0.052848775	0.059467096	0.085998194	0.031936275	0.099293822	-0.568280504
2.4.1.83	0.009411553	0.005169003	0.007965804	0.010857301	0.002936794	0.017909288	-0.615067304
2.7.1.92	0.0004848	0.000873799	0.00055501	0.000414591	0.357271103	0.507301616	0.430627435

EC	Mean abundance	Std_deviation	Mean abundance - health	Mean abundance - peri-implantitis	P value	FDR corrected P value	LDA correlation (CAP)
2.3.1.189	0.000325856	0.000483629	0.000449143	0.000202569	0.02615741	0.086192466	0.268897101
2.3.1.182	0.000658607	0.001628556	0.000747559	0.000569656	0.114505827	0.236355258	-0.526338733
1.2.1.60	0.000100903	0.000299803	0.000168474	3.33308E-05	0.03968246	0.115138756	0.335240217
1.3.1.28	0.000247836	0.000399944	0.00034388	0.000151793	0.08203383	0.185402857	0.334958015
3.5.1.108	0.012409585	0.00748044	0.012552098	0.012267072	0.56008305	0.699299095	-0.207338254
2.4.99.19	0.000195523	0.000249559	0.000157626	0.000233421	0.48776223	0.635480327	-0.105663092
3.5.1.119	0.001066764	0.001211991	0.001401564	0.000731964	0.064963533	0.158457409	0.420278449
1.1.1.140	0.030203942	0.046608614	0.046435497	0.013972386	0.000142315	0.001932364	0.515957447
2.7.1.144	0.012934192	0.022174439	0.021111081	0.004757304	3.79844E-05	0.00084637	0.611810682
2.7.7.33	0.001706599	0.001669276	0.001371879	0.002041319	0.238172101	0.399945036	-0.109422492
2.1.1.195	0.013975312	0.007996336	0.014452291	0.013498334	0.546368678	0.686615157	-0.130373426
2.4.1.208	0.009388804	0.006289284	0.013647141	0.005130467	3.10079E-07	4.89924E-05	0.793638732
2.3.1.243	0.000370628	0.000694076	0.00050547	0.000235785	0.366866422	0.516704895	0.378296657
3.4.24.84	0.001090345	0.00082156	0.000965805	0.001214885	0.185272618	0.32924725	-0.424012158
3.5.4.4	0.001421817	0.001699228	0.000768688	0.002074946	0.035508142	0.105854462	-0.155015198
3.1.3.27	0.003009722	0.001394157	0.003210781	0.002808663	0.34086786	0.490420812	0.262158055
3.5.3.11	0.003454917	0.004646218	0.002329708	0.004580126	0.089030196	0.195611925	0.071428571
2.8.2.22	0.001515681	0.001911815	0.000938122	0.00209324	0.000429499	0.004217336	-0.633521494
2.5.1.56	0.004963343	0.006745397	0.006455231	0.003471455	0.178473761	0.321104965	0.159574468
5.1.3.13	0.014373479	0.004634461	0.014349264	0.014397693	0.91055459	0.956219866	-0.299392097
3.1.4.53	0.001799685	0.002876185	0.002003314	0.001596056	0.983548617	1	-0.126848862
3.5.1.10	0.001748002	0.001717201	0.001913277	0.001582727	0.926767515	0.966239916	0.056231003
2.4.1.276	9.03198E-05	0.000179912	0.000168224	1.24153E-05	0.000194325	0.002378425	0.425944018
5.4.99.1	0.009218042	0.006015371	0.006822712	0.011613372	0.016256115	0.061021876	-0.680308294
2.7.1.199	0.012176886	0.015804873	0.010263997	0.014089775	0.506231587	0.649800959	0.298632219
3.2.1.89	0.001194706	0.001273937	0.001078144	0.001311268	0.384241116	0.530429754	-0.230894485
5.1.3.6	0.001128362	0.001674128	0.00138121	0.000875514	0.705414169	0.811389693	0.163048198
6.3.2.5	0.002587722	0.002043063	0.003868323	0.001307121	4.17358E-07	5.1812E-05	0.674446374
1.3.1.108	0.014224243	0.020254841	0.009157571	0.019290915	0.081408821	0.184237696	-0.512049501
3.5.1.23	0.000718636	0.001100254	0.001099552	0.000337721	0.019083522	0.068527191	0.393999152
2.3.1.82	0.000921765	0.001111439	0.001260739	0.000582791	0.006692326	0.031938366	0.306155685
2.7.1.83	0.001949231	0.003953972	0.001980674	0.001917788	0.026633118	0.087008193	0.505048315
2.7.1.76	0.001039719	0.002858993	0.000960571	0.001118868	0.598817997	0.731368732	0.036114813
2.4.1.245	0.000174037	0.000335916	0.000232338	0.000115737	0.344372044	0.492202806	0.190859731
4.2.2.1	0.003758906	0.003460041	0.004504607	0.003013205	0.030266661	0.09546907	0.262483717
2.4.1.10	0.000180108	0.000331891	0.000247983	0.000112233	0.70419244	0.811389693	0.266949932
4.1.1.39	0.001034546	0.001001136	0.00118716	0.000881931	0.395580536	0.543063958	0.295484151
2.4.1.289	0.000875619	0.001164207	0.001189238	0.000562	0.047254209	0.12832471	0.471223077
5.3.2.6	0.006161333	0.006242408	0.00849021	0.003832457	0.006327364	0.030632198	0.590534086
2.4.1.290	0.000177529	0.000249382	0.000124298	0.00023076	0.526174983	0.671433275	-0.218217385
4.1.3.39	0.000451082	0.000811065	0.000238921	0.000663243	0.074968318	0.176312499	-0.372675055
1.1.1.91	0.00023049	0.000297024	0.000224513	0.000236466	0.74847522	0.845559951	0.009910748
3.4.22.37	0.162830402	0.305670019	0.096599418	0.229061386	0.105955089	0.221867402	-0.43475901
2.7.1.198	0.041793484	0.063074551	0.063416023	0.020170945	0.000606942	0.005581297	0.553625706
2.6.1.19	0.000295584	0.000454228	0.000145397	0.000445771	0.050915183	0.135100135	-0.147952102
2.7.1.95	0.000921469	0.001222695	0.001368389	0.000474549	3.41151E-05	0.000796167	0.583586626
4.2.1.32	0.004530043	0.018638645	0.00292033	0.006139756	0.130510373	0.26012274	0.282674772
4.1.3.4	0.000228064	0.000346909	0.000257463	0.000198665	0.176169231	0.319272288	0.096651558
4.1.1.82	0.001565472	0.002534266	0.000426321	0.002704623	0.000180753	0.002309923	-0.543282365
5.4.99.21	0.001551995	0.001577285	0.001638832	0.001465159	0.926063634	0.966239916	-0.036478124
6.3.5.10	0.01115182	0.006391677	0.010252487	0.012051154	0.767049595	0.857319741	-0.376682588
2.1.1.148	0.002000903	0.002251752	0.000781666	0.003220141	1.71057E-05	0.000484774	-0.727933996
1.1.1.41	0.001574309	0.00308561	0.000965888	0.00218273	0.483248591	0.631018822	-0.263413575
2.4.1.52	0.010431416	0.012788587	0.016654416	0.004208416	7.4311E-07	6.7975E-05	0.745549284
3.2.2.24	0.000292679	0.00047307	0.000371823	0.000213535	0.812278847	0.893434284	0.087736566
2.7.8.40	0.001324276	0.002892625	0.0013066	0.001341952	0.975245057	0.997952845	0.277392027
1.4.1.21	5.02588E-05	0.000110873	2.30527E-05	7.74648E-05	0.250810184	0.415174041	-0.278584384

EC	Mean abundance	Std_deviation	Mean abundance - health	Mean abundance - peri-implantitis	P value	FDR corrected P value	LDA correlation (CAP)
5.3.1.27	0.000373404	0.000760856	0.000520092	0.000226715	0.420239421	0.56600887	-0.128658806
4.3.1.2	0.002537867	0.003157524	0.001250309	0.003825426	0.005362539	0.027492901	-0.605570357
2.7.1.16	0.001582811	0.005191865	0.002741477	0.000424144	0.055961569	0.144949638	0.336843248
2.1.1.242	0.000343985	0.000576663	0.000533495	0.000154475	0.012395334	0.049638455	0.583833534
2.5.1.120	0.00058592	0.000766651	0.000355643	0.000816196	0.208452181	0.361566758	-0.367519744
2.4.1.8	0.018907171	0.062470467	0.029237836	0.008576507	0.009356868	0.04015367	0.560573165
3.5.3.9	0.000128924	0.000546855	0.000224938	3.29105E-05	0.254723683	0.418959172	0.252957444
2.7.7.80	0.001149774	0.002616035	0.001869248	0.000430301	0.003525556	0.020561799	0.543544929
1.1.1.105	6.3223E-05	0.000212066	0.000104294	2.21516E-05	0.045117236	0.12546201	0.090263566
3.6.3.24	0.005865578	0.004928614	0.005159221	0.006571934	0.455184044	0.604362008	-0.420972644
3.6.1.67	0.001436561	0.003541384	0.002136248	0.000736874	0.042229004	0.119146118	0.47302139
2.3.2.5	0.000518211	0.000550431	0.000577449	0.000458973	0.87691359	0.93357138	0.345292855
1.8.4.12	0.002294052	0.002524137	0.00305402	0.001534085	0.002936794	0.017909288	0.615610074
3.1.3.71	0.000783873	0.001450594	0.000560764	0.001006982	0.099629508	0.21456764	-0.475429393
1.10.3.12	0.001105954	0.003387511	0.001983952	0.000227956	0.003517504	0.020561799	0.461860053
2.1.3.1	0.001266092	0.001791045	0.001403347	0.001128836	0.320383949	0.489384158	0.194637429
2.7.1.197	0.012272529	0.024336837	0.018136158	0.006408899	0.00148899	0.010693652	0.625054277
5.5.1.2	5.34499E-05	0.000130117	4.29682E-05	6.39317E-05	0.365720456	0.515927072	0.037079497
2.3.2.18	0.001008553	0.001602871	0.001659866	0.000357241	0.000689362	0.006112813	0.382535084
5.1.1.20	0.001642453	0.002368205	0.001084002	0.002200903	0.021717525	0.075039878	-0.406969171
2.7.8.26	0.006895017	0.004072327	0.005839335	0.0079507	0.105955089	0.221867402	-0.544832827
2.7.1.8	0.000144418	0.000313795	0.000239213	4.96226E-05	0.055162792	0.144169823	0.252267427
2.1.2.5	0.027925221	0.01264864	0.023526859	0.032323583	0.025692206	0.085215753	-0.466131133
3.1.3.12	0.000181952	0.000503359	0.000296313	0.000067591	0.10339917	0.219600359	0.16352012
3.5.3.18	0.00038862	0.00056053	0.000377689	0.00039955	0.192913103	0.33969906	-0.312371613
3.5.3.6	0.067557119	0.062976916	0.071356288	0.063757951	0.602179894	0.731368732	0.141554494
2.4.1.292	0.000288298	0.000284507	0.000215184	0.000361411	0.036856623	0.108940154	-0.300185747
5.1.3.4	0.007532169	0.006054826	0.008855538	0.0062088	0.045920684	0.125487655	0.585214937
5.3.1.5	0.001966664	0.001764048	0.0014683	0.002465028	0.00591703	0.029309765	-0.584129396
5.4.3.3	0.028897941	0.023974407	0.019029082	0.0387668	0.00148899	0.010693652	-0.823708207
2.4.1.293	0.000112861	0.000177275	8.6853E-05	0.00013887	0.587223175	0.721702848	-0.174615728
1.2.7.3	0.022782758	0.019248669	0.016709931	0.028855586	0.001174568	0.008953503	-0.830004342
2.7.8.24	0.000142846	0.000194135	0.000164585	0.000121107	0.99172454	1	0.078738998
2.4.1.60	6.01709E-05	0.000296965	2.76844E-05	9.26575E-05	0.098900014	0.213260825	-0.185709166
2.7.1.204	0.013915027	0.021363937	0.021624337	0.006205716	0.000300628	0.003286107	0.658923144
1.1.1.57	0.000584825	0.001041933	0.000851695	0.000317956	0.50275778	0.64966024	0.251418026
3.6.3.16	0.006369545	0.006271357	0.005399022	0.007340068	0.077797772	0.181066372	-0.488927486
2.4.1.7	0.003424135	0.003916243	0.003734378	0.003113892	0.045920684	0.125487655	0.607251411
2.4.2.12	0.000273314	0.00048401	0.000365301	0.000181326	0.220571261	0.378807165	0.370745207
1.4.7.1	0.003780465	0.004207945	0.005144948	0.002415983	0.039407961	0.114533506	0.330004342
1.9.3.1	0.016494014	0.025829195	0.01953033	0.013457698	0.185272618	0.32924725	0.436170213
4.3.1.15	0.011462485	0.01401461	0.012785265	0.010139706	0.053297186	0.140349256	0.3048198
4.2.1.53	0.011340787	0.028953221	0.015751033	0.006930542	0.010616164	0.043930696	0.491641337
1.1.99.1	0.000479957	0.00131011	0.000854603	0.000105312	0.004119567	0.023083911	0.40295566
4.1.2.19	0.001674163	0.001627644	0.001743494	0.001604833	0.255039588	0.418959172	-0.103343465
2.3.2.3	0.003964319	0.005567015	0.006468472	0.001460166	0.000156664	0.002031961	0.595853235
1.5.1.28	0.000201148	0.000708375	0.000315868	8.64283E-05	0.497390203	0.64370745	0.057395253
3.2.1.3	2.47254E-05	7.97946E-05	2.44112E-05	2.50395E-05	0.134065144	0.265684402	0.237959089
2.3.1.19	0.004841487	0.004496465	0.003211263	0.006471711	0.007712559	0.034998504	-0.597698654
2.1.1.137	0.002251368	0.002483193	0.001397538	0.003105198	0.025948416	0.085901614	-0.550981084
1.12.99.6	0.010582704	0.009675513	0.011769871	0.009395536	0.281836841	0.455448476	0.302540165
1.1.1.339	0.000731865	0.000909435	0.000911787	0.000551944	0.092549416	0.201820434	0.451471152
1.2.1.8	0.000810496	0.00242163	0.001477052	0.000143941	0.001601336	0.011371391	0.503719004
4.2.1.6	9.44455E-05	0.000318547	0.000112937	0.000075954	0.921656259	0.963801792	-0.097453966
3.1.3.104	0.004016136	0.00305619	0.006071612	0.00196066	3.82801E-08	2.21769E-05	0.825879288
2.4.1.187	0.003088275	0.001803174	0.003065598	0.003110952	0.61651648	0.739990085	-0.155557968
5.4.99.62	0.001084605	0.001305419	0.001400479	0.000768731	0.077797772	0.181066372	0.519431177

EC	Mean abundance	Std_deviation	Mean abundance - health	Mean abundance - peri-implantitis	P value	FDR corrected P value	LDA correlation (CAP)
3.2.1.40	0.001671865	0.002342315	0.001028916	0.002314815	0.050737544	0.134834635	-0.376899696
4.1.1.74	6.15764E-05	0.000204097	5.13624E-05	7.17905E-05	0.761891469	0.854301531	-0.006103222
2.6.1.83	0.003815417	0.003961027	0.003015269	0.004615565	0.077797772	0.181066372	-0.398176292
3.4.19.11	0.000251983	0.000526028	0.000223336	0.000280629	0.647531033	0.76610547	-0.185772327
4.6.1.13	0.001009702	0.001790002	0.001133913	0.000885492	0.358384719	0.508052725	0.025425812
2.7.1.176	0.005906543	0.00819782	0.006668322	0.005144764	0.101506067	0.216463245	0.20668693
1.21.4.1	0.032517893	0.034199512	0.034610277	0.030425509	0.418845832	0.565922893	-0.005861919
2.3.1.209	7.28598E-05	0.000212739	0.000013171	0.000132549	0.321589139	0.489384158	-0.105553982
2.6.1.92	0.000422846	0.000422621	0.000272113	0.00057358	0.00588324	0.029309765	-0.396546165
1.13.11.2	0.005185492	0.00621264	0.007839702	0.002531282	4.69671E-05	0.000927601	0.757924446
3.6.3.4	0.010823553	0.010966936	0.012499464	0.009147642	0.44288188	0.590282751	0.311984368
2.4.1.12	0.000587337	0.00067455	0.000722546	0.000452129	0.135903357	0.267800492	0.328267477
3.5.1.105	0.000237235	0.000434362	0.000143694	0.000330775	0.090797571	0.198248969	-0.133222843
2.8.3.21	6.13862E-05	0.000245827	9.49238E-05	2.78485E-05	0.482431366	0.63089971	-0.095598149
2.6.1.88	3.37886E-05	6.2512E-05	4.03806E-05	2.71966E-05	0.177376112	0.320790513	0.257895298
2.1.1.61	0.000613838	0.001005593	0.000864144	0.000363532	0.165425496	0.302323356	0.377442466
3.5.4.25	0.000873871	0.001039629	0.001109682	0.000638061	0.129624174	0.26012274	0.404917633
1.1.1.65	0.000190627	0.00031846	0.000193971	0.000187283	0.474865507	0.625140455	0.240331739
2.4.2.45	0.000029983	7.46038E-05	3.60996E-05	2.38664E-05	0.105546762	0.221867402	0.255231907
1.13.11.27	6.9345E-05	0.000176293	8.64723E-05	5.22178E-05	0.868155962	0.929670401	-0.136020792
1.2.1.9	0.01521703	0.01467525	0.019198035	0.011236026	0.06461309	0.158457409	0.473187147
4.2.3.3	0.001027518	0.000815159	0.000794384	0.001260652	0.040181504	0.116005736	-0.526218655
2.7.1.60	0.000453937	0.001185602	0.000705787	0.000202087	0.43938886	0.587881323	0.272180444
3.1.4.3	7.32234E-05	0.000197126	9.44945E-05	5.19522E-05	0.348901899	0.497042213	0.123099129
2.8.3.19	0.000341507	0.000581249	0.00020652	0.000476494	0.220571261	0.378807165	-0.193144693
1.16.3.2	0.044407105	0.028646272	0.039796981	0.04901723	0.024303877	0.081387551	-0.248588797
2.1.1.197	0.002046695	0.002011807	0.001960068	0.002133321	0.602179894	0.731368732	-0.362462006
3.1.2.28	0.002697725	0.001367129	0.001897935	0.003497515	1.3982E-05	0.000433941	-0.682696483
4.2.1.28	0.017563	0.103304554	0.033030049	0.002095951	0.272703911	0.444198124	0.125814155
1.17.99.7	0.001436245	0.001660656	0.001282742	0.001589747	0.679969308	0.791021859	-0.086215324
1.3.3.3	0.000168112	0.000291164	0.000173343	0.000162882	0.79410138	0.881883833	0.260400096
2.3.1.48	0.000268368	0.000649927	0.000110464	0.000426271	0.030178858	0.09546907	-0.383647851
5.1.1.12	0.000122416	0.000215364	0.000142822	0.00010201	0.772587804	0.861648321	-0.104809134
2.7.7.49	0.012022247	0.034045024	0.012175186	0.011869309	0.846132513	0.915107845	0.034954407
4.2.1.70	0.002752104	0.00302625	0.002609419	0.002894789	0.602179894	0.731368732	0.353343465
5.4.99.20	0.000489247	0.000861317	0.000713741	0.000264752	0.031089587	0.09788714	0.545563239
1.14.99.58	0.001253389	0.002364927	0.001258508	0.001248269	0.627191591	0.750119378	0.257215822
3.1.3.89	0.001238315	0.001249416	0.001349211	0.001127419	0.238172101	0.399945036	0.238927486
3.5.99.7	0.000544847	0.000571316	0.000483711	0.000605984	0.549480536	0.690026858	0.201124694
3.7.1.20	1.5532E-05	4.39604E-05	1.35375E-05	1.75266E-05	0.768718583	0.858081501	-0.024621541
1.10.2.2	0.002160753	0.002504485	0.002128039	0.002193467	0.506231587	0.649800959	0.484476769
2.3.3.9	0.000398942	0.000737602	0.000571288	0.000226596	0.043897336	0.12285599	0.557246546
1.97.1.9	0.011476641	0.017894427	0.009851331	0.013101951	0.493199174	0.639686689	0.206361268
6.2.1.41	2.86175E-05	5.52221E-05	3.28903E-05	2.43447E-05	0.786889511	0.874433485	-0.202948598
3.1.1.81	0.000130448	0.000306093	8.19963E-05	0.000178899	0.805856806	0.889821556	0.172347805
3.4.21.66	0.000177499	0.00045445	0.000243131	0.000111867	0.237199126	0.399856528	0.036709018
3.1.27.1	0.000252494	0.000625457	0.000432751	7.22377E-05	0.015680052	0.058986862	0.345532919
1.2.1.38	0.003621849	0.003809239	0.003981464	0.003262234	0.61651648	0.739990085	0.099109857
2.7.1.90	0.001686023	0.002027587	0.001419543	0.001952503	0.991851953	1	0.159683022
1.2.5.3	0.001979907	0.001861851	0.001913441	0.002046373	0.573959013	0.706974319	-0.168475901
3.7.1.22	0.001263945	0.00204843	0.001493884	0.001034005	0.141463386	0.276250972	-0.103017803
1.8.1.15	0.002332426	0.003691692	0.00355205	0.001112803	0.003762988	0.021372789	0.525892957
3.1.25.1	0.000220678	0.000580482	0.000354593	8.67633E-05	0.556071531	0.69679331	0.344010625
3.5.1.11	0.001288329	0.00204394	0.001779022	0.000797636	0.082971621	0.187173947	0.377638505
6.2.1.20	0.000116309	0.000186924	0.000137904	9.47143E-05	0.524247356	0.66946503	0.264741929
1.3.8.6	0.013190183	0.030731322	0.017088758	0.009291609	0.943010334	0.976729416	0.181936604
3.1.4.37	0.002856571	0.002275722	0.002790236	0.002922906	0.814269221	0.893434284	-0.259769865

EC	Mean abundance	Std_deviation	Mean abundance - health	Mean abundance - peri-implantitis	P value	FDR corrected P value	LDA correlation (CAP)
2.5.1.46	0.004266626	0.007368014	0.003996236	0.004537016	0.73365983	0.834513772	0.168059929
2.4.1.166	0.001102525	0.001399422	0.001649767	0.000555283	0.007757057	0.035108761	0.432999408
1.21.98.1	0.000716149	0.000803178	0.000463201	0.000969096	0.040181504	0.116005736	-0.462381937
4.4.1.11	0.012312868	0.010180746	0.007959419	0.016666317	0.001174568	0.008953503	-0.76498046
1.1.1.306	0.000842511	0.001282572	0.001360375	0.000324647	0.061180943	0.154307626	0.306579724
2.7.1.19	0.000130835	0.000397071	2.86492E-05	0.000233021	0.189703597	0.336433521	-0.100953386
2.3.1.203	0.000434608	0.000900428	0.000597819	0.000271397	0.836269001	0.91120856	-0.326515921
3.4.19.5	0.000266911	0.000417499	0.000249572	0.00028425	0.942451995	0.976729416	-0.120744887
2.3.1.247	0.01694046	0.014680512	0.01188705	0.02199387	0.011299384	0.045991404	-0.773773339
6.2.1.48	0.000161597	0.000269681	0.000232817	9.03769E-05	0.106959352	0.223431916	0.199217145
1.11.1.1	0.005553757	0.008028397	0.007231908	0.003875606	0.005205424	0.026766353	0.610741716
4.1.3.40	0.000145565	0.000347141	0.00018488	0.00010625	0.424679021	0.57083692	0.416477563
2.1.1.132	0.001006868	0.00162934	0.00058538	0.001428357	0.028049799	0.090783148	-0.478581917
4.1.1.68	1.13578E-05	5.46942E-05	2.21906E-05	0.000000525	0.144764517	0.281747738	0.137371194
3.5.3.8	0.006407791	0.002975369	0.006388726	0.006426855	0.645619886	0.764364688	-0.128528007
1.13.12.4	0.011261614	0.014016401	0.016006678	0.006516549	0.015318868	0.057878678	0.594767694
5.1.3.7	0.001093024	0.001811318	0.000466325	0.001719724	0.04330629	0.121790181	-0.527068937
5.1.99.1	0.009233342	0.006724707	0.007142239	0.011324446	0.012020504	0.048360269	-0.521168042
2.4.2.28	0.001813434	0.001513283	0.001755569	0.001871299	0.733680693	0.834513772	0.100469509
2.1.1.246	5.41617E-05	0.000110712	2.75391E-05	8.07843E-05	0.1834175	0.327962566	-0.256795661
3.4.24.20	0.000122411	0.000308939	4.67127E-05	0.000198109	0.337800355	0.489384158	-0.058613781
1.14.12.10	3.00703E-05	0.00012191	5.90873E-05	1.05333E-06	0.000349106	0.003633213	0.26831056
2.5.1.26	0.00016517	0.000331237	0.000254583	7.57563E-05	0.112101084	0.232218932	0.266046486
3.1.3.105	9.92266E-05	0.000158824	0.00009017	0.000108283	0.752635928	0.845559951	0.235000618
4.2.1.79	0.000123364	0.000357823	0.000144897	0.00010183	0.676362223	0.787352675	-0.036795895
1.2.1.3	0.00325478	0.00505147	0.004584651	0.001924909	0.058733676	0.149456998	0.288536691
2.7.1.175	0.000333327	0.000371802	0.000375754	0.0002909	0.157773503	0.299420483	0.330257303
3.4.24.70	0.002093521	0.003110482	0.002644781	0.001542261	0.028671131	0.091431973	0.551128962
6.5.1.1	8.70132E-05	0.000326847	0.000163293	1.07333E-05	0.10446669	0.221418424	0.174222625
3.2.1.4	0.003870066	0.003370032	0.003116925	0.004623208	0.165425496	0.302323356	-0.291901867
4.1.3.30	0.000313464	0.000641906	0.000224975	0.000401952	0.561893913	0.70105644	0.061849788
5.5.1.19	3.06875E-06	1.43065E-05	1.62083E-06	4.51667E-06	0.572000957	0.706974319	0.104515084
2.7.7.82	7.96115E-05	0.00017147	3.87739E-05	0.000120449	0.113170483	0.234155119	-0.370325606
1.5.1.40	0.00082306	0.002220511	0.001196372	0.000449748	0.326905914	0.489384158	0.448863376
4.1.99.1	0.061250137	0.054125244	0.034840644	0.087659631	0.000142315	0.001932364	-0.869626574
1.8.99.2	0.000951621	0.00259672	0.00024003	0.001663211	0.019936692	0.070714227	-0.429201907
6.3.4.6	7.49144E-05	0.000167489	0.000124763	2.5066E-05	0.004781403	0.025635801	0.347253238
3.5.1.16	0.00126896	0.001984383	0.001056177	0.001481743	0.630998004	0.750119378	0.142640035
5.3.3.14	0.005446121	0.005724544	0.0074965	0.003395741	0.035508142	0.105854462	0.423903604
4.1.99.2	0.153867708	0.133667769	0.107467743	0.200267674	0.013583315	0.052578624	-0.59118541
5.3.1.17	0.00276793	0.002263443	0.001616345	0.003919514	3.41151E-05	0.000796167	-0.595744681
6.2.1.14	0.001075306	0.001454991	0.0015768	0.000573813	0.003522871	0.020561799	0.470307244
1.14.14.5	8.83518E-05	0.000190264	0.000136877	3.98263E-05	0.012061658	0.04841377	0.442704476
1.17.1.11	0.000146697	0.000363408	0.000113209	0.000180185	0.741601003	0.840771391	-0.367774485
4.2.1.117	0.000389547	0.001125743	0.000257303	0.000521791	0.491145162	0.639408458	0.296883566
2.3.1.245	0.000137781	0.00021777	0.000101987	0.000173575	0.118378996	0.242907551	-0.494522946
6.3.4.22	0	0	0	0	NA	NA	0
1.1.1.336	0.003601444	0.005053095	0.002053163	0.005149724	0.006327364	0.030632198	-0.596504559
2.6.1.37	0.002134727	0.00364304	0.000671055	0.003598399	0.001782792	0.012295606	-0.612094239
3.1.2.29	0.000516764	0.000923433	0.000280167	0.000753361	0.119170542	0.244243398	-0.31548652
6.2.1.17	3.47229E-05	9.01698E-05	6.33625E-05	6.08333E-06	0.039324523	0.114533506	0.044324443
3.2.1.31	0.000614777	0.000998854	0.000650316	0.000579238	0.018215582	0.066186695	0.417652809
3.2.1.141	0.001337455	0.004743462	0.000867643	0.001807267	0.812554047	0.893434284	0.06887942
3.1.6.6	7.2311E-05	0.000169838	0.000114842	2.97805E-05	0.131445225	0.261087772	0.107115998
2.7.1.221	0.000392643	0.00062515	0.00037328	0.000412006	0.162599656	0.301277401	0.414508856
1.1.1.11	1.29971E-05	3.86844E-05	0.000013325	1.26692E-05	0.650245177	0.767748722	0.183186275
2.7.8.41	0.000208495	0.000516705	6.0583E-05	0.000356406	0.121995622	0.248276805	-0.387275801

EC	Mean abundance	Std_deviation	Mean abundance - health	Mean abundance - peri-implantitis	P value	FDR corrected P value	LDA correlation (CAP)
1.18.1.1	0.000118874	0.000175946	0.000114151	0.000123597	0.497410302	0.64370745	-0.238855808
2.7.1.168	0.000826853	0.001711342	0.000689578	0.000964129	0.006085134	0.030021434	-0.432379624
2.7.7.99	0.000363776	0.0007521	0.000264239	0.000463313	0.823975447	0.901806881	0.273964868
3.5.1.2	0.009227854	0.004801731	0.007728389	0.010727318	0.030266661	0.09546907	-0.553951368
3.2.1.28	0.000178769	0.000330335	0.000221456	0.000136083	0.241895382	0.405023289	0.093341184
1.14.13.83	1.94183E-05	5.51301E-05	1.72874E-05	2.15492E-05	0.727682909	0.829319931	0.024548679
2.1.1.196	0.002745744	0.002134493	0.00278299	0.002708499	0.407115953	0.554954923	-0.334780721
1.3.5.3	0.000438584	0.000646559	0.000597655	0.000279514	0.081279985	0.184237696	0.523297568
4.2.99.21	6.65334E-05	0.00015019	6.3742E-05	6.93249E-05	0.241548934	0.404833219	-0.020711258
1.3.1.74	5.13752E-05	8.32887E-05	2.5897E-05	7.68534E-05	0.103482563	0.219600359	-0.256348804
3.2.1.156	0.000318401	0.00072822	0.000390372	0.000246429	0.914762065	0.958900162	0.131518558
3.5.3.1	0.000466664	0.000948367	0.000516208	0.00041712	0.877086067	0.93357138	-0.377700578
4.2.1.7	0.000312211	0.000530082	0.000348347	0.000276076	0.046399228	0.126596323	0.130826967
1.14.99.57	0.001379234	0.005597306	0.002584456	0.000174012	0.000167761	0.002159761	0.625342633
1.1.1.251	7.84848E-05	0.000319448	0.000144925	1.20443E-05	0.34961243	0.497646522	0.111245093
5.4.99.15	0.000173585	0.000218992	0.000168876	0.000178294	0.975245057	0.997952845	0.08113744
1.7.1.7	0.009236351	0.006439926	0.01313108	0.005341621	8.74923E-06	0.000316795	0.733282675
4.3.1.18	0.001697393	0.002523537	0.001323649	0.002071136	0.705414169	0.811389693	-0.443768997
1.2.3.3	0.108497252	0.098292352	0.156721919	0.060272585	0.000329015	0.00344475	0.689318281
2.1.1.200	0.001698797	0.003302676	0.002436109	0.000961484	0.279003266	0.453184745	0.273075149
5.3.3.17	0.000133129	0.000227792	0.000112917	0.000153342	0.173692104	0.315111562	-0.401857059
3.5.1.81	0.000919358	0.001148288	0.001017437	0.000821278	0.369691028	0.518636598	-0.311583978
5.4.99.26	0.000392407	0.000947628	0.000641446	0.000143368	0.058374808	0.149456998	0.387934983
3.5.2.5	0.000227535	0.000356424	0.000310954	0.000144116	0.223646152	0.381075502	0.090993
1.6.1.1	0.000138755	0.000294813	0.00022954	4.79712E-05	0.07356794	0.174197657	0.154999976
5.1.3.11	0.000393847	0.000576713	0.000203878	0.000583816	0.009916718	0.042259647	-0.511304684
3.6.1.63	0.000101234	0.000215104	0.000157505	4.49623E-05	0.123209678	0.250454292	0.118791908
2.7.1.189	0.000316444	0.00061869	0.000335154	0.000297735	0.77905453	0.867390629	-0.278220864
5.3.99.11	0.000175807	0.000759354	0.000331332	2.02822E-05	0.511606316	0.655248178	0.08650511
6.2.1.25	1.66435E-05	6.38589E-05	1.39134E-05	1.93737E-05	1	1	0.107779155
3.1.1.11	0.00192957	0.0021261	0.000866995	0.002992146	6.05566E-05	0.001096327	-0.6178522
4.1.99.14	0.001476603	0.000966635	0.001463786	0.00148942	0.705414169	0.811389693	-0.448979592
3.2.2.8	0.000655251	0.002189252	0.000446919	0.000863583	0.366866422	0.516704895	0.232067089
1.2.1.81	8.7205E-06	3.15921E-05	0.000005849	0.000011592	0.098796683	0.213260825	-0.239456755
1.11.1.10	0.000400345	0.000681332	0.000658252	0.000142439	0.001480663	0.010693652	0.450112935
5.3.3.19	9.45477E-05	0.000182398	0.000104974	8.41211E-05	0.83675999	0.91120856	0.040362793
4.4.1.3	0.000835955	0.001167516	0.000693754	0.000978155	0.725930771	0.827865932	-0.226287079
2.4.2.30	7.30529E-05	0.000176031	4.44017E-05	0.000101704	0.740932086	0.840627106	-0.157986248
3.4.21.121	0.000704603	0.002680282	0.000755256	0.00065395	0.654132926	0.77042391	0.175808336
2.8.3.12	0.034772374	0.021898982	0.02304344	0.046501308	0.000228358	0.002681659	-0.830438558
2.4.2.52	0.002141284	0.001395421	0.002062154	0.002220414	0.407115953	0.554954923	-0.270082501
3.1.4.52	0.001163484	0.00278115	0.001467291	0.000859677	0.702851617	0.811389693	0.160338698
3.2.1.180	0.003082211	0.004233615	0.003416306	0.002748116	0.467672658	0.616703398	0.216022579
3.4.17.11	0.000121766	0.000222551	6.52656E-05	0.000178267	0.008111551	0.036374662	-0.437204021
1.21.4.3	0.001798393	0.003220537	0.001809652	0.001787135	0.958825385	0.987691024	-0.156882113
2.6.1.112	0.000502313	0.000810485	0.00060539	0.000399236	0.034071323	0.103476952	0.252705707
1.3.8.4	0.000157221	0.000236162	0.000159549	0.000154893	0.508068804	0.651486726	0.226649219
4.1.2.43	0.000625955	0.002065075	0.001053309	0.0001986	0.342099775	0.491379677	0.042814968
1.8.5.4	0.000993277	0.001536113	0.000599323	0.001387231	0.45099111	0.599252713	-0.277560661
1.1.1.58	0.00022517	0.000533291	0.000226143	0.000224197	0.975245057	0.997952845	0.110434046
2.7.1.203	1.05406E-05	5.55433E-05	1.58153E-05	5.26583E-06	0.344004795	0.492119174	-0.079595394
3.1.21.7	0.000124175	0.000549223	0.000224152	2.41978E-05	0.157595952	0.299420483	0.395740062
2.5.1.101	4.7545E-05	0.000138994	6.59334E-05	2.91567E-05	0.87307532	0.93357138	0.060295854
2.4.2.53	0.000155716	0.000267231	9.53862E-05	0.000216046	0.205194192	0.357700607	-0.498185427
4.2.1.151	0.000509942	0.000765079	0.000210781	0.000809102	0.045900375	0.125487655	-0.590941947
2.7.1.51	0.000210715	0.000853609	0.000346465	7.49655E-05	0.370888588	0.519842231	0.042254542
1.1.1.405	0.000487757	0.001306072	0.000633237	0.000342278	0.358110107	0.508052725	-0.379213312

EC	Mean abundance	Std_deviation	Mean abundance - health	Mean abundance - peri-implantitis	P value	FDR corrected P value	LDA correlation (CAP)
4.1.1.83	5.3233E-05	0.000124321	3.67384E-05	6.97277E-05	0.243037236	0.406152611	-0.270943171
2.6.1.59	0.001376784	0.002485555	0.000755902	0.001997666	0.247763426	0.410890109	-0.292994455
3.4.15.6	0.000239143	0.000348415	0.000344545	0.000133741	0.008172857	0.036374662	0.533372076
1.1.99.3	1.58719E-05	6.4234E-05	2.62812E-05	5.4625E-06	0.660627721	0.775267373	-0.133423667
2.8.1.11	0.000190201	0.000379271	0.000133822	0.000246581	0.467008287	0.616703398	0.029977395
3.6.1.26	0.000790773	0.00140685	0.000977115	0.000604432	0.035847188	0.10613699	0.293370088
4.1.1.2	0.000235367	0.000574139	0.000362151	0.000108584	0.22042706	0.378807165	0.197931829
2.3.1.16	0.000230124	0.000735934	0.000405919	5.43277E-05	0.021894208	0.075500266	0.337562057
1.4.1.11	0.019650337	0.018319611	0.014480164	0.02482051	0.004823307	0.025635801	-0.756513244
2.3.3.5	0.000502688	0.001508074	0.000241225	0.00076415	0.818348305	0.897343441	0.06749078
4.1.1.47	9.08447E-05	0.000222669	0.000107201	7.44888E-05	0.939491241	0.976729416	0.33197969
1.1.1.261	0.0004237	0.000835399	0.000498287	0.000349113	0.852638155	0.921294276	-0.145600805
3.6.1.45	0.000211931	0.000377313	0.000268836	0.000155026	0.909658189	0.956219866	-0.014662759
2.7.8.37	5.20132E-05	0.000177492	0.000102406	1.62083E-06	0.002144171	0.014388301	0.501712594
2.7.1.218	0.000235	0.000548305	0.000285758	0.000184242	0.17917372	0.322030947	-0.12773358
2.4.2.4	0.000103712	0.000166313	0.000114203	9.32204E-05	0.416766967	0.565922893	0.398858954
3.2.1.170	0.000367788	0.000693433	0.000508633	0.000226943	0.153865306	0.294513108	0.417801112
1.11.2.4	0.000133641	0.000562872	0.00024562	2.16623E-05	0.041531735	0.117944699	0.424382461
3.1.3.8	9.92985E-05	0.000256615	0.000166536	3.20615E-05	0.062438801	0.15591758	0.293130433
1.3.3.5	3.89855E-05	0.000122573	3.71558E-05	4.08153E-05	0.420435818	0.56600887	0.208017893
3.2.1.199	0.000165226	0.000628667	0.000296609	3.3843E-05	0.120068522	0.24493926	0.199501273
4.2.2.2	0.000455695	0.000796941	0.000406691	0.000504699	0.67204229	0.786603029	0.151446142
2.6.1.76	0.000266128	0.000481734	0.000252268	0.000279988	0.139105577	0.273800105	0.324114124
4.1.1.104	0.000137414	0.000523166	9.60449E-05	0.000178782	0.339213183	0.490420812	0.187740943
2.7.1.58	3.0672E-05	9.69148E-05	5.76191E-05	0.000003725	0.017961341	0.065719603	0.192844168
1.14.11.2	3.11289E-05	9.0629E-05	2.21568E-05	4.01009E-05	0.772905485	0.861648321	0.058787887
3.1.1.24	0.000189794	0.000705193	0.000298405	8.11828E-05	0.290289063	0.467150362	0.097013316
1.1.1.135	4.60882E-05	7.6269E-05	3.11717E-05	6.10047E-05	0.155239153	0.296164267	-0.229619617
4.1.1.36	5.99539E-05	0.000100062	5.5502E-05	6.44057E-05	0.835523522	0.91100369	-0.20135117
2.7.10.1	9.25562E-05	0.00024082	8.83333E-05	9.67791E-05	0.605766111	0.731543474	0.319205732
4.1.1.35	0.000632934	0.000819127	0.000337579	0.000928289	0.00824604	0.036374662	-0.615217728
2.4.1.336	0.000202367	0.000374728	0.000257846	0.000146889	0.041769901	0.118427548	0.257107308
3.6.1.61	0.000112062	0.000452541	9.13901E-05	0.000132733	0.504748588	0.649800959	0.205265882
3.4.22.48	2.95748E-05	0.000117677	5.91496E-05	0	0.010294231	0.043215876	0.354887191
1.14.13.239	2.86958E-05	0.000124844	5.4829E-05	2.5625E-06	0.024119531	0.081387551	0.135283176
3.2.1.172	0.000249681	0.000589655	0.000410681	8.86811E-05	0.250824363	0.415174041	0.242153403
5.1.3.32	0.000625611	0.001980426	0.000303105	0.000948118	0.049605773	0.133047583	-0.407464887
2.7.7.47	0.000140793	0.000284777	0.000232581	4.90048E-05	0.003734422	0.021280083	0.429958279
3.1.1.45	0.000710647	0.001130099	0.000475797	0.000945496	0.188425668	0.334508489	-0.351883177
2.4.1.345	0.000265162	0.000374228	0.000238526	0.000291799	1	1	0.182157064
3.2.1.85	0.003418199	0.011347393	0.00144093	0.005395467	0.362345299	0.511996853	-0.170545
1.14.12.17	0.001388828	0.002218963	0.001708598	0.001069059	0.322286463	0.489384158	0.237522729
3.2.1.139	0.000298143	0.000698036	0.000162453	0.000433834	0.132599147	0.263079129	-0.248229879
1.1.1.367	0.000946021	0.001747118	0.000531179	0.001360862	0.555470063	0.696541825	-0.153562135
2.3.2.13	0.000262892	0.00044581	0.000287247	0.000238537	0.575997633	0.708982922	0.471013134
2.1.1.152	0.000101196	0.000204238	5.83841E-05	0.000144009	0.267307562	0.437458138	-0.088222621
6.2.1.34	1.54408E-05	5.50631E-05	2.63265E-05	0.000004555	0.861315265	0.924389386	0.314629538
2.7.7.66	1.50625E-06	1.03263E-05	3.0125E-06	0	0.3378947	0.489384158	0.121085619
3.5.4.19	0.000138504	0.000245019	0.000151378	0.000125629	0.245951276	0.409285723	0.303616178
2.4.1.287	0.000252878	0.000419631	0.000271882	0.000233874	0.57051169	0.706974319	0.205134689
1.21.4.4	0.001585832	0.002205703	0.001241392	0.001930272	0.33752139	0.489384158	-0.279005385
4.2.2.8	0.000262351	0.001256942	4.6077E-05	0.000478625	0.143640295	0.280187243	-0.442527893
1.1.1.291	5.94369E-05	0.000219158	2.44538E-05	9.442E-05	0.116954725	0.24026869	-0.12248057
2.7.8.42	9.83871E-06	2.36058E-05	9.66804E-06	1.00094E-05	1	1	0.265535057
1.1.1.350	0.02189669	0.033535578	0.026901211	0.016892169	0.135903357	0.267800492	0.343899262
2.4.1.58	0.001908765	0.003846306	0.003218524	0.000599007	3.79844E-05	0.00084637	0.600195397
1.14.11.17	4.01011E-05	0.000109988	7.47314E-05	5.47083E-06	0.020872646	0.072498376	0.294815671

EC	Mean abundance	Std_deviation	Mean abundance - health	Mean abundance - peri-implantitis	P value	FDR corrected P value	LDA correlation (CAP)
2.7.1.187	4.62169E-05	0.000124547	3.22581E-05	6.01757E-05	0.571071387	0.706974319	-0.091132202
4.1.1.12	0.001132926	0.002256621	0.001371126	0.000894725	0.764734534	0.85638442	-0.262570406
2.4.1.20	0.000132711	0.000284878	0.000122404	0.000143019	0.803038746	0.887838003	-0.354815783
1.12.1.3	0.000752234	0.002184424	0.000650286	0.000854182	0.857100564	0.923612514	0.012881312
2.1.1.144	2.44081E-05	7.14849E-05	4.50788E-05	3.7375E-06	0.032183727	0.099884494	0.375151811
1.5.3.1	7.35405E-05	0.000194024	9.85621E-05	4.8519E-05	0.653378458	0.77042391	0.119603772
3.1.1.102	0.000133923	0.000200339	0.000158295	0.000109551	0.33283061	0.489384158	0.071048378
4.1.1.8	0.000129559	0.000338261	0.000103586	0.000155532	0.860034475	0.924389386	0.12857052
6.4.1.4	6.50181E-05	0.0002435	0.000110934	1.91018E-05	0.143937342	0.280451905	-0.057757185
1.12.7.2	0.000303558	0.000415207	0.000172124	0.000434992	0.068960252	0.167158882	-0.452944279
1.10.3.10	0.000425587	0.000810347	0.000690554	0.00016062	0.000988273	0.007915289	0.538378532
2.4.1.325	7.4988E-05	0.000250981	0.000110722	0.000039254	0.251744487	0.416300588	0.307380456
1.20.4.3	0.000900493	0.001232915	0.001383843	0.000417143	0.007039635	0.033067259	0.436725881
1.1.1.67	7.34482E-05	0.000126669	5.76981E-05	8.91983E-05	0.086574949	0.192167639	-0.1365096
1.1.1.302	0	0	0	0	NA	NA	0
2.3.2.17	0.00049401	0.001096298	0.00093332	5.47006E-05	0.001037483	0.008271313	0.238253279
2.4.2.47	3.58812E-05	0.000103003	4.68706E-05	2.48918E-05	0.644825294	0.764364688	0.063738692
2.8.3.5	0.000183429	0.000370993	0.000265059	0.000101799	0.733101688	0.834513772	0.237243574
2.8.3.1	0.001775395	0.002318448	0.001729064	0.001821726	0.369730003	0.518636598	-0.36800825
3.5.3.15	0.000104434	0.000232008	0.000131237	7.76318E-05	0.585680367	0.720390997	0.114790506
3.5.1.32	4.14199E-05	9.90227E-05	2.67122E-05	5.61277E-05	0.177908988	0.321086004	-0.262546343
1.1.1.333	0.000077603	0.000171545	0.000106279	4.89271E-05	0.062127702	0.155363952	0.284127155
1.2.1.76	0.022716846	0.027828776	0.012269178	0.033164514	0.033682558	0.102522391	-0.586734694
1.3.1.22	0.000470094	0.001214913	0.000160016	0.000780172	0.129854762	0.26012274	-0.312497277
2.4.1.180	0.00079112	0.001643356	0.000298266	0.001283974	0.034462621	0.104167016	-0.481955726
1.1.1.159	0.000268952	0.000440271	0.000184596	0.000353307	0.059063136	0.149751441	-0.071575985
1.1.1.385	5.74111E-05	0.000303849	9.72363E-05	1.75858E-05	0.087585531	0.194162822	0.244890766
2.4.1.349	0.000417703	0.001355312	0.000181195	0.00065421	0.959039757	0.987691024	-0.09307907
3.2.1.65	0.000155504	0.000717465	0.000267735	4.32725E-05	0.66572436	0.780721281	-0.017334824
1.2.1.10	0.000448501	0.000966894	0.000586887	0.000310116	0.715512904	0.821374786	-0.124334661
2.4.1.144	2.48689E-05	0.000124603	0.00000525	4.44878E-05	0.379300746	0.527801998	-0.302934049
1.1.1.34	6.60625E-06	2.02255E-05	9.08333E-06	4.12917E-06	0.914269083	0.958900162	0.27780762
3.6.3.55	0.000295593	0.001256686	0.000098487	0.000492699	0.037749487	0.110825352	-0.214197918
5.3.1.15	0.000100962	0.00026291	0.000111878	9.00458E-05	0.163760961	0.302323356	-0.06923223
2.7.11.5	0.000183748	0.000505222	0.000317012	5.04838E-05	0.015579913	0.058737286	0.325656064
2.6.1.103	1.68689E-05	5.29923E-05	3.22879E-05	0.00000145	0.035801554	0.10613699	0.223501402
3.5.3.23	9.35415E-05	0.000324444	0.000148005	3.90775E-05	1	1	0.003503969
5.1.3.24	8.07317E-05	0.000194656	0.000128176	3.32872E-05	0.070135605	0.169174036	0.271300921
4.1.3.1	0.000599012	0.000909846	0.000713884	0.00048414	0.197370914	0.345797025	0.420218268
3.4.21.116	7.65044E-05	0.000201232	0.000118287	3.47214E-05	0.26118845	0.428250497	0.077542158
3.1.4.14	2.26541E-05	9.35402E-05	0.000044484	8.24167E-07	0.323720698	0.489384158	0.052330983
2.7.1.113	0.000118022	0.000389011	0.000209699	2.63441E-05	0.046505809	0.126688238	0.257486586
4.2.1.42	9.25859E-05	0.000318406	0.000151396	3.37763E-05	0.193202873	0.339864974	0.188044044
3.4.14.13	7.353E-05	0.000203596	0.000112336	3.47241E-05	0.073382864	0.173996477	0.325975482
3.1.3.85	9.82293E-05	0.000246502	0.000122848	7.36103E-05	0.830991437	0.906745422	0.068353725
1.3.99.33	0.00022924	0.000297654	0.000205098	0.000253383	0.427541885	0.574240955	-0.355260916
4.1.1.22	0.008254921	0.054339393	0.015901202	0.00060864	0.292408947	0.469692006	0.040219351
1.1.3.6	1.89015E-05	5.63752E-05	3.62655E-05	1.5375E-06	0.009629385	0.041221356	0.363447981
2.6.1.87	4.44929E-05	0.000117223	7.53988E-05	1.3587E-05	0.056260228	0.145506364	0.188394103
5.3.3.7	1.27917E-06	6.45258E-06	2.55833E-06	0	0.161691678	0.299914766	0.162237233
2.7.1.25	0.000478995	0.000895777	0.000537226	0.000420765	0.641601728	0.761163005	-0.119146791
1.14.13.127	1.9025E-05	6.57852E-05	3.43205E-05	3.72958E-06	0.302828169	0.482415543	0.0740882
1.16.3.3	0.000139417	0.000618783	0.00027292	5.91375E-06	0.000216062	0.002572021	0.314115018
5.1.99.3	2.54385E-05	9.39718E-05	2.84891E-05	0.000022388	0.682710228	0.793532671	-0.008607978
5.3.1.35	0.000100105	0.000572444	0.000182065	1.81445E-05	0.744889324	0.843948921	0.10763488
2.5.1.132	0.000311042	0.000520374	0.000240105	0.000381979	0.345834505	0.493481421	0.009033647
4.1.1.25	0.000272094	0.000839395	0.000328793	0.000215395	0.635281478	0.754179788	-0.067794347

EC	Mean abundance	Std_deviation	Mean abundance - health	Mean abundance - peri-implantitis	P value	FDR corrected P value	LDA correlation (CAP)
5.4.3.5	0.000972593	0.002800852	0.001662209	0.000282978	0.059194039	0.149751441	0.250603848
4.2.1.12	0.000402868	0.000958475	0.000315848	0.000489888	0.179741205	0.322384123	0.546274661
2.8.3.6	3.6613E-05	0.000111551	7.2626E-05	0.0000006	0.001629626	0.011420526	0.48107838
3.2.1.91	8.64574E-05	0.000178456	0.000125373	4.75415E-05	0.476306947	0.6257154	0.187363634
1.3.1.1	0.000744129	0.001785824	0.000820659	0.000667599	1	1	-0.099227764
2.3.3.15	0.002861383	0.006829383	0.002214092	0.003508673	0.876364829	0.93357138	-0.018783783
1.2.1.28	0	0	0	0	NA	NA	0
3.1.27.3	6.01923E-05	0.000152936	8.93717E-05	3.10128E-05	0.990683864	1	-0.027539012
3.2.1.83	0.000727305	0.001338566	0.001052361	0.00040225	0.002251412	0.014934937	0.502066671
3.5.2.17	0.00124739	0.005188067	0.002412693	8.20869E-05	0.085614357	0.19052209	0.216973941
3.2.1.8	0.000658354	0.001140755	0.000410736	0.000905971	0.16891629	0.308055102	-0.518517252
2.8.3.17	0.001656222	0.003058424	0.000902655	0.00240979	0.059063136	0.149751441	-0.543390978
1.1.1.132	0.00108516	0.004345526	0.001165174	0.001005146	0.59769524	0.731368732	0.283831904
1.8.5.5	0.00092988	0.001192524	0.000544167	0.001315593	0.010837031	0.044632131	-0.575866274
2.3.1.263	0.000487647	0.001636158	0.000823706	0.000151589	0.038134998	0.111768343	0.286086715
4.1.1.86	6.94791E-05	0.000118194	0.000108772	3.01865E-05	0.023656837	0.080936188	0.198732306
1.16.1.9	3.55236E-05	0.00014011	0.000069493	1.55417E-06	0.072772322	0.172897287	0.167572498
1.17.1.10	0.000316587	0.000430122	0.000284039	0.000349136	0.410710909	0.559416583	-0.166437403
4.4.1.15	0.00020201	0.000304485	0.000173763	0.000230258	0.752635928	0.845559951	-0.187989433
1.1.1.127	0.000485207	0.000665011	0.000264398	0.000706016	0.1195118	0.244654308	-0.306293593
4.1.1.85	0.001229591	0.002869738	0.000596595	0.001862588	0.542387706	0.684085511	0.041095938
3.2.1.37	0.000725511	0.001672755	0.000426118	0.001024904	0.439332478	0.587881323	-0.079253067
4.1.2.14	5.60399E-05	0.000234461	7.26868E-05	3.9393E-05	0.377427551	0.526037757	-0.133993466
6.5.1.4	6.92473E-06	3.20957E-05	1.25036E-05	1.34583E-06	0.572844873	0.706974319	0.136142838
1.2.1.46	0.001078828	0.002744692	0.001147934	0.001009723	0.45099111	0.599252713	-0.359969976
4.1.1.52	0.000325812	0.001270218	0.000590956	6.06686E-05	0.461928411	0.612405446	0.199492773
3.2.1.81	1.16557E-05	3.34513E-05	7.64971E-06	1.56616E-05	0.44586436	0.593802496	-0.176670798
1.14.14.47	0.0004584	0.001632693	0.000733147	0.000183654	0.347686283	0.495716784	0.064922747
1.14.19.1	0.00103579	0.002758858	0.000998936	0.001072645	0.242474953	0.405602953	0.164997613
2.4.1.212	0.000200047	0.000890027	0.000293092	0.000107002	0.20078043	0.351062763	-0.359247432
1.1.1.60	0.000150215	0.00021972	9.43935E-05	0.000206036	0.016818366	0.062860904	-0.511896685
2.7.1.89	9.68125E-06	2.04166E-05	1.07458E-05	8.61667E-06	0.535804634	0.679728799	-0.086277366
1.18.1.3	4.77307E-05	0.000118553	4.75484E-05	4.7913E-05	0.800063272	0.885111372	0.149968787
2.4.2.26	3.14202E-05	0.000115407	3.64023E-05	2.64381E-05	0.415952213	0.565668972	-0.036758596
1.17.1.5	5.63224E-05	0.000145623	1.9127E-05	9.35178E-05	0.223142972	0.380591252	-0.09494774
1.14.13.149	7.92584E-05	0.000280704	0.000147663	1.08542E-05	0.06500583	0.158457409	0.156318016
2.3.2.29	0.000116685	0.000176473	9.80603E-05	0.00013531	0.226203208	0.38505502	-0.372143132
3.1.2.23	5.83274E-05	0.000133573	8.30297E-05	3.36252E-05	0.420435818	0.56600887	0.31632981
3.4.24.78	8.36609E-05	0.000279635	0.00014004	2.72813E-05	0.333426187	0.489384158	0.099174744
1.1.1.36	7.21393E-05	0.000368759	0.000129831	1.44475E-05	0.049927218	0.133588508	0.24527396
4.2.1.149	1.79308E-05	6.7932E-05	3.237E-05	3.49167E-06	0.243717721	0.406592018	0.224604476
3.6.1.57	1.80461E-05	4.69789E-05	7.99625E-06	2.8096E-05	0.316826506	0.489384158	-0.182857793
1.1.1.264	0.000100982	0.000326647	0.000184527	1.74376E-05	0.011805487	0.047827359	0.249551279
2.6.1.106	1.18333E-06	6.80537E-06	0.00000195	4.16667E-07	1	1	0.017399355
5.1.1.8	2.96844E-06	1.71948E-05	0.000001	4.93688E-06	1	1	-0.101261113
4.1.1.45	4.98187E-05	0.000122193	2.75882E-05	7.20493E-05	0.086085648	0.191325903	-0.376448457
3.6.3.7	9.18252E-05	0.00014133	6.96209E-05	0.00011403	0.148513017	0.28584233	-0.315800353
1.14.12.1	3.30833E-06	1.16784E-05	0.00000545	1.16667E-06	0.282231305	0.455448476	0.048798883
1.1.1.103	0.000232776	0.001047104	0.000427018	3.85351E-05	0.078006621	0.181250679	0.436612483
2.3.1.59	0.000133402	0.000493389	7.95298E-05	0.000187275	0.508294592	0.651486726	0.028887657
2.1.1.178	0.000249374	0.000640913	0.00011765	0.000381099	0.496149062	0.643032863	-0.1567647
2.3.1.118	0.000142608	0.000346879	0.000222728	6.24879E-05	0.14846968	0.28584233	0.462681612
1.20.1.1	0.000134716	0.000281715	0.000258744	1.06875E-05	3.4357E-05	0.000796167	0.493145411
1.4.1.3	0.002139004	0.002382328	0.001541553	0.002736455	0.402589098	0.552249291	-0.324992688
4.2.1.40	0.000252237	0.000622398	0.00041779	0.000086684	0.077742425	0.181066372	0.217769121
3.4.24.40	0.000298305	0.000544176	0.000478867	0.000117743	0.007488266	0.034613315	0.260926591
2.5.1.141	0.001072496	0.001786329	0.001594031	0.000550961	0.000642701	0.005848243	0.512973622

EC	Mean abundance	Std_deviation	Mean abundance - health	Mean abundance - peri-implantitis	P value	FDR corrected P value	LDA correlation (CAP)
5.3.1.14	0.00161333	0.001437131	0.001050028	0.002176632	0.002936794	0.017909288	-0.620929223
1.8.5.3	0.000431031	0.000733123	0.000663952	0.00019811	0.002658173	0.01679965	0.406828042
3.4.23.49	0.000246758	0.000780921	0.000399561	9.39546E-05	0.004130665	0.023083911	0.556169973
2.3.2.27	3.80654E-06	1.70848E-05	6.01308E-06	0.0000016	0.9485568	0.980720832	0.148434653
4.4.1.1	2.08333E-07	1.42826E-06	0	4.16667E-07	0.3378947	0.489384158	-0.068439698
3.7.1.3	6.53556E-06	2.31022E-05	1.2122E-05	9.49167E-07	0.089252162	0.195611925	0.127256486
4.1.2.20	3.72272E-05	0.000127886	6.04677E-05	1.39867E-05	0.31039944	0.489384158	0.209312774
1.4.3.4	0.000168936	0.000452135	0.000279198	5.86729E-05	0.114244284	0.236095798	0.241897888
3.1.1.15	3.29792E-06	1.0644E-05	3.5125E-06	3.08333E-06	0.68304078	0.793532671	0.155796541
2.3.1.20	2.49366E-05	7.54313E-05	4.83358E-05	1.5375E-06	0.00043578	0.004254972	0.389773954
6.2.1.44	1.93703E-05	8.03526E-05	3.80656E-05	0.000000675	0.07868443	0.18185311	0.170878607
2.8.4.1	2.94226E-05	0.000193091	5.67244E-05	2.12083E-06	1	1	-0.278300822
1.5.98.1	0	0	0	0	NA	NA	0
1.10.3.16	2.59792E-06	8.92579E-06	1.50417E-06	3.69167E-06	0.322518942	0.489384158	-0.307625441
1.1.1.410	4.58783E-06	2.62741E-05	0	9.17567E-06	0.041041673	0.116743743	-0.034646075
1.1.1.130	0.000152531	0.000362944	0.00020284	0.000102222	0.567338827	0.706974319	0.156304402
2.7.1.55	0.000130572	0.000387728	0.00010716	0.000153983	0.758712589	0.851286301	0.153293186
2.1.4.1	0.001751832	0.002232306	0.00131508	0.002188584	0.546368678	0.686615157	-0.188884064
1.1.1.90	0.000146172	0.000685297	0.00021515	7.71935E-05	0.989182828	1	-0.043111053
1.14.14.9	0.000102601	0.000293856	9.85766E-05	0.000106625	0.193472747	0.339995586	0.106658591
5.1.3.26	0.000154115	0.000471051	2.87386E-05	0.000279492	0.053269602	0.140349256	-0.290955902
4.2.1.68	0	0	0	0	NA	NA	0
2.3.1.202	1.02044E-05	2.92332E-05	6.04163E-06	1.43671E-05	0.123429385	0.250607794	-0.237623478
5.5.1.1	1.33375E-05	4.78349E-05	2.49292E-05	1.74583E-06	0.013196063	0.051771461	0.258599902
1.2.1.39	5.90873E-05	0.000196536	0.000106423	1.17513E-05	0.019931867	0.070714227	0.311366186
2.8.3.24	0.002417104	0.004683826	0.001306345	0.003527864	0.034458726	0.104167016	-0.504290288
1.1.1.61	0.015970027	0.019400166	0.009315342	0.022624713	0.033682558	0.102522391	-0.583369518
2.7.8.44	0.000216352	0.000911978	0.000363995	6.87096E-05	0.376974183	0.525827552	0.050882889
2.4.2.43	0.000147105	0.000313894	0.000130725	0.000163484	0.240433965	0.403353506	-0.160342914
4.2.1.109	2.58568E-05	0.000102093	4.72927E-05	4.42083E-06	0.220084762	0.378807165	0.03251903
1.3.8.13	9.00546E-05	0.000179591	0.000107307	7.28025E-05	0.226982334	0.386003225	-0.109345567
6.2.1.13	0.002844196	0.00801359	0.002407575	0.003280816	0.069931624	0.169041951	-0.476192697
2.1.1.184	0.002652938	0.004525354	0.002689664	0.002616212	0.081412284	0.184237696	0.331234396
2.3.1.210	0.000181164	0.000604496	0.000321197	4.11307E-05	0.356305122	0.506343665	0.307232101
3.5.1.110	2.22451E-05	7.82498E-05	4.39653E-05	0.000000525	0.001629626	0.011420526	0.1175052
1.5.99.6	3.12685E-05	4.75079E-05	2.47296E-05	3.78074E-05	0.145013936	0.281917473	-0.046561906
3.4.17.18	0.000102494	0.000425022	3.64167E-06	0.000201347	0.04567182	0.125487655	-0.24984769
2.5.1.105	0.000145956	0.000207777	7.13014E-05	0.000220611	0.019143184	0.0685997	-0.565295673
2.3.1.5	5.04167E-07	3.45639E-06	1.00833E-06	0	0.3378947	0.489384158	0.131614803
2.7.1.206	0.000672525	0.001686408	0.000773377	0.000571673	0.857716109	0.923612514	-0.100269943
3.5.1.49	0.000118559	0.000474844	0.000225834	1.12838E-05	0.123700848	0.250865897	0.123019265
3.1.4.17	3.70702E-05	6.59973E-05	3.28428E-05	4.12976E-05	0.890100897	0.942715027	0.087293173
5.1.3.10	8.411E-05	0.000145326	7.5993E-05	9.2227E-05	0.691556892	0.801283919	0.04496487
1.1.1.202	0.002091979	0.011128241	0.003151479	0.001032479	0.918187388	0.960752366	-0.036569192
2.7.1.72	1.69899E-05	5.90416E-05	0.000010375	2.36049E-05	0.897830881	0.946862908	0.06692241
3.1.4.4	0	0	0	0	NA	NA	0
4.4.1.24	5.72439E-05	0.000140592	2.12075E-05	9.32803E-05	0.034811569	0.105039076	-0.360255249
1.3.99.8	0	0	0	0	NA	NA	0
1.8.1.14	0.000208309	0.000818228	0.000367559	4.90584E-05	0.17646346	0.319472388	0.285774752
3.1.21.1	2.28981E-05	0.000122054	4.52129E-05	5.83333E-07	0.0421536	0.119126758	0.117762606
1.1.1.35	0.000320069	0.001082524	0.000558035	8.21025E-05	0.004230617	0.023416602	0.529593778
2.1.3.6	6.42459E-05	0.000123822	8.20628E-05	4.64291E-05	0.473780347	0.624283732	-0.360995385
1.1.1.411	9.66415E-05	0.000377741	0.000156816	3.64669E-05	0.894460117	0.943880803	0.207619786
1.1.1.30	5.47448E-05	0.000181506	7.04319E-05	3.90578E-05	0.752563976	0.845559951	0.149497493
2.6.1.18	2.26337E-05	6.9327E-05	3.51173E-05	0.00001015	0.899714468	0.948273951	-0.001916647
3.4.21.19	7.09596E-05	0.000308378	0.000114529	2.73905E-05	0.181515366	0.324895681	0.094424358
1.2.1.71	2.94656E-05	7.54624E-05	3.65918E-05	2.23395E-05	0.860097162	0.924389386	0.220765706

EC	Mean abundance	Std_deviation	Mean abundance - health	Mean abundance - peri-implantitis	P value	FDR corrected P value	LDA correlation (CAP)
3.8.1.5	0.000145148	0.000555477	0.000217289	7.30077E-05	0.854730732	0.922111739	0.099789083
3.5.3.4	2.14389E-05	6.40738E-05	3.28161E-05	1.00617E-05	0.309490769	0.489384158	0.182704828
1.17.8.1	5.62279E-06	1.87899E-05	5.75808E-06	5.4875E-06	0.654727339	0.77042391	0.074315564
2.4.2.57	1.44792E-06	7.60978E-06	0	2.89583E-06	0.161691678	0.299914766	-0.068343414
1.13.11.8	3.77083E-07	2.58515E-06	7.54167E-07	0	0.3378947	0.489384158	0.036852145
1.17.4.2	0.002322561	0.01134795	0.001342463	0.003302659	0.339213183	0.490420812	0.036129675
2.5.1.97	2.08333E-07	1.42826E-06	0	4.16667E-07	0.3378947	0.489384158	-0.068439698
3.5.1.94	2.60423E-05	8.52086E-05	3.93069E-05	1.27777E-05	0.130946962	0.260693951	0.275627377
1.14.19.20	1.1387E-05	5.15787E-05	2.27739E-05	0	0.02067882	0.072168252	0.352947748
1.2.1.88	0.000560702	0.002380545	0.00104013	8.12742E-05	0.054740688	0.143498213	0.219516024
3.6.3.6	3.57447E-05	0.000212043	0.000062988	8.50133E-06	1	1	0.022304826
3.6.3.33	2.40076E-05	8.16068E-05	2.06461E-05	2.73692E-05	0.19119301	0.33838437	0.158648213
3.4.25.1	0.00010691	0.000312088	0.000200501	1.33189E-05	0.004978469	0.026219939	0.311028031
2.6.1.86	4.18445E-05	0.000178264	7.57981E-05	7.89083E-06	0.388162871	0.534993711	-0.322161565
3.2.1.136	5.99189E-05	0.000181634	0.000118492	1.34583E-06	0.002144171	0.014388301	0.391715888
6.3.2.38	1.79808E-05	7.33076E-05	3.11241E-05	4.8375E-06	0.148388649	0.28584233	0.093059441
1.1.1.47	0.00028246	0.000787126	0.000502316	6.26047E-05	0.020585785	0.072168252	0.318644687
1.2.1.99	0.000033152	0.000107338	3.95988E-05	2.67052E-05	0.971373961	0.997193116	0.213291263
3.4.11.19	0.000118415	0.000214426	0.00012639	0.00011044	0.599391347	0.731368732	0.030750846
2.6.1.113	1.42687E-05	3.21859E-05	1.19875E-05	0.00001655	0.320138176	0.489384158	-0.390995974
1.13.11.20	3.88588E-05	0.00024226	7.67333E-05	9.84167E-07	0.05451502	0.143122516	0.273861242
5.1.3.37	0.003025129	0.008087795	0.001332139	0.004718119	0.611157194	0.736609711	0.020855457
4.2.2.10	5.87913E-05	0.000250808	0.000112849	4.73396E-06	0.11588072	0.238626412	0.407939813
2.4.1.304	0.000270506	0.00055988	9.30809E-05	0.000447932	0.103482563	0.219600359	-0.302371929
1.14.99.44	0.000419674	0.001871569	0.00079735	4.19977E-05	0.105521781	0.221867402	0.166347793
1.1.1.281	4.90536E-05	0.000112282	3.16145E-05	6.64928E-05	0.309490769	0.489384158	0.01439973
2.1.1.230	2.56922E-05	6.79333E-05	2.00707E-05	3.13138E-05	0.15728925	0.299418091	0.017544691
2.3.1.28	0.002047735	0.005116237	0.003266168	0.000829302	0.00134265	0.009972334	0.577027471
2.5.1.140	0.000487313	0.001007681	0.000861294	0.000113331	1.83885E-05	0.000499363	0.542873176
4.1.1.77	3.80404E-06	2.03154E-05	1.80833E-06	5.79975E-06	0.605877247	0.731543474	0.032596289
1.2.1.59	4.47813E-05	8.52589E-05	2.61935E-05	6.33692E-05	0.033485326	0.102522391	-0.416499816
5.3.3.10	7.40817E-06	2.71277E-05	7.56667E-06	7.24967E-06	0.106347434	0.222420987	0.210646376
1.2.7.12	0.000194911	0.000365861	7.87E-05	0.000311122	0.020898554	0.072498376	-0.502165668
4.1.3.17	1.17523E-05	5.51092E-05	1.71753E-05	6.32917E-06	0.440392808	0.588771308	-0.132055441
1.14.15.7	4.87904E-05	0.000111164	5.73413E-05	4.02395E-05	0.094669044	0.205925907	0.323669696
3.1.11.3	4.77083E-06	1.41486E-05	8.72917E-06	8.125E-07	0.07868443	0.18185311	0.048458113
3.6.3.38	0.000787144	0.001322761	0.000852721	0.000721568	0.301444368	0.480651661	0.331520831
1.13.11.79	8.52773E-06	2.18508E-05	8.21667E-06	8.83879E-06	0.877588483	0.93357138	-0.015185741
1.14.13.40	3.09025E-05	0.000129059	5.90258E-05	2.77917E-06	0.340635772	0.490420812	-0.009280861
2.3.1.184	8.08956E-06	2.36859E-05	3.92917E-06	1.225E-05	0.053453355	0.140547551	-0.459733896
2.3.1.109	8.83523E-05	0.000368523	0.000143843	3.28615E-05	0.972443249	0.997701515	0.009638621
2.3.1.168	6.58333E-06	1.62044E-05	1.30833E-06	1.18583E-05	0.035801554	0.10613699	-0.482013308
2.4.2.6	0.000171511	0.000788631	0.0002784	6.46218E-05	0.345312092	0.493140851	0.195021041
4.2.1.137	9.55832E-05	0.000301805	0.000159469	3.16977E-05	0.553300309	0.694321977	0.182541823
1.14.15.3	1.68831E-05	3.9969E-05	2.32349E-05	1.05313E-05	0.757957011	0.850987911	-0.062731309
5.1.3.23	0.000246274	0.000358138	0.00012336	0.000369188	0.018483391	0.066786141	-0.460041555
2.1.1.181	4.2106E-05	0.000179039	8.02988E-05	3.91333E-06	0.085339084	0.190152984	0.113763308
2.4.1.33	3.38076E-05	8.79906E-05	2.89849E-05	3.86303E-05	0.61240231	0.737598902	-0.088202477
1.14.99.48	0.00012241	0.00050383	0.000241885	2.93417E-06	0.005919291	0.029309765	0.435607212
3.2.1.184	2.24639E-05	5.6744E-05	2.74069E-05	1.75208E-05	0.344030378	0.492119174	-0.046451098
1.1.5.2	0.000127762	0.000277798	0.000224529	3.09955E-05	0.014845391	0.056581776	0.334899389
4.2.1.103	1.36952E-05	3.4963E-05	7.5525E-06	1.98378E-05	0.014125176	0.054554569	-0.408646294
1.1.1.384	4.62933E-05	0.000153797	1.86043E-05	7.39822E-05	0.154573422	0.295395674	-0.279436709
3.5.1.54	2.03486E-05	7.4774E-05	1.31331E-05	2.75642E-05	0.325081038	0.489384158	0.011636712
3.5.2.9	1.12221E-05	2.63574E-05	5.85417E-06	1.65901E-05	0.154666319	0.295395674	-0.263853004
1.2.1.19	0.000084668	0.000263854	0.000134944	3.43918E-05	0.004960471	0.026219939	0.526426332
4.1.1.61	3.26175E-05	0.000185275	6.456E-05	0.000000675	0.53905226	0.68086688	0.092873553

EC	Mean abundance	Std_deviation	Mean abundance - health	Mean abundance - peri-implantitis	P value	FDR corrected P value	LDA correlation (CAP)
4.1.1.100	1.06769E-05	5.0293E-05	1.88163E-05	2.5375E-06	0.886340355	0.939304596	0.063293798
1.1.5.8	0	0	0	0	NA	NA	0
1.14.13.22	7.20406E-05	0.000202898	0.000111488	3.25933E-05	0.025348591	0.084560176	0.346362783
1.1.2.8	1.37215E-05	5.00746E-05	1.44992E-05	1.29438E-05	0.139672904	0.274605778	-0.334027373
4.2.1.96	2.64807E-05	6.48798E-05	2.30218E-05	2.99397E-05	0.831096351	0.906745422	0.103968691
1.10.3.3	1.1375E-06	7.79831E-06	0	0.000002275	0.3378947	0.489384158	-0.10002725
4.99.1.4	1.73355E-05	7.93801E-05	9.28233E-06	2.53888E-05	0.711807323	0.818201804	0.109118996
1.17.2.1	1.50625E-06	1.03263E-05	3.0125E-06	0	0.3378947	0.489384158	0.121085619
6.4.1.3	0.000195179	0.000355306	0.000148284	0.000242074	0.156861842	0.298931887	-0.137854522
2.3.1.60	3.17592E-06	1.66478E-05	0	6.35183E-06	0.161691678	0.299914766	-0.025080152
4.2.1.22	7.6875E-07	5.27028E-06	0	1.5375E-06	0.3378947	0.489384158	-0.057910513
1.13.11.1	2.49167E-06	8.58809E-06	3.44583E-06	1.5375E-06	0.344004795	0.492119174	0.063744249
1.1.98.3	0.000143753	0.000334339	0.000139128	0.000148377	0.87935097	0.934176031	0.146423943
1.2.1.26	1.05538E-05	5.00089E-05	1.88075E-05	0.0000023	0.853442914	0.921294276	-0.073380603
2.7.8.15	0	0	0	0	NA	NA	0
1.14.11.55	9.08333E-07	6.22722E-06	1.81667E-06	0	0.3378947	0.489384158	-0.047381329
3.5.3.26	0.000345631	0.000494948	0.000351839	0.000339422	0.90957008	0.956219866	-0.081648279
2.4.1.310	0	0	0	0	NA	NA	0
2.7.1.217	7.12585E-05	0.000360009	0.00011858	2.39366E-05	0.953675771	0.985427164	0.127453769
4.1.1.79	2.74779E-06	1.88379E-05	5.49558E-06	0	0.3378947	0.489384158	0.078968882
1.3.1.31	0.000171619	0.000451283	0.000298206	4.50315E-05	0.004975049	0.026219939	0.313197005
1.2.7.5	0.000337106	0.001095279	0.00019961	0.000474603	0.384833703	0.530826171	0.194577844
1.4.3.21	0.000114287	0.000310238	0.000136294	9.2281E-05	0.116747848	0.240127527	0.206974311
5.3.3.1	3.76948E-06	1.77325E-05	5.96813E-06	1.57083E-06	0.982841692	1	0.118770367
1.14.99.46	5.56681E-06	2.36328E-05	4.9875E-06	6.14613E-06	0.344004795	0.492119174	0.172890706
3.4.24.28	0.004598257	0.005500161	0.003076679	0.006119835	0.101121584	0.216463245	-0.546411902
1.8.98.3	0	0	0	0	NA	NA	0
3.1.1.61	2.45417E-06	1.01588E-05	0	4.90833E-06	0.041041673	0.116743743	-0.397297634
1.1.1.53	6.40665E-05	0.000306431	0.000126683	0.00000145	0.53905226	0.68086688	0.034407194
2.1.1.206	0	0	0	0	NA	NA	0
1.13.11.3	9.36463E-06	3.03474E-05	1.75251E-05	1.20417E-06	0.07868443	0.18185311	0.146035559
1.4.1.9	0.000289895	0.001110534	0.000546619	3.31701E-05	0.04040166	0.116447901	0.052982107
4.2.1.141	9.16667E-07	6.28435E-06	1.83333E-06	0	0.3378947	0.489384158	0.078968882
4.1.1.87	4.57312E-05	0.000142642	0.000049208	4.22544E-05	0.767755494	0.857557229	0.151887197
3.5.1.41	1.10688E-05	3.74255E-05	9.6405E-06	1.24971E-05	0.971373961	0.997193116	0.06564988
6.3.2.43	0.000321454	0.002003941	5.65388E-05	0.00058637	0.674606122	0.786603029	-0.021041667
5.3.3.18	7.90254E-05	0.000484397	0.000154806	0.000003245	0.177845297	0.321086004	0.13964054
2.3.1.222	0.001277867	0.00874961	0.002552589	3.14583E-06	0.605877247	0.731543474	-0.127798148
2.7.4.23	1.92458E-06	6.52566E-06	0	3.84917E-06	0.02067882	0.072168252	-0.280065266
3.4.22.38	0.002004135	0.003191993	0.00152631	0.00248196	0.210298606	0.364405759	-0.509423488
5.3.2.5	0.000151076	0.000346908	0.000165053	0.000137099	0.079179066	0.182753276	-0.208990092
5.2.1.4	8.34634E-05	0.000237487	0.000140455	2.64721E-05	0.173512593	0.315111562	0.221510612
1.1.1.203	4.35417E-07	2.98507E-06	8.70833E-07	0	0.3378947	0.489384158	0.226377461
1.3.1.25	4.83333E-06	1.44605E-05	7.65417E-06	2.0125E-06	0.341626883	0.491106305	0.067161244
1.6.3.3	1.02467E-05	7.02479E-05	2.04934E-05	0	0.3378947	0.489384158	-0.005264592
3.1.1.76	5.04167E-07	2.42212E-06	0	1.00833E-06	0.161691678	0.299914766	-0.292497271
1.14.12.19	1.01413E-05	4.75393E-05	1.60867E-05	4.19583E-06	0.484077306	0.631626395	0.107306854
3.4.11.14	0	0	0	0	NA	NA	0
3.2.1.185	9.15054E-05	0.000308493	2.24073E-05	0.000160604	0.44771328	0.595808332	-0.201922465
2.7.1.13	0	0	0	0	NA	NA	0
2.3.2.22	6.64894E-05	0.000312947	0.000132429	0.00000055	0.017206066	0.063355381	0.251753797
2.7.8.28	5.70627E-06	2.77715E-05	1.58333E-06	9.82921E-06	0.179721247	0.322384123	-0.128363247
2.4.1.332	0.00013515	0.000319581	0.000189409	8.08922E-05	0.20311821	0.354793415	0.223016819
5.1.3.25	0.000391389	0.001301255	0.000169285	0.000613494	1	1	-0.104746622
1.1.1.87	0	0	0	0	NA	NA	0
4.2.1.80	2.66622E-05	0.000122819	0.000049666	3.65833E-06	0.058432043	0.149456998	0.001912884
2.7.1.87	4.52653E-05	0.000157988	4.49273E-05	4.56033E-05	0.069257586	0.167645801	0.113202202

EC	Mean abundance	Std_deviation	Mean abundance - health	Mean abundance - peri-implantitis	P value	FDR corrected P value	LDA correlation (CAP)
2.7.8.39	2.19212E-05	6.57174E-05	4.20674E-05	0.000001775	0.004603188	0.025001065	0.22647578
4.2.1.82	0.000109773	0.000404359	0.000135318	8.42286E-05	0.593067314	0.726392524	0.288281563
4.1.2.21	5.57917E-06	1.75505E-05	4.92083E-06	6.2375E-06	0.740990061	0.840627106	-0.048110627
3.1.2.32	7.13123E-06	2.09172E-05	1.50417E-06	1.27583E-05	0.026185053	0.086192466	-0.465882483
1.14.13.59	0.000621419	0.002013395	0.000664941	0.000577897	0.842656023	0.915107845	-0.071552254
3.5.3.13	6.24583E-07	2.49387E-06	0.0000005	7.49167E-07	0.605877247	0.731543474	-0.214721584
4.2.3.153	1.92083E-07	1.31686E-06	0	3.84167E-07	0.3378947	0.489384158	0.142143987
2.4.1.9	0.000192346	0.000408661	8.48152E-05	0.000299876	0.056574511	0.145885016	-0.564045317
6.3.3.7	0	0	0	0	NA	NA	0
4.2.3.130	3.06697E-05	0.000129527	6.09553E-05	3.84167E-07	0.035801554	0.10613699	0.301656164
3.4.21.62	0.000287679	0.001127213	0.000534019	4.13385E-05	0.075467146	0.177006613	0.442031084
4.99.1.2	6.62544E-06	2.69979E-05	1.27009E-05	0.00000055	0.282231305	0.455448476	0.129186834
5.1.3.38	9.1625E-06	2.20243E-05	1.04333E-05	7.89167E-06	0.867735986	0.929670401	-0.273861242
3.11.1.1	1.00417E-06	5.41225E-06	2.00833E-06	0	0.161691678	0.299914766	0.121481986
3.3.2.13	2.66667E-07	1.82817E-06	0	5.33333E-07	0.3378947	0.489384158	-0.17373154
3.2.1.132	0.000159311	0.000431548	0.000256238	6.23848E-05	0.038716771	0.113282403	0.278764042
4.7.1.1	3.95833E-06	1.19872E-05	7.10417E-06	8.125E-07	0.07868443	0.18185311	0.155481585
4.4.1.19	4.12241E-05	0.000146891	8.24481E-05	0	0.002417912	0.015622046	0.339712945
5.3.1.32	3.85218E-05	0.000134342	6.12239E-05	1.58198E-05	0.363353466	0.513004325	-0.020945148
1.5.1.21	4.85565E-06	1.95215E-05	1.00833E-06	8.70296E-06	0.167432392	0.305669639	0.101748858
3.4.24.15	6.70774E-05	0.000185331	0.000118327	1.58277E-05	0.014706198	0.056174442	0.21076292
2.3.1.102	5.35417E-07	3.67063E-06	0	1.07083E-06	0.3378947	0.489384158	-0.17373154
2.3.1.187	0.000126716	0.000284501	0.000106104	0.000147328	0.823975447	0.901806881	-0.257458945
1.1.1.2	5.38585E-06	3.51318E-05	1.02467E-05	0.000000525	1	1	-0.147502643
1.13.11.11	2.48333E-06	1.03721E-05	3.87083E-06	1.09583E-06	0.53905226	0.68086688	-0.09183875
2.7.10.2	7.86346E-05	0.000309817	0.000143307	1.39618E-05	0.075307843	0.176871665	0.137893497
6.1.2.1	2.91667E-07	1.99957E-06	0	5.83333E-07	0.3378947	0.489384158	-0.24743583
1.13.11.33	1.52875E-06	5.81492E-06	2.30833E-06	7.49167E-07	0.9485568	0.980720832	-0.169494032
1.1.1.312	2.82083E-06	1.35652E-05	5.64167E-06	0	0.161691678	0.299914766	0.114428193
3.5.1.96	5.215E-05	0.000169231	9.03565E-05	1.39435E-05	0.02826504	0.091309738	0.40993508
3.6.3.36	0.000114743	0.000322882	0.000208192	2.12952E-05	0.221974362	0.379172858	0.417113782
2.7.1.162	0	0	0	0	NA	NA	0
2.5.1.108	0	0	0	0	NA	NA	0
2.7.1.192	3.52203E-05	0.000129093	2.71453E-05	4.32953E-05	0.546169769	0.686615157	0.321569281
1.14.11.45	1.34237E-05	6.24354E-05	2.68474E-05	0	0.02067882	0.072168252	0.303608764
1.14.16.1	4.42789E-05	0.000152588	5.34313E-05	3.51265E-05	0.532853819	0.676479136	-0.217125535
2.3.1.251	6.69087E-05	0.000192772	0.000126397	7.42083E-06	0.006388138	0.03084051	0.32254219
2.3.1.264	2.37739E-05	0.000126676	4.75479E-05	0	0.081078758	0.184237696	0.289227387
1.3.3.11	4.29542E-06	1.24797E-05	0.000005925	2.66583E-06	0.853442914	0.921294276	-0.19149685
3.4.24.25	1.81429E-05	3.70982E-05	6.54167E-06	2.97442E-05	0.007691626	0.034998504	-0.475430262
6.3.1.14	0	0	0	0	NA	NA	0
1.8.7.3	0	0	0	0	NA	NA	0
2.7.1.85	8.10206E-06	2.43494E-05	1.24166E-05	3.7875E-06	0.220229651	0.378807165	0.03147409
3.8.1.3	0	0	0	0	NA	NA	0
2.1.1.243	3.71458E-06	1.36973E-05	0.000002925	4.50417E-06	0.344004795	0.492119174	-0.046534434
3.5.1.102	0	0	0	0	NA	NA	0
4.2.1.90	6.82171E-05	0.00025289	0.000125463	1.09711E-05	0.006984909	0.032899111	0.307150198
2.5.1.89	2.27917E-06	1.39042E-05	4.03333E-06	0.000000525	1	1	-0.047652289
2.7.1.47	3.43856E-06	1.80113E-05	1.3625E-06	5.51463E-06	0.572000957	0.706974319	0.119261025
3.2.1.204	8.4206E-06	2.81136E-05	1.09738E-05	5.86738E-06	0.886340355	0.939304596	-0.214128686
1.4.99.5	2.33333E-06	8.31165E-06	0	4.66667E-06	0.041041673	0.116743743	-0.447908077
2.6.1.77	0.001484723	0.004856218	0.000353973	0.002615473	0.060819231	0.153639279	-0.359171121
1.5.99.15	0	0	0	0	NA	NA	0
3.4.21.110	0.001614335	0.002344253	0.002768816	0.000459854	0.000189085	0.002334886	0.493186402
1.14.11.33	3.00317E-05	0.000145372	5.06438E-05	9.4195E-06	0.650245177	0.767748722	0.035187325
1.1.1.374	0	0	0	0	NA	NA	0
2.1.1.98	0	0	0	0	NA	NA	0

EC	Mean abundance	Std_deviation	Mean abundance - health	Mean abundance - peri-implantitis	P value	FDR corrected P value	LDA correlation (CAP)
5.1.99.4	8.1955E-06	3.68782E-05	0.000016391	0	0.041041673	0.116743743	0.164512243
3.2.1.187	5.64188E-05	0.000382946	0.0000005	0.000112338	0.53905226	0.68086688	0.018626451
2.4.1.44	0.000208981	0.000510565	0.000328812	8.91504E-05	0.019075972	0.068527191	0.443466604
6.3.2.32	2.31896E-06	1.04935E-05	3.02125E-06	1.61667E-06	0.572000957	0.706974319	-0.082266824
1.8.7.1	2.66059E-05	8.25669E-05	3.53273E-05	1.78844E-05	0.589209293	0.722176129	0.14966433
1.4.99.6	2.81458E-06	8.32261E-06	3.26667E-06	2.3625E-06	0.971373961	0.997193116	-0.21555831
1.12.98.1	3.14346E-06	1.59465E-05	0	6.28692E-06	0.081078758	0.184237696	-0.236711144
1.8.5.6	9.16667E-07	6.28435E-06	1.83333E-06	0	0.3378947	0.489384158	0.078968882
3.1.1.57	7.97042E-06	4.93985E-05	1.59408E-05	0	0.161691678	0.299914766	0.284816475
1.14.14.35	2.45658E-05	6.92475E-05	3.61461E-05	1.29855E-05	0.129775075	0.26012274	0.15965474
1.1.1.320	2.81451E-05	0.000139558	5.62902E-05	0	0.161691678	0.299914766	0.196722441
3.4.24.3	0.000340886	0.000635326	0.000196416	0.000485355	0.064783393	0.158457409	-0.092851654
1.1.5.9	6.54167E-07	4.48474E-06	1.30833E-06	0	0.3378947	0.489384158	0.026322961
2.7.1.14	1.01884E-05	3.15267E-05	8.45417E-06	1.19227E-05	0.749670045	0.845559951	-0.257450982
4.2.2.23	3.75993E-05	9.72635E-05	5.5505E-05	1.96937E-05	0.911934721	0.957090909	-0.131137012
4.3.99.4	0.000312928	0.000783484	9.93917E-05	0.000526465	0.00080989	0.006702805	-0.610867946
3.2.1.123	0.000163709	0.000351734	0.000240805	8.66116E-05	0.00329512	0.019680135	0.436340632
4.2.1.114	0	0	0	0	NA	NA	0
1.5.1.33	6.35833E-06	1.73189E-05	0	1.27167E-05	0.002417912	0.015622046	-0.513373735
1.3.99.16	7.16029E-06	1.96609E-05	9.64142E-06	4.67917E-06	0.915595853	0.959049686	-0.266783243
1.2.1.68	5.41784E-05	0.00017653	9.36945E-05	1.46623E-05	0.021431053	0.07419755	0.378286369
4.1.1.101	0.000274436	0.000687673	0.000168892	0.000379981	0.70232627	0.811389693	-0.338850041
3.5.4.32	3.13064E-05	9.24887E-05	3.30833E-06	5.93045E-05	0.005791741	0.029176944	-0.457365595
2.3.1.18	0.000264343	0.001038796	4.20633E-05	0.000486623	0.877496177	0.93357138	-0.190516501
3.13.1.1	1.46454E-05	7.18045E-05	1.76913E-05	1.15995E-05	1	1	0.115682201
2.1.1.67	1.24505E-05	4.06142E-05	1.35716E-05	1.13294E-05	0.971373961	0.997193116	-0.078779856
3.4.23.52	0	0	0	0	NA	NA	0
6.2.1.19	0	0	0	0	NA	NA	0
1.3.99.4	3.39583E-07	2.32807E-06	6.79167E-07	0	0.3378947	0.489384158	0.152673171
1.13.11.24	8.83081E-05	0.000296077	0.000162758	1.38586E-05	0.361971144	0.511996853	0.120996032
3.5.2.14	2.90821E-06	1.59987E-05	5.81642E-06	0	0.161691678	0.299914766	0.168193769
2.3.1.178	1.23211E-05	6.09216E-05	2.21913E-05	2.45083E-06	0.391102978	0.538618839	0.270482741
4.1.1.7	0	0	0	0	NA	NA	0
1.1.1.108	6.19965E-05	0.000194933	0.000116426	7.56667E-06	0.475149908	0.625140455	0.100550247
5.1.3.40	7.60417E-07	5.21315E-06	1.52083E-06	0	0.3378947	0.489384158	0.184260724
4.2.2.5	2.63382E-05	9.66811E-05	1.25967E-05	4.00798E-05	0.674606122	0.786603029	-0.183716738
1.8.98.5	5.47917E-07	3.75633E-06	0	1.09583E-06	0.3378947	0.489384158	-0.184260724
1.14.13.182	3.375E-07	2.31378E-06	0	0.000000675	0.3378947	0.489384158	-0.194789908
2.7.1.101	9.66523E-06	3.53615E-05	9.01613E-06	1.03143E-05	0.057741754	0.14845439	0.046680672
2.5.1.77	0	0	0	0	NA	NA	0
1.2.7.7	0	0	0	0	NA	NA	0
1.14.13.82	3.77083E-07	2.58515E-06	7.54167E-07	0	0.3378947	0.489384158	0.036852145
3.5.4.39	0	0	0	0	NA	NA	0
4.1.1.75	0	0	0	0	NA	NA	0
1.5.98.2	0.000000275	1.88531E-06	0	0.00000055	0.3378947	0.489384158	-0.184260724
3.2.1.82	4.9439E-05	9.94359E-05	7.61116E-05	2.27663E-05	0.419069484	0.565922893	0.045333495
4.2.1.104	2.6807E-05	8.39675E-05	5.2939E-05	0.000000675	0.00367754	0.021024884	0.372116074
4.2.1.41	0.000115922	0.000431671	0.000212925	1.89185E-05	0.006523503	0.031320022	0.364414696
2.4.99.18	1.78156E-05	4.8295E-05	4.42542E-06	3.12058E-05	0.044798244	0.124774597	-0.459446768
4.1.1.4	0.00011518	0.000436984	0.000227193	3.16667E-06	0.089252162	0.195611925	0.149580718
2.5.1.44	3.79583E-06	1.02914E-05	0	7.59167E-06	0.010294231	0.043215876	-0.513485965
2.7.13.1	0.000008761	2.57152E-05	1.06374E-05	6.88458E-06	0.26052237	0.427561737	0.121475841
4.1.1.9	1.92294E-05	7.21908E-05	2.53815E-05	1.30772E-05	0.654727339	0.77042391	0.189576342
1.8.99.5	0.003180068	0.005241532	0.00153329	0.004826846	0.195479628	0.342829055	-0.35324622
5.1.99.7	1.60657E-05	4.76254E-05	1.89079E-05	1.32235E-05	0.415952213	0.565668972	0.12669225
2.4.1.19	8.45407E-05	0.0002029	1.14487E-05	0.000157633	0.00165569	0.011556582	-0.546479696
5.3.99.10	0.000121332	0.000410281	0.000232415	1.02495E-05	0.111358358	0.23095564	0.283022562

EC	Mean abundance	Std_deviation	Mean abundance - health	Mean abundance - peri-implantitis	P value	FDR corrected P value	LDA correlation (CAP)
3.2.1.73	1.05833E-06	4.17067E-06	0	2.11667E-06	0.081078758	0.184237696	-0.216015088
4.1.2.52	7.93255E-05	0.000235456	4.23817E-05	0.000116269	0.232531062	0.392749257	-0.275591427
2.7.1.20	6.25686E-05	0.000216762	7.08435E-05	5.42937E-05	0.083032862	0.187173947	-0.349942155
5.99.1.4	2.91667E-07	1.99957E-06	0	5.83333E-07	0.3378947	0.489384158	-0.24743583
2.4.1.268	0.000423448	0.002788285	0.000834685	1.22107E-05	0.379300746	0.527801998	-0.030510663
3.5.1.107	7.91667E-07	5.42739E-06	1.58333E-06	0	0.3378947	0.489384158	0.236906645
2.4.1.338	4.72917E-07	3.24215E-06	0	9.45833E-07	0.3378947	0.489384158	-0.236906645
1.14.13.20	1.32454E-05	7.22435E-05	2.64908E-05	0	0.041041673	0.116743743	0.165531246
2.7.7.91	3.07806E-05	0.000194257	3.6625E-06	5.78987E-05	0.572000957	0.706974319	-0.084853831
3.1.1.17	7.90013E-06	2.18111E-05	5.66708E-06	1.01332E-05	0.811848786	0.893434284	-0.217318275
2.4.1.56	1.61042E-06	7.73946E-06	1.50833E-06	1.7125E-06	1	1	0.004075525
2.7.1.188	1.4625E-06	1.00264E-05	0.000002925	0	0.3378947	0.489384158	0.089498066
1.17.99.1	0.000001275	8.74096E-06	0	0.00000255	0.3378947	0.489384158	0.110556435
1.4.1.25	0.000000275	1.88531E-06	0	0.00000055	0.3378947	0.489384158	-0.152673171
4.2.2.17	0.000011085	3.25468E-05	0.000002555	0.000019615	0.324784491	0.489384158	-0.240800709
1.13.11.5	6.56667E-06	1.66935E-05	1.58333E-06	0.00001155	0.0421536	0.119126758	-0.389536066
1.3.7.8	1.11107E-05	4.94098E-05	0	2.22214E-05	0.02067882	0.072168252	-0.319782124
4.4.1.31	1.1375E-06	7.79831E-06	0	0.000002275	0.3378947	0.489384158	-0.10002725
2.4.1.213	0	0	0	0	NA	NA	0
1.3.8.8	8.71313E-06	2.53569E-05	1.28167E-05	4.60958E-06	0.057741754	0.14845439	0.125542718
6.3.4.23	0	0	0	0	NA	NA	0
6.3.2.49	9.84468E-05	0.000276267	0.000170842	2.60521E-05	0.034115244	0.103476952	0.404640231
1.14.13.208	1.71458E-06	1.03945E-05	3.0125E-06	4.16667E-07	1	1	0.040441745
1.5.1.24	0	0	0	0	NA	NA	0
4.2.1.118	7.6875E-07	5.27028E-06	0	1.5375E-06	0.3378947	0.489384158	-0.057910513
1.1.1.342	1.32708E-06	6.23069E-06	0	2.65417E-06	0.081078758	0.184237696	-0.227656619
4.2.1.83	3.39583E-07	2.32807E-06	6.79167E-07	0	0.3378947	0.489384158	0.152673171
3.5.1.26	6.54167E-07	4.48474E-06	1.30833E-06	0	0.3378947	0.489384158	0.026322961
2.3.1.262	3.77708E-06	1.24691E-05	0	7.55417E-06	0.02067882	0.072168252	-0.471074691
3.5.1.106	0	0	0	0	NA	NA	0
1.13.11.15	4.84292E-06	2.07867E-05	8.13583E-06	0.00000155	0.885863346	0.939304596	-0.018986512
4.1.2.42	1.2108E-05	4.73376E-05	4.43333E-06	1.97828E-05	0.626901968	0.750119378	0.037260145
3.1.3.87	2.34909E-05	0.000121698	3.99952E-05	6.98667E-06	0.567494145	0.706974319	-0.110366992
5.1.1.17	1.5625E-06	7.18722E-06	0.00000245	0.000000675	0.572000957	0.706974319	-0.03311369
1.14.13.2	9.08333E-07	6.22722E-06	1.81667E-06	0	0.3378947	0.489384158	-0.047381329
1.1.3.48	0.000106016	0.000285423	1.85332E-05	0.000193499	0.041017386	0.116743743	-0.408044555
2.3.1.194	0	0	0	0	NA	NA	0
1.14.19.49	3.85417E-06	1.60177E-05	3.92917E-06	3.77917E-06	0.605877247	0.731543474	-0.238522049
1.14.19.9	8.99485E-06	3.38822E-05	1.36399E-05	4.34979E-06	0.098796683	0.213260825	0.210740836
5.5.1.27	5.79771E-05	0.000309726	0.000108534	7.42058E-06	0.650245177	0.767748722	-0.016621149
1.14.12.7	0	0	0	0	NA	NA	0
4.1.1.18	8.14375E-06	2.09762E-05	4.11667E-06	1.21708E-05	0.131356584	0.261087772	-0.277840541
2.6.1.82	2.43708E-06	1.67078E-05	4.87417E-06	0	0.3378947	0.489384158	0.089498066
1.14.13.231	2.54366E-05	0.000143748	5.08733E-05	0	0.041041673	0.116743743	0.325174923
1.2.1.22	0	0	0	0	NA	NA	0
2.1.3.9	5.67265E-06	3.12718E-05	1.13453E-05	0	0.081078758	0.184237696	0.263357317
3.1.2.1	8.58685E-05	0.00038206	0.000104841	0.000066896	0.41942732	0.565966369	0.068389227
4.3.1.30	0	0	0	0	NA	NA	0
3.6.1.15	0	0	0	0	NA	NA	0
1.14.13.113	1.80954E-05	7.25384E-05	3.01366E-05	6.05417E-06	0.181515366	0.324895681	0.172716295
1.14.13.142	1.69167E-06	9.29274E-06	2.61667E-06	7.66667E-07	1	1	0.118660469
3.7.1.18	0	0	0	0	NA	NA	0
2.7.7.62	0	0	0	0	NA	NA	0
2.5.1.41	0	0	0	0	NA	NA	0
2.7.7.68	6.14475E-06	2.23432E-05	0.000003805	8.4845E-06	0.484676702	0.631934064	-0.197322803
1.2.99.7	0.000238494	0.000577785	7.75474E-05	0.00039944	0.010176102	0.04303179	-0.363932759
5.3.1.29	3.89583E-07	2.67085E-06	0	7.79167E-07	0.3378947	0.489384158	-0.142143987

EC	Mean abundance	Std_deviation	Mean abundance - health	Mean abundance - peri-implantitis	P value	FDR corrected P value	LDA correlation (CAP)
1.3.3.6	7.91667E-07	5.42739E-06	1.58333E-06	0	0.3378947	0.489384158	0.236906645
4.1.1.97	4.57953E-05	0.000211249	9.17921E-06	8.24113E-05	0.019448687	0.069266021	-0.340275991
3.5.3.3	5.04167E-07	3.45639E-06	1.00833E-06	0	0.3378947	0.489384158	0.131614803
1.13.11.4	0.000000275	1.88531E-06	0	0.00000055	0.3378947	0.489384158	-0.152673171
6.2.1.31	9.9375E-07	5.00884E-06	1.9875E-06	0	0.161691678	0.299914766	0.126027763
1.14.13.1	9.72654E-06	3.68518E-05	1.34887E-05	5.96442E-06	0.9485568	0.980720832	0.114241468
4.2.1.153	4.2899E-05	0.000157689	8.08773E-05	4.92083E-06	0.00816984	0.036374662	0.205017299
3.13.1.4	7.74915E-06	4.04134E-05	4.50808E-06	1.09902E-05	1	1	0.324160963
6.4.1.5	1.82883E-05	7.40549E-05	2.84912E-05	8.08546E-06	0.310416155	0.489384158	0.198302319
1.13.11.18	0	0	0	0	NA	NA	0
1.3.1.104	7.34152E-05	0.000226859	0.00010971	3.71207E-05	0.14686981	0.284246208	0.366372574
2.1.1.209	2.33567E-05	8.16651E-05	5.51667E-06	4.11967E-05	0.360154597	0.510145631	-0.273179067
1.1.1.51	0.00000145	9.9407E-06	0	0.0000029	0.3378947	0.489384158	-0.015793776
2.7.7.97	2.10704E-06	1.44451E-05	0	4.21408E-06	0.3378947	0.489384158	0.068439698
2.3.1.101	1.37083E-07	9.39796E-07	0	2.74167E-07	0.3378947	0.489384158	-0.184260724
4.2.1.81	1.08958E-06	6.0308E-06	0	2.17917E-06	0.161691678	0.299914766	-0.323220457
3.5.4.29	0	0	0	0	NA	NA	0
1.3.8.14	0.000000975	6.68426E-06	0.00000195	0	0.3378947	0.489384158	0.089498066
6.2.1.8	0	0	0	0	NA	NA	0
4.6.1.16	0	0	0	0	NA	NA	0
3.4.24.75	7.44021E-06	3.44414E-05	7.53425E-06	7.34617E-06	0.605877247	0.731543474	-0.151081211
4.2.2.6	2.68292E-05	0.000121484	2.69793E-05	2.66791E-05	0.461946801	0.612405446	-0.013035516
4.2.1.57	0.000000275	1.88531E-06	0	0.00000055	0.3378947	0.489384158	-0.152673171
1.1.1.404	0	0	0	0	NA	NA	0
5.1.2.7	0	0	0	0	NA	NA	0
2.8.1.15	0.000000625	4.28478E-06	0.00000125	0	0.3378947	0.489384158	0.057910513
2.7.4.26	2.54898E-06	1.74749E-05	0	5.09796E-06	0.3378947	0.489384158	0.110556435
1.12.1.2	9.60417E-07	6.58428E-06	0	1.92083E-06	0.3378947	0.489384158	0.142143987
1.1.1.9	2.18844E-06	1.50032E-05	4.37688E-06	0	0.3378947	0.489384158	0.015793776
1.4.1.24	1.39192E-05	5.58382E-05	7.20392E-06	2.06345E-05	0.971373961	0.997193116	-0.106362251
2.4.2.54	0	0	0	0	NA	NA	0
2.3.3.14	5.70833E-07	3.91344E-06	0	1.14167E-06	0.3378947	0.489384158	-0.110556435
5.3.3.4	2.57708E-06	1.3701E-05	3.92917E-06	0.000001225	0.605877247	0.731543474	-0.184971003
2.8.3.22	0.000000275	1.88531E-06	0	0.00000055	0.3378947	0.489384158	-0.152673171
1.2.1.50	0	0	0	0	NA	NA	0
1.1.2.4	0	0	0	0	NA	NA	0
1.3.7.9	4.35417E-07	2.98507E-06	8.70833E-07	0	0.3378947	0.489384158	0.226377461
4.1.3.32	1.60208E-06	7.81693E-06	1.30833E-06	1.89583E-06	1	1	-0.154399685
1.14.13.235	1.1375E-06	7.79831E-06	0	0.000002275	0.3378947	0.489384158	-0.10002725
6.2.1.46	0.00000025	1.71391E-06	0.0000005	0	0.3378947	0.489384158	0.047381329
1.6.2.2	3.39583E-07	2.32807E-06	6.79167E-07	0	0.3378947	0.489384158	0.152673171
1.14.13.218	3.38063E-06	1.75359E-05	6.76125E-06	0	0.161691678	0.299914766	0.264909104
1.7.1.6	1.7091E-05	7.34693E-05	3.24779E-05	1.70417E-06	0.04567182	0.125487655	0.098312325
3.5.4.27	0	0	0	0	NA	NA	0
3.1.3.92	4.55535E-06	2.22855E-05	6.02738E-06	3.08333E-06	0.605877247	0.731543474	0.041392113
1.3.99.5	2.50067E-05	0.0001678	4.89633E-05	0.00000105	1	1	-0.139821847
2.7.7.15	5.3696E-05	0.00023175	1.98166E-05	8.75755E-05	0.811848786	0.893434284	-0.205965828
2.4.2.48	0	0	0	0	NA	NA	0
1.12.2.1	0.000000625	4.28478E-06	0	0.00000125	0.3378947	0.489384158	-0.068439698
2.1.1.302	3.33558E-05	0.00016023	6.67115E-05	0	0.081078758	0.184237696	0.022506961
3.5.1.68	1.5125E-06	1.03692E-05	0.000003025	0	0.3378947	0.489384158	0.131614803
3.6.1.17	0	0	0	0	NA	NA	0
3.2.2.26	1.52968E-05	4.58631E-05	0.000002625	2.79686E-05	0.016646719	0.062353445	-0.282336837
2.1.1.327	8.33333E-07	4.31728E-06	0.0000005	1.16667E-06	1	1	-0.147345892
1.14.13.148	7.60417E-07	5.21315E-06	1.52083E-06	0	0.3378947	0.489384158	0.184260724
3.1.3.103	0	0	0	0	NA	NA	0
4.2.1.25	0	0	0	0	NA	NA	0

EC	Mean abundance	Std_deviation	Mean abundance - health	Mean abundance - peri-implantitis	P value	FDR corrected P value	LDA correlation (CAP)
4.2.1.148	2.25404E-06	1.54529E-05	4.50808E-06	0	0.3378947	0.489384158	0.205319093
2.3.1.230	8.71875E-06	2.16417E-05	2.8125E-06	0.000014625	0.066968649	0.163013322	-0.404096768
1.1.1.337	0	0	0	0	NA	NA	0
2.1.1.213	0	0	0	0	NA	NA	0
3.1.3.77	1.74375E-06	1.04196E-05	3.0125E-06	0.000000475	1	1	-0.077434969
6.1.1.23	0	0	0	0	NA	NA	0
1.14.99.50	4.90926E-05	0.000120094	1.17774E-05	8.64078E-05	0.000420513	0.004217336	-0.581156281
6.3.2.39	2.72728E-05	0.000123996	5.00793E-05	4.46625E-06	0.373797556	0.521815383	0.196904747
3.5.4.43	1.24286E-05	5.58661E-05	1.52083E-06	2.33364E-05	0.026185053	0.086192466	-0.235198748
4.1.2.28	0	0	0	0	NA	NA	0
6.3.4.24	0	0	0	0	NA	NA	0
1.3.4.1	0	0	0	0	NA	NA	0
2.5.1.114	0	0	0	0	NA	NA	0
2.5.1.131	0	0	0	0	NA	NA	0
1.1.1.193	2.74779E-06	1.88379E-05	5.49558E-06	0	0.3378947	0.489384158	0.078968882
3.5.2.19	0	0	0	0	NA	NA	0
4.2.1.159	4.26667E-06	1.74338E-05	6.86667E-06	1.66667E-06	0.53905226	0.68086688	-0.093908356
1.5.3.22	0	0	0	0	NA	NA	0
4.2.1.157	0.00021542	0.00063075	0.000190303	0.000240537	0.822792348	0.901647604	-0.140235398
2.6.1.97	0	0	0	0	NA	NA	0
1.12.98.2	0	0	0	0	NA	NA	0
1.14.15.13	4.78746E-06	2.80217E-05	9.57492E-06	0	0.161691678	0.299914766	0.180733845
2.1.1.248	3.92171E-05	0.000127505	3.06731E-05	4.77611E-05	0.090208957	0.197459908	-0.298702053
2.3.1.174	2.8715E-06	1.9686E-05	0.000005743	0	0.3378947	0.489384158	0.163202356
5.3.1.31	0.000152249	0.000572038	0.000256858	4.764E-05	0.071103867	0.169285646	0.386444923
1.13.11.78	3.20833E-07	2.19952E-06	6.41667E-07	0	0.3378947	0.489384158	0.194789908
4.1.1.41	4.56654E-06	1.8992E-05	8.50392E-06	6.29167E-07	0.282231305	0.455448476	0.201649212
4.1.3.16	5.83375E-06	1.46627E-05	1.97083E-06	9.69667E-06	0.035220366	0.105854462	-0.336487125
4.3.1.27	0	0	0	0	NA	NA	0
2.5.1.128	0	0	0	0	NA	NA	0
6.3.5.7	0	0	0	0	NA	NA	0
2.4.1.266	2.87026E-05	0.000196775	5.74053E-05	0	0.3378947	0.489384158	0.005264592
2.3.1.37	5.04167E-07	3.45639E-06	1.00833E-06	0	0.3378947	0.489384158	0.131614803
1.13.11.16	5.04167E-07	3.45639E-06	1.00833E-06	0	0.3378947	0.489384158	0.131614803
3.5.1.100	9.01615E-06	6.18116E-05	1.80323E-05	0	0.3378947	0.489384158	0.205319093
1.3.1.64	0	0	0	0	NA	NA	0
1.13.11.41	0.0000003	2.0567E-06	0	0.0000006	0.3378947	0.489384158	0.068439698
2.7.1.161	0	0	0	0	NA	NA	0
5.2.1.1	0	0	0	0	NA	NA	0
5.4.1.4	0	0	0	0	NA	NA	0
2.7.7.40	1.89583E-06	9.19267E-06	2.98417E-06	8.075E-07	0.982841692	1	-0.227463935
2.1.1.21	2.87083E-06	1.40176E-05	0	5.74167E-06	0.161691678	0.299914766	-0.330587752
1.14.13.7	9.39583E-07	4.51865E-06	1.87917E-06	0	0.161691678	0.299914766	0.254406791
2.7.8.34	1.24578E-05	5.09326E-05	0	2.49156E-05	0.005039521	0.026393688	-0.367433475
6.4.1.9	0	0	0	0	NA	NA	0
4.2.2.7	0	0	0	0	NA	NA	0
3.4.21.71	6.7621E-06	4.63587E-05	1.35242E-05	0	0.3378947	0.489384158	0.205319093
6.3.2.36	0	0	0	0	NA	NA	0
3.4.21.81	1.12141E-05	7.17458E-05	2.24283E-05	0	0.161691678	0.299914766	0.201895223
2.5.1.43	0	0	0	0	NA	NA	0
4.2.1.30	6.8125E-07	4.67041E-06	1.3625E-06	0	0.3378947	0.489384158	0.152673171
2.7.1.91	0	0	0	0	NA	NA	0
3.5.4.10	0	0	0	0	NA	NA	0
1.14.11.38	7.91667E-07	5.42739E-06	1.58333E-06	0	0.3378947	0.489384158	0.236906645
3.4.15.1	2.37229E-06	1.62636E-05	4.74458E-06	0	0.3378947	0.489384158	0.236906645
1.1.1.56	3.39583E-07	2.32807E-06	6.79167E-07	0	0.3378947	0.489384158	0.152673171
1.17.98.2	0	0	0	0	NA	NA	0

G. Metatranscriptome-based class level composition

Class	Mean abundance	Std_deviation	Mean abundance - health	Mean abundance - peri-implantitis	P value	FDR corrected P value
Actinobacteria	5.187252427	5.702561569	6.743957167	3.630547688	0.027147143	0.129161707
Alphaproteobacteria	0.000377612	0.000803922	0.000479772	0.000275453	0.029354933	0.129161707
Anaerolineae	0.046375467	0.094753015	0.020110093	0.07264084	0.048279687	0.151736159
Bacilli	26.03809246	20.37947657	38.24562821	13.83055671	2.51875E-06	5.54126E-05
Bacteroidia	28.56941518	18.25386596	18.54700367	38.59182669	7.10844E-05	0.000781928
Betaproteobacteria	2.743085495	3.897979578	2.737837177	2.748333813	0.17185877	0.33603354
Chlamydiia	0.000169703	0.000510201	0.000302323	3.70818E-05	0.064783393	0.173456923
Clostridia	2.726670624	2.660045625	2.29810052	3.155240727	0.110554262	0.243219377
Coriobacteriia	0.655040932	1.242829902	0.310556962	0.999524903	0.214346279	0.362739856
Deltaproteobacteria	0.108243862	0.217371863	0.041576178	0.174911546	0.045920684	0.151736159
Epsilonproteobacteria	0.887294875	0.876408207	0.780375679	0.994214072	0.373099055	0.456009957
Erysipelotrichia	0.181764228	0.23862743	0.200187157	0.1633413	0.782704652	0.836467794
Flavobacteriia	2.573674121	3.688207529	2.298096733	2.849251509	0.79844653	0.836467794
Fusobacteriia	16.71751063	8.172603175	14.37468264	19.06033862	0.07095965	0.173456923
Gammaproteobacteria	3.431468673	6.855694249	4.965341804	1.897595542	0.014428574	0.105809541
Methanobacteria	0.000864296	0.001972061	0.001355065	0.000373526	0.183291022	0.33603354
Mollicutes	0.085479485	0.199194664	0.065172889	0.105786081	0.467672658	0.541515709
Negativicutes	7.533680617	6.532579521	7.361520118	7.705841116	0.91055459	0.91055459
Spirochaetia	1.935960639	3.546971857	0.700556047	3.171365231	0.362155525	0.456009957
SR1 [C-1]	0.010824439	0.043301393	0.002441193	0.019207686	0.367589119	0.456009957
Synergistia	0.272043887	0.457228804	0.165014907	0.379072868	0.291171026	0.456009957
TM7 [C-1]	0.294710342	0.828439598	0.139703691	0.449716993	0.351411522	0.456009957

H. Curated amino acid metabolism dataset

Class_Group	Group	Class	Mean abundance	Mean abundance - health	Mean abundance - peri-implantitis	P value	FDR corrected P value
Actinomycetia_Arg_a	Arg_a	Actinomycetia	17.80507468	19.53616374	16.07398561	0.281836841	0.400026483
Actinomycetia_Arg_u	Arg_u	Actinomycetia	75.3415874	110.8282395	39.85493526	0.008824822	0.053212279
Actinomycetia_Cys_a	Cys_a	Actinomycetia	49.46448595	75.82535637	23.10361552	0.007223852	0.048744432
Actinomycetia_Cys_u	Cys_u	Actinomycetia	0.937144438	1.437726625	0.43656225	0.332478478	0.452444939
Actinomycetia_His_a	His_a	Actinomycetia	18.44387011	22.11280833	14.77493189	0.147193088	0.271741086
Actinomycetia_His_u	His_u	Actinomycetia	3.29674014	2.240519925	4.352960354	0.612270771	0.701053422
Actinomycetia_Lys_a	Lys_a	Actinomycetia	118.1590672	164.4029263	71.91520802	0.008777645	0.053212279
Actinomycetia_Met_a	Met_a	Actinomycetia	59.9897199	91.1211881	28.85825169	0.035442905	0.126677697
Actinomycetia_Met_u	Met_u	Actinomycetia	29.46309545	46.60922945	12.31696145	0.022979765	0.097849321
Actinomycetia_phe_a	phe_a	Actinomycetia	3.240497298	3.633996017	2.846998579	0.256308442	0.375919048
Actinomycetia_Pro_a	Pro_a	Actinomycetia	7.489616175	9.29212805	5.6871043	0.094872447	0.217794139
Actinomycetia_Pro_u	Pro_u	Actinomycetia	0.034503908	0.047862817	0.021145	0.440392808	0.54892402
Actinomycetia_Thr_a	Thr_a	Actinomycetia	50.73359348	75.90243741	25.56474956	0.022979765	0.097849321
Actinomycetia_Thr_u	Thr_u	Actinomycetia	290.8361297	340.7544608	240.9177987	0.050737544	0.157584842
Actinomycetia_Trp_a	Trp_a	Actinomycetia	64.2806322	87.42205425	41.13921015	0.035508142	0.126677697
Actinomycetia_Tyr_a	Tyr_a	Actinomycetia	26.12845757	37.94674245	14.31017269	0.009969192	0.053711563
AlphaProteobacteria_Arg_a	Arg_a	AlphaProteobac	0.16491771	0.212348213	0.117487208	0.806299636	0.861793943
AlphaProteobacteria_Arg_u	Arg_u	AlphaProteobac	0.199007292	0.398014583	0	0.081078758	0.202824453
AlphaProteobacteria_Lys_a	Lys_a	AlphaProteobac	0.092514617	0.167488542	0.017540692	0.110150879	0.231108113
AlphaProteobacteria_Thr_u	Thr_u	AlphaProteobac	0.281523198	0.353445004	0.209601392	0.080712774	0.202824453
Anaerolineae_Arg_a	Arg_a	Anaerolineae	12.94976978	4.547562663	21.35197689	0.004877841	0.034804053
Anaerolineae_Arg_u	Arg_u	Anaerolineae	1.62047836	0.676573542	2.564383179	0.00080491	0.008854006
Anaerolineae_Cys_a	Cys_a	Anaerolineae	0.397263254	0.144885292	0.649641217	0.001645465	0.014480092
Anaerolineae_His_a	His_a	Anaerolineae	0.422396565	0.002740379	0.84205275	0.001629626	0.014480092
Anaerolineae_Lys_a	Lys_a	Anaerolineae	1.112297165	0.190901546	2.033692783	0.001219251	0.01192157
Anaerolineae_Met_a	Met_a	Anaerolineae	0.229682788	0.068542167	0.390823408	0.005441311	0.037802791
Anaerolineae_Pro_a	Pro_a	Anaerolineae	0.622222292	0.412619767	0.831824817	0.008868713	0.053212279
Anaerolineae_Thr_a	Thr_a	Anaerolineae	1.188837606	0.276174708	2.101500504	0.000535556	0.006732705
Anaerolineae_Thr_u	Thr_u	Anaerolineae	2.221354817	0.560368254	3.882341379	0.000372479	0.005175491
Anaerolineae_Trp_a	Trp_a	Anaerolineae	0.112490975	0.008221125	0.216760825	0.010509994	0.053897446
Anaerolineae_Tyr_a	Tyr_a	Anaerolineae	0.668469413	0.264480458	1.072458367	0.027422865	0.10969146
Bacilli_Arg_a	Arg_a	Bacilli	79.9735141	116.4746018	43.47242645	0.000129181	0.002131482
Bacilli_Arg_u	Arg_u	Bacilli	850.4760286	1277.984708	422.9673496	0.000660648	0.007583086
Bacilli_Cys_a	Cys_a	Bacilli	283.3677055	454.1514099	112.584001	6.09E-06	0.000178655
Bacilli_Cys_u	Cys_u	Bacilli	1.404676917	2.745123833	0.06423	0.155785777	0.276023122
Bacilli_His_a	His_a	Bacilli	26.18651788	46.5208348	5.85220095	0.000511256	0.006732705
Bacilli_His_u	His_u	Bacilli	134.9561212	201.1180026	68.79423971	0.013583315	0.066407319
Bacilli_Lys_a	Lys_a	Bacilli	495.5521536	746.6849894	244.4193178	4.17E-07	5.51E-05
Bacilli_Lys_u	Lys_u	Bacilli	111.0347868	181.2480352	40.82153843	1	1
Bacilli_Met_a	Met_a	Bacilli	859.9605461	1393.861471	326.0596216	3.80E-05	0.000771375
Bacilli_Met_u	Met_u	Bacilli	171.2628594	286.6042327	55.92148622	0.000999669	0.010150485
Bacilli_phe_a	phe_a	Bacilli	38.85465496	60.38867667	17.32063325	8.55E-07	5.65E-05
Bacilli_Pro_a	Pro_a	Bacilli	126.7958427	192.6307428	60.96094267	1.48E-06	6.52E-05
Bacilli_Pro_u	Pro_u	Bacilli	45.00719529	44.93628183	45.07810875	0.427228726	0.537087541
Bacilli_Thr_a	Thr_a	Bacilli	574.9762315	876.0310189	273.9214441	2.87E-06	9.46E-05
Bacilli_Thr_u	Thr_u	Bacilli	3450.312346	4990.353437	1910.271255	2.87E-06	9.46E-05
Bacilli_Trp_a	Trp_a	Bacilli	371.4566443	621.2162862	121.6970024	1.13E-06	5.96E-05
Bacilli_Tyr_a	Tyr_a	Bacilli	201.7613588	305.2530573	98.26966028	4.17E-07	5.51E-05
Bacteroidales_Arg_u	Arg_u	Bacteroidales	5.312079633	4.409251958	6.214907308	0.172526683	0.299432162
Bacteroidales_Cys_a	Cys_a	Bacteroidales	3.452459513	2.70276875	4.202150275	0.032286895	0.121767719
Bacteroidales_His_a	His_a	Bacteroidales	0.683074138	0.619115542	0.747032733	0.038613719	0.134131867
Bacteroidales_His_u	His_u	Bacteroidales	19.6492302	15.68291792	23.61554249	0.145420322	0.271741086
Bacteroidales_Lys_a	Lys_a	Bacteroidales	9.873816569	8.856149254	10.89148388	0.304504372	0.423100812
Bacteroidales_Lys_u	Lys_u	Bacteroidales	12.14256528	10.927876	13.35725455	0.268397015	0.391474099
Bacteroidales_Thr_u	Thr_u	Bacteroidales	10.77104377	7.800577875	13.74150967	0.119170542	0.245789242
Bacteroidales_Trp_a	Trp_a	Bacteroidales	0.260242404	0.161559083	0.358925725	0.098457983	0.220278877

Class_Group	Group	Class	Mean abundance	Mean abundance - health	Mean abundance - peri-implantitis	P value	FDR corrected P value
Bacteroidales_Tyr_a	Tyr_a	Bacteroidales	3.392181183	2.714239333	4.070123033	0.34793865	0.465125143
Bacteroidia_Arg_a	Arg_a	Bacteroidia	28.75425743	13.82203033	43.68648453	0.002535725	0.020919729
Bacteroidia_Arg_u	Arg_u	Bacteroidia	196.3014878	110.5197838	282.0831919	0.000142315	0.002210061
Bacteroidia_Cys_a	Cys_a	Bacteroidia	94.37224798	102.7922846	85.95221141	0.035508142	0.126677697
Bacteroidia_His_a	His_a	Bacteroidia	4.221618642	3.240991325	5.202245958	0.045430196	0.148068786
Bacteroidia_His_u	His_u	Bacteroidia	509.392409	227.2042655	791.5805524	7.43E-07	5.65E-05
Bacteroidia_Lys_a	Lys_a	Bacteroidia	546.25057	331.1189715	761.3821685	2.46E-05	0.000540864
Bacteroidia_Lys_u	Lys_u	Bacteroidia	305.3254394	185.7927489	424.8581299	0.009356868	0.053711563
Bacteroidia_Met_a	Met_a	Bacteroidia	101.2977182	61.18408349	141.411353	1.57E-05	0.000414009
Bacteroidia_Met_u	Met_u	Bacteroidia	50.41326093	36.58173774	64.24478412	0.004823307	0.034804053
Bacteroidia_Pro_a	Pro_a	Bacteroidia	6.124299075	3.336715083	8.911883067	0.0096207	0.053711563
Bacteroidia_Pro_u	Pro_u	Bacteroidia	4.413709167	2.666374333	6.161044	0.035247518	0.126677697
Bacteroidia_Thr_a	Thr_a	Bacteroidia	4.059017079	3.056219125	5.061815033	0.031162802	0.120984997
Bacteroidia_Thr_u	Thr_u	Bacteroidia	1512.649108	857.1365369	2168.161678	1.97E-05	0.000472123
Bacteroidia_Trp_a	Trp_a	Bacteroidia	56.13559347	36.64926669	75.62192025	4.23E-05	0.000796823
Bacteroidia_Trp_u	Trp_u	Bacteroidia	107.5447533	38.26858158	176.8209251	0.003912486	0.031299887
Bacteroidia_Tyr_a	Tyr_a	Bacteroidia	124.5834037	69.72875417	179.4380532	5.79E-05	0.001018637
BetaProteobacteria_Arg_a	Arg_a	BetaProteobact	28.47437202	29.53670356	27.41204049	0.081437091	0.202824453
BetaProteobacteria_Arg_u	Arg_u	BetaProteobact	11.22786396	11.73688623	10.71884168	0.223331652	0.351443953
BetaProteobacteria_Cys_a	Cys_a	BetaProteobact	18.74422764	16.5007528	20.98770248	0.223646152	0.351443953
BetaProteobacteria_His_a	His_a	BetaProteobact	12.38460588	14.47892478	10.29028698	0.06799539	0.186987323
BetaProteobacteria_Lys_a	Lys_a	BetaProteobact	67.05496744	58.94063718	75.16929769	0.278964122	0.400026483
BetaProteobacteria_Lys_u	Lys_u	BetaProteobact	2.558644079	3.287606563	1.829681596	0.076129118	0.198990962
BetaProteobacteria_Met_a	Met_a	BetaProteobact	61.07519482	56.15627938	65.99411026	0.135903357	0.26188676
BetaProteobacteria_Met_u	Met_u	BetaProteobact	0.796121154	0.864763079	0.727479229	0.107987615	0.231108113
BetaProteobacteria_phe_a	phe_a	BetaProteobact	0.053534231	0.027563417	0.079505046	0.053453355	0.15975379
BetaProteobacteria_Pro_a	Pro_a	BetaProteobact	5.314529369	5.648000863	4.981057875	0.183291022	0.308209107
BetaProteobacteria_Pro_u	Pro_u	BetaProteobact	1.495556398	1.926994417	1.064118379	0.14421664	0.271741086
BetaProteobacteria_Thr_a	Thr_a	BetaProteobact	27.65003439	29.05550295	26.24456583	0.096901932	0.21933264
BetaProteobacteria_Thr_u	Thr_u	BetaProteobact	124.6459101	110.0281709	139.2636494	0.093046618	0.215476379
BetaProteobacteria_Trp_a	Trp_a	BetaProteobact	22.10708945	21.15712678	23.05705211	0.147193088	0.271741086
BetaProteobacteria_Tyr_a	Tyr_a	BetaProteobact	9.214517221	9.896386167	8.532648275	0.235514085	0.361486735
BetaProteobacteria_Tyr_u	Tyr_u	BetaProteobact	0.152327869	0.293066663	0.011589075	0.108699762	0.231108113
Clostridia_Arg_a	Arg_a	Clostridia	26.41698825	38.69466683	14.13930967	0.764926752	0.831031533
Clostridia_Arg_u	Arg_u	Clostridia	40.14800105	37.86867287	42.42732924	0.153095027	0.273088427
Clostridia_Cys_a	Cys_a	Clostridia	13.53593777	10.66082249	16.41105304	0.085107982	0.206133094
Clostridia_Cys_u	Cys_u	Clostridia	0.14370815	0.257628333	0.029787967	0.057741754	0.165693727
Clostridia_His_a	His_a	Clostridia	1.71397856	0.630269929	2.797687192	0.090762593	0.215476379
Clostridia_His_u	His_u	Clostridia	10.72566171	5.5209935	15.93032993	0.108443812	0.231108113
Clostridia_Lys_a	Lys_a	Clostridia	54.26590289	43.43550675	65.09629903	0.048279687	0.154004245
Clostridia_Lys_u	Lys_u	Clostridia	55.11555674	61.65329785	48.57781564	0.630998004	0.714950528
Clostridia_Met_a	Met_a	Clostridia	14.05406298	10.56850092	17.53962503	0.246506703	0.369760055
Clostridia_Met_u	Met_u	Clostridia	11.51472201	9.602506675	13.42693735	0.060594982	0.172011563
Clostridia_Pro_a	Pro_a	Clostridia	7.645123194	6.22514175	9.065104638	0.010541814	0.053897446
Clostridia_Pro_u	Pro_u	Clostridia	20.24177058	19.08940117	21.39413999	0.069331203	0.188695232
Clostridia_Thr_a	Thr_a	Clostridia	19.76068254	16.1710416	23.35032348	0.127035843	0.254071687
Clostridia_Thr_u	Thr_u	Clostridia	70.817971	51.1186861	90.5172559	0.008777645	0.053212279
Clostridia_Trp_a	Trp_a	Clostridia	4.387535785	3.760132658	5.014938913	0.598469965	0.692965223
Clostridia_Trp_u	Trp_u	Clostridia	3.486034521	2.91597125	4.056097792	0.550640923	0.657779202
Clostridia_Tyr_a	Tyr_a	Clostridia	41.05061091	33.85176373	48.24945808	0.120214723	0.245881869
Coriobacteriia_Arg_a	Arg_a	Coriobacteriia	8.589425206	5.415785133	11.76306528	0.157705813	0.277562231
Coriobacteriia_Arg_u	Arg_u	Coriobacteriia	145.7609752	61.8940642	229.6278863	0.016256115	0.076942437
Coriobacteriia_Cys_a	Cys_a	Coriobacteriia	0.967825758	0.595558329	1.340093188	0.479924552	0.581702505
Coriobacteriia_His_a	His_a	Coriobacteriia	2.431305273	0.787712371	4.074898175	0.251220884	0.371280987
Coriobacteriia_His_u	His_u	Coriobacteriia	16.21559669	8.2921605	24.13903288	0.352367533	0.465125143
Coriobacteriia_Lys_a	Lys_a	Coriobacteriia	35.93539804	17.73619102	54.13460505	0.173534549	0.299432162
Coriobacteriia_Lys_u	Lys_u	Coriobacteriia	11.06179367	3.291126167	18.83246117	0.857716109	0.898559733

Class_Group	Group	Class	Mean abundance	Mean abundance - health	Mean abundance - peri-implantitis	P value	FDR corrected P value
Coriobacteriia_Met_a	Met_a	Coriobacteriia	17.31032632	5.205065338	29.41558731	0.189995174	0.315463685
Coriobacteriia_Met_u	Met_u	Coriobacteriia	6.796765304	3.435716967	10.15781364	0.197249217	0.323439709
Coriobacteriia_Pro_a	Pro_a	Coriobacteriia	1.808045377	0.715438021	2.900652733	0.29921705	0.421740766
Coriobacteriia_Thr_a	Thr_a	Coriobacteriia	6.700266671	3.034450517	10.36608283	0.048418001	0.154004245
Coriobacteriia_Thr_u	Thr_u	Coriobacteriia	45.18604633	24.30782615	66.0642665	0.153095027	0.273088427
Coriobacteriia_Trp_a	Trp_a	Coriobacteriia	1.869711248	0.663735942	3.075686554	0.460876197	0.565913098
Coriobacteriia_Tyr_a	Tyr_a	Coriobacteriia	5.784564438	2.4130872	9.156041675	0.092315455	0.215476379
DeltaProteobacteria_Arg_a	Arg_a	DeltaProteobact	5.459735196	1.362261863	9.557208529	0.024734002	0.102027759
DeltaProteobacteria_Arg_u	Arg_u	DeltaProteobact	5.219333833	1.795776542	8.642891125	0.030409148	0.119821121
DeltaProteobacteria_His_a	His_a	DeltaProteobact	1.25412164	0.194248904	2.313994375	0.001409172	0.013286476
DeltaProteobacteria_Lys_a	Lys_a	DeltaProteobact	0.66098405	0.116236708	1.205731392	0.000286652	0.004204227
DeltaProteobacteria_Met_a	Met_a	DeltaProteobact	3.631259369	2.682104696	4.580414042	0.176426896	0.302446107
DeltaProteobacteria_Pro_u	Pro_u	DeltaProteobact	0.193350919	0.050065363	0.336636475	0.018685406	0.085050814
DeltaProteobacteria_Thr_a	Thr_a	DeltaProteobact	1.882423658	0.455998792	3.308848525	0.011594353	0.057753001
DeltaProteobacteria_Thr_u	Thr_u	DeltaProteobact	2.197289773	0.763686613	3.630892933	0.016321123	0.076942437
DeltaProteobacteria_Trp_a	Trp_a	DeltaProteobact	0.311476533	0.048979375	0.573973692	0.004620412	0.034804053
DeltaProteobacteria_Tyr_a	Tyr_a	DeltaProteobact	1.532724331	1.227174863	1.8382738	0.051819207	0.159072916
Desulfovibrionia_Met_a	Met_a	Desulfovibrionia	0.013406021	0	0.026812042	0.081078758	0.202824453
EpsilonProteobacteria_Arg_a	Arg_a	EpsilonProteoba	15.17274831	13.78391965	16.56157698	0.093046618	0.215476379
EpsilonProteobacteria_Arg_u	Arg_u	EpsilonProteoba	7.554381833	6.794875292	8.313888375	0.231707369	0.357723657
EpsilonProteobacteria_Cys_a	Cys_a	EpsilonProteoba	8.765381729	10.38469758	7.146065875	0.153095027	0.273088427
EpsilonProteobacteria_His_a	His_a	EpsilonProteoba	9.880128031	8.666033063	11.094223	0.30070683	0.421740766
EpsilonProteobacteria_His_u	His_u	EpsilonProteoba	0.106712879	0.051467875	0.161957883	0.172206073	0.299432162
EpsilonProteobacteria_Lys_a	Lys_a	EpsilonProteoba	27.53942606	24.6400864	30.43876571	0.11530651	0.239692273
EpsilonProteobacteria_Met_a	Met_a	EpsilonProteoba	17.42051031	16.61277308	18.22824754	0.351411522	0.465125143
EpsilonProteobacteria_Met_u	Met_u	EpsilonProteoba	6.868742083	6.810080542	6.927403625	0.613421744	0.701053422
EpsilonProteobacteria_phe_a	phe_a	EpsilonProteoba	0.139594792	0.158424504	0.120765079	0.476740591	0.581702505
EpsilonProteobacteria_Pro_a	Pro_a	EpsilonProteoba	1.227063794	0.917485538	1.53664205	0.109558256	0.231108113
EpsilonProteobacteria_Thr_a	Thr_a	EpsilonProteoba	8.581944646	8.09252025	9.071369042	0.373099055	0.485212565
EpsilonProteobacteria_Thr_u	Thr_u	EpsilonProteoba	35.41045233	32.40012363	38.42078104	0.135903357	0.26188676
EpsilonProteobacteria_Trp_a	Trp_a	EpsilonProteoba	8.788573042	8.409985125	9.167160958	0.14318437	0.271741086
EpsilonProteobacteria_Tyr_a	Tyr_a	EpsilonProteoba	5.84044199	5.469949313	6.210934667	0.702851617	0.76954273
Erysipelotrichia_Arg_a	Arg_a	Erysipelotrichia	0.140996754	0.262664625	0.019328883	0.184615205	0.308470976
Erysipelotrichia_Arg_u	Arg_u	Erysipelotrichia	25.18151813	17.143881	33.21915527	0.415245542	0.52968542
Erysipelotrichia_Cys_a	Cys_a	Erysipelotrichia	1.492801715	2.353332479	0.63227095	0.421486749	0.533884964
Erysipelotrichia_His_a	His_a	Erysipelotrichia	0.328844444	0.578814321	0.078874567	0.363683895	0.47767437
Erysipelotrichia_Lys_a	Lys_a	Erysipelotrichia	1.085696665	1.488795021	0.682598308	0.674324168	0.745900145
Erysipelotrichia_Met_a	Met_a	Erysipelotrichia	0.605671917	0.606461233	0.6048826	0.128879634	0.255821227
Erysipelotrichia_Met_u	Met_u	Erysipelotrichia	0.084649671	0.16210975	0.007189592	0.323720698	0.444670435
Erysipelotrichia_Pro_a	Pro_a	Erysipelotrichia	0.156853417	0.28114195	0.032564883	0.579454317	0.679893065
Erysipelotrichia_Thr_a	Thr_a	Erysipelotrichia	0.864061429	1.379341567	0.348781292	0.956427206	0.967420622
Erysipelotrichia_Thr_u	Thr_u	Erysipelotrichia	10.30422414	11.38862629	9.219821992	0.570582118	0.672582723
Erysipelotrichia_Tyr_a	Tyr_a	Erysipelotrichia	0.664407769	0.607123954	0.721691583	0.152285714	0.273088427
Flavobacteriia_Arg_a	Arg_a	Flavobacteriia	2.586642583	2.014524667	3.1587605	0.966204917	0.973580527
Flavobacteriia_Arg_u	Arg_u	Flavobacteriia	45.12194166	44.12277638	46.12110695	0.606199966	0.698850615
Flavobacteriia_Cys_a	Cys_a	Flavobacteriia	36.71066375	52.28981125	21.13151625	0.934203743	0.952238564
Flavobacteriia_Cys_u	Cys_u	Flavobacteriia	2.501662602	1.914022083	3.089303121	0.640687917	0.721959207
Flavobacteriia_His_u	His_u	Flavobacteriia	6.551035213	4.972314125	8.1297563	0.77976889	0.843684373
Flavobacteriia_Lys_a	Lys_a	Flavobacteriia	53.78100425	44.52187784	63.04013067	0.909697266	0.938125306
Flavobacteriia_Met_a	Met_a	Flavobacteriia	57.72294812	49.36964633	66.07624991	0.868974131	0.904702061
Flavobacteriia_Met_u	Met_u	Flavobacteriia	6.788994769	5.39325125	8.184738288	0.850945858	0.895018751
Flavobacteriia_Pro_u	Pro_u	Flavobacteriia	12.73966148	9.782974583	15.69634838	0.796413989	0.856869447
Flavobacteriia_Thr_a	Thr_a	Flavobacteriia	3.70641574	3.443083104	3.969748375	0.42265893	0.533884964
Flavobacteriia_Thr_u	Thr_u	Flavobacteriia	53.69435341	50.76319611	56.62551071	0.836633031	0.883484481
Flavobacteriia_Trp_a	Trp_a	Flavobacteriia	12.58981062	10.81770275	14.36191849	0.62780045	0.714393615
Flavobacteriia_Tyr_a	Tyr_a	Flavobacteriia	15.05103671	13.9606796	16.14139381	0.926063634	0.948072943
Fusobacteriia_Arg_a	Arg_a	Fusobacteriia	35.05259458	21.7506906	48.35449856	0.570676249	0.672582723

Class_Group	Group	Class	Mean abundance	Mean abundance - health	Mean abundance - peri-implantitis	P value	FDR corrected P value
Fusobacteriia_Arg_u	Arg_u	Fusobacteriia	648.8529767	597.334016	700.3719374	0.272703911	0.395570509
Fusobacteriia_Cys_a	Cys_a	Fusobacteriia	208.866814	158.5139272	259.2197008	0.07095965	0.189653769
Fusobacteriia_Cys_u	Cys_u	Fusobacteriia	10.21647905	10.30835921	10.1245989	0.123270213	0.248422415
Fusobacteriia_His_a	His_a	Fusobacteriia	23.48444345	14.77527921	32.1936077	0.642653082	0.721959207
Fusobacteriia_His_u	His_u	Fusobacteriia	1073.944644	853.5744013	1294.314886	0.00419749	0.032592276
Fusobacteriia_Lys_a	Lys_a	Fusobacteriia	134.8953356	120.4048671	149.3858041	0.587992654	0.686858675
Fusobacteriia_Lys_u	Lys_u	Fusobacteriia	2325.607234	1667.178407	2984.036061	0.009969192	0.053711563
Fusobacteriia_Met_a	Met_a	Fusobacteriia	222.5140599	133.1490948	311.879025	0.894378126	0.925944413
Fusobacteriia_Met_u	Met_u	Fusobacteriia	97.23764708	68.88389333	125.5914008	0.009969192	0.053711563
Fusobacteriia_Pro_a	Pro_a	Fusobacteriia	13.09358164	9.521577671	16.6655856	0.414662067	0.52968542
Fusobacteriia_Thr_a	Thr_a	Fusobacteriia	45.02101514	43.1610177	46.88101258	0.6603774	0.736422912
Fusobacteriia_Thr_u	Thr_u	Fusobacteriia	1317.782625	1014.075443	1621.489808	0.097204238	0.21933264
Fusobacteriia_Trp_a	Trp_a	Fusobacteriia	34.67813527	31.95468775	37.4015828	0.705414169	0.76954273
Fusobacteriia_Trp_u	Trp_u	Fusobacteriia	371.0068394	218.4321413	523.5815375	0.000999669	0.010150485
Fusobacteriia_Tyr_a	Tyr_a	Fusobacteriia	162.9117571	145.1987922	180.624722	0.310444458	0.429096004
GammaProteobacteria_Arg_a	Arg_a	GammaProteob	27.21548495	39.77236417	14.65860573	0.02051487	0.090265426
GammaProteobacteria_Arg_u	Arg_u	GammaProteob	12.54663772	16.81569025	8.277585188	0.053856391	0.15975379
GammaProteobacteria_Cys_a	Cys_a	GammaProteob	21.13039635	27.83534793	14.42544478	0.002045983	0.017423857
GammaProteobacteria_His_a	His_a	GammaProteob	12.54721388	21.82155769	3.272870079	0.054927189	0.161119755
GammaProteobacteria_His_u	His_u	GammaProteob	0.518164788	1.004630333	0.031699242	0.000614172	0.00737006
GammaProteobacteria_Lys_a	Lys_a	GammaProteob	102.4527921	148.4675775	56.43800671	0.02051487	0.090265426
GammaProteobacteria_Lys_u	Lys_u	GammaProteob	0.063412275	0.076004458	0.050820092	0.820351346	0.869770102
GammaProteobacteria_Met_a	Met_a	GammaProteob	147.1703217	230.9057037	63.43493973	0.010616164	0.053897446
GammaProteobacteria_Met_u	Met_u	GammaProteob	6.849002854	8.951589542	4.746416167	0.105193106	0.231108113
GammaProteobacteria_phe_a	phe_a	GammaProteob	0.219432563	0.127614042	0.311251083	0.181515366	0.30717985
GammaProteobacteria_phe_u	phe_u	GammaProteob	0.031895396	0.043976708	0.019814083	0.9485568	0.963149982
GammaProteobacteria_Pro_a	Pro_a	GammaProteob	12.83578689	19.00886807	6.662705708	0.042229004	0.142928936
GammaProteobacteria_Pro_u	Pro_u	GammaProteob	0.018422698	0.036845396	0	0.081078758	0.202824453
GammaProteobacteria_Thr_a	Thr_a	GammaProteob	29.42028421	44.11130298	14.72926544	0.040197747	0.137820847
GammaProteobacteria_Thr_u	Thr_u	GammaProteob	238.7708083	339.2247255	138.316891	0.024303877	0.10184482
GammaProteobacteria_Trp_a	Trp_a	GammaProteob	26.87084819	36.88487253	16.85682385	0.031936275	0.121767719
GammaProteobacteria_Trp_u	Trp_u	GammaProteob	0.248268325	0.090021708	0.406514942	0.325081038	0.444670435
GammaProteobacteria_Tyr_a	Tyr_a	GammaProteob	17.21324087	25.08183649	9.344645246	0.071120164	0.189653769
GammaProteobacteria_Tyr_u	Tyr_u	GammaProteob	0.298982681	0.128806779	0.469158583	0.220084762	0.35001432
Mollicutes_Arg_u	Arg_u	Mollicutes	28.15668423	19.95762288	36.35574558	0.203708787	0.331969875
Mollicutes_Thr_u	Thr_u	Mollicutes	0.575429871	0.386073417	0.764786325	0.103496571	0.229605839
Negativicutes_Arg_a	Arg_a	Negativicutes	200.3701559	231.7856787	168.954633	0.281836841	0.400026483
Negativicutes_Arg_u	Arg_u	Negativicutes	244.5258246	311.307652	177.7439972	0.238172101	0.363103746
Negativicutes_Cys_a	Cys_a	Negativicutes	70.62956178	88.90776202	52.35136154	0.055961569	0.162350047
Negativicutes_Cys_u	Cys_u	Negativicutes	0.465471721	0.213922296	0.717021146	0.089662835	0.215190805
Negativicutes_His_a	His_a	Negativicutes	35.4254712	37.79024907	33.06069333	0.814269221	0.866802719
Negativicutes_Lys_a	Lys_a	Negativicutes	225.1615832	243.1470054	207.176161	0.675265662	0.745900145
Negativicutes_Lys_u	Lys_u	Negativicutes	107.3599285	68.35636773	146.3634893	0.130510373	0.256875098
Negativicutes_Met_a	Met_a	Negativicutes	160.3726043	212.2876352	108.4575734	0.053297186	0.15975379
Negativicutes_Met_u	Met_u	Negativicutes	83.61762485	111.5945917	55.64065801	0.025692206	0.104349884
Negativicutes_phe_a	phe_a	Negativicutes	2.667652265	0.739042488	4.596262042	0.073389634	0.193748634
Negativicutes_Pro_a	Pro_a	Negativicutes	83.11201723	95.37615978	70.84787468	0.44288188	0.54892402
Negativicutes_Pro_u	Pro_u	Negativicutes	29.87168445	20.41880577	39.32456314	0.415321522	0.52968542
Negativicutes_Thr_a	Thr_a	Negativicutes	90.0209584	95.28808759	84.75382922	0.705414169	0.76954273
Negativicutes_Thr_u	Thr_u	Negativicutes	412.5771999	473.7758031	351.3785968	0.48034525	0.581702505
Negativicutes_Trp_a	Trp_a	Negativicutes	54.02405337	47.00214983	61.0459569	0.79844653	0.856869447
Negativicutes_Tyr_a	Tyr_a	Negativicutes	50.77793005	50.84518077	50.71067933	0.44288188	0.54892402
Saccharimonadia_Arg_u	Arg_u	Saccharimonad	0.566006427	0.398761913	0.733250942	0.661106933	0.736422912
Saccharimonadia_Cys_u	Cys_u	Saccharimonad	1.175150542	0.924118875	1.426182208	0.373024084	0.485212565
Saccharimonadia_Lys_a	Lys_a	Saccharimonad	1.878710127	2.456272125	1.301148129	0.593067314	0.689734674
Saccharimonadia_Thr_u	Thr_u	Saccharimonad	0.00985575	0.014949292	0.004762208	1	1
Saccharimonadia_Trp_a	Trp_a	Saccharimonad	0.071967729	0.08587625	0.058059208	0.344004795	0.463826967

Class_Group	Group	Class	Mean abundance	Mean abundance - health	Mean abundance - peri-implantitis	P value	FDR corrected P value
Spirochaetia_Arg_a	Arg_a	Spirochaetia	3.128715621	1.747094371	4.510336871	0.142033079	0.271715456
Spirochaetia_Arg_u	Arg_u	Spirochaetia	180.561291	57.05996254	304.0626194	0.56008305	0.666044708
Spirochaetia_Cys_a	Cys_a	Spirochaetia	0.40265741	0.277172804	0.528142017	0.459871268	0.565913098
Spirochaetia_His_a	His_a	Spirochaetia	2.354371973	1.727292696	2.98145125	0.214123965	0.342598344
Spirochaetia_His_u	His_u	Spirochaetia	25.90157128	6.381244942	45.42189761	0.349792805	0.465125143
Spirochaetia_Lys_a	Lys_a	Spirochaetia	42.82805665	14.38050236	71.27561094	0.403408355	0.522057871
Spirochaetia_Lys_u	Lys_u	Spirochaetia	47.13135887	20.56633138	73.69638636	0.513477771	0.618986902
Spirochaetia_Met_a	Met_a	Spirochaetia	7.643341063	1.533897875	13.75278425	0.067711879	0.186987323
Spirochaetia_Met_u	Met_u	Spirochaetia	11.80844748	4.767871458	18.8490235	0.180458834	0.30717985
Spirochaetia_Pro_a	Pro_a	Spirochaetia	1.881905492	1.355844083	2.4079669	0.153075904	0.273088427
Spirochaetia_Thr_a	Thr_a	Spirochaetia	9.085833956	1.925620417	16.2460475	0.084063744	0.205489152
Spirochaetia_Thr_u	Thr_u	Spirochaetia	31.20451614	12.30133829	50.10769399	0.25173976	0.371280987
Spirochaetia_Trp_a	Trp_a	Spirochaetia	0.13471259	0.142101613	0.127323567	0.926525831	0.948072943
Spirochaetia_Trp_u	Trp_u	Spirochaetia	24.74727093	7.477360125	42.01718173	0.037091141	0.130560816
Spirochaetia_Tyr_a	Tyr_a	Spirochaetia	4.55906849	4.062730313	5.055406667	0.239318378	0.363103746
Synergistia_Arg_a	Arg_a	Synergistia	0.204924813	0.053419967	0.356429658	0.017507679	0.081088199
Synergistia_Arg_u	Arg_u	Synergistia	5.869953381	2.533906521	9.206000242	0.225209558	0.351806646
Synergistia_Cys_a	Cys_a	Synergistia	0.3753515	0.23303925	0.51766375	0.870433044	0.904702061
Synergistia_His_u	His_u	Synergistia	4.654657742	2.341338092	6.967977392	0.065308958	0.183420904
Synergistia_Lys_a	Lys_a	Synergistia	2.449592213	1.439794571	3.459389854	0.27817912	0.400026483
Synergistia_Met_a	Met_a	Synergistia	1.825350206	0.899838338	2.750862075	0.082303403	0.203066339
Synergistia_Pro_a	Pro_a	Synergistia	0.130852088	0.065672042	0.196032133	0.00738552	0.048744432
Synergistia_Thr_a	Thr_a	Synergistia	0.424518852	0.265643033	0.583394671	0.344356385	0.463826967
Synergistia_Thr_u	Thr_u	Synergistia	5.37426874	2.835793575	7.912743904	0.243568886	0.367441062
Synergistia_Tyr_a	Tyr_a	Synergistia	1.145749492	0.367467783	1.9240312	0.04369352	0.144188616
Tissierellia_Arg_a	Arg_a	Tissierellia	3.367241094	1.704496379	5.029985808	0.250810184	0.371280987
Tissierellia_Arg_u	Arg_u	Tissierellia	44.47973058	33.1970961	55.76236506	0.043251351	0.144188616
Tissierellia_Cys_a	Cys_a	Tissierellia	0.034512483	0.0128355	0.056189467	0.301928049	0.421740766
Tissierellia_His_u	His_u	Tissierellia	1.022864552	0.5527806	1.492948504	0.19213282	0.317019153
Tissierellia_Lys_a	Lys_a	Tissierellia	2.970888471	2.151428488	3.790348454	0.121078193	0.245881869
Tissierellia_Lys_u	Lys_u	Tissierellia	2.72905164	1.755696071	3.702407208	0.110301599	0.231108113
Tissierellia_Met_a	Met_a	Tissierellia	16.69871339	13.8252247	19.57220208	0.231707369	0.357723657
Tissierellia_Met_u	Met_u	Tissierellia	0.678412598	0.664533113	0.692292083	0.543668713	0.652402455
Tissierellia_Pro_a	Pro_a	Tissierellia	0.181035546	0.083328208	0.278742883	0.207848174	0.334584866
Tissierellia_Thr_a	Thr_a	Tissierellia	0.116978669	0.019253246	0.214704092	0.049434266	0.155364836
Tissierellia_Thr_u	Thr_u	Tissierellia	137.7878943	154.3774028	121.1983859	0.206792441	0.334584866
Tissierellia_Tyr_a	Tyr_a	Tissierellia	0.154521521	0.057657208	0.251385833	0.131356584	0.256875098

I. OTUs designations

Sr no.	Species_OTUs	16S reference sequence
1	Neisseria sp. OTU_2	ATTGAACGCTGGCGGCATGCTTTACACATGCAAGTCGGACGGCAGCACAGAGAAGCTTGCTTCTTGGGTGGCGAGTGGCGAACGGGTGAGTAACATATCGGAACGTACCGAGCAGTGGGGGATAACTAATCGAAAGATTAGCTAATACCGCATATATTCTGAGGAAGAAAGCAGGGGACCATTTGGCCTTGCGCTGTTTGAGCGGCCGATATCTGATTAGCTAGTTGGTGGGGTAAAGGCCTACCAAGGCGACGATCAGTAGCGGGTCTGAGAGGATGATCCGCCACACTGGGACTGAGACACGGCCCAGACTCCTACGGGAGGCAGCAGTGGGGAATTTTGGACAATGGGCGCAAGCCTGATCCAGCCATGCCGCGTGTCTGAAGAAGGCCTTCGGGTTGTAAAGGACTTTTGTCAGGGAAGAAAAGGGCGGGGTTAATACCCCTGTCTGATGACGGTACCTGAAGAATAAGCACCGGCTAACTACGTGCCAGCAGCCGCGGTAATACGTAGGGTGCGAGCGTTAATCGGAATTACTGGGCGTAAAGCGGGCGCAGACGGTTACTTAAGCAGGATGTGAAATCCCCGGGCTCAACCTGGGAACTGCGTTCTGAACTGGGTGACTAGAGTGTGTCAGAGGGAGGTAGAATTCCACGTGTAGCAGTGAAATGCGTAGAGATGTGGAGGAATACCGATGGCGAAGGCAGCCTCCTGGGATAACACTGACGTTCATGCCCGAAAGCGTGGGTAGCAAACAGGATTAGATACCCTGGTAGTCCACGCCCTAAACGATGTCGATTAGCTGTTGGGCAGCATGACTGCTTAGTAGCGAAGCTAACGCGTGAAATCGACCGCCTGGGGAGTACGGTCGCAAGATTAAAACTCAAAGGAATTGACGGGGACCCGCACAAGCGGTGGATGATGTGGATTAATTCGATGCAACGCGAAGAACCTTACCTGGTCTTGACATGTACGGAACCCTCCAGAGACGGAGGGGTGCCTTCGGGAGCCGTAACACAGGTGCTGCATGGCTGTCGTCAGCTCGTGTCGTGAGATGTTGGGTTAAGTCCCGCAACGAGCGCAACCCTTGTCATTAGTTGCCATCATTAAGTTGGGCACTCTAATGAGACTGCCGGTGACAAGCCGGAGGAAGGTGGGGATGACGTCAAGTCCTCATGGCCCTTATGACCAGGGCTTCACACGTCATACAATGGTCGGTACAGAGGGTAGCCAAGCCGCGAGGTGGAGCCAATCTCACAAAACCGATCGTAGTCCGGATTGCACTCTGCAACTCGAGTGCATGAAGTCGGAATCGCTAGTAATCGCAGGTCAGCATACTGCGGTGAATACGTTCCCGGGTCTTGTACACACCGCCCGTCACACCATGGGAGTGGGGGATACCAGAAGTAGGTAGGGTAACCGCAAGGAGCCCGCTTACCACGGTATGCTTCATGACTGGGGTG
2	Streptococcus sp. OTU_3	GACGAACGCTGGCGGCGTGCCTAATACATGCAAGTAGAACGCTGAAGAGAGGAGCTTGCTCTTCTTGGATGAGTTGCGAACGGGTGAGTAACGCGTAGGTAACCTGCCTGGTAGCGGGGGATAACTATTGGAAACGATAGCTAATACCGCATGAAATTGCTTATCGCATGATAATTAATTGAAAGATGCAATTGCATCACTACCAGATGGACCTGCGTTGTATTAGCTAGTTGGTGAGGTAACGGCTCACCAAGGCGACGATACATAGCCGACCTGAGAGGGTGATCGGCCACACTGGGACTGAGACACGGCCCAGACTCCTACGGGAGGCAGCAGTAGGGAATCTTCGGCAATGGGGGGAACCCTGACCGAGCAACGCCGCGTGAGTGAAGAAGGTTTTCGGATCGTAAAGCTCTGTTGTAAGAGAAGAACGGGTGTGAGAGTGGAAAGTTCACACTGTGACGGTATCTTACCAGAAAGGGACGGCTAACTACGTGCCAGCAGCCGCGGTAATACGTAGGTCCCGAGCGTTGTCCGGATTTATTGGGCGTAAAGCGAGCGCAGGCGGTTAGATAAGTCTGAAGTTAAAGGCTGTGGCTTAACCATAGTATGCTTTGGAAACTGTTTAACTTGAGTGCAGAAGGGGAGAGTGGAATTCCATGTGTAGCGGTGAAATGCGTAGATATATGGAGGAACACCGGTGGCGAAAGCGGCTCTCTGGTCTGTAACTGACGCTGAGGCTCGAAAGCGTGGGGAGCAAACAGGATTAGATACCCTGGTAGTCCACGCCGTAAACGATGAGTGCTAGGTGTTAGGCCCTTTCCGGGGCTTAGTGCCGCAGCTAACGCATTAAGCACTCCGCCTGGGGAGTACGACCGCAAGGTTGAAACTCAAAGGAATTGACGGGGGCCCGCACAAGCGGTGGAGCATGTGGTTTAATTCGAAGCAACGCGAAGAACCTTACCAGGTCTTGACATCCCTCTGACCGCTCTAGAGATAGAGTTTTCCTTCGGGACAGAGGTGACAGGTGGTGCATGGTTGTCGTCAGCTCGTGTCGTGAGATGTTGGGTTAAGTCCCGCAACGAGCGCAACCCCTATTGTTAGTTGCCATCATTGAGTTGGGCACTCTAGCGAGACTGCCGGTAATAAACCGGAGGAAGGTGGGGATGACGTCAAATCATCATGCCCCTTATGACCTGGGCTACACACGTGCTACAATGGCTGGTACAACGAGTCGCAAGCCGGTGACGGCAAGCTAATCTCTGAAAGCCAGTCTCAGTTCGGATTGTAGGCTGCAACTCGCCTACATGAAGTCGGAATCGCTAGTAATCGCGGATCAGCACGCCGCGGTGAATACGTTCCCGGGCCTTGTACACACCGCCCGTCACACCACGAGAGTTTGTAACACCCGAAGTCGGTGAGGTAACCGTAAGGAGCCAGCCGCCTAAGGTGGGATAGATGATTGGGGTG
3	Streptococcus sp. OTU_4	GACGAACGCTGGCGGCGTGCCTAATACATGCAAGTAGAACGCTGAAGAGAGGAGCTTGCTCTTCTTGGATGAGTTGCGAACGGGTGAGTAACGCGTAGGTAACCTGCCTTGTAGCGGGGGATAACTATTGGAAACGATAGCTAATACCGCATAACAATGGATGACACATGTCATTTATTTGAAAGGGGCAATTGCTCCACTACAAGATGGACCTGCGTTGTATTAGCTAGTAGGTGAGGTAACGGCTCACCTAGGCGACGATACATAGCCGACCTGAGAGGGTGATCGGCCACACTGGGACTGAGACACGGCCCAGACTCCTACGGGAGGCAGCAGTAGGGAATCTTCGGCAATGGGGGCAACCCTGACCGAGCAACGCCGCGTGAGTGAAGAAGGTTTTCGGATCGTAAAGCTCTGTTGTAAGTCAAGAACGAGTGTGAGAGTGGAAAGTTCACACTGTGACGGTAGCTTACCAGAAAGGGACGGCTAACTACGTGCCAGCAGCCGCGGTAATACGTAGGTCCCGAGCGTTGTCCGGATTTATTGGGCGTAAAGCGAGCGCAGGCGGTTTGATAAGTCTGAAGTTAAAGGCTGTGGCTCAACCATAGTTCGCTTTGGAAACTGTCAAACTTGAGTGCAGAAGGGGAGAGTGGAATTCCATGTGTAGCGGTGAAATGCGTAGATATATGGAGGAACACCGGTGGCGAAAGCGGCTCTCTGGTCTGTAACTGACGCTGAGGCTCGAAAGCGTGGGGAGCGAACAGGATTAGATACCCTGGTAGTCCACGCCGTAAACGATGAGTGCTAGGTGTTGGATCCTTTCCGGGATTCAGTGCCGCAGCTAACGCATTAAGCACTCCGCCTGGGGAGTACGACCGCAAGGTTGAAACTCAAAGGAATTGACGGGGGCCCGCACAAGCGGTGGAGCATGTGGTTTAATTCGAAGCAACGCGAAGAACCTTACCAGGTCTTGACATCCCGATGCTATTTCTAGAGATAGAAAGTTACTTCGGTACATCGGTGACAGGTGGTGCATGGTTGTCGTCAGCTCGTGTCGTGAGATGTTGGGTTAAGTCCCGCAACGAGCGCAACCCCTATTGTTAGTTGCCATCATTCAGTTGGGCACTCTAGCGAGACTGCCGGTAATAAACCGGAGGAAGGTGGGGATGACGTCAAATCATCATGCCCCTTATGACCTGGGCTACACACGTGCTACAATGGTTGGTACAACGAGTTGCGAGTCGGTGACGGCAAGCTAATCTCTTAAAGCCAATCTCAGTTCGGATTGTAGGCTGCAACTCGCCTACATGAAGTCGGAATCGCTAGTAATCGCGGATCAGCACGCCGCGGTGAATACGTTCCCGGGCCTTGTACACACCGCCCGTCACACCACGAGAGTTTGTAACACCCGAAGTCGGTGAGGTAACCTTTTGGAGCCAGCCGCCTAAGGTGGGATAGATGATTGGGGTG
4	Rothia sp. OTU_5	GACGAACGCTGGCGGCGTGCTTAACACATGCAAGTCGAACGATGAAGCCTAGCTTGCTAGGTGGATTAGTGGCGAACGGGTGAGTAATACGTGAGTAACCTACCTTTAACTCTGGGATAAGCCTGGGAAACTGGGTCTAATACCGGATACGACCAATCTCCGCATGGGGTGTTGGTGGAAAGCGTTATGTAGTGGTTATAGATGGGCTCACGGCCTATCAGCTTGTTGGTGAGGTAACGGCTCACCAAGGCGACGACGGGTAGCCGGCCTGAGAGGGTGACCGGCCACACTGGGACTGAGACACGGCCCAGACTCCTACGGGAGGCAGCAGTGGGGAATATTGCACAATGGGCGCAAGCCTGATGCAGCGACGCCGCGTGAGGGATGACGGCCTTCGGGTTGTAAACCTCTGTTAGCAGGGAAGAAGAGAGATTGACGGTACCTGCAGAGAAAGCGCCGGCTAACTACGTGCCAGCAGCCGCGGTAATACGTAGGGCGCGAGCGTTGTCCGGAATTATTGGGCGTAAAGAGCTTGTAGGCGGTTTGTCGCGTCTGCTGTGAAAGGCCGGAGCTTAACTCCGTGTATTGCAGTGGGTACGGGCAGACTAGAGTGCAGTAGGGGAGACTGGAATTCCTGGTGTAGCGGTGGAATGCGCAGATATCAGGAGGAACACCGATGGCGAAGGCAGGTCTCTGGGCTGTAACTGACGCTGAGAAGCGAAAGCATGGGGAGCGAACAGGATTAGATACCCTGGTAGTCCATGCCGTAAACGTTGGGCACTAGGTGTGGGGAACATTCCACGTTTTCCGCGCCGTAGCTAACGCATTAAGTGCCCCGCCTGGGGAGTACGGCCGCAAGGCTAAAACTCAAAGAAATTGACGGGGGCCCGCACAAGCGGCGGAGCATGCGGATTAATTCGATGCAACGCGAAGAACCTTACCAAGGCTTGACATATACTGGACCGCATCAGAGATGGTGTTTCCCTTCGGGGCTGGTATACAGGTGGTGCATGGTTGTCGTCAGCTCGTGTCGTGAGATGTTGGGTTAAGTCCCGCAACGAGCGCAACCCTCGTTCTATGTTGCCAGCACGTTATGGTGGGGACTCATAGGAGACTGCCGGGGTCAACTCGGAGGAAGGTGGGGATGACGTCAAATCATCATGCCCCTTATGTCTTGGGCTTCACGCATGCTACAATGGCCGGTACAGAGGGTTGCGATACTGTGAGGTGGAGCTAATCCCTAAAAGCCGGTCTCAGTTCGGATTGGGGTCTGCAACTCGACCCCATGAAGTCGGAGTCGCTAGTAATCGCAGATCAGCAACGCTGCGGTGAATACGTTCCCGGGCCTTGTACACACCGCCCGTCAAGTCACGAAAGTTGGTAACACCCAAAGCCGGTGGCCTAACCCTTTTGGGAGGGAGCCGTCTAAGGTGGGATTGGCGATTGGGACT

Sr no.	Species_OTUs	16S reference sequence
5	Rothia sp. OTU_6	GACGAACGCTGGCGGCGTGCTTAACACATGCAAGTCGAACGATGAAGCGGTGCTTGCACCGTGGATTAGTGGCGAACGG<br>GTGAGTAATACGTGAGTGACCTACCTTTGACTCTGGGATAAGCCTGGGAAACTGGGTCTAATACCGGATACGACCAATCTC<br>TGCATGGGGTGTTGGTGGAAAGCGTTATGGAGTGGTTTTAGATGGGCTCACGGCCTATCAGCTTGTTGGTGAGGTAATGGC<br>TTACCAAGGCGACGACGGGTAGCCGGCCTGAGAGGGTGACCGGCCACACTGGGACTGAGACACGGCCCAGACTCCTAC<br>GGGAGGCAGCAGTGGGGAATATTGCACAATGGGCGCAAGCCTGATGCAGCGACGCCGCGTGAGGGATGACGGCCTTCG<br>GGTTGTAAACCTCTGTTAGCATCGAAGAAGCGAAAGTGACGGTAGGTGCAGAGAAAGCGCCGGCTAACTACGTGCCAGCA<br>GCCGCGGTAATACGTAGGGCGCGAGCGTTGTCCGGAATTATTGGGCGTAAAGAGCTTGTAGGCGGTTGGTCGCGTCTGC<br>TGTGAAAGGCCGGGGCTTAACTCCGGTTTTGCAGTGGGTACGGGCTAACTAGAGTGCAGTAGGGGAGACTGGAATTCCTG<br>GTGTAGCGGTGGAATGCACAGATATCAGGAGGAACACCGATGGCGAAGGCAGGTCTCTGGGCTGTAACTGACGCTGAGAA<br>GCGAAAGCATGGGGAGCGAACAGGATTAGATACCCTGGTAGTCCATGCCGTAAACGTTGGGCACTAGGTGTGGGGGACAT<br>TCCACGTTTTCCGCGCCGTAGCTAACGCATTAAGTGCCCCGCCTGGGGAGTACGGCCGCAAGGCTAAAACTCAAAGGAAT<br>TGACGGGGGCCCGCACAAGCGGCGGAGCATGCGGATTAATTCGATGCAACGCGAAGAACCTTACCAAGGCTTGACATGCA<br>TTAGATCGCGTCAGAGATGGCGTTTCCCTTCGGGGCTGGTGTACAGGTGGTGCATGGTTGTCGTCAGCTCGTGTCGTGAG<br>ATGTTGGGTTAAGTCCCGCAACGAGCGCAACCCTCGTTCTATGTTGCCAGCGGTTCGGCCGGGGACTCATAGGAGACTG<br>CCGGGGTCAACTCGGAGGAAGGTGGGGATGACGTCAAATCATCATGCCCCTTATGTCTTGGGCTTCACGCATGCTACAAT<br>GGCCGGTACAGAGGGTTGCGATACTGTGAGGTGGAGCTAATCCCTTAAAGCCGGTCTCAGTTCGGATTGGGGTCTGCAAC<br>TCGACCCCATGAAGTCGGAGTCGCTAGTAATCGCAGATCAGCAACGCTGCGGTGAATACGTTCCCGGGCCTTGTACACAC<br>CGCCCGTCAAGTCACGAAAGTTGGTAACACCCGAAGCCGGTGGCCTAACCCTTGTGGGGGGAGCCGTCGAAGGTGGGAT<br>TGGCGATTGGGACT
6	Veillonella sp. OTU_7	GACGAACGCTGGCGGCGTGCTTAACACATGCAAGTCGAACGAAGAGCGATGGAAGCTTGCTTCTATCAATCTTAGTGGCGA<br>ACGGGTGAGTAACGCGTAATCAACCTGCCCTTCAGAGGGGGACAACAGTTGGAAACGACTGCTAATACCGCATACGATCTA<br>ATCTCGGCATCGAGGATAGATGAAAGGTGGCCTCTACATGTAAGCTATCACTGAAGGAGGGGATTGCGTCTGATTAGCTAG<br>TTGGAGGGGTAACGGCCCACCAAGGCGATGATCAGTAGCCGGTCTGAGAGGATGAACGGCCACATTGGGACTGAGACAC<br>GGCCCAGACTCCTACGGGAGGCAGCAGTGGGGAATCTTCCGCAATGGACGAAAGTCTGACGGAGCAACGCCGCGTGAGT<br>GATGACGGCCTTCGGGTTGTAAAGCTCTGTTAATCGGGACGAAAGGCCTTCTTGCGAATAGTGAGAAGGATTGACGGTACC<br>GGAATAGAAAGCCACGGCTAACTACGTGCCAGCAGCCGCGGTAATACGTAGGTGGCAAGCGTTGTCCGGAATTATTGGGC<br>GTAAAGCGCGCGCAGGCGGATAGGTCAGTCTGTCTTAAAAGTTCGGGGCTTAACCCCGTGATGGGATGGAAACTGCCAAT<br>CTAGAGTATCGGAGAGGAAAGTGGAATTCCTAGTGTAGCGGTGAAATGCGTAGATATTAGGAAGAACACCAGTGGCGAAGG<br>CGACTTTCTGGACGAAAACTGACGCTGAGGCGCGAAAGCCAGGGGAGCGAACGGGATTAGATACCCCGGTAGTCCTGGC<br>CGTAAACGATGGGTACTAGGTGTAGGAGGTATCGACCCCTTCTGTGCCGGAGTTAACGCAATAAGTACCCCGCCTGGGGA<br>GTACGACCGCAAGGTTGAAACTCAAAGGAATTGACGGGGGCCCGCACAAGCGGTGGAGTATGTGGTTTAATTCGACGCAA<br>CGCGAAGAACCTTACCAGGTCTTGACATTGATGGACAGAACCAGAGATGGTTCCTCTTCTTCGGAAGCCAGAAAACAGGTG<br>GTGCACGGTTGTCGTCAGCTCGTGTCGTGAGATGTTGGGTTAAGTCCCGCAACGAGCGCAACCCCTATCTTATGTTGCCA<br>GCACTTTGGGTGGGGACTCATGAGAGACTGCCGCAGACAATGCGGAGGAAGGCGGGGATGACGTCAAATCATCATGCCC<br>CTTATGACCTGGGCTACACACGTACTACAATGGGAGTTAATAGACGGAAGCGAGATCGCGAGATGGAGCAAACCCGAGAAA<br>CACTCTCTCAGTTCGGATCGTAGGCTGCAACTCGCCTACGTGAAGTCGGAATCGCTAGTAATCGCAGGTCAGCATACTGC<br>GGTGAATACGTTCCCGGGCCTTGTACACACCGCCCGTCACACCACGAAAGTCGGAAGTGCCCAAAGCCGGTGGGGTAAC<br>CTTCGGGAGCCAGCCGTCTAAGGTAAAGTCGATGATTGGGGTG
7	Streptococcus sp. OTU_8	GACGAACGCTGGCGGCGTGCCTAATACATGCAAGTAGAACGCACAGTTTATACCGTAGCTTGCTACACCATAGACTGTGAG<br>TTGCGAACGGGTGAGTAACGCGTAGGTAACCTGCCTGGTAGCGGGGGATAACTATTGGAAACGATAGCTAATACCGCATAA<br>TATTAATTATTGCATGATAATTGATTGAAAGGTGCAATTGCACCACTACCAGATGGACCTGCGTTGTATTAGCTAGTAGGTGA<br>GGTAACGGCTCACCTAGGCGACGATACATAGCCGACCTGAGAGGGTGATCGGCCACACTGGGACTGAGACACGGCCCAG<br>ACTCCTACGGGAGGCAGCAGTAGGGAATCTTCGGCAATGGACGAAAGTCTGACCGAGCAACGCCGCGTGAGTGAAGAAGG<br>TTTTCGGATCGTAAAGCTCTGTTGTAAGAGAAGAACGGGTGTGAGAGTGGAAAGTTCACACTGTGACGGTATCTTACCAGAA<br>AGGGACGGCTAACTACGTGCCAGCAGCCGCGGTAATACGTAGGTCCCGAGCGTTGTCCGGATTTATTGGGCGTAAAGCGA<br>GCGCAGGCGGTTAGATAAGTCTGAAGTTAAAGGCTGTGGCTTAACCATAGTACGCTTTGGAAACTGTTTAACTTGAGTGCAG<br>AAGGGGAGAGTGGAATTCCATGTGTAGCGGTGAAATGCGTAGATATATGGAGGAACACCGGTGGCGAAAGCGGCTCTCTG<br>GTCTGTAACTGACGCTGAGGCTCGAAAGCGTGGGGAGCGAACAGGATTAGATACCCTGGTAGTCCACGCCGTAAACGATG<br>AGTGCTAGGTGTTAGGCCCTTTCCGGGGCTTAGTGCCGCAGCTAACGCATTAAGCACTCCGCCTGGGGAGTACGACCGC<br>AAGGTTGAAACTCAAAGGAATTGACGGGGGCCCGCACAAGCGGTGGAGCATGTGGTTTAATTCGAAGCAACGCGAAGAACC<br>TTACCAGGTCTTGACATCCCGATGCCCGCTCTAGAGATAGAGTTTTACTTCGGTACATCGGTGACAGGTGGTGCATGGTTG<br>TCGTCAGCTCGTGTCGTGAGATGTTGGGTTAAGTCCCGCAACGAGCGCAACCCTTATTGTTAGTTGCCATCATTCAGTTGG<br>GCACTCTAGCGAGACTGCCGGTAATAAACCGGAGGAAGGTGGGGATGACGTCAAATCATCATGCCCCTTATGACCTGGGC<br>TACACACGTGCTACAATGGCTGGTACAACGAGTCGCAAGTCGGTGACGGCAAGCTAATCTCTTAAAGCCAGTCTCAGTTCG<br>GATTGTAGGCTGCAACTCGCCTACATGAAGTCGGAATCGCTAGTAATCGCGGATCAGCACGCCGCGGTGAATACGTTCCC<br>GGGCCTTGTACACACCGCCCGTCACACCACGAGAGTTTGTAACACCCGAAGTCGGTGAGGTAACCTTTTAGGAGCCAGCC<br>GCCTAAGGTGGGATAGATGATTGGGGTG
8	Gemella sp. OTU_9	GACGAACGCTGGCGGCGTGCCTAATACATGCAAGTCGAGCGAAGTTTTTCTGGTGCTTGCACTAGAAAAACTTAGCGGCGA<br>ACGGGTGAGTAACACGTAAAGAACCTGCCTCATAGACTGGGACAACTATTGGAAACGATAGCTAATACCGGATAACAGCATT<br>AACTGCATGGTTGATGTTTAAAAGTTGGTTTTGCTAACACTATGAGATGGCTTTGCGGTGCATTAGCTAGTTGGTGGGGTAAA<br>GGCCTACCAAGGCGACGATGCATAGCCGACCTGAGAGGGTGATCGGCCACACTGGGACTGAGACACGGCCCAGACTCCT<br>ACGGGAGGCAGCAGTAGGGAATCTTCCGCAATGGGCGAAAGCCTGACGGAGCAACGCCGCGTGAGTGAAGAAGGATTTC<br>GGTTCGTAAAGCTCTGTTGTTAGGGAAGAATGATTGTGTAGTAACTATACACAGTAGAGACGGTACCTAACCAGAAAGCCAC<br>GGCTAACTACGTGCCAGCAGCCGCGGTAATACGTAGGTGGCAAGCGTTGTCCGGAATTATTGGGCGTAAAGCGCGCGCA<br>GGTGGTTTAATAAGTCTGATGTGAAAGCCCACGGCTCAACCGTGGAGGGTCATTGGAAACTGTTAAACTTGAGTGCAGGAG<br>AGAAAAGTGGAATTCCTAGTGTAGCGGTGAAATGCGTAGAGATTAGGAGGAACACCAGTGGCGAAGGCGGCTTTTTGGCCT<br>GTAACTGACACTGAGGCGCGAAAGCGTGGGGAGCAAACAGGATTAGATACCCTGGTAGTCCACGCCGTAAACGATGAGTG<br>CTAAGTGTTGGTCTCATAAGAGATCAGTGCTGCAGCTAACGCATTAAGCACTCCGCCTGGGGAGTACGACCGCAAGGTTGA<br>AACTCAAAGGAATTGACGGGGACCCGCACAAGCGGTGGAGCATGTGGTTTAATTCGAAGCAACGCGAAGAACCTTACCAAG<br>TCTTGACATACTGTGAGGACACAAGAGATTGTGTTGTTCTGACCTTTGGTTAGACACAGATACAGGTGGTGCATGGTTGTCG<br>TCAGCTCGTGTCGTGAGATGTTGGGTTAAGTCCCGCAACGAGCGCAACCCTTATATCTAGTTGCCAGCAGTAAGATGGGGA<br>CTCTAGATAGACTGCCAGTGATAAACTGGAGGAAGGTGGGGATGACGTCAAATCATCATGCCCCTTATGACTTGGGCTACA<br>CACGTGCTACAATGGATAGGAACAAAGAGAAGCGAGCTCGCGAGAGTAAGCAAACCTCACAAAACTATTCTCAGTTCGGATT<br>GTAGTCTGCAACTCGACTACATGAAGCTGGAATCGCTAGTAATCGCGAATCAGAATGTCGCGGTGAATACGTTCCCGGGTC<br>TTGTACACACCGCCCGTCACACCACGAGAGTTTGTAACACCCGAAGACGGTGGCCTAACCTTTTAGGAGGGAGCCGGTCA<br>CGGTGGGACAGATGATTGGGGTG

Sr no.	Species_OTUs	16S reference sequence
9	Fusobacterium sp. OTU_10	GATGAACGCTGACAGAATGCTTAACACATGCAAGTCAACTTGAATTTGGGTTTTTAACTTAGATTTGGGTGGCGGACGGGTG<br>AGTAACGCGTAAAGAACTTGCCTCACAGCTAGGGACAACATTTAGAAATGAATGCTAATACCTGATATTATGATTTTAAGGCAT<br>CTTAGAATTATGAAAGCTATAAGCACTGTGAGAGAGCTTTGCGTCCCATTAGCTAGTTGGAGAGGTAACAGCTCACCAAGGC<br>GATGATGGGTAGCCGGCCTGAGAGGGTGAACGGCCACAAGGGGACTGAGACACGGCCCTTACTCCTACGGGAGGCAGCA<br>GTGGGGAATATTGGACAATGGACCGAGAGTCTGATCCAGCAATTCTGTGTGCACGATGAAGTTTTTCGGAATGTAAAGTGCT<br>TTCAGTTGGGAAGAAAGAAATGACGGTACCAACAGAAGAAGTGACGGCTAAATACGTGCCAGCAGCCGCGGTAATACGTAT<br>GTCACGAGCGTTATCCGGATTTATTGGGCGTAAAGCGCGTCTAGGTGGTTATGTAAGTCTGATGTGAAAATGCAGGGCTCA<br>ACTCTGTATTGCGTTGGAAACTGTATAACTAGAGTACTGGAGAGGTAAGCGGAACTACAAGTGTAGAGGTGAAATTCGTAGAT<br>ATTTGTAGGAATGCCGATGGGGAAGCCAGCTTACTGGACAGATACTGACGCTAAAGCGCGAAAGCGTGGGTAGCAAACAGG<br>ATTAGATACCCTGGTAGTCCACGCTGTAAACGATGATTACTAGGTGTTGGGGGTCGAACCTCAGCGCCCAAGCAAACGCGA<br>TAAGTAATCCGCCTGGGGAGTACGTACGCAAGTATGAAACTCAAAGGAATTGACGGGGACCCGCACAAGCGGTGGAGCAT<br>GTGGTTTAATTCGACGCAACGCGAGGAACCTTACCAGCGTTTGACATCTTAGGAATGAGATAGAGATATTTCAGTGTCCCTT<br>CGGGGAAACCTAAAGACAGGTGGTGCATGGCTGTCGTCAGCTCGTGTCGTGAGATGTTGGGTTAAGTCCCGCAACGAGCG<br>CAACCCCTTTCGTATGTTACCATCATTAAGTTGGGGACTCATGCGATACTGCCTACGATGAGTAGGAGGAAGGTGGGGATG<br>ACGTCAAGTCATCATGCCCCTTATACGCTGGGCTACACACGTGCTACAATGGGTAGAACAGAGAGTTGCAAAGCCGTGAGG<br>TGAAGCTAATCTCAGAAAACTATTCTTAGTTCGGATTGTACTCTGCAACTCGAGTACATGAAGTTGGAATCGCTAGTAATCGC<br>GAATCAGCAATGTCGCGGTGAATACGTTCTCGGGTCTTGTACACACCGCCCGTCACACCACGAGAGTTGGTTGCACCTGA<br>AGTAGCAGGCCTAACCGTAAGGAGGGATGCTCCGAGGGTGTGATTAGCGATTGGGGTG
10	Streptococcus sp. OTU_11	GACGAACGCTGGCGGCGTGCCTAATACATGCAAGTAGGACGCACAGTTTATACCGTAGCTTGCTACACCATAGACTGTGAG<br>TTGCGAACGGGTGAGTAACGCGTAGGTAACCTGCCTATTAGAGGGGGATAACTATTGGAAACGATAGCTAATACCGCATAAC<br>AGTATGTAACACATGTTAGATGCTTGAAAGATGCAATTGCATCGCTAGTAGATGGACCTGCGTTGTATTAGCTAGTAGGTAGG<br>GTAATGGCCTACCTAGGCGACGATACATAGCCGACCTGAGAGGGTGATCGGCCACACTGGGACTGAGACACGGCCCAGA<br>CTCCTACGGGAGGCAGCAGTAGGGAATCTTCGGCAATGGGGGGAACCCTGACCGAGCAACGCCGCGTGAGTGAAGAAGG<br>TTTTCGGATCGTAAAGCTCTGTTGTTAAGGAAGAACGAGTGTGAGAATGGAAAGTTCATACTGTGACGGTACTTAACCAGAAA<br>GGGACGGCTAACTACGTGCCAGCAGCCGCGGTAATACGTAGGTCCCGAGCGTTGTCCGGATTTATTGGGCGTAAAGCGA<br>GCGCAGGCGGTTAGAAAAGTCTGAAGTGAAAGGCAGTGGCTCAACCATTGTAGGCTTTGGAAACTGTTTAACTTGAGTGCA<br>GAAGGGGAGAGTGGAATTCCATGTGTAGCGGTGAAATGCGTAGATATATGGAGGAACACCGGTGGCGAAAGCGGCTCTCT<br>GGTCTGTAACTGACGCTGAGGCTCGAAAGCGTGGGGAGCGAACAGGATTAGATACCCTGGTAGTCCACGCCGTAAACGAT<br>GAGTGCTAGGTGTTAGGTCCTTTCCGGGACTTAGTGCCGCAGCTAACGCATTAAGCACTCCGCCTGGGGAGTACGACCG<br>CAAGGTTGAAACTCAAAGGAATTGACGGGGGCCCGCACAAGCGGTGGAGCATGTGGTTTAATTCGAAGCAACGCGAAGAAC<br>CTTACCAGGTCTTGACATCCCGATGCTATTTCTAGAGATAGGAAGTTTCTTCGGAACATCGGTGACAGGTGGTGCATGGTT<br>GTCGTCAGCTCGTGTCGTGAGATGTTGGGTTAAGTCCCGCAACGAGCGCAACCCTTATTGTTAGTTGCCATCATTGAGTTG<br>GGCACTCTAGCGAGACTGCCGGTAATAAACCGGAGGAAGGTGGGGATGACGTCAAATCATCATGCCCCTTATGACCTGGG<br>CTACACACGTGCTACAATGGCTGGTACAACGAGTCGCAAGCCGGTGACGGCAAGCTAATCTCTGAAAGCCAGTCTCAGTT<br>CGGATTGTAGGCTGCAACTCGCCTACATGAAGTCGGAATCGCTAGTAATCGCGGATCAGCACGCCGCGGTGAATACGTTC<br>CCGGGCCTTGTACACACCGCCCGTCACACCACGAGAGTTTGTAACACCCGAAGTCGGTGAGGTAACCGTAAGGAGCCAG<br>CCGCCTAAGGTGGGATAGATGATTGGGGTG
11	Streptococcus sp. OTU_13	GACGAACGCTGGCGGCGTGCCTAATACATGCAAGTAGAACGCACAGGATGCACCGTAGTTTACTACACCGTATTCTGTGAG<br>TTGCGAACGGGTGAGTAACGCGTAGGTAACCTGCCTGGTAGCGGGGGATAACTATTGGAAACGATAGCTAATACCGCATAA<br>GAACATTTACTGCATGGTAGATGTTTAAAAGGTGCAAAAGCATCACTACCAGATGGACCTGCGTTGTATTAGCTAGTTGGTGA<br>GGTAACGGCTCACCAAGGCAACGATACATAGCCGACCTGAGAGGGTGATCGGCCACACTGGGACTGAGACACGGCCCAG<br>ACTCCTACGGGAGGCAGCAGTAGGGAATCTTCGGCAATGGGGGGAACCCTGACCGAGCAACGCCGCGTGAGTGAAGAAG<br>GTTTTCGGATCGTAAAGCTCTGTTGTTAAGGAAGAACGTGTGTGAGAGTGGAAAGTTCACACAGTGACGGTACTTAACCAGA<br>AAGGGACGGCTAACTACGTGCCAGCAGCCGCGGTAATACGTAGGTCCCGAGCGTTGTCCGGATTTATTGGGCGTAAAGCG<br>AGCGCAGGCGGTTAGATAAGTCTGAAGTGAAAGGCAGTGGCTCAACCATTGTAGGCTTTGGAAACTGTTTAACTTGAGTGCA<br>GAAGGGGAGAGTGGAATTCCATGTGTAGCGGTGAAATGCGTAGATATATGGAGGAACACCGGTGGCGAAAGCGGCTCTCT<br>GGTCTGTAACTGACGCTGAGGCTCGAAAGCGTGGGGAGCGAACAGGATTAGATACCCTGGTAGTCCACGCCGTAAACGAT<br>GAGTGCTAGGTGTTAGGTCCTTTCCGGGACTTAGTGCCGCAGCTAACGCATTAAGCACTCCGCCTGGGGAGTACGACCG<br>CAAGGTTGAAACTCAAAGGAATTGACGGGGGCCCGCACAAGCGGTGGAGCATGTGGTTTAATTCGAAGCAACGCGAAGAAC<br>CTTACCAGGTCTTGACATCCCTCTGACCACTCTAGAGATAGAGTTTCCCTTCGGGGCAGAGGTGACAGGTGGTGCATGGT<br>TGTCGTCAGCTCGTGTCGTGAGATGTTGGGTTAAGTCCCGCAACGAGCGCAACCCTTATTGTTAGTTGCCATCATTCAGTT<br>GGGCACTCTAGCGAGACTGCCGGTAATAAACCGGAGGAAGGTGGGGATGACGTCAAATCATCATGCCCCTTATGACCTGG<br>GCTACACACGTGCTACAATGGCTGGTACAACGAGTTGCAAGCCGGTGACGGCAAGCTAATCTCTGAAAGCCAGTCTCAGTT<br>CGGATTGTAGGCTGCAACTCGCCTACATGAAGTCGGAATCGCTAGTAATCGCGGATCAGCACGCCGCGGTGAATACGTTC<br>CCGGGCCTTGTACACACCGCCCGTCACACCACGAGAGTTTGTAACACCCGAAGTCGGTGAGGTAACCGTAAGGAGCCAG<br>CCGCCTAAGGTGGGATAGATGATTGGGGTG
12	Schaalia sp. OTU_14	GACGAACGCTGGCGGCGTGCTTAACACATGCAAGTCGAACGCTGAAGCCTAGCTTTTGTTGGGTGGATGAGTGGCGAACG<br>GGTGAGTAACACGTGAGTAACCTGCCCCCTTCTTTGGGATAACGCCCGGAAACGGGTGCTAATACTGGATATTCACTGATC<br>TTCGCATGGGGGTTGGTGGAAAGGTTTTTTCTGGTGGGGGATGGGCTCGCGGCCTATCAGCTTGTTGGTGGGGTGATGG<br>CCTACCAAGGCTTTGACGGGTAGCCGGCCTGAGAGGGTGACCGGTCACATTGGGACTGAGATACGGCCCAGACTCCTAC<br>GGGAGGCAGCAGTGGGGAATATTGCACAATGGGCGAAAGCCTGATGCAGCGACGCCGCGTGAGGGATGGAGGCCTTCGG<br>GTTGTAAACCTCTTTCGCTCATGGTCAAGCCGCAACATTGGTTGTGGTGAGGGTAGTGGGTAAAGAAGCGCCGGCTAACTA<br>CGTGCCAGCAGCCGCGGTAATACGTAGGGCGCGAGCGTTGTCCGGAATTATTGGGCGTAAAGGGCTTGTAGGCGGTTGG<br>TCGCGTCTGCCGTGAAATCCTCTGGCTTAACTGGGGGCGTGCGGTGGGTACGGGCTGACTTGAGTGCGGTAGGGGAGAC<br>TGGAACTCCTGGTGTAGCGGTGGAATGCGCAGATATCAGGAAGAACACCGGTGGCGAAGGCGGGTCTCTGGGCCGTTAC<br>TGACGCTGAGGAGCGAAAGCGTGGGGAGCGAACAGGATTAGATACCCTGGTAGTCCACGCTGTAAACGTTGGGCACTAGG<br>TGTGGGGGCCACCCGTGGTTTCTGCGCCGTAGCTAACGCTTTAAGTGCCCCGCCTGGGGAGTACGGCCGCAAGGCTAAA<br>ACTCAAAGGAATTGACGGGGGCCCGCACAAGCGGCGGAGCATGCGGATTAATTCGATGCAACGCGAAGAACCTTACCAAG<br>GCTTGACATGCACGGCGGCACTGCAGAGATGTGGTGGCATTTAGTTGGTCGTGTGCAGGTGGTGCATGGTTGTCGTCAGC<br>TCGTGTCGTGAGATGTTGGGTTAAGTCCCGCAACGAGCGCAACCCTTGCCCTATGTTGCCAGCACGTGATGGTGGGGACT<br>CGTGGGGGACTGCCGGGGTTAACTCGGAGGAAGGTGGGGATGACGTCAAATCATCATGCCCCTTATGTCTTGGGCTTCAC<br>GCATGCTACAATGGCTGGTACAGAGGGTTGCGATATCGTGAGGTGGAGCGAATCCCTTAAAGCCAGTCTCAGTTCGGATTG<br>GGGTCTGCAACTCGACCCCATGAAGGTGGAGTCGCTAGTAATCGCAGATCAGCAACGCTGCGGTGAATACGTTCTCGGGC<br>CTTGTACACACCGCCCGTCACGTCACGAAAGTTGGTAACACCCGAAGCCCATGGCCTAACCGTTTATCGGGGGGAGTGGT<br>CGAAGGTGGGATTGGCGATTGGGACG

Sr no.	Species_OTUs	16S reference sequence
13	Lacticaseibacillus sp. OTU_15	GATGAACGCTGGCGGCGTGCCTAATACATGCAAGTCGAACGAGTTCTCGTTGATGATCGGTGCTTGCACCGAGATTCAACA<br>TGGAACGAGTGGCGGACGGGTGAGTAACACGTGGGTAACCTGCCCTTAAGTGGGGGATAACATTTGGAAACAGATGCTAAT<br>ACCGCATAGATCCAAGAACCGCATGGTTCTTGGCTGAAAGATGGCGTAAGCTATCGCTTTTGGATGGACCCGCGGCGTATT<br>AGCTAGTTGGTGAGGTAATGGCTCACCAAGGCGATGATACGTAGCCGAACTGAGAGGTTGATCGGCCACATTGGGACTGA<br>GACACGGCCCAAACTCCTACGGGAGGCAGCAGTAGGGAATCTTCCACAATGGACGCAAGTCTGATGGAGCAACGCCGCG<br>TGAGTGAAGAAGGCTTTCGGGTCGTAAAACTCTGTTGTTGGAGAAGAATGGTCGGCAGAGTAACTGTTGTCGGCGTGACGG<br>TATCCAACCAGAAAGCCACGGCTAACTACGTGCCAGCAGCCGCGGTAATACGTAGGTGGCAAGCGTTATCCGGATTTATTG<br>GGCGTAAAGCGAGCGCAGGCGGTTTTTTAAGTCTGATGTGAAAGCCCTCGGCTTAACCGAGGAAGCGCATCGGAAACTGG<br>GAAACTTGAGTGCAGAAGAGGACAGTGGAACTCCATGTGTAGCGGTGAAATGCGTAGATATATGGAAGAACACCAGTGGCG<br>AAGGCGGCTGTCTGGTCTGTAACTGACGCTGAGGCTCGAAAGCATGGGTAGCGAACAGGATTAGATACCCTGGTAGTCCA<br>TGCCGTAAACGATGAATGCTAGGTGTTGGAGGGTTTCCGCCCTTCAGTGCCGCAGCTAACGCATTAAGCATTCCGCCTGG<br>GGAGTACGACCGCAAGGTTGAAACTCAAAGGAATTGACGGGGGCCCGCACAAGCGGTGGAGCATGTGGTTTAATTCGAAG<br>CAACGCGAAGAACCTTACCAGGTCTTGACATCTTTTGATCACCTGAGAGATCAGGTTTCCCCTTCGGGGGCAAAATGACAG<br>GTGGTGCATGGTTGTCGTCAGCTCGTGTCGTGAGATGTTGGGTTAAGTCCCGCAACGAGCGCAACCCTTATGACTAGTTG<br>CCAGCATTTAGTTGGGCACTCTAGTAAGACTGCCGGTGACAAACCGGAGGAAGGTGGGGATGACGTCAAATCATCATGCC<br>CCTTATGACCTGGGCTACACACGTGCTACAATGGATGGTACAACGAGTTGCGAGACCGCGAGGTCAAGCTAATCTCTTAAA<br>GCCATTCTCAGTTCGGACTGTAGGCTGCAACTCGCCTACACGAAGTCGGAATCGCTAGTAATCGCGGATCAGCACGCCGC<br>GGTGAATACGTTCCCGGGCCTTGTACACACCGCCCGTCACACCATGAGAGTTTGTAACACCCGAAGCCGGTGGCGTAAC<br>CCTTTTAGGGAGCGAGCCGTCTAAGGTGGGACAAATGATTAGGGTG
14	Arachnia sp. OTU_16	GACGAACGCTGGCGGTGTGCTTAACACATGCAAGTCGAACGGTAAGGCCCTTTTTTGGGGTACACGAGTGGCGAACGGGT<br>GAGTAACACGTGAGTAACCTGCCCTCATCTCTGGGATAACAGTCGGAAACGGTTGCTAATACTGGATATGAGCTTGCAAGG<br>CATCTTGTGGGTTGGAAAGATATTTTTTGGTTGGGGATGGACTCGCGGCCTATCAGCTTGTTGGTGAGGTAGTGGCTCACC<br>AAGGCTTCGACGGGTAGCCGGCCTGAGAGGGCGACCGGCCACATTGGGACTGAGATACGGCCCAAACTCCTACGGGAG<br>GCAGCAGTGGGGAATATTGCACAATGGACGGAAGTCTGATGCAGCAACGCCGCGTGCGGGATGACGGCCTTCGGGTTGT<br>AAACCGCTTTCAATTGTGACGAAGCTTTTTGTGACGGTAGCAGTAGAAGAAGCACCGGCTAACTACGTGCCAGCAGCCGCG<br>GTGATACGTAGGGTGCGAGCGTTGTCCGGATTTATTGGGCGTAAAGAGCTTGTAGGTGGTTTGTCGCGTCGGGAGTGAAAA<br>CTCAGGGCTTAACTCTGAGCTTGCTTTCGATACGGGCTGACTTGAGGGAGGTAGGGGAGAATGGAATTCCTGGTGGAGCG<br>GTGGAATGCGCAGATATCAGGAGGAACACCGGTGGCGAAGGCGGTTCTCTGGACCTTTCCTGACGCTGAGAAGCGAAAGC<br>GTGGGGAGCGAACAGGCTTAGATACCCTGGTAGTCCACGCTGTAAACGGTGGGTACTAGGTGTGGGGGACATTCCACGTT<br>CTCCGTGCCGTAGCTAACGCATTAAGTACCCCGCCTGGGGAGTACGGCCGCAAGGCTAAAACTCAAAGGAATTGACGGGG<br>CCCCGCACAAGCGGCGGAGCATGCGGATTAATTCGATGCAACGCGAAGAACCTTACCTGGGTTTGACATATGCCGGGAGC<br>GTTCAGAGATGGGCGTGCCTTTTTGGTCGGTTTACAGGTGGTGCATGGCTGTCGTCAGCTCGTGTCGTGAGATGTTGGGT<br>TAAGTCCCGCAACGAGCGCAACCCTCGTCCAATGTTGCCAGCGGGTTATGCCGGGGACTCATTGGAGACCGCCGGGGTC<br>AACTCGGAGGAAGGTGGGGATGACGTCAAGTCATCATGCCCCTTATGTCCAGGGCTTCACGCATGCTACAATGGCCGGTA<br>CAGTGAGCTGCGAGCCTGTGAGGGTGAGCGAATCTCTAAAAGCCGGTCTCAGTTCGGATTGGGGTCTGCAACTCGACCC<br>CATGAAGTCGGAGTCGCTAGTAATCGCAGATCAGCAACGCTGCGGTGAATACGTTCCCGGGGCTTGTACACACCGCCCGT<br>CAAGTCATGAAAGTCGGTAACACCCGAAGCCGGTGGCCTAACCCTTTTTTGGGAGGGAGCTGTCGAAGGTGGGACTGGTG<br>ATTAGGACT
15	Granulicatella sp. OTU_19	GACGAACGCTGGCGGCGTGCCTAATACATGCAAGTCGAACGAGAGCGACCGGTGCTTGCACTGGTCAATCTAGTGGCGAA<br>CGGGTGAGTAACACGTGGGTAACCTGCCCATCAGAGGGGGATAACATTCGGAAACGGATGCTAAAACCGCATAGGTCCTTT<br>AACCGCATGGTTGAAGGAGGAAAAGAGGCGCAAGCTTCTGCTGATGGATGGACCCGCGGTGCATTAGCTAGTTGGTGAGG<br>TAACGGCTCACCAAGGCCGTGATGCATAGCCGACCTGAGAGGGTGATCGGCCACATTGGGACTGAGACACGGCCCAAAC<br>TCCTACGGGAGGCAGCAGTAGGGAATCTTCCGCAATGGACGCAAGTCTGACGGAGCAACGCCGCGTGAGTGAAGAAGGTT<br>TTCGGATCGTAAAACTCTGTTGTTAGAGAAGAACAAGTGCTAGAGTAACTGTTAGCGCCTTGACGGTATCTAACCAGAAAGC<br>CACGGCTAACTACGTGCCAGCAGCCGCGGTAATACGTAGGTGGCAAGCGTTGTCCGGATTTATTGGGCGTAAAGCGAGCG<br>CAGGCGGTTCCTTAAGTCTGATGTGAAAGCCCCCGGCTCAACCGGGGAGGGTCATTGGAAACTGGGGAACTTGAGTGCAG<br>AAGAGGAGAGTGGAATTCCATGTGTAGCGGTGAAATGCGTAGATATATGGAGGAACACCAGTGGCGAAGGCGACTCTCTGG<br>TCTGTAACTGACGCTGAGGCTCGAAAGCGTGGGTAGCAAACAGGATTAGATACCCTGGTAGTCCACGCCGTAAACGATGAG<br>TGCTAAGTGTTGGAGGGTTTCCGCCCTTCAGTGCTGCAGTTAACGCATTAAGCACTCCGCCTGGGGAGTACGACCGCAAG<br>GTTGAAACTCAAAGGAATTGACGGGGACCCGCACAAGCGGTGGAGCATGTGGTTTAATTCGAAGCAACGCGAAGAACCTTA<br>CCAAGTCTTGACATCCTTTGACCACTCTAGAGATAGAGCTTTCCCTTCGGGGACAAAGTGACAGGTGGTGCATGGTTGTCG<br>TCAGCTCGTGTCGTGAGATGTTGGGTTAAGTCCCGCAACGAGCGCAACCCTTATTACTAGTTGCCAGCATTGAGTTGGGCA<br>CTCTAGTGAGACTGCCGGTGACAAACCGGAGGAAGGTGGGGATGACGTCAAATCATCATGCCCCTTATGACTTGGGCTAC<br>ACACGTGCTACAATGGATGGTACAACGAGCAGCGAACTCGCGAGGGTAAGCGAATCTCTTAAAGCCATTCTCAGTTCGGAT<br>TGTAGGCTGCAACTCGCCTACATGAAGCCGGAATCGCTAGTAATCGCGGATCAGCACGCCGCGGTGAATACGTTCCCGG<br>GTCTTGTACACACCGCCCGTCACACCACGAGAGTTTGTAACACCCAAAGTCGGTGAGGTAACCTTTTGGAGCCAGCCGCC<br>TAAGGTGGGATAGATGATTGGGGTG
16	Prevotella sp. OTU_20	GATGAACGCTAGCTACAGGCTTAACACATGCAAGTCGAGGGGAAACGGCATAGAGTGCTTGCACTCTTTGGACGTCGACC<br>GGCGCACGGGTGAGTAACGCGTATCCAACCTTCCCATTACTGTGGGATAACCTGCCGAAAGGCAGACTAATACCGCATGG<br>TCTTCGATGACGGCATCAGATTTGAAGTAAAGATTTATCGGTAATGGATGGGGATGCGTCTGATTAGCTTGTTGGCGGGGTA<br>ACGGCCCACCAAGGCGACGATCAGTAGGGGTTCTGAGAGGAAGGTCCCCCACATTGGAACTGAGACACGGTCCAAACTC<br>CTACGGGAGGCAGCAGTGAGGAATATTGGTCAATGGGCGAGAGCCTGAACCAGCCAAGTAGCGTGCAGGATGACGGCCC<br>TATGGGTTGTAAACTGCTTTTGTATGGGGATAAAGTCAGTCACGTGTGACTGTTTGCAGGTACCATACGAATAAGGACCGGC<br>TAATTCCGTGCCAGCAGCCGCGGTAATACGGAAGGTCCAGGCGTTATCCGGATTTATTGGGTTTAAAGGGAGCGTAGGCC<br>GGAGATTAAGTGTGTTGTGAAATGTAGACGCTCAACGTCTGACTTGCAGCGCATACTGGTTTCCTTGAGTACGCACAACGTT<br>GGCGGAATTCGTCGTGTAGCGGTGAAATGCTTAGATATGACGAAGAACTCCGATTGCGAAGGCAGCTGACGGGAGCGCAA<br>CTGACGCTTAAGCTCGAAGGTGCGGGTATCAAACAGGATTAGATACCCTGGTAGTCCGCACAGTAAACGATGGATGCCCG<br>CTGTTGGTACCTGGTATCAGCGGCTAAGCGAAAGCATTAAGCATCCCACCTGGGGAGTACGCCGGCAACGGTGAAACTCA<br>AAGGAATTGACGGGGGCCCGCACAAGCGGAGGAACATGTGGTTTAATTCGATGATACGCGAGGAACCTTACCCGGGCTTG<br>AATTGCAGAGGAAGAATTTAGAGATAATGACGCCCTTCGGGGTCTCTGTGAAGGTGCTGCATGGTTGTCGTCAGCTCGTGC<br>CGTGAGGTGTCGGCTTAAGTGCCATAACGAGCGCAACCCCTCTCTTCAGTTGCCATCAGGTGATGCTGGGCACTCTGGAG<br>ACACTGCCACCGTAAGGTGTGAGGAAGGTGGGGATGACGTCAAATCAGCACGGCCCTTACGTCCGGGGCTACACACGTG<br>TTACAATGGCCGGTACAGAGGGAGGGTGTAATGTAAATTGCATCAAATCTTGAAAGCCGGTCCCAGTTCGGACTGGGGTCT<br>GCAACCCGACCCCACGAAGCTGGATTCGCTAGTAATCGCGCATCAGCCATGGCGCGGTGAATACGTTCCCGGGCCTTGT<br>ACACACCGCCCGTCAAGCCATGAAAGCCGGGGGTGCCTGAAGTCCGTGACCGCAAGGATCGGCCTAGGGCAAAACTGGT<br>GATTGGGGCT

Sr no.	Species_OTUs	16S reference sequence
17	Filifactor sp. OTU_21	GATGAACGCTGGCGGCGTGCTTAACACATGCAAGTCGAACGAACGACTATTAACAGAACCTTTCGGGGCGAAGATGATAGA<br>AGTTAGTGGCGGACGGGTGCGTAACGCGTGGGTAATCTGCCTTTGTCAAAGGAATAACTATTCGAAAGAATAGCTAAAACCG<br>TATAACATATTAATAGGGCATCCTAATAATATCAAAACGATAGTGGACAAAGATGAGCCCGCGTCTGATTAGCTAGTTGGTGA<br>GATAAAAGCCCACCAAGGCAACGATCAGTAGCCGACCTGAGAGGGTGAACGGCCACATTGGAACTGAGACACGGTCCAAA<br>CTCCTACGGGAGGCAGCAGTGGGGAATATTGCACAATGGGGGGAACCCTGATGCAGCAACGCCGCGTGAGTGAAGAAGG<br>CATTCGTGTCGTAAAACTCTGTAGTAGGGGAAGAAAGAAATGACAGTACCCTAAAAGAAAGCCCCGGCTAACTACGTGCCAG<br>CAGCCGCGGTAATACGTAGGGGGCAAGCGTTATCCGGAATAACTGGGCGTAAAGGGTGCGCAGGTGGTTTAACAAGTTAG<br>TGGTGAAAGGCATAGGCTCAACCAATGTAAGCCATTAAAACTGTTTAACTTGAGTGCAGGAGAGGAAAGTGGAATTCCTAGT<br>GTAGCGGTGAAATGCGTAGATATTAGGAGGAATACCAGTGGCGAAGGCGACTTTCTGGACTGCAACTGACACTGAGGCACG<br>AAAGCGTGGGGAGCAAACAGGATTAGATACCCTGGTAGTCCACGCCGTAAACGATGAGTACTAGGTGTCGGGTGAAGAACT<br>CGGTGCCGAAGTTAACACATTAAGTACTCCGCCTGGGGAGTACGCTCGCAAGAGTAAAACTCAAAGGAATTGACGGGGACC<br>CGCACAAGCAGCGGAGCATGTGGTTTAATTCGAAGCAACGCGAAGAACCTTACCTAAACTTGACATACCAATGACAGCCTTT<br>TAATCGAGGACTTTTACCTTCGGGTAGACAATGGATACAGGTGGTGCATGGTTGTCGTCAGCTCGTGTCGTGAGATGTTGG<br>GTTAAGTCCCGCAACGAGCGCAACCCTTGTCGATAGTTACCAGCAGTAAGATGGGGACTCTATCGAGACAGCTAAGGACAA<br>CTTAGAGGAAGGTGGGGATGACGTCAAATCATCATGCCCCTTATGTTTAGGGCTACACACGTGCTACAATGGTCATTACAGA<br>GAGAAGCGAAACTGCGAAGTCAAGCAAACCTCAAAAAGATGATCCAAGTTCGGATTGTAGGCTGAAACTCGCCTACATGAAG<br>TTGGAGTTGCTAGTAATCGCGAATCAGAATGTCGCGGTGAATGCGTTCCCGGGTCTTGTACACACCGCCCGTCACACCAT<br>GGGAGTTTGGGGGGCCCAAAGTCAGCGACTCAACCGCAAGGAGAGAGCTGCCCAAGGCAAAACAAATGACTGGGGTG
18	Eggerthia sp. OTU_22	GATGAACGCTGGCGGCGTGCCTAATACATGCAAGTCGAACGGGTTGCCATAAGGCAGCCAGTGGCGAACGGGTGAGGAG<br>CACATGGGGAACCTGCCCGTCAGAGGGGGACAACAGCTGGAAACGGCTGCTAAGACCGCATAGGCTCAAGGAGGGCATC<br>CTCGTTGAGTTAAAGTGTCCTTTCGGGGACAGCTGACGGATGGACCTGTGGCGCATTAGCCAGTTGGCGGGGTAAAGGCC<br>CACCAAAGCGACGATGCGTAGCCGGCCTGAGAGGGCGGACGGCCACACTGGGACTGAGACACGGCCCAGACTCCTGCG<br>GGAGGCAGCAGTAGGGAATTTTCGGCAATGGGGGGAACCCTGACCGAGCAACGCCGCGTGAGTGAGGAAGGCCTTCGGG<br>CTGTAAAGCTCTGTTGCAGAGGAAGAACGGGAGAGCGAGGAAATGCGCTTTTCGTGACGGTACTCTGCGAGGAAGCCACG<br>GCTAACTACGTGCCAGCAGCCGCGGTAATACGTAGGTGGCAAGCGTTATCCGGAATCATTGGGCGTAAAGAGGGAGCAGG<br>CGGACGCGAAGGTCCGTAGTAAAAGCCCGAAGCTAAACTTCGGGCGGCTATGGAAACCGCGCGCCTGGAGTGCGGAAGA<br>GGATCGCGGAATTCCATGTGTAGCGGTGAAATGCGTAGATATATGGAGGAACACCGGTGGCGAAGGCGGCGGTCTGGTCC<br>GGCACTGACGCTCATTCCCGAAAGCGTGGGGAGCAAATAGGATTAGATACCCTAGTAGTCCACGCTGTAAACGATCGATAC<br>CAGGTGTCGGGAGTCACATCCCGGTGCCGAAGCCAACGCATTAAGTATCGCGCCTGAGTAGTACGTCCGCAAGGATGAAA<br>CTCAAAGGAATTGACGGGGGCCCGCACAAGCGGTGGAGCATGTGGTTTAATTCGAAGCAACGCGAAGAACCTTACCAGGC<br>CTTGACATCGATCCGATCGTGCGGAGACGCGCGAGACCTTGCCGAAAGGGAAGGAGGAGAAGACAGGTGGTGCATGGTT<br>GTCGTCAGCTCGTGTCGTGAGATGTTGGGTTAAGTCCCGCAACGAGCGCAACCCCTGCCGCCAGTTACCAGCATTAAGGA<br>TGGGGACTCTGGCGGGACTGCCCCTGTAAGGGGGAGGAAGGCGGGGATGACGTCAAATCATCATGCCCCTTATGGCCTG<br>GGCTACACACGTGCTACAATGGACGGAGCAGAGGGAGGCGAAGCCGCGAGGCGGAGCGGAGCCCAGAAACCCGTTCAC<br>AGTTCGGACTGCAGCCTGCAACTCGGCTGCACGAAGCTGGAATCGCTAGTAATCGCAGATCAGCATGCTGCGGTGAATAC<br>GTTCCCGGGCCTTGTACACACCGCCCGTCACACCATGAGAGTCGGCAACGCCCGAAGCCGGCGGCCCAACCCCGCAAG<br>GGGAGGGAGCTGTCGAAGGCGGGGCTGATGATTGGGGTG
19	Caviibacter sp. OTU_23	GATAAACGCTGACAGAATGCTTAACACATGCAAGTCGATGACTAAGTATAGCTTGCTATACGAGGTCATGGCGGACGGGTGA<br>GTAACGTGTAAAGAACTTACCTTATAGACTGGGATAACAGATAGAAATGTCTGATAATACTAGATACTATTACACTGTGGCATC<br>ACAGAGTAATGAAAGAATACGCTATAAGAGAGCTTTGCATCCTATTAGCTAGTTGGTAAGGTAAAGGCTTACCAAGGCGATGA<br>TAGGTAGCCGGCCTGAGAGGGTGGACGGCCACAAGGGGACTGAGATACGGCCCTTACTCCTACGGGAGGCAGCAGTGG<br>GGAATATTGGACAATGGAGGAAACTCTGATCCAGCAATTCTGTGTGTGTGAAGAAGGTCTTCGGATTGTAAAACACTTTTAGT<br>TGGGAAGAAACAAATGACGGTACCAACAGAAGAAGCGACGGCTAAATACGTGCCAGCAGCCGCGGTAATACGTATGTCGCA<br>AGCGTTATCCGGAATTATTGGGCTTAAAGGGCATCTAGGCGGTATAACAAGTTGAAGGTGAAAAACTGTAGCTCAACTATAGT<br>CTTGCCTACAAAACTGTAATACTAGAGTACTGGAAAGGTGGGTGGAACTACACGAGTAGAGGTGAAATTCGTAGATATGTGTA<br>GGAATGCCGATGATGAAGATAACTCACTGGACAGAAACTGACGCTGAAGTGCGAAAGCTAGGGGAGCAAACAGGATTAGATA<br>CCCTGGTAGTCCTAGCTGTAAACGATGATTACTGGGTGTGGGGATGAGAAGTCTCTGTGCCGAAGCAAAAGCGATAAGTAA<br>TCCGCCTGGGGAGTACGTTCGCAAGAATGAAACTCAAAGGAATTGACGGGGGCCCGCACAAGTGGTGGAGCATGTGGTTT<br>AATTCGACGCAACGCGAGGAACCTTACCAGACCTTGACATCGCCGGGAGGTCATAGAAGTATGACTGTGCCTTCGGGAAC<br>CGGTAGACAGGTGGTGCATGGCTGTCGACAGCTCGTGTTGTGAGATGTTGGGTTAAGTCCCGCAACGAGCGAAACCCCTA<br>TCATTAGTTGCCATCATTAAGTTGGGGACTCTAATGAAACTGCCTGCGAAGAGCAGGAGGAAGGTGGGGATGACGTCAAGT<br>CATCATGCCCCTTATGGTCTGGGCTACACACGTGCTACAATGGGTAGTACAACGAGAAGCGATATGGCGACATAAAGCAAA<br>CCTTAAAAACTACTCTAAGTTCGGATTGAAGTCTGCAACTCGACTTCATGAAGTTGGAATCACTAGTAATCGCAAATCAGCAA<br>TGTTGCGGTGAATACGTTCTCGGGCCTTGTACACACCGCCCGTCACACCACGAGAGTTGTTTGCACCTGAAATTACTGGC<br>CTAACCGTAAGGAGGGAGGTAATGAAGGTGTGAATAGTGATTGGGGTG
20	Desulfovibrio sp. OTU_24	ATTGAACGCTGGCGGCGTGCTTAACACATGCAAGTCGAACGTGAAAGGGGCTTCGGCCCCGAGTAAAGTGGCGCACGGG<br>TGAGTAACGCGTGGACAATCTGCCCTTATGAGTGGGATAACAGTTGGAAACGACTGCTAATACCGCATACGCTCAGGAATG<br>ACTTTTTGAGGAAAGGCGGCCTCTGCATGCAAGCTGTCGCATAAGGATGAGTCCGCGTCCCATTAGCTTGTTGGTGGGGT<br>AACGGCCTACCAAGGCAACGATGGGTAGCCGATTTGAGAGGATGATCGGCCACACTGGAACTGAAACACGGTCCAGACTC<br>CTACGGGAGGCAGCAGTGGGGAATATTGCGCAATGGGCGAAAGCCTGACGCAGCGACGCCGCGTGAGGGATGAAGGTTT<br>TCGGATCGTAAACCTCTGTCGGAAGGGAAGAAGGTGCACTCTGCTAATCAGGGGTGTATTGACGGTACCTTCAAAGGAAGC<br>ACCGGCTAACTCCGTGCCAGCAGCCGCGGTAATACGGAGGGTGCGAGCGTTAATCGGAATTACTGGGCGTAAAGCGCAC<br>GTAGGCTGTCATGTAAGTCAGGGGTGAAATCCCACGGCTCAACCGTGGAACTGCCCTTGATACTGCGTGACTTGAATCCG<br>GGAGAGGGTGGCGGAATTCCAGGTGTAGGAGTGAAATCCGTAGATATCTGGAGGAACATCAGTGGCGAAGGCGGCCACCT<br>GGACCGGTATTGACGCTGAGGTGCGAAAGCGTGGGGAGCAAACAGGATTAGATACCCTGGTAGTCCACGCTGTAAACGAT<br>GGATGCTAGATGTCGGGGAGTATTCTTCGGTGTCGTAGTTAACGCGTTAAGCATCCCGCCTGGGGAGTACGGTCGCAAGG<br>CTGAAACTCAAAGAAATTGACGGGGGCCCGCACAAGCGGTGGAGTATGTGGTTTAATTCGATGCAACGCGAAGAACCTTAC<br>CTGGGTTTGACATCCACGGAACCTTCCGGAAATGGAGGGGTGCCCTTCGGGGAGCCGTGAGACAGGTGCTGCATGGCTG<br>TCGTCAGCTCGTGTCGTGAGATGTTGGGTTAAGTCCCGCAACGAGCGCAACCCCTATGCATAGTTGCCATCAAGTGAAGTT<br>GGGCACTCTATGCAGACTGCCCGGGTTAACCGGGAGGAAGGTGGGGACGACGTCAAGTCATCATGGCCCTTACGCCCAG<br>GGCTACACACGTACTACAATGGCGCGCACAAAGGGGAGCGAGACCGCGAGGTGGAGCCAATCCCAAAAAACGCGTCCCA<br>GTCCGGATTGCAGTCTGCAACTCGACTGCATGAAGTCGGAATCGCTAGTAATTCGAGATCAGCATGCTCGGGTGAATGCGT<br>TCCCGGGCCTTGTACACACCGCCCGTCACACCACGAAAGTCGGTTTTACCCGAAGCCGGTGGGCCAACCAGCAATGGAG<br>GCAGCCGTCTACGGTAGGGCTGATGATTGGGGTG

Sr no.	Species_OTUs	16S reference sequence
21	Actinomyces sp. OTU_26	GACGAACGCTGGCGGCGTGCTTAACACATGCAAGTCGAACGGTGAAGGGATTGGCTTTTGTTGGTCCTGGATGAGTGGCG<br>AACGGGTGAGTAACACGTGAGTAACCTGCCCCCTTCTTCTGGATAACCGCATGAAAGTGTGGCTAATACGGGATATTCTGG<br>GTCTGTCGCATGGCGGGCCCGGGAAAGATATTGTTTCGGTGGGGGATGGGCTCGCGGCCTATCAGCTTGTTGGTGGGGT<br>GATGGCCTACCAAGGCGGTGACGGGTAGCCGGCCTGAGAGGGTGGACGGTCACACTGGGACTGAGACACGGCCCAGAC<br>TCCTACGGGAGGCAGCAGTGGGGAATATTGCACAATGGGCGCAAGCCTGATGCAGCGACGCCGCGTGAGGGATGGAGGC<br>CTTCGGGTTGTAAACCTCTTTCGCCAGTGAAGCAGGCCTGCTCCTTGTGGGTGGGTTGACGGTAGCTGGATAAGAAGCGC<br>CGGCTAACTACGTGCCAGCAGCCGCGGTAATACGTAGGGCGCGAGCGTTGTCCGGAATTATTGGGCGTAAAGAGCTCGTA<br>GGCGGCTGGTCGCGTCTGTCGTGAAATCCTCTGGCTTAACTGGGGGCTTGCGGTGGGTACGGGCCGGCTTGAGTGCGG<br>TAGGGGAGACTGGAACTCCTGGTGTAGCGGTGGAATGCGCAGATATCAGGAAGAACACCGGTGGCGAAGGCGGGTCTCT<br>GGGCCGTTACTGACGCTGAGGAGCGAAAGCGTGGGGAGCGAACAGGATTAGATACCCTGGTAGTCCACGCCGTAAACGTT<br>GGGCACTAGGTGTGGGGGGCCTTTTCCGGGTCTTCCGCGCCGTAGCTAACGCATTAAGTGCCCCGCCTGGGGAGTACG<br>GCCGCAAGGCTAAAACTCAAAGGAATTGACGGGGGCCCGCACAAGCGGCGGAGCATGCGGATTAATTCGATGCAACGCGA<br>AGAACCTTACCAAGGCTTGACATGTGCCGGTCGGCTCCGGAGACGGGGCTTCCTCCTTGTGGGGCCGGTTCACAGGTGG<br>TGCATGGTTGTCGTCAGCTCGTGTCGTGAGATGTTGGGTTAAGTCCCGCAACGAGCGCAACCCTTGTCCCGTGTTGCCAG<br>CACGTTGTGGTGGGGACTCGCGGGAGACTGCCGGGGTCAACTCGGAGGAAGGTGGGGATGACGTCAAATCATCATGCCC<br>CTTATGTCTTGGGCTTCACGCATGCTACAATGGCCGGTACAGAGGGCTGCGATACCGTGAGGTGGAGCGAATCCCTTAAA<br>GCCGGTCTCAGTTCGGATCGGTGTCTGCAACTCGACACCGTGAAGTTGGAGTCGCTAGTAATCGCAGATCAGCAACGCTG<br>CGGTGAATACGTTCTCGGGCCTTGTACACACCGCCCGTCACGTCATGAAAGTCGGCAACACCCGAAGCCCGTGGCCCTA<br>CGGGGAGCGGTCGAAGGTGGGGCTGGTGATTGGGACG
22	Haemophilus sp. OTU_27	ATTGAACGCTGGCGGCAGGCTTAACACATGCAAGTCGAACGGTAGCAGGAGAAAGCTTGCTTTCTTGCTGACGAGTGGCG<br>GACGGGTGAGTAATGCTTGGGAATCTGGCTTATGGAGGGGGATAACTACGGGAAACTGTAGCTAATACCGCGTAATATCGA<br>AAGATTAAAGTGTGGGACCTTCGGGCCACATGCCATAAGATGAGCCCAAGTGGGATTAGGTAGTTGGTGGGGTAAAGGCCT<br>ACCAAGCCGACGATCTCTAGCTGGTCTGAGAGGATGACCAGCCACACTGGAACTGAGACACGGTCCAGACTCCTACGGGA<br>GGCAGCAGTGGGGAATATTGCGCAATGGGGGGAACCCTGACGCAGCCATGCCGCGTGAATGAAGAAGGCCTTCGGGTTG<br>TAAAGTTCTTTCGGTATTGAGGAAGGGATGTGTGCTAATAGTACACGTCATTGACGTTAAATACAGAAGAAGCACCGGCTAAC<br>TCCGTGCCAGCAGCCGCGGTAATACGGAGGGTGCGAGCGTTAATCGGAATAACTGGGCGTAAAGGGCACGCAGGCGGTT<br>ATTTAAGTGAGGTGTGAAAGCCCCGGGCTTAACCTGGGAATTGCATTTCAGACTGGGTAACTAGAGTACTTTAGGGAGGGG<br>TAGAATTCCACGTGTAGCGGTGAAATGCGTAGAGATGTGGAGGAATACCGAAGGCGAAGGCAGCCCCTTGGGAATGTACTG<br>ACGCTCATGTGCGAAAGCGTGGGGAGCAAACAGGATTAGATACCCTGGTAGTCCACGCTGTAAACGCTGTCGATTTGGGG<br>ATTGGGCTTTAAGCTTGGTGCCCGTAGCTAACGTGATAAATCGACCGCCTGGGGAGTACGGCCGCAAGGTTAAAACTCAAA<br>TGAATTGACGGGGGCCCGCACAAGCGGTGGAGCATGTGGTTTAATTCGATGCAACGCGAAGAACCTTACCTACTCTTGACA<br>TCCATGGAATCCTGTAGAGATACGGGAGTGCCTTCGGGAACCATGAGACAGGTGCTGCATGGCTGTCGTCAGCTCGTGTT<br>GTGAAATGTTGGGTTAAGTCCCGCAACGAGCGCAACCCTTATCCTTTGTTGCCAGCGATTCGGTCGGGAACTCAAAGGAGA<br>CTGCCAGTGATAAACTGGAGGAAGGTGGGGATGACGTCAAGTCATCATGGCCCTTACGAGTAGGGCTACACACGTGCTAC<br>AATGGCGTATACAGAGGGTGGCGAAACTGCGAGGTGGAGCGAATCTCAGAAAGTACGTCTAAGTCCGGATTGGAGTCTGCA<br>ACTCGACTCCATGAAGTCGGAATCGCTAGTAATCGCGAATCAGAATGTCGCGGTGAATACGTTCCCGGGCCTTGTACACAC<br>CGCCCGTCACACCATGGGAGTGGGTTGTACCAGAAGTAGATAGCTTAACCGCAAGGGGGGCGTTTACCACGGTATGATTC<br>ATGACTGGGGTG
23	Campylobacter sp. OTU_29	AGTGAACGCTGGCGGCGTGCCTAATACATGCAAGTCGAACGGAGATTAAGTAGCTTGCTATTTAATCTTAGTGGCGCACGG<br>GTGAGTAATATATAGCTAATCTGCCCTACACTAGAGGACAACAGTTGGAAACGACTGCTAATACTCTATACTCCTTCTAAGCA<br>TAAGCTTAGTCGGGAAAGTTAATAAAATTTGCTTTTTGGTTTCATCTTGAGAGATATGGACGTATAACAACTAAATTGATACGC<br>CGATGTGGCAAACATCGGTGTGCTTTAGGTGGGTCAAGGGAGCGCTTGCGCTTCTTGTCGCAAAGAAAGGCTTTGCCTTT<br>CTGCGAAGTCCGTATTTGTCAAGATGAAATCTTGCAAATGTATTTTTTCGGTGTAGGATGAGGCTATATCGTATCAGCTAGTT<br>GGTGAGGTAATGGCTCACCAAGGCTATGACGCGTAACTGGTCTGAGAGGATGATCAGTCACACTGGAACTGAGACACGGT<br>CCAGACTCCTACGGGAGGCAGCAGTAGGGAATATTGCTCAATGGGGGAAACCCTGAAGCAGCAACGCCGCGTGGAGGAT<br>GACACTTTTCGGAGCGTAAACTCCTTTTCTTGGGAAAGAATTATGACGGTACCCAAGGAATAAGCACCGGCTAACTCCGTG<br>CCAGCAGCCGCGGTAATACGGAGGGTGCAAGCGTTACTCGGAATCACTGGGCGTAAAGGACGCGTAGGCGGATTATCAAG<br>TCTCTTGTGAAATCTAACGGCTTAACCGTTAAACTGCTTGGGAAACTGATAATCTAGAGTAAGGGAGAGGCAGATGGAATTCT<br>TGGTGTAGGGGTAAAATCCGTAGAGATCAAGAAGAATACCCATTGCGAAAGCGATCTGCTGGAACTTAACTGACGCTAATGC<br>GTGAAAGCGTGGGGAGCAAACAGGATTAGATACCCTGGTAGTCCACGCCCTAAACGATGTATACTAGTTGTTGCTTCGCTA<br>GTCGAGGCAGTAATGCACCTAACGGATTAAGTATACCGCCTGGGGAGTACGGTCGCAAGATTAAAACTCAAAGGAATAGAC<br>GGGGACCCGCACAAGCGGTGGAGCATGTGGTTTAATTCGAAGATACGCGAAGAACCTTACCCGGACTTGATATCTAACAAA<br>TCATCCAGAGATGGAAGAGTGTCTGCTTGCAGAAATGTTAAGACAGGTGCTGCACGGCTGTCGTCAGCTCGTGTCGTGAGA<br>TGTTGGGTTAAGTCCCGCAACGAGCGCAACCCACGTATTTAGTTGCTAACGGTTAGGCCGAGCACTCTAAATAGACTGCCT<br>TCGTAAGGAGGAGGAAGGTGTGGACGACGTCAAGTCATCATGGCCCTTATGTCCGGGGCGACACACGTGCTACAATGGCG<br>TATACAATGAGACGCAATATCGCGAGATGGAGCAAATCTATAAAATACGTCCCAGTTCGGATTGGAGTCTGCAACTCGACTC<br>CATGAAGCCGGAATCGCTAGTAATCGTAGATCAGCCATGCTACGGTGAATACGTTCCCGGGTCTTGTACTCACCGCCCGT<br>CACACCATGGGAGTTGATTTCACTCGAAGCCCAAATACCAAACCGGTTATGGTCCACAGTGGAATCAGCGACTGGGGTG
24	Limosilactobacillus sp. OTU_30	GATGAACGCCGGCGGTGTGCCTAATACATGCAAGTCGTACGCACTGGCCCAACTGATTGATGGTGCTTGCACCTGATTGA<br>CGATGGATCACCAGTGAGTGGCGGACGGGTGAGTAACACGTAGGTAACCTGCCCCGGAGCGGGGGATAACATTTGGAAAC<br>AGATGCTAATACCGCATAACAACAAAAGCCGCATGGTTTTTGTTTGAAAGATGGCTTTGGCTATCACTCTGGGATGGACCTG<br>CGGTGCATTAGCTAGTTGGTAAGGTAACGGCTTACCAAGGCGATGATGCATAGCCGAGTTGAGAGACTGATCGGCCACAAT<br>GGAACTGAGACACGGTCCATACTCCTACGGGAGGCAGCAGTAGGGAATCTTCCACAATGGGCGCAAGCCTGATGGAGCAA<br>CACCGCGTGAGTGAAGAAGGGTTTCGGCTCGTAAAGCTCTGTTGTTGGAGAAGAACGTGCGTGAGAGTAACTGTTCACGCA<br>GTGACGGTATCCAACCAGAAAGTCACGGCTAACTACGTGCCAGCAGCCGCGGTAATACGTAGGTGGCAAGCGTTATCCGG<br>ATTTATTGGGCGTAAAGCGAGCGCAGGCGGTTGCTTAGGTCTGATGTGAAAGCCTTCGGCTTAACCGAAGAAGTGCATCGG<br>AAACCGGGCGACTTGAGTGCAGAAGAGGACAGTGGAACTCCATGTGTAGCGGTGGAATGCGTAGATATATGGAAGAACACC<br>AGTGGCGAAGGCGGCTGTCTGGTCTGCAACTGACGCTGAGGCTCGAAAGCATGGGTAGCGAACAGGATTAGATACCCTGG<br>TAGTCCATGCCGTAAACGATGAGTGCTAGGTGTTGGAGGGTTTCCGCCCTTCAGTGCCGGAGCTAACGCATTAAGCACTC<br>CGCCTGGGGAGTACGACCGCAAGGTTGAAACTCAAAGGAATTGACGGGGGCCCGCACAAGCGGTGGAGCATGTGGTTTAA<br>TTCGAAGCTACGCGAAGAACCTTACCAGGTCTTGACATCTTGCGCTAACCTTAGAGATAAGGCGTTCCCTTCGGGGACGCA<br>ATGACAGGTGGTGCATGGTCGTCGTCAGCTCGTGTCGTGAGATGTTGGGTTAAGTCCCGCAACGAGCGCAACCCTTGTTA<br>CTAGTTGCCAGCATTGAGTTGGGCACTCTAGTGAGACTGCCGGTGACAAACCGGAGGAAGGTGGGGACGACGTCAGATCA<br>TCATGCCCCTTATGACCTGGGCTACACACGTGCTACAATGGACGGTACAACGAGTCGCAAACTCGCGAGAGTAAGCTAATC<br>TCTTAAAGCCGTTCTCAGTTCGGACTGTAGGCTGCAACTCGCCTACACGAAGTCGGAATCGCTAGTAATCGCGGATCAGCA<br>TGCCGCGGTGAATACGTTCCCGGGCCTTGTACACACCGCCCGTCACACCATGGGAGTTTGTAACGCCCAAAGTCGGTGG<br>CCTAACCTTTATGGAGGGAGCCGCCTAAGGCGGGACAGATGACTGGGGTG

Sr no.	Species_OTUs	16S reference sequence
25	Veillonellaceae gen. sp. OTU_33	GACGAACGCTGGCGGCGTGCTTAACACATGCAAGTCGAACGAGAGACGGTGAAAAGCTTGCTTTTAATCGAATCGAGTGGCAAACGGGTGAGTAACGCGTAAACAACCTGCCCCTGGGATGGGGACAACAGCTGGAAACGGCTGCTAATACCGAATGAGCTCCACGTGCCGCATGGCAGGAGGAGGAAAGGGTGGCCTCTGGAACAAGCTACCGCCGAGGGAGGGGTTTGCGTCTGATTAGCTGGTTGGTGAGGTAAGGGCTCACCAAGGCGACGATCAGTAGCCGGTCTGAGAGGATGAACGGCCACATTGGAACTGAGAGACGGTCCAGACTCCTACGGGAGGCAGCAGTGGGGAATCTTCCGCAATGGGCGAAAGCCTGACGGAGCAACGCCGCGTGAGTGAAGACGGCCTTCGGGTTGTAAAGCTCTGTTACACGGGACGAACGGGTTTTGTGCGAACAGCATAAGACCGTGACGGTACCGTAGAAGAAAGCCACGGCTAACTACGTGCCAGCAGCCGCGGTAATACGTAGGTGGCAAGCGTTGTCCGGAATGATTGGGCGTAAAGGGCGCGCAGGCGGCCGGCCAAGTCTGTCTAAAAAGTGCGGGGCTTAACCCCGTGAGGGGAGAGAAACTGGTTGGCTGAGAGTATCGGAGAGGAAAGCGGAATTCCTAGTGTAGCGGTGAAATGCGTAGATATTAGGAGGAACACCGGTGGCGAAAGCGGCTTTCTGGACGACAACTGACGCTGAGGCGCGAAAGCCAGGGGAGCAAACGGGATTAGATACCCCGGTAGTCCTGGCCGTAAACGATGGGTACCAGGTGTGGGAGGTATCGACCCCTTCCGTGCCGGAGTTAACGCAATAAGTACCCCGCCTGGGGAGTACGGCCGCAAGGTTGAAACTCAAAGGAATTGACGGGGGCCCGCACAAGCGGTGGAGTATGTGGTTTAATTCGACGCAACGCGAAGAACCTTACCAAGCCTTGACATTGAGTGAAGGGCGTAGAGATATGCCGTTCTTCTTCGGAAGACACGAAAACAGGTGGTGCACGGCTGTCGTCAGCTCGTGTCGTGAGATGTTGGGTTAAGTCCCGCAACGAGCGCAACCCTTATCTTCAGTTACCAGCACGTAGAGGTGGGGACTCAGGAGAGACTGCCGCAGACAATGCGGAGGAAGGCGGGGATGACGTCAAGTCATCATGCCCCTAATGGCTTGGGCTACACACGTACTACAATGGCTCTAAATAGAGGGAAGCAAGGGAGCGATCCGGAGCAAACCCCAAAAACAGAGTCCCAGTTCGGATTGCAGGCTGCAACTCGCCTGCATGAAGGAGGAATCGCTAGTAATCGCAGGTCAGCATACTGCGGTGAATACGTTCCCGGGCCTTGTACACACCGCCCGTCACACCACGAAAGTCATTCACACCCGAAGCCGGTGAGGTAACCGCAAGGAGCCAGCCGTCGAAGGTGGGGGCGATGATTGGGGTG
26	Haemophilus sp. OTU_34	ATTGAACGCTGGCGGCAGGCTTAACACATGCAAGTCGAACGGTAACAGGAATTAGCTTGCTAATTTGCTGACGAGTGGCGGACGGGTGAGTAATGCTTGGGAATCTGGCTTATGGAGGGGGATAACTACGGGAAACTGTAGCTAATACCGCGTAAAATCTTCGGATTAAAGGGTGGGACTTTCGAGCCACCTGCCATAAGATGAGCCCAAGTGGGATTAGGTAGTTGGTTAGGTAAAGGCTGACCAAGCCGACGATCTCTAGCTGGTCTGAGAGGATGACCAGCCACACTGGAACTGAGACACGGTCCAGACTCCTACGGGAGGCAGCAGTGGGGAATATTGCACAATGGGGGGAACCCTGATGCAGCCATGCCGCGTGAATGAAGAAGGCCTTCGGGTTGTAAAGTTCTTTCGGTGACGAGGAAGGTTGATAGGTTAATAGCCTATCAAATTGACGTTAATCACAGAAGAAGCACCGGCTAACTCCGTGCCAGCAGCCGCGGTAATACGGGGGGTGCGAGCGTTAATCGGAATAACTGGGCGTAAAGGGCACGCAGGCGGTGACTTAAGTGAGATGTGAAAGCCCCGAGCTTAACTTGGGAATTGCATTTCATACTGGGTCGCTAGAGTACTTTAGGGAGGGGTAGAATTCCACGTGTAGCGGTGAAATGCGTAGAGATGTGGAGGAATACCGAAGGCGAAGGCAGCCCCTTGGGAATGTACTGACGCTCATGTGCGAAAGCGTGGGGAGCAAACAGGATTAGATACCCTGGTAGTCCACGCTGTAAACGATGTCGATTTGGGGATTGGGCTTTGAGCTTGGTGCCCGTAGCTAACGTGATAAATCGACCGCCTGGGGAGTACGGCCGCAAGGTTAAAACTCAAATGAATTGACGGGGGCCCGCACAAGCGGTGGAGCATGTGGTTTAATTCGATGCAACGCGAAGAACCTTACCTACTCTTGACATCCATGGAATCTTGTAGAGATATGAGAGTGCCTTCGGGAACCATGAGACAGGTGCTGCATGGCTGTCGTCAGCTCGTGTTGTGAAATGTTGGGTTAAGTCCCGCAACGAGCGCAACCCTTATCCTTTGTTGCCAGCGATTAGGTCGGGAACTCAAAGGAGACTGCCAGTGATAAACTGGAGGAAGGTGGGGATGACGTCAAGTCATCATGGCCCTTACGAGTAGGGCTACACACGTGCTACAATGGCGTATACAGAGGGAAGCGAGCCTGCGAGGGGGAGCAAATCTCAGAAAGTACGTCTAAGTCCGGATTGGAGTCTGCAACTCGACTCCATGAAGTCGGAATCGCTAGTAATCGCGAATCAGAATGTCGCGGTGAATACGTTCCCGGGCCTTGTACACACCGCCCGTCACACCATGGGAGTGGGTTGTACCAGAAGTAGATAGCTTAACCTTCGGGAGGGCGTTTACCACGGTATGATTCATGACTGGGGTG
27	Capnocytophaga sp. OTU_36	GATGAACGCTAGCGGCAGGCCTAACACATGCAAGTCGAGGGAGAAGCCTTTCGGGGCAGAAACCGGCGCACGGGTGCGTAACGCGTATGCAACCTACCTTTTACAGGGGGATAGCCCGAAGAAATTTGGATTAATACCCCATAATATTATTGTACGACATCGTTTGATAATTAAAGCTGAGGCGGTAGAAGATGGGCATGCGTCCTATTAGCTAGTTGGAGTGGTAACGGCACCCCAAGGCTACGATAGGTAGGGGTCCTGAGAGGGAGATCCCCCACACTGGTACTGAGACAGGGACCAGACTCCTACGGGAGGCAGCAGTGAGGAATATTGGTCAATGGTCGGAAGACTGAACCAGCCATGCCGCGTGCAGGAAGAATGCCTTATGGGTTGTAAACTGCTTTTATATAGGGAGAATAAGGAGTACGTGTACTCTGATGACGGTACTATATGAATAAGCATCGGCTAACTCCGTGCCAGCAGCCGCGGTAATACGGAGGATGCGAGCGTTATTCGGAATCATTGGGTTTAAAGGGTCTGTAGGCGGGCTATTAAGTCAGGGGTGAAATCTTACAGCTTAACTGTGAAATTGCCTTTGATACTGGTAGTCTTGAATATTTGTGAAGTTTTTGGAATGTGTAGTGTAGCGGTGAAATGCTTAGATATTACACAGAACACCGATTGCGAAGGCAGGAGACTAACAAATGATTGACGCTGAGAGACGAGAGCGTGGGGAGCGAACAGGATTAGATACCCTGGTAGTCCACGCTGTAAACGATGGATACTAGCTGTTTGGCGTAAGCTGAGTGGCTAAGCGAAAGTGATAAGTATCCCACCTGGGGAGTACGCACGCAAGTGTGAAACTCAAAGGAATTGACGGGGGCCCGCACAAGCGGTGGAGCATGTGGTTTAATTCGATGATACGCGAGGAACCTTACCAAGGTTTAAATGGGAACTGACGTACTTAGAGATAGGTATTTCTTCGGACAGTTTTCAAGGTGCTGCATGGTTGTCGTCAGCTCGTGCCGTGAGGTGTCAGGTTAAGTCCTATAACGAGCGCAACCCCTATTGTTAGTTACCAGCACGTGATGGTGGGGACTCTAGCAAGACTGCCGGTGTAAACCGTGAGGAAGGTGGGGATGACGTCAAATCATCACGGCCCTTACATCTTGGGCTACACACGTGCTACAATGGTCGTTACAGAGAGCAGCCACTGCGCGAGCAGGAGCGAATCTATAAAGACGGTCACAGTTCGGATCGGAGTCTGCAACTCGACTCCGTGAAGCTGGAATCGCTAGTAATCGGATATCAGCCATGATCCGGTGAATACGTTCCCGGGCCTTGTACACACCGCCCGTCAAGCCATGGAAGCTGGGGGTACCTGAAGTCGGTGACCGTAAGGAGCTGCCTAGGGTAAAACTAGTGACTGGGGCT
28	Bacteroidaceae gen. sp. OTU_37	GATGAACGCTAGCTACAGGCTTAACACATGCAAGTCGAGGGGCAGCATGGGCTTAGCTTGCTAAGCCCGATGGCGACCGGCGCACGGGTGAGTAACACGTATCCAACCTTCCGGTAACTCCGGTATAGCCTTTCGAAAGAAAGATTAATCCCGGATAGTATGGTGATTCCGCATGGTTTCTCCATTAAAGGATTCCGGTTACCGATGGGGATGCGTTCCATTAGGCAGTTGGCGGGGTAACGGCCCACCAAACCTACGATGGATAGGGGTTCTGAGAGGAAGGTCCCCCACATTGGAACTGAGACACGGTCCAAACTCCTACGGGAGGCAGCAGTGAGGAATATTGGTCAATGGGCGGAAGCCTGAACCAGCCAAGTAGCGTGAAGGATGACTGCCCTATGGGTTGTAAACTTCTTTTATAAGGGAATAATTGTGGGTACGCGTACCCATTTGCATGTACCTTATGAATAAGGATCGGCTAACTCCGTGCCAGCAGCCGCGGTAATACGGAGGATCCGAGCGTTATCCGGATTTATTGGGTTTAAAGGGAGCGTAGGCGGGCGCTTAAGTCAGCTGTGAAAGTTTGGGGCTCAACCTTAAAATTGCAGTTGATACTGGGTGCCTTGAGTGCAGTATAGGCAGGCGGAATTCGTGGTGTAGCGGTGAAATGCTTAGATATCACGAAGAACTCCTATTGCGAAGGCAGCTTGCTGGAGTGTAACTGACGCTGATGCTCGAAAGTGTGGGTATCAAACAGGATTAGATACCCTGGTAGTCCACACAGTAAACGATGAATACTCGCTGTTTGCGATATACTGCAAGCGGCCAAGCGAAAGCGTTAAGTATTCCACCTGGGGAGTACGCCGGCAACGGTGAAACTCAAAGGAATTGACGGGGGCCCGCACAAGCGGAGGAACATGTGGTTTAATTCGATGATACGCGAGGAACCTTACCCGGGCTTGAATTGCAACCGAATTATCCGGAAACGGGTAAGCCGCAAGGCGGTTGTGAAGGTGCTGCATGGTTGTCGTCAGCTCGTGCCGTGAGGTGTCGGCTTAAGTGCCATAACGAGCGCAACCCTTATCCACAGTTACCATCAGGTGATGCTGGGGACTCTGTGGAGACTGCCGTCGTAAGATGCGAGGAAGGGGGGGATGACGTCAAATCAGCACGGCCCTTACGTCCGGGGCTACACACGTGTTACAATGGGGGGTACAGCAGGCAGCTCGGCGGCGACGCCCGGCCAATCCCTAAATCCCCTCTCAGTTCGGACTGGAGTCTGCAACCCGACTCCACGAAGCTGGATTCGCTAGTAATCGCGCATCAGCCACGGCGCGGTGAATACGTTCCCGGGCCTTGTACACACCGCCCGTCAAGCCATGAAAGCCGGGGGTACCTGAAGTGCGTAACCGTCCAGGAGCGCCCTAGGGTAAAACCGGTGATTGGGGCT

Sr no.	Species_OTUs	16S reference sequence
29	Streptococcus sp. OTU_38	GATGAACGCTGGCGGCGTGCCTAATACATGCAAGTAGAACGCTGAAGCTTGGTGCTTGCACCGAGCGGATGAGTTGCGAA<br>CGGGTGAGTAACGCGTAGGTAACCTGCCTGGTAGCGGGGGATAACTATTGGAAACGATAGCTAATACCGCATAACAGTAGA<br>TATTGCATGATATCTGCTTGAAAGGGGCAATTGCTCCACTACCAGATGGACCTGCGTTGTATTAGCTAGTTGGTGAGGTAAC<br>GGCTCACCAAGGCGACGATACATAGCCGACCTGAGAGGGTGATCGGCCACACTGGGACTGAGACACGGCCCAGACTCCT<br>ACGGGAGGCAGCAGTAGGGAATCTTCGGCAATGGACGGAAGTCTGACCGAGCAACGCCGCGTGAGTGAAGAAGGTTTTCG<br>GATCGTAAAGCTCTGTTGTAAGAGAAGAACGAGTGTGAGAGTGGAAAGTTCACACTGTGACGGTATCTTACCAGAAAGGGAC<br>GGCTAACTACGTGCCAGCAGCCGCGGTAATACGTAGGTCCCGAGCGTTATCCGGATTTATTGGGCGTAAAGCGAGCGCAG<br>GCGGTTAGATAAGTCTGAAGTTAAAGGCTGTGGCTTAACCATAGTACGCTTTGGAAACTGTTTAACTTGAGTGCAAGAGGGG<br>AGAGTGGAATTCCATGTGTAGCGGTGAAATGCGTAGATATATGGAGGAACACCGGTGGCGAAAGCGGCTCTCTGGCTTGTA<br>ACTGACGCTGAGGCTCGAAAGCGTGGGGAGCAAACAGGATTAGATACCCTGGTAGTCCACGCCGTAAACGATGAGTGCTA<br>GGTGTTGGGTCCTTTCCGGGACTCAGTGCCGCAGCTAACGCATTAAGCACTCCGCCTGGGGAGTACGACCGCAAGGTTG<br>AAACTCAAAGGAATTGACGGGGGCCCGCACAAGCGGTGGAGCATGTGGTTTAATTCGAAGCAACGCGAAGAACCTTACCAG<br>GTCTTGACATCCCTCTGACCGCTCTAGAGATAGAGTTTTCCTTCGGGACAGAGGTGACAGGTGGTGCATGGTTGTCGTCA<br>GCTCGTGTCGTGAGATGTTGGGTTAAGTCCCGCAACGAGCGCAACCCCTATTGTTAGTTGCCATCATTCAGTTGGGCACT<br>CTAGCGAGACTGCCGGTAATAAACCGGAGGAAGGTGGGGATGACGTCAAATCATCATGCCCCTTATGACCTGGGCTACAC<br>ACGTGCTACAATGGCTGGTACAACGAGTCGCAAGTCGGTGACGGCAAGCTAATCTCTTAAAGCCAGTCTCAGTTCGGATTG<br>TAGGCTGCAACTCGCCTACATGAAGTCGGAATCGCTAGTAATCGCGGATCAGCACGCCGCGGTGAATACGTTCCCGGGC<br>CTTGTACACACCGCCCGTCACACCACGAGAGTTTGTAACACCCGAAGTCGGTGAGGTAACCTTTTAGGAGCCAGCCGCCT<br>AAGGTGGGATAGATGATTGGGGTG
30	Prevotella sp. OTU_39	GATGAACGCTAGCTACAGGCTTAACACATGCAAGTCGCGGGGCAGCATGGGGGTTGCTTGCAACCCCTGATGGCGACCG<br>GCGCACGGGTGAGTAACGCGTATCCAACCTGCCCTTCACCACGGGATAACCCGGCGAAAGTCGGACTAATACCGTATGTT<br>GTCCATTGACGGCATCCGATTTGGACGAAAGGCTTAGCGGTGAGGGATGGGGATGCGTCCGATTAGCTTGACGGCGGGG<br>TAACGGCCCACCGTGGCAACGATCGGTAGGGGTTCTGAGAGGAAGGTCCCCCACACTGGAACTGAGACACGGTCCAGAC<br>TCCTACGGGAGGCAGCAGTGAGGAATATTGGTCAATGGGCGGAAGCCTGAACCAGCCAAGTAGCGTGCAGGATGACGGC<br>CCTATGGGTTGTAAACTGCTTTTATGCGGGGATAAAGTGGCCCACGTGTGGGTTTTTGCAGGTACCGCATGAATAAGGACC<br>GGCTAATTCCGTGCCAGCAGCCGCGGTAATACGGAAGGTCCGGGCGTTATCCGGATTTATTGGGTTTAAAGGGAGCGTAG<br>GCCGCCCCTTAAGCGTGTTGTGAAATGCGGGTGCTCAACATCCGACTTGCAGCGCGAACTGGGGGGCTTGAGTGCGCCG<br>AAAGTAGGCGGAATTCGTGGTGTAGCGGTGAAATGCTTAGATATCACGAAGAACTCCGATTGCGAAGGCAGCTTACTGTAG<br>CGCAACTGACGCTGATGCTCGAAAGCGTGGGTATCGAACAGGATTAGATACCCTGGTAGTCCACGCGGTAAACGATGGAT<br>GCTCGTTGTCGGCCTTTGTGGTCGGTGACCAAGCGAAAGCGTTAAGCATCCCACCTGGGGAGTACGCCGGCAACGGTGA<br>AACTCAAAGGAATTGACGGGGGCCCGCACAAGCGGAGGAACATGTGGTTTAATTCGATGATACGCGAGGAACCTTACCCG<br>GGCTTGAACTGCGGCGGAACGATTCCGAGAGGATGAGGCCCTTCGGGGCTGCCGCGGAGGTGCTGCATGGTTGTCGTC<br>AGCTCGTGCCGTGAGGTGTCGGCTTAAGTGCCATAACGAGCGCAACCCCCGTCTTCAGTTGCCATCGGATGATGCCGGG<br>CACTCTGGAGATACTGCCACCGCAAGGTGTGAGGAAGGTGGGGATGACGTCAAATCAGCACGGCCCTTACGTCCGGGGC<br>TACACACGTGTTACAATGGCGCATACAGAGAGTAGGTGGCATGCGAATGCCATCCAATCCTTAAAGTGCGTCTCAGTTCGG<br>ACTGGGGTCTGCAACCCGACCCCACGAAGCTGGATTCGCTAGTAATCGCGCATCAGCCATGGCGCGGTGAATACGTTCC<br>CGGGCCTTGTACACACCGCCCGTCAAGCCATGAAAGCCGGGGGCGCCTGAAGTCCGTGACCGCGAGGGTCGGCCTAGG<br>GTGAAACCGGTGATTGGGGCT
31	Phocaeicola sp. OTU_40	GATGAACGCTAGCTACAGGCTTAACACATGCAAGTCGTGGGGCATCATGATGTAGTAATACATTGATGGCGACCGGCGCAC<br>GGGTGAGTAACGCGTATCCAACCTACCCTCCACTCAAGTATAGGCTTCCGAAAGGGAGATTAATCCTTGATATCGTCATTCT<br>CTCTCCTGTTAGAATGATAAAAGGACATCGTTTCGGTAGAGGATGGGGATGCGTTCCATTAGGTAGTAGGTGGTTTAACAGG<br>CCACCTAGCCGATGATGGATAGGGGTTCTGAGAGGAATATCCCCCACACTGGTACTGAGATACGGACCAGACTCCTACGG<br>GAGGCAGCAGTGAGGAATATTGGTCAATGGGCGCAAGCCTGAACCAGCCATGTAGCGTGAAGGATGACGGTTTTTTTGATT<br>GTAAACTTCTTTTCTGTGCGATTAAAGTGTGGCATTTATGCCATATTGCAGTTATCTCAGGAATAAGCATCGGCTAACTCCGT<br>GCCAGCAGCCGCGGTAATACGGAGGATGCGAGCGTTATCCGGATTTATTGGGTTTAAAGGGAGCGTAGATGGTTTTTTAAG<br>TCAGCTGTCAAATGTATGGGCTCAACCCTTATTATCAGTTGAAACTGTAAGACTTGAGTGCGGTGTGGGTACATGGAATTCG<br>TGGTGTAGCGGTGAAATGCATAGATATCACGAAGAACTCCGAAAGCGTAGGCATTGTACCATAGCGCCACTGACATTGATG<br>CTCGAAGGTGCGGGAATCAAACAGGATTAGATACCCTGGTAGTCCGCACAGTAAACGATGAATGCTCGACGTTGTCGATAT<br>ACTGTCAGCGTCCAAGCGAAAGCGTTAAGCATTCCACCTGGGGAGTACGCCGGCAACGGTGAAACTCAAAGGAATTGACG<br>GGGGCCCGCACAAGCGGAGGAACATGTGGTTTAATTCGATGATACGCGCGGAACCTTACCCGGGCTTGAATTGTACCTGT<br>AGGTGTTTGAAAGATCATCGTCTTGTAAAGAGCAGGTATGAAGGTGCTGCATGGTTGTCGTCAGCTCGTGCCGTGAGGTGT<br>CGGCTCAAGTGCCATAACGAGCGCAACCCTTTCCATCAGTTACAAACGAAATTAGTCGTGTACTCTGTTGGTACTGCCATC<br>GTAAGATGTGAGGAAGGTGGGGATGACGTCAAATCAGCACGGCCCTTACGTTCGGGGCTACACACGTGTTACAATGGGGG<br>GTACAGTGGGTCGCTATCCGGCGACGGAGTGCCAATCCTCAAAACCCCTCCCAGTTCGGACTGGAGTCTGCAACCCGAC<br>TCCACGAAGCTGGATTCGCTAGTAATCGCGCATCAGCCATGGCGCGGTGAATACGTTCCCGGGCCTTGTACACACCGCC<br>CGTCAAGCCATGAAAGCCGGGGGTACCTGAAGCACGTTATCGCAAGTATCGTTCCAGGGTAAATCTGGTGATTGGGGCT
32	Campylobacter sp. OTU_44	AGTGAACGCTGGCGGCGTGCCTAATACATGCAAGTCGAACGGACAAGTAAGAGCTTGCTCTTATGAGTTAGTGGCGCACG<br>GGTGAGTAATGTATAGCTAATCTGCCCCATAGTGGAGGACAACAGTTGGAAACGACTGCTAATACTCCATACTCCATTTGTA<br>TATAAGTATAAATGGGAAAGTAGCCCTATGTAATGTTTATTTAAGCAATTAGATGAATTTTTAGACTAAACCTAAATTTTATGATT<br>TATGACAAGCGAAGCTTGGAGTAAATCTTTATAGCTTTAGGTGGGTGCAGGGAGTGAAACTCCCGCTCGTAAAGACTAGCTA<br>AGCTAGTCTGCAAAGCAAGTAAATATTACATAGGGTTTCTCGCTATGGGATGAGGCTATATCGTATCAGCTAGTTGGTAGGG<br>TAATGGCTTACCAAGGCTATGACGCGTAACTGGTCTGAGAGGATGATCAGTCACACTGGAACTGAGACACGGTCCAGACTC<br>CTACGGGAGGCAGCAGTAGGGAATATTGCTCAATGGGGGAAACCCTGAAGCAGCAACGCCGCGTGGAGGATGACACTTTT<br>CGGAGCGTAAACTCCTTTTCTTGGGGAAGAAATTTGACGGTACCCAAGGAATAAGCACCGGCTAACTCCGTGCCAGCAGC<br>CGCGGTAATACGGAGGGTGCAAGCGTTACTCGGAATCACTGGGCGTAAAGGACGCGTAGGCGGATGAATAAGTCTCTTGT<br>GAAATCTAACGGCTTAACCGTTAAACTGCTTGGGAAACTATTTATCTAGAGTGAGGGAGAGGCAGATGGAATTGGTGGTGTA<br>GGGGTAAAATCCGTAGAGATCACCAGGAATACCCATTGCGAAGGCGATCTGCTGGAACTCAACTGACGCTAATGCGTGAAA<br>GCGTGGGGAGCAAACAGGATTAGATACCCTGGTAGTCCACGCCCTAAACGATGTATACTAGTTGTTGCTGAGCTAGTCTTG<br>GCAGTAATGCACCTAACGGATTAAGTATACCGCCTGGGGAGTACGGTCGCAAGATTAAAACTCAAAGGAATAGACGGGGAC<br>CCGCACAAGCGGTGGAGCATGTGGTTTAATTCGAAGATACGCGAAGAACCTTACCCGGACTTGATATCTAACAAATCGTCTA<br>GAGATAGAAGAGTGTCTGCTTGCAGAAATGTTAAGACAGGTGCTGCACGGCTGTCGTCAGCTCGTGTCGTGAGATGTTGGG<br>TTAAGTCCCGCAACGAGCGCAACCCACGTCATTAGTTGCTAACGGTTCGGCCGAGCACTCTAATGAGACTGCCTTCGCAA<br>GGAGGAGGAAGGTGTGGACGACGTCAAGTCATCATGGCCCTTATGTCCGGGGCGACACACGTGCTACAATGGCATATACA<br>ATGAGACGCAATATCGCGAGATGGAGCAAATCTATAAAATATGTCCCAGTTCGGATTGGAGTCTGCAACTCGACTCCATGAA<br>GCCGGAATCGCTAGTAATCGTAGATCAGCCATGCTACGGTGAATACGTTCCCGGGTCTTGTACTCACCGCCCGTCACACC<br>ATGGGAGTTGATTTCACTCGAAGCCCAAATACCAAACCGGTTATGGTCCACAGTGGAATCAGCGACTGGGGTG

Sr no.	Species_OTUs	16S reference sequence
33	Prevotella sp. OTU_45	GATGAACGCTAGCTACAGGCTTAACACATGCAAGTCGAGGGGAAACGACGTTGAAGCTTGCTTCGATGGTCGTCGACCGG<br>CGCACGGGTGAGTAACGCGTATCCAACCTGCCTCTGACTAGGGGATAACCCGGCGAAAGTCGGACTAATACCTTATGGCA<br>TCGTCTGCGGGCATCCAACGACGATTAAAGATTCATCGGTCAGAGATGGGGATGCGTCTGATTAGCTTGTTGGCGGGGTAA<br>CGGCCCACCAAGGCGACGATCAGTAGGGGTTCTGAGAGGAAGGTCCCCCACATTGGAACTGAGACACGGTCCAAACTCC<br>TACGGGAGGCAGCAGTGAGGAATATTGGTCAATGGGCGGAAGCCTGAACCAGCCAAGTAGCGTGCAGGATGACGGCCCTA<br>TGGGTTGTAAACTGCTTTTATGCGGGGATAAAGTGAGGGACGTGTCCTTCATTGCAGGTACCGCATGAATAAGGACCGGCT<br>AATTCCGTGCCAGCAGCCGCGGTAATACGGAAGGTCCAGGCGTTATCCGGATTTATTGGGTTTAAAGGGAGCGTAGGCCG<br>TGGATTAAGCGTGTTGTGAAATGTAGACGCTCAACGTCTGACTTGCAGCGCGAACTGGTTCACTTGAGTATGCGCAACGTA<br>GGCGGAATTCGTCGTGTAGCGGTGAAATGCTTAGATATGACGAAGAACTCCGATTGCGAAGGCAGCTTACGGGAGCATAAC<br>TGACGCTGAAGCTCGAAAGTGCGGGTATCGAACAGGATTAGATACCCTGGTAGTCCGCACGGTAAACGATGGATGCCCGT<br>TGTCAGGCTGTTTCAGCCTGGTGACCAAGCGAAAGCATTAAGCATCCCACCTGGGGAGTACGCCGGCAACGGTGAAACTC<br>AAAGGAATTGACGGGGGCCCGCACAAGCGGAGGAACATGTGGTTTAATTCGATGATACGCGAGGAACCTTACCCGGGCTT<br>GAATTGCAGACGAACGATTCAGAGATGATGAGGCCTTTCGGGGCGTCTGTGAAGGTGCTGCATGGTTGTCGTCAGCTCGT<br>GCCGTGAGGTGTCGGCTTAAGTGCCATAACGAGCGCAACCCCTGTCTTCAGTTGCCATCAGGTGATGCTGGGCACTCTG<br>GAGATACTGCCACCGTAAGGTGTGAGGAAGGTGGGGATGACGTCAAATCAGCACGGCCCTTACGTCCGGGGCTACACAC<br>GTGTTACAATGGCCGGTACAGAGCGTCGGTCGTATGCAAATGCGATCCAATCCTTAAAGCCGGTCCCAGTTCGGACTGGG<br>GTCTGCAACCCGACCCCACGAAGCTGGATTCGCTAGTAATCGCGCATCAGCCATGGCGCGGTGAATACGTTCCCGGGCC<br>TTGTACACACCGCCCGTCAAGCCATGAAAGCCGGGGGTGCCTGAAGTCTGTGACCGCAAGGAACGGCCTAGGGCAAAAC<br>TGGTGATTGGGGCT
34	Porphyromonas sp. OTU_46	GATGAACGCTAGCGATAGGCTTAACACATGCAAGTCGAGGGGCAGCATGGTCTTAGCTTGCTAAGACTGATGGCGACCGG<br>CGCACGGGTGCGTAACGCGTATGCAACTTGCCTCACAGAGGGGGATAACCCGTCGAAAGACGGACTAATACCGCGTACAC<br>TCCAGTCTGGGCATCCAGGCTAGAGGAAATGAAATTCGCTGTGAGATAGGCATGCGTCCCATTAGCTAGTTGGTGAGGTAA<br>CGGCTCACCAAGGCAACGATGGGTAGGGGAACTGAGAGGTTGAACCCCCACACTGGTACTGAGACACGGACCAGACTCC<br>TACGGGAGGCAGCAGTGAGGAATATTGGTCAATGGGCGAGAGCCTGAACCAGCCAAGTCGCGTGAAGGATGACTGTCTTA<br>TGGATTGTAAACTTCTTTTGTAGGGGAATAAAGAACAGTACGTGTACTGTAGTGAATGTACCCTACGAATAAGCATCGGCTAA<br>CTCCGTGCCAGCAGCCGCGGTAATACGGAGGATGCGAGCGTTATCCGGATTTATTGGGTTTAAAGGGTGCGTAGGCGGC<br>CTGTTAAGTCAGCGGTGAAATCTAGGGGCTTAACTCCTAAATTGCCATTGATACTGGTGGGCTTGAGTGTAGATGAGGTAGG<br>CGGAATGCGTGGTGTAGCGGTGGAATGCATAGATATCACGCAGAACTCCAATTGCGAAGGCAGCTTACTAAGGTACAACTG<br>ACGCTGAAGCACGAAAGCGTGGGTATCAAACAGGATTAGATACCCTGGTAGTCCACGCAGTAAACGATGATAACTGGGCGT<br>ATGCGATATACAGTATGCTCCTAAGCGAAAGCGTTAAGTTATCCACCTGGGGAGTACGCCGGCAACGGTGAAACTCAAAGG<br>AATTGACGGGGGCCCGCACAAGCGGAGGAACATGTGGTTTAATTCGATGATACGCGAGGAACCTTACCCGGGATTGAAATG<br>TAGATGACGGCCGGTGAAAGTCGGCTTCCCTTCGGGGCATCTATGTAGGTGCTGCATGGTTGTCGTCAGCTCGTGCCGT<br>GAGGTGTCGGCTTAAGTGCCATAACGAGCGCAACCCCTGTCTACGGTTGCCATCAGGTAATGCTGGGAACTCCGTAGAGA<br>CTGCCGTCGTAAGGCGTGAGGAAGGTGGGGATGACGTCAAATCAGCACGGCCCTTACATCCGGGGCGACACACGTGTTA<br>CAATGGTGGGGACAAAGGGCAGCTACCTGGCGACAGGATGCGAATCTCCAAACCCCATCACAGTTCGGATCGGAGTCTGC<br>AACTCGACTCCGTGAAGCTGGATTCGCTAGTAATCGCGCATCAGCCATGGCGCGGTGAATACGTTCCCGGGCCTTGTACA<br>CACCGCCCGTCAAGCCATGGGAGTCTGGAGTACCTAAAGTCCGTACCTGCGAGGGTCGGCCTAGGGTAATACAGGTGAC<br>TGGGGCT
35	Treponema sp. OTU_47	AACGAACGCTGGCGGCGCGTCTTAAGCATGCAAGTCGAACGGCAAGAGAGGAGCTTGCTTCTCTCCTAGAGTGGCGGACT<br>GGTGAGGAACACGTGGGTAATCTACCCTTAAGATGGGGATAGCTGCTAGAAATAGCAGATAATACCGAATACACTCGGCGC<br>TTCATAAGAGGCGTTGAGGAAAGGAAGCTACGGCTTCGCTTGAGGATGAGCTTGCGTCCCATTAGCTAGTTGGTGAGGTAA<br>CGGCCCACCAAGGCGACGATGGGTATCCGGCCTGAGAGGGTGAACGGACACATTGGGACTGAGATACGGCCCAAACTCC<br>TACGGGAGGCAGCAGCTAAGAATATTCCGCAATGGACGGAAGTCTGACGGAGCGACGCCGCGTGGATGAAGAAGGCTGAA<br>AAGTTGTAAAATCCTTTTGTTGATGAAGAATAAGGATGAGAGGGAATGCTCATCTGATGACGGTAATCGACGAATAAGCCCCG<br>GCTAATTACGTGCCAGCAGCCGCGGTAACACGTAAGGGGCGAGCGTTGTTCGGAATTATTGGGCGTAAAGGGCATGTAGG<br>CGGTTATGTAAGCCTGATGTGAAATCCTGGGGCTTAACCCCAGAATAGCATTGGGTACTGTGTAACTTGAATTACGGAAGGG<br>AAACTGGAATTCCAAGTGTAGGGGTGGAATCTGTAGATATTTGGAAGAACACCGGTGGCGAAGGCGGGTTTCTGGCCGATA<br>ATTGACGCTGAGATGCGAAAGTGTGGGGATCGAACAGGATTAGATACCCTGGTAGTCCACACCGTAAACGATGTACACTAG<br>GTGTTGGGGCAAGAGCTTCAGTGCCAAAGCAAACGCGATAAGTGTACCGCCTGGGGAGTATGCCCGCAAGGGTGAAACTC<br>AAAGGAATTGACGGGGGCCCGCACAAGCGGTGGAGCATGTGGTTTAATTCGATGGTACGCGAGGAACCTTACCTGGGTTT<br>GACATCTAGTAGAAGGTCTTAGAGATAAGGCTGGGTAGCAATACCCTGCTAGACAGGTGCTGCATGGCTGTCGTCAGCTCG<br>TGCCGTGAGGTGTTGGGTTAAGTCCCGCAACGAGCGCAACCCCTACTGCCAGTTACTAACAGGTAAAGCTTGAGGACTCT<br>GGCGGAACTGCCGATGACAAATCGGAGGAAGGTGGGGATGACGTCAAGTCATCATGGCCCTTATGTCCAGGGCTACACAC<br>GTGCTACAATGGTTGCTACAAAGCGAAGCGAGACCGTAAGGTGGAGCAAACCGCAAAAAAGCAATCGTAGTTCGGATTGAA<br>GTCTGAAACTCGACTTCATGAAGTTGGAATCGCTAGTAATCGCGCATCAGCACGGCGCGGTGAATACGTTCCCGGGCCTT<br>GTACACACCGCCCGTCACACCATCCGAGTTGGGGGTACCCGAAGTCGCTTGTCTAACCTGCAAAGGAGGACGGTGCCGA<br>AGGTACGCTTGGTAAGGAGGGTG
36	Mogibacterium sp. OTU_48	GATGAACGCTGGCGGCGTGCCTAATACATGCAAGTCGAGCGAGATGTTAGCGCATGAACCTTCGGGGGATTATGCTAACG<br>GACAGCGGCGGACGGGTGAGTAACGCGTAGGCAACCTGCCCCTGACAGAGGGATAGCCATTGGAAACGATGATTAAAACC<br>TCATGACACCGTAGAAGCACATGCTTCATCGGTCAAAGATTTATCGGTCAGGGATGGGCCTGCGTCTGATTAACTAGTTGG<br>TGAAGTAACGGCTCACCAAGGTGACGATCAGTAGCCGACCTGAGAGGGTGATCGGCCACATTGGAACTGAGACACGGTCC<br>AAACTTCTACGGAAGGCAGCAGTAGGGAATCTTGCACAATGGGCGAAAGCCTGATGCAGCAACGCCGCGTGAAGGATGAA<br>GGCCTTCGGGTTGTAAACTTCTGTTCTAAGGGAAGAAACAAATGACGGTACCTTAGGAGCAAGCCCCGGCTAACTACGTGC<br>CAGCAGCCGCGGTAATACGTAGGGGGCAAGCGTTATCCGGAATTATTGGGCGTAAAGAGTGCGTAGGTGGTTACCTAAGC<br>GCAAGGTTTAATTTAGAGGCTCAACCTCTACTTGCCTTGCGAACTGGGCTACTTGAGTGCAGGAGGGGAAAGCGGAATTCC<br>TAGTGTAGCGGTGAAATGCGTAGATATTAGGAGGAACACCAGCGGCGAAGGCGGCTTTCTGGACTGTAACTGACACTGAGG<br>CACGAAAGCGTGGGTAGCAAACAGGATTAGATACCCTGGTAGTCCACGCCGTAAACGATGAGCACTAGGTGTTGGGCCCG<br>TTAGGGCTCAGTGCCGCAGTTAACGCAATAAGTGCTCCGCCTGGGGAGTACGCTCGCAAGAGTAAAACTCAAAGGAATTGA<br>CGGGGACCCGCACAAGCAGCGGAGCATGTGGTTTAATTCGAAGCAACGCGAAGAACCTTACCAGGGCTTGACATCCTGCT<br>GACAGGACCTTAATCGGTCCCTTCTTCGGACAGCAGAGACAGGTGGTGCATGGTTGTCGTCAGCTCGTGTCGTGAGATGT<br>TGGGTTAAGTCCCGCAACGAGCGCAACCCTTGTCGCTAGTTACTAACATTCAGTTGAGGACTCTAGCGAGACTGCCGAGG<br>TCAACTCGGAGGAAGGTGGGGATGACGTCAAATCATCATGCCCCTTATGTTCTGGGCTACACACGTGCTACAATGGTCGGT<br>ACAATGAGAGGCAATACTGTGAAGTGGAGCAAATCACCAAAACCGATCCCAGTTCGGATTGTAGGCTGCAACTCGCCTACA<br>TGAAGTTGGAGTTGCTAGTAATCGCAGATCAGAATGCTGCGGTGAATGCGTTCCCGGGTCTTGTACACACCGCCCGTCAC<br>ACCATGGAAGTTGGGGGTGCCCAAAGTCGGTTAATTAATCTATCGCCTAAGGCAAAACCAATGACTGGGGTG

Sr no.	Species_OTUs	16S reference sequence
37	Lactobacillus sp. OTU_50	GACGAACGCTGGCGGCGTGCCTAATACATGCAAGTCGAGCGAGCTGAACCAACAGATTTACTTCGGTAATGACGTTGGGAA<br>CGCGAGCGGCGGATGGGTGAGTAACACGTGGGGAACCTGCCCCTCAGTCTGGGATACCACTTGGAAACAGGTGCTAATA<br>CCGGATAATAAAGCAGATCGCATGATCAGCTTTTGAAAGGCGGCGTAAGCTGTCGCTAAGGGATGGCCCCGCGGTGCATT<br>AGCTAGTTGGTAGGGTAACGGCCTACCAAGGCAATGATGCATAGCCGAGTTGAGAGACTGATCGGCCACATTGGGACTGA<br>GACACGGCCCAAACTCCTACGGGAGGCAGCAGTAGGGAATCTTCCACAATGGACGAAAGTCTGATGGAGCAACGCCGCGT<br>GAGTGAAGAAGGTTTTCGGATCGTAAAGCTCTGTTGTTGGTGAAGAAGGATAGAGGTAGTAACTGGCCTTTATTTGACGGTA<br>ATCAACCAGAAAGTCACGGCTAACTACGTGCCAGCAGCCGCGGTAATACGTAGGTGGCAAGCGTTGTCCGGATTTATTGG<br>GCGTAAAGCGAGCGCAGGCGGAAGAATAAGTCTGATGTGAAAGCCCTCGGCTTAACCGAGGAACTGCATCGGAAACTGTTT<br>TTCTTGAGTGCAGAAGAGGAGAGTGGAACTCCATGTGTAGCGGTGGAATGCGTAGATATATGGAAGAACACCAGTGGCGAA<br>GGCGGCTCTCTGGTCTGCAACTGACGCTGAGGCTCGAAAGCATGGGTAGCGAACAGGATTAGATACCCTGGTAGTCCATG<br>CCGTAAACGATGAGTGCTAAGTGTTGGGAGGTTTCCGCCTCTCAGTGCTGCAGCTAACGCATTAAGCACTCCGCCTGGGG<br>AGTACGACCGCAAGGTTGAAACTCAAAGGAATTGACGGGGGCCCGCACAAGCGGTGGAGCATGTGGTTTAATTCGAAGCAA<br>CGCGAAGAACCTTACCAGGTCTTGACATCTAGTGCCATCCTAAGAGATTAGGAGTTCCCTTCGGGGACGCTAAGACAGGTG<br>GTGCATGGCTGTCGTCAGCTCGTGTCGTGAGATGTTGGGTTAAGTCCCGCAACGAGCGCAACCCTTGTTATTAGTTGCCA<br>GCATTAAGTTGGGCACTCTAATGAGACTGCCGGTGACAAACCGGAGGAAGGTGGGGATGACGTCAAGTCATCATGCCCCT<br>TATGACCTGGGCTACACACGTGCTACAATGGGCAGTACAACGAGAAGCAAGCCTGCGAAGGCAAGCGAATCTCTTAAAGCT<br>GTTCTCAGTTCGGACTGCAGTCTGCAACTCGACTGCACGAAGCTGGAATCGCTAGTAATCGCGGATCAGCACGCCGCGGT<br>GAATACGTTCCCGGGCCTTGTACACACCGCCCGTCACACCATGGGAGTCTGCAATGCCCAAAGCCGGTGGCCTAACCTT<br>CGGGAAGGAGCCGTCTAAGGCAGGGCAGATGACTGGGGTG
38	Capnocytophaga sp. OTU_52	GATGAACGCTAGCGGCAGGCCTAACACATGCAAGTCGAGGGGGAGGTTACTTTCGGGTGACTGAGACCGGCGTACGGGT<br>GCGTAACGCGTATACAATCTGCCTTTCACTGGGGGATAGCCCGAAGAAATTTGGATTAATACCCCATAGTATAGTAGTGTGG<br>CATCACACAACTATTAAAGCTTAGGTGGTGAAAGATGAGTATGCGTTCTATTAGCTAGTTGGAGAGGTAACGGCTCCCCAAG<br>GCGATGATAGATAGGGGTTCTGAGAGGGATGTCCCCCACACTGGTACTGAGATACGGACCAGACTCCTACGGGAGGCAGC<br>AGTGAGGAATATTGGACAATGGTCGGAAGACTGATCCAGCCATGCCGCGTGCAGGATGACGGCCTTATGGGTTGTAAACTG<br>CTTTTGTAAGGGAAGAATAAGGACTACGTGTAGTTTGATGACGGTACCTTATGAATAAGCATCGGCTAACTCCGTGCCAGCA<br>GCCGCGGTAATACGGAGGATGCGAGCGTTATCCGGAATCATTGGGTTTAAAGGGTCTGTAGGCGGGCTGGTAAGTCAGAG<br>GTGAAAGCGCTTAGCTCAACTAAGCAACTGCCTTTGAAACTGTTGGTCTTGAATGGTTGTGAAGTAGTTGGAATGTGTAGTG<br>TAGCGGTGAAATGCTTAGATATTACACAGAACACCGATAGCGAAGGCATATTACTAACAATTAATTGACGCTGATGGACGAAA<br>GCGTGGGGAGCGAACAGGATTAGATACCCTGGTAGTCCACGCTGTAAACGATGGATACTAGCTGTTTGGAGCAATCTGAGT<br>GGCTAAGCGAAAGTGATAAGTATCCCACCTGGGGAGTACGTTCGCAAGAATGAAACTCAAAGGAATTGACGGGGGCCCGCA<br>CAAGCGGTGGAGCATGTGGTTTAATTCGATGATACGCGAGGAACCTTACCAAGGTTTAAATGGGAACTGACAGAGGTAGAGA<br>TACCTTCTTCTTCGGACAGTTTTCAAGGTGCTGCATGGTTGTCGTCAGCTCGTGCCGTGAGGTGTCAGGTTAAGTCCTATA<br>ACGAGCGCAACCCCTGCCATTAGTTGCTAACGAGTCGAGTCGAGCCCTCTAATGGGACTGCCGGTGCAAACCGAGAGGAA<br>GGTGGGGATGACGTCAAATCATCACGGCCCTTACATCTTGGGCTACACACGTGCTACAATGGCCGTTACAGAGAGCAGCC<br>ACTGCGTGAGCAGGCGCGAATCTATAAAGACGGTCACAGTTCGGATCGGAGTCTGCAACTCGACTCCGTGAAGCTGGAAT<br>CGCTAGTAATCGGATATCAGCCATGATCCGGTGAATACGTTCCCGGGCCTTGTACACACCGCCCGTCAAGCCATGGAAGC<br>TGGGGGTACCTGAAGACGGTCACCGCGAGGAACTGTTTAGGGTAAAACTAGTGACTGGGGCT
39	Cryptobacterium sp. OTU_53	GATGAACGCTGGCGGCGCGCCTAACACATGCAAGTCGAACGATTAAACCGCCCTTGGGCGGATATACAGTGGCGAACGG<br>GTGAGTAACGCGTGACCAACCTTCCCCCCGCATCGGGACAACCCGGGGAAACTCGGGCTAATACCGGATATGCCGGGTA<br>TGGCGCATGTCATACCCAGGAAAGCTTTTGCGGCGGGGGATGGGGTCGCGTCCCATCAGGTAGACGGCGGGGTAGTAGC<br>CCACCGTGCCAACGACGGGTAGCCGGGTTGAGAGACCGACCGGCCACATTGGGACTGAGATACGGCCCAGACTCCTAC<br>GGGAGGCAGCAGTGGGGAATCTTGCGCAATGGGGGAAACCCTGACGCAGCGACGCCGCGTGCGGGATGGAGGCCTTCG<br>GGCCGTGAACCGCTTTCAGCAGGGAAGACATTAAGGACGGTACCTGCAGAAGAAGCCCCGGCTAACTACGTGCCAGCAG<br>CCGCGGTAATACGTAGGGGGCGAGCGTTATCCGGATTCATTGGGCGTAAAGCGCGCGCAGGCGGCGCACCAGGCGGGA<br>TCTCGAAACCGGGGGCTCAACCTCCGGGCGGATCCCGAACCGGTGTGCTCGAGTGCGGTAGAGGAAGTCGGAATTCCC<br>GGTGTAGCGGTGAAATGCGCAGATATCGGGAAGAACACCGATGGCGAAGGCAGACTTCTGGGCCGACACTGACGCTCAG<br>GCGCGAAAGCTGGGGGAGCGAACAGGATTAGATACCCTGGTAGTCCCAGCCGTAAACGATGGGCACTAGGTGTGGGGGG<br>CATTTGCCTTCCGTGCCGCAGCTAACGCATTAAGTGCCCCGCCTGGGGAGTACGGCCGCAAGGCTAAAACTCAAAGGAAT<br>TGACGGGGGCCCGCACAAGCAGCGGAGCATGTGGCTTAATTCGAAGCAACGCGAAGAACCTTACCAGGGCTTGACATGTA<br>GGTGAAGCGACAGAGATGTCGCAGCCGAAAGGAGCCTACACAGGTGGTGCATGGCTGTCGTCAGCTCGTGTCGTGAGAT<br>GTTGGGTTAAGTCCCGCAACGAGCGCAACCCCTGCCGCATGTTGCCAGCATTTAGTTGGGGACTCATGCGGGACCGCCG<br>GCGGCAAGCCGGAGGAAGGCGGGGACGACGTCAAGTCATCATGCCCCTTATGCCCTGGGCTGCACACGTGCTACAATGG<br>CCGGCACAACGGGATGCAAAGGCGCGAGCCCAAGCGGATCCCCCAAAGCCGGTCCCAGTTCGGATCGGAGGCTGCAAC<br>CCGCCTCCGTGAAGCCGGAGTTGCTAGTAATCGCGGATCAGCATGCCGCGGTGAATACGTTCCCGGGCCTTGTACACAC<br>CGCCCGTCACACCACCCGAGTCGTCTGCACCCGAAGTCGCCGGCCGAACCCTTTTGGGACGGAGGCGCCGAAGGCGC<br>GGGGGATAAGGGGGGTG
40	Peptococcus sp. OTU_54	GACAAACGCTGGCGGCATGCCTAACACATGCAAGTCGAACGGACAGAACAGGGAAGCTTTTCGGGGTGGAACTGGAAAGT<br>TAGTGGCGGATGGGTGAGTAACGCGTGGACAACCTACCTTGTAAAGGGGAATAACTTCGAGAAATCGAAGCTAATACCCCA<br>TAATAACCTTGAATCGCATGAAGCAAGGAGGAAAGGCGGCCTTAGAGCTGTCGATAGAAGATGGGTCCGCGTCTGATTAGC<br>TAGTTGGTGAGGTAAAGGCTCACCAAGGCGACGATCAGTAGCCGGCCTGAGAGGGTGAACGGCCACATTGGGACTGAGAC<br>ACGGCCCAAACTCCTACGGGAGGCAGCAGTGGGGAATCTTCCGCAATGGGCGAAAGCCTGACGGAGCAATGCCGCGTGA<br>GTGAAGAAGGCCTTCGGGTTGTAAAACTCTGTCCTAGACGAAGAGAGAGTATTGTAGTAACTGACAATACTAGGACGGTAGA<br>CTAGGAGGAAGCCCCGGCTAACTATGTGCCAGCAGCCGCGGTAAAACATAGGGGGCAAGCGTTGTCCGGAATCACTGGG<br>CGTAAAGGGCGCGCAGGCGGTAAATTAAGTCAGGTGTGAAAGTTCGGGGCTCAACCCCGTGATTGCACCTGATACTGATAA<br>ACTAGAGTGTTGGAGAGGTAAGTGGAATTCCTAGTGTAGCGGTGGAATGCGTAGATATTAGGAGGAACACCAGTGGCGAAG<br>GCGACTTACTGGCCAATAACTGACGCTGAGGCGCGAAAGCGTGGGGAGCAAACAGGATTAGATACCCTGGTAGTCCACGC<br>CGTAAACGATGAGTACTAGGTGTTGGTTGTTTCGAAACAATCGGTGCCGCAGTAAACACAATAAGTACTCCGCCTGGGGAG<br>TACGGTCGCAAGACTGAAACTCAAAGGAATTGACGGGGGCCCGCACAAGCGGTGGAGCATGTGGTTTAATTCGACGCAAC<br>GCGAAGAACCTTACCAAGACTTGACATCCTCTTGACGGGATATGTAATGTATCCGTTTCTTCGGAACAAGAGAGACAGGTGG<br>TGCATGGTTGTCGTCAGCTCGTGTCGTGAGATGTTGGGTTAAGTCCCGCAACGAGCGCAACCCTTGTTGTTAGTTGCCAT<br>CATTGAGTTGGGCACTCTAACAAGACTGCCGGTGACAAACCGGAGGAAGGTGGGGATGACGTCAAATCATCATGCCCCTTA<br>TGTCTTGGGCTACACACGTGCTACAATGGTCGGTACAGAGGGCAGCAAGAGAGTGATCTTGAGCGAATCTCAAAAAGCCGA<br>TCCCAGTTCGGATTGTAGGCTGCAACTCGCCTACATGAAGTCGGAATCGCTAGTAATCGCAGGTCAGCATACTGCGGTGAA<br>TACGTTCCCGGGCCTTGTACACACCGCCCGTCACACCACGAAAGTTGGTAACACCCGAAGTCGGTGATCTAACCTGCAAA<br>GGAGGAAGCCGCCCACGGTGGGATCGATGATTGGGGTG

Sr no.	Species_OTUs	16S reference sequence
41	Selenomonas sp. OTU_58	GACGAACGCTGGCGGCGTGCTTAACACATGCAAGTCGAACGGAGTCTAAAAATTACCACTGAGATCTTAGTCAGTGGGCGA<br>GCAGCGTCATGGACACGAAGTGGCCATCACGCGTTAAAAAGTGCCAACACAGGGCTGAGGCCTGAGTGGTAATTTTTAGAC<br>TGAGTGGCAAACGGGTGAGTAACACGTAGGCAACCTGCCGACAGGATGGGGACAACATTCCGAAAGGAATGCTAATACCGA<br>ATGGAGTCCGGGGATGGCATCATCCCCGGATAAAAGATGGCCTCTGAATATGCTATCGCCTGTCGATGGGCCTGCGTCTG<br>ATTAGCCAGTTGGCGGGGTAACGGCCCACCAAAGCGACGATCAGTAGCCGGTCTGAGAGGATGAACGGCCACATTGGGA<br>CTGAGACACGGCCCAGACTCCTACGGGAGGCAGCAGTGGGGAATCTTCCACAATGGGCGAAAGCCTGATGGAGCAACGC<br>CGCGTGAGTGAAGAAGGTCTTCGGATCGTAAAGCTCTGTTGCGCGGGACGAAAACCGAGCTTGAGAATATTGAACCTGGGT<br>GACGGTACCGAGCGAGGAAGCCACGGCTAACTACGTGCCAGCAGCCGCGGTAATACGTAGGTGGCGAGCGTTGTCCGGA<br>ATTATTGGGCGTAAAGGGAGCGCAGGCGGACATATAAGTCCATCTTAAAAGTGCGGGGCTCAACCCCGTGAGGGGATGGA<br>AACTGTATGCCTTGAGTGCAGGAGAGGAAAGCGGAATTCCCAGTGTAGCGGTGAAATGCGTAGATATTGGGAGGAACACCA<br>GTGGCGAAGGCGGCTTTCTGGACTGTAACTGACGCTGAGGCTCGAAAGCCAGGGGAGCGAACGGGATTAGATACCCCGG<br>TAGTCCTGGCCGTAAACGATGAATGCTAGGTGTAGGAGGTATCGACCCCTCCTGTGCCGGAGTTAACGCAATAAGCATTCC<br>GCCTGGGGAGTACGGTCGCAAGACTGAAACTCAAAGGAATTGACGGGGGCCCGCACAAGCGGTGGAGTATGTGGTTTAAT<br>TCGACGCAACGCGAAGAACCTTACCAGGGCTTGACATTGAGTGAAAGAGCTAGAGATAGCTTCCTTCCTTCGGGAACACGA<br>AAACAGGTGGTGCATGGCTGTCGTCAGCTCGTGTCGTGAGATGTTGGGTTAAGTCCCGCAACGAGCGCAACCCCTGTCCT<br>TTGTTGCCAGCGCGTAATGGCGGGAACTCAAAGGAGACTGCCGCGGAGAACGCGGAGGAAGGCGGGGATGACGTCAAGT<br>CATCATGCCCCTTATGTCCTGGGCTACACACGTACTACAATGGGATGGACAGAGGGAAGCGAAGGCGCGAGCCGGAGCG<br>GACCCCACAAACCATCCCCCAGTTCGGATTGCAGGCTGCAACCCGCCTGCATGAAGTCGGAATCGCTAGTAATCGCAGGT<br>CAGCATACTGCGGTGAATACGTTCCCGGGCCTTGTACACACCGCCCGTCACACCACGGAAGTCATTCACGCCCAAAGCC<br>GGCGGGCCAACCGCAAGGAGGCAGCCGTCTAAGGCGGGGGCGATGACTGGGGTG
42	Corynebacterium sp. OTU_60	GACGAACGCTGGCGGCGTGCTTAACACATGCAAGTCGAACGGAAAGGCCCTCACCTTGGTGGGGGTGCTCGAGTGGCGA<br>ACGGGTGAGTAACACGTGGGTGATCTGCCCTGTACTTCGGGATAAGCTTGGGAAACTGGGTCTAATACCGGATAGGACCAT<br>GGTTTAGTGTCTCATGGTGGAAAAATTATTGTTTTGGTGCAGGATGAGCTCGCGGCCTATCAGCTTGTTGGTGGGGTAATG<br>GCCTACCAAGGCGTCGACGGGTAGCCGGCCTGAGAGGGTGGACGGCCACATTGGGACTGAGATACGGCCCAGACTCCT<br>ACGGGAGGCAGCAGTGGGGAATATTGCACAATGGGCGCAAGCCTGATGCAGCGACGCCGCGTGGGGGATGACGGCCTT<br>CGGGTTGTAAACTCCTTTCGTTAGGGACGAAGCCGCACCTTTTTTGGGTGTGGTGACGGTACCTTTGTTAAGAAGCACCGG<br>CTAACTACGTGCCAGCAGCCGCGGTAATACGTAGGGTGCGAGCGTTGTCCGGAATTACTGGGCGTAAAGAGCTCGTAGGT<br>GGTTTGTTGCGTCGTCTGTGAAATTCCGGGGCTTAACTTCGGGGTGGCAGGCGATACGGGCATAACTAGAGTGCTGTAGG<br>GGAGACTGGAATTCCTGGTGTAGCGGTGGAATGCGCAGATATCAGGAGGAACACCGATGGCGAAGGCAGGTCTCTGGGCA<br>GTAACTGACGCTGAGGAGCGAAAGCATGGGGAGCGAACAGGATTAGATACCCTGGTAGTCCATGCTGTAAACGGTGGGCG<br>TTAGGTGTGGGGACCGGTTTTGGTTTCTGTGCCGTAGCTAACGCATTAAGCGCCCCGCCTGGGGAGTACGGCCGCAAGG<br>CTAAAACTCAAAGGAATTGACGGGGGCCCGCACAAGCGGCGGAGCATGTGGATTAATTCGATGCAACGCGAAGAACCTTAC<br>CTGGGTTTGACATGCACCAGACGGTTGTAGAGATACGTACCTCCCTTTGTGGTTGGTGTGCAGGTGGTGCATGGTTGTCG<br>TCAGCTCGTGTCGTGAGATGTTGGGTTAAGTCCCGCAACGAGCGCAACCCTTGTCTTATGTTGCCAGCACTTTGGGTGGG<br>GACTCATGAGAGACTGCCGGGGTTAACTCGGAGGAAGGTGGGGATGACGTCAAATCATCATGCCCTTTATGCCCAGGGCT<br>TCACACATGCTACAATGGTTAGTACAACGCGCTGCGAGCCTGTGAGGGTGAGCGAATCGCTGAAAGCTAATCTCAGTTCGG<br>ATTGGGGTCTGCAACTCGACCCCATGAAGTCGGAGTCGCTAGTAATCGCAGATCAGCAATGCTGCGGTGAATACGTTCCC<br>GGGCCTTGTACACACCGCCCGTCACGTCATGAAAGTTGGTAACACCCGAAGCCCATGGCCTAACCACCATTTTTGGTGGG<br>GGGAGTGGTCGAAGGTGGGATTGGCGATTGGGACG
43	Eubacterium sp. OTU_61	GATGAACGCTGGCGGCGTGCCTAACACATGCAAGTTGAGCGAGAAATTGCTTAAAGAAGCTTCGGTAGACTTAAGTAATGGA<br>GAGCAGCGGACGGGTGAGTAACGCGTGGGAAACCTGCCCTTGACAGGAGGATAGCCGAGAGAAATTTCGATTAATACTTCA<br>TAAAGCAGAGCATTCGCATGGATGAACTGCCAAAGAATTATCGGTCAAGGATGGTCCCGCGTCTGATTAGCTGGTTGGTAA<br>GGTAGCGGCTTACCAAGGCGACGATCAGTAGCCGGCCTGAGAGGGTGAACGGCCACATTGGAACTGAGACACGGTCCAG<br>ACTCCTACGGGAGGCAGCAGTGGGGAATATTGCACAATGGGCGAAAGCCTGATGCAGCAACGCCGCGTGAAGGAAGAAGG<br>CTTTCGAGTCGTAAACTTCTGTCCAAAGGGAAGAATAATGACGGTACCTTTGAAGAAAGCCCCGGCTAACTACGTGCCAGC<br>AGCCGCGGTAATACGTAGGGGGCGAGCGTTATCCGGAATTACTGGGCGTAAAGAGTATGTAGGTGGTTAAGTAAGCGTAGG<br>GTTTAAGGCGACAGCCCAACTGTCGTATGCCCCGCGAACTGTTTAACTTGAGTACAGGAGGGGAAGGCGGAATTCCTAGT<br>GTAGCGGTGAAATGCGTAGATATTAGGAGGAACACCAGTGGCGAAGGCGGCCTTCTGGACTGTAACTGACACTGAGATACG<br>AAAGCGTGGGTAGCAAACAGGATTAGATACCCTGGTAGTCCACGCCGTAAACGATGAGCACTAGGTGTCGGGCTCGCAAG<br>AGTTCGGTGCCGGAGCAAACGCATTAAGTGCTCCGCCTGGGGAGTACGCACGCAAGTGTGAAACTCAAAGGAATTGACGG<br>GGACCCGCACAAGCAGCGGAGCATGTGGTTTAATTCGAAGCAACGCGAAGAACCTTACCAGGACTTGACATCCCACTGAAA<br>GCTCGGTTAAAGCTGAGCCCTTCTTCGGAACAGTGGAGACAGGTGGTGCATGGTTGTCGTCAGCTCGTGTCGTGAGATGT<br>TGGGTTAAGTCCCGCAACGAGCGCAACCCTTGCCGTTAGTTGCCATCATTAAGTTGGGCACTCTAATGGGACTGCCGGGG<br>AGAACCCGGAGGAAGGCGGGGATGACGTCAAATCATCATGCCCCTTATGTTCTGGGCTACACACGTGCTACAATGGCCGT<br>CACAGAGGGAAGCGAGAGAGCGATCTTAAGCGAAACCAAAAAGGCGGTCCCAGTTCGGACTGCAGGCTGCAACTCGCCTG<br>CACGAAGCCGGAGTTGCTAGTAATCGCGGATCAGAATGTCGCGGTGAATGCGTTCCCGGGTCTTGTACACACCGCCCGT<br>CACACCATGGAAGTTGGGGGTGCCCAAAGTCGGTCAAGAAAAGGTCGCCTAAGGCAAAACCAATGACTGGGGTG
44	Schwartzia sp. OTU_63	GACGAACGCTGGCGGCGTGCTTAACACATGCAAGTCGAACGGAGCTATTTATTTCGGTAGATAGCTTAGTGGCAAACGGGT<br>GAGTAACGCGTAGGCAACCTGCCCTCAGGATGGGGACAACGGCTCGAAAGAGCCGCTAATACCGAATGACGTAACTCTTC<br>CACATGGAAGAGATACTAAAGATGGGTCAAACCATCGCCTAAGGATGGGCCTGCGTCTGATTAGCCAGTTGGTGAGGTAAC<br>GGCTCACCAAAGCGACGATCAGTAGCCGGTCTGAGAGGATGAACGGCCACAATGGGACTGAGACACGGCCCATACTCCTA<br>CGGGAGGCAGCAGTGGGGAATCTTCCGCAATGGGCGAAAGCCTGACGGAGCAACGCCGCGTGAGTGAAGAAGGTCTTCG<br>GATCGTAAAGCTCTGTTGTTGGGGACGAAAGAGTAGACGAGGAAATGCGTCTACTAAGACGGTACCTGACGAGGAAGCCAC<br>GGCTAACTACGTGCCAGCAGCCGCGGTAATACGTAGGCGGCAAGCGTTGTCCGGAATTATTGGGCGTAAAGGGAGCGCA<br>GGTGGGACGGTAAGTCCGTCTTAAAAGGCAGGGGCTCAGCCCCTGTAAGGGATGGAAACTATCGATCTTGAGTGCCGGAG<br>AGGAAAGCGGAATTCCCAGTGTAGCGGTGAAATGCGTAGATATTGGGAAGAACACCAGTGGCGAAGGCGGCTTTCTGGAC<br>GGCAACTGACACTGAGGCTCGAAAGCCAGGGGAGCGAACGGGATTAGATACCCCGGTAGTCCTGGCCGTAAACGATGGAT<br>ACTAGGTGTAGGAGGTATCGACCCCTCCTGTGCCGGAGTTAACGCAATAAGTATCCCGCCTGGGGAGTACGGTCGCAAGA<br>CTGAAACTCAAAGGAATTGACGGGGGCCCGCACAAGCGGTGGAGTATGTGGTTTAATTCGACGCAACGCGAAGAACCTTAC<br>CAGGGCTTGACATTGAGTGAAAGGGCAAGAGATTGTCCCCTTATCTTCGGATAACACGAAAACAGGTGGTGCATGGCTGTC<br>GTCAGCTCGTGTCGTGAGATGTTGGGTTAAGTCCCGCAACGAGCGCAACCCCTATATTCTGTTGCCAACAGGTAAAGCTG<br>GGAACTCAGGATAGACTGCCGCGGAGAACGCGGAGGAAGGCGGGGATGACGTCAAGTCATCATGCCCCATATGACCTGG<br>GCTACACACGTACTACAATGGGATGTACAGAGGGCAGCGAAAGAGCGATCTGGAGCGAACCCCAGAAAACATCTCCCAGTT<br>CGGATTGCAGGCTGAAACCCGCCTGCATGAAGATGGAATCGCTAGTAATCGCAGGTCAGCATACTGCGGTGAATACGTTC<br>CCGGGCCTTGTACACACCGCCCGTCACACCACGGAAGTCATTCACACCCGAAGCCGGTGGAAGCCGTCGAAGGTGGGG<br>GCGATGACTGGGGTG

Sr no.	Species_OTUs	16S reference sequence
45.	Treponema sp. OTU_65	AACGAACGCTGGCGGCGCGTCTTAACCATGCAAGTCGAGCGGCAGGCAGCAATGCCGAGAGCGGCGGACTGGTGAGTAACACGTGGATAACGTACCCCGATGCCCGGGACAGCCTGTAGAAATAGAGGGTAATACCGGATAGGTCACCGTTTTTGTAAGGAAGACGGCGGGAAAGGAGCTTCGGCTCCGCGCTGGGATCGGTCTGCGGCCCATCAAGCTCGTTGGTGAGGTAAGAGCTCACCAAGGCGAGGACGGGTATCCGGCCTGAGAGGGCGGACGGACACATTGGGACTGAGATACGGCCCAGACTCCTACGGGAGGCAGCAGGTAAGAATATTCCGCAATGGGGGGAACCCTGACGGAGCGACGCCGCGTGAACGAAGAAGGCCGGAAGGTTGTAAAGTTCTTTTCTGTTCGAGGAATAAGTGTCGGAGGAAATGCCGACATGGTGACGGTAGGGCAGGAATAAGCACCGGCTAATTACGTGCCAGCAGCCGCGGTAACACGTAAGGTGCGAGCGTTGTTCGGAATTATTGGGCGTAAAGGGCATGCAGGCGGGACGCCAAGCTTGATGAGAAATACCGGGGCTCAACTCCGGAGCTATATTGAGAACTGGCGAGCTAGAGTTGCCGAAGGGTATCCGGAATTCCGCGTGAAGGGGTGAAATCTGTAGATATGCGGAAGAACACCGATGGCGAAGGCAGGATACCGGCGGACGACTGACGCTGAGGTGCGAAGGTGCGGGGAGCAAACAGGATTAGATACCCTGGTAGTCCGCACAGTCAACGATGTACACTGGGCGTGTGCGCAAGAGCGTGCGTGCCGAAGCAAACGCGATAAGTGTACCGCCTGGGGAGTATGCCCGCAAGGGTGAAACTCAAAGGAATTGACGGGGGCCCGCACAAGCGGTGGAGCATGTGGTTTAATTCGATGGTACGCGAGGAACCTTACCTGGGTTTGACATCAAGAGGGATCATATAGAGATATGTGAGCGTAGCAATACGGCTCTTGACAGGTGCTGCATGGCTGTCGTCAGCTCGTGCCGTGAGGTGTTGGGTTAAGTCCCGCAACGAGCGCAACCCCTACTGCCGGTTACTAACAGGTAGCGCTGAGGACTCAGGCGGAACTGCCTGCGACAAGCAGGAGGAAGGCGGGGACGACGTCAAGTCATCATGGCCCTTATGTCCAGGGCTACACACGTGCTACAATGGCCGCCACAGAGCGGGGCGAAGCCGAGAGGCGGAGCAGAACGCAGAAAAGCGGTCGTAGTCCGGATTGAAGTCTGAAACTCGACTTCATGAAGCTGGAATCGCTAGTAACCGCACATCAGCACGGCGCGGTGAATACGTTCCCGGGCCTTGTACACACCGCCCGTCACACCATCCGAGCAGGGGGTACCCGAAGCCGGCAGTCCAACCGCAAGGGGGACGCTGTCGAAGGTACGCTTTGTGAGGGGGGTG
46	Lachnoanaerobaculum sp. OTU_67	GATGAACGCTGGCGGCGTGCTTAACACATGCAAGTCGAACGAAGCTGCTTAAAGGAAGTCTTCGGATGGAATTTAGGTAGACTTAGTGGCGGACGGGTGAGTAACGCGTGGATAACCTGCCTTATACAGGGGGATAACGGAGAGAAATTTCCGCTAAGACCGCATAAGACCACAGCACCGCATGGTGCAGGGGTAAAATATTTATAGGTATAAGATGGATCCGCGTCCGATTAGCTAGTTGGTGAGGTAAAGGCCCACCAAGGCGACGATCGGTAGCCGGCCTGAGAGGGTGAACGGCCACATTGGGACTGAGACACGGCCCAAACTCCTACGGGAGGCAGCAGTGGGGAATATTGGACAATGGGGGAAACCCTGATCCAGCGACGCCGCGTGAGTGAAGAAGTATTTCGGTATGTAAAGCTCTATCAGCAGGGAAGAAAATGACGGTACCTGACTAAGAAGCCCCGGCTAACTACGTGCCAGCAGCCGCGGTAATACGTAGGGGGCAAGCGTTATCCGGATTTACTGGGTGTAAAGGGAGCGCAGACGGCAATGCAAGTCTGAAGTGAAAGGCGTGGGCTCAACCCATGAACTGCTTTGGAAACTGTATAGCTTGAGTGTCGGAGGGGTAAGCGGAATTCCTAGTGTAGCGGTGAAATGCGTAGATATTAGGAGGAACACCGGAGGCGAAGGCGGCTTACTGGACGACAACTGACGTTGAGGCTCGAAGGCGTGGGGAGCAAACAGGATTAGATACCCTGGTAGTCCACGCAGTAAACGATGAATACTTGGTGTCGGGGAGGTAAACTCTTCGGTGCCGCAAGCTAACGCATTAAGTATTCCACCTGGGGAGTACGTTCGCAAGAATGAAACTCAAAGGAATTGACGGGGACCCGCACAAGCGGTGGAGCATGTGGTTTAATTCGAAGCAACGCGAAGAACCTTACCAAATCTTGACATACTCTTGAATAACTTTGTAATGAAGTTAGTCTTTCGGGACAAGGGATACAGGTGGTGCATGGTTGTCGTCAGCTCGTGTCGTGAGATGTTGGGTTAAGTCCCGCAACGAGCGCAACCCTTGTCGTCAGTAGCCAGCAGTAAGATGGGCACTCTGACGAGACAGCCGGAGATAATCCGGAGGAAGGTGGGGATGACGTCAAATCATCATGCCCCTTATGATTTGGGCTACACACGTGCTACAATGGCGTAAACAAAGTGAAGCAAAGCCGCGAGGCCAAGCAAATCACAAAAATAACGTCTCAGTTCGGATTGTAGTCTGCAACTCGACTATATGAAGCTGGAATCGCTAGTAATCGCAGATCAGAATGCTGCGGTGAATACGTTCCCGGGTCTTGTACACACCGCCCGTCACACCATGGGAGTCATCAATGCCCGAAGTCAGTGACCTAACCGTAAGGAAGGAGCTGCCGAAGGCAGGGAGGATAACTGGGGTG
47	Parvimonas sp. OTU_69	GACGAACGCTGGCGGCGTGCTTAACACATGCAAGTCGAACGTGATTTTTGTGGAAAGCCCTTCGGGGACGGAAGCGAAATGAAAGTGGCGAACGGGTGAGTAACACGTGAGCAACCTACCTTACACAGGGGGATAGCCGTTGGAAACGACGATTAATACCGCATGAGACCACAAAGCCACATGGCTTAGGGGTAAAAGATTTATCGGTGTAAGATGGGCTCGCGTCTGATTAGCTAGTTGGAAGGGTAAAGGCCTACCAAGGCAACGATCAGTAGCCGGTCTGAGAGGATGAACGGCCACATTGGAACTGAGACACGGTCCAAACTCCTACGGGAGGCAGCAGTGGGGAATATTGCACAATGGGGGGAACCCTGATGCAGCGACGCCGCGTGAGCGAAGAAGGTTTTCGAATCGTAAAGCTCTGTCCTATGAGAAGATAATGACGGTATCATAGGAGGAAGCCCCGGCTAAATACGTGCCAGCAGCCGCGGTAATACGTATGGGGCGAGCGTTGTCCGGAATTATTGGGCGTAAAGGGTACGTAGGCGGCCTATTAAGTCAGGTGTGAAAGCGTGAGGCTTAACCTCATTAAGCACTTGAAACTGGAAGGCTTGAGTGAAGGAGAGGAAAGTGGAATTCCTAGTGTAGCGGTGAAATGCGTAGATATTAGGAGGAATACCGGTGGCGAAGGCGACTTTCTGGACTTTTACTGACGCTCAGGTACGAAAGCGTGGGGAGCAAACAGGATTAGATACCCTGGTAGTCCACGCCGTAAACGATGAATGCTAGGTGTTGGGAGTCAAATCTCGGTGCCGAAGTTAACACATTAAGCATTCCGCCTGGGGAGTACGGTGGCAACACTGAAACTCAAAGGAATTGACGGGGACCCGCACAAGCAGCGGAGCATGTGGTTTAATTCGAAGCAACGCGAAGAACCTTACCAAGGCTTGACATATAGTTGAGTTATTGAGAAATTGATAAGTCCCTCGGGACAACTATACAGGTGGTGCATGGTTGTCGTCAGCTCGTGTCGTGAGATGTTGGGTTAAGTCCCGCAACGAGCGCAACCCTTATCTTCAGTTACCAGCATGTAATGATGGGGACTCTGGAGAGACTGCCGATGACAAATCGGAGGAAGGTGGGGATGACGTCAAATCATCATGCCCTTTATGTCTTGGGCTACACACGTGCTACAATGGTTGGTACAACGAGAAGCTAGACAGTGATGTTAAGCAAAACTCTAAAAACCAATCTCAGTTCGGATTGTAGGCTGCAACTCGCCTACATGAAGTCGGAGTTGCTAGTAATCGCGAATCAGAATGTCGCGGTGAATGCGTTCCCGGGTCTTGTACACACCGCCCGTCACACCATGGGAGTTGGCAATACCCGAAGCCGCCGATCTAACCGCAAGGAGGAAGGCGTCGAAGGTAGGGTTAATGACTGGGGTG
48	Porphyromonas sp. OTU_70	GATGAACGCTAGCGATAGGCTTAACACATGCAAGTCGAGGGGCAGCGAGATGTAGCAATACGTCGTCGGCGACCGGCGAATGGGTGAGTAACACGTATGCAACTTACCTCTTAGTGGTGAATAACCCGATGAAAGTCGGACTAATACACCATACTCTCCTTAGATCACATGAGAAGAGGAGGAAAGATTAATCGCTAAGAGATAGGCCTGCGTTCCATTAGCTAGTTGGTAAGGTAACGGCTTACCAAGGCAACGATGGATAGGGGGACTGAGAGGTTGACCCCCCACATTGACACTGAGATACGGGTCAAACTCCTACGGGAGGCAGCAGTGAGGAATATTGGTCAATGGGCGAGAGCCTGAACCAGCCAAGTCGCGTGAAGGAAGACTGCCCGCAAGGGTTGTAAACTTCTTTTGTATGGGATTAAAGTCGTCTACGTGTAGGCGTTTGCAGTTACCATACGAATAAGCATCGGCTAACTCCGTGCCAGCAGCCGCGGTAATACGGAGGATGCGAGCGTTATCCGGAATTATTGGGTTTAAAGGGTGCGTAGGTTGCAAGGGAAGTCAGGGGTGAAAAGCTGTAGCTCAACTATGGTCTTGCCTTTGAAACTCTCTAGCTAGAGTGTACTGGAGGTACGTGGAACGTGTGGTGTAGCGGTGAAATGCATAGATATCACACAGAACTCCGATTGCGCAGGCAGCGTACTACATTACAACTGACACTGAAGCACGAAAGCGTGGGTATCAAACAGGATTAGATACCCTGGTAGTCCACGCAGTAAACGATGAATACTAGATCTATGCGATATACAGTATGGGTCTAAGCGAAAGCGATAAGTATTCCACCTGGGGAGTACGCCGGCAACGGTGAAACTCAAAGAGATTGGCGGGGGTCCGCACAAGCGGAGGAACATGTGGTTTAATTCGATGATACGCGAGGAACCTTACCCGGGATTGAAATGTAGATGCATGAGGCTGAGAGGTCTCTTCCCTTCGGGGCTTCTATGTAGGTGCTGCATGGTTGTCGTCAGCTCGTGCCGTGAGGTGTCGGCTTAAGTGCCATAACGAGCGCAACCCGCGTCGATAGTTACTAACGAGTCATGTCGAGGACTCTATCGAGACAGCCGTCGTAAGACGTGAGGAAGGAGCGGATGACGTCAAATCAGCACGGCCCTTACATCCGGGGCGACACACGTGTTACAATGGTAGGGACAGCGAGCAGCCATCTGGTGACAGAGAGCTAATCTATAAACCCTATCCCAGTTCGGATCGGAGTCTGCAACTCGACTCTGTGAAGCTGGATTCGCTAGTAATCGCGCATCAGCCATGGCGCGGTGAATACGTTCCCGGACCTTGCACACACCGCCCGTCAAGCCATGGGAGTCGGGGGTACCTGAAGAGCGTGACCGTCACAGGAGCGCTTGAGGGTAAAACTGGTGACTGGGGCT

Sr no.	Species_OTUs	16S reference sequence
49	Prevotella sp. OTU_72	GATGAACGCTGGCTACAGGCTTAACACATGCAAGTCGAGGGGCAGCATGGCGGATGCTTGCATCTGCCGATGGCGACCG<br>GCGCACGGGTGAGTAACGCGTATCCAACCTACCTTCAAGCAGGGGACAATCCGTAGAAATGCGGTCTGATACCCTATGTAT<br>TCCAACGAGGTCCTCCGTGTTGGAAGAAAGGTTTATCCGCTTGTTGATGGGGATGCGTCCGATTAGCTTGTTGGCGGGGC<br>AACGGCCCACCAAGGCAACGATCGGTAGGGGTTCTGAGAGGAAGGTCCCCCACACTGGAACTGAGACACGGTCCAGACT<br>CCTACGGGAGGCAGCAGTGAGGAATATTGGTCAATGGGCGTAAGCCTGAACCAGCCAAGTAGCGTGCAGGATGACGGCC<br>CTATGGGTTGTAAACTGCTTTTATACGGGGATAAAGTGCGCCACGTGTGGTGTTTTGCAGGTACCGTATGAATAAGGACCG<br>GCTAATTCCGTGCCAGCAGCCGCGGTAATACGGAAGGTCCGGGCGTTATCCGGATTTATTGGGTTTAAAGGGAGCGTAGG<br>CCGCATGTCAAGCGTGTTGTGAAATGCAGGTGCTCAACACCTGGATTGCAGCGCGAACTGCCGTGCTTGAGTGTGCGCAA<br>CGTTGGCGGAATTCGTCGTGTAGCGGTGAAATGCTTAGATATGACGAGGAACCCCGATTGCGAAGGCAGCTGACGGGAGC<br>ATAACTGACGCTGAAGCTCGAAGGTGCGGGTATCGAACAGGATTAGATACCCTGGTAGTCCGCACGGTAAACGATGGATGC<br>CCGCCGTCCGCCCTTTTATTGGGCGTGTGGCCAAGCGAAAGCATTAAGCATCCCACCTGGGGAGTACGCCGGCAACGGT<br>GAAACTCAAAGGAATTGACGGGGGCCCGCACAAGCGGAGGAACATGTGGTTTAATTCGATGATACGCGAGGAACCTTACCC<br>GGGCTTGAATTGCAGAGGAACGATCCAGAGATGGTGAGGCCCTTCGGGGCTTCTGCGAAGGTGCTGCATGGTTGTCGTCA<br>GCTCGTGCCGTGAGGTGTCGGCTCAAGTGCCATAACGAGCGCAACCCCTCTCCGCAGTTGCCATCGGGTTATGCCGGG<br>CACTCTGTGGACACTGCCGCCGCAAGGTGTGAGGAAGGTGGGGATGACGTCAAATCAGCACGGCCCTTACGTCCGGGGC<br>TACACACGTGTTACAATGGGGGGCACAGCAAGCCGGCCGCGTGCAAACGCGGTCCAATCCTGAAATCCCCCCTCAGTTC<br>GGACTGGGGTCTGCAACCCGACCCCACGAAGCTGGATTCGCTAGTAATCGCGCATCAGCCATGGCGCGGTGAATACGTT<br>CCCGGGCCTTGTACACACCGCCCGTCAAGCCATGAAAGCCGGGGGTGCCTGAAGTCCGTGACCGTAAGGATCGGCCTA<br>GGGCAAAACTGGTGATTGGGGCT
50	Selenomonas sp. OTU_73	GACGAACGCTGGCGGCGTGCTTAACACATGCAAGTCGAACGGAGCGAATGAAAGCTTGCTTTTATGAGCTTAGTGGCAAAC<br>GGGTGAGTAACACGTAGACAACCTGCCGATCAGATGGGGACAACATTCCGAAAGGAATGCTAATACCGAATGAAGTGTGGG<br>GGAGGCATCTTCCCTACATGAAAGATGGCCTCTATTTATAAGCTATCACTGATCGATGGGTCTGCGTCTGATTAGCTAGTAG<br>GTAAGGTAACGGCTTACCTAGGCGACGATCAGTAGCCGGTCTGAGAGGATGAACGGCCACATTGGGACTGAGACACGGCC<br>CAGACTCCTACGGGAGGCAGCAGTGGGGAATCTTCCGCAATGGGCGCAAGCCTGACGGAGCAACGCCGCGTGAGTGAAG<br>AAGGTCTTCGGATCGTAAAGCTCTGTTGAAGGGGACGAACATACATAAGTTGAATAAGCTTATGTAATGACGGTACCTTTTGA<br>GGAAGCCACGGCTAACTACGTGCCAGCAGCCGCGGTAATACGTAGGTGGCGAGCGTTGTCCGGAATCATTGGGCGTAAA<br>GGGAGCGCAGGCGGCCATGTAAGTCTTGCTTAAAAGTTCGGGGCTCAACCCCGTGATGGGCAAGAAACTATATGGCTTGA<br>GTGCAGGAGAGGAAAGCGGAATTCCCAGTGTAGCGGTGAAATGCGTAGATATTGGGAGGAACACCAGTGGCGAAGGCGGC<br>TTTCTGGACTGCAACTGACGCTGAGGCTCGAAAGCCAGGGGAGCGAACGGGATTAGATACCCCGGTAGTCCTGGCCGTAA<br>ACGATGGATACTAGGTGTGGGAGGTATCGACCCCTACCGTGCCGGAGTTAACGCAATAAGTATCCCGCCTGGGGAGTACG<br>GTCGCAAGACTGAAACTCAAAGGAATTGACGGGGACCCGCACAAGCGGTGGAGTATGTGGTTTAATTCGAAGCAACGCGAA<br>GAACCTTACCAGGTCTTGACATTGATTGACAGGGATAGAGATATCCTATTCTCTTTGGAGACAAGAAAACAGGTGGTGCATG<br>GCTGTCGTCAGCTCGTGTCGTGAGATGTTGGGTTAAGTCCCGCAACGAGCGCAACCCCTGTCCTTTGTTGCCAGCGCGT<br>AAAGGTGGGCACTCAAAGGAGACTGCCGCGGAGAACGCGGAGGAAGGCGGGGATGACGTCAAGTCATCATGCCCCTTAT<br>GACCTGGGCTACACACGTACTACAATGGGACGGACAGAGAGCAGCGAGAGGGCGACCTTAAGCGAACCTCAAAAACCGTT<br>TCTCAGTTCGGATTGCAGGCTGCAACTCGCCTGCATGAAGTCGGAATCGCTAGTAATCGCAGGTCAGCATACTGCGGTGA<br>ATACGTTCCCGGGTCTTGTACACACCGCCCGTCACACCACGGAAGTCGTTCACACCCGAAGCCGGCGCAGCCGTCTAAG<br>GTGGGGAAGGTGACTGGGGTG
51	Cardiobacterium sp. OTU_76	ATTGAACGCTGGCGGCATGCTTAACACATGCAAGTCGAACGGAAACGACGGAGCTTGCTCCGGGCGTCGAGTGGCGGAC<br>GGGTGAGTAATGCATGGGAATCTGCCTTTTGCTGGGGGATAACGTAGGGAAACTTACGCTAATACCGCATAAGACCTGAGG<br>GTGAAAGTGGGGGACCGCAAGGCCTCATGGCAGAAGATGAGCCCATGTCGGATTAGCTAGTTGGTGGGGTAAGGGCCTAC<br>CAAGGCGACGATCCGTAGCTGGTCTGAGAGGATGATCAGCCACACTGGGACTGAGACACGGCCCAGACTCCTACGGGAG<br>GCAGCAGTGGGGAATATTGGACAATGGGGGCAACCCTGATCCAGCAATGCCGCGTGTGTGAAGAAGGCCTTCGGGTTGTA<br>AAGCACTTTCAGCAGGGAGGAAAGAGCCATGGCTAATATCCATGGAAGTGACGTTACCTGCAGAAGAAGCACCGGCTAACT<br>CCGTGCCAGCAGCCGCGGTAATACGGAGGGTGCGAGCGTTATTCGGAATTACTGGGCGTAAAGCGCACGCAGGCGGTTA<br>TTTAAGTCAGATGTGAAAGCCCTGGGCTTAACCTAGGAACTGCATTTGAAACTGAATGGCTAGAGTATGAAAGAGGAAAGCG<br>GAATTTCCAGTGTAGCAGTGAAATGCGTAGATATTGGAAGGAACACCGATGGCGAAGGCAGCTTTCTGGGTCGATACTGAC<br>GCTCATGTGCGAAAGCGTGGGGAGCAAACAGGATTAGATACCCTGGTAGTCCACGCCCTAAACGATGTCAACTAGGTGTTG<br>GGTTTTTTAAGACTCGGTGCCGCAGCTAACGCATTAAGTTGACCGCCTGGGGAGTACGGCCGCAAGGTTGAAACTCAAAGA<br>AATTGACGGGGACCCGCACAAGCGGTGGAGCATGTGGTTTAATTCGATGCAACGCGAAGAACCTTACCAGGCCTTGACAT<br>CCAGAGAACTTGGCAGAGATGCTTAGGTGCCTTCGGGAACTCTGAGACAGGTGTTGCATGGCTGTCGTCAGCTCGTGTCG<br>TGAGATGTTGGGTTAAGTCCCGCAACGAGCGCAACCCTTATCCTTACTTGCCAGCACTTAGGGTGGGAACTATAAGGAGAC<br>TGCCGGTGACAAGCCGGAGGAAGGCGGGGATGACGTCAAGTCATCATGGCCCTTACGGCCTGGGCTACACACGTGCTAC<br>AATGGTCGGTACAGACGGTCGCGAAGCCGCGAGGTGGAGCCAATCTGAGAAAGCCGATCGTAGTCCGGATTGCAGTCTG<br>CAACTCGACTGCATGAAGTCGGAATCGCTAGTAATCGCGAATCAGCATGTCGCGGTGAATACGTTCCCGGGTCTTGTACAC<br>ACTGCCCGTCACACCATGGGAGTTTGTTGCACCAGAAGCAGGCAGCTTAACGAAAGAGGGCGCTTGCCACGGTGTGGCC<br>GATGACTGGGGTG
52	Neisseria sp. OTU_77	ATTGAACGCTGGCGGCATGCTTTACACATGCAAGTCGGACGGCAGCACAGAGAAGCTTGCTTCTTGGGTGGCGAGTGGCG<br>AACGGGTGAGTAATATATCGGAACGTACCGAGTAATGGGGGATAACTAATCGAAAGATTAGCTAATACCGCATATTCTCTGAG<br>GAGGAAAGCAGGGGACCTTCGGGCCTTGCGTTATTCGAGCGGCCGATATCTGATTAGCTAGTTGGTGGGGTAAAGGCCTA<br>CCAAGGCGACGATCAGTAGCGGGTCTGAGAGGATGATCCGCCACACTGGGACTGAGACACGGCCCAGACTCCTACGGGA<br>GGCAGCAGTGGGGAATTTTGGACAATGGGCGCAAGCCTGATCCAGCCATGCCGCGTGTCTGAAGAAGGCCTTCGGGTTG<br>TAAAGGACTTTTGTCAGGGAAGAAAAGGCTGTTGCTAATACCGACAGCTGATGACGGTACCTGAAGAATAAGCACCGGCTAA<br>CTACGTGCCAGCAGCCGCGGTAATACGTAGGGTGCGAGCGTTAATCGGAATTACTGGGCGTAAAGCGAGCGCAGACGGTT<br>ACTTAAGCAGGATGTGAAATCCCCGGGCTCAACCTGGGAACTGCGTTCTGAACTGGGTGACTAGAGTGTGTCAGAGGGAG<br>GTAGAATTCCACGTGTAGCAGTGAAATGCGTAGAGATGTGGAGGAATACCGATGGCGAAGGCAGCCTCCTGGGATAACACT<br>GACGTTCATGCTCGAAAGCGTGGGTAGCAAACAGGATTAGATACCCTGGTAGTCCACGCCCTAAACGATGTCAATTAGCTG<br>TTGGGCAACTTGATTGCTTAGTAGCGTAGCTAACGCGTGAAATTGACCGCCTGGGGAGTACGGTCGCAAGATTAAAACTCA<br>AAGGAATTGACGGGGACCCGCACAAGCGGTGGATGATGTGGATTAATTCGATGCAACGCGAAGAACCTTACCTGGTCTTGA<br>CATGTACGGAATCCTCCAGAGACGGAGGAGTGCCTTCGGG

Sr no.	Species_OTUs	16S reference sequence
53	Olsenella sp. OTU_79	GATGAACGCTGGCGGCACGCTTAACACATGCAAGTCGAACGATTAAAGCACCTTCGGGTGTGTATAGAGTGGCGAACGGC<br>TGAGTAACACGTGGGCAACCTGCCCTTCAGTTCGGAATAGCTACGGGAAACCGTGGGTAATACCGGATGATCCACCTCCC<br>TCGCATGAAGGAGGTGGCAAAGATTTATCGCTGAAGGATGGGCCCGCGGCCTGTTAGCTTGTTGGTGAGGTAACGGCTCA<br>CCAAGGCAGTAATGGGTAGCCGGGTTGAGAGACCGACCGGCCAGATTGGGACTGAGACACGGCCCAGACTCCTACGGGA<br>GGCAGCAGTGGGGAATCTTGCGCAATGGGCGAAAGCCTGACGCAGCGATGCCGCGTGCGGGATGAAGGCCTTCGGGTC<br>GTAAACCGCTTTCAGCAGGGACGAGGCCGCAAGGTGACGGTACCTGCAGAAGAAGCCCCGGCTAACTACGTGCCAGCAG<br>CCGCGGTAATACGTAGGGGGCGAGCGTTATCCGGATTCATTGGGCGTAAAGCGCGCGTAGGCGGCCTGTTAGGTCAAGA<br>GTTAAATCCGGGGGCTCAACCCCCGTCCGCTCCTGATACCGGCAGGCTTGAGTCTGGTAGGGGAAGGCGGAATTCCAAG<br>TGTAGCGGTGGAATGCGCAGATATTTGGAAGAACACCGGTGGCGAAGGCGGCCTTCTGGGCCACGACTGACGCTGAGGC<br>GCGAAAGCTAGGGGAGCAAACAGGATTAGATACCCTGGTAGTCCTAGCCGTAAACGATGGACACTAGGTGTGGGGGGAAC<br>ATCCCTCCGTGCCGCAGCTAACGCATTAAGTGTCCCGCCTGGGGAGTACGGCCGCAAGGCTAAAACTCAAAGGAATTGAC<br>GGGGGCCCGCACAAGCAGCGGAGCATGTGGCTTAATTCGAAGCAACGCGAAGAACCTTACCAGGGCTTGACATATAGGTG<br>AAGCGGTGGAAACACCGTAGCCGAAAGGAGCCTATACAGGTGGTGCATGGCTGTCGTCAGCTCGTGTCGTGAGATGTTGG<br>GTTAAGTCCCGCAACGAGCGCAACCCCCGTCGTATGTTGCCAGCGGTTCGGCCGGGCACCCATACGAGACCGCCGGCG<br>TCAAGCCGGAGGAAGGTGGGGACGACGTCAAGTCATCATGCCCCTTATGTCCTGGGCTGCACACGTGCTACAATGGCCG<br>GCACAATGGGCTGCCAAGTCGCAAGACCGAGCGAATCCCTAAAGCCGGCCCCAGTTCGGATTGGAGGCTGCAACCCGCC<br>TCCATGAAGTCGGAGTTGCTAGTAATCGCGGATCAGCACGCCGCGGTGAATGCGTTCCCGGGCCTTGTACACACCGCCC<br>GTCACACCACCCGAGTCGTCTGCACCCGAAGTCGCCGGCCCAACCTTTTGGAGGGAGGCGCCGAAGGTGTGGAGGGTA<br>AGGGGGGTG
54	Selenomonas sp. OTU_80	GACGAACGCTGGCGGCGTGCTTAACACATGCAAGTCGAACGGGATGATCTTAAAGCTTGCTTTTAGAGAATCTAGTGGCAA<br>ACGGGTGAGTAACACGTAGACAACCTGCCGACAGGATGGGGACAACATTCCGAAAGGAATGCTAATACCGAATGGAGCGGA<br>GGAGAGGCATCTCTCTTCCGTGAAAGATGGCCTCTGAACATGCTATCACCTATCGATGGGTCTGCGTCTGATTAGCTGGTT<br>GGTGAGGTAACGGCTCACCAAGGCGACGATCAGTAGCCGGTCTGAGAGGATGAACGGCCACATTGGGACTGAGACACGG<br>CCCAGACTCCTACGGGAGGCAGCAGTGGGGAATCTTCCGCAATGGGCGCAAGCCTGACGGAGCAACGCCGCGTGAGTG<br>AAGAAGGTCTTCGGATCGTAAAGCTCTGTTGACGGGGACGAACGTGCGAGAGGTGAATAATCTCTTGTAATGACGGTACCT<br>GTCGAGGAAGCCACGGCTAACTACGTGCCAGCAGCCGCGGTAATACGTAGGTGGCGAGCGTTGTCCGGAATCATTGGGC<br>GTAAAGGGAGCGCAGGCGGGCATGTAAGTCTTTCTTAAAAGTTCGGGGCTCAACCCCGTGATGGGAAAGAAACTATATGTC<br>TTGAGTACAGGAGAGGAAAGCGGAATTCCCAGTGTAGCGGTGAAATGCGTAGATATTGGGAGGAACACCAGTGGCGAAGGC<br>GGCTTTCTGGACTGCAACTGACGCTGAGGCTCGAAAGCCAGGGGAGCGAACGGGATTAGATACCCCGGTAGTCCTGGCC<br>GTAAACGATGGATACTAGGTGTGGGAGGTATCGACCCCTACCGTGCCGGAGTTAACGCAATAAGTATCCCGCCTGGGGAG<br>TACGGTCGCAAGACTGAAACTCAAAGGAATTGACGGGGACCCGCACAAGCGGTGGAGTATGTGGTTTAATTCGAAGCAACG<br>CGAAGAACCTTACCAGGCCTTGACATTGACTGAAAGTGCTAGAGATAGCATCCTCTCTTCGGAGACAGGAAAACAGGTGGT<br>GCATGGCTGTCGTCAGCTCGTGTCGTGAGATGTTGGGTTAAGTCCCGCAACGAGCGCAACCCCTGTTCTTTGTTGCCATC<br>ACGTAAAGGTGGGCACTCAAAGGAGACTGCCGCGGAGAACGCGGAGGAAGGCGGGGATGACGTCAAGTCATCATGCCCC<br>TTATGGTCTGGGCTACACACGTACTACAATGGAACGGACAGAGAGCAGCGAACCCGCGAGGGCAAGCGAACCTCAAAAAC<br>CGTTTCCCAGTTCGGATTGCAGGCTGCAACTCGCCTGCATGAAGTCGGAATCGCTAGTAATCGCAGGTCAGCATACTGCG<br>GTGAATACGTTCCCGGGTCTTGTACACACCGCCCGTCACACCACGGAAGTCGTTCACACCCGAAGCCGGCGCAGCCGTC<br>TAAGGTGGGGAAGGTGACTGGGGTG
55	Fudania sp. OTU_81	GACGAACGCTGGCGGCGTGCTTAACACATGCAAGTCGAACGATGAAGCGCTGTCTTTTTGATGGTGTGGATTAGTGGCGAA<br>CGGGTGAGTAATACGCGGGCAACCTGCCCCTCTGCTTCGGGATAAGCTTTGGAAACGGGGTCTAATACCGGATGTGCCTG<br>TCTGGTCGCATGGCTGGGCGGGGAAAGATGTTTTTCGGCGGGGGATGGGCTCGCGGCCTATCAGCTTGTTGGTGGGGTG<br>ACGGCCTACCAAGGCGTCGACGGGTAGCCGGCCTGAGAGGGTGGTCGGCCACACTGGGACTGAGATACGGCCCAGACT<br>CCTACGGGAGGCAGCAGTGGGGGATATTGCACAATGGGCGCAAGCCTGATGCAGCGACGCCGCGTGGGGGATGGAGGC<br>TTTCGGGTTGTAAACCCCTTTCGGCGCGGGACAAGGCTCCGTTTGGCGGGGTTGAGGGTACGCGTCAAAGAAGCGCCGG<br>CTAACTACGTGCCAGCAGCCGCGGTAATACGTAGGGCGCGAGCGTTGTCCGGAATTATTGGGCGTAAAGGGCTCGTAGG<br>CGGCTTGTCGCGTCTGCTGTGAAAACGCGGGGCTCAACTCCGCGCGTGCAGTGGGTACGGGCAGGCTGGAGTGCGGCA<br>GGGGTGGCTGGAATTCCTGGTGTAGCGGTGGAATGCGCAGATATCAGGAAGAACACCGATGGCGAAGGCAGGCCACTGG<br>GCCGTCACTGACGCTGAGGAGCGAAAGCGTGGGGAGCGAACAGGATTAGATACCCTGGTAGTCCACGCTGTAAACGTTGG<br>GAACTAGGTGTGGGGTCTGTTCCACGGATTCTGCGCCGGAGCTAACGCGTTAAGTTCCCCGCCTGGGGAGTACGGCCGC<br>AAGGCTAAAACTCAAAGGAATTGACGGGGGCCCGCACAAGCGGCGGAGCATGCGGATTAATTCGATGCAACGCGAAGAAC<br>CTTACCAAGGCTTGACATGTGCCGGGAGGGCGCGGAGACGCGTCTGCCTTCGGGCCGGTTCACAGGTGGTGCATGGTT<br>GTCGTCAGCTCGTGTCGTGAGATGTTGGGTTAAGTCCCGCAACGAGCGCAACCCTTGTCCCGTGTTGCCAGCGCGTTGT<br>GGCGGGGACTCACGGGGGACTGCCGGGGTCAACTCGGAGGAAGGTGGGGACGACGTCAAATCATCATGCCCCTTATGC<br>CTTGGGCTTCACGCATGCTACAATGGCCGGTACAGAGGGTGGCGATGCCGTGAGGCGGAGCGAATCTCGTAAAGCCGGT<br>CTCAGTTCGGATCGGGGTCTGCAACTCGGGCCCGTGAAGTCGGAGTCGCTAGTAATCGCAGATCAGCAACGCTGCGGTG<br>AATACGTTCTCGGGCCTTGTACACACCGCCCGTCACGTCACGAAAGTCGGCGACGCCCGAAGCCCGTGGCTCTGCTTGT<br>GCGGGGAGCGGTCGAAGGCGGGGTTGGCGATTGGGACG
56	Leptotrichia sp. OTU_83	GATGAACGCTGACAGAATGCTTAACACATGCAAGTCTTTGGCAAAGCTGTGCTTGCACAGCCAAGCCAAGGCGGACGGGT<br>GAGTAACGCGTAAAGAACTTGTCCCGCAGACAGGGATAACAGACGGAAACGACTGATAAAACCTGATATAATTGCCGGTACG<br>CATGTGCCTGGCAATGAAAAGAGATGCTGCGGGAGAGCTTTGCGTCCTATTAGCTGGTTGGCGGGGTAATGGCCCACCAA<br>GGCGATGATAGGTAGCCGGCCTGAGAGGGTGAACGGCCACAAGGGGACTGAGATACGGCCCTTACTCCTACGGGAGGCA<br>GCAGTGGGGAATATTGGACAATGGGGGCAACCCTGATCCAGCAATTCTGTGTGCACGAAGAAGGTTTTCGGATTGTAAAGT<br>GCTTTCAGCAGGGAAGAAGAGAATGACGGTACCTGCAGAAGAAGCGACGGCTAAATACGTGCCAGCAGCCGCGGTAATAC<br>GTATGTCGCAAGCGTTATCCGGAATTATTGGGCATAAAGGGCATCTAGGCGGCCCTGTAAGTCTAGGGTGAAAACCTGCGG<br>CTCAACCGCAGGCCTGCCCCGGAAACTATAGGGCTGGAGTACTGGAGAGGTGGACGGAACTACACGAGTAGAGGTGAAAT<br>TCGTAGATATGTGTAGGAATGCCGATGATGAAGATAGTTCACTGGACGGTAACTGACGCTGAAGTGCGAAAGCTGGGGGAG<br>CAAACAGGATTAGATACCCTGGTAGTCCCAGCCGTAAACGATGATTACTGGGTGTGGGCATGAAGAGTGTCCGTGCCGAAG<br>CAAATGCGATAAGTAATCCGCCTGGGGAGTACGGCCGCAAGGCTGAAACTCAAAGGAATTGACGGGGACCCGCACAAGCG<br>GTGGAGCATGTGGTTTAATTCGACGCAACGCGAGGAACCTTACCAGATCTTGACATCCTACGAATGCCTGTGAAAGCAGGC<br>AGTGCCTTCGGGAACGTAGAGACAGGTGGTGCATGGCTGTCGACAGCTCGTGTCGTGAGATGTTGGGTTAAGTCCCGCAA<br>CGAGCGCAACCCCTATCGCCAGTTGCCATCATTAAGTTGGGGACTCTGGCGAGACTGCCTGCGAAGAGCAGGAGGAAGG<br>TGGGGATGACGTCAAGTCATCATGCCCCTTATGATCTGGGCTACACACGTGCTACAATGGCCGGTACAAAAAGCTGCAAAA<br>CGGCAACGTTTAGCCAATCTTTAAAGCCGGTCCAAGTTCAGATTGAAGTCTGCAACTCGACTTCATGAAGCCGGAATCGCT<br>AGTAATCGCAGATCAGCAATGCTGCGGTGAATACGTTCTCGGGTCTTGTACACACCGCCCGTCACACCACGAGAGTTGTTT<br>GCACCTGAAGCTGCCGGTCCAACCGCAAGGGGGAAGGCATCTAAGGTGTGGACAGTGATTGGGGTG

Sr no.	Species_OTUs	16S reference sequence
57	Ligilactobacillus sp. OTU_86	GACGAACGCTGGCGGCGTGCCTAATACATGCAAGTCGAACGAAACTTTCTTACACCGAATGATTGCATTCACCGTAAGAAG<br>TTGAGTGGCGGACGGGTGAGTAACACGTGGGTAACCTGCCTAAAAGAAGGGGATAACACTTGGAAACAGGTGCTAATACCG<br>TATATCTCTAAGGATCGCATGATCTTTAGATGAAAGATGGTTCTGCTATCGCTTTTAGATGGACCCGCGGCGTATTAACTAGT<br>TGGTGGGGTAACGGCCTACCAAGGTGATGATACGTAGCCGAACTGAGAGGTTGATCGGCCACATTGGGACTGAGACACGG<br>CCCAAACTCCTACGGGAGGCAGCAGTAGGGAATCTTCCACAATGGACGCAAGTCTGATGGAGCAACGCCGCGTGAGTGAA<br>GAAGGTCTTCGGATCGTAAAACTCTGTTGTTAGAGAAGAACACGAGTGAGAGTAACTGTTCATTCGATGACGGTATCTAACC<br>AGCAAGTCACGGCTAACTACGTGCCAGCAGCCGCGGTAATACGTAGGTGGCAAGCGTTGTCCGGATTTATTGGGCGTAAA<br>GGGAACGCAGGCGGTCTTTTAAGTCTGATGTGAAAGCCTTCGGCTTAACCGGAGTAGTGCATTGGAAACTGGAAGACTTGA<br>GTGCAGAAGAGGAGAGTGGAACTCCATGTGTAGCGGTGAAATGCGTAGATATATGGAAGAACACCAGTGGCGAAAGCGGCT<br>CTCTGGTCTGTAACTGACGCTGAGGTTCGAAAGCGTGGGTAGCAAACAGGATTAGATACCCTGGTAGTCCACGCCGTAAAC<br>GATGAATGCTAGGTGTTGGAGGGTTTCCACCCTTCAGTGCCGCAGCTAACACAATAAGCATTCCGCCTGGGGAGTACGAC<br>CGCAAGGTTGAAACTCAAAGGAATTGACGGGGGCCCGCACAAGCGGTGGAGCATGTGGTTTAATTCGAAGCAACGCGAAG<br>AACCTTACCAGGTCTTGACATCCTTTGACCACCTAAGAGATTAGGCTTTCCCTTCGGGGACAAAGTGACAGGTGGTGCATG<br>GCTGTCGTCAGCTCGTGTCGTGAGATGTTGGGTTAAGTCCCGCAACGAGCGCAACCCTTGTTGTCAGTTGCCAGCATTAA<br>GTTGGGCACTCTGGCGAGACTGCCGGTGACAAACCGGAGGAAGGTGGGGACGACGTCAAGTCATCATGCCCCTTATGAC<br>CTGGGCTACACACGTGCTACAATGGACGGTACAACGAGTCGCGAGACCGCGAGGTTTAGCTAATCTCTTAAAGCCGTTCT<br>CAGTTCGGATTGTAGGCTGCAACTCGCCTACATGAAGTCGGAATCGCTAGTAATCGCGAATCAGCATGTCGCGGTGAATAC<br>GTTCCCGGGCCTTGTACACACCGCCCGTCACACCATGAGAGTTTGTAACACCCAAAGCCGGTGGGGTAACTGCAAGGAG<br>CCAGCCGTCTAAGGTGGGACAGATGATTGGGGTG
58	Porphyromonas sp. OTU_88	GATGAACGCTAGCGATAGGCTTAACACATGCAAGTCGAGGGGCAGCGAGATGTAGCAATACATTGTCGGCGACCGGCGAA<br>TGGGTGCGTAACACGTATGCAACTTACCTTTTAGAGGCGAATAACCCGATGAAAGTCGGACTAATACGCCATATGCTCCCTT<br>AGTCACATGATTAATGGAGGAAAGATTCATCGCTAAGAGATAGGCCTGCGTTCCATTAGCTAGTTGGAGAGGTAACGGCTCA<br>CCAAGGCAACGATGGATAGGGGGACTGAGAGGTTGACCCCCCACATTGACACTGAGATACGGGTCAAACTCCTACGGGAG<br>GCAGCAGTGAGGAATATTGGTCAATGGGCGAAAGCCTGAACCAGCCAAGTCGCGTGAAGGAAGACTGCCCGCAAGGGTTG<br>TAAACTTCTTTTGTATGGGATTAAAGGTGTCTACGAGTAGACAGTTGCAGTTACCATACGAATAAGCATCGGCTAACTCCGTG<br>CCAGCAGCCGCGGTAATACGGAGGATGCGAGCGTTATCCGGAATTATTGGGTTTAAAGGGTGCGTAGGTTGCAAGGGAAG<br>TCAGGGGTGAAAAGCCATAGCTTAACTAGGGTCTTGCCTTTGAAACTCTCTAGCTAGAGTGTAGTGGAGGTACGTGGAACG<br>TGTGGTGTAGCGGTGAAATGCATAGATATCACACAGAACTCCGATTGCGCAGGCAGCGTACTATACTACAACTGACACTGAA<br>GCACGAAAGCGTGGGTATCAAACAGGATTAGATACCCTGGTAGTCCACGCAGTAAACGATGAATACTAGATCTATGCGATAT<br>ACAGTATGGGTCTAAGCGAAAGCGATAAGTATTCCACCTGGGGAGTACGCCGGCAACGGTGAAACTCAAAGAGATTGGCGG<br>GGGTCCGCACAAGCGGAGGAACATGTGGTTTAATTCGATGATACGCGAGGAACCTTACCCGGGATTGAAATGTAGATGCAT<br>GAGGCAGAGATGCTTCTTCCCTTCGGGGCTTCTATGTAGGTGCTGCATGGTTGTCGTCAGCTCGTGCCGTGAGGTGTCG<br>GCTTAAGTGCCATAACGAGCGCAACCCGCGTCGATAGTTACTAACGAGTAAAGTCGAGGACTCTATCGAGACAGCCGTCGT<br>AAGACGAGAGGAAGGGGCGGATGACGTCAAATCAGCACGGCCCTTACATCCGGGGCGACACACGTGTTACAATGGTAGGG<br>ACAGCGAGCAGCCATCTGGTGACAGAGAGCTAATCTATAAACCCTATCCCAGTTCGGATCGGAGTCTGCAACTCGACTCTG<br>TGAAGCTGGATTCGCTAGTAATCGCGCATCAGCCATGGCGCGGTGAATACGTTCCCGGACCTTGCACACACCGCCCGTC<br>AAGCCATGGGAGTCGGGGGTACCTGAAGGGCGTGACCGTCACAGGAGCGCTTGAGGGTAAAACTGGTGACTGGGGCT
59	Selenomonas sp. OTU_89	GACGAACGCTGGCGGCGTGCTTAACACATGCAAGTCGAACGGGAACCTTTATTTCGGTAAGGGTGAGAGTGGCAAACGGG<br>TGAGTAACACGTAGGCAACCTGCCGACAGGATGGGGACAACATTCCGAAAGGAATGCTAATACCGAATGGAGTCCGGGGAT<br>GGCATCATCCCTGGATAAAAGATGGCCTCTGAATATGCTATCGCCTGTCGATGGGCCTGCGTCTGATTAGCCAGTTGGCG<br>GGGTAACGGCCCACCAAAGCGACGATCAGTAGCCGGTCTGAGAGGATGAACGGCCACATTGGGACTGAGACACGGCCCA<br>GACTCCTACGGGAGGCAGCAGTGGGGAATCTTCCACAATGGGCGAAAGCCTGATGGAGCAACGCCGCGTGAGTGAAGAA<br>GGTCTTCGGATCGTAAAGCTCTGTTGCACGGGACGAAAACCGGGTTTGAGAATATTGAACCCGGGTGACGGTACCGAGCG<br>AGGAAGCCACGGCTAACTACGTGCCAGCAGCCGCGGTAATACGTAGGTGGCGAGCGTTGTCCGGAATTATTGGGCGTAAA<br>GGGAGCGCAGGCGGACATATAAGTCCATCTTAAAAGTGCGGGGCTCAACCCCGTGAGGGGATGGAAACTGTATGCCTTGA<br>GTGCAGGAGAGGAAAGCGGAATTCCCAGTGTAGCGGTGAAATGCGTAGATATTGGGAGGAACACCAGTGGCGAAGGCGGC<br>TTTCTGGACTGCAACTGACGCTGAGGCTCGAAAGCCAGGGGAGCGAACGGGATTAGATACCCCGGTAGTCCTGGCCGTAA<br>ACGATGAATGCTAGGTGTAGGAGGTATCGACCCCTCCTGTGCCGGAGTTAACGCAATAAGCATTCCGCCTGGGGAGTACG<br>GTCGCAAGACTGAAACTCAAAGGAATTGACGGGGGCCCGCACAAGCGGTGGAGTATGTGGTTTAATTCGACGCAACGCGA<br>AGAACCTTACCAGGGCTTGACATTGAGTGAAAGAGCTAGAGATAGCTTCCTCCCTTCGGGGACACGAAAACAGGTGGTGCA<br>TGGCTGTCGTCAGCTCGTGTCGTGAGATGTTGGGTTAAGTCCCGCAACGAGCGCAACCCCTGTCCTTTGTTGCCAGCGC<br>GTAATGGCGGGAACTCAAAGGAGACTGCCGCGGAGAACGCGGAGGAAGGCGGGGATGACGTCAAGTCATCATGCCCCTT<br>ATGTCCTGGGCTACACACGTACTACAATGGGATGGACAGAGGGAAGCGAAGGCGCGAGCCGGAGCGGACCCCACAAACC<br>ATCCCCCAGTTCGGATTGCAGGCTGCAACCCGCCTGCATGAAGTCGGAATCGCTAGTAATCGCAGGTCAGCATACTGCGG<br>TGAATACGTTCCCGGGCCTTGTACACACCGCCCGTCACACCACGGAAGTCATTCACGCCCGAAGCCGGCGGGCCAACC<br>GCAAGGAGGCAGCCGTCTAAGGCGGGGGCGATGACTGGGGTG
60	Porphyromonadaceae gen. sp. OTU_95	GATGAACGCTAGCGATAGGCTTAACACATGCAAGTCGAGGGGTAACATGAGAAGAGCTTGCTCTTTTTGATGACGACCGGC<br>GCACGGGTGCGTAACACGTATGCAACCTGCCCTTTATTAGCGGATACCCCGATGAAAGTCGGACTAATACGTTATGCATTG<br>CGAAGTTTGCATGAACTTTGCAATAAAGATTTATCGATAAAGGATGGGCATGCGGTCCATTAGATAGTTGGTAGGGTAAAAGC<br>CTACCAAGTCAATGATGGATAGGGGGACTGAGAGGTTGACCCCCCACACCGGTACTGAGATACGGACCGGACTCCTACGG<br>GAGGCAGCAGTGAGGAATATTGGTCAATGGGCGAGAGCCTGAACCAGCCACGTCGCGTGAAGGAAGACTGACCTTACGGT<br>TTGTAAACTTCTTTTGTAGAGGGGTAATGGCAGATACGTGTATTTGCGTGAAAGTACTCTACGAATAAGCATCGGCTAACTCC<br>GTGCCAGCAGCCGCGGTAATACGGGGGATGCGAGCGTTATCCGGAATTATTGGGTTTAAAGGGTGCGCAGGTGGCTAATC<br>AGGTCAGTGGTGAAAAGCTATGGCTCAACCATGGTCTTGCCATTGAAACCGTATAGCTAGAGAGGAGACGAGGTAGGCGGA<br>ATGTGTGGTGTAGCGGTGAAATGCATAGATATCACACAGAACTCCGATTGCGAAGGCAGCTTACCAGGCTTTATCTGACACT<br>TAGGCACGACAGCGTGGGGATCAAACAGGATTAGATACCCTGGTAGTCCACGCCGTAAACGATGAATACTAGTTTTTTGCG<br>ATAAACAGTAAGAGACTAAGCGAAAGCGATAAGTATTCCACCTGGGGAGTACGCCGGCAACGGTGAAACTCAAAGGAATTGA<br>CGGGGGCCCGCACAAGCGGAGGAACATGTGGTTTAATTCGATGATACGCGAGGAACCTTACCCGGGATTGAAATTTAGGT<br>GCATGGCGAAGAGATTTGCCTTTCCTTCGGGACTCCTAAGTAGGTGCTGCATGGTTGTCGTCAGCTCGTGCCGTGAGGTG<br>TTGGCTTAAGTGCCATAACGAGCGCAACCCACGTCGATAGTTACTAACAGGTAAAGCTGAGGACTCTATTGATACTGCCGT<br>CGTAAGACGTGAGGAAGGTGTGGATGACGTCAAATCAGCACGGCCCTTATATCCGGGGCGACACACGTGTTACAATGGAG<br>AGGACAGCGAGAAGCGACTATGTGAGTAGGAGCGGATCTCTTAAACCTTTCCCCAGTTCGGATCGGAGTCTGCAACTCGA<br>CTCTGTGAAGCCGGATTCGCTAGTAATCGCGCATCAGCCATGGCGCGGTGAATACGTTCCCGGGCCTTGTACACACCGC<br>CCGTCAAGCCATGGGAATCGGGGGTACCTGAAGTGCGTGACCGTCAAAGGAGCGTTTTAGGGTAATACTGGTGACTGGGG<br>CT

Sr no.	Species_OTUs	16S reference sequence
61	Selenomonas sp. OTU_97	GACGAACGCTGGCGGCGTGCTTAACACATGCAAGTCGAACGGGACTAGAAAAGCTTGCTTTGCTAGTCTAGTGGCAAACG<br>GGTGAGTAACACGTAGACAACCTGCCGATAGGATGGGGACAACATTCCGAAAGGAATGCTAATACCGAATGAAGTGTAGGG<br>AAGGCATCTTCCCTACATGAAAGATGGCCTCTATATATAAGCTATCACCTATTGATGGGTCTGCGTCTGATTAGCTAGTAGG<br>TAAGGTAACGGCTTACCTAGGCGACGATCAGTAGCCGGTCTGAGAGGATGAACGGCCACATTGGGACTGAGACACGGCCC<br>AGACTCCTACGGGAGGCAGCAGTGGGGAATCTTCCGCAATGGGCGCAAGCCTGACGGAGCAACGCCGCGTGAGTGAAGA<br>AGGTCTTCGGATCGTAAAGCTCTGTTGAAGGGGACGAACGTACATAGTGCGAATAGTGCTGTGTAATGACGGTACCTTTTGA<br>GGAAGCCACGGCTAACTACGTGCCAGCAGCCGCGGTAATACGTAGGTGGCGAGCGTTGTCCGGAATCATTGGGCGTAAA<br>GGGAGCGCAGGCGGTCATGTAAGTCTTGCTTAAAAGTTCGGGGCTCAACCCCGTGATGGGCAAGAAACTATGTGACTTGA<br>GTGCAGGAGAGGAAAGCGGAATTCCCAGTGTAGCGGTGAAATGCGTAGATATTGGGAGGAACACCAGTGGCGAAGGCGGC<br>TTTCTGGACTGCAACTGACGCTGAGGCTCGAAAGCCAGGGGAGCGAACGGGATTAGATACCCCGGTAGTCCTGGCCGTAA<br>ACGATGGATACTAGGTGTGGGAGGTATCGACCCCTACCGTGCCGGAGTTAACGCAATAAGTATCCCGCCTGGGGAGTACG<br>GTCGCAAGACTGAAACTCAAAGGAATTGACGGGGACCCGCACAAGCGGTGGAGTATGTGGTTTAATTCGAAGCAACGCGAA<br>GAACCTTACCAGGTCTTGACATTGATTGAAAGGCGTAGAGATACGTCCCTCTCTTCGGAGACAAGAAAACAGGTGGTGCAT<br>GGCTGTCGTCAGCTCGTGTCGTGAGATGTTGGGTTAAGTCCCGCAACGAGCGCAACCCCTGTCCTTTGTTGCCAGCATGT<br>AGTGATGGGCACTCAAAGGAGACTGCCGCGGAGAACGCGGAGGAAGGCGGGGATGACGTCAAGTCATCATGCCCCTTAT<br>GACCTGGGCTACACACGTACTACAATGGGACGGACAGAGAGCAGCGAGAGGGCGACCTTAAGCGAACCTCAAAAACCGTT<br>TCTCAGTTCGGATTGCAGGCTGCAACTCGCCTGCATGAAGTCGGAATCGCTAGTAATCGCAGGTCAGCATACTGCGGTGA<br>ATACGTTCCCGGGTCTTGTACACACCGCCCGTCACACCACGGAAGTCGTTCACACCCGAAGCCGGCGCAGCCGTCTAAG<br>GTGGGGAAGGTGACTGGGGTG
62	Selenomonas sp. OTU_99	GACGAACGCTGGCGGCGTGCTTAACACATGCAAGTCGAACGGAGCGAATGAAAGCTTGCTTTTATGAGCTTAGTGGCAAAC<br>GGGTGAGTAACACGTAGACAACCTGCCGACAGGATGGGGACAACATTCCGAAAGGAATGCTAATACCGAATGAAGTCGAGG<br>GAAGGCATCTTCCTTCGATGAAAGATGGCCTCTGAATATGCTATCACCTGTCGATGGGTCTGCGTCTGATTAGCTGGTAGG<br>TGAGGTAACGGCTCACCTAGGCGACGATCAGTAGCCGGTCTGAGAGGATGAACGGCCACATTGGGACTGAGACACGGCC<br>CAGACTCCTACGGGAGGCAGCAGTGGGGAATCTTCCGCAATGGGCGCAAGCCTGACGGAGCAACGCCGCGTGAGTGAAG<br>AAGGTCTTCGGATCGTAAAGCTCTGTTGACGGGGACGAACGTGCGTGATGCGAACAGTGTCATGCAATGACGGTACCTGT<br>CGAGGAAGCCACGGCTAACTACGTGCCAGCAGCCGCGGTAATACGTAGGTGGCGAGCGTTGTCCGGAATCATTGGGCGT<br>AAAGGGAGCGCAGGCGGGCAAGTAAGTCTTTCTTAAAAGTGCGGGGCTCAACCCCGTGATGGGAAAGAAACTACATGTCTT<br>GAGTACAGGAGAGGAAAGCGGAATTCCCAGTGTAGCGGTGAAATGCGTAGATATTGGGAGGAACACCAGTGGCGAAGGCG<br>GCTTTCTGGACTGCAACTGACGCTGAGGCTCGAAAGCCAGGGGAGCGAACGGGATTAGATACCCCGGTAGTCCTGGCCG<br>TAAACGATGGATACTAGGTGTGGGAGGTATCGACCCCTACCGTGCCGGAGTTAACGCAATAAGTATCCCGCCTGGGGAGT<br>ACGGCCGCAAGGCTGAAACTCAAAGGAATTGACGGGGACCCGCACAAGCGGTGGAGTATGTGGTTTAATTCGAAGCAACG<br>CGAAGAACCTTACCAGGCCTTGACATTGACTGAAAGCGCTAGAGATAGCTCCCTCTCTTCGGAGACAGGAAAACAGGTGGT<br>GCATGGCTGTCGTCAGCTCGTGTCGTGAGATGTTGGGTTAAGTCCCGCAACGAGCGCAACCCCTGTTCTTTGTTGCCATC<br>AGGTAAAGCTGGGCACTCAAAGGAGACTGCCGCGGAGAACGCGGAGGAAGGCGGGGATGACGTCAAGTCATCATGCCCC<br>TTATGGTCTGGGCTACACACGTACTACAATGGAACGGACAGAGAGCAGCGAACCCGCGAGGGCAAGCGAACCTCAAAAAC<br>CGTTTCCCAGTTCGGATTGCAGGCTGCAACCCGCCTGCATGAAGTCGGAATCGCTAGTAATCGCAGGTCAGCATACTGCG<br>GTGAATACGTTCCCGGGTCTTGTACACACCGCCCGTCACACCACGGAAGTCATTCACACCCGAAGCCGGCGCAGCCGTC<br>TAAGGTGGGGAAGGTGACTGGGGTG
63	Prevotella sp. OTU_102	GATGAACGCTAGCTACAGGCTTAACACATGCAAGTCGAGGGGAAACGGGGAAGCAGCTTGCTGCTTTTTGTCGTCGACCG<br>GCGCACGGGTGAGTAACGCGTATCCAACCTTCCCATAACAAAGGGATACTCCGTAGAAATGCGGTCTAATACCTTATGTTC<br>TCTTTATTGGGCATCTTATTTAGAGCAAAGATTTATCGGTTATGGATGGGGATGCGTCTGATTAGCTTGTTGGCGGGGTAAC<br>GGCCCACCAAGGCATCGATCAGTAGGGGTTCTGAGAGGAAGGTCCCCCACATTGGAACTGAGACACGGTCCAAACTCCTA<br>CGGGAGGCAGCAGTGAGGAATATTGGTCAATGGGCGGAAGCCTGAACCAGCCAAGTAGCGTGCAGGAAGACGGCCCTAT<br>GGGTTGTAAACTGCTTTTACAGGGGAATAAAAAGGAGCACGTGTGCTCTGTTGCATGTACCCTGCGAATAAGGACCGGCTA<br>ATTCCGTGCCAGCAGCCGCGGTAATACGGAAGGTCCTGGTGTTATCCGGATTTATTGGGTTTAAAGGGAGCGTAGGCCGT<br>AGATTAAGTGTGTTGTGAAATGTAGTCGCCCAACGTCTGCCTTGCAGCGCAAACTGGTTTACTTGAGTACGCACAACGCAG<br>GCGGAATTCGTCGTGTAGCGGTGAAATGCTTAGATATGACGAAGAACTCCTATTGCGAAGGCAGCTTGCGGGAGCGTTACT<br>GACGCTGAAGCTCGAAAGTGCGGGTATCGAACAGGATTAGATACCCTGGTAGTCCGCACGGTAAACGATGGATGCCCGTT<br>TTTGGCTTATTTTAAGCCAGAGACCAAGCGAAAGCATTAAGCATCCCACCTGGGGAGTACGCCGGCAACGGTGAAACTCAA<br>AGGAATTGACGGGGGCCCGCACAAGCGGAGGAACATGTGGTTTAATTCGATGATACGCGAGGAACCTTACCCGGGCTTGA<br>ATTGCAGACGACATATTTAGAGATAGATATTCACTTCGGTGCGTCTGTGAAGGTGCTGCATGGTTGTCGTCAGCTCGTGCC<br>GTGAGGTGTCGGCTTAAGTGCCATAACGAGCGCAACCCCTGTCTTCAGTTGCCATCAGGTGATGCTGGGCACTCTGTAGA<br>TACTGCCGCCGTAAGGTGTGAGGAAGGTGGGGATGACGTCAAATCAGCACGGCCCTTACGTCCGGGGCTACACACGTGT<br>TACAATGGGGGGCACAGCGAGCGGGATGCCCGCAAGGTCATTCAAATCAATAAATCCCCCCTCAGTTCGGACTGGGGTCT<br>GCAACCCGACCCCACGAAGCTGGATTCGCTAGTAATCGCGCATCAGCCATGGCGCGGTGAATACGTTCCCGGGCCTTGT<br>ACACACCGCCCGTCAAGCCATGAAAGCCGGGGGTGCCTGAAGTCCGTGACCGTAAGGATCGGCCTAGGGCAAAACTGGT<br>AATTGGGGCT
64	Fusobacterium sp. OTU_103	GATGAACGCTGACAGAATGCTTAACACATGCAAGTCTACTTGAACTTCGGTTTGGGTGGCGGACGGGTGAGTAACGCGTAA<br>AGAACTTGCCTCACAGTTAGGGACAACATTTGGAAACGAATGCTAATACCTGATATTATGATTTTAGGGCATCCTAAGATTAT<br>GAAAGCTATATGTGCTGTGAGAGAGCTTTGCGTCCCATTAGCTAGTTGGAGAGGTAACGGCTCACCAAGGCGATGATGGGT<br>AGCCGGCCTGAGAGGGTGAACGGCCACAAGGGGACTGAGACACGGCCCTTACTCCTACGGGAGGCAGCAGTGGGGAATA<br>TTGGACAATGGACCAAAAGTCTGATCCAGCAATTCTGTGTGCACGATGACGTTTTTCGGAATGTAAAGTGCTTTCAGTTGGG<br>AAGAAAAAAATGACGGTACCAACAGAAGAAGTGACGGCTAAATACGTGCCAGCAGCCGCGGTAATACGTATGTCACAAGCGT<br>TATCCGGATTTATTGGGCGTAAAGCGCGTCTAGGTGGTTATGTAAGTCTGATGTGAAAATGCAGGGCTCAACTCTGTATTGC<br>GTTGGAAACTGTATGACTAGAGTACTGGAGAGGTAAGCGGAACTACAAGTGTAGAGGTGAAATTCGTAGATATTTGTAGGAAT<br>GCCGATGGGGAAGCCAGCTTACTGGACAGATACTGACGCTAAAGCGCGAAAGCGTGGGTAGCAAACAGGATTAGATACCC<br>TGGTAGTCCACGCCGTAAACGATGATTACTAGGTGTTGGGGGTCGAACCTCAGCGCCCAAGCTAACGCGATAAGTAATCC<br>GCCTGGGGAGTACGTACGCAAGTATGAAACTCAAAGGAATTGACGGGGACCCGCACAAGCGGTGGAGCATGTGGTTTAATT<br>CGACGCAACGCGAGGAACCTTACCAGCGTTTGACATCTTAGGAATGAGACAGAGATGTTTCAGTGTCACTTCGGTGAAACC<br>TAAAGACAGGTGGTGCATGGCTGTCGTCAGCTCGTGTCGTGAGATGTTGGGTTAAGTCCCGCAACGAGCGCAACCCCTTT<br>CGTATGTTACCATCATTAAGTTGGGGACTCATGCGATACTGCCTGCGATGAGCAGGAGGAAGGTGGGGATGACGTCAAGTC<br>ATCATGCCCCTTATACGCTGGGCTACACACGTGCTACAATGGGTAGTACAGAGAGTCGCAAAGCCGCGAGGTGGAGCTAA<br>TCTCAGAAAACTATTCTTAGTTCGGATTGTACTCTGCAACTCGAGTACATGAAGTTGGAATCGCTAGTAATCGCGAATCAGCA<br>ATGTCGCGGTGAATACGTTCTCGGGTCTTGTACACACCGCCCGTCACACCACGAGAGTTGGTTGCACCTGAAGTAGCAGG<br>CCTAACCGTAAGGAGGGATGCTCCGAGGGTGTGATTAGCGATTGGGGTG

Sr no.	Species_OTUs	16S reference sequence
65	Treponema sp. OTU_105	AACGAACGCTGGCGGCGCGTCTTAAGCATGCAAGTCGAACGGCAAGATACTTTCGGGTATCCTAGAGTGGCGGACTGGTG<br>AGTAACGCGTGGATGACGTACCCTCGTGACGGGGATAGCCGGTAGAAATACCGGATAATACCGGATAAGGTCTCACGGAAT<br>AAAGGTGTGAGAGGAAAGCAGCGTATGCTGCGCAGGAGGAACGGTCTGCGACCTATCAGCTGGTTGGCAGGGTAAAGGCC<br>TACCAAGGCGACGACGGGTATCCGGCCTGAGAGGGTGGACGGACACATTGGGACTGAGATACGGCCCAGACTCCTACGG<br>GAGGCAGCAGCTAAGAATATTCCGCAATGGGGGAAACCCTGACGGAGCGACGCCGCGTGGACGATGAAGGTCGGAAGATT<br>GTAAAGTCCTTTTATACATGAAGAAGAAGTTATACAGGAAATGGTATGATGGTGACTGTAGTGTATGAATAAGCACCGGCTAAT<br>TACGTGCCAGCAGCCGCGGTAACACGTAAGGTGCGAGCGTTGTTCGGAATTATTGGGCGTAAAGGGCATGCAGGCGGTC<br>GTGCAAGCCCGGCGTGAAAGGCGGGGGCTCAACTCCCGAACTGCGCTGGGAACTGCAAGGCTTGAGTTTCTGAAGGGTA<br>GTCGGAATTCCACGTGTAGGGGTGAAATCTGTAGATATGTGGAAGAACACCAATGGCGAAGGCAGGCTACCGGCAGAAGAC<br>TGACGCTGAGGTGCGAAGGTGCGGGGAGCAAACAGGATTAGATACCCTGGTAGTCCGCACAGTCAACGATGTGCACTAGA<br>TGTCTGTCCACGAGGATGGGTGTCAAAGCAAACGCGATAAGTGCACCGCCTGGGGAGTATGCCCGCAAGGGTGAAACTCA<br>AAGGAATTGACGGGGGCCCGCACAAGCGGTGGAGCATGTGGTTTAATTCGATGGTACGCGAGGAACCTTACCTGGGTTTG<br>ACATACACAGGGATTATGTAGAGATATGTAAGCGCAGCAATGCGCCTGTGAACAGGTGCTGCATGGCTGTCGTCAGCTCGT<br>GCCGTGAGGTGTTGGGTTAAGTCCCGCAACGAGCGCAACCCCTACTGCCAGTTACCAGCGCGTTATGGCGGGGACTCTG<br>GCGGAACTGCCTGCGACAAGCAGGAGGAAGGTGGGGATGACGTCAAGTCATCATGGCCCTTATGTCCAGGGCTACACAC<br>GTGCTACAATGGTTGGGACAGAGCGAAGCGAGTGCGAGAGTACAAGCAGAACGCAGAAATCCGATCGTAGTCCGGATTGG<br>AGTCTGAAACTCGACTCCATGAAGTTGGAATCGCTAGTAATCGCACATCAGCACGGTGCGGTGAATACGTTCCCGGGCCTT<br>GTACACACCGCCCGTCACACCATCCGAGTGGAGGGTACCCGAAGCCGGCAGTCTAACCGCAAGGGGGACGCTGTCGAA<br>GGTATGCTTCGTGAGGGGGGTG
66	Sphaerochaeta sp. OTU_106	AACGAACGCTGGCGGCGCGTTTTAAGCATGCAAGTCGAGCGGCAGCAGCAATGCGAGAGCGGCGGACGGGTGAGTAACA<br>CGTGGGTAATCTGCCCCCCTGTCGGGGATAGTCTGTGGAAACACAGGGTAATACCGGATGAGATATTTCTAACAAAAGATA<br>GGGATAGGAAAGGCGCCAAGGCGCCGCAGAGGGATGAGCCCGCGTCCCATTAGCTTGATGGTGAGGTAAAGGCCCACCA<br>TGGCGATGATGGGTAGCCGGCCTGAGAGGGTGAACGGCCACATTGGAACTGAGACACGGTCCAGACTCCTACGGGAGGC<br>AGCAGCTAAGAATCTTCCACAATGGACGAAAGTCTGATGGAGCGACGCCGCGTGAATGATGAAGGCCGTGAGGTTGTAAAG<br>TTCTTTTCGGGAGGAGGAATGAAGTTATCAGGGAATGGATGACAGATGACGAGAATCCCGGAATAAGCCCCGGCTAACTAC<br>GTGCCAGCAGCCGCGGTAACACGTAGGGGGCGAGCGTTGTTCGGAATCATTGGGCGTAAAGGGTGCGCAGGCGGCTTAG<br>CAAGTCTGGTGTGAAATGGTTGTGCTCAACACAATCAGGCGCCGGAAACTGAGAAGCTAGAGTCAACGAGGGGATGCCGG<br>AATTCCAGGTGTAGGGGTGAAATCTGTAGATATCTGGAAGAACACCAATGGCGAAGGCAGGCATCTGGCGATTGACTGACG<br>CTGAGGCACGAAGGTGCGGGGAGCAAACAGGTTTAGATACCCTGGTAGTCCGCACAGTAAACGATGTGCACCAGGTGTCG<br>GGCAGCAGTGTTCGGTACCGAAGCAAACGCAATAAGTGCACCGCCTGGGGAGTATGCCCGCAAGGGTGAAACTCAAAGGA<br>ATTGACGGGGGCCCGCACAAGCGGTGGAGCATGTGGTTTAATTCGACGATACGCGAGAAACCTTACCAGGGCTTGACATA<br>CACCGGAAGACACTTGAGAGAGTGTTGCTGCTTGCAGTCGGTGAACAGGTGCTGCATGGCTGTCGTCAGCTCGTGCTGTG<br>AAGTGTTGGGTTAAGTCCCGCAACGAGCGCAACCCCTGCCGTCTGTTACCAGCATGTAAAGATGGGGACTCAGATGGAAC<br>CGCCGGTGACAAACCGGAGGAAGGTGGGGACGACGTCAAGTCATCATGGCCCTTACGTCCTGGGCTACACACGTGCTAC<br>AATGGCCGGTACAGAGTGCGGCGAACCCGAGAGGGCAAGCGAATCACGGAAAGCCGGTCCCAGTACGGATTGGAGTCTG<br>CAACCCGACTCCATGAAGTTGGAATCGCTAGTAATCCCACATCAGCATGGTGGGGTGAATACGTTCCCGGGCCTTGTACA<br>CACCGCCCGTCACACCATCCGAGTCGGGGGTACCCGAAGTCGCCAGCCCAACCGCAAGGGGGGCGGTGCCGAAGGTAT<br>GTCCGGTGAGGGGGGTG
67	Selenomonadaceae gen. sp. OTU_108	GACGAACGCTGGCGGCGTGCTTAACACATGCAAGTCGAACGAAATTAAAAAATTATCATTGAGCTCTCCGATGAGATGGGAG<br>CCAAGCGTCGTGACCGCGCAGCGGGCACCACGCGTTCAACAAGTGCAACACCAAGTCGAAGGGTTGAATGGTAATTTTTT<br>AATTTAGTGGCAAACGGGTGAGTAACACGTAGACAACCTGCCGACAGGATGGGGACAACATTCCGAAAGGAATGCTAATAC<br>CGAATGAAGTCGAGGGAAGGCATCTTCCTTCGATGAAAGATGGCCTCTGAACATGCTATCACCTGTTGATGGGTCTGCGTC<br>TGATTAGCTAGTTGGTGAGGTAATGGCTCACCAAGGCGACGATCAGTAGCCGGTCTGAGAGGATGAACGGCCACATTGGG<br>ACTGAGACACGGCCCAGACTCCTACGGGAGGCAGCAGTGGGGAATCTTCCGCAATGGGCGCAAGCCTGACGGAGCAACG<br>CCGCGTGAGTGAAGAAGGTCCTCGGATCGTAAAGCTCTGTTGATGGGGACGAACGTGCGAAGGGTGAATAATCCTTTGCAA<br>TGACGGTACCTATCGAGGAAGCCACGGCTAACTACGTGCCAGCAGCCGCGGTAATACGTAGGTGGCGAGCGTTGTCCGG<br>AATCATTGGGCGTAAAGGGAGCGCAGGCGGGCATGTAAGTCTTTCTTAAAAGTTCGGGGCTCAACCCCGTGATGGGAAAGA<br>AACTACATGTCTTGAGTACAGGAGAGGAAAGCGGAATTCCCAGTGTAGCGGTGAAATGCGTAGATATTGGGAGGAACACCA<br>GTGGCGAAGGCGGCTTTCTGGACTGCAACTGACGCTGAGGCTCGAAAGCCAGGGGAGCGAACGGGATTAGATACCCCGG<br>TAGTCCTGGCCGTAAACGATGGATACTAGGTGTGGGAGGTATCGACCCCTACCGTGCCGGAGTTAACGCAATAAGTATCC<br>CGCCTGGGGAGTACGGTCGCAAGACTGAAACTCAAAGGAATTGACGGGGACCCGCACAAGCGGTGGAGTATGTGGTTTAA<br>TTCGAAGCAACGCGAAGAACCTTACCAGGCCTTGACATTGACTGAAAGAGCTAGAGATAGCTCCCTCTCTTCGGAGACAGG<br>AAAACAGGTGGTGCATGGCTGTCGTCAGCTCGTGTCGTGAGATGTTGGGTTAAGTCCCGCAACGAGCGCAACCCCTGTTC<br>TTTGTTGCCATCACGTAAAGGTGGGCACTCAAAGGAGACTGCCGCGGAGAACGCGGAGGAAGGCGGGGATGACGTCAAGT<br>CATCATGCCCCTTATGGTCTGGGCTACACACGTACTACAATGGAACGGACAGAGAGCAGCGAACCCGCGAGGGCAAGCGA<br>ACCTCAAAAACCGTTTCCCAGTTCGGATTGCAGGCTGCAACTCGCCTGCATGAAGTCGGAATCGCTAGTAATCGCAGGTCA<br>GCATACTGCGGTGAATACGTTCCCGGGTCTTGTACACACCGCCCGTCACACCACGGAAGTCGTTCACACCCGAAGCCGG<br>CGCAGCCGTCTAAGGTGGGGAAGGTGACTGGGGTG
68	Bacteroidales gen. sp. OTU_111	GATGAACGCTAGCGACAGGCCTAACACATGCAAGTCGAGGGGCAGCACAAGGGTAGCAATACCCTGGTGGCGACCGGCG<br>CACGGGTGAGTAACGCGTATGCAACCTGCCTACCACAGGGGGATAGCCTCCCGAAAGGAGATTAATACCGCATAAAACA<br>GGGGCCCCGCATGGGGATATTTGTTAAAGCAATCGCGGTGGTAGATGGGCATGCGTTCCATTAGCTTGTTGGCGGGGTAA<br>CGGCCCACCAAGGCGTCGATGGATAGGGGAACTGAGAGGTTTATCCCCCACACTGGGACTGAGACACGGCCCAGACTCC<br>TACGGGAGGCAGCAGTGAGGAATATTGGTCAATGGGCGCAAGCCTGAACCAGCCAAGTCGCGTGGAGGACGACGGTTCTA<br>TGGATTGTAAACTCCTTTTGCAGGGGAGTAAACTGCGCCACGTGTGGCGCATTGCAAGTACCCTGCGAATAAGCATCGGCT<br>AACTCCGTGCCAGCAGCCGCGGTAATACGGAGGATGCGAGCGTTATCCGGATTTATTGGGTTTAAAGGGTGCGTAGGCGG<br>CTTGTTAAGTCAGCGGTAAAATTTCCGGGCTCAACCCGGTCGTGCCGTTGAAACTGGCAGGCTTGAGTATGGATGAAGTGG<br>GCGGAATTTGTAGTGTAGCGGTGAAATGCTTAGATATTACAAGGAACTCCGATTGCGCAGGCAGCTCACTAAACCATAACTG<br>ACGCTGAAGCACGAAAGCGTGGGTATCAAACAGGATTAGATACCCTGGTAGTCCACGCAGTAAACGATGATCACTCGCTGT<br>TTGCGATATACAGCAAGCGGCTTAGCGAAAGCGTTAAGTGATCCACCTGGGGAGTACGCCGGCAACGGTGAAACTCAAAG<br>GAATTGACGGGGGCCCGCACAAGCGGAGGAACATGTGGTTTAATTCGATGATACGCGAGGAACCTTACCCGGGCTTGAAA<br>TGCAGAAGAATGGATTGGAAACATTCCCGCCAGCAATGGCTTCTGTGTAGGTGCTGCATGGTTGTCGTCAGCTCGTGCCG<br>TGAGGTGTCGGCTTAAGTGCCATAACGAGCGCAACCCTCATCGGTAGTTACCATCAGGTCATGCTGGGGACTCTACCGAG<br>ACTGCCGCCGTAAGGTGCGAGGAAGGTGGGGATGACGTCAAATCAGCACGGCCCTTACGTCCGGGGCGACACACGTGTT<br>ACAATGGGGGGTACAAAGGGCAGCTACCTGGCGACAGGATGCTAATCTCCAAAACCCTTCTCAGTTCGGATCGGAGTCTG<br>CAACTCGACTCCGTGAAGCTGGATTCGCTAGTAATCGCGCATCAGCCATGGCGCGGTGAATACGTTCCCGGGCCTTGTAC<br>ACACCGCCCGTCAAGCCATGAAAGCCGGGGGTACCTGAAGTACGTAACCGCAAGGAGCGTCCTAGGGTAAAACTGGTGAT<br>TGGGGCT

Sr no.	Species_OTUs	16S reference sequence
69	Haemophilus sp. OTU_113	ATTGAACGCTGGCGGCAGGCTTAACACATGCAAGTCGAACGGTAGCAGGAGAAAGCTTGCTTTCTTGCTGACGAGTGGCG<br>AACGGGTGAGTAATGCTTGGGAATCTGGCTTATGGAGGGGGATAACTACGGGAAACTGTAGCTAATACCGCGTAGTGTCGA<br>GAGACGAAAGTGCGGGACCGCAAGGCCGCATGCCATGAGATGAGCCCAAGTGGGATTAGGTAGTTGGTGGGGTAAAGGC<br>CTACCAAGCCTGCGATCTCTAGCTGGTCTGAGAGGATGGCCAGCCACACTGGAACTGAGACACGGTCCAGACTCCTACG<br>GGAGGCAGCAGTGGGGAATATTGCGCAATGGGGGGAACCCTGACGCAGCCATGCCGCGTGAATGAAGAAGGCCTTCGGG<br>TTGTAAAGTTCTTTCGGTATTGAGGAAGGTTGATGTGTTAATAGTACATCAAATTGACGTTAAATACAGAAGAAGCACCGGCTA<br>ACTCCGTGCCAGCAGCCGCGGTAATACGGAGGGTGCGAGCGTTAATCGGAATAACTGGGCGTAAAGGGCACGCAGGCGG<br>TTATTTAAGTGAGGTGTGAAAGCCCCGGGCTTAACCTGGGAATTGCATTTCAGACTGGGTAACTAGAGTACTTTAGGGAGGG<br>GTAGAATTCCACGTGTAGCGGTGAAATGCGTAGAGATGTGGAGGAATACCGAAGGCGAAGGCAGCCCCTTGGGAATGTACT<br>GACGCTCATGTGCGAAAGCGTGGGGAGCAAACAGGATTAGATACCCTGGTAGTCCACGCTGTAAACGCTGTCGATTTGGG<br>GATTGGGCTTAGAGCTTGGTGCCCGTAGCTAACGTGATAAATCGACCGCCTGGGGAGTACGGCCGCAAGGTTAAAACTCA<br>AATGAATTGACGGGGGCCCGCACAAGCGGTGGAGCATGTGGTTTAATTCGATGCAACGCGAAGAACCTTACCTACTCTTGA<br>CATCCAGAGAACTTTCCAGAGATGGATTGGTGCCTTCGGGAACTCTGAGACAGGTGCTGCATGGCTGTCGTCAGCTCGTG<br>TTGTGAAATGTTGGGTTAAGTCCCGCAACGAGCGCAACCCTTATCCTTTGTTGCCAGCGATTTGGTCGGGAACTCAAAGGA<br>GACTGCCAGTGATAAACTGGAGGAAGGTGGGGATGACGTCAAGTCATCATGGCCCTTACGAGTAGGGCTACACACGTGCT<br>ACAATGGCGTATACAGAGGGTGGCGAAACTGCGAGGTGGAGCGAATCTCAGAAAGTACGTCTAAGTCCGGATTGGAGTCTG<br>CAACTCGACTCCATGAAGTCGGAATCGCTAGTAATCGCGAATCAGAATGTCGCGGTGAATACGTTCCCGGGCCTTGTACAC<br>ACCGCCCGTCACACCATGGGAGTGGGTTGTACCAGAAGTAGATAGCTTAACCTTTTGGAGGGCGTTTACCACGGTATGATT<br>CATGACTGGGGTG
70	Lachnospiraceae gen. sp. OTU_116	GATGAACGCTGGCGGCGTGCTTAACACATGCAAGTCGAACGGAGTTTGTATGGAATGAGTCTTCGGACAAATGAAATATAAA<br>CTTAGTGGCGGACGGGTGAGTAACGTGTGGGCAACCTACCCTATACAGGAGGATAACAGTAAGAAATTATTGCTAAAACTGC<br>ATAAGATTACGAGTTCGCATGAACAAGTGATAAAAACTTTAGTGGTATAGGATGGGCCCGCATCTGATTAGGTAGTTGGTAGA<br>GTAAAAGCCTACCAAGCCGACGATCAGTAGCCGATCTGAGAGGATGACCGGCCACATTGGGACTGAGACACGGCCCAAAC<br>TCCTACGGGAGGCAGCAGTGGGGAATATTGGACAATGGGGGAAACCCTGATCCAGCGACGTCGCGTGAGTGAAGAAGTAT<br>TTCGGTATGTAAAGCTCTATCAGCAGGGAAGAAAATGACGGTACCTGACTAAGAAGCCCCGGCTAACTACGTGCCAGCAGC<br>CGCGGTAATACGTAGGGGGCAAGCGTTATCCGGATTTACTGGGTGTAAAGGGAGCGTAGACGGCGATGTAAGTCTGAAGT<br>GAAAGCCCACGGCTCAACTGTGGGACTGCTTTGGAAACTATATAGCTAGAGTATCGGAGGGGCAAGCGGAATTCCTAGTGT<br>AGCGGTGAAATGCGTAGATATTAGGAGGAACACCGGAGGCGAAGGCGGCTTGCTGGACGAAGACTGACGTTGAGGCTCGA<br>AGGCGTGGGGAGCAAACAGGATTAGATACCCTGGTAGTCCACGCAGTAAACGATGAATACTAGATGTCGGAGAGGTTACTT<br>TTCGGTGTCGTCGCAAACGCAATAAGTATTCCACCTGGGGAGTACGTTCGCAAGAATGAAACTCAAAGGAATTGACGGGGA<br>CCCGCACAAGCGGTGGAGCATGTGGTTTAATTCGAAGCAACGCGAAGAACCTTACCAAGTCTTGACATACCCTTGAATAGT<br>GAGTAAAGTTACTAGCCCTTCGGGGCAGGGGATACAGGTGGTGCATGGTTGTCGTCAGCTCGTGTCGTGAGATGTTGGGT<br>TAAGTCCCGCAACGAGCGCAACCCTTGTTCTTAGTAGCCAGCAGTAAGATGGGAACTCTAAGAAGACTGCCATGGATAACA<br>TGGAGGAAGGTGGGGATGACGTCAAATCATCATGCCCCTTATGATTTGGGCTACACACGTGCTACAATGGCGTAAACAGAG<br>GGAGGCGAAGGAGAGATCTGGAGCAAATCCCAAAAATAACGTCTCAGTTCGGATTGTAGGCTGCAACTCGCCTACATGAAG<br>CTGGAATCGCTAGTAATCGCAAATCAGAATGTTGCGGTGAATACGTTCCCGGGTCTTGTACACACCGCCCGTCACACCATG<br>GGAGTCATCAATGCCCGAAGTCAGTGACCTAACCGAAAGGGAGGAGCTGCCGAAGGCAGGGAGGATAACTGGGGTG
71	Lachnoanaerobaculum sp. OTU_117	GATGAACGCTGGCGGCGTGCTTAACACATGCAAGTCGAACGAAGCTACCCAAAGGAAGTCTTCGGATGGAATTTAGGTAGA<br>CTTAGTGGCGGACGGGTGAGTAACGCGTGGATAACCTGCCTTATACAGGGGGATAACGGAGAGAAATTTCCGCTAACACC<br>GCATAAGACCACAGCACCGCATGGTGCAGGGGTAAAATATTTATAGGTATAAGATGGATCCGCGTCCGATTAGCTAGTTGGT<br>GAGGTAAAGGCCCACCAAGGCGACGATCGGTAGCCGGCCTGAGAGGGTGAACGGCCACATTGGGACTGAGACACGGCC<br>CAAACTCCTACGGGAGGCAGCAGTGGGGAATATTGGACAATGGGGGAAACCCTGATCCAGCGACGCCGCGTGAGTGAAGA<br>AGTATTTCGGTATGTAAAGCTCTATCAGCAGGGAAGAAAATGACGGTACCTGACTAAGAAGCCCCGGCTAACTACGTGCCA<br>GCAGCCGCGGTAATACGTAGGGGGCAAGCGTTATCCGGATTTACTGGGTGTAAAGGGAGCGCAGACGGCAATGCAAGTCT<br>GAAGTGAAAGGCGTGGGCTCAACCCATGAACTGCTTTGGAAACTGTATAGCTTGAGTGTCGGAGGGGTAAGCGGAATTCCT<br>AGTGTAGCGGTGAAATGCGTAGATATTAGGAGGAACACCGGAGGCGAAGGCGGCTTACTGGACGACAACTGACGTTGAGG<br>CTCGAAGGCGTGGGGAGCAAACAGGATTAGATACCCTGGTAGTCCACGCAGTAAACGATGAATACTTGGTGTCGGGGAGG<br>TAAACTCTTCGGTGCCGCAAGCTAACGCATTAAGTATTCCACCTGGGGAGTACGTTCGCAAGAATGAAACTCAAAGGAATTG<br>ACGGGGACCCGCACAAGCGGTGGAGCATGTGGTTTAATTCGAAGCAACGCGAAGAACCTTACCAAATCTTGACATACTCTT<br>GAATAGTTTTGTAATGAGACTAGTCCTTCGGGACAAGGGATACAGGTGGTGCATGGTTGTCGTCAGCTCGTGTCGTGAGAT<br>GTTGGGTTAAGTCCCGCAACGAGCGCAACCCTTGTCGTCAGTAGCCAGCAGTAAGATGGGCACTCTGACGAGACAGCCG<br>GAGATAATCCGGAGGAAGGTGGGGATGACGTCAAATCATCATGCCCCTTATGATTTGGGCTACACACGTGCTACAATGGCG<br>TAAACAAAGTGAAGCAAAGCCGCGAGGTCAAGCAAATCACAAAAATAACGTCTCAGTTCGGATTGTAGTCTGCAACTCGACT<br>ATATGAAGCTGGAATCGCTAGTAATCGCAGATCAGAATGCTGCGGTGAATACGTTCCCGGGTCTTGTACACACCGCCCGTC<br>ACACCATGGGAGTCATCAATGCCCGAAGTCAGTGACCTAACCCACACTGGTAAACATAAAGCTGGGATAAGAATACGAGGG<br>AATAAGCCGACAGGCTTATGAGTGAGTATTCTTATATGATATAAAGAATAGCAGTAGTTATGTTTAGCAGTGTGGGAAGGAGC<br>TGCCGAAGGCAGGGAGGATAACTGGGGTG
72	Lactobacillus sp. OTU_118	GACGAACGCTGGCGGCGTGCCTAATACATGCAAGTCGAGCGAGCTTGCCTAGATGATTTTAGTGCTTGCACTAAATGAAAC<br>TAGATACAAGCGAGCGGCGGACGGGTGAGTAACACGTGGGTAACCTGCCCAAGAGACTGGGATAACACCTGGAAACAGAT<br>GCTAATACCGGATAACAACACTAGACGCATGTCTAGAGTTTGAAAGATGGTTCTGCTATCACTCTTGGATGGACCTGCGGTG<br>CATTAGCTAGTTGGTAAGGTAACGGCTTACCAAGGCAATGATGCATAGCCGAGTTGAGAGACTGATCGGCCACATTGGGAC<br>TGAGACACGGCCCAAACTCCTACGGGAGGCAGCAGTAGGGAATCTTCCACAATGGACGAAAGTCTGATGGAGCAACGCCG<br>CGTGAGTGAAGAAGGGTTTCGGCTCGTAAAGCTCTGTTGGTAGTGAAGAAAGATAGAGGTAGTAACTGGCCTTTATTTGACG<br>GTAATTACTTAGAAAGTCACGGCTAACTACGTGCCAGCAGCCGCGGTAATACGTAGGTGGCAAGCGTTGTCCGGATTTATT<br>GGGCGTAAAGCGAGTGCAGGCGGTTCAATAAGTCTGATGTGAAAGCCTTCGGCTCAACCGGAGAATTGCATCAGAAACTGT<br>TGAACTTGAGTGCAGAAGAGGAGAGTGGAACTCCATGTGTAGCGGTGGAATGCGTAGATATATGGAAGAACACCAGTGGCG<br>AAGGCGGCTCTCTGGTCTGCAACTGACGCTGAGGCTCGAAAGCATGGGTAGCGAACAGGATTAGATACCCTGGTAGTCCA<br>TGCCGTAAACGATGAGTGCTAAGTGTTGGGAGGTTTCCGCCTCTCAGTGCTGCAGCTAACGCATTAAGCACTCCGCCTGG<br>GGAGTACGACCGCAAGGTTGAAACTCAAAGGAATTGACGGGGGCCCGCACAAGCGGTGGAGCATGTGGTTTAATTCGAAG<br>CAACGCGAAGAACCTTACCAGGTCTTGACATCCAGTGCAAACCTAAGAGATTAGGTGTTCCCTTCGGGGACGCTGAGACAG<br>GTGGTGCATGGCTGTCGTCAGCTCGTGTCGTGAGATGTTGGGTTAAGTCCCGCAACGAGCGCAACCCTTGTCATTAGTTG<br>CCATCATTAAGTTGGGCACTCTAATGAGACTGCCGGTGACAAACCGGAGGAAGGTGGGGATGACGTCAAGTCATCATGCC<br>CCTTATGACCTGGGCTACACACGTGCTACAATGGACGGTACAACGAGAAGCGAACCTGCGAAGGCAAGCGGATCTCTTAAA<br>GCCGTTCTCAGTTCGGACTGTAGGCTGCAACTCGCCTACACGAAGCTGGAATCGCTAGTAATCGCGGATCAGCACGCCG<br>CGGTGAATACGTTCCCGGGCCTTGTACACACCGCCCGTCACACCATGAGAGTCTGTAACACCCAAAGCCGGTGGGATAAC<br>CTTTATAGGAGTCAGCCGTCTAAGGTAGGACAGATGATTAGGGTG

Sr no.	Species_OTUs	16S reference sequence
73	Porphyromonadaceae gen. sp. OTU_119	GATGAACGCTAGCGACAGGCTTAACACATGCAAGTCGAGGGGCATCACGGGATAGCAATATTCTGGTGGCGACCGGCGCA<br>CGGGTGAGTAACGCGTATGCAACTTACCTGTCAGAGGGGGATAATCCGGCGAAAGTCGGTCTAATACCCCATAAAACAGGG<br>ACCCCACCTGGGGTTATTTGTTAAAGGATTTATCGCTGACAGATAGGCATGCGTTCCATTAGGTAGTTGGTCGGGGTAACG<br>GCCCACCAAGCCATCGATGGATAGGGGTTCTGAGAGGAAGGTCCCCCACACTGGTACTGAGACACGGACCAGACTCCTA<br>CGGGAGGCAGCAGTGAGGAATATTGGTCAATGGGCGAGAGCCTGAACCAGCCAAGTCGCGTGAAGGATGACTGCCCTATG<br>GGTTGTAAACTTCTTTTATAAGGGAATAAAGTGCGGGACGCGTCCCGTTTTGTATGTACCTTATGAATAAGCATCGGCTAACT<br>CCGTGCCAGCAGCCGCGGTAATACGGAGGATGCGAGCGTTATCCGGATTTATTGGGTTTAAAGGGTGCGTAGGTGGGTAA<br>TTAAGTCAGCGGTGAAAGTTTGTGGCTCAACCATAAAATTGCCGTTGAAACTGGTCACCTTGAGTATGTTTGCTGTAGGCGG<br>AATGCGTGTTGTAGCGGTGAAATGCATAGATATCACGCAGAACTCCGATTGCGAAGGCAGCTTACAAAACCATCACTGACAC<br>TGAAGCACGAAAGCGTGGGAATCAAACAGGATTAGATACCCTGGTAGTCCACGCCGTAAACGATGATTACTAGGGATTTGC<br>GATATAGTGTAAGTCCTACAGCGAAAGCGTTAAGTAATCCACCTGGGGAGTACGCCGGCAACGGTGAAACTCAAAGGAATT<br>GACGGGGGCCCGCACAAGCGGAGGAACATGTGGTTTAATTCGATGATACGCGAGGAACCTTACCCGGGTTTGAACGCATT<br>AGGAATACGATTGAAAGGTTGTAGCTAGCAATAGCCTATTGCGAGGTGCTGCATGGTTGTCGTCAGCTCGTGCCGTGAGGT<br>GTCGGCTTAAGTGCCATAACGAGCGCAACCCTGATTGTCAGTTGCTAGCAGGTAAAGCTGAGGACTCTGACAAGACTGCC<br>GGCGTAAGCTGTGAGGAAGGTTGGGATGACGTCAAATCAGCACGGCCCTTACATCCGGGGCGACACACGTGTTACAATGG<br>GGGGGACAAAGGGGCGCTACCTGGCGACAGGATGCCAATCTCAAAACCCTCTCTCAGTTCGGATCGGAGTCTGCAACTC<br>GACTCCGTGAAGCTGGATTCGCTAGTAATCGCGCATCAGCCATGGCGCGGTGAATACGTTCCCGGGCCTTGTACACACC<br>GCCCGTCAAGCCATGGGAGCCGGGGGTACCTGAAGTACGTAACCGTCAAAGGATCGTCCTAGGGTAAAACCGGTGACTG<br>GGGCT
74	Bacteroidales gen. sp. OTU_120	GATGAACGCTAGCTACAGGCTTAACACATGCAAGTAGAGGGGCATCATTGTATTGATTTCGGTCATTACAGATGGCGACCTG<br>CGGATGGGTGAGTAACACGTATCCAACCTGCCCGTCACCGGAGTATAGCCTCCCGAAAGGGAAATTAACACTCCGCGACA<br>TCATTTCGGGGCATCCCGTTATGATTAAAGTTTTACGGTGTCGGATGGGGATGCGTCCCATTAGATAGTAGGCGGGGTAAC<br>GGCCCACCTAGTCGTCGATGGGTAGGGGTTCTGAGAGGAAGGTCCCCCACATTGGAACTGAGACACGGTCCAAACTCCTA<br>CGGGAGGCAGCAGTGAGGAATATTGGTCAATGGGCGAGAGCCTGAACCAGCCAAGTAGCGTGCAGGAAGACGGCCCTAT<br>GGGTTGTAAACTGCTTTTATACGGGAATAAAGTTTGCCACGTGTGGCATTTTGCATGTACCGTAAGAAAAAGGATCGGCTAAT<br>TCCGTGCCAGCAGCCGCGGTAATACGGAAGATCCGAGCGTTATCCGGATTTATTGGGTTTAAAGGGAGCGTAGGCGGGAA<br>TCCAAGTCAGTTGTGAAACCCTGCGGCTCAACCGTAGTCGTGCAGTTGAAACTGGATTTCTTGAGTGCGCACAGGGCAGA<br>CGGAATTTGTGGTGTAGCGGTGAAATGCTTAGATATCACGAAGAACTCCGATCGCGAAGGCAGTCTGCCGGGGCGCTACT<br>GACGCTGAGGCTCGAAAGTGCGGGTATCAAACAGGATTAGATACCCTGGTAGTCCGCACTGTAAACGATGGATACCCGTG<br>GTCGGTTTTTTTATTCGGTCACCAAGCGAAAGCGTTAAGTATCCCACCTGGGGAGTACGCCGGCAACGGTGAAACTCAAA<br>GGAATTGACGGGGGCCCGCACAAGCGGAGGAACATGTGGTTTAATTCGATGATACGCGAGGAACCTTACCCGGGCTTGAA<br>TTGCAGAGGAAGTCACCGGAGACGGTGATGTCTTTATAGTCTCTGTGAAGGTGCTGCATGGTTGTCGTCAGCTCGTGCCG<br>TGAGGTGTCGGCTTAAGTGCCATAACGAGCGCAACCCCTTTTTCCGGTTGCCATCAGGTGATGCTGGGCACTCCGGAGAC<br>ACTGCCATCGTAAGATGTGAGGAAGGTGGGGATGACGTCAAATCAGCACGGCCCTTACGTCCGGGGCTACACACGTGTTA<br>CAATGGCCGGTACAGAAGGCAGCTCCCACGCGAGTGGTTGCGAATCCCGAAAGCCGGTCCCAGTTCGGACTGGAGTCTG<br>CAACCCGACTCCACGAAGCTGGATTCGCTAGTAATCGCGCATCAGCCACGGCGCGGTGAATACGTTCCCGGGCCTTGTA<br>CACACCGCCCGTCAAGCCATGAAAGCCGAAGGTACCTGAAGTCCGTGACCGCGAGGGTCGGCCGAGGGTAAATTTGGTG<br>ATTGGGGCT
75	Schaalia sp. OTU_122	GACGAACGCTGGCGGCGTGCTTAACACATGCAAGTCGAACGGACTGCTTGGGGGCTTGCTCCTGGGTGGTGAGTGGCGA<br>ACGGGTGAGTAACACGTGAGTAACCTGCCCTCTTCTTTGGGATAACGGCCGGAAACGGCTGCTAATACCGGGTAGTCACT<br>GGTCTCCGCATGGGGGTTGGTGGAAAGGGGCCCTTTTTGGGTTTCTGGTGGGGGATGGGCTCGCGGCTTATCAGCTTGT<br>TGGTGGGGTGATGGCTCACCAAGGCTTTGACGGGTAGCCGGCCTGAGAGGGTGACCGGTCACATTGGGACTGAGATACG<br>GCCCAGACTCCTACGGGAGGCAGCAGTGGGGAATTTTGCACAATGGGCGCAAGCCTGATGCAGCGACGCCGCGTGAGG<br>GATGGAGGCCTTCGGGTTGTGAACCTCTTTCGCTCATGGTCAAGCCGCGGATGTGTCCGTGGTGAGGGTAGTGGGTAAAG<br>AAGCGCCGGCTAACTACGTGCCAGCAGCCGCGGTAATACGTAGGGCGCGAGCGTTGTCCGGAATTATTGGGCGTAAAGG<br>GCTTGTAGGCGGTTTGTCGCGTCTGCCGTGAAATTCCCTGGCTTAACTGGGGGCGTGCGGTGGGTACGGGCAGGCTTGA<br>GTGCGGTAGGGGAGACTGGAATTCCTGGTGTAGCGGTGGAATGCGCAGATATCAGGAGGAACACCGGTGGCGAAGGCGG<br>GTCTCTGGGCCGTTACTGACGCTGAGGAGCGAAAGCATGGGGAGCGAACAGGATTAGATACCCTGGTAGTCCATGCTGTA<br>AACGTTGGGCACTAGGTGTGGGGGCCGTCCTTGGTTTCTGCGCCGTAGCTAACGCTTTAAGTGCCCCGCCTGGGGAGTA<br>CGGCCGCAAGGCTAAAACTCAAAGGAATTGACGGGGGCCCGCACAAGCGGCGGAGCATGCGGATTAATTCGATGCAACG<br>CGAAGAACCTTACCAAGGCTTGACATGCACCGCGACACTGCAGAGATGTGGTGGCCTTCGGGGTGGTGTGCAGGTGGTG<br>CATGGTTGTCGTCAGCTCGTGTCGTGAGATGTTGGGTTAAGTCCCGCAACGAGCGCAACCCTTGCCCTATGTTGCCAGCA<br>CGTGATGGTGGGGACTCGTGGGGGACTGCCGGGGTTAACTCGGAGGAAGGTGGGGATGACGTCAAATCATCATGCCCCT<br>TATGTCTTGGGCTTCACGCATGCTACAATGGTCGGTACAGAGGGTTGCGATGCCGTGAGGTGGAGCGAATCCCTTAAAGC<br>CGGTCTCAGTTCGGATTGGGGTCTGCAACTCGACCCCATGAAGGTGGAGTCGCTAGTAATCGCAGATCAGCAACGCTGCG<br>GTGAATACGTTCTCGGGCCTTGTACACACCGCCCGTCACGTCACGAAAGTTGGTAACACCCGAAGCCCACGGCCCAACC<br>GGCTTGTCCGGGGGGAGTGGTCGAAGGTGGGATTGGCGATTGGGACG
76	Catonella sp. OTU_126	GATGAACGCTGGCGGCGTGCCTAACACATGCAAGTCGAACGGAGATTACGGACGGAAGTTTTCGGATGGAAGGCTGTAATT<br>CTTAGTGGCGGACGGGTGAGTAACGCGTGGGCAACCTGCCCTGTACAGGGGGATAGCAGCTGGAAACGGCTGGTAAGAC<br>CGCATAAGTCGGCGGGACCGCATGGTTCTGTCGGGAAATGAGCAATCAGGTACAGGATGGGCCCGCGTCCGATTAGCCA<br>GTTGGCAGGGTAAGAGCCTACCAAAGCGACGATCGGTAGCCGGACTGAGAGGTCGGACGGCCACATTGGGACTGAGACA<br>CGGCCCAAACTCCTACGGGAGGCAGCAGTGGGGGATATTGCACAATGGGGGAAACCCTGATGCAGCGACGCCGCGTGAG<br>TGAAGAAGTATTTCGGTATGTAAAGCTCTATCAGCAGGGAAGATGATGACGGTACCTGACTAAGAAGCCCCGGCTAACTACG<br>TGCCAGCAGCCGCGGTAATACGTAGGGGGCAAGCGTTATCCGGATTTACTGGGTGTAAAGGGAGCGCAGGCGGTTTTGCA<br>AGTTGAGAGTGGAAGCAGGGGGCTCAACCCCCTGACTGCTCCCAAAACTGTAGGACTTGAGTATGGGAGAGGCAGGCGGA<br>ATTCCTAGTGTAGCGGTGAAATGCTTAGATATTAGGAAGAACACCGGTGGCGAAGGCGGCCTGCTGGACCAAAACTGACGC<br>TGAGGCTCGAAAGCGTGGGTAGCAAACAGGATTAGATACCCTGGTAGTCCACGCTGTAAACGATGAATACCAGGTGCCGG<br>GGGGCATAAGCCTTTCGGTGCCGTCGCAAACGCATTAAGTATTCCACCTGGGGAGTACGTTCGCAAGAATGAAACTCAAAG<br>GAATTGACGGGGACCCGCACAAGCGGTGGAGCATGTGGTTTAATTCGACGCAACGCGAAGAACCTTACCCGGTCTTGAGA<br>TCCTCCTGAATATGGGGTAATGTCCATAGTCCTTCGGGACAGGAGAGACAGGTGGTGCATGGTTGTCGTCAGCTCGTGTC<br>GTGAGATGTTGGGTTAAGTCCCGCAACGAGCGCAACCCTTATTCTTAGTAGCCAGCGGTTTGGCCGGGCACTCTAGGGAG<br>ACTGCCGGGGATAACCCGGAGGAAGGCGGGGATGACGTCAAATCATCATGCCCCTTATGACCGGGGCTACACACGTGCT<br>ACAATGGCGGCTACAGAGGGATGCGAAGGCGTGAGCCGGAGCGAATCCCATAAAGGCCGCCTCAGTTCGGATTGCAGTC<br>TGCAACTCGACTGCATGAAGCTGGAATCGCTAGTAATCGCAAATCAGAATGTTGCGGTGAATACGTTCCCGGGTCTTGTAC<br>ACACCGCCCGTCACACCATGGGAGTCGGAAACGCCCGAAGTCAGTGGCCTAACCGTAAGGGAGGAGCTGCCGAAGGCG<br>GGTCTGGTGACTGGGGTG

Sr no.	Species_OTUs	16S reference sequence
77	Erysipelotrichaceae gen. sp. OTU_127	GATGAACGCTGGCGGCGTGCCTAATACATGCAAGTCGAACGAAGAAGTAAGCTTGCTTACTTCTTAGTGGCGAACGGGTGA<br>GTAATACATAAGCAATCTGCCCATATGCCTGGGATAACCGTTGGAAACGATGGCTAATACCGGATAGGTGAATTGAAGGCAT<br>CTTTGATTCATTAAAGTTGGGATACAACACATATGGATGAGCTTATGGCGCATTAGCTAGTTGGTGAGGTAACGGCTCACCA<br>AGGCGATGATGCGTAGCCGACCTGAGAGGGTGACCGGCCACACTGGGACTGAGACACGGCCCAGACTCCTACGGGAGG<br>CAGCAGTAGGGAATTTTCGGCAATGGGGGAAACCCTGACCGAGCAACGCCGCGTGAGTGAAGAAGGCCTTCGGGTTGTAA<br>AGCTCTGTTGTAAGGGAAGAAGAGATCATAGAGGGAATGCTATGATTGTGACGGTACCTTACCAGAAAGCCACGGCTAACTA<br>CGTGCCAGCAGCCGCGGTAATACGTAGGTGGCGAGCGTTATCCGGAATTATTGGGCGTAAAGGGTGCGTAGGCGGCACA<br>TTAAGTCTAAGGTTAAAGTTCGAGGCTCAACTTCGGATTGCCTTAGAAACTGGTGAGCTAGAGTGCAGGAGAGGGCAATGGA<br>ATTCCATGTGTAGCGGTAAAATGCGTAGATATATGGAGGAACACCAGTGGCGAAGGCGGTTGCCTGGCCTGTAACTGACGC<br>TGAGGCACGAAAGCGTGGGGAGCAAATAGGATTAGATACCCTAGTAGTCCACGCTGTAAACGATGATTACTAAGTGTTGGAG<br>AAATTCAGTGCTGCAGTTAACGCAATAAGTAATCCGCCTGGGGAGTATGCACGCAAGTGTGAAACTCAAAGGAATTGACGGG<br>GGCCCGCACAAGCGGTGGAGTATGTGGTTTAATTCGAAGCAACGCGAAGAACCTTACCAGGTCTTGACATACCGGGCAAA<br>GCTCTAGAGATAGAGAAATAGTTATGCCGGATACAGGTGGTGCATGGTTGTCGTCAGCTCGTGTCGTGAGATGTTGGGTTA<br>AGTCCCGCAACGAGCGCAACCCTTGTCTTTAGTTACCATCATTCAGTTGGGGACTCTAAAGAGACTGCCGGTGACAAACCG<br>GAGGAAGGTGGGGATGACGTCAAATCATCATGCCCCTTATGACCTGGGCTACACACGTACTACAATGGCGCATACAACGG<br>GAAGCGAAGTGGTGACATGGAGCAAATCTCAAAAAGTGCGTCTCAGTTCAGATTGAAGTCTGCAACTCGACTTCATGAAGTC<br>GGAATCGCTAGTAATCGCGGATCAGAATGCCGCGGTGAATACGTTCTCGGGCCTTGTACACACCGCCCGTCAAACCACGA<br>AAGTTGATAATACCCGAAGCCGGTGGGCTAACCGCAAGGAGGCAGCCGTCGAAGGTAGGATTGATGATTGGGGTT
78	Lachnospiraceae gen. sp. OTU_128	GATGAACGCTGGCGGCGTGCTTAACACATGCAAGTCGAACGGAGTTTGTATGGAATAAGTCTTCGGACAAATGAAATATAAA<br>CTTAGTGGCGGACGGGTGAGTAACGTGTGGGTAACCTACCCTATACAGGAGGATAACAGTAAGAAATTATTGCTAAAACTGC<br>ATAAGATTACGAGTTCGCATGAACAAGTGATAAAAACTTTAGTGGTATAGGATGGGCCCGCATCTGATTAGGTAGTTGGTGGA<br>GTAAAAGCCTACCAAGCCGACGATCAGTAGCCGATCTGAGAGGATGACCGGCCACATTGGGACTGAGACACGGCCCAAAC<br>TCCTACGGGAGGCAGCAGTGGGGAATATTGGACAATGGGGGAAACCCTGATCCAGCGACGCCGCGTGAGTGAAGAAGTAT<br>TTCGGTATGTAAAGCTCTATCAGCAGGGAAGAAAATGACGGTACCTGACTAAGAAGCCCCGGCTAACTACGTGCCAGCAGC<br>CGCGGTAATACGTAGGGGGCAAGCGTTATCCGGATTTACTGGGTGTAAAGGGAGCGTAGACGGCGATGTAAGTCTGAAGT<br>GAAAGCCCACGGCTCAACTGTGGGACTGCTTTGGAAACTATATAGCTAGAGTATCGGAGGGGCAAGCGGAATTCCTAGTGT<br>AGCGGTGAAATGCGTAGATATTAGGAGGAACACCGGAGGCGAAGGCGGCTTGCTGGACGAAGACTGACGTTGAGGCTCGA<br>AGGCGTGGGGAGCAAACAGGATTAGATACCCTGGTAGTCCACGCAGTAAACGATGAATACTAGATGTCGGAGAGGTTACTT<br>TTCGGTGTCGTCGCAAACGCAATAAGTATTCCACCTGGGGAGTACGTTCGCAAGAATGAAACTCAAAGGAATTGACGGGGA<br>CCCGCACAAGCGGTGGAGCATGTGGTTTAATTCGAAGCAACGCGAAGAACCTTACCAAGTCTTGACATACCTTTGAATAGT<br>GAGTAAAGTTACTAGCCCTTCGGGGCAGGGGATACAGGTGGTGCATGGTTGTCGTCAGCTCGTGTCGTGAGATGTTGGGT<br>TAAGTCCCGCAACGAGCGCAACCCTTGTTCTTAGTAGCCAGCAGTAAGATGGGAACTCTAAGAAGACTGCCATGGATAACA<br>TGGAGGAAGGTGGGGATGACGTCAAATCATCATGCCCCTTATGATTTGGGCTACACACGTGCTACAATGGCGTAAACAGAG<br>GGAAGCGAAAGAGAGATCTGGAGCAAATCCCAAAAATAACGTCTCAGTTCGGATTGTAGGCTGCAACTCGCCTACATGAAG<br>CTGGAATCGCTAGTAATCGCAAATCAGAATGTTGCGGTGAATACGTTCCCGGGTCTTGTACACACCGCCCGTCACACCATG<br>GGAGTCATCAATGCCCGAAGTCAGTGACCTAACCCTTATCAGTGGTTATGAACCGCAAGGGTTGAGAAATGATAAATCATTT<br>CTCAACATTATAAAATACATCTGACGTATATTTGGAATATATAGTTATGGGAGAGATTTAATAGGAATTTATCATTTCTCAACATT<br>CTATAAAGAATATGTCTGATATAAAAAGATGTAAATATAAGAGAAGAAGTTCATAGCTAGTGATAAGGGGAGGAGCTGCCGAAG<br>GCAGGGAGGATAACTGGGGTG
79	Propionivibrio sp. OTU_130	ATTGAACGCTGGCGGCATGCCTTACACATGCAAGTCGGACGGCAGCACGGGTGCTTGCACCTGGTGGCGAGTGGCGAAC<br>GGGTGAGTAAAGCATCGGAACGTACCCGGGAGTGGGGGATAACGTAGCGAAAGTTACGCTAATACCGCATACCCTGTGAG<br>CAGGAAAGCGGGGGCTCGCAAGACCTCGCGCTTTCGGAGCGGCCGATGTCGGATTAGCTAGTTGGTGGGGTAAAGGCCT<br>ACCAAGGCAACGATCCGTAGCGGGTCTGAGAGGATGATCCGCCACACTGGGACTGAGACACGGCCCAGACTCCTACGGG<br>AGGCAGCAGTGGGGAATTTTGGACAATGGGCGCAAGCCTGATCCAGCCATGCCGCGTGAGTGAAGAAGGCCTTCGGGTT<br>GTAAAGCTCTTTTGTTGGGAAAGAAAAGATTCGGGCTAATACCCTGGATTAATGACGGTACCCAAAGAATAAGCACCGGCTA<br>ACTACGTGCCAGCAGCCGCGGTAATACGTAGGGTGCAAGCGTTAATCGGAATTACTGGGCGTAAAGCGTGCGCAGGCGGT<br>TGTGTAAGTCAGAGGTGAAATCCCCGGGCTCAACCTGGGAACTGCCTTTGAGACTGTGCAACTAGAGGATGGCAGAGGGG<br>GGTAGAATTCCACGTGTAGCAGTGAAATGCGTAGAGATGTGGAGGAATACCGATGGCGAAGGCAGCCCCCTGGGCTATAA<br>CTGACGCTCATGCACGAAAGCGTGGGGAGCAAACAGGATTAGATACCCTGGTAGTCCACGCCCTAAACGATGTCAACTAGA<br>TGTTGGGCGGGTTAAACCGCTTAGTGTCGTAGCTAACGCGTGAAGTTGACCGCCTGGGGAGTACGGCCGCAAGGCTAAAA<br>CTCAAAGGAATTGACGGGGACCCGCACAAGCGGTGGATGATGTGGATTAATTCGATGCAACGCGAAAAACCTTACCTGGTC<br>TTGACATGTACGGAACCTTCCAGAGACGGAAGGGTGCCTTCGGGAACCGTAACACAGGTGCTGCATGGCTGTCGTCAGCT<br>CGTGTCGTGAGATGTTGGGTTAAGTCCCGCAACGAGCGCAACCCTTGCCACTAATTGCCATCATTTAGTTGGGCACTTTAG<br>TGGGACTGCCGGTGACAAACCGGAGGAAGGTGGGGATGACGTCAAGTCCTCATGGCCCTTATGACCAGGGCTTCACACG<br>TCATACAATGGTCGGTTCAGAGGGTAGCCAAGTCGCGAGGCGGAGCCAATCCCAGAAAGCCGATCGTAGTCCGGATTGCA<br>GTCTGCAACTCGACTGCATGAAGTCGGAATCGCTAGTAATCGCGGATCAGCATGTCGCGGTGAATACGTTCCCGGGTCTT<br>GTACACACCGCCCGTCACACCATGGGAGCGGGTTCTGCCAGAAGTGGGTAGCTTAACCGAAAGGAGAGCGCTTACCACG<br>GCGGGGTTCGTGACTGGGGTG
80	Campylobacter sp. OTU_134	AGTGAACGCTGGCGGCGTGCCTAATACATGCAAGTCGAACGGAGATTAAGTAGCTTGCTATTTAATCTTAGTGGCGCACGG<br>GTGAGTAATGTATAGCTAATCTGCCCCATAGTGGAGGACAACAGTTGGAAACGACTGCTAATACTCCATACTCCATTTGTATA<br>TAAGTATAAATGGGAAAGTAGCCCTATGTAATGTTTATTTAAGCAATTAGATGAAAATTAGACTAAACCTAAATTTATGATTTATG<br>ACAAGCGAAGCTTGGAGTGAATCTTTATAACTTTAGGTGGGTGCAGGGAGCGAAACTCCCGCTCGTAAAGACTAGCTTGGC<br>TAGTCTGCGAAGTCGTAATATTTAATTGCGAAGCAAGTAAATATTACATAGGGTTTCTCGCTATGGGATGAGGCTATATCGTA<br>TCAGCTAGTTGGTAGGGTAATGGCTTACCAAGGCTATGACGCGTAACTGGTCTGAGAGGATGATCAGTCACACTGGAACTG<br>AGACACGGTCCAGACTCCTACGGGAGGCAGCAGTAGGGAATATTGCTCAATGGGGGAAACCCTGAAGCAGCAACGCCGC<br>GTGGAGGATGACACTTTTCGGAGCGTAAACTCCTTTTCTTGGGGAAGAAATTTGACGGTACCCAAGGAATAAGCACCGGCT<br>AACTCCGTGCCAGCAGCCGCGGTAATACGGAGGGTGCAAGCGTTACTCGGAATCACTGGGCGTAAAGGACGCGTAGGCG<br>GATTATCAAGTCTCTTGTGAAATCCAATGGCTTAACCATTGAACTGCTTGAGAAACTGATAATCTAGAGTGAGGGAGAGGCAG<br>ATGGAATTGGTGGTGTAGGGGTAAAATCCGTAGAGATCACCAGGAATACCCATTGCGAAGGCGATCTGCTGGAACTCAACT<br>GACGCTAATGCGTGAAAGCGTGGGGAGCAAACAGGATTAGATACCCTGGTAGTCCACGCCCTAAACGATGTATACTAGTTG<br>TTGCTAAGCTAGTCTTGGCAGTAATGCACCTAACGGATTAAGTATACCGCCTGGGGAGTACGGTCGCAAGATTAAAACTCAA<br>AGGAATAGACGGGGACCCGCACAAGCGGTGGAGCATGTGGTTTAATTCGAAGATACGCGAAGAACCTTACCCGGACTTGAT<br>ATCTAACAAATCATCTAGAGATAGAAGAGTGTCTGCTTGCAGAAATGTTAAGACAGGTGCTGCACGGCTGTCGTCAGCTCGT<br>GTCGTGAGATGTTGGGTTAAGTCCCGCAACGAGCGCAACCCACGTCATTAGTTGCTAACGGTTCGGCCGAGCACTCTAAT<br>GAGACTGCCTTCGCAAGGAGGAGGAAGGTGTGGACGACGTCAAGTCATCATGGCCCTTATGTCCGGGGCGACACACGTG<br>CTACAATGGCATATACAATGAGACGCAATATCGCGAGATGGAGCAAATCTATAAAATATGTCCCAGTTCGGATTGGAGTCTG<br>CAACTCGACTCCATGAAGCCGGAATCGCTAGTAATCGTAGATCAGCCATGCTACGGTGAATACGTTCCCGGGTCTTGTACT<br>CACCGCCCGTCACACCATGGGAGTTGATTTCACTCGAAGCCCAAATACCAAACCGGTTATGGTCCACAGTGGAATCAGCG<br>ACTGGGGTG

Sr no.	Species_OTUs	16S reference sequence
81	Actinomyces sp. OTU_141	GACGAACGCTGGCGGCGTGCTTAACACATGCAAGTCGAACGGTGAAGCGCCTTCCTTTTTGGTGGGTGTGGATGAGTGGC<br>GAACGGGTGAGTAACACGTGAGTAACCTGCCCCCTTCTTCTGGATAACCGCATGAAAGTGTGGCTAATACGGGATATTCTG<br>TGCCTGCTGCATGGTGGGTGTGGGAAAGATTGCGCCTTTTTTGGGTGTTTTTGGTGGGGGATGGGCTCGCGGCCTATCAG<br>CTTGTTGGTGGGGTGATGGCCTACCAAGGCGGTGACGGGTAGCCGGCCTGAGAGGGTGGACGGTCACACTGGGACTGA<br>GACACGGCCCAGACTCCTACGGGAGGCAGCAGTGGGGAATATTGCACAATGGGCGCAAGCCTGATGCAGCGACGCCGC<br>GTGAGGGATGGAGGCCTTCGGGTTGTGAACCTCTTTCGCCAGTGAAGCAGGCCTGCTCCTTGGTGGGTGGGTTGACGGT<br>AGCTGGATAAGAAGCGCCGGCTAACTACGTGCCAGCAGCCGCGGTAATACGTAGGGCGCGAGCGTTGTCCGGAATTATTG<br>GGCGTAAAGAGCTCGTAGGCGGCTGGTCGCGTCTGTCGTGAAATCCTCTGGCTTAACTGGGGGCTTGCGGTGGGTACGG<br>GCCGGCTTGAGTGCGGTAGGGGAGGCTGGAACTCCTGGTGTAGCGGTGGAATGCGCAGATATCAGGAAGAACACCGGTG<br>GCGAAGGCGGGTCTCTGGGCCGTTACTGACGCTGAGGAGCGAAAGCGTGGGGAGCGAACAGGATTAGATACCCTGGTAG<br>TCCACGCCGTAAACGTTGGGCACTAGGTGTGGGGGGCCTTTTCCGGGTTTTCCGCGCCGTAGCTAACGCATTAAGTGCC<br>CCGCCTGGGGAGTACGGCCGCAAGGCTAAAACTCAAAGGAATTGACGGGGGCCCGCACAAGCGGCGGAGCATGCGGATT<br>AATTCGATGCAACGCGAAGAACCTTACCAAGGCTTGACATGTGCCGGTCGGCTCCGGAGACGGGGTTTCCTCCTTGTGGG<br>GCCGGTTCACAGGTGGTGCATGGTTGTCGTCAGCTCGTGTCGTGAGATGTTGGGTTAAGTCCCGCAACGAGCGCAACCC<br>TTGTCTCGTGTTGCCAGCACGTTGTGGTGGGGACTCGCGGGAGACTGCCGGGGTCAACTCGGAGGAAGGTGGGGATGAC<br>GTCAAATCATCATGCCCCTTATGTCTTGGGCTTCACGCATGCTACAATGGCTGGTACAGAGGGCTGCGATACTGTGAGGTG<br>GAGCGAATCCCTTAAAGCCGGTCTCAGTTCGGATCGGTGTCTGCAACTCGACACCGTGAAGTTGGAGTCGCTAGTAATCG<br>CAGATCAGCAACGCTGCGGTGAATACGTTCTCGGGCCTTGTACACACCGCCCGTCACGTCATGAAAGTCGGCAACACCC<br>GAAGCCCGTGGCCTTATGGGGAGCGGTCGAAGGTGGGGCTGGTGATTGGGACG
82	Brachymonas sp. OTU_142	ATTGAACGCTGGCGGCATGCTTTACACATGCAAGTCGAACGGCAGCGGAGAGGTGCTTGCACTTCTGCCGGCGAGTGGC<br>GAACGGGTGAGTAATGTATCGGAACGTGCCCAGTAGTGGGGGATAACTACTCGAAAGAGTGGCTAATACCGCATGAGATCT<br>CTGGATGAAAGCGGGGGCCGTAAGGCCTCGCGCTACTGGAGCGGCCGATATCAGATTAGGTAGTTGGTGGGGTAAAGGC<br>CTACCAAGCCTGCGATCTGTAGCTGGTTTGAGAGGGCGACCAGCCACACTGGGACTGAGACACGGCCCAGACTCCTACG<br>GGAGGCAGCAGTGGGGAATTTTGGACAATGGGCGCAAGCCTGATCCAGCAATGCCGCGTGCAGGATGAAGGCCTTCGGG<br>TTGTAAACTGCTTTTGTACAGAACGAAAAGCTCTTCTCTAATACAGGGGGGTAATGACGGTACTGTAAGAATAAGCACCGGC<br>TAACTACGTGCCAGCAGCCGCGGTAATACGTAGGGTGCGAGCGTTAATCGGAATTACTGGGCGTAAAGCGTGCGCAGGCG<br>GTTTTGTAAGACAGAGGTGAAATCCCCGGGCTTAACCTGGGAACTGCCTTTGTGACTGCAAGGCTTGAGTGCGGCAGAGG<br>GGGATGGAATTCCGCGTGTAGCAGTGAAATGCGTAGATATGCGGAGGAACACCGATGGCGAAGGCAGTCCCCTGGGCCT<br>GCACTGACGCTCATGCACGAAAGCGTGGGGAGCAAACAGGATTAGATACCCTGGTAGTCCACGCCCTAAACGATGTCAAC<br>TGGTTGTTGGAAATTTATTTTTCAGTAACGAAGCTAACGCGTGAAGTTGACCGCCTGGGGAGTACGGCCGCAAGGTTGAAA<br>CTCAAAGGAATTGACGGGGACCCGCACAAGCGGTGGATGATGTGGTTTAATTCGATGCAACGCGAAAAACCTTACCCACCT<br>TTGACATGGCAGGAAGTTTCCAGAGATGGATTCGTGCTCGCAAGAGAACCTGCGCACAGGTGCTGCATGGCTGTCGTCAG<br>CTCGTGTCGTGAGATGTTGGGTTAAGTCCCGCAACGAGCGCAACCCTTGCCATTAGTTGCTACGAAAGGGCACTCTAATG<br>GGACTGCCGGTGACAAACCGGAGGAAGGTGGGGATGACGTCAAGTCCTCATGGCCCTTATAGGTGGGGCTACACACGTC<br>ATACAATGGCCGGTACAAAGGGTAGCCAACCCGCGAGGGGGAGCCAATCCCGCAAAGCCGGTCGTAGTCCGGATCGCAG<br>TCTGCAACTCGACTGCGTGAAGTCGGAATCGCTAGTAATCGTGGATCAGCATGTCACGGTGAATACGTTCCCGGGTCTTGT<br>ACACACCGCCCGTCACACCATGGGAGCGGGTTCTGCCAGAAGTGGTTAGCCTAACCGTAAGGAGGGCGATCACCACGGC<br>AGGGTTCGTGACTGGGGTG
83	Centipeda sp. OTU_143	GACGAACGCTGGCGGCGTGCTTAACACATGCAAGTCGAACGGGATGATTTAGAAGCTTGCTTTTAGAGAATCTAGTGGCAA<br>ATGGGTGAGTAACACGTAGGCAACCTGCCGACAGGATGGGGACAACATTCCGAAAGGAATGCTAATACCGAATGAAGTCAG<br>AGAAGGGCATCCTTTTCTGATGAAAGATGGCCTCTATATATAAGCTATCACCTGTCGATGGGCCTGCGTCTGATTAGCTGGT<br>TGGTGAGGTAACGGCTCACCAAGGCGACGATCAGTAGCCGGTCTGAGAGGATGAACGGCCACATTGGGACTGAGACACG<br>GCCCAGACTCCTACGGGAGGCAGCAGTGGGGAATCTTCCGCAATGGGCGCAAGCCTGACGGAGCAACGCCGCGTGAGT<br>GAAGAAGGTCTTCGGATCGTAAAGCTCTGTTGATGGGGACGAACGTGCGAAGGGTGAATAATCCTTTGTAATGACGGTACC<br>TATCGAGGAAGCCACGGCTAACTACGTGCCAGCAGCCGCGGTAATACGTAGGTGGCGAGCGTTGTCCGGAATCATTGGG<br>CGTAAAGGGAGCGCAGGCGGGCATGTAAGTCTTTCTTAAAAGTGCGGGGCTCAACCCCGTGATGGGAAAGAAACTACATGT<br>CTTGAGTACAGGAGAGGAAAGCGGAATTCCCAGTGTAGCGGTGAAATGCGTAGATATTGGGAGGAACACCAGTGGCGAAGG<br>CGGCTTTCTGGACTGCAACTGACGCTGAGGCTCGAAAGCCAGGGGAGCGAACGGGATTAGATACCCCGGTAGTCCTGGC<br>CGTAAACGATGGATACTAGGTGTGGGAGGTATCGACCCCTACCGTGCCGGAGTTAACGCAATAAGTATCCCGCCTGGGGA<br>GTACGGTCGCAAGACTGAAACTCAAAGGAATTGACGGGGACCCGCACAAGCGGTGGAGTATGTGGTTTAATTCGAAGCAAC<br>GCGAAGAACCTTACCAGGCCTTGACATTGACTGAAGTTACTAGAGATAGTAATGTTTCTTCGGAAACAGGAAAACAGGTGGT<br>GCATGGCTGTCGTCAGCTCGTGTCGTGAGATGTTGGGTTAAGTCCCGCAACGAGCGCAACCCCTGTTCTTTGTTGCCAGC<br>GCGTAATGGTGGGCACTCAAAGGAGACTGCCGCGGAGAACGCGGAGGAAGGCGGGGATGACGTCAAGTCATCATGCCCC<br>TTATGGTCTGGGCTACACACGTACTACAATGGGACGGACAGAGAGCAGCGAACCCGCGAGGGCGAGCGAACCTCAAAAAC<br>CGTTTCTCAGTTCGGATTGCAGGCTGCAACTCGCCTGCATGAAGTCGGAATCGCTAGTAATCGCAGGTCAGCATACTGCG<br>GTGAATACGTTCCCGGGTCTTGTACACACCGCCCGTCACACCACGGAAGTCGTTCACACCCGAAGCCGGCTAAGCCGTC<br>TAAGGTGGGGAAGGTGACTGGGGTG
84	Selenomonas sp. OTU_155	GACGAACGCTGGCGGCGTGCTTAACACATGCAAGTCGAACGGGAACCTTTATTTCGGTAAGGGTGAGAGTGGCAAACGGG<br>TGAGTAACACGTAGGCAACCTGCCGACAGGATGGGGACAACATTCCGAAAGGAATGCTAATACCGAATGAAGTCCGAGAGG<br>GGCATCCCTCTTGGATGAAAGATGGCCTCTGAACATGCTATCACCTGTCGATGGGCCTGCGTCTGATTAGCCAGTTGGCG<br>GGGTAACGGCCCACCAAAGCGACGATCAGTAGCCGGTCTGAGAGGATGAACGGCCACATTGGGACTGAGACACGGCCCA<br>GACTCCTACGGGAGGCAGCAGTGGGGAATCTTCCACAATGGGCGAAAGCCTGATGGAGCAACGCCGCGTGAGTGAAGAA<br>GGTCTTCGGATCGTAAAGCTCTGTTGCAGGGGACGAAAACCGGGTTTGAAAACATTGAGCCTGGGTGACGGTACCTTGCG<br>AGGAAGCCACGGCTAACTACGTGCCAGCAGCCGCGGTAATACGTAGGTGGCGAGCGTTGTCCGGAATTATTGGGCGTAAA<br>GGGAGCGCAGGCGGGCTGCCAAGTCCATCTTAAAAGTGCGGGGCTCAACCCCGTGAGGGGATGGAAACTGGCAGTCTTG<br>AGTGCAGGAGAGGAAAGCGGAATTCCCAGTGTAGCGGTGAAATGCGTAGATATTGGGAGGAACACCAGTGGCGAAGGCGG<br>CTTTCTGGACTGTAACTGACGCTGAGGCTCGAAAGCCAGGGGAGCGAACGGGATTAGATACCCCGGTAGTCCTGGCCGTA<br>AACGATGAATGCTAGGTGTAGGAGGTATCGACCCCTCCTGTGCCGGAGTTAACGCAATAAGCATTCCGCCTGGGGAGTAC<br>GGTCGCAAGACTGAAACTCAAAGGAATTGACGGGGGCCCGCACAAGCGGTGGAGTATGTGGTTTAATTCGACGCAACGCG<br>AAGAACCTTACCAGGGCTTGACATTGAGTGAAACGTGTAGAGATACACGCCTCCCTTCGGGGACACGAAAACAGGTGGTGC<br>ATGGCTGTCGTCAGCTCGTGTCGTGAGATGTTGGGTTAAGTCCCGCAACGAGCGCAACCCCTGTCCCTTGTTGCCAGCA<br>CGTAAAGGTGGGAACTCAAGGGAGACTGCCGTGGAGAACGCGGAGGAAGGCGGGGATGACGTCAAGTCATCATGCCCCT<br>TATGTCCTGGGCTACACACGTACTACAATGGGATGGACAGAGGGAAGCGAGGGCGCGAGCCGGAGCGGATCCCAGAAAC<br>CATCCCCCAGTTCGGATTGCAGGCTGCAACCCGCCTGCATGAAGTCGGAATCGCTAGTAATCGCAGGTCAGCATACTGCG<br>GTGAATACGTTCCCGGGCCTTGTACACACCGCCCGTCACACCACGGAAGTCATTCACGCCCGAAGCCGGCGGGCCAAC<br>CGCAAGGAGGCAGCCGTCTAAGGCGGGGGCGATGACTGGGGTG

Sr no.	Species_OTUs	16S reference sequence
85	Selenomonadaceae gen. sp. OTU_160	GACGAACGCTGGCGGCGTGCTTAACACATGCAAGTCGAACGAAATTAAAAAATTATCATTGAGCTCTCCGATGAGATGGGAG<br>CCAAGCGTCGTGACCGCGGAGCGGGCACCACGCGTTCAACAAGTGCAACACCAAGTCGAAGGGTTGAATGGTAATTTTTT<br>AATTTAGTGGCAAACGGGTGAGTAACACGTAGACAACCTGCCGACAGGATGGGGACAACATTCCGAAAGGAATGCTAATAC<br>CGAATGAAGCGGAGGAGAGGCATCTCTCCTGCGTGAAAGATGGCCTCTATTTATAAGCTATCACCTGTCGATGGGTCTGCG<br>TCTGATTAGCTAGTTGGTGAGGTAACGGCTCACCAAGGCGACGATCAGTAGCCGGTCTGAGAGGATGAACGGCCACATTG<br>GGACTGAGACACGGCCCAGACTCCTACGGGAGGCAGCAGTGGGGAATCTTCCGCAATGGGCGCAAGCCTGACGGAGCAA<br>CGCCGCGTGAGTGAAGAAGGTCTTCGGATCGTAAAGCTCTGTTGACGGGGACGAACGTGCGGAGTGCGAATAGCGCTTTG<br>TAATGACGGTACCTGTCGAGGAAGCCACGGCTAACTACGTGCCAGCAGCCGCGGTAATACGTAGGTGGCGAGCGTTGTC<br>CGGAATCATTGGGCGTAAAGGGAGCGCAGGCGGGCCGGTAAGTCTTACTTAAAAGTGCGGGGCTCAACCCCGTGATGGG<br>AGAGAAACTATCGGTCTTGAGTACAGGAGAGGAAAGCGGAATTCCCAGTGTAGCGGTGAAATGCGTAGATATTGGGAAGAAC<br>ACCAGTGGCGAAGGCGGCTTTCTGGACTGCAACTGACGCTGAGGCTCGAAAGCCAGGGGAGCGAACGGGATTAGATACC<br>CCGGTAGTCCTGGCCGTAAACGATGGATACTAGGTGTGGGAGGTATCGACCCCTACCGTGCCGGAGTTAACGCAATAAGT<br>ATCCCGCCTGGGGAGTACGGCCGCAAGGCTGAAACTCAAAGGAATTGACGGGGACCCGCACAAGCGGTGGAGTATGTGG<br>TTTAATTCGAAGCAACGCGAAGAACCTTACCAGGCCTTGACATTGACTGAAAGTGCTAGAGATAGCACCCTCTCTTCGGAGA<br>CAGGAAAACAGGTGGTGCATGGCTGTCGTCAGCTCGTGTCGTGAGATGTTGGGTTAAGTCCCGCAACGAGCGCAACCCCT<br>GTTCTTTGTTGCCATCAGGTAAAGCTGGGCACTCAAAGGAGACTGCCGCGGAGAACGCGGAGGAAGGCGGGGATGACGT<br>CAAGTCATCATGCCCCTTATGGCCTGGGCTACACACGTACTACAATGGAACGGACAGAGAGCAGCGAACCCGCGAGGGCA<br>AGCGAACCTCAAAAACCGTTTCCCAGTTCGGATTGCAGGCTGCAACCCGCCTGCATGAAGTCGGAATCGCTAGTAATCGC<br>AGGTCAGCATACTGCGGTGAATACGTTCCCGGGTCTTGTACACACCGCCCGTCACACCACGGAAGTCATTCACACCCGAA<br>GCCGGCGCAGCCGTCTAAGATGGGGAAGGTGACTGGGGTG
86	Aggregatibacter sp. OTU_165	ATTGAACGCTGGCGGCAGGCTTAACACATGCAAGTCGAACGGTAGCAGGTAAGTACTTGTACTTATGCTGACGAGTGGCGG<br>ACGGGTGAGTAATGCTTGGGGATCTGGCTTATGGAGGGGGATAACGACGGGAAACTGTCGCTAATACCGCGTAGAATCGA<br>GAGATGAAAGTGTGGGACCTTCGGGCCACATGCCATAGGATGAACCCAAGTGGGATTAGGTAGTTGGTAGGGTAATGGCCT<br>ACCAAGCCGACGATCTCTAGCTGGTCTGAGAGGATGACCAGCCACACCGGGACTGAGACACGGCCCGGACTCCTACGGG<br>AGGCAGCAGTGGGGAATATTGCGCAATGGGGGCAACCCTGACGCAGCCATGCCGCGTGAATGAAGAAGGCCTTCGGGTT<br>GTAAAGTTCTTTCGGTGACGAGGAAGGTTGTTGTGTGAATAGCACAACAAATTGACGTTAATCACAGAAGAAGCACCGGCTA<br>ACTCCGTGCCAGCAGCCGCGGTAATACGGAGGGTGCGAGCGTTAATCGGAATAACTGGGCGTAAAGGGCACGCAGGCGG<br>ACTTTTAAGTGAGGTGTGAAATCCCCGGGCTTAACCTGGGAATTGCATTTCAGACTGGGGGTCTAGAGTACTTTAGGGAGG<br>GGTAGAATTCCACGTGTAGCGGTGAAATGCGTAGAGATGTGGAGGAATACCGAAGGCGAAGGCAGCCCCTTGGGAATGTA<br>CTGACGCTCATGTGCGAAAGCGTGGGGAGCAAACAGGATTAGATACCCTGGTAGTCCACGCTGTAAACGCTGTCGATTTG<br>GGGATTGGGCTTTGAGCTTGGTGCCCGTAGCTAACGTGATAAATCGACCGCCTGGGGAGTACGGCCGCAAGGTTAAAACT<br>CAAATGAATTGACGGGGGCCCGCACAAGCGGTGGAGCATGTGGTTTAATTCGATGCAACGCGAAGAACCTTACCTACTCTT<br>GACATCCATGGAATCCTGTAGAGATACGGGAGTGCCTTCGGGAACCATGAGACAGGTGCTGCATGGCTGTCGTCAGCTCG<br>TGTTGTGAAATGTTGGGTTAAGTCCCGCAACGAGCGCAACCCTTATCCTTTGTTGCCAGCGATTAGGTCGGGAACTCAAAG<br>GAGACTGCCGGTGATAAACCGGAGGAAGGTGGGGATGACGTCAAGTCATCATGGCCCTTACGAGTAGGGCTACACACGTG<br>CTACAATGGCGTATACAGAGGGCGACGAAGCCGCGAGGTGGAGTGAATCTCGGAAAGTACGTCTAAGTCCGGATTGGAGT<br>CTGCAACTCGACTCCATGAAGTCGGAATCGCTAGTAATCGCGAATCAGAATGTCGCGGTGAATACGTTCCCGGGCCTTGTA<br>CACACCGCCCGTCACACCATGGGAGTGGGTTGTACCAGAAGTAGATAGCTTAACCGCAAGGGGGGCGTTTACCACGGTAT<br>GATTCATGACTGGGGTG
87	Prevotella sp. OTU_166	GATGAACGCTAGCTATAGGCTTAACACATGCAAGTCGAGGGGAAACGATGAGGAAGCTTGCTTCTTTAGGCGTCGACCGGC<br>GAATGGGTGAGTAACGCGTATCCAACCTGCCCTTAAGTAGGGAACAGCCCAGTGAAAATTGGATTAATGCCCTATGTTCTTC<br>CACGAGGTCCTCCGAGTGGGAGCAAAGATTTATCGCTTAAGGATGGGGATGCGTCTGATTAGCTTGTTGGCGGGGTAACG<br>GCCCACCAAGGCAACGATCAGTAGGGGTTCTGAGAGGAAGGTCCCCCACATTGGAACTGAGACACGGTCCAAACTCCTAC<br>GGGAGGCAGCAGTGAGGAATATTGGTCAATGGGCGAGAGCCTGAACCAGCCAAGTAGCGTGCAGGATGACGGCCCTATG<br>GGTTGTAAACTGCTTTTATACGGGGATAAAGTGGCGAACGTGTTTGCTATTGCAGGTACCGTATGAATAAGGACCGGCTAAT<br>TCCGTGCCAGCAGCCGCGGTAATACGGAAGGTCCAGGCGTTATCCGGATTTATTGGGTTTAAAGGGAGCGTAGGCCGCTG<br>ATTAAGCGTGTTGTGAAATTTGGATGCTCAACATCTGAACTGCAGCGCGAACTGGTTAGCTTGAGTGTGCGCAACGCAGGC<br>GGAATTTGTGGTGTAGCGGTGAAATGCTTAGATATCACGAAGAACTCCGATTGCGAAGGCAGCTTGCGGGAGCACAACTGA<br>CGCTGAAGCTCGAAAGCGCGGGTATCGAACAGGATTAGATACCCTGGTAGTCCGCGCGGTAAACGATGGATGCCCGCTAT<br>TCGCTCATTTCTGAGCGTGTGGCCAAGCGAAAGCGTTAAGCATCCCACCTGGGGAGTACGCCGGCAACGGTGAAACTCAA<br>AGGAATTGACGGGGGCCCGCACAAGCGGAGGAACATGTGGTTTAATTCGATGATACGCGAGGAACCTTACCCGGGCTTGA<br>ATTGCAGAGGAAAGATCCAGAGATGGTGATGCCCTTCGGGGCTTCTGTGAAGGTGCTGCATGGTTGTCGTCAGCTCGTGC<br>CGTGAGGTGTCGGCTTAAGTGCCATAACGAGCGCAACCCCTCTCCTTAGTTGCCATCGGGTCATGCCGGGCACTCTGGG<br>GACACTGCCACCGCAAGGTGTGAGGAAGGTGGGGATGACGTCAAATCAGCACGGCCCTTACGTCCGGGGCTACACACGT<br>GTTACAATGGGGGGTACAGAGTGTCGGGTGCGCGCAAGCTCACTCCAATCAATAAATCCTCCCTCAGTTCGGACTGGGGT<br>CTGCAACCCGACCCCACGAAGCTGGATTCGCTAGTAATCGCGCATCAGCCATGGCGCGGTGAATACGTTCCCGGGCCTT<br>GTACACACCGCCCGTCAAGCCATGAAAGCCGGGGGTGCCTGAAGTCCGTGACCGCAAGGATCGGCCTAGGGCAAAACTG<br>GTGATTGGGGCT
88	Bacteroidales gen. sp. OTU_167	GATGAACGCTAGCGGCAGGCTTAACACATGCAAGTCGAGGGGCATCGAGATGTAGCAATACATTGTCGGCGACCGGCGGA<br>CGGGTGCGTAACGCGTGAGCAACCTTCCCGTATCAGGGGGATAACCGATGGAAACGTCGCCTAATACCCCATAACAACAC<br>ATGTCTCCTGACGTGTGTTTGAAAGATTCGTCAGATACGGCTGGGCTCGCGTGACATTAGCTAGTTGGTGAGGTAACGGCT<br>CACCAAGGCAACGATGTCTAGGGGTTCTGAGAGGAAGACCCCCCACACTGGAACTGAGACACGGTCCAGACTCCTACGG<br>GAGGCAGCAGTGAGGAATATTGGTCAATGGGCGCAAGCCTGAACCAGCCATGCCGCGTGAAGGAATAAGGTCCTATGGAT<br>CGTAAACTTCTTTTGAGCGGGAGCAATAAGGTCTACGTGTAGACCGATGAGAGTACCGTTCGAATAAGCATCGGCTAACTC<br>CGTGCCAGCAGCCGCGGTAATACGGAGGATGCAAGCGTTATCCGGATTTATTGGGTTTAAAGGGTGCGTAGGCCGTTGTG<br>CAAGTCAGAGGTGAAATTGTGGGGCTCAACTCCGCCGCTGCCTTTGAAACTGCATGACTTGAATCCTGTTGCCGTGGGAG<br>GAATGAGTAGTGTAGCGGTGAAATGCATAGATATTACTCAGAACCCCGATTGCGAAGGCATCTCACGAAACAGGTATTGACG<br>CTGAGGCACGAAAGCGTGGGTATCAAACAGGATTAGATACCCTGGTAGTCCACGCTGTAAACGATGATAACTAACCGCCGG<br>CGATATAGTGTCGGTGGCCAAGCGAAAGCGATAAGTTATCCACCTGGGGAGTACGACCGCAAGGTTGAAACTCAAAGGAAT<br>TGACGGGGGCCCGCACAAGCGGAGGAACATGTGGTTTAATTCGATGATACGCGAGGAACCTTACCCGGGCTCGAACGGC<br>AGAGGAAGAGACTCAGAGATGAAGACGTCCCTTCGGGGACCTCTGTCGAGGTGCTGCATGGTTGTCGTCAGCTCGTGTCG<br>TGAGATGTTGGCTCAAGTGCCATAACGAGCGCAACCCTTGTCATTAGTTGCCATCAGGTTAAGCTGGGGACTCTAATGAGA<br>CTGCCACCGCAAGGTGTGAGGAAGGAGTGGATGACGTCAAATCAGCACGGCCCTTACGTCCGGGGCGACACACGTGTTA<br>CAATGGTGACTACAGATGGAAGCCAGCCGGCGACGGCGCGCAAATCTTAAAAAGTCATCTCAGTTCGGACTGGAGTCTGC<br>AACCCGACTCCACGAAGCTGGATTCGCTAGTAATCGCGCATCAGCCATGGCGCGGTGAATACGTTCCCGGGCCTTGTACA<br>CACCGCCCGTCAAGCCATGAAAGCCGAGGGCGCCTGAAGTCCGGGACCTCAAGGACCGGCCTAAGGCGAACTTGGTAAT<br>TGGGGCT

Sr no.	Species_OTUs	16S reference sequence
89	Saccharibacteria sp. OTU_168	GATGAACGCTGGCGGCGTGCCTAACACATGCAAGTCGAGCGGCAGCGAGTTTCTCACTGTTTGTTCCTCTTCTTTCGAGA<br>GCAGGACGCGAAGATTTCCGAAGGAAATCGGTCGCATCTAATACGAAGGAGGAAATCTAGTAAATAATATTTATCAAGATGAG<br>AGGAAAAAACTGTGAGGAATGTCGGCGAGCGGCGGACGGCTGAGTAACGCGTGGGAACATACCCCAAAGTGAGGGATAAC<br>TGCCCGAAAGGGTGGCTAATACCGCATATGATCTTCGGATTAAAGCATTTATGCGCTTTGGGAATGGCCTGCGTCGGATTA<br>GGTAGTTGGTGAGGTAAAGGCTCACCAAGCCGACGATCCGTAACTGGTTTGAGAGGATGACCAGTCAGATTGGAACTGAGA<br>CACGGTCCAGACTCCTACGGGAGGCAGCAGTGAGGAATCTTCCACAATGGGCGAAAGCCTGATGGAGCAACGCCGCGTG<br>CAGGATGAAGGCCTTCGGGTTGTAAACTGCTTTTATGATTGAGGAATTTGACGGTAGATCATGAATAAGGATCGGCTAACTA<br>CGTGCCAGCAGCCGCGGTCATACGTAGGATCCGAGCGTTATCCGGAGTGACTGGGCGTAAAGAGTTGCGTAGGTGGTTTA<br>TTAAGTAGGTGATGAAAGCTGGTGGCTCAACCATTCAGATTGTTATCTAAACTGGTAAACTTGAGAGTAGCAGAGGTAACTGG<br>AATTTCTAGTGTAGGAGTGAAATCCGTAGATATTAGAAGGAACACCAATGGCGTAGGCAGGTTACTGGGCTATTTCTGACAC<br>TAAGGCACGAAAGCGTGGGGAGCGAACGGGATTAGATACCCCGGTAGTCCACGCCCTAAACGATGGATACTAGCTGTTTG<br>AGGAATCGACCCCTTGAGTAGCGAAGCTAACGCGTTAAGTATCCCGCCTGTGGAGTACGATCGCAAGATTAAAACATAAAG<br>GAATTGACGGGGACCCGCACAAGCGGTGGAGCATGTTCTTTAATTCGATGCTAATCGATATACCTTACCAAGGCTTGACAT<br>CTCGGGAAGGCCTCCGAAAGGAGACTGTGCCTTTTAGGAACCCGATGACAGGTGATGCATGGCCGTCGTCAGCTCGTGT<br>CGTGAGATGTTTGGTTAAGTCCATTAACGAGCGCAACCCTTGCAACTAGTTGGATTTTTCTAGTTGGACTGCCCCGGTAAC<br>GGGGAGGAAGGAGGGGATGATGTCAGGTCATTATTTCCCTTACGCCTTGGGCTAGAAACGTGCTACAATGGCTGGTACAAA<br>GTGCATAAGCGAACTCGCGAGAGCAAGCAAATCACATCAAAACCAGTCCCAGTTCGGATTGGAGGCTGAAACTCGCCTCCA<br>TGAAGTCGGAATCGCTAGTAATCGTAAATCAGCAAGTTACGGTGAATACGTTCCCGGGTCTTGTACACACCGCCCGTCAAA<br>CCATGAGAGTGACCAACACCCGAAGTCCGATTCGTCGGCCTAAGGTGGGGGGCATGATTGGGGTT
90	Schwartzia sp. OTU_172	GACGAACGCTGGCGGCGTGCTTAACACATGCAAGTCGAACGGAGCTGTTTATTTCGGTAGATAGCTTAGTGGCAAACGGGT<br>GAGTAACGCGTAGGCAACCTGCCCTTAGGATGGGGACAACGGCCCGAAAGGACCGCTAATACCGAATGGACTCTAACTTT<br>CGCATGAAAGAAAGAGGAAAGATGGTGCAAGCCATCGCCGAAGGAAGGGCCTGCGTCTGATTAGCCAGTTGGTGAGGTAA<br>CGGCCCACCAAAGCGACGATCAGTAGCCGGTCTGAGAGGATGAACGGCCACAATGGGACTGAGACACGGCCCATACTCC<br>TACGGGAGGCAGCAGTGGGGAATCTTCCGCAATGGGCGAAAGCCTGACGGAGCAACGCCGCGTGAGTGAAGAAGGTCTT<br>CGGATCGTAAAGCTCTGTTGTTGGGGACGAAAGGGTAGACGAGGCAATGCGTCTACTAAGACGGTACCCGACGAGGAAGC<br>CACGGCTAACTACGTGCCAGCAGCCGCGGTAATACGTAGGTGGCGAGCGTTGTCCGGAATGATTGGGCGTAAAGGGAGC<br>GCAGGTGGGACGGTAAGTCCTTCTTAAAAGCGTGGGGCTCAGCCCCATGAAGGGAAGGAAACTATCGATCTTGAGTGCCG<br>GAGAGGAAAGCGGAATTCCCAGTGTAGCGGTGAAATGCGTAGATATTGGGAAGAACACCAGTGGCGAAGGCGGCTTTCTG<br>GACGGCAACTGACACTGAGGCTCGAAAGCCAGGGGAGCGAACGGGATTAGATACCCCGGTAGTCCTGGCCGTAAACGAT<br>GGATACTAGGTGTAGGAGGTATCGACCCCTTCTGTGCCGGAGTTAACGCAATAAGTATCCCGCCTGGGGAGTACGGTCGC<br>AAGATTGAAACTCAAAGGAATTGACGGGGGCCCGCACAAGCGGTGGAGTATGTGGTTTAATTCGACGCAACGCGAAGAACC<br>TTACCAGGGCTTGACATTGAGTGAAAAGCGTAGAGATACGCTCCTTATCTTCGGATAACACGAAAACAGGTGGTGCATGGCT<br>GTCGTCAGCTCGTGTCGTGAGATGTTGGGTTAAGTCCCGCAACGAGCGCAACCCCTATGTTCTGTTGCCAGCGCGTAAAG<br>GCGGGCACTCAGAAGAGACTGCCGCGGAGAACGCGGAGGAAGGCGGGGATGACGTCAAGTCATCATGCCCCTTATGACC<br>TGGGCTACACACGTACTACAATGGGATGTACAGAGGGCAGCGAACGGGCGACCGGAAGCGAACCCCAGAAAACATCTCCC<br>AGTTCGGATTGCAGGCTGAAACCCGCCTGCATGAAGATGGAATCGCTAGTAATCGCAGGTCAGCATACTGCGGTGAATACG<br>TTCCCGGGCCTTGTACACACCGCCCGTCACACCACGGAAGTCATTCACACCCGAAGCCGGAGGGAATCCGTCGAAGGTG<br>GGGGCGATGACTGGGGTG
91	Dialister sp. OTU_173	GACGAACGCTGGCGGCGTGCTTAACACATGCAAGTCGAACGGGAAGACATGAAGAGCTTGCTCTTTATGAAATCCAGTGGC<br>AAACGGGTGAGTAACACGTAAACAACCTGCCTTCAGGATGGGGACAACAGACGGAAACGACTGCTAATACCGAATACGATT<br>CTTGAGTCGCATGACACAAGAAAGAAAGGGTGGCCTCTACAAGTAAGCTATCGCCTGAAGAGGGGTTTGCGTCCGATTAGG<br>TAGTTGGTGAGGTAACGGCCCACCAAGCCGACGATCGGTAGCCGGTCTGAGAGGATGAACGGCCACATTGGAACTGAGAC<br>ACGGTCCAGACTCCTACGGGAGGCAGCAGTGGGGAATCTTCCGCAATGGGCGAAAGCCTGACGGAGCAACGCCGCGTGA<br>GTGAAGACGGCCTTCGGGTTGTAAAACTCTGTGATTCGGGACGAAAGATAAGCAGACGAATAATCTGCATAAGTGACGGTAC<br>CGAAAAAGCAAGCCACGGCTAACTACGTGCCAGCAGCCGCGGTAATACGTAGGTGGCAAGCGTTGTCCGGAATTATTGGG<br>CGTAAAGCGCGCGCAGGCGGCTACTTAAGTCCATCTTAAAAGTGCGGGGCTTAACCCCGTGATGGGATGGAAACTGAGAA<br>GCTGGAGTGTCGGAGAGGAAAGTGGAATTCCTAGTGTAGCGGTGAAATGCGTAGAGATTAGGAAGAACACCGGTGGCGAAG<br>GCGACTTTCTGGACGACAACTGACGCTTAGGCGCGAAAGCGTGGGGAGCAAACAGGATTAGATACCCTGGTAGTCCACGC<br>CGTAAACGATGGATACTAGGTGTAGGAGGTATCGACCCCTTCTGTGCCGGAGTTAACGCAATAAGTATCCCGCCTGGGAAG<br>TACGATCGCAAGATTAAAACTCAAAGGAATTGACGGGGGCCCGCACAAGCGGTGGAGTATGTGGTTTAATTCGACGCAACG<br>CGAAGAACCTTACCAGGTCTTGACATTGATCGCTATTTTCAGAAATGAGAAGTTCTCCTTCGGGAGACGAGAAAACAGGTGG<br>TGCACGGCTGTCGTCAGCTCGTGTCGTGAGATGTTGGGTTAAGTCCCGCAACGAGCGCAACCCCTATCATTTGTTGCCAG<br>CACGCAAAGGTGGGAACTCAAATGAGACCGCCGCAGACAATGCGGAGGAAGGCGGGGACGACGTCAAGTCATCATGCCC<br>CTTATGACCTGGGCTACACACGTACTACAATGGGTGTCAACAAAGAGAAGCGAAGGAGCGATCCGGAGCAAACCTCAAAAA<br>CACACCCCCAGTTCAGATCGCAGGCTGCAACTCGCCTGCGTGAAGCAGGAATCGCTAGTAATCGCGGGTCAGCATACCG<br>CGGTGAATACGTTCCCGGGCCTTGTACACACCGCCCGTCACACTATGAGAGTCGGAAACACCCGAAGCCGGTGAGGTAA<br>CCGCAAGGAGCCAGCCGTCGAAGGTGGAGCTGATGATTGGAGTG
92	Lachnoanaerobaculum sp. OTU_175	GATGAACGCTGGCGGCGTGCTTAACACATGCAAGTCGAACGAAGCTGCTTAAAGGAAGTTTTCGGATGGAATTTAGGTAGA<br>CTTAGTGGCGGACGGGTGAGTAACGCGTGGATAACCTGCCTTATACAGGGGGATAACGGAGAGAAATTTCCGCTAACACC<br>GCATAAGACCACAGCACCGCATGGTGCAGGGGTAAAATATTTATAGGTATAAGATGGATCCGCGTCCGATTAGCTTGTTGGT<br>GAGGTAGAGGCTCACCAAGGCGACGATCGGTAGCCGGCCTGAGAGGGTGAACGGCCACATTGGGACTGAGACACGGCC<br>CAAACTCCTACGGGAGGCAGCAGTGGGGAATATTGGACAATGGGGGAAACCCTGATCCAGCGACGCCGCGTGAGTGAAGA<br>AGTATTTCGGTATGTAAAGCTCTATCAGCAGGGAAGAAAATGACGGTACCTGACTAAGAAGCCCCGGCTAACTACGTGCCA<br>GCAGCCGCGGTAATACGTAGGGGGCAAGCGTTATCCGGATTTACTGGGTGTAAAGGGAGCGCAGACGGCAATGCAAGTCT<br>GAAGTGAAAGGCGTGGGCTCAACCCATGAACTGCTTTGGAAACTGTATAGCTTGAGTGTCGGAGGGGTAAGCGGAATTCCT<br>AGTGTAGCGGTGAAATGCGTAGATATTAGGAGGAACACCGGAGGCGAAGGCGGCTTACTGGACGACAACTGACGTTGAGG<br>CTCGAAGGCGTGGGGAGCAAACAGGATTAGATACCCTGGTAGTCCACGCAGTAAACGATGAATACTTGGTGTCGGAGAGGT<br>AAACTCTTCGGTGCCGCAAGCTAACGCATTAAGTATTCCACCTGGGGAGTACGTTCGCAAGAATGAAACTCAAAGGAATTGA<br>CGGGGACCCGCACAAGCGGTGGAGCATGTGGTTTAATTCGAAGCAACGCGAAGAACCTTACCAAATCTTGACATACTCTTG<br>AATAGACTTGTAATGAGTCTAGTCCTTCGGGACAAGGGATACAGGTGGTGCATGGTTGTCGTCAGCTCGTGTCGTGAGATG<br>TTGGGTTAAGTCCCGCAACGAGCGCAACCCTTGTCGTCAGTAGCCAGCAGTAAGATGGGCACTCTGACGAGACAGCCGGA<br>GATAATCCGGAGGAAGGTGGGGATGACGTCAAATCATCATGCCCCTTATGATTTGGGCTACACACGTGCTACAATGGCGTA<br>AACAAAGTGAAGCAAAGCTGTGAAGCCAAGCAAATCACAAAAATAACGTCTCAGTTCGGATTGTAGTCTGCAACTCGACTATA<br>TGAAGCTGGAATCGCTAGTAATCGCAGATCAGAATGCTGCGGTGAATACGTTCCCGGGTCTTGTACACACCGCCCGTCAC<br>ACCATGGGAGTCATCAATGCCCGAAGTCAGTGACCTAACCCACACTGGTAAACATAAAGCTGTGATTGCTTTTTTACTTGG<br>CGAAAGCGAGCCGAAGGCGAAGCTTAAGTCTTAGTAAAAAGTAGTTCTTCATACGAACGAAGTGAGTATGATAGAACGTCC<br>TTAGAAATTATAGCAGTAAGTTGTGTTTAGCAGTGTGGGAAGGAGCTGCCGAAGGCAGGGAGGATAACTGGGGTG

Sr no.	Species_OTUs	16S reference sequence
93	Peptoanaerobacter sp. OTU_178	GATGAACGCTGGCGGCATGCCTAACACATGCAAGTCGAACGGAGTCATATGTGAAGTTTTCGGATGGAATATAAGACTTAGT<br>GGCGGACGGGTGAGTAACACGTAAGTAATCAACCTATAACACACGAATAACTAATAGAAATGTTAGCTAATACGAGATAAAATA<br>TATGATATGGAGATGTTATATATCAAAGCTGAGGCGGTTATAGACGAGCTTGCGTCTGATTAGCTAGTTGGTGGGGTAAGAG<br>CCTACCAAGGCGACGATCAGTAGCCGACCTGAGAGGGTGAACGGCCACATTGGAACTGAGAGACGGTCCAAACTCCTACG<br>GGAGGCAGCAGTGGGGGATATTGCACAATGGGGGGAACCCTGATGCAGCAATGCCGCGTGAACGAAGAAGGCCCTAGGG<br>TCGTAAAGTTCTGTCGTATGGGAAGAAAGAAATGACAGTACCATACAAGAAAGCCCCGGCTAACTACGTGCCAGCAGCCGC<br>GGTAATACGTAGGGGGCAAGCGTTATCCGGAATTACTGGGCGTAAAGGGTGCGTAGGCGGCTTGTTAAGTCAAGGTTAAAA<br>GGCAGCAGCTCAACTGCTGTTCGGCCTTGAAACTAATAAGCTTGAGTATAGGAGAGGAAAGTGGAATTCCCAGTGTAGCGG<br>TGAAATGCGTAGATATTGGGAGGAATACCGGTGGCGAAGGCGACTTTCTGGACTAAAACTGACGCTGAGGCACGAAAGTGT<br>GGGTAGCAAACAGGATTAGATACCCTGGTAGTCCACACCGTAAACGATGAACACTAGGTGTCGGGAGGAATCTCGGTGCC<br>GGCGCAAACGCAATAAGTGTTCCGCCTGGGGAGTACGTTCGCAAGAATAAAACTCAAAGGAATTGACGGGGACCCGCACAA<br>GTAGCGGAGCATGTGGTTTAATTCGAAGCAACGCGAAGAACCTTACCTAAGCTTGACATGGGATTGAAAAGCAATGTAAAGT<br>TGTAATAATACCTTCGGGTATACAATCCACACAGGTGGTGCATGGTTGTCGTCAGCTCGTGTCGTGAGATGTTGGGTTAAGT<br>CCCGCAACGAGCGCAACCCCTATTAATAGTTGCCAGTGCGTAAAGGCAGGCACTCTAATAAGACAGTTAGGGATAACCTAA<br>AGGAAGGTGGGGATGACGTCAAATCATCATGCCCCTTATGCTTAGGGCTACACACGTGCTACAATGGCCGTAACAAAGAGA<br>AGCGAATCTGTGAAGAAGAGCAAACCTGAAAAAAACGGTCCAAGTTCGGATTGTAGGCTGAAACTCGCCTACATGAAGTCGG<br>AGTTACTAGTAATCGCAAATCAGAATGTTGCGGTGAATGCGTTCCCGGGTCTTGTACACACCGCCCGTCACACCATGGGAG<br>CTGAGGGCACCCAAAGTCAGTGATCTAACGCAAGGAGAAGCTGCCTAAGGTGAACTTAGTAACTGGGGTG
94	Alloprevotella sp. OTU_183	GATGAACGCTAGCTACAGGCTTAACACATGCAAGTCGAGGGGCATCGGGTCGAAAGCTTGCTTTCGATGCCGGCGACCGG<br>CGCACGGGTGCGTAACGCGTATCAAACCTACCTTATACTCGGGGATAGCCTTGCGAAAGTAAGATTAATACCCGATGTTGT<br>TGTATTACCGCATGGTAGTTCAACAAAAGATTTTATTGGTATGAGATGGTGATGCGTCCGATTAGGTAGTCGGTGAGGTAAC<br>GGCTCACCGAGCCTTCGATCGGTAGGGGTTCTGAGAGGAAGGTCCCCCACACTGGAACTGAGACACGGTCCAGACTCCT<br>ACGGGAGGCAGCAGTGAGGAATATTGGTCAATGGGCGAGAGCCTGAACCAGCCAAGTAGCGTGCAGGATGACTGCCTTAT<br>GGGTTGTAAACTGCTTTTAGTTGGGAATATAATCAGCCACGTGTGGTTGTTGGAACGTACCATCAGAAAAAGGACCGGCTAA<br>TTCCGTGCCAGCAGCCGCGGTAATACGGAAGGTCCAGGCGTTATCCGGATTTATTGGGTTTAAAGGGAGCGTAGGCGGAT<br>TGTTAAGTCAGCGGTCAAAGGGTGTGGCTCAACCATGCATAGCCGTTGAAACTGGTAATCTTGAGTGCATACAGGGATGCT<br>GGAATTCGTGGTGTAGCGGTGAAATGCTTAGATATCACGAAGAACTCCGATCGCGAAGGCAGGTGTCCGGTCTGTAACTGA<br>CGCTGAGGCTCGAAAGTGTGGGTATCCAACAGGATTAGATACCCTGGTAGTCCACACAGTAAACGATGTATACTCGCGGTT<br>TGCGATATATTGTAAGCCGCCAAGCGAAAGCATTAAGTATACCACCTGGGGAGTACGCCGGCAACGGTGAAACTCAAAGGA<br>ATTGACGGGGGCCCGCACAAGCGGAGGAACATGTGGTTTAATTCGATGATACGCGAGGAACCTTACCCGGGCTTGAACTA<br>ACGGTGACGTATTTAGAGATAGATATTTCTTCGGACACCGTTGGAGGTGCTGCATGGTTGTCGTCAGCTCGTGCCGTGAGG<br>TGTCGGCTTAAGTGCCATAACGAGCGCAACCCTTCTCCTCGGTTGCCATCGGGTAATGCCGGGCACTCCGTGGACACTG<br>CCATCGTAAGATGTGAGGAAGGTGGGGATGACGTCAAATCAGCACGGCCCTTACGTCCGGGGCTACACACGTGTTACAAT<br>GGGGGGTACAGAGGGTTGCTACCTGGCGACAGGATGCTAATCTTTAAAACCTCTCTCAGTTCGGATCGGAGTCTGCAACC<br>CGACTCCGTGAAGCCGGATTCGCTAGTAATCGCGCATCAGCCATGGCGCGGTGAATACGTTCCCGGGCCTTGTACACAC<br>CGCCCGTCAAGCCATGAAAGCCGGGGGTGCCTGAAAGCCGTGACCGCGAGGGTCGGCCTAGGGTAAAACCGGTGATTG<br>GGGCT
95	Lachnoanaerobaculum sp. OTU_184	GATGAACGCTGGCGGCGTGCTTAACACATGCAAGTCGAACGAAGCTGTTAAAAGGAAGTCTTCGGATGAAATTTTAACAGAC<br>TTAGTGGCGGACGGGTGAGTAACGCGTGGATAACCTGCCTTATACAGGGGGATAACGGAGAGAAATTTCCGCTAAGACCG<br>CATAAGACCACAGCACCGCATGGTGCAGGGGTAAAATATTAATAGGTATAAGATGGATCCGCGTCCGATTAGCTAGTTGGTG<br>AGGTAGAGGCTCACCAAGGCGACGATCGGTAGCCGGCCTGAGAGAGTGAACGGCCACATTGGGACTGAGACACGGCCCA<br>AACTCCTACGGGAGGCAGCAGTGGGGAATATTGGACAATGGGGGAAACCCTGATCCAGCGACGCCGCGTGAGTGAAGAAG<br>TATTTCGGTATGTAAAGCTCTATCAGCAGGGAAGAAAATGACGGTACCTGACTAAGAAGCCCCGGCTAACTACGTGCCAGC<br>AGCCGCGGTAATACGTAGGGGGCAAGCGTTATCCGGATTTACTGGGTGTAAAGGGAGCGCAGACGGCCAAGCAAGTCTGA<br>AGTGAAATGCATGGGCTCAACCCATGAATTACTTTGGAAACTGTTAGGCTTGAGTGTCGGAGGGGTAAGCGGAATTCCTAGT<br>GTAGCGGTGAAATGCGTAGATATTAGGAGGAACACCGGAGGCGAAGGCGGCTTACTGGACGACAACTGACGTTGAGGCTC<br>GAAGGCGTGGGGAGCAAACAGGATTAGATACCCTGGTAGTCCACGCAGTAAACGATGAATACTTGGTGTCGGGGAGGTAAA<br>CTCTTCGGTGCCGCAAGCAAACGCATTAAGTATTCCACCTGGGGAGTACGTTCGCAAGAATGAAACTCAAAGGAATTGACG<br>GGGACCCGCACAAGCGGTGGAGCATGTGGTTTAATTCGAAGCAACGCGAAGAACCTTACCAAATCTTGACATACTCTTGAA<br>CAGTCTTGTAATGAGACTAGTCCTTCGGGACAAGGGATACAGGTGGTGCATGGTTGTCGTCAGCTCGTGTCGTGAGATGTT<br>GGGTTAAGTCCCGCAACGAGCGCAACCCTTATCGTCAGTAGCCAGCAGTAAGATGGGAACTCTGACGAGACAGCCGGAGA<br>TAATCCGGAGGAAGGTGGGGATGACGTCAAATCATCATGCCCCTTATGATTTGGGCTACACACGTGCTACAATGGCGTAAA<br>CAAAGTGAAGCAAGACCGCGAGGTTAAGCAAATCACAGAAATAACGTCTCAGTTCGGATTGTAGTCTGCAACTCGACTATAT<br>GAAGCCGGAATCGCTAGTAATCGCAGATCAGAATGCTGCGGTGAATACGTTCCCGGGTCTTGTACACACCGCCCGTCACA<br>CCATGGGAGTCATCAATGCCCGAAGTCAGTGACCTAACCCACACTGGTAAACATAAAGCTGGGATAAGAATACGAGAGAATA<br>AGCCGCCAGGCTTATGAGCGAGTATTCTTATATGACTTAAAGAATTGCAGTAAGAGCAGTTTTAAGTCAGAGTATACAGGATA<br>GATTATGTTTAGCAGTGTGGGAAGGAGCTGCCGAAGGCAGGGAGGATAACTGGGGTG
96	Prevotella sp. OTU_194	GATGAACGCTAGCTACAGGCTTAACACATGCAAGTCGAGGGGCAGCATAAAGATTGCTTGCAATTTTTGATGGCGACCGGC<br>GCACGGGTGAGTAACGCGTATCCAACCTACCTATGACATAGGGATAATCCGGCGAAAGTCGGCCTAATACCTTATATAGTTT<br>ATCGTTGGCATCTTCTATGAACAAAAGATTTATCGGTTATAGATGGGGATGCGTCTGATTAGTTTGTTGGCGGGGTAACGGC<br>CCACCAAGGCATCGATCAGTAGGGGTTCTGAGAGGAAGGTCCCCCACATTGGAACTGAGACACGGTCCAAACTCCTACGG<br>GAGGCAGCAGTGAGGAATATTGGTCAATGGGCGTAAGCCTGAACCAGCCAAGTAGCGTGCAGGACGACTGCCCTATGGGT<br>TGTAAACTGCTTTTATGCGGGGATAAATGATATCACGTGTGATGTTTTGCAGGTACCGCATGAATAAGGACCGGCTAATTCC<br>GTGCCAGCAGCCGCGGTAATACGGAAGGTCCTAGCGTTATCCGGATTTATTGGGTTTAAAGGGAGCGTAGGCCGTTTTATA<br>AGCGTGTTGTGAAATGTACGGGCTCAACCTGTGATTTGCAGCGCGAACTGTATGACTTGAGTACGTTGGAAGTAGGCGGAA<br>TTCGTGGTGTAGCGGTGAAATGCTTAGATATCACGAAGAACTCCGATTGCGAAGGCAGCCTACTGTGTCGTAACTGACGCT<br>GAAGCTCGAAAGTGCGGGTATCGAACAGGATTAGATACCCTGGTAGTCCGCACGGTAAACGATGGATGCTCGCTGTCCGG<br>TCTTTTGGCTGGGTGGCCCAGCGAAAGCGTTAAGCATCCCACCTGGGGAGTACGCCGGCAACGGTGAAACTCAAAGGAAT<br>TGACGGGGGCCCGCACAAGCGGAGGAACATGTGGTTTAATTCGATGATACGCGAGGAACCTTACCCGGGCTTGAATTGCA<br>GAGGAAAGATCCAGAGATGGTGATGCCCTTCGGGGCCTCTGTGAAGGTGCTGCATGGTTGTCGTCAGCTCGTGCCGTGA<br>GGTGTCGGCTTAAGTGCCATAACGAGCGCAACCCCTTTTCTTAGTTGCCATCAGTATTAGCTGGGCACTCTGAGAATACTG<br>CCACCGTAAGGTGTGAGGAAGGTGGGGATGACGTCAAATCAGCACGGCCCTTACGTCCGGGGCTACACACGTGTTACAAT<br>GGCGCATACAGAGAGTCGGTGTTATGCAAATATCATCTAATCTTCAAATTGCGTCTCAGTTCGGACTGGGGTCTGCAACTC<br>GACCCCACGAAGCTGGATTCGCTAGTAATCGCGCATCAGCCATGGCGCGGTGAATACGTTCCCGGGCCTTGTACACACC<br>GCCCGTCAAGCCATGAAAGCTGGGGGTGCCTGAAGTCCGTGACCGCGAGGATCGGCCTAGGGCAAAACCGGTGATTGGG<br>GCT

Sr no.	Species_OTUs	16S reference sequence
97	Lachnospiraceae gen. sp. OTU_211	GATGAACGCTGGCGGCGTGCCTAACACATGCAAGTCGAACGGAGTTGTTTATAGGAAGTTTTCGGATGGAATATAAATAACT<br>TAGTGGCGGACGGGTGAGTAACGCGTGGGTAACCTGCCTTACACAGGGGGATAACAGCCAGAAATGGCTGCTAATACCGC<br>ATAAGCGCACAGAGTCGCATGACACAGTGTGAAAAGATTTATCGGTGTAAGATGGACCCGCGTCTGATTAGCTAGTTGGTAA<br>GGTAAAAGCTTACCAAGGCGACGATCAGTAGCCGGCCTGAGAGGGTGAACGGCCACATTGGGACTGAGACACGGCCCAAA<br>CTCCTACGGGAGGCAGCAGTGGGGAATATTGCACAATGGGGGAAACCCTGATGCAGCGACGCCGCGTGAGTGAAGAAGTA<br>TTTCGGTATGTAAAGCTCTATCAGTAGGGAAGAAAATGACGGTACCTAACTAAGAAGCCCCGGCTAACTACGTGCCAGCAG<br>CCGCGGTAATACGTAGGGGGCAAGCGTTATCCGGATTTACTGGGTGTAAAGGGAGCGTAGGCGGCGATGCAAGTCAGAAG<br>TGAAATTCCAAGGCTCAACCATGGAACTGCTTTTGAAACTGTGTTGCTAGAGTGCGGGAGAGGTAAGTGGAATTCCTAGTGT<br>AGCGGTGAAATGCGTAGATATTAGGAGGAACATCAGTGGCGAAGGCGACTTACTGGACCGATACTGACGCTGAGGCTCGAA<br>AGCGTGGGGAGCAAACAGGATTAGATACCCTGGTAGTCCACGCCGTAAACGATGAATACTAGGTGTCGGGTGGCAGAGCC<br>ATTCGGTGCCGCAGCTAACGCAATAAGTATTCCACCTGGGGAGTACGTTCGCAAGAATGAAACTCAAAGGAATTGACGGGG<br>ACCCGCACAAGCGGTGGAGCATGTGGTTTAATTCGAAGCAACGCGAAGAACCTTACCAAGTCTTGACATCTGACTGACCGG<br>TCCGTAACGGGACCTTCCCTTCGGGGCAGTCAAGACAGGTGGTGCATGGTTGTCGTCAGCTCGTGTCGTGAGATGTTGG<br>GTTAAGTCCCGCAACGAGCGCAACCCTTATCCTTAGTAGCCAGCAAGTAAGGTTGGGCACTCTATGGAGACTGCCGGGGA<br>TAACCCGGAGGAAGGTGGGGATGACGTCAAATCATCATGCCCCTTATGATTTGGGCTACACACGTGCTACAATGGCGTAAA<br>CAAAGGGAAGCGAAAGGGTGACCTGAAGCAAATCCCAAAAATAACGTCTCAGTTCGGATTGTAGTCTGCAACTCGACTACAT<br>GAAGCTGGAATCGCTAGTAATCGCGAATCAGAATGTCGCGGTGAATACGTTCCCGGGTCTTGTACACACCGCCCGTCACA<br>CCATGGGAGTCGGAAATGCCCGAAGTCAGTGACCTAACCCTCATCAGTAAGCATAACGGATCGTGATAATGGAAGGAATGA<br>AATGAGTTCCATTATATTCGAAAGAAAAAATACGATGCCGAGGGCTTAAAAAGCCAGTTGTGCTTAGTGATGAGGGAGGGAG<br>CTGCCGAAGGTGGAGCCGGTAACTGGGGTG
98	Selenomonas sp. OTU_225	GACGAACGCTGGCGGCGTGCTTAACACATGCAAGTCGAACGGGACTAGAAAAGCTTGCTTTGCTAGTCTAGTGGCAAACG<br>GGTGAGTAACACGTAGACAACCTGCCGATCAGATGGGGACAACATTCCGAAAGGAATGCTAATACCGAATGGTGTGCGGGG<br>GAGGCATCTCCCCCGCATGAAAGATGGCCAATACATGTAAGCTATCACTGATCGATGGGTCTGCGTCTGATTAGCTGGTAG<br>GTAAGGTAACGGCTTACCTAGGCGACGATCAGTAGCCGGTCTGAGAGGATGAACGGCCACATTGGGACTGAGACACGGCC<br>CAGACTCCTACGGGAGGCAGCAGTGGGGAATCTTCCGCAATGGGCGCAAGCCTGACGGAGCAACGCCGCGTGAGTGAAG<br>AAGGTCTTCGGATCGTAAAGCTCTGTTGATGGGGACGAACGTGCCTAATGCGAACAGTTTTAGGTAATGACGGTACCTTTTG<br>AGGAAGCCACGGCTAACTACGTGCCAGCAGCCGCGGTAATACGTAGGTGGCGAGCGTTGTCCGGAATCATTGGGCGTAAA<br>GGGAGCGCAGGCGGCCGTGTAAGTCTTGCTTAAAAGTTCGGGGCTCAACCCCGAGATGGGCAAGAAACTATATGGCTTGA<br>GTGCAGGAGAGGAAAGCGGAATTCCCAGTGTAGCGGTGAAATGCGTAGATATTGGGAGGAACACCAGTGGCGAAGGCGGC<br>TTTCTGGACTGCAACTGACGCTGAGGCTCGAAAGCCAGGGGAGCGAACGGGATTAGATACCCCGGTAGTCCTGGCCGTAA<br>ACGATGGATACTAGGTGTGGGAGGTATCGACCCCTACCGTGCCGGAGTTAACGCAATAAGTATCCCGCCTGGGGAGTACG<br>GTCGCAAGACTGAAACTCAAAGGAATTGACGGGGACCCGCACAAGCGGTGGAGTATGTGGTTTAATTCGAAGCAACGCGAA<br>GAACCTTACCAGGCCTTGACATTGATTGAAAGACATAGAGATATGTCCCTCTCTTCGGAGACAAGAAAACAGGTGGTGCATG<br>GCTGTCGTCAGCTCGTGTCGTGAGATGTTGGGTTAAGTCCCGCAACGAGCGCAACCCCTGTCCTTTGTTGCCATCACGTA<br>AAGGTGGGCACTCAAAGGAGACTGCCGCGGAGAACGCGGAGGAAGGCGGGGATGACGTCAAGTCATCATGCCCCTTATG<br>GTCTGGGCTACACACGTACTACAATGGAGTGGACAGAGAGCAGCAAGAGAGCGATCTGGAGCGAACCTCAAAAACCACTTC<br>CCAGTTCGGATTGCAGGCTGCAACTCGCCTGCATGAAGTCGGAATCGCTAGTAATCGCAGGTCAGCATACTGCGGTGAAT<br>ACGTTCCCGGGTCTTGTACACACCGCCCGTCACACCACGGAAGTCGTTCACACCCGAAGCCGGCGCAGCCGTCTAAGGT<br>GGGGAAGGTGACTGGGGTG
99	Prevotella sp. OTU_242	GATGAACGCTAGCTACAGGCTTAACACATGCAAGTCGAGGGGAAACGGCATTTGGTGCTTGCACCGGATGGACGTCGACC<br>GGCGCACGGGTGAGTAACACGTATCCAACCTTCCCATGACTGCGGGATAACCTGCCGAAAGGCTGACTAATACCGCATGT<br>TCTTCCCTGAAGACATCTGAGGGGAAGCAAAGATTCATCGGTCATGGCTGGGGATGCGTCCGATTAGCTTGTTGGCGGGG<br>CAACGGCCCACCAAGGCGACGATCGGTAGGGGTTCTGAGAGGAAGGTCCCCCACATTGGAACTGAGACACGGTCCAAAC<br>TCCTACGGGAGGCAGCAGTGAGGAATATTGGTCAATGGGCGCAAGCCTGAACCAGCCAAGTAGCGTGCAGGATGACGGC<br>CCTATGGGTTGTAAACTGCTTTTTTGCGGGAATAAAGTCGGCTACGTGTAGCCGTTTGCATGTACCGCACGAATAAGAACC<br>GGCTAATTCCGTGCCAGCAGCCGCGGTAATACGGAAGGTTCGGGCGTTATCCGGATTTATTGGGTTTAAAGGGAGCGTAG<br>GCGGCAGGTTAAGCGTGTTGTGAAATGCAGTTGCTCAACATCTGAATTGCAGCGCGAACTGTCCTGCTTGAGTGCGCACAA<br>CGTAGGCGGAATTCGTCGTGTAGCGGTGAAATGCTTAGATATGACGAAGAACCCCGATTGCGAAGGCAGCCTTCGGGAGC<br>GCAACTGACGCTGAAGCTCGAAAGTGCGGGTATCGAACAGGATTAGATACCCTGGTAGTCCGCACGGTAAACTATGGACG<br>CTCGCCGTTGGTGCCTGGCATCTGCGGCTCAGCGAAAGCGTTAAGCGTCCCACCTGGGGAGTACGCCGGCAACGGTGA<br>AACTCAAAGGAATTGACGGGGGCCCGCACAAGCGGAGGAACATGTGGTTTAATTCGATGATACGCGAGGAACCTTACCCG<br>GGCTTGAATTGCAGAAGACAGTTACAGAGATGTATCTTTCCTTCGGGACTTCTGTGAAGGTGCTGCATGGTTGTCGTCAGC<br>TCGTGCCGTGAGGTGTCGGCTTAAGTGCCATAACGAGCGCAACCCCTCTCCCCAGTTGCCATCGGGTGATGCCGGGCAC<br>TCCGGGGACACTGCCGCCGCAAGGTGTGAGGAAGGTGGGGATGACGTCAAATCAGCACGGCCCTTACGTCCGGGGCTA<br>CACACGTGTTACAATGGCCGGTACAGAGGGTCGGTGTATCGCAAGGTGCATCCAATCTTGAAATCCGGTCTCAGTTCGGA<br>CTGGGGTCTGCAACCCGACCCCACGAAGCTGGATTCGCTAGTAATCGCGCATCAGCCATGGCGCGGTGAATACGTTCCC<br>GGGCCTTGTACACACCGCCCGTCAAGCCATGAAAGCCGGGGGTGCCTGAAGTCCGTGACCGCAAGGGTCGGCCTAGGG<br>CAAGACCGGTGATTGGGGCT
100	Prevotella sp. OTU_243	GATGAACGCTGGCTACAGGCTTAACACATGCAAGTCGGGGGGAAACGGGAGAACAGCTTGCTGTTCTTTGTCGTCGACCG<br>GCGCACGGGTGAGTAACGCGTATCCAACCTTCCCATAACTAAGGGATACCCCGTAGAAATGCGGCCTAATACCTTATGTTC<br>TCCCGGCCTGACATCAGAACGGGAGCAAAGATTCATCGGTTATGGATGGGGATGCGTCTGATTAGCCTGCTGGCGGGGTA<br>ACGGCCCACCAGGGCATCGATCAGTAGGGGTTCTGAGAGGAAGGTCCCCCACATTGGAACTGAGACACGGTCCAAACTC<br>CTACGGGAGGCAGCAGTGAGGAATATTGGTCAATGGGCGGAAGCCTGAACCAGCCAAGTAGCGTGCAGGATGACGGCCC<br>TATGGGTTGTAAACTGCTTTTGAAGGGGAATAAAGAAGGGCACGTGTGCCCTGTTGCATGTACCCTTGGAATAAGGACCGG<br>CTAATTCCGTGCCAGCAGCCGCGGTAATACGGAAGGTCCTGGTGTTATCCGGATTTATTGGGTTTAAAGGGAGCGTAGGC<br>CGCATATTAAGTGTGTTGTGAAAAGCAGTCGCCCAACGTCTGTCTTGCAGCGCAAACTGGTGTGCTTGAGTACGCACAACG<br>CAGGCGGAATTCGTCGTGTAGCGGTGAAATGCTTAGATATGACGAAGAACCCCGATTGCGAAGGCAGCCTGCGGGAGCGT<br>TACTGACGCTGAAGCTCGAAAGTGCGGGTATCGAACAGGATTAGATACCCTGGTAGTCCGCACGGTAAACGATGGATGCC<br>CGTTTTTGGCTTATGAAGTCAGAGACCAAGCGAAAGCATTAAGCATCCCACCTGGGGAGTACGCCGGCAACGGTGAAACTC<br>AAAGGAATTGACGGGGGCCCGCACAAGCGGAGGAACATGTGGTTTAATTCGATGATACGCGAGGAACCTTACCCGGGCTT<br>GAATTGCCAGAGACGGCTCCGGAGACGGAGCTTCCCTTCGGGGTTCTGGTGAAGGTGCTGCATGGTTGTCGTCAGCTCG<br>TGCCGTGAGGTGTCGGCTTAAGTGCCATAACGAGCGCAACCCCTTTCTTCAGTTGCCATCGGGTAATGCCGGGCACTCTG<br>TAGATACTGCCGCCGCAAGGTGTGAGGAAGGTGGGGATGACGTCAAATCAGCACGGCCCTTACGTCCGGGGCTACACAC<br>GTGTTACAATGGGGGGTACAGCGAGCCGGATGCCCGCAAGGTCATTCCAATCATGAAATCCTCCCTCAGTTCGGACTGGG<br>GTCTGCAACCCGACCCCACGAAGCTGGATTCGCTAGTAATCGCGCATCAGCCATGGCGCGGTGAATACGTTCCCGGGCC<br>TTGTACACACCGCCCGTCAAGCCATGAAAGCCGGGGGTGCCTGAAGTCCGTGACCGCAAGGATCGGCCTAGGGCAAAAC<br>CGGTAATTGGGGCT

Sr no.	Species_OTUs	16S reference sequence
101	Capnocytophaga sp. OTU_249	GATGAACGCTAGCGGCAGGCCTAACACATGCAAGTCGAGGGGGAGGTTACTTTCGGGTAACTGAGACCGGCGTACGGGT<br>GCGTAACGCGTATACAATCTGCCTTTCACTGGGAGATAGCCCGAAGAAATTTGGATTAATACCCCATAGTATAGGAGCACGG<br>CATCGTGATACTATTAAAGCTTAGGTGGTGAAAGATGAGTATGCGTTCTATTAGCTAGTTGGAGAGGTAACGGCTCCCCAAG<br>GCGATGATAGATAGGGGTTCTGAGAGGGATGTCCCCCACACTGGTACTGAGATACGGACCAGACTCCTACGGGAGGCAGC<br>AGTGAGGAATATTGGACAATGGTCGGAAGACTGATCCAGCCATGCCGCGTGCAGGATGAAGGTCTTATGGATTGTAAACTG<br>CTTTTGTAAGGGAAGAATAAGGGCTACGCGTAGTTTGATGACGGTACCTTACGAATAAGCATCGGCTAACTCCGTGCCAGC<br>AGCCGCGGTAATACGGAGGATGCGAGCGTTATCCGGAATCATTGGGTTTAAAGGGTCTGTAGGCGGGCTAATAAGTCAGAG<br>GTGAAAGCGCTCAGCTCAACTAAGCAACTGCCTTTGAAACTGTTGGTCTTGAATGGTTGTGAAGTAGTTGGAATGTGTAGTG<br>TAGCGGTGAAATGCTTAGATATTACACAGAACACCGATAGCGAAGGCATATTACTAACAATTAATTGACGCTGATGGACGAAA<br>GCGTGGGGAGCGAACAGGATTAGATACCCTGGTAGTCCACGCTGTAAACGATGGATACTAGCTGTTTGGTTTAAGGATTGA<br>GTGGCTAAGCGAAAGTGATAAGTATCCCACCTGGGGAGTACGTTCGCAAGAATGAAACTCAAAGGAATTGACGGGGGCCCG<br>CACAAGCGGTGGAGCATGTGGTTTAATTCGATGATACGCGAGGAACCTTACCAAGGTTTAAATGGAGACTGACAGGTGTAGA<br>AATATGCCCTTCTTCGGACAGTTTTCAAGGTGCTGCATGGTTGTCGTCAGCTCGTGCCGTGAGGTGTCAGGTTAAGTCCTA<br>TAACGAGCGCAACCCCTGCCATTAGTTGCTAACGAGTTAAGTCGAGCCCTCTAATGGGACTGCCGGTGCAAACCGAGAGG<br>AAGGTGGGGATGACGTCAAATCATCACGGCCCTTACATCTTGGGCTACACACGTGCTACAATGGCCGTTACAGAGAGCAG<br>CCACTGCGTGAGCAGGCGCGAATCTATAAAGACGGTCACAGTTCGGATCGGAGTCTGCAACTCGACTCCGTGAAGCTGGA<br>ATCGCTAGTAATCGGATATCAGCCATGATCCGGTGAATACGTTCCCGGGCCTTGTACACACCGCCCGTCAAGCCATGGAA<br>GCTGGGAGTACCTGAAGACGGTTACCTAAAGGAGCTGTTTAGGGTAAAACTAGTGACTGGGGCT
102	Limosilactobacillus sp. OTU_259	GATGAACGCCGGCGGTGTGCCTAATACATGCAAGTCGAACGCACTGGCCCAACTGAGATGACGTGCTTGCACTGATTTGA<br>CGATGGATTACCAGTGAGTGGCGGACGGGTGAGTAACACGTAGGCAACCTGCCCAAGAGCGGGGGATAACATTTGGAAAC<br>AAGTGCTAATACCGCATAAGATTGATGACCACATGGTCATCAATTTAAAGATGGCTTCGGCTATCACTCTTGGATGGGCCTG<br>CGGTGCATTAGCTAGTTGGTAAGGTAACGGCTTACCAAGGCAGTGATGCATAGCCGAGTTGAGAGACTGATCGGCCACAAT<br>GGGACTGAGACACGGCCCATACTCCTACGGGAGGCAGCAGTAGGGAATCTTCCACAATGGGCGCAAGCCTGATGGAGCG<br>ACACCGCGTGAGTGAAGAAGGGTTTCGGCTCGTAAAGCTCTGTTGTTGAAGAAGAACGTGCGTGAGAGTAACTGTTCACGC<br>AGTGACGGTATTCAACCAGAAAGTCACGGCTAACTACGTGCCAGCAGCCGCGGTAATACGTAGGTGGCAAGCGTTATCCG<br>GATTTATTGGGCGTAAAGCGAGCGCAGGCGGTTTTCTAAGTCTGATGTGAAAGCCTTCGGCTTAACCGAAGAAGGGCATCG<br>GAAACTGGATGACTTGAGTGCAGAAGAGGGCAGTGGAACTCCATGTGTAGCGGTGGAATGCGTAGATATATGGAAGAACAC<br>CAGTGGCGAAGGCGGCTGCCTAGTCTGCAACTGACGCTGAGGCTCGAAAGCATGGGTAGCGAACAGGATTAGATACCCTG<br>GTAGTCCATGCCGTAAACGATGAGTGCTAGGTGTTGGAGGGTTTCCGCCCTTCAGTGCCGCAGCTAACGCAATAAGCACT<br>CCGCCTGGGGAGTACGACCGCAAGGTTGAAACTCAAAGGAATTGACGGGGGCCCGCACAAGCGGTGGAGCATGTGGTTT<br>AATTCGAAGCTACGCGAAGAACCTTACCAGGTCTTGACATCTTGCGCTAACCTAAGAGATTAGGCGTTCCCTTCGGGGACG<br>CAATGACAGGTGGTGCATGGTCGTCGTCAGCTCGTGTCGTGAGATGTTGGGTTAAGTCCCGCAACGAGCGCAACCCTTGT<br>TACTAGTTGCCAGCATTCAGTTGGGCACTCTAGTGAGACTGCCGGTGACAAACCGGAGGAAGGTGGGGACGACGTCAGAT<br>CATCATGCCCCTTATGACCTGGGCTACACACGTGCTACAATGGACGGTACAACGAGCAGCGAACTCGCGAGGGCAAGCAA<br>ATCTCTTAAAACCGTTCTCAGTTCGGACTGTAGGCTGCAACTCGCCTACACGAAGTCGGAATCGCTAGTAATCGCGGATCA<br>GCATGCCGCGGTGAATACGTTCCCGGGCCTTGTACACACCGCCCGTCACACCATGGAAGTTTGCAATGCCCAAAGTCGG<br>TGGGCTAACCTTCGGGAAGCAGCCGCCTAAGGCAGGGCAGATGACTGGGGTG
103	Prevotella sp. OTU_269	GATGAACGCTGGCTACAGGCCTAACACATGCAAGTCGTGGGGCAGCATGGGGGTTGCTTGCAATCCCTGATGGCGACCG<br>GCGCACGGGTGAGTAACGCGTATCCAACCTGCCTGTCACAAGGGAATAACCCGTCGAAAGTCGGCCTAATGCCCTATACA<br>TTCCGATGAAGGCTTCTGAATCGGAAGAAAGCTGCGGCGGTGACAGATGGGGATGCGTCCGATTAGCTTGTCGGTGAGGT<br>AACGGCCCACCGAGGCAACGATCGGTAGGGGTTCTGAGAGGAAGGTCCCCCACATTGGAACTGAGACACGGTCCAAACT<br>CCTACGGGAGGCAGCAGTGAGGAATATTGGTCAATGGGCGCTAGCCTGAACCAGCCAAGTAGCGTGCAGGATGACGGCC<br>CTATGGGTTGTAAACTGCTTTTATACGGGGATAAAGTGGGGAACGTGTTTCTCCATTGCAGGTACCGTATGAATAAGGACCG<br>GCTAATTCCGTGCCAGCAGCCGCGGTAATACGGAAGGTCCGGGCGTTATCCGGATTTATTGGGTTTAAAGGGAGCGCAGG<br>CCGCACCTTAAGCGTGTTGTGAAATGTACCGGCTCAACCGGTAACGTGCAGCGCGAACTGGGGTGCTTGAGTACGAAGAG<br>GGAAGGCGGAACTCGTGGTGTAGCGGTGAAATGCTTAGATATCACGAGGAACTCCGATCGCGAAGGCAGCTTTCCGTTTC<br>GTCACTGACGCTGAGGCTCGAAAGTGCGGGTATCGAACAGGATTAGATACCCTGGTAGTCCGCACGGTAAACGATGGATG<br>CTCGCTGTTGGGCCATTTGGTTCAGCGGCTAAGTGAAAACGTTAAGCATCCCACCTGGGGAGTACGCCGGCAACGGTGAA<br>ACTCAAAGGAATTGACGGGGGCCCGCACAAGCGGAGGAACATGTGGTTTAATTCGATGATACGCGAGGAACCTTACCCGG<br>GCTTGAATTGCAGACGAACGATCCAGAGATGGTGAGGCCCTTCGGGGCGTCTGTGAAGGTGCTGCATGGTTGTCGTCAGC<br>TCGTGCCGTGAGGTGTCGGCTTAAGTGCCATAACGAGCGCAACCCCTCTCCGCAGTTGCCATCGGGTAATGCCGGGCAC<br>TCTGCGGACACTGCCTCCGCAAGGAGTGAGGAAGGTGGGGATGACGTCAAATCAGCACGGCCCTTACGTCCGGGGCTAC<br>ACACGTGTTACAATGGCGCGTACAGAGGGCCGGTTCTGTGCAAACAGGATCGAATCTATAAAACGCGTCCCAGTTCGGAC<br>CGGGGTCTGCAACCCGACCCCGCGAAGCTGGATTCGCTAGTAATCGCGCATCAGCCATGGCGCGGTGAATACGTTCCCG<br>GGCCTTGTACACACCGCCCGTCAAGCCATGAAAGCCGGGGGCGCCTGAAGTCCGTGACCGCAAGGATCGGCCTAGGGC<br>GAGACCGGTGATTGGGGCT
104	Fretibacterium sp. OTU_282	GATGAACGCTGGCGGCGTGCGTAACACATGCAAGTTGGACGACGGTGCCATGAAGTGGTGACACGGAGTGGTATACGGAG<br>TAGCGGACGGGTGAGTAATACATGAGGAGCTGTCCTGTTGAGGGGGATAACGTACGGAAACGGACCCTAATACCCCATAG<br>GCCGAGAGGTTAAAGGCAGAGATGCGCGACAGGAGGTACTCGTGTCCTATCAGCTAGTTGGTGAGGTAAGGGCTCACCAA<br>GGCGACGACGGGTAGCCGGCCTGAGAGGGTGACCGGCCACACTGGAACTGAGATACGGTCCAGACTCCTACGGGAGGC<br>AGCAGTGGGGAATATTGGGCAATGGGAGGAATCCTGACCCAGCGACGCCGCGTGAACGAAGACGGCCTTCGGGTTGTAAA<br>GTTCTTTTATGTGGGAAGAAGGAAGTGACGGTACCACATGAATAAGCCCCGGCTAACTACGTGCCAGCAGCCGCGGTAATA<br>CGTAGGGGGCGAGCGTTGTCCGGAATTACTGGGCGTAAAGGGCACGCAGGCTGTGCTTCAAGTCAGCTGTAAAAGGATGC<br>GGCTTAACCGTGTTATGCAGTTGAGACTGAGGTGCTGGAGTACTGGAGAGGCAAGTGGAATTCCCAGTGTAGCGGTGAAAT<br>GCGTAGATATTGGGAAGAACATCGGTGGCGAAGGCGACTTGCTGGACAGTAACTGACGCTGAGGTGCGAAAGCCAGGGTA<br>GCGAACGGGATTAGATACCCCGGTAGTCCTGGCTGTAAACGATGAATGCTAGGTGTGCGAGCAGAGATGTTGGTGTGCCG<br>CAGTTAACGCGATAAGCATTCCGCCTGGGGAGTACGGTCGCAAGATTGAAACTCAAAGGAATTGACGGGGGCCCGCACAA<br>GCGGTGGAGCACGTGGTTTAATTCGATGCAAACCGAAGAACCTTACCTGGGTTTGACATGTATGTGGTAGGAGATGGAAAC<br>ATTGACGACGCTGCTTTCGGGTAGTGAGCATACACAGGTGCTGCATGGCTGTCGTCAGCTCGTGTCGTGAGATGTTGGGT<br>TAAGTCCCGCAACGAGCGCAACCCCTGCGATTAGTTGCCAACGGGTAGAGCCGGGCACTCTAATGGGACTGCCATCGAC<br>AAGATGGAGGAAGGTGGGGATGACGTCAAGTCATCATGGCCCTTATGTCCAGGGCGACACACGTGCTACAATGGCCGGTA<br>CAGAGGGAAGCGAGGCTGAGAGGTTGAGCGGATCCCGAAAAGCCGGTCTCAGTTCGGATTGAAGTCTGCAATTCGACTTC<br>ATGAAGCTGGAATCGCTAGTAATCGCAGATCAGCCAAGCTGCGGTGAATACGTTCCCGGGCCTTGTACACACCGCCCGTC<br>ACACCACCCGAGCCGGGTGTACCCGAAGCCGGTGGCCTAACCTTAGGGGAGGAGCCGTCGAAGGTGTGTCTGGTGAGG<br>GGGGTG

Sr no.	Species_OTUs	16S reference sequence
105	Porphyromonas sp. OTU_283	GATGAACGCTAGCGATAGGCTTAACACATGCAAGTCGAGGGGCAGCGCGAGGTAGCAATACTTTGGCGGCGACCGGCGC<br>ACGGGTGCGTAACACGTATGTAACTTACCTCTTACAATCGGATAACACGGTGAAAGTCGTGCTAATACGGTATAGTTTCGCA<br>AGCTCGCATGGGTATGCGAATAAAGATTTATCGGTAAGAGATAGACATGCGGTCCATTAGATAGTTGGTGAGGTAACGGCTC<br>ACCAAGTCGACGATGGATAGGGGGACTGAGAGGTTGACCCCCCACACTGGTACTGAGACACGGACCAGACTCCTACGGG<br>AGGCAGCAGTGAGGAATATTGGTCAATGGGCGAGAGCCTGAACCAGCCAAGTCGCGTGAAGGAAGACGGTTCATAAGGATT<br>GTAAACTTCTTTTGCAGAGGAGTAACGATAGGCACGTGTGCCTGTGTGAACGTACTCTGCGAATAAGTATCGGCTAACTCC<br>GTGCCAGCAGCCGCGGTAATACGGAGGATACGAGCGTTATCCGGAATTATTGGGTTTAAAGGGTGCGTAGGTGGCTATTTA<br>AGTCAGGGGTGAAAAGCATGAGCTCAACTCATGTCTTGCCTTTGAAACTGGACAGCTAGAGAAAAGACGAGGTATGTGGAAT<br>GTGTGGTGTAGCGGTGAAATGCATAGATATCACACAGAACTCCGATTGCGCAGGCAGCGTACCAGACTTCTTCTGACACTG<br>AAGCACGAAAGCGTGGGGATCAAACAGGATTAGATACCCTGGTAGTCCACGCAGTAAACGATGAATACTAGTTTTTTGCGAT<br>ATAATGTAAGAGACTAAGCGAAAGCGATAAGTATTCCACCTGGGGAGTACGCCGGCAACGGTGAAACTCAAAGGAATTGACG<br>GGGGCCCGCACAAGCGGAGGAACATGTGGTTTAATTCGATGATACGCGAGGAACCTTACCCGGGATTGAAATGTAGGAGTT<br>AGATGATGAAAGTCATTGTCCCTTCGGGGCTCCTATGTAGGTGCTGCATGGTTGTCGTCAGCTCGTGCCGTGAGGTGTTT<br>GCTTAAGTGCAATAACGAGCGCAACCCGCGTTGATAGTTACTAACAGGTAAAGCTGAGGACTCTATCGAGACAGCCGTCGT<br>AAGACGAGAGGAAGGTGCGGATGACGTCAAATCAGCACGGCCCTTACATCCGGGGCGACACACGTGTTACAATGGCAGGG<br>ACAAAAGGAAGCGACATAGCGATATGAAGCAGATCCCAAAACCCTGCCCCAGTTCGGATCGGAGTCTGCAACTCGACTCC<br>GTGAAGCCGGATTCGCTAGTAATCGCGCATCAGCCATGGCGCGGTGAATACGTTCCCGGGCCTTGTACACACCGCCCGT<br>CAAGCCATGGGAGTCGGGGGTACCTGAAGAGCGTGACCGTCACAGGAGCGCTTAAGGGTAATACCGGTAACTGGGGCT
106	Saccharibacteria sp. OTU_288	GATGAATGCTGGCGGCGTGCCTAATACATGCAAGTCGAGCGGTAACAGGGTTTCACCGTGTTCTTGACACTCCGGGCTTA<br>TAGCGGGAGTTAGTTTTTCGAGAGCACCGTGAAGCTGCTGACGAGCGGCGGACGGCTGAGTAACGCGTGGGAACATACC<br>CCAAATTGAGGGATAACTACTCGAAAGAGTAGCTAATACCGCATGTGATCTTTGGATTAAAGCTTCGGCGATTTGGGAATGG<br>CCTGCGTACGATTAGATAGTTGGTGAGGTAAAGGCTCACCAAGTCGACGATCGTTAGATGGTTTGAGAGGATGATCATCCA<br>GACTGGGACTGAGACACGGCCCAGACTCCTACGGGAGGCAGCAGTAGGGAATCTTTCACAATGGGCGAAAGCCTGATGGA<br>GCAACGCCGCGTGCAGGATGAAGGCCTTCGGGTTGTAAACTGCTTTTATAAGCGAGAAATATGATGGTAACTTATGAATAAG<br>GATCGGCTAACTACGTGCCAGCAGCCGCGGTCATACGTAGGATCCGAGCATTATCCGGAGTGACTGGGTGTAAAGAGTTG<br>CGTAGGTGGCATAGTAAGTAGATAGTGAAATCTGGTGGCTCAACCATTCAGACTATTATCTAAACTGCTAAGCTCGAGACCG<br>TTAGGGGTAACTGGAATTTCTAGTGTAGGAGTGAAATCCGTAGATATTAGAAGGAACACCGATAGCGTAGGCAGGTTACTGG<br>GACGGTTCTGACACTAAGGCACGAAAGCGTAGGGAGCAAACGGGATTAGATACCCCGGTAGTCTACGCCGTAAACGATGG<br>ATACTAGCAGTTTTGGGTATCGACCCCCGGAGTTGCGAAGCTAACGCGTTAAGTATCCCACCTGTGTAGTACGATCGCAAG<br>ATTAAAACATAAAGGAATTGACGGGGACCCGCACAAGCGGTGGAGCGTGATCTTTAATTCGATGGTAAACGATAAACCTTAC<br>CAAGGCTTGACATCCGATGAAGGCTCTCGAAAGAGAACTGTGCCTTTTAGGAACATCGAGACAGGTGATGCATGGCCGTCG<br>TCAGCTCGTGTCGTGAGATGTTTGGTTAAGTCCATCAACGAGCGCAACCCTTGCAACCAGTTGTATTTTTCTGGTAGGACT<br>GCCCCGGTAACGGGGAGGAAGGAGGGGATGATGTCAGGTCAGTATTTCCCTTACGCCTTGGGCTAGAATCGCGCTACAAT<br>GGCCGGTACAATGCGAAGCGAAGCAGTGATGTGAAGCAAATCGCACCAAAGCCGGTCCCAGTTCGGATAGAAGGCTGAAA<br>CTCGCCTTCTTGAAGTCGGAATCGCTAGTAATCGCAGATCAGCATGTTGCGGTGAATACGTTCCCGGGTCTTGTACACACC<br>GCCCGTCAAACCATGAGAGTCACCAACACCCGAAGTCCGTCTAGGCGGCCTAAGGTGGGGGAGATGATTGGGGTT
107	Rothia sp. OTU_289	GACGAACGCTGGCGGCGTGCTTAACACATGCAAGTCGAACGATGAAGACTGGTGCTTGCACTGGTTGGATTAGTGGCGAA<br>CGGGTGAGTAATACGTGAGTAACCTGCCTTTGACTCTGGGATAAGCCTGGGAAACTGGGTCTAATACCGGATATGACAAGG<br>AACCGCATGGTTTTTTGTGGAAAGGGTTTGTACTGGTTTTAGATGGGCTCACGGCCTATCAGCTTGTTGGTGGGGTAATGG<br>CTCACCAAGGCGACGACGGGTAGCCGGCCTGAGAGGGTGACCGGCCACACTGGGACTGAGACACGGCCCAGACTCCTA<br>CGGGAGGCAGCAGTGGGGAATATTGCACAATGGGCGCAAGCCTGATGCAGCGACGCCGCGTGAGGGATGACGGCCTTC<br>GGGTTGTAAACCTCTTTCAGCAGGGAAGAAGCGAAAGTGACGGTACCTGCAGAAGAAGCGCCGGCTAACTACGTGCCAGC<br>AGCCGCGGTAATACGTAGGGCGCGAGCGTTGTCCGGAATTATTGGGCGTAAAGAGCTTGTAGGCGGTTTGTTGCGTCTGC<br>TGTGAAAGACCGGGGCTTAACCCCGGTATTGCAGTGGGTACGGGCAGACTAGAGTGCAGTAGGGGAGACTGGAATTCCTG<br>GTGTAGCGGTGAAATGCGCAGATATCAGGAGGAACACCGATGGCGAAGGCAGGTCTCTGGGCTGTAACTGACGCTGAGAA<br>GCGAAAGCATGGGGAGCGAACAGGATTAGATACCCTGGTAGTCCATGCCGTAAACGTTGGGCACTAGGTGTGGGGGACAT<br>TCCACGTTTTCCGCGCCGTAGCTAACGCATTAAGTGCCCCGCCTGGGGAGTACGGCCGCAAGGCTAAAACTCAAAGGAAT<br>TGACGGGGGCCCGCACAAGCGGCGGAGCATGCGGATTAATTCGATGCAACGCGAAGAACCTTACCAAGGCTTGACATATA<br>CTGGACCGCCTCAGAGATGGGGTTTCCCTTCGGGGCTGGTATACAGGTGGTGCATGGTTGTCGTCAGCTCGTGTCGTGA<br>GATGTTGGGTTAAGTCCCGCAACGAGCGCAACCCTCGTTCTATGTTGCCAGCACGTTATGGTGGGGACTCATAGGAGACT<br>GCCGGGGTCAACTCGGAGGAAGGTGGGGATGACGTCAAATCATCATGCCCCTTATGTCTTGGGCTTCACGCATGCTACAA<br>TGGCCGGTACAAAGGGTTGCGATACTGTGAGGTTGAGCTAATCCCAAAAAGCCGGTCTCAGTTCGGATTGGGGTCTGCAA<br>CTCGACCCCATGAAGTCGGAGTCGCTAGTAATCGCAGATCAGCAACGCTGCGGTGAATACGTTCCCGGGCCTTGTACACA<br>CCGCCCGTCAAGTCACGAAAGTTGGTAACACCCGAAGCCGATGGCCTAACCCTTTTGGGAGGGAGTCGTCGAAGGTGGG<br>ATTGGCGATTGGGACT
108	Lachnoanaerobaculum sp. OTU_292	GATGAACGCTGGCGGCGTGCTTAACACATGCAAGTCGAACGAAGCTGTTAAAAGGAAGTCTTCGGATGGAATTTTAACAGAC<br>TTAGTGGCGGACGGGTGAGTAACGCGTGGATAACCTGCCTTATACAGGGGGATAACGGAGAGAAATTTCCGCTAAGACCG<br>CATAAGACCACAGCACCGCATGGTGCAGGGGTAAAATATTAATAGGTATAAGATGGATCCGCGTCCGATTAGCTTGTTGGTG<br>AGGTAGAGGCTCACCAAGGCGACGATCGGTAGCCGGCCTGAGAGGGTGAACGGCCACATTGGGACTGAGACACGGCCCA<br>AACTCCTACGGGAGGCAGCAGTGGGGAATATTGGACAATGGGGGAAACCCTGATCCAGCGACGCCGCGTGAGTGAAGAAG<br>TATTTCGGTATGTAAAGCTCTATCAGCAGGGAAGAAAATGACGGTACCTGACTAAGAAGCCCCGGCTAACTACGTGCCAGC<br>AGCCGCGGTAATACGTAGGGGGCAAGCGTTATCCGGATTTACTGGGTGTAAAGGGAGCGCAGACGGCCAAGCAAGTCTGA<br>AGTGAAATGCATGGGCTCAACCCATGAATTGCTTTGGAAACTGTTAGGCTTGAGTGTCGGAGGGGTAAGCGGAATTCCTAG<br>TGTAGCGGTGAAATGCGTAGATATTAGGAGGAACACCGGAGGCGAAGGCGGCTTACTGGACGACAACTGACGTTGAGGCT<br>CGAAGGCGTGGGGAGCAAACAGGATTAGATACCCTGGTAGTCCACGCAGTAAACGATGAATACTTGGTGTCGGGGAGATAA<br>ACTCATCGGTGCCGCAAGCTAACGCATTAAGTATTCCACCTGGGGAGTACGTTCGCAAGAATGAAACTCAAAGGAATTGAC<br>GGGGACCCGCACAAGCGGTGGAGCATGTGGTTTAATTCGAAGCAACGCGAAGAACCTTACCAAATCTTGACATACTCTTGA<br>ACAGCTTTGTAATGAAGTTGGACCTTCGGGACAAGGGATACAGGTGGTGCATGGTTGTCGTCAGCTCGTGTCGTGAGATGT<br>TGGGTTAAGTCCCGCAACGAGCGCAACCCTTGTCGTCAGTAGCCAGCAGTAAGATGGGAACTCTGACGAGACAGCCGGAG<br>ATAATCCGGAGGAAGGTGGGGATGACGTCAAATCATCATGCCCCTTATGATTTGGGCTACACACGTGCTACAATGGCGTAA<br>ACAAAGTGAAGCAAGACCGTGAGGTTAAGCAAATCACAGAAATAACGTCTCAGTTCGGATTGTAGTCTGCAACTCGACTATAT<br>GAAGCCGGAATCGCTAGTAATCGCAGATCAGAATGCTGCGGTGAATACGTTCCCGGGTCTTGTACACACCGCCCGTCACA<br>CCATGGGAGTCATCAATGCCCGAAGTCAGTGACCTAACCCACACTGGTAAACATAAAGCTGGGATAAGAATACGAGAGAATA<br>AGCCGCCAGGCTTATGAGCGAGTATTCTTATATGATATAAAGAATAGCAGTAAGTTATGTTTAGCAGTGTGGGAAGGAGCTG<br>CCGAAGGCAGGGAGGATAACTGGGGTG

Sr no.	Species_OTUs	16S reference sequence
109	Saccharibacteria sp. OTU_302	GATGAATGCTGGCGGCGTGCCTAATACATGCAAGTCGAGCGGTAACAGGGTTTCACCGTTTTCTTGTGGCAGGATTTGTTTTTGACACACTTGGCCCAGATGGCAAGAACGTAGTCTAGAAAAATGAATTTTGATACCTACAAGGGAACAGTGAAACTGCTGACGAGCGGCGGACGGCTGAGTAACGCGTGGGAACAGACCCCAAACTGAGGGATAACTACTCGAAAGAGTGGCTAATACCGCATGTGATCTTCGGATTAAAGGATTTATCCGGTTTGGGACTGGCCTGCGTAGGATTAGATAGTTGGTGAGGTAATGGCTCACCAAGTCGACGATCCTTAGATGGTTTGAGAGGATGATCATCCAGACTGGGACTGAGACACGGCCCAGACTCCTACGGGAGGCAGCAGTAGGGAATCTTTCACAATGGGCGAAAGCCTGATGGAGCAACGCCGCGTGCAGGATGAAGGCCTTCGGGTTGTAAACTGCTTTTAAAGCCGAGAAATATGATGGTAAGCTTTGAATAAGGACCGGCTAACTACGTGCCAGCAGCCGCGGTCATACGTAGGGTCCAAGCATTATCCGGAGTGACTGGGTGTAAAGAGTTGCGTAGGTGGCATAATAAGTAGCTAGTGAAATCTGGTGGCTCAACCATTCAGACTATTAGCTAAACTGTTAAGCTCGAGACCGTTAGGGGTAACTGGAATTTCTAGTGTAGGAGTGAAATCCGTAGATATTAGAAGGAACACCGATAGCGTAGGCAGGTTACTGGGGCGGTTCTGACACTAAGGCACGAAAGCGTAGGGAGCAAACGGGATTAGATACCCCGGTAGTCTACGCCGTAAACGATGGATACTAGCAGTTTGGGGTATCGACCCCCTGAGTTGCGAAGCTAACGCGTTAAGTATCCCACCTGTGTAGTACGATCGCAAGATTAAAACATAAAGGAATTGACGGGGACCCGCACAAGCGGTGGAGCGTGATCTTTAATTCGATGGTAAACGATAAACCTTACCAAGGCTTGACATCTCGGGAAGGCTTCCGAAAGGAAACTGTGCCCTCGGGAACCCGAAGACAGGTGATGCATGGCCGTCGTCAGCTCGTGTCGTGAGATGTTTGGTTAAGTCCATCAACGAGCGCAACCCTTGCAAGCAGTTGTATTTTTCTGCTAGGACTGCCCCGGTAACGGGGAGGAAGGAGGGGATGATGTCAGGTCAGTATTTCCCTTACGCCTTGGGCTAGAATCGCGCTACAATGGCCGGTACAATGCGAAGCGAAGCAGTGATGTGAAGCAAATCGCAGCAAAGCCGGTCCCAGTTCGGATAGAAGGCTGAAACTCGCCTTCTTGAAGTCGGAATCGCTAGTAATCGCAGATCAGCATGTTGCGGTGAATACGTTCCCGGGTCTTGTACACACCGCCCGTCAAACCATGAGAGTCACCAACACCCGAAGTCCGCTCAAGCGGCCTAAGGTGGGGGAGATGATTGGGGTT
110	Peptostreptococcus sp. OTU_303	GATGAACGCTGGCGGCGTGCCTAACACATGCAAGTCGAGCGAGGGCTTACTCGGAATTGAGTATTCTATCAGCGTTTTTTCGTTTCTGACCCTAGTAGTCAGATTGGAAGAAACGAAACACGCACTATAAAGTGCCAGTTCGCACTATAAAGTGCCGGTAAAGTAGAGTATTGAATTCTGAGTAAGACCAAGCGGCGGACGGGTGAGTAACGCGTGGGTAACCTGCCCTATACACATGGATAACATACTGAAAAGTTTACTAATACATGATAATATATATTTACGGCATCGTAGATATATCAAAGTGTTAGCGGTATAGGATGGACCCGCGTCTGATTAGCTAGTTGGTGAGATAACTGCCCACCAAGGCGACGATCAGTAGCCGACCTGAGAGGGTGATCGGCCACATTGGAACTGAGACACGGTCCAAACTCCTACGGGAGGCAGCAGTGGGGAATATTGCACAATGGGCGCAAGCCTGATGCAGCAACGCCGCGTGAACGATGAAGGTCTTCGGATCGTAAAGTTCTGTTGCAGGGGAAGATAATGACGGTACCCTGTGAGGAAGCCCCGGCTAACTACGTGCCAGCAGCCGCGGTAATACGTAGGGGGCTAGCGTTATCCGGATTTACTGGGCGTAAAGGGTGCGTAGGTGGTCTTTCAAGTCGGTGGTTAAAGGCTACGGCTCAACCGTAGTTAGCCTCCGAAACTGGAAGACTTGAGTGCAGGAGAGGAAAGTGGAATTCCCAGTGTAGCGGTGAAATGCGTAGATATTGGGAGGAACACCAGTAGCGAAGGCGGCTTTCTGGACTGCAACTGACACTGAGGCACGAAAGCGTGGGTAGCAAACAGGATTAGATACCCTGGTAGTCCACGCTGTAAACGATGAGTACTAGGTGTCGGGGGTTACCCCCCTCGGTGCCGCAGCTAACGCATTAAGTACTCCGCCTGGGGAGTACGCACGCAAGTGTGAAACTCAAAGGAATTGACGGGGACCCGCACAAGTAGCGGAGCATGTGGTTTAATTCGAAGCAACGCGAAGAACCTTACCTAAGCTTGACATCCCTTAGACCGGTGTTTAATCACACCTTCCCTTCGGGGCTGAGGTGACAGGTGGTGCATGGTTGTCGTCAGCTCGTGTCGTGAGATGTTGGGTTAAGTCCCGCAACGAGCGCAACCCTTGTCTTTAGTTGCCAGCATTCAGTTGGGCACTCTAGAGAGACTGCCAGGGATAACCTGGAGGAAGGTGGGGATGACGTCAAATCATCATGCCCCTTATGCTTAGGGCTACACACGTGCTACAATGGGTGGTACAGAGGGTTGCCAAACCGTGAGGTGGAGCTAATCCCTTAAAGCCATTCTCAGTTCGGATTGTAGGCTGAAACTCGCCTACATGAAGCTGGAGTTACTAGTAATCGCAGATCAGAATGCTGCGGTGAATGCGTTCCCGGGTCTTGTACACACCGCCCGTCACACCATGGGAGTCGGAAACACCCGAAGCCGATTATCCAACCGCAAGGAGGAAGTCGTCGAAGGTGGCGTCGATAACTGGGGTG
111	Schaalia sp. OTU_304	GACGAACGCTGGCGGCGTGCTTAACACATGCAAGTCGAACGGGCCTGCTCTGGTTTTTCTGGGGTGGGTGAGTGGCGAACGGGTGAGTAACACGTGAGTAACCTGCCCTCTTCTTGGGGATAACGGTCTGAAAGGGCTGCTAATACCGGATGTTCACTGTCTGTCGCATGGTGGGTGGTGGAAAGAGGCACTGTTGTGGTGTTTTGGTGGGGGATGGGCTCGCGGCTTATCAGCTTGTTGGTGGGGTGATGGCTTACCAAGGCTTTGACGGGTAGCCGGCCTGAGAGGGTGACCGGTCACATTGGGACTGAGATACGGCCCAGACTCCTGCGGGAGGCAGCAGTGGGGAATTTTGCACAATGGACGGAAGTCTGATGCAGCGACGCCGCGTGAGGGATGGAGGCCTTCGGGTTGTAAACCTTTGTTGCCCTTGACCAAGGTGATACGTGGGTATTGTTGAGGGTAGGGGGTGAGGAAGCGCCGGCTAACTACGTGCCAGCAGCCGCGGTAATACGTAGGGCGCGAGCGTTGTCCGGAATTATTGGGCGTAAAGGGCTTGTAGGCGGTTGATTGCGTCTGCCGTGAAATCCTCTGGCTTAACTGGGGGCGTGCGGTGGGTACGGGTTGGCTTGAGTGCGGTAGGGGAGGCTGGAATTCCTGGTGTAGCGGTGGAATGCGCAGATATCAGGAGGAACACCGGTGGCGAAGGCGGGTCTCTGGGCCGTTACTGACGCTGAGGAGCGAAAGCGTGGGGAGCGAACAGGATTAGATACCCTGGTAGTCCATGCTGTAAACGTTGGGCACTAGGTGTGGGGGCCACCCGTGGTTTCTGCGCCGTAGCTAACGCTTTAAGTGCCCCGCCTGGGGAGTACGGCCGCAAGGCTAAAACTCAAAGGAATTGACGGGGGCCCGCACAAGCGGCGGAGCATGCGGATTAATTCGATGCAACGCGAAGAACCTTACCAAGGCTTGACATGCGCCCAGAGCCTTCAGAGATGGGGGTGCTTTTGGTTGGGGGTGTGCAGGTGGTGCATGGTTGTCGTCAGCTCGTGTCGTGAGATGTTGGGTTAAGTCCCGCAACGAGCGCAACCCTTGCCCTATGTTGCCAGCGCGTTGTGGTGGGGACTCGTGGGGGACTGCCGGGGTTAACTCGGAGGAAGGTGGGGATGACGTCAAATCATCATGCCCCTTATGTCTTGGGCTTCACGCATGCTACAATGGCTGGTACAGAGGGTTGCGATACTGTGAGGTGGAGCGAATCCCTTAAAGCTGGTCTCAGTTCGGATCGGAGTCTGCAACTCGACTTCGTGAAGGTGGAGTCGCTAGTAATCGCAGATCAGCAGTGCTGCGGTGAATACGTTCTCGGGCCTTGTACACACCGCCCGTCACGTCACGAAAGTTGGTAACGCCCGAAGCTCATGGCCTAACCGTGTGGGGGGAGTGGGTGACGGTGGGATTGGCGATTGGGACG
112	Streptococcus sp. OTU_315	GACGAACGCTGGCGGCGTGCCTAATACATGCAAGTAGAACGCTGAAGCTTGGTGCTTGCACCGAGCGGAAGAGTTGCGAACGGGTGAGTAACGCGTAGGTAACCTGCCTGGTAGCGGGGGATAACTATTGGAAACGATAGCTAATACCGCATGATATTGATTATTGCATGATAGTCAATTAAAAGGTGCAATTGCATCACTACCAGATGGACCTGCGTTGTATTAGCTAGTTGGTGGGGTAACGGCTCACCAAGGCGACGATACATAGCCGACCTGAGAGGGTGATCGGCCACACTGGGACTGAGACACGGCCCAGACTCCTACGGGAGGCAGCAGTAGGGAATCTTCGGCAATGGACGGAAGTCTGACCGAGCAACGCCGCGTGAGTGAAGAAGGTTTTCGGATCGTAAAGCTCTGTTGTAAGAGAAGAACGAGTGTGAGAGTGGAAAGTTCACACTGTGACGGTATCTTACCAGAAAGGGACGGCTAACTACGTGCCAGCAGCCGCGGTAATACGTAGGTCCCGAGCGTTGTCCGGATTTATTGGGCGTAAAGCGAGCGCAGGCGGTTAGATAAGTCTGAAGTTAAAGGCTGTGGCTTAACCATAGTACGCTTTGGAAACTGTTTAACTTGAGTGCAAGAGGGGAGAGTGGAATTCCATGTGTAGCGGTGAAATGCGTAGATATATGGAGGAACACCGGTGGCGAAAGCGGCTCTCTGGCTTGTAACTGACGCTGAGGCTCGAAAGCGTGGGGAGCAAACAGGATTAGATACCCTGGTAGTCCACGCCGTAAACGATGAGTGCTAGGTGTTAGACCCTTTCCGGGGTTTAGTGCCGCAGCTAACGCATTAAGCACTCCGCCTGGGGAGTACGACCGCAAGGTTGAAACTCAAAGGAATTGACGGGGGCCCGCACAAGCGGTGGAGCATGTGGTTTAATTCGAAGCTACGCGAAGAACCTTACCAGGTCTTGACATCTTGCGCCAACCCTAGAGATAGGGCGTTTCCTTCGGGAACGCAATGACAGGTGGTGCATGGTCGTCGTCAGCTCGTGTCGTGAGATGTTGGGTTAAGTCCCGCAACGAGCGCAACCCTTGTTACTAGTTGCCAGCATTAAGTTGGGCACTCTAGTGAGACTGCCGGTGACAAACCGGAGGAAGGTGGGGACGACGTCAGATCATCATGCCCCTTATGACCTGGGCTACACACGTGCTACAATGGACGGTACAACGAGTCGCGAACTCGCGAGGGCAAGCAAATCTCTTAAAACCGTTCTCAGTTCGGACTGCAGGCTGCAACTCGCCTGCACGAAGTCGGAATCGCTAGTAATCGCGGATCAGCATGCCGCGGTGAATACGTTCCCGGGCCTTGTACACACCGCCCGTCACACCATGAGAGTTTGTAACACCCAAAGTCGGTGGGGTAACCTTTTAGGAGCCAGCCGCCTAAGGTGGGACAGATGATTAGGGTG

Sr no.	Species_OTUs	16S reference sequence
113	Kingella sp. OTU_325	ATTGAACGCTGGCGGCATGCTTTACACATGCAAGTCGAACGGCAGCACGAAAGAGCTTGCTCTTTTGGTGGCGAGTGGCG<br>AACGGGTGAGTAAGATATTGGAACGTACCGAGTAATGGGGGATAACTATCCGAAAGGATAGCTAATACCGCATACGCTTTGA<br>GAAGGAAAGTGGGGGATCTTCGGACCTCACGTTATTCGAGCGGCCAATAACTGATTAGCTAGTTGGAGGGGTAAAGGCCCA<br>CCAAGGCGACGATCAGTAGCGGGTCTGAGAGGATGATCCGCCACACTGGGACTGAGACACGGCCCAGACTCCTACGGGA<br>GGCAGCAGTGGGGAATTTTGGACAATGGGCGCAAGCCTGATCCAGCCATGCCGCGTGTCTGAAGAAGGCCTTCGGGTTG<br>TAAAGGACTTTTGTTAGGGAAGAAAAGGAGTATGTTAATACCATACTCTGCTGACGGTACCTAAAGAATAAGCACCGGCTAAC<br>TACGTGCCAGCAGCCGCGGTAATACGTAGGGTGCGAGCGTTAATCGGAATTACTGGGCGTAAAGCGAGCGCAGACGGTTT<br>ATTAAGCAAGATGTGAAATCCCCGAGCTTAACTTGGGAACTGCGTTTTGAACTGGTAAGCTAGAGTATGTCAGAGGGGGGTA<br>GAATTCCACGTGTAGCAGTGAAATGCGTAGAGATGTGGAGGAATACCGATGGCGAAGGCAGCCCCCTGGGATAATACTGAC<br>GTTCATGCTCGAAAGCGTGGGTAGCAAACAGGATTAGATACCCTGGTAGTCCACGCCCTAAACGATGTCAATTAGCTGTTG<br>GGGCACTTGATGCCTTAGTAGCGAAGCTAACGCGTGAAATTGACCGCCTGGGGAGTACGGTCGCAAGATTAAAACTCAAAG<br>GAATTGACGGGGACCCGCACAAGCGGTGGATGATGTGGATTAATTCGATGCAACGCGAAGAACCTTACCTGGTCTTGACAT<br>GTACGGAATGACTCAGAGACGAGTTAGTGCCTTCGGGAACCGTAACACAGGTGCTGCATGGCTGTCGTCAGCTCGTGTCG<br>TGAGATGTTGGGTTAAGTCCCGCAACGAGCGCAACCCTTGTCATTAGTTGCCACCATTCAGTTGGGCACTCTAATGAGACT<br>GCCGGTGACAAACCGGAGGAAGGTGGGGATGACGTCAAGTCCTCATGGCCCTTATGACCAGGGCTTCACACGTCATACAA<br>TGGTCGGTACAGAGGGTAGCCAAGCCGCGAGGTGGAGCCAATCTCACAAAACCGATCGTAGTCCGGATTGCACTCTGCAA<br>CTCGAGTGCATGAAGTCGGAATCGCTAGTAATCGCAGGTCAGCATACTGCGGTGAATACGTTCCCGGGTCTTGTACACAC<br>CGCCCGTCACACCATGGGAGTGGGGGATACCAGAAGTAGGTAGGCTAACCGCAAGGAGGCCGCTTACCACGGTATGTTT<br>CATGACTGGGGTG
114	Anaerocella sp. OTU_337	GATGAACGCTAGCGGTAGGCTTAACACATGCAAGTCGAGGGGCATCATGAGCGGTAGAGATACTGTTTGATGGCGACCGG<br>CGGATGGGTGAGTAACGCGTATGCAACCTACCTTTTACATTGGGATAGACACTGGAAACGGTGCGTAAACTCGGATGATATT<br>TGGCATAGGCATCTATGTTAGATTAAATGTATTATTACAGGTAAGAGATGGGCATGCGTAGGATTAGCTAGTTGGTGTGGTAA<br>TGGCGCACCAAGGCATCGATCCTTAGGGGTTCTGAGAGGAAGGTCCCCCACATTGGAACTGAGAACGGTCCAAACTCCTA<br>CGGGAGGCAGCAGTGAGGAATATTGGTCAATGGACGGAAGTCTGAACCAGCCATACCGCGTGCAGGAAGACGGTCTTATG<br>GATTGTAAACTGCTTTTTTGCTAGGGTACAGACCCTGTCGAGCACAGGGGGAAGAAAGTATAGTAAGAATAAGCATCGGCTA<br>ACTCCGTGCCAGCAGCCGCGGTAATACGGAGGATGCGAGCGTTATCCGGATTTATTGGGTTTAAAGGGTGCGTAGGCTGT<br>TAAGTAGGTCTATGGTGAAATCGTTGTGCTTAACACAACTGAGCTGTGGAAACCGTTTAACTAGAATTTAAGTGGCGTAGGC<br>GGAATGAATCATGTAGAGGTGACATTCATAGATATGATTCAGAACACCGTTCGCGAAGGCGGCTTACGAAGGTAAAATTGAC<br>GCTAATGCACGAAAGCGTGGGTATCGAACAGGATTAGATACCCTGGTAGTCCACGCTGTAAACGATGATAACTATATGTCGG<br>CGATATAATGTCGGTGTAGCAGTGAAAGCGATAAGTTATCCACCTGGGGAGTACGATCGCAAGGTTGAAACTCAAAGGAATT<br>GACGGGGGCCCGCACAAGCGGAGGAGCATGTGGTTTAATTCGATGATACGCGAGGAACCTTACCTGGACTTGAACGTGTA<br>ATTAATATACAAGAGATTGTATAGTATAGCAATATAAATTATGCGAGGTGCTGCATGGTTGTCGTCAGCTCGTGCCGTGAGGT<br>GTCAGGTTAAGTCCTATAACGAGCGCAACCCTTATCGTTAGTTACCAGCGATTATAGTCGGGGACTCTAGCGATACTGCCG<br>GCGTAAGCCGAGAGGAAGGTGGGGATGACGTCAAATCAGCACGGCCCTTACGTCCAGGGCGACACACGTGCTACAATGG<br>TCGGTACAGAGGGCAGTTACTTAGTGATAAGATACGAATCTCTAAAGCCGGTCTCAGTACGGATTGGAGTCTGCAACCCGA<br>CTCCATGAAGTTGGATTCGCTAGTAATCGCAGATCAGCCATGCTGCGGTGAATACGTTCCCGGGCCTTGTACACACCGCC<br>CGTCAAGCCATGGAAGTTGGTTGTACCTAAAGAGCGTAACCGTTGAGGAGCGTTTTAGGGTAAAGCCAGTAACTGGGGCT
115	Corynebacterium sp. OTU_346	GACGAACGCTGGCGGCGTGCTTAACACATGCAAGTCGAACGGAAAGGCCCTGCTTGCAGGGTACTCGAGTGGCGAACGG<br>GTGAGTAACACGTGGGTGATCTGCCCTGCACTTCGGGATAAGCCTGGGAAACTGGGTCTAATACCGGATAGGAGCCATTTT<br>TAGTGTGATGGTTGGAAAGTTTTTTCGGTGTAGGATGAGCTCGCGGCCTATCAGCTTGTTGGTGGGGTAATGGCCTACCAA<br>GGCGGCGACGGGTAGCCGGCCTGAGAGGGTGGACGGCCACATTGGGACTGAGATACGGCCCAGACTCCTACGGGAGGC<br>AGCAGTGGGGAATATTGCACAATGGGCGCAAGCCTGATGCAGCGACGCCGCGTGGGGGATGACGGCCTTCGGGTTGTAA<br>ACTCCTTTCGCTAGGGACGAAGCTTTTTGTGACGGTACCTAGATAAGAAGCACCGGCTAACTACGTGCCAGCAGCCGCGG<br>TAATACGTAGGGTGCGAGCGTTGTCCGGAATTACTGGGCGTAAAGGGCTCGTAGGTGGTTTGTCGCGTCGTCTGTGAAATT<br>CCGGGGCTTAACTCCGGGCGTGCAGGCGATACGGGCATAACTTGAGTACTGTAGGGGTAACTGGAATTCCTGGTGTAGCG<br>GTGAAATGCGCAGATATCAGGAGGAACACCGATGGCGAAGGCAGGTTACTGGGCAGTTACTGACGCTGAGGAGCGAAAGC<br>ATGGGTAGCGAACAGGATTAGATACCCTGGTAGTCCATGCCGTAAACGGTGGGCGCTAGGTGTGAGGGTCTTTTCACGAC<br>TTTCGTGCCGTAGCTAACGCATTAAGCGCCCCGCCTGGGGAGTACGGCCGCAAGGCTAAAACTCAAAGGAATTGACGGGG<br>GCCCGCACAAGCGGCGGAGCATGTGGATTAATTCGATGCAACGCGAAGAACCTTACCTGGGCTTGACATACACTGGATCG<br>GGCTAGAGATAGTCTTTCCCTTTGTGGCTGGTGTACAGGTGGTGCATGGTTGTCGTCAGCTCGTGTCGTGAGATGTTGGG<br>TTAAGTCCCGCAACGAGCGCAACCCTTGTCTTATGTTGCCAGCATTTGGTTGGGGACTCATGAGAGACTGCCGGGGTCAA<br>CTCGGAGGAAGGTGGGGATGACGTCAAATCATCATGCCCCTTATGTCCAGGGCTTCACACATGCTACAATGGTCGGTACAA<br>CGCGCAGCGACACTGTGAGGTGGAGCGAATCGCTGAAAGCCGGCCTTAGTTCGGATTGGGGTCTGCAACTCGACCCCAT<br>GAAGTCGGAGTCGCTAGTAATCGCAGATCAGCAATGCTGCGGTGAATACGTTCCCGGGCCTTGTACACACCGCCCGTCAC<br>GTCATGAAAGTTGGTAACACCCGAAGCCGGTGGCCCAAACTTGTTAGGGAGCCGTCGAAGGTGGGATCGGCGATTGGGAC<br>G
116	Treponema sp. OTU_357	AACGAACGCTGGCGGCGCGTCTTAAACATGCAAGTCGAACGGCAAGGGAGAGCTTGCTCTCCCCTAGAGTGGCGGACTG<br>GTGAGTAACACGTGGGTGACATACCTTTTAGTTGGGGATAGCTATTAGAAATAGTAGGTAATACCGAATGTGACCGTACCTG<br>TTAGAGGGGTACGAGGAAAGGAGCTAAGGCTTTGCTAAAAGAATGGCTCGCGGCCCATTATGCTAGTTGGTGAGGTAACGG<br>CCCACCAAGGCGATGATGGGTATCCGGCCTGAGAGGGTGAACGGACACATTGGGACTGAGATACGGCCCAGACTTCTAC<br>GGGAGGCAGCAGTTAAGAATATTCCGCAATGGACGCAAGTCTGACGGAGCGACGCCGCGTGGATGAAGAATGCCGAAAGG<br>TTGTAAAATCCTTTTAAGCCTGATGAATAAGCACAATAGGGAATGATTGTGCGGTGACCGTAGGGCTTGAATAAGCACCGGC<br>TAATTACGTGCCAGCAGCCGCGGTAACACGTAAGGTGCGAGCGTTGTTCGGAATTATTGGGCGTAAAGGGCATGTAGGCG<br>GATAGGCAAGCTTGGTGTGAAATGTTACAGCTTAACTGTGAGACAGCATTGAGAACTGCCGATCTTGAATTACTGAAGGGTA<br>ACCAGAATTCCACGTGTAGGGGTGAAATCTGTAGATATGTGGAAGAATACCAATGGCGAAGGCAGGTTACCGGCAGATAATT<br>GACGCTGAGGTGCGAAAGTGCGGGGAGCGAACAGGATTAGATACCCTGGTAGTCCGCACCGTAAACGATGTACACTAGGT<br>GTCTGGCATAACAGCTGGGTGCCAAAGCAAACGTGATAAGTGTACCGCCTGGGGAGTATGCCCGCAAGGGTGAAACTCAA<br>AGGAATTGACGGGGGCCCGCACAAGCGGTGGAGCATGTGGTTTAATTCGATGGTACGCGAGGAACCTTACCTGGGTTTGA<br>CATAGTATCTGATGCCGTAGAGATACGGCAGCGTAGCAATACGAGGTACAACAGGTGCTGCATGGCTGTCGTCAGCTCGT<br>GCCGTGAGGTGTTGGGTTAAGTCCCGCAACGAGCGCAACCCCTACTGCCAGTTACTAACAGGTAAAGCTGAGGACTCTGG<br>CGGAACTGCCGGTGACAAACCGGAGGAAGGTGGGGATGACGTCAAGTCATCATGGCCCTTATGTCCAGGGCTACACACGT<br>GCTACAATGGTAGGAACAAAGTGAAGCGAAGCTGCAAAGCGGAGCAAAACGCAAAAAAGCTATCGTAGTTCGGATTGGAGTC<br>TGAAACTCGACTCCATGAAGTTGGAATCGCTAGTAATCGCATATCAGCACGATGCGGTGAATACGTTCCCGGGCCTTGTAC<br>ACACCGCCCGTCACACCATCCGAGTAGAGGGTACCCGAAGTCGGTAGTCTAACCGCAAGGAGGACGCTGCCGAAGGTAT<br>GCTTTGTAAGGAGGGTG

Sr no.	Species_OTUs	16S reference sequence
117	Erysipelotrichaceae gen. sp. OTU_388	GATGAACGCTGGCGGCGTGCCTAATACATGCAAGTCGAACGCAGCCAAGAGAAGCTTGCTTCTGTTGACTGAGTGGCGAA<br>CGGGTGAGTAATACATAAGTAATCTGCCCCTAAGACTGGGATAACCGTTGGAAACGATGGCTAATACCGGATAGGTAAAGAC<br>CAGACCTCTGGACTTTATTAAAGATGGGATACATCGCGCAGGGATGAGCTTATGGCGCATTAGTTAGTTGGCGGGGTAAAG<br>GCCCACCAAGACGATGATGCGTAGCCGACCTGAGAGGGTGACCGGCCACACTGGGACTGAGACACGGCCCAGACTCCTA<br>CGGGAGGCAGCAGTAGGGAATTTTCGGCAATGGGGGAAACCCTGACCGAGCAACGCCGCGTGAGTGAAGAAGGCCTTCG<br>GGTTGTAAAGCTCTGTTGTAAGGGAAGAACGGTCGCTGGAGGGAATGCCAGCGAAGTGACGGTACCTTACCAGCAAGTCA<br>CGGCTAACTACGTGCCAGCAGCCGCGGTAATACGTAGGTGGCAAGCGTTATCCGGAATTATTGGGCGTAAAGGGAGCGCA<br>GGCGGCGCATTAAGTCTATGGTGAAAGTTCGAAGCTCAACTTCGAATTGCCCTAGAAACTGGTGTGCTCGAGTGCAGGAGA<br>GGTGAGTGGAATTCCATGTGTAGCGGTAAAATGCGTAGATATATGGAGGAACACCAGTGGCGAAGGCGGCTCACTGGCCT<br>GTAACTGACGCTGAGGCTCGAAAGCGTGGGGAGCAAATAGGATTAGATACCCTAGTAGTCCACGCTGTAAACGATGATGAC<br>TAAGTGTTGGGGAAACTCAGTGCTGCAGTTAACGCAATAAGTCATCCGCCTGGGGAGTATGCACGCAAGTGTAAAACTCAAA<br>GGAATTGACGGGGGCCCGCACAAGCGGTGGAGTATGTGGTTTAATTCGACGCAACGCGAAGAACCTTACCAAGTCTTGAC<br>AGCTCATTAAAGGCTCTAGAGATAGAGAGATAGTTATAGTGAGTACAGGTGGTGCATGGTTGTCGTCAGCTCGTGTCGTGAG<br>ATGTTGGGTTAAGTCCCGCAACGAGCGCAACCCTTGTCTTTAGTTACCAGCATTAAGTTGGGGACTCTAAAGAGACTGCCG<br>GTGACAAACCGGAGGAAGGTGGGGATGAGGTCAAATCATCATGCCCCTTATGACTTGGGCTACACACGTCCTACAATGGC<br>GACTACAAAGAGCAGCAAGACAGTGATGTTGAGCGAATCTCACAAAGGTCGTCTCAGTTCGGATTGAAGTCTGCAACTCGA<br>CTTCATGAAGCTGGAATCGCTAGTAATCGCAGATCAGCATGCTGCGGTGAATACGTTCTCGGGCCTTGTACACACCGCAC<br>GTCAAACCATGAGAGTTGGCAATACCCGAAGCCGGTGGCCTAACCTTTTAGGAGGGAGCCGTCGAAGGTAGGGCTGATGA<br>TTGGGGTT
118	Veillonellaceae gen. sp. OTU_391	GACGAACGCTGGCAGCGTGCTTAACACATGCAAGTCGAACGAAAGGAAGAGAAAAGCTTGCTTTTTCTGAATTGAGTGGCA<br>AACGGGTGAGTAACACGTAAACAACCTGCCTTCAGGATGGGGACAACAGACGGAAACGACTGCTAATACCGAATACGTTCC<br>ACAGAGCGCATGCTTTATGGAAGAAAGGGTGGCCTCTACCTGTAAGCTATCGCCTGAAGAGGGGTTTGCGTCTGATTAGGC<br>AGTTGGCGGGGTAACGGCCCACCAAACCGACGATCAGTAGCCGGTCTGAGAGGATGAACGGCCACACTGGAACTGAGAC<br>ACGGTCCAGACTCCTACGGGAGGCAGCAGTGGGGAATCTTCCGCAATGGGCGAAAGCCTGACGGAGCAACGCCGCGTGA<br>GTGAAGACGGCCTTCGGGTTGTAAAGCTCTGTGATCCGGGACGAAAGAGCGCCATACGAAGAATATGGCGAAGTGACGGT<br>ACCGTAGAAGAAAGCCACGGCTAACTACGTGCCAGCAGCCGCGGTAATACGTAGGTGGCAAGCGTTGTCCGGAATTATTG<br>GGCGTAAAGGGCGCGCAGGCGGCCTCTTAAGTCCATCTTAAAAGAGCGGGGCTTAACCCCGTTATGGGATGGAAACTGAG<br>AAGCTGGAGTATCGGAGAGGAAAGTGGAATTTCTAGTGTAGCGGTGAAATGCGTAGAGATTAGGAAGAACACCGGTGGCGA<br>AGGCGGCTTTCTGGACGAAAACTGACGCTGAGGCGCGAAAGCCAGGGGAGCGAACGGGATTAGATACCCCGGTAGTCCT<br>GGCCGTAAACGATGGGTACTAGGTGTGGGAGGTATCGACCCCTTCTGTGCCGGAGTTAACGCAATAAGTACCCCGCCTGG<br>GGAGTACGGCCGCAAGGTTGAAACTCAAAGGAATTGACGGGGGCCCGCACAAGCGGTGGAGTATGTGGTTTAATTCGACG<br>CAACGCGAAGAACCTTACCAAGCCTTGACATTGATCGCAAGGAGTAGAGATACTCCGTTCTTCTTCGGAAGACGAGAAAACA<br>GGTGGTGCACGGCTGTCGTCAGCTCGTGTCGTGAGATGTTGGGTTAAGTCCCGCAACGAGCGCAACCCCTATCTTCTGTT<br>ACCAGCACGTAAAGGTGGGGACTCAGGAGAGACTGCCGCAGACAATGCGGAGGAAGGCGGGGATGACGTCAAGTCATCAT<br>GCCCCTTATGGCTTGGGCTACACACGTACTACAATGGCTCTTAATAGAGGGAAGCGAAGGAGCGATCCGGAGCAAACCCC<br>AAAAACAGAGTCTCAGTTCGGATTGTAGGCTGCAACTCGCCTACATGAAGCAGGAATCGCTAGTAATCGCAGGTCAGCATA<br>CTGCGGTGAATACGTTCCCGGGCCTTGTACACACCGCCCGTCACACCACGAAAGTCATTCACACCCGAAGCCGGTGAGG<br>TAACCGCAAGGAGCCAGCCGTCGAAGGTGGGGGCGATGATTGGGGTG
119	Oribacterium sp. OTU_394	GATGAACGCTGGCGGCGTGCCTAACACATGCAAGTCGAACGAGATGCTTATTGGAAAGCTTCGGCCGGAAGATAAGTTATC<br>TAGTGGCGGACGGGTGAGTAACACGTGGGTAACCTGCCTTATGGAGGGGGATAACAGAGAGAAATCACTGCTAATACCGCA<br>TAAGCACACAGTACCGCATGGTACAGGGTGAAAAGATTTATCGCCATAAGATGGACCCGCGTCTGATTAGCCAGTTGGCAG<br>GGTAAAAGCCTACCAAAGCAACGATCAGTAGCCGATCTGAGAGGATGACCGGCCACATTGGGACTGAGACACGGCCCAAA<br>CTCCTACGGGAGGCAGCAGTGGGGAATATTGCACAATGGGGGAAACCCTGATGCAGCGACGCCGCGTGAGTGAAGAAGTA<br>TTTCGGTATGTAAAGCTCTATCAGCAGGGAAGATAATGACAGTACCTGACTAAGAAGCCCCGGCTAACTACGTGCCAGCAG<br>CCGCGGTAATACGTAGGGGGCAAGCGTTATCCGGATTTACTGGGTGTAAAGGGAGCGTAGACGGAATGGCAAGTCTGAAG<br>TGAAATACCCGGGCTCAACCTGGGAACTGCTTTGGAAACTGTTGTTCTAGAGTGTTGGAGAGGTAAGTGGAATTCCTGGTG<br>TAGCGGTGAAATGCGTAGATATCAGGAAGAACACCGGAGGCGAAGGCGGCTTACTGGACAATAACTGACGTTGAGGCTCGA<br>AAGCGTGGGGATCAAACAGGATTAGATACCCTGGTAGTCCACGCTGTAAACGATGAATACTAGGTGTCGGGGTGCAAAGCA<br>TCTCGGTGCCGTCGCTAACGCAATAAGTATTCCACCTGGGGAGTACGTTCGCAAGAATGAAACTCAAAGGAATTGACGGGG<br>ACCCGCACAAGCGGTGGAGCATGTGGTTTAATTCGAAGCAACGCGAAGAACCTTACCAAGTCTTGAGATCCCATTGACAGA<br>GTATGTAATGTACTTTCCCTTCGGGGCAATGGTGACAGGTGGTGCATGGTTGTCGTCAGCTCGTGTCGTGAGATGTTGGGT<br>TAAGTCCCGCAACGAGCGCAACCCCTATAGTTAGTAGCCAGCAGTAAGATGGGCACTCTAACTAGACTGCCAGGGATAACC<br>TGGAGGAAGGCGGGGATGACGTCAAATCATCATGCCCCTTATGACTTGGGCTACACACGTGCTACAATGGCGTAAACAAAG<br>GGAAGCAAGAGGGTGACCTTAAGCAAATCTCAAAAATAACGTCTCAGTTCGGACTGTAGTCTGCAACCCGACTACACGAAG<br>CTGGAATCGCTAGTAATCGCAGATCAGCATGCTGCGGTGAATACGTTCCCGGGTCTTGTACACACCGCCCGTCACACCAT<br>GGGAGTTGGTAATGCCCGAAGTCAGTGTTCCAAAAGAGGAAGCTGCCGAAGGCAGGACTGATGACTGGGGTG
120	Streptococcus sp. OTU_398	GACGAACGCTGGCGGCGTGCCTAATACATGCAAGTGGACGCAAGGAAACACACTGTGCTTGCACACCGTGTTTTCTTGA<br>GTCGCGAACGGGTGAGTAACGCGTAGGTAACCTGCCTATTAGCGGGGGATAACTATTGGAAACGATAGCTAATACCGCATA<br>ATATTAATTATTGCATGATAATTGATTGAAAGATGCAAGCGCATCACTAGTAGATGGACCTGCGTTGTATTAGCTAGTTGGTAA<br>GGTAAGAGCTTACCAAGGCGACGATACATAGCCGACCTGAGAGGGTGATCGGCCACACTGGGACTGAGACACGGCCCAG<br>ACTCCTACGGGAGGCAGCAGTAGGGAATCTTCGGCAATGGGGGGAACCCTGACCGAGCAACGCCGCGTGAGTGAAGAAG<br>GTTTTCGGATCGTAAAGCTCTGTTGTAAGAGAAGAACGGGTGTGAGAGTGGAAAGTTCACACTGTGACGGTATCTTACCAGA<br>AAGGGACGGCTAACTACGTGCCAGCAGCCGCGGTAATACGTAGGTCCCGAGCGTTGTCCGGATTTATTGGGCGTAAAGCG<br>AGCGCAGGCGGTTAGATAAGTCTGAAGTTAAAGGCTGTGGCTTAACCATAGTATGCTTTGGAAACTGTTTAACTTGAGTGCA<br>GAAGGGGAGAGTGGAATTCCATGTGTAGCGGTGAAATGCGTAGATATATGGAGGAACACCGGTGGCGAAAGCGGCTCTCT<br>GGTCTGTAACTGACGCTGAGGCTCGAAAGCGTGGGGAGCAAACAGGATTAGATACCCTGGTAGTCCACGCCGTAAACGAT<br>GAGTGCTAGGTGTTAGGCCCTTTCCGGGGCTTAGTGCCGCAGCTAACGCATTAAGCACTCCGCCTGGGGAGTACGACCG<br>CAAGGTTGAAACTCAAAGGAATTGACGGGGGCCCGCACAAGCGGTGGAGCATGTGGTTTAATTCGAAGCAACGCGAAGAAC<br>CTTACCAGGTCTTGACATCCCTCTGACCGCTCTAGAGATAGAGTTTTCCTTCGGGACAGAGGTGACAGGTGGTGCATGGTT<br>GTCGTCAGCTCGTGTCGTGAGATGTTGGGTTAAGTCCCGCAACGAGCGCAACCCCTATTGTTAGTTGCCATCATTCAGTTG<br>GGCACTCTAGCGAGACTGCCGGTAATAAACCGGAGGAAGGTGGGGATGACGTCAAATCATCATGCCCCTTATGACCTGGG<br>CTACACACGTGCTACAATGGCTGGTACAACGAGTCGCAAGCCGGTGACGGCAAGCTAATCTCTGAAAGCCAGTCTCAGTT<br>CGGATTGTAGGCTGCAACTCGCCTACATGAAGTCGGAATCGCTAGTAATCGCGGATCAGCACGCCGCGGTGAATACGTTC<br>CCGGGCCTTGTACACACCGCCCGTCACACCACGAGAGTTTGTAACACCCGAAGTCGGTGAGGTAACCGTAAGGAGCCAG<br>CCGCCTAAGGTGGGATAGATGATTGGGGTG

Sr no.	Species_OTUs	16S reference sequence
121	Tannerella sp. OTU_399	GATGAACGCTAGCGATAGGCTTAACACATGCAAGTCGAGGGGCATCGAGGGTAGCAATACCTGTCGGCGACCGGCGCAC<br>GGGTGAGTAACGCGTATGTAACCTGCCCGCAACAGAGGGATAACCCGGCGAAAGTCGGACTAATACCTCATAAAACAGGG<br>GTTCCGCATGGGGCTATTTGTTAAAGATTTATTGGTTGCGGATGGGCATGCGTACCATTAGGTAGTTGGTGAGGTAACGGC<br>TCACCAAGCCATCGATGGTTAGGGGTTCTGAGAGGAAGGTCCCCCACACTGGTACTGAGACACGGACCAGACTCCTACGG<br>GAGGCAGCAGTGAGGAATATTGGTCAATGGGCGAGAGCCTGAACCAGCCAAGTCGCGTGAAGGATGACTGCCCTATGGGT<br>TGTAAACTTCTTTTACAGGGGAATAAAATGAGATACGTGTATTTTATTGCATGTACCTTGTGAATAAGCATCGGCTAACTCCGT<br>GCCAGCAGCCGCGGTAATACGGAGGATGCGAGCGTTATCCGGATTTATTGGGTTTAAAGGGTGCGTAGGTGGGCTGTTAA<br>GTCCGCGGTGAAAGTTTGTCGCTTAACGATAAAATTGCCGTTGAAACTGGTAGTCTTGAGTATAGATGAAGTAGGCGGAATG<br>CGTGGTGTAGCGGTGAAATGCATAGAGATCACGCAGAACTCCGATTGCGAAGGCAGCTTACTAAGGTATAACTGACACTGA<br>AGCACGAAAGCGTGGGTATCAAACAGGATTAGATACCCTGGTAGTCCACGCAGTAAACGATGATTACTAGGAGTTTGCGATA<br>TAGTGTAAGCTCTACAGCGAAAGCGTTAAGTAATCCACCTGGGGAGTACGCCGGCAACGGTGAAACTCAAAGGAATTGACG<br>GGGGCCCGCACAAGCGGAGGAACATGTGGTTTAATTCGATGATACGCGAGGAACCTTACCCGGGATTGAAATGTAGATGAC<br>GGATGGTGAAAACCGTCTTCCCTTCGGGGCGTCTATGTAGGTGCTGCATGGTTGTCGTCAGCTCGTGCCGTGAGGTGTC<br>GGCTTAAGTGCCATAACGAGCGCAACCCACATCGGTAGTTGCTAACAGTTTTCGCTGAGGACTCTACCGAGACTGCCGTC<br>GTAAGGCGTGAGGAAGGTGTGGATGACGTCAAATCAGCACGGCCCTTACATCCGGGGCGACACACGTGTTACAATGGGAG<br>GGACAAAGGGCAGCTACCGGGCGACCGGATGCGAATCTCTAAACCCTTCCCCAGTTCGGATCGGAGTCTGCAACTCGAC<br>TCCGTGAAGCTGGATTCGCTAGTAATCGCGCATCAGCCATGGCGCGGTGAATACGTTCCCGGGCCTTGTACACACCGCC<br>CGTCAAGCCATGGGAGTCGGGGGTACCTGAAGGGCGTAACCGCAAGGGGCGCACTAGGGTAATACCGGTGACTGGGGCT
122	Lautropia sp. OTU_400	ATTGAACGCTGGCGGCATGCTTTACACATGCAAGTCGGACGGCAGCACAGAGAAGCTTGCTTCTTGGGTGGCGAGTGGCG<br>AACGGGTGAGTAACATATCGGAACGTACCGGGCAGTGGGGGATAACTAATCGAAAGATTAGCTAATACCGCATATTTTCTGA<br>GGAAGAAAGCAGGGGACCATTTGGCCTTGCGCTGTTTGAGCGGCCGATATCTGATTAGCTAGTTGGTGGGGTAAAGGCCT<br>ACCAAGGCGACGATCAGTAGCGGGTCTGAGAGGATGATCCGCCACACTGGGACTGAGACACGGCCCAGACTCCTACGGG<br>AGGCAGCAGTGGGGAATTTTGGACAATGGGCGAAAGCCTGATCCAGCAATGCCGCGTGTGTGATGAAGGCCTTCGGGTTG<br>TAAAGCACTTTTGGCGGGAACGAAAAGGACTGTGCCAATACCACGGTTCGATGACGGTACCCGCAGAATAAGCACCGGCTA<br>ACTACGTGCCAGCAGCCGCGGTAATACGTAGGGTGCAAGCGTTAATCGGAATTACTGGGCGTAAAGAGTGCGCAGGCGGT<br>TTTGCAAGACCGATGTGAAATCCCCGGGCTTAACCTGGGAACTGCATTGGTGACTGCAAGGCTAGAGTGTGTCAGAGGGA<br>GGTGGAATTCCGCATGTAGCAGTGAAATGCGTAGATATGCGGAGGAACACCGATGGCGAAGGCAGCCTCCTGGGATAACA<br>CTGACGCTCATGCACGAAAGCGTGGGGAGCAAACAGGATTAGATACCCTGGTAGTCCACGCCCTAAACGATGTCTACTAGT<br>TGTCGGGGATTAATTTCCTTGGTAACGCAGCTAACGCGTGAAGTAGACCGCCTGGGGAGTACGGTCGCAAGATTAAAACTC<br>AAAGGAATTGACGGGGACCCGCACAAGCGGTGGATGATGTGGATTAATTCGATGCAACGCGAAAAACCTTACCTACGCTTG<br>ACATGTCCGGAACCCTGAAGAGATTTGGGGGTGCTCGAAAGAGAGCCGGAACACAGGTGCTGCATGGCTGTCGTCAGCTC<br>GTGTCGTGAGATGTTGGGTTAAGTCCCGCAACGAGCGCAACCCTTATCATTAGTTGCTACGCAAGGGCACTCTAATGAGAC<br>TGCCGGTGACAAACCGGAGGAAGGTGGGGATGACGTCAAGTCCTCATGGCCCTTATGTGTAGGGCTTCACACGTCATACA<br>ATGGTCGGTACAGAGGGTTGCGAAGCCGCGAGGTGGAGCCAATCCCAAAAAACCGATCGTAGTCCGGATTGCAGTCTGCA<br>ACTCGACTGCATGAAGTCGGAATCGCTAGTAATCGCGGATCAGCATGCCGCGGTGAATACGTTCCCGGGTCTTGTACACA<br>CCGCCCGTCACACCATGGGAGTGGGTTTTACCAGAAGTAGGTAGCTTAACCGCAAGGAGGGCGCTTACCACGGTAGGGC<br>TCATGACTGGGGTG
123	Acidimicrobiales gen. sp. OTU_403	GACGAACGCTGGCGGCGCGCCTAAAACATGCAAGTCGAACGAGAAGCCCGGGCTTGCCCGGGTGGACAGTGGCGAACG<br>GGTGAGTAACACGTGAGGAACCTGCCCCGAAGACCGGGACAACACCGGGAAACCGGTGCTAATACCGGATACCTTCATCG<br>GACCGCATGATCTGATGAAGAAATGGATTCCGCTTCGGGATGGCCTCGCGGCCTATCAGCTTGTTGGTGGGGTAACGGCC<br>CACCAAGGCAACGACGGGTAGCTGGTCTGAGAGGATGTCCAGCCACACTGGGACTGAGACACGGCCCAGACTCCTACGG<br>GAGGCAGCAGTTAGGAATCTTGCGCAATGGACGAAAGTCTGACGCAGCGACGCCGCGTGCGGGATGAATGCCTTCGGGT<br>TGTAAACCGCTTTCAGCAGGGACGAAATTGACGGTACCTGCAGAAGAAGCCCCGGCCAACTACGTGCCAGCAGCCGCGG<br>TAATACGTAGGGGGCGAGCGTTGTCCGGATTTATTGGGCGTAAAGAGCTCGTAGGCGGTTTGGTAAGTCGGATGTGAAATC<br>TCCAGGCTCAACCTGGAGGGGTCATTCGAGACTGCTATGACTAGAGTTCGGTAGAGGAGTGTGGAATTCCTGGTGGAGCG<br>GTGAAATGCGCAGATATCAGGAGGAACACCCGTAGCGAAGGCGGCACTCTGGGCCGATACTGACGCTGAGGAGCGAAAG<br>CGTGGGGAGCGAACAGGATTAGATACCCTGGTAGTCCACGCCGTAAACGTTGGGCACTAGGTGTGGGGTTCTATCGACGG<br>ATTCCGTGCCGTAGCTAACGCATTAAGTGCCCCGCCTGGGGAGTACGGCCGCAAGGCTAAAACTCAAAGGAATTGACGGG<br>GGCCCGCACAAGCGGCGGAGCATGTGGCTTAATTCGATGCGACGCGAAGAACCTTACCTGGGTTTGACATGTAGGGAAAA<br>GCCGTAGAGATACGGTGTCCTTCGGGGCCTTACACAGGTGGTGCATGGCTGTCGTCAGCTCGTGTCGTGAGATGTTGGG<br>TTAAGTCCCGCAACGAGCGCAACCCCTATCCTATGTTGCCAGCGGATTATGCCGGGGACTCGTAGGAGACTGCCGGTGC<br>CAAACCGGAGGAAGGTGGGGACGACGTCAAGTCATCATGCCCCTTACGTCCAGGGCTGCACACATGCTACAATGGCCGG<br>TACAAAGGGTTGCTACTTCGCGAGAAGACGCCAATCCCAGAAAGCCGGTCTCAGTTCGGATTGCAGTCTGCAACTCGACT<br>GCATGAAGTCGGAGTCGCTAGTAATCGCAGATCAGCATTGCTGCGGTGAATACGTTCCCGGGCCTTGTACACACCGCCCG<br>TCACACCACGAAAGTTGGTAACACCCGAAGCCAGTGGCCCAACCCTCGGGAGGGAGCTGTCGAAGGTGGGATCAGCGAT<br>TGGGGTG
124	Eikenella sp. OTU_434	ATTGAACGCTGGCGGCATGCTTTACACATGCAAGTCGAACGGCAGCGGGGTAGTGCTTGCACTACTGTCCGGCGAGTGG<br>CGAACGGGTGAGTAATATATCGGAACGTACCGAGTAATGGGGGATAACCAATCGAAAGATTGGCTAATACCGCATACGTCCT<br>AAGGGAGAAAGCGGGGGATCGCAAGACCTCGCGTTATTCGAGCGGCCGATAACTGATTAGCTAGTTGGTGGGGTAAAGGC<br>CTACCAAGGCGACGATCAGTAGCGGGTCTGAGAGGACGATCCGCCACACTGGGACTGAGACACGGCCCAGACTCCTACG<br>GGAGGCAGCAGTGGGGAATTTTGGACAATGGGGGCAACCCTGATCCAGCCATGCCGCGTGTATGAAGAAGGCCTTCGGG<br>TTGTAAAGTACTTTTGTTAGGGAAGAAAAGGGAAGTGCTAATACCACTTTTTGCTGACGGTACCTAAAGAATAAGCACCGGCT<br>AACTACGTGCCAGCAGCCGCGGTAATACGTAGGGTGCGAGCGTTAATCGGAATTACTGGGCGTAAAGCGAGCGCAGACGG<br>TTATTTAAGCAGGATGTGAAATCCCCGGGCTCAACCTGGGAACTGCGTTCTGAACTGGATAGCTAGAGTGTGTCAGAGGGG<br>GGTAGAATTCCACGTGTAGCAGTGAAATGCGTAGAGATGTGGAGGAATACCGATGGCGAAGGCAGCCCCCTGGGATAACA<br>CTGACGTTCATGCTCGAAAGCGTGGGTAGCAAACAGGATTAGATACCCTGGTAGTCCACGCCCTAAACGATGTCGATTAGC<br>TGTTGGGCAACTTGATTGCTTAGTAGCGTAGCTAACGCGTGAAATCGACCGCCTGGGGAGTACGGTCGCAAGATTAAAACT<br>CAAAGGAATTGACGGGGACCCGCACAAGCGGTGGATGATGTGGATTAATTCGATGCAACGCGAAGAACCTTACCTGGTCTT<br>GACATGTACGGAAGAACTCAGAGACGAGTTTGTGCCTTCGGG

Sr no.	Species_OTUs	16S reference sequence
125	Saccharibacteria sp. OTU_448	GATGAATGCTGGCGGCGTGCCTAACACATGCAAGTCGAGCGGCAGCGCGGGTAGTTTACTACCTGGCGGCGAGCGGCGGACGGCTGAGTAACGCGTAGGAACGTGCCCCAAAGTGAGGAATAACTGCCCGAAAGGGTGGCTAATGCCGCATATGATCTTCGGATTAAAGTATTTATACGCTTTGGGAGCGTCCTGCGTTAGATTAGGTAGTTGGTAGGGTAATGGCCTACCAAGCCGACGATCTATAGCTGGTCTGAGAGGATGATCAGCCAGACTGGGACTGAGACACGGCCCAGACTCCTACGGGAGGCAGCAGTGAGGAATCTTCCACAATGGGCGAAAGCCTGATGGAGCAACGCCGCGTGAAGGATGAAGGCCCTAGGGTTGTAAACTTCTTTTATAAGTGAAGAATATGACGGTAACTTATGAATAAGCACCGGCTAACTACGTGCCAGCAGCCGCGGTCATACGTAGGGTGCAAGCATTATCCGGAGTGACTGGGCGTAAAGAGTTGCGTAGGTGGTTTGTTAAGTAGGTAGTGAAATCTGGCGGCTCAACCGTACAGGCTATTACCTAAACTGGCAAACTCGAGAATGGTAGAGGTAACTGGAATTTCTTGTGTAGGAGTGAAATCCGTAGATATAAGAAGGAACACCAATGGCGTAGGCAGGTTACTGGACCATTTCTGACACTGAGGCACGAAAGCGTGGGGAGCGAACGGGATTAGATACCCCGGTAGTCCACGCCGTAAACGATGGATACTAGCTGTTGGGGGTATCGACCCCCCCAGTAGCGAAGCTAACGCGTTAAGTATCCCGCCTGTGGAGTACGGTCGCAAGACTAAAACATAAAGGAATTGACGGGGACCCGCACAAGCGGTGGATTGTGTTCTTTAATTCGATGATAAACGACGAACCTTACCAGGGCTTGACATCCCGAGAATTAATCCGAAAGGATTGAGTGCTTTATTGAACTCGGTGACAGATCTTGCATGGCCGTCGTCAGCTCGTGTCGTGAGATGTTTGGTTAAGTCCATCAACGAGCGCAACCCTTGTGAATAGTTGTATTTTTCTATTCAGACTGCCCCGGCAACGGGGAGGAAGGAGGGGATGATGTCAGGTCAGTATTAGTCTTACGTCCTGGGCTAGAAACGCAATACAATGGCGAGTACAATGCGCAGCGAAGCCGCGAGGTGAAGCAAATCGCATCAAAGCTCGTCCCAGTTCGGATAAGAGGCTGAAACTCGCCTCTTTGAAGTCGGAATCGCTAGTAATCGCAGATCAGCAAGCTGCGGTGAATACGTTCCCGGGTCTTGTACACACCGCCCGTCAAACCATGAAAGTTACCAACATCCGAAGTCCGATTCGTCGGCCTAAGGTGGGGGGCATGATTGGGGTT
126	Neisseria sp. OTU_457	ATTGAACGCTGGCGGCATGCTTTACACATGCAAGTCGGACGGCAGCACAGAGGAGCTTGCTTCTTGGGTGGCGAGTGGCGAACGGGTGAGTAACGCATCGGAACGTACCGAGTAATGGGGGATAACTGTCCGAAAGGACAGCTAATACCGCATACGCCCTGAGGGGGAAAGCGGGGGATCTTAGGACCTCGCGTTATTCGAGCGGCCGATGTCTGATTAGCTGGTTGGCGGGGTAAAGGCCCACCAAGGCGACGATCAGTAGCGGGTCTGAGAGGATGATCCGCCACACTGGGACTGAGACACGGCCCAGACTCCTACGGGAGGCAGCAGTGGGGAATTTTGGACAATGGGCGCAAGCCTGATCCAGCCATGCCGCGTGTCTGAAGAAGGCCTTCGGGTTGTAAAGGACTTTTGTCAGGGAAGAAAAGGAACGTGTTAATACCATGTTCTGATGACGGTACCTGAAGAATAAGCACCGGCTAACTACGTGCCAGCAGCCGCGGTAATACGTAGGGTGCGAGCGTTAATCGGAATTACTGGGCGTAAAGCGGGCGCAGACGGTTACTTAAGCGGGATGTGAAATCCCCGGGCTCAACCCGGGAACTGCGTTCCGAACTGGGTGGCTAGAGTGTGTCAGAGGGGGGTAGAATTCCACGTGTAGCAGTGAAATGCGTAGAGATGTGGAGGAATACCGATGGCGAAGGCAGCCCCCTGGGATAACACTGACGTTCATGCCCGAAAGCGTGGGTAGCAAACAGGATTAGATACCCTGGTAGTCCACGCCCTAAACGATGTCGATTAGCTGTTGGGGCACTTGATGCCTTAGTAGCGTAGCTAACGCGTGAAATCGACCGCCTGGGGAGTACGGTCGCAAGATTAAAACTCAAAGGAATTGACGGGGACCCGCACAAGCGGTGGATGATGTGGATTAATTCGATGCAACGCGAAGAACCTTACCTGGTCTTGACATGTACGGAATCTTCCAGAGACGGAAGGGTGCCTTCGGG
127	Catonella sp. OTU_460	GATGAACGCTGGCGGCGTGCCTAACACATGCAAGTCGAACGGAGAACATTAACGGAAGTTTTCGGATGGAAGGAAATGTTCTTAGTGGCGGACGGGTGAGTAACGCGTGGGCAACCTGCCCCATACAGGGGGATAGCGTCGGGAAACTGACGGTAAGACCGCATAGGACCACAGAGTCGCATGGCTTGGTGGTGAAATGGGAAACCGGGTATGGGATGGGCCCGCGTCCGATTAGCCAGTTGGCAGGGTAAGAGCCTACCAAAGCAACGATCGGTAGCCGGACTGAGAGGTCGGACGGCCACATTGGGACTGAGACACGGCCCAGACTCCTACGGGAGGCAGCAGTGGGGGATATTGCACAATGGAGGAAACTCTGATGCAGCGACGCCGCGTGAGTGAAGAAGTGCTCCGGCATGTAAAGCTCTTTCAGCAGGGAAGATGATGACGGTACCTGAATAAGAAGCCCCGGCTAACTACGTGCCAGCAGCCGCGGTAATACGTAGGGGGCGAGCGTTATCCGGATTTACTGGGTGTAAAGGGAGCGCAGGCGGTCTGGCAAGTTGAGAGTGGAAGCAGGGGGCTCAACCCCCTGACTGCTCCCAAAACTGTTGGACTGGAGTATGGGAGAGGCAGGCGGAATTCCTAGTGTAGCGGTGAAATGCTCAGATATTAGGAAGAACACCGGTGGCGAAGGCGGCCTGCTGGACCAAAACTGACGCTGAGGCTCGAGAGCGTGGGGAGCGAACAGGATTAGATACCCTGGTAGTCCACGCCGTAAACGATGAATACTAGGTGCCGGGAGGGGATGCCCTATCGGTGCCGTCGCAAACGCATTGAGTATTCCACCTGGGGAGTACGTTCGCAAGAATGAAACTCAAAGGAATTGACGGGGACCCGCACAAGCGGTGGAGCATGTGGTTTAATTCGAAGCAACGCGAAGAACCTTACCCGGTCTTGAGATCCTCCTGACGGATGAGTAATGTCATCTTTTCTTCGGAACAGGAGAGACAGGTGGTGCATGGTTGTCGTCAGCTCGTGTCGTGAGATGTTGGGTTAAGTCCCGCAACGAGCGCAACCCTTATTCTTAGTAGCCAGCGCGTAAAGGCGGGCACTCTAAGGAGACTGCCGGGGATAACCCGGAGGAAGGCGGGGATGACGTCAAATCATCATGCCCCTTATGACCGGGGCTACACACGTGCTACAATGGCGGCCACAGAGAGAGGCGAGGGCGCGAGCCGGAGCGAATCTCATAAAAGCCGTCCCAGTTCGGATTGTAGTCTGCAACTCGACTACATGAAGCTGGAATCGCTAGTAATCGCGAATCAGAATGTCGCGGTGAATACGTTCCCGGGTCTTGTACACACCGCCCGTCACACCATGGGAGTCGGGGACGCCCGAAGTCAGTGACCCAACCGTAAGGAGGGAGCTGCCGAAGGCGGATCCGGTGACTGGGGTG
128	Sphingobacteriaceae gen. sp. OTU_496	GATGAACGCTAGCGGCAGGCCTAATACATGCAAGTCGGACGGGATTGGAGGCTTGCTTCCATGAGAGTGGCGCACGGGTGCGTAACACGTATGTAACCTACCTTTATCTGGGGGATAGCCTCTCGAAAGAGAGATTAACACCGCATAAAATCACAGTACGGCATTGTACAATGATCAAATATTTATAGGATAAAGATGGGCATGCGGGACATTAGCTAGTTGGCGGGGTAACGGCCCACCAAGGCTACGATGTCTAGGGGATCTGAGAGGATGGCCCCCCACACTGGTACTGAGACACGGACCAGACTCCTACGGGAGGCAGCAGTAAGGAATATTGGTCAATGGAGGCAACTCTGAACCAGCCATGCCGCGTGCAGGAAGACAGCCCTATGGGTCGTAAACTGCTTTTATACCGGAATAAACCTCTTTACGTGTAAAGAGTTGAATGTACGGTAGGAATAAGGATCGGCTAACTCCGTGCCAGCAGCCGCGGTAATACGGAGGATCCAAGCGTTATCCGGATTTATTGGGTTTAAAGGGTGCGTAGGCGGCCTGTTAAGTCAGGGGTGAAAGACGGTGGCTCAACCATCGCAGTGCCTTTGATACTGACGGGCTTGAATGCAGCTGAGGTAGGCGGAATGTGACAAGTAGCGGTGAAATGCATAGATATGTCACAGAACACCAATTGCGAAGGCAGCTTACTAAAGTGTGATTGACGCTGAGGCACGAAAGCGTGGGGATCAAACAGGATTAGATACCCTGGTAGTCCACGCCCTAAACGATGAATACTCGATGTTAGCGATATACTGTTAGCGTCCAAGCGAAAGCGTTAAGTATTCCACCTGGGGAGTACGCCCGCAAGGGTGAAACTCAAAGGAATTGACGGGGGCCCGCACAAGCGGAGGAGCATGTGGTTTAATTCGATGATACGCGAGGAACCTTACCCGGGCTTGAAAGTTAGTGAATGATCCAGAGACGGATCAGTCCTTCGGGACACGAAACTAGGTGCTGCATGGCTGTCGTCAGCTCGTGCCGTGAGGTGTTGGGTTAAGTCCCGCAACGAGCGCAACCCCTATGTTTAGTTGCCAGCACGTTATGGTGGGGACTCTAAACAGACTGCCTGTGCAAACAGAGAGGAAGGAGGGGACGACGTCAAGTCATCATGGCCCTTACGTCCGGGGCTACACACGTGCTACAATGGACGGTACAGCGGGCAGCTACCTGGCAACAGGATGCCAATCTCGTAAAGCCGTTCACAGTTCGGATCGGGGTCTGCAACTCGACCCCGTGAAGTTGGATTCGCTAGTAATCGCGTATCAGCAATGACGCGGTGAATACGTTCCCGGGCCTTGTACACACCGCCCGTCAAGCCATGGAAGCTGGGAGTACCTGAAGTACGTAACCGCAAGGAGCGTCCTAGGGTAAAATCGGTAACTGGGGCT

Sr no.	Species_OTUs	16S reference sequence
129	Oribacterium sp. OTU_527	GATGAACGCTGGCGGCGTGCCTAACACATGCAAGTCGAACGGAGTGCCCAGGAGGAAGTTTTCGGATGGAATTCGAAGGCACTTAGTGGCGGACGGGTGAGTAACACGTGGGTAACCTGCCTCATAGAGGGGGATAACAGAGAGAAATCACTGCTAATACCGCATAAGCACACACTGCCGCATGGCAGGGGGTGAAAAGAGATATCGCTATGAGATGGACCCGCGACTGATTAGCCGGTTGGTAAGGTAAGGGCTTACCAAAGCAACGATCAGTAGCCGATCTGAGAGGATGAACGGCCACATTGGGACTGAGACACGGCCCAAACTCCTACGGGAGGCAGCAGTGGGGGATATTGCACAATGGGGGGAACCCTGATGCAGCGACGCCGCGTGAGTGAAGAAGTATTTCGGTATGTAAAGCTCTATCAGCAGGGAAGATAATGACAGTACCTGAATAAGAAGCCCCGGCTAACTACGTGCCAGCAGCCGCGGTAATACGTAGGGGGCAAGCGTTATCCGGATTTACTGGGTGTAAAGGGAGCGTAGACGGAACGGCAAGTCTGAAGTGAAAACCCGTGGCTCAACCGCGGGACTGCTTTGGAAACTGTCGATCTAGAGTGTCGGAGAGGTAAGTGGAATTCCTGGTGTAGCGGTGAAATGCGTAGATATCAGGAAGAACACCGGAGGCGAAGGCGGCTTACTGGACGAAGACTGACGTTGAGGCTCGAAAGCGTGGGAAGCAAACAGGATTAGATACCCTGGTAGTCCACGCCGTAAACGATGAATACTAGGTGTCGGTGGGCGAAGCTCATCGGTGCCGTCGCAAACGCAATAAGTATTCCACCTGGGGAGTACGTTCGCAAGAATGAAACTCAAAGGAATTGACGGGGACCCGCACAAGCGGTGGAGCATGTGGTTTAATTCGAAGCAACGCGAAGAACCTTACCAAGTCTTGAGATCTCTCTGACAGAGTATGTAATGTACTTTTCCTACGGGACAGAGAAGACAGGTGGTGCATGGTTGTCGTCAGCTCGTGTCGTGAGATGTTGGGTTAAGTCCCGCAACGAGCGCAACCCCTATATTCAGTAGCCAGCAGTAAGATGGGCACTCTGGAGAGACTGCCGGGGATAACCCGGAGGAAGGCGGGGATGACGTCAAATCATCATGCCCCTTATGACTTGGGCGACACACGTGCTACAATGGCGTAAACAAAGAGAAGCGAGAGCGCGAGCTTAAGCAAATCCCAAAAATAACGTCTCAGTTCGGACTGTAGTCTGCAACCCGACTACACGAAGCTGGAATCGCTAGTAATCGCAGATCAGCATGCTGCGGTGAATACGTTCCCGGGTCTTGTACACACCGCCCGTCACACCATGGGAGTCGGTAATGCCCGAAGCCAGTGAACCAAAAGAGGGAGCTGTCGAAGGCAGGACTGATAACTGGGGTG
130	Limosilactobacillus sp. OTU_528	GATGAACGCCGGCGGTGTGCCTAATACATGCAAGTCGAACGCGTTGGCCCAATTGATTGATGGTGCTTGCACCTGATTGATTTTGGTCGCCAACGAGTGGCGGACGGGTGAGTAACACGTAGGTAACCTGCCCAGAAGCGGGGGACAACATTTGGAAACAGATGCTAATACCGCATAACAACGTTGTTCGCATGAGCAACGCTTAAAAGATGGCTTCTCGCTATCACTTCTGGATGGACCTGCGGTGCATTAGCTTGTTGGTGGGGTAACGGCCTACCAAGGCGATGATGCATAGCCGAGTTGAGAGACTGATCGGCCACAATGGGACTGAGACACGGCCCATACTCCTACGGGAGGCAGCAGTAGGGAATCTTCCACAATGGGCGCAAGCCTGATGGAGCAACACCGCGTGAGTGAAGAAGGGTTTCGGCTCGTAAAGCTCTGTTGTTAAAGAAGAACACGTATGAGAGTAACTGTTCATACGTTGACGGTATTTAACCAGAAAGTCACGGCTAACTACGTGCCAGCAGCCGCGGTAATACGTAGGTGGCAAGCGTTATCCGGATTTATTGGGCGTAAAGAGAGTGCAGGCGGTTTTCTAAGTCTGATGTGAAAGCCTTCGGCTTAACCGGAGAAGTGCATCGGAAACTGGATAACTTGAGTGCAGAAGAGGGTAGTGGAACTCCATGTGTAGCGGTGGAATGCGTAGATATATGGAAGAACACCAGTGGCGAAGGCGGCTACCTGGTCTGCAACTGACGCTGAGACTCGAAAGCATGGGTAGCGAACAGGATTAGATACCCTGGTAGTCCATGCCGTAAACGATGAGTGCTAGGTGTTGGAGGGTTTCCGCCCTTCAGTGCCGGAGCTAACGCATTAAGCACTCCGCCTGGGGAGTACGACCGCAAGGTTGAAACTCAAAGGAATTGACGGGGGCCCGCACAAGCGGTGGAGCATGTGGTTTAATTCGAAGCAACGCGAAGAACCTTACCAGGTCTTGACATCCCTCTGACCGCTCTAGAGATAGAGTTTTCCTTCGGGACAGAGGTGACAGGTGGTGCATGGTTGTCGTCAGCTCGTGTCGTGAGATGTTGGGTTAAGTCCCGCAACGAGCGCAACCCCTATTGTTAGTTGCCATCATTCAGTTGGGCACTCTAGCGAGACTGCCGGTAATAAACCGGAGGAAGGTGGGGATGACGTCAAATCATCATGCCCCTTATGACCTGGGCTACACACGTGCTACAATGGTTGGTACAACGAGTCGCAAGCCGGTGACGGCAAGCTAATCTCTTAAAGCCAGTCTCAGTTCGGATTGTAGGCTGCAACTCGCCTACATGAAGTCGGAATCGCTAGTAATCGCGGATCAGCACGCCGCGGTGAATACGTTCCCGGGCCTTGTACACACCGCCCGTCACACCACGAGAGTTTGTAACACCCGAAGTCGGTGAGGTAACCTTTTAGGAGCCAGCCGCCTAAGGTGGGATAGATGATTGGGGTG
131	Neisseriaceae gen. sp. OTU_531	ATTGAACGCTGGCGGCATGCTTTACACATGCAAGTCGGACGGCAGCACAGAGAAGCTTGCTTCTTGGGTGGCGAGTGGCGAACGGGTGAGTAATATATCGGAACGTACCGAGTAATGGGGGATAACTAATCGAAAGATTAGCTAATACCGCATATTCTCTGAGGAGGAAAGCAGGGGACCTTCGGGCCTTGCGTTATTCGAGCGGCCGATATCTGATTAGCTAGTTGGTGGGGTAAAGGCCTACCAAGGCGACGATCAGTAGCGGGTCTGAGAGGATGATCCGCCACACTGGGACTGAGACACGGCCCAGACTCCTACGGGAGGCAGCAGTGGGGAATTTTGGACAATGGGCGCAAGCCTGATCCAGCCATGCCGCGTGTCTGAAGAAGGCCTTCGGGTTGTAAAGGACTTTTGTCAGGGAAGAAAAGGCTGTTGCTAATATCGACAGCTGATGACGGTACCTGAAGAATAAGCACCGGCTAACTACGTGCCAGCAGCCGCGGTAATACGTAGGGTGCGAGCGTTAATCGGAATTACTGGGCGTAAAGCGAGCGCAGACGGTTACTTAAGCAGGATGTGAAATCCCCGGGCTCAACCTGGGAACTGCGTTCTGAACTGGGTGACTAGAGTGTGTCAGAGGGAGGTAGAATTCCACGTGTAGCAGTGAAATGCGTAGAGATGTGGAGGAATACCGATGGCGAAGGCAGCCTCCTGGGATAACACTGACGTTCATGCTCGAAAGCGTGGGTAGCAAACAGGATTAGATACCCTGGTAGTCCACGCCGTAAACGATGAGTGCTAGGTGTTAGACCCTTTCCGGGGTTTAGTGCCGCAGCTAACGCATTAAGCACTCCGCCTGGGGAGTACGACCGCAAGGTTGAAACTCAAAGGAATTGACGGGGGCCCGCACAAGCGGTGGAGCATGTGGTTTAATTCGAAGCAACGCGAAGAACCTTACCAGGTCTTGACATCCCTCTGACCGCTCTAGAGATAGAGTTTTCCTTCGGGACAGAGGTGACAGGTGGTGCATGGTTGTCGTCAGCTCGTGTCGTGAGATGTTGGGTTAAGTCCCGCAACGAGCGCAACCCCTATTGTTAGTTGCCATCATTCAGTTGGGCACTCTAGCGAGACTGCCGGTAATAAACCGGAGGAAGGTGGGGATGACGTCAAATCATCATGCCCCTTATGACCTGGGCTACACACGTGCTACAATGGCTGGTACAACGAGTCGCAAGCCGGTGACGGCAAGCTAATCTCTTAAAGCCAGTCTCAGTTCGGATTGTAGGCTGCAACTCGCCTACATGAAGTCGGAATCGCTAGTAATCGCGGATCAGCACGCCGCGGTGAATACGTTCCCGGGCCTTGTACACACCGCCCGTCACACCACGAGAGTTTGTAACACCCGAAGTCGGTGAGGTAACCTTTTAGGAGCCAGCCGCCTAAGGTGGGATAGATGATTGGGGTG
132	Fudania sp. OTU_532	GACGAACGCTGGCGGCGTGCTTAACACATGCAAGTCGAACGATGAAGCGCTGTCCTTTGGGGTGGTGTGGATTAGTGGCGAACGGGTGAGTAATACGTGAGTAACCTGCCCTCTTCTTCGGGATAAGCTTTGGAAACGGGGTCTAATACCGGATAGTCCTGTCTGGTCGCATGGCTGGGTGGGGAAAGAATGTTTTTGGTGGGGGATGGGCTCACGGCCTATCAGCTTGTTGGTGGGGTGATGGCCTACCAAGGCGTCGACGGGTAGCCGGCCTGAGAGGGTGACCGGCCACACTGGGACTGAGATACGGCCCAGACTCCTACGGGAGGCAGCAGTGGGGGATATTGCACAATGGGCGCAAGCCTGATGCAGCGACGCCGCGTGGGGGATGACGGCTTTCGGGTTGTAAACCTCTTTCGGCATGGAACAAGGCTCCGTGTGTGCGGGGTTGAGGGTACGTGCTAAAGAAGCGCCGGCTAACTACGTGCCAGCAGCCGCGGTAATACGTAGGGCGCGAGCGTTGTCCGGAATTATTGGGCGTAAAGAGCTCGTAGGCGGCTTGTCGCGTCTGCTGTGAAAACGCGGGGCTTAACTCCGCGCGTGCAGTGGGTACGGGCAGGCTGGAGTGCGGTAGGGGTGATTGGAATTCCTGGTGTAGCGGTGGAATGCGCAGATATCAGGAAGAACACCGATGGCGAAGGCAGGTCACTGGGCCGTTACTGACGCTGAGGAGCGAAAGCATGGGGAGCGAACAGGATTAGATACCCTGGTAGTCCATGCCGTAAACGTTGGGAACTAGGTGTGGGGTCTGTTCCACGGATTCTGCGCCGGAGCTAACGCGTTAAGTTCCCCGCCTGGGGAGTACGGCCGCAAGGCTAAAACTCAAAGGAATTGACGGGGGCCCGCACAAGCGGCGGAGCATGCGGATTAATTCGATGCAACGCGAAGAACCTTACCAAGGCTTGACATACACCGGAAGGGCGCAGAGATGTGTCTGCCGTTAGGCTGGTGTACAGGTGGTGCATGGTTGTCGTCAGCTCGTGTCGTGAGATGTTGGGTTAAGTCCCGCAACGAGCGCAACCCTTGTCTCGTGTTGCCAGCGCGTGGTGGCGGGGACTCACGGGAGACTGCCGGGGTCAACTCGGAGGAAGGTGGGGATGACGTCAAATCATCATGCCCCTTATGTCTTGGGCTTCACGCATGCTACAATGGCTGGTACAGAGGGTGGCGAGACCGTGAGGTGGAGCGAATCCCGGAAAGCCGGTCTCAGTTCGGATCGGGGTCTGCAACTCGGCCCCGTGAAGTCGGAGTCGCTAGTAATCGCAGATCAGCAACGCTGCGGTGAATACGTTCTCGGGCCTTGTACACACCGCCCGTCACGTCACGAAAGTCGGCAACGCCCGAAGCCCGTGGCCCAACCTGTGTGGGGGGAGCGGTCGAAGGCGGGGTTGGCGATTGGGACG

Sr no.	Species_OTUs	16S reference sequence
133	Porphyromonas sp. OTU_538	GATGAACGCTAGCGATAGGCTTAACACATGCAAGTCGAGGGGCAGCATTATTTTAGCTTGCTAAGATAGATGGCGACCGGC<br>GCACGGGTGCGTAACGCGTATGCAACCTGCCTGTAATTAGGGAATAACCCGGTGAAAGTCGGACTAATACCCTATATTCTT<br>CTTTCTCCGCATGGGGGAGGACGGAAAGATTTATTGATTACAGATGGGCATGCGTCCCATTAGCTTGTTGGTAAGGTAACG<br>GCTTACCAAGGCAACGATGGGTAGGGGGACTGAGAGGTTGACCCCCCACACTGGTACTGAGACACGGACCAGACTCCTA<br>CGGGAGGCAGCAGTGAGGAATATTGGTCAATGGGCGAGAGCCTGAACCAGCCAAGTCGCGTGAAGGACGACGGTTCTATG<br>GATTGTAAACTTCTTTTGTAGAGGAATAATGGCAGCTACGCGTAGCTGAGATGCATGTACTCTACGAATAAGTATCGGCTAAC<br>TCCGTGCCAGCAGCCGCGGTAATACGGAGGATACGAGCGTTATCCGGAATTATTGGGTTTAAAGGGTGCGTAGGTGGCGT<br>ATTAAGTCAGTGGTGAAAAGCTGCAGCTCAACTGTAGTCTTGCCGTTGAAACTGATATGCTAGAGAGGAGACGAGGTATGCG<br>GAATGTGTGGTGTAGCGGTGAAATGCATAGATATCACACAGAACGCCGATTGCGAAGGCAGCGTACCAGGCTCCGTCTGA<br>CACTGAAGCACGAAAGCGTGGGGATCAAACAGGATTAGATACCCTGGTAGTCCACGCAGTAAACGATGAATACTAGATTTTT<br>GCGATATACTGTAAGAGTCTAAGCGAAAGCGATAAGTATTCCACCTGGGGAGTACGCCGGCAACGGTGAAACTCAAAGGAA<br>TTGACGGGGGCCCGCACAAGCGGAGGAACATGTGGTTTAATTCGATGATACGCGAGGAACCTTACCCGGGATTGAAATGTA<br>GATGACGGATGGTGAAAACCGTCTTCCCTTCGGGGCGTCTATGTAGGTGCTGCATGGTTGTCGTCAGCTCGTGCCGTGAG<br>GTGTCGGCTTAAGTGCCATAACGAGCGCAACCCACATCGGTAGTTGCTAACAGTTTTCGCTGAGGACTCTACCGAGACTG<br>CCGTCGTAAGGCGCGAGGAAGGTGTGGATGACGTCAAATCAGCACGGCCCTTACATCCGGGGCGACACACGTGTTACAAT<br>GGGAGGGACAAAGGGCAGCTACCGGGCGACCGGATGCGAATCTCTAAACCCTTCCCCAGTTCGGATCGGAGTCTGCAAC<br>TCGACTCCGTGAAGCTGGATTCGCTAGTAATCGCGCATCAGCCATGGCGCGGTGAATACGTTCCCGGGCCTTGTACACAC<br>CGCCCGTCAAGCCATGGGAGTCGGGGGTACCTGAAGGGCGTAACCGCAAGGGGCGCACTAGGGTAATACCGGTGACTG<br>GGGCT
134	Saccharibacteria sp. OTU_591	GATGAACGCTGGCGGCGTGCCTAATACATGCAAGTCGAGCGGTAAGGTTCTGCACTTAGTATTTGACCAGATCAAGTCCGT<br>ATCTCCTCGTGAGATATAGAAACTGACTGAAGCAAGTTGCCTCTGGCTAGAGGTAACGAGTCGAATCCTGGGTGTAGAATAC<br>ACGAGCGGCGGACGGCTGAGTAACACGTAGGAACGTACCCCAAAGTGAGGGATAAGCACCAGAAATGGTGTCTAATACCG<br>CATGTGATCTTCGGATTAAAGCTTCGGCGCTTTGGGAACGGCCTGCGCATGATTAGCTAGTTGGTGAGGTAATGGCTCACC<br>AAGGCGACGATCATTAGCTGGTCTGAGAGGATGATCAGCCAGACTGGAACTGAGAACGGTCCAGACTCCTACGGGAGGCA<br>GCAGTAGGGAATTTTCCACAATGGACGAAAGTCTGATGGAGCAACGCCGCGTGCAGGATGAAGGCCCTTGGGTCGTAAAC<br>TGCTTTTATATGTGACGATAATGACGGTAGCATATGAATAAGGATCGGCTAACTCCGTGCCAGCAGCCGCGGTCATACGGA<br>GGATCCAAGCGTTATCCGGAATTACTGGGCGTAAAGAGTTGCGTAGGTGGCATTGTAAGTTGATAGTGAAAGCGTCAGGCT<br>CAACCGGATATCCATTATCAAAACTGCAAAGCTAGAGGATGAGAGAGGTTATTGGAATTCCTTGTGTAGGAGTGAAATCCGTA<br>GATATAAGGAGGAACACCGATGGCGTAGGCAGATAACTGGCTCATTCCTGACACTAAGGCACGAAAGCGTGGGTAGCAAAC<br>GGGATTAGATACCCCGGTAGTCCACGCCGTAAACGATGGATGCTAGCTGTTATCGGTATCGACCCCGGTAGTAGCGAAGC<br>TAACGCGTTAAGCATCCCGCCTGTGGAGTACGGTCGCAAGACTAAAACATAAAGGAATTGACGGGGACCCGCACAAGCGG<br>TGGAGCGTGTTGTTTAATTCGATGGTAAGCGAAGAACCTTACCCAGGTTTGACATCCTTGGAATTTTTCGAAAGATAAAAGT<br>GCTTTATTGAGCCAAGTGACAGGTGTTGCATGGCCGTCGTCAGCTCGTGTCGTGAGATGTTTGGTTAAGTCCATCAACGAG<br>CGCAACCCTTGTGAGTAGTTGAATTTTTCTACTCAGACTGCCCTGGCAACAGGGAGGAAGGAGGGGATGATGTCAGGTCAG<br>TATTACCCTTACATCTGGGGCTACAAACACGCTACAATGGCCGGTACAAAGGGCAGCCAAGTCGCGAGACGGAGCAAATC<br>CCATCAAAGCCGGTCTCAGTTCGGATAGCAGGCTGAAACTCGCCTGCTTGAAGTCGGAATCGCTAGTAACGGTAGGTCAG<br>CTATACTACCGTGAATACGTTCCCGGGTCTTGTACACACCGCCCGTCAAACCATGAAAGTCGCCAATACCTGACGTATGAG<br>CTTCGACTCGTCCTAAGGTAGGGGAGATGATTGGGGTT
135	Streptococcus sp. OTU_598	GACGAACGCTGGCGGCGTGCCTAATACATGCAAGTAGAACGCTGAAGGAGGAGCTTGCTTCTCCGGATGAGTTGCGAACG<br>GGTGAGTAACGCGTAGGTAACCTGCCTGGTAGCGGGGGATAACTATTGGAAACGATAGCTAATACCGCATAAGAGTAGATG<br>TTGCATGACATTTGCTTAAAAGGTGCAATTGCATCACTACCAGATGGACCTGCGTTGTATTAGCTAGTTGGTGGGGTAACGG<br>CTCACCAAGGCGACGATACATAGCCGACCTGAGAGGGTGATCGGCCACACTGGGACTGAGACACGGCCCAGACTCCTAC<br>GGGAGGCAGCAGTAGGGAATCTTCGGCAATGGACGGAAGTCTGACCGAGCAACGCCGCGTGAGTGAAGAAGGTTTTCGGA<br>TCGTAAAGCTCTGTTGTAAGAGAAGAACGAGTGTGAGAGTGGAAAGTTCACACTGTGACGGTATCTTACCAGAAAGGGACGG<br>CTAACTACGTGCCAGCAGCCGCGGTAATACGTAGGTCCCGAGCGTTGTCCGGATTTATTGGGCGTAAAGCGAGCGCAGG<br>CGGTTAGATAAGTCTGAAGTTAAAGGCTGTGGCTTAACCATAGTACGCTTTGGAAACTGTTTAACTTGAGTGCAAGAGGGGA<br>GAGTGGAATTCCATGTGTAGCGGTGAAATGCGTAGATATATGGAGGAACACCGGTGGCGAAAGCGGCTCTCTGGCTTGTAA<br>CTGACGCTGAGGCTCGAAAGCGTGGGGAGCAAACAGGATTAGATACCCTGGTAGTCCACGCCGTAAACGATGAGTGCTAG<br>GTGTTAGACCCTTTCCGGGGTTTAGTGCCGCAGCTAACGCATTAAGCACTCCGCCTGGGGAGTACGACCGCAAGGTTGAA<br>ACTCAAAGGAATTGACGGGGGCCCGCACAAGCGGTGGAGCATGTGGTTTAATTCGAAGCAACGCGAAGAACCTTACCAGG<br>TCTTGACATCCCTCTGACCGCTCTAGAGATAGAGCTTTCCTTCGGGACAGAGGTGACAGGTGGTGCATGGTTGTCGTCAG<br>CTCGTGTCGTGAGATGTTGGGTTAAGTCCCGCAACGAGCGCAACCCTTATATCTAGTTGCCAGCAGTAAGATGGGGACTCT<br>AGATAGACTGCCAGTGATAAACTGGAGGAAGGTGGGGATGACGTCAAATCATCATGCCCCTTATGACTTGGGCTACACACG<br>TGCTACAATGGATAGGAACAAAGAGAAGCGAGCTCGCGAGAGTAAGCAAACCTCACAAAACTATTCTCAGTTCGGATTGTAG<br>TCTGCAACTCGACTACATGAAGCTGGAATCGCTAGTAATCGCGAATCAGAATGTCGCGGTGAATACGTTCCCGGGTCTTGT<br>ACACACCGCCCGTCACACCACGAGAGTTTGTAACACCCGAAGACGGTGGCCTAACCTTTTAGGAGGGAGCCGGTCACGG<br>TGGGACAGATGATTGGGGTG
136	Prevotella sp. OTU_599	GATGAACGCTAGCTACAGGCTTAACACATGCAAGTCGAGGGGAAACGACATTGAAGCTTGCTTCGATGGCCGTCGACCGG<br>CGCACGGGTGAGTAACGCGTATCCAACCTGCCTCTGACTAAGGGATAACCCGGCGAAAGTCGGACTAATACCTTATGAGG<br>TTTTCAGCAGACATCTAATGAAAACGAAAGATTTATCGGTCAGCGATGGGGATGCGTCTGATTAGCTTGTTGGCGGGGTAAC<br>GGCCCACCAAGGCAACGATCAGTAGGGGTTCTGAGAGGAAGGTCCCCCACATTGGAACTGAGACACGGTCCAAACTCCTA<br>CGGGAGGCAGCAGTGAGGAATATTGGTCAATGGGCGAGAGCCTGAACCAGCCAAGTAGCGTGCAGGATGACGGCCCTAT<br>GGGTTGTAAACTGCTTTTATGTGGGGATAAAGTGAGCTACGTGTAGTTTATTGCAGGTACCACATGAATAAGGACCGGCTAA<br>TTCCGTGCCAGCAGCCGCGGTAATACGGAAGGTCCGGGCGTTATCCGGATTTATTGGGTTTAAAGGGAGCGTAGGCCGTG<br>GATTAAGCGTGTTGTGAAATGTAGACGCTCAACGTCTGAATTGCAGCGCGAACTGGTTCACTTGAGTATGCGCAACGTAGG<br>CGGAATTCGTCGTGTAGCGGTGAAATGCTTAGATATGACGAAGAACTCCGATTGCGAAGGCAGCTTACGGGAGCACAACTG<br>ACGCTGAAGCTCGAAGGTGCGGGTATCAAACAGGATTAGATACCCTGGTAGTCCGCACAGTAAACGCTGGATGCCCGTTG<br>TTAGTTCACTTATGAATTAGCGACCAAGCGAAAGCATTAAGCATCCCACCTGGGGAGTACGCCGGCAACGGTGAAACTCAA<br>AGGAATTGACGGGGGCCCGCACAAGCGGAGGAACATGTGGTTTAATTCGATGATACGCGAGGAACCTTACCCGGGCTTGA<br>ATTGCAGTCGCTGCCCACAGAGACGTGGGCTTCCCTTCGGGGCGTCTGTGAAGGTGCTGCATGGTTGTCGTCAGCTCGT<br>GCCGTGAGGTGTCGGCTTAAGTGCCATAACGAGCGCAACCCCCTTTCCCAGTTGCCATCGGGTTATGCCGGGCACTCTG<br>GGGACACTGCCACCGCAAGGTGCGAGGAAGGTGGGGATGACGTCAAATCAGCACGGCCCTTACGTCCGGGGCTACACAC<br>GTGTTACAATGGCCGGTACAGAGGGACGGTGCAATGCAAGTTGCATCCAATCTTGAAATCCGGTCCCAGTTCGGACTGGG<br>GTCTGCAACCCGACCCCACGAAGCTGGATTCGCTAGTAATCGCGCATCAGCCATGGCGCGGTGAATACGTTCCCGGGCC<br>TTGTACACACCGCCCGTCAAGCCATGAAAGCCGGGGGTGCCTGAAGTTCGTGACCGCGAGGAGCGACCTAGGGCAAGAC<br>CGGTGATTGGGGCT

Sr no.	Species_OTUs	16S reference sequence
137	Eubacterium sp. OTU_600	GATGAACGCTGGCGGCGTGCTTAACACATGCAAGTCGAGCGAGAAGTTTTGAAAAGATTCTTCGGATGAAATTTAAAATGGA<br>AAGCGGCGGACGGGTGAGTAACGCGTAGGCAACCTGCCCCATACAAAGGGATAGCATTTGGAAACGAATATTAATACCTTA<br>TGAAACTTAACTAGTGCATGCTAGGTAGGTCAAAGATTTATCGGTATGGGATGGGCCTGCGTCTGATTAGCTAGTTGGTGAG<br>GTAACGGCTCACCAAGGCGACGATCAGTAGCCGACCTGAGAGGGTGAACGGCCACATTGGAACTGAGACACGGTCCAAAC<br>TCCTACGGGAGGCAGCAGTGGGGAATATTGCACAATGGGGGAAACCCTGATGCAGCAACGCCGCGTGAACGATGAAGGC<br>CTTTGGGTCGTAAAGTTCTGTTCTAGGTGATGAAAACTGACAGTAACCTAGGAGAAAGCCCCGGCTAACTCCGTGCCAGCA<br>GCCGCGGTAATACGGAGGGGGCAAGCGTTATCCGGAATTATTGGGCGTAAAGGGTACGTAGGTGGCCTTTTAAGCGTAGG<br>GTATAAGGCAATGGCTCAACCATTGTTCGCCCTATGAACTGGAAGGCTTGAGTGCAGGAGAGGAAAGCGGAATTCCTAGTG<br>TAGCGGTGGAATGCATAGATATTAGGAGGAACATCAGCGGCGAAGGCGGCTTTCTGGACTGCAACTGACACTGAGGTACGA<br>AAGCGTGGGGAGCAAACAGGATTAGATACCCTGGTAGTCCACGCCGTAAACGATGAGCACTAGGTGTCGGGGTCGTAAGG<br>CTTCGGTGCCGTAGTTAACGCATTAAGTGCTCCGCCTGGGGAGTACGCACGCAAGTGTGAAACTCAAAGGAATTGACGGG<br>GACCCGCACAAGCAGCGGAGCATGTGGTTTAATTCGAAGCAACGCGAAGAACCTTACCAGGACTTGACATCCTTCTGACC<br>GGTCTTTAATAGGACCTTTCTTTTGACAGAAGAGACAGGTGGTGCATGGTTGTCGTCAGCTCGTGTCGTGAGATGTTGGGT<br>TAAGTCCCGCAACGAGCGCAACCCCTATTGTTAGTTGCCATCATTCAGTTGGGCACTCTAGCGAGACTGCCGGTAATAAAC<br>CGGAGGAAGGTGGGGATGACGTCAAATCATCATGCCCCTTATGACCTGGGCTACACACGTGCTACAATGGCTGGTACAAC<br>GAGTCGCAAGCCGGTGACGGCAAGCTAATCTCTTAAAGCCAGTCTCAGTTCGGATTGTAGGCTGCAACTCGCCTACATGAA<br>GTCGGAATCGCTAGTAATCGCGGATCAGCACGCCGCGGTGAATACGTTCCCGGGCCTTGTACACACCGCCCGTCACACC<br>ACGAGAGTTTGTAACACCCGAAGTCGGTGAGGTAACCTTTTAGGAGCCAGCCGCCTAAGGTGGGATAGATGATTGGGGTG
138	Rothia sp. OTU_601	GACGAACGCTGGCGGCGTGCCTAATACATGCAAGTAGAACGCTGAAGAGAGGAGCTTGCTCTTCTTGGATGAGTTGCGAA<br>CGGGTGAGTAACGCGTAGGTAACCTGCCTGGTAGCGGGGGATAACTATTGGAAACGATAGCTAATACCGCATAAAATTGATT<br>ATTGCATGATAATTAATTGAAAGATGCAATTGCATCACTACCAGATGGACCTGCGTTGTATTAGCTAGTTGGTGAGGTAATGG<br>CTTACCAAGGCGACGACGGGTAGCCGGCCTGAGAGGGTGACCGGCCACACTGGGACTGAGACACGGCCCAGACTCCTA<br>CGGGAGGCAGCAGTGGGGAATATTGCACAATGGGCGCAAGCCTGATGCAGCGACGCCGCGTGAGGGATGACGGCCTTC<br>GGGTTGTAAACCTCTGTTAGCATCGAAGAAGCGAAAGTGACGGTAGGTGCAGAGAAAGCGCCGGCTAACTACGTGCCAGC<br>AGCCGCGGTAATACGTAGGGCGCGAGCGTTGTCCGGAATTATTGGGCGTAAAGAGCTTGTAGGCGGTTGGTCGCGTCTG<br>CTGTGAAAGGCTGGGGCTTAACCCTGGTTTTGCAGTGGGTACGGGCTAACTAGAGTGCAGTAGGGGAGACTGGAATTCCT<br>GGTGTAGCGGTGGAATGCGCAGATATCAGGAGGAACACCGATGGCGAAGGCAGGTCTCTGGGCTGTAACTGACGCTGAGA<br>AGCGAAAGCATGGGGAGCGAACAGGATTAGATACCCTGGTAGTCCATGCCGTAAACGTTGGGCACTAGGTGTGGGGGACA<br>TTCCACGTTTTCCGCGCCGTAGCTAACGCATTAAGTGCCCCGCCTGGGGAGTACGGCCGCAAGGCTAAAACTCAAAGAAA<br>TTGACGGGGGCCCGCACAAGCGGCGGAGCATGCGGATTAATTCGATGCAACGCGAAGAACCTTACCAAGGCTTGACATAT<br>ACTGGACTGCGTCAGAGATGGCGTTTCCCTTCGGGGCTGGTATACAGGTGGTGCATGGTTGTCGTCAGCTCGTGTCGTGA<br>GATGTTGGGTTAAGTCCCGCAACGAGCGCAACCCTCGTTCTATGTTGCCAGCACGTGATGGTGGGGACTCATAGGAGACT<br>GCCGGGGTCAACTCGGAGGAAGGTGGGGATGACGTCAAATCATCATGCCCCTTATGTCTTGGGCTTCACGCATGCTACAA<br>TGGCCGGTACAGAGGGTTGCGATACTGTGAGGTGGAGCTAATCCCTAAAAGCCGGTCTCAGTTCGGATTGGGGTCTGCAA<br>CTCGACCCCATGAAGTCGGAGTCGCTAGTAATCGCAGATCAGCAACGCTGCGGTGAATACGTTCCCGGGCCTTGTACACA<br>CCGCCCGTCAAGTCACGAAAGTTGGTAACACCCGAAGCCGGTGGCCTAACCCTGGTGGGGGGAGCCGTCGAAGGTGGG<br>ACTGGCGATTGGGACT
139	Rothia sp. OTU_633	GACGAACGCTGGCGGCGTGCTTAACACATGCAAGTCGAACGATGAAGCCTAGCTTGCTAGGTGGATTAGTGGCGAACGGG<br>TGAGTAATACGTGAGTGACCTACCTTTGACTCTGGGATAAGCCTGGGAAACTGGGTCTAATACCGGATACGACCAATCTCC<br>GCATGGGGTGTTGGTGGAAAGCGTTATGGAGTGGTTTTAGATGGGCTCACGGCCTATCAGCTTGTTGGTGAGGTAATGGCT<br>TACCAAGGCGACGACGGGTAGCCGGCCTGAGAGGGTGACCGGCCACACTGGGACTGAGACACGGCCCAGACTCCTACG<br>GGAGGCAGCAGTGGGGAATATTGCACAATGGGCGCAAGCCTGATGCAGCGACGCCGCGTGAGGGATGACGGCCTTCGG<br>GTTGTAAACCTCTGTTAGCATCGAAGAAGCGAAAGTGACGGTAGGTGCAGAGAAAGCGCCGGCTAACTACGTGCCAGCAG<br>CCGCGGTAATACGTAGGGCGCGAGCGTTGTCCGGAATTATTGGGCGTAAAGAGCTTGTAGGCGGTTGGTCGCGTCTGCT<br>GTGAAAGGCTGGGGCTTAACCCTGGTTTTGCAGTGGGTACGGGCTAACTAGAGTGCAGTAGGGGAGACTGGAATTCCTGG<br>TGTAGCGGTGGAATGCGCAGATATCAGGAGGAACACCGATGGCGAAGGCAGGTCTCTGGGCTGTAACTGACGCTGAGAAG<br>CGAAAGCATGGGGAGCGAACAGGATTAGATACCCTGGTAGTCCATGCCGTAAACGTTGGGCACTAGGTGTGGGGGACATT<br>CCACGTTTTCCGCGCCGTAGCTAACGCATTAAGTGCCCCGCCTGGGGAGTACGGCCGCAAGGCTAAAACTCAAAGAAATT<br>GACGGGGGCCCGCACAAGCGGCGGAGCATGCGGATTAATTCGATGCAACGCGAAGAACCTTACCAAGGCTTGACATATAC<br>TGGACTGCGTCAGAGATGGCGTTTCCCTTCGGGGCTGGTATACAGGTGGTGCATGGTTGTCGTCAGCTCGTGTCGTGAGA<br>TGTTGGGTTAAGTCCCGCAACGAGCGCAACCCTCGTTCTATGTTGCCAGCACGTGATGGTGGGGACTCATAGGAGACTGC<br>CGGGGTCAACTCGGAGGAAGGTGGGGATGACGTCAAATCATCATGCCCCTTATGACCTGGGCTACACACGTGCTACAATG<br>GCTGGTACAACGAGTCGCAAGCCGGTGACGGCAAGCTAATCTCTGAAAGCCAGTCTCAGTTCGGATTGTAGGCTGCAACT<br>CGCCTACATGAAGTCGGAATCGCTAGTAATCGCGGATCAGCACGCCGCGGTGAATACGTTCCCGGGCCTTGTACACACC<br>GCCCGTCACACCACGAGAGTTTGTAACACCCGAAGTCGGTGAGGTAACCGTAAGGAGCCAGCCGCCTAAGGTGGGATAGA<br>TGATTGGGGTG
140	Saccharibacteria sp. OTU_643	GATGAACGCTGGCGGTGTGCCTAATACATGCAAGTCGAGCGGTAAGGCTTTCACAGATTATTTGAACCTGATAAGTCCATG<br>CAAATGGAAACTATCCGACAATGTTTCTACAGGCTAGTAGAAGATAGTCGAATAATGTGTGAAAGTACACGAGCGGCGGACG<br>GCTGAGTAACGCGTAGGAACGTACCCCAAAGTGAGGGATAAGCACCGGAAACGGTGTCTAATACCGCATGTGATCTAAGGA<br>TTAAAGCCTTCGGGCGCTTTGGGAGCGGCCTGCGTAAGATTAGCTAGTTGGTGAGGTAAAAGCTCACCAAGGCGACGATC<br>TTTAGCTGGTCTGAGAGGATGATCAGCCAGACTGGGACTGAGACACGGCCCAGACTCCTACGGGAGGCAGCAGTAGGGAA<br>TTTTCCACAATGGGCGAAAGCCTGATGGAGCAACACCGCGTGCAGGATGAAGGCCTTCGGGTCGTAAACTGCTTTTATGAG<br>TGATGATTTAAGACAGTAGCTCAAGAATAAGGGTCGGCTAACTACGTGCCAGCAGCCGCGGTCATACGTAGGACCCAAGCG<br>TTATCCGGAATTACTGGGCGTAAAGAGTTGCGTAGGCGGCTTGTTAAGCAAGGTGTGAAATCGTACGGCTCAACCGTATAC<br>ACATACTTTGAACTGGCAAGCTAGAGAGTGAGAGAGGTTATTGGAATTCCAAGTGTAGGAGTGAAATCCGTAGATATTTGGAG<br>GAACACCGATGGCGTAGGCAGATAACTGGCTCACTTCTGACGCTCAGGCACGAAAGCGTGGGGAGCAAATGGGATTAGAT<br>ACCCCAGTAGTCCACGCCCTAAACTATGGATGCTAGCTGTATGTCGAATCGACCCGATATGTAGCGAAGCTAACGCGTTAA<br>GCATCCCGCCTGTGGAGTACGGTCGCAAGACTAAAACATAAAGGAATTGACGGGGACCCGCACAAGCGGTGGAGTGTGTT<br>GTTTAATTCGATGGTAAACGAAGAACCTTACCCAGACTTGACATCCTTGGAATGACTACGAAAGTAGACAGTGTCTTCGGAA<br>GCCAAGTGACAGGTGTTGCATGGCCGTCGTCAGCTCGTGTCGTGAGATGTTAGGTTAAGTCCTTCAACGAGCGCAACCCT<br>TGTAGTTAGTTGTATTTTTCTAGCTAGACTGCCCTGGTAACAGGGAGGAAGGAGGGGATGATGTCAGGTCAGTATTACCCTT<br>ACGTCTGGGGCTACAAACACGCTACAATGGCCGGTACAAAGGGAAGCCAACCCGCGAGGGGGAGCAAATCCCATCAAAGC<br>CGGTCTCAGTTCGGATTGCAGGCTGAAACTCGCCTGCATGAAGCTGGAATCGCTAGTAACGGTAAGTCAGCAGATTACCGT<br>GAATACGTTCCCGGGTCTTGTACACACCGCCCGTCAAACCATGAAAGTCGCCAATACCTGACGTCCTAGCTTGTCTAGGC<br>CTAAGGTAGGGGAGATGATTGGGGTT

Sr no.	Species_OTUs	16S reference sequence
141	Abiotrophia sp. OTU_650	GACGAACGCTGGCGGCGTGCCTAATACATGCAAGTCGAACGAACCGCGACTAGGTGCTTGCACTTGGTCAAGGTGAGTGG<br>CGAACGGGTGAGTAACACGTGGGTAACCTACCTCATAGTGGGGGATAACAGTCGGAAACGACTGCTAATACCGCATAGGAC<br>ATAGGATCACATGATTCCGTGAGGAAAGGTGGCGCAAGCTATCGCTAAGAGATGGACCCGCGGTGCATTAGCTAGTTGGTA<br>GGGTAAAGGCCTACCAAGGCGATGATGCATAGCCGACCTGAGAGGGTGATCGGCCACATTGGGACTGAGACACGGCCCA<br>AACTCCTACGGGAGGCAGCAGTAGGGAATCTTCCGCAATGGACGCAAGTCTGACGGAGCAACGCCGCGTGAGTGAAGAAG<br>GTCTTCGGATCGTAAAGCTCTGTTGTTAGAGAAGAACAGCGCATAGAGTAACTGTTATGCGTGTGACGGTATCTAACCAGAA<br>AGCCACGGCTAACTACGTGCCAGCAGCCGCGGTAATACGTAGGTGGCGAGCGTTGTCCGGATTTATTGGGCGTAAAGGGA<br>GTGTAGGCGGTCTTTTAAGTCTGATGTGAAAGCCCACGGCTCAACCGTGGAGGGTCATTGGAAACTGGGAGACTTGAGTG<br>CAGAAGAGGAGAGCGGAATTCCATGTGTAGCGGTGAAATGCGTAGATATATGGAGGAACACCAGTGGCGAAGGCGGCTCT<br>CTGGTCTGTAACTGACGCTGAGGCTCGAAAGCGTGGGGAGCAAACAGGATTAGATACCCTGGTAGTCCACGCCGTAAACG<br>ATGAGTGCTAGGTGTTAGGCCCTTTCCGGGGCTTAGTGCCGCAGCTAACGCATTAAGCACTCCGCCTGGGGAGTACGAC<br>CGCAAGGTTGAAACTCAAAGGAATTGACGGGGGCCCGCACAAGCGGTGGAGCATGTGGTTTAATTCGAAGCAACGCGAAG<br>AACCTTACCAGGTCTTGACATCCCTCTGACCGCTCTAGAGATAGAGTTTTCCTTCGGGACAGAGGTGACAGGTGGTGCATG<br>GTTGTCGTCAGCTCGTGTCGTGAGATGTTGGGTTAAGTCCCGCAACGAGCGCAACCCCTATTGTTAGTTGCCATCATTCAG<br>TTGGGCACTCTAGCGAGACTGCCGGTAATAAACCGGAGGAAGGTGGGGATGACGTCAAATCATCATGCCCCTTATGACCT<br>GGGCTACACACGTGCTACAATGGCTGGTACAACGAGTCGCAAGCCGGTGACGGCAAGCTAATCTCTGAAAGCCAGTCTCA<br>GTTCGGATTGTAGGCTGCAACTCGCCTACATGAAGTCGGAATCGCTAGTAATCGCGGATCAGCACGCCGCGGTGAATACG<br>TTCCCGGGCCTTGTACACACCGCCCGTCACACCACGAGAGTTTGTAACACCCGAAGTCGGTGAGGTAACCGTAAGGAGC<br>CAGCCGCCTAAGGTGGGATAGATGATTGGGGTG
142	Desulfovibrio sp. OTU_654	ATTGAACGCTGGCGGCGTGCTTAACACATGCAAGTCGAACGCGAAAGGGGCTTCGGCCCTGAGTAAAGTGGCGCACGGG<br>TGAGTAACACGTGGATAATCTGCCCTTATGATCGGGATAACAGTCGGAAACGGCTGCTAATACCGGATACGCTCAAAATGAA<br>CTTTTTGGGGAAAGGTGACCTCTGCTTGCAAGTTACCGCATGAGGATGAGTCCGCGTCCCATTAGCTTGTTGGTGGGGTAA<br>TGGCCTACCAAGGCAACGATGGGTAGCCGATTTGAGAGGATGATCGGCCACACTGGGACTGAAACACGGCCCAGACTCCT<br>ACGGGAGGCAGCAGTGGGGAATATTGCGCAATGGGCGAAAGCCTGACGCAGCGACGCCGCGTGAGGGATGAAGGTTTTC<br>GGATCGTAAACCTCTGTCAGAAGGGAAGAAACCGCACTGTGCTAATCAGCAGTGCCTTGACGGTACCTTCAAAGGAAGCAC<br>CGGCTAACTCCGTGCCAGCAGCCGCGGTAATACGGAGGGTGCAAGCGTTAATCGGAATTACTGGGCGTAAAGCGCACGTA<br>GGCTGCTTTGTAAGTCAGGGGTGAAATCCCACGGCTCAACCGTGGAACTGCCTTTGATACTGCTTAGCTTGAATTCGGGAG<br>AGGGTGGCGGAATTCCAGGTGTAGGAGTGAAATCCGTAGATATCTGGAGGAACATCAGTGGCGAAGGCGGCCACCTGGAC<br>CGGTATTGACGCTGAGGTGCGAAAGCGTGGGGAGCAAACAGGATTAGATACCCTGGTAGTCCACGCTGTAAACGATGGAT<br>GCTAGATGTCGGGGAGTACTCTTCGGTGTCGTAGTTAACGCGTTAAGCATCCCGCCTGGGGAGTACGGTCGCAAGGCTGA<br>AACTCAAAGAAATTGACGGGGGCCCGCACAAGCGGTGGAGTATGTGGTTTAATTCGATGCAACGCGAAGAACCTTACCTAG<br>GTTTGACATCCACGGAACCTCTCCGAAAAGAGAGGGTGCCCTTCGGGGAGCCGTGAGACAGGTGCTGCATGGCTGTCGT<br>CAGCTCGTGTCGTGAGATGTTGGGTTAAGTCCCGCAACGAGCGCAACCCCTATGGATAGTTGCCAGCAAGTAATGTTGGG<br>CACTCTATTCAGACTGCCCGGGTTAACCGGGAGGAAGGTGGGGACGACGTCAAGTCATCATGGCCCTTACGCCTAGGGC<br>TACACACGTACTACAATGGCGCGCACAAAGGGGAGCGAGACCGCGAGGTGGAGCCAATCCCAAAAAACGCGTCCCAGTC<br>CGGATTGCAGTCTGCAACTCGACTGCATGAAGTCGGAATCGCTAGTAATTCGAGATCAGCATGCTCGGGTGAATGCGTTCC<br>CGGGCCTTGTACACACCGCCCGTCACACCACGAAAGTCGGTTTTACCCGAAGTCGGTGAGCCAACCAGCAATGGAGGCA<br>GCCGCCTACGGTAGGGCTGATGATTGGGGTG
143	Selenomonas sp. OTU_669	CCCTCTCGCTGCTCTCTGTCCGTCCCATTGTAGTACGTGTGTAGCCCAGGTCATAAGGGGCATGATGACTTGACGTCATC<br>CCCGCCTTCCTCCGCGTTCTCCGCGGCAGTCTCCTTTGAGTGCCCACCACTACATGCTGGCAACAAAGGACAGGGGTTG<br>CGCTCGTTGCGGGACTTAACCCAACATCTCACGACACGAGCTGACGACAGCCATGCACCACCTGTTTTCTTGTCTCCGAA<br>GAGAGGGACGTATCTCTACGCCTTTCAATCAATGTCAAGACCTGGTAAGGTTCTTCGCGTTGCTTCGAATTAAACCACATAC<br>TCCACCGCTTGTGCGGGTCCCCGTCAATTCCTTTGAGTTTCAGTCTTGCGACCGTACTCCCCAGGCGGGATACTTATTGC<br>GTTAACTCCGGCACGGTAGGGGTCGATACCTCCCACACCTAGTATCCATCGTTTACGGCCAGGACTACCGGGGTATCTAA<br>TCCCGTTCGCTCCCCTGGCTTTCGAGCCTCAGCGTCAGTTGCAGTCCAGAAAGCCGCCTTCGCCACTGGTGTTCCTCCC<br>AATATCTACGCATTTCACCGCTACACTGGGAATTCCGCTTTCCTCTCCTGCACTCAAGCCATATAGTTTCTTGCCCATCAC<br>GGGGTTGAGCCCCGAACTTTTAAGCAAGACTTACATGGCCGCCTGCGCTCCCTTTACGCCCAATGATTCCGGACAACGCT<br>CGCCACCTACGTATTACCGCGGCTGCTGGCACGTAGTTAGCCGTGGCTTCCTCAAAAGGTACCGTCATTACATAAGCTTAT<br>TCAACTTATGTACGTTCGTCCCCTTCAACAGAGCTTTACGATCCGAAGACCTTCTTCACTCACGCGGCGTTGCTCCGTCAG<br>GCTTGCGCCCATTGCGGAAGATTCCCCACTGCTGCCTCCCGTAGGAGTCTGGGCCGTGTCTCAGTCCCAATGTGGCCGT<br>TCATCCTCTCAGACCGGCTACTGATCGTCGCCTAGGTAAGCCGTTACCTTACCTACCAGCTAATCAGACGCAGACCCATC<br>GATAGGTGATAGCTTATATATAGAGGCCATCTTTCATATAGGGGAGATGCCTCCCCTACACGCCATTCGGTATTAGCATTCC<br>TTTCGGAATGTTGTCCCCATCCTATCGGCAGGTTGTCTACGTGTTACTCACCCGTTTGCCACT
144	Centipeda sp. OTU_695	GACGAACGCTGGCGGCGTGCTTAACACATGCAAGTCGAACGGGATGATCTTAAAGCTTGCTTTTAGAGAATCTAGTGGCAA<br>ACGGGTGAGTAACACGTAGACAACCTGCCGACAGGATGGGGACAACATTCCGAAAGGAATGCTAATACCGAATGAAGCGGA<br>GGAGAGGCATCTCTCTTCCGTGAAAGATGGCCTCTTTTTATGCTATCACCTGTTGATGGGTCTGCGTCTGATTAGCTAGTT<br>GGTGAGGTAACGGCTCACCAAGGCGACGATCAGTAGCCGGTCTGAGAGGATGAACGGCCACATTGGGACTGAGACACGG<br>CCCAGACTCCTACGGGAGGCAGCAGTGGGGAATCTTCCGCAATGGGCGCAAGCCTGACGGAGCAACGCCGCGTGAGTG<br>AAGAAGGTCTTCGGATCGTAAAGCTCTGTTGACGGGGACGAACGTGCGGAGTGCGAATAGCGCTTTGTAATGACGGTACCT<br>GTCGAGGAAGCCACGGCTAACTACGTGCCAGCAGCCGCGGTAATACGTAGGTGGCGAGCGTTGTCCGGAATCATTGGGC<br>GTAAAGGGAGCGCAGGCGGGCCGGTAAGTCTTACTTAAAAGTGCGGGGCTCAACCCCGTGATGGGAGAGAAACTATCGGT<br>CTTGAGTACAGGAGAGGAAAGCGGAATTCCCAGTGTAGCGGTGAAATGCGTAGATATTGGGAAGAACACCAGTGGCGAAGG<br>CGGCTTTCTGGACTGCAACTGACGCTGAGGCTCGAAAGCCAGGGGAGCGAACGGGATTAGATACCCCGGTAGTCCTGGC<br>CGTAAACGATGGATACTAGGTGTGGGAGGTATCGACCCCTACCGTGCCGGAGTTAACGCAATAAGTATCCCGCCTGGGGA<br>GTACGGCCGCAAGGCTGAAACTCAAAGGAATTGACGGGGACCCGCACAAGCGGTGGAGTATGTGGTTTAATTCGAAGCAA<br>CGCGAAGAACCTTACCAGGCCTTGACATTGACTGAAAGCGCTAGAGATAGTGCCCTCTCTTCGGAGACAGGAAAACAGGTG<br>GTGCATGGCTGTCGTCAGCTCGTGTCGTGAGATGTTGGGTTAAGTCCCGCAACGAGCGCAACCCCTATTGTTAGTTGCCA<br>TCATTGAGTTGGGCACTCTAGCGAGACTGCCGGTAATAAACCGGAGGAAGGTGGGGATGACGTCAAATCATCATGCCCCTT<br>ATGACCTGGGCTACACACGTGCTACAATGGCTGGTACAACGAGTCGCAAGCCGGTGACGGCAAGCTAATCTCTGAAAGCC<br>AGTCTCAGTTCGGATTGTAGGCTGCAACTCGCCTACATGAAGTCGGAATCGCTAGTAATCGCGGATCAGCACGCCGCGGT<br>GAATACGTTCCCGGGCCTTGTACACACCGCCCGTCACACCACGAGAGTTTGTAACACCCGAAGTCGGTGAGGTAACCGTA<br>AGGAGCCAGCCGCCTAAGGTGGGATAGATGATTGGGGTG

Sr no.	Species_OTUs	16S reference sequence
145	Bifidobacterium sp. OTU_700	GATGAACGCTGGCGGCGTGCTTAACACATGCAAGTCGAACGGGATCCCGGGGGTTCGCTTCCGGGTGAGAGTGGCGAAC<br>GGGTGAGTAATGCGTGACCGACCTGCCCCATACACCGGAATAGCTCCTGGAAACGGGTGGTAATGCCGGATGCTCCGGT<br>TGGATGCATGTCCTTCCGGGAAAGATTCTATCGGTATGGGATGGGGTCGCGTCCTATCAGCTTGATGGCGGGGTAACGGC<br>CCACCATGGCTTCGACGGGTAGCCGGCCTGAGAGGGCGACCGGCCACATTGGGACTGAGATACGGCCCAGACTCCTAC<br>GGGAGGCAGCAGTGGGGAATATTGCACAATGGGCGCAAGCCTGATGCAGCGACGCCGCGTGCGGGATGGAGGCCTTCG<br>GGTTGTAAACCGCTTTTGATCGGGAGCAAGCCCTTCGGGGTGAGTGTACCTTTCGAATAAGCACCGGCTAACTACGTGCC<br>AGCAGCCGCGGTAATACGTAGGGTGCAAGCGTTATCCGGAATTATTGGGCGTAAAGGGCTCGTAGGCGGTTCGTCGCGTC<br>CGGTGTGAAAGCCCATCGCTTAACGGTGGGTCTGCGCCGGGTACGGGCGGGCTGGAGTGCGGTAGGGGAGACTGGAAT<br>TCCCGGTGTAACGGTGGAATGTGTAGATATCGGGAAGAACACCAATGGCGAAGGCAGGTCTCTGGGCCGTCACTGACGCT<br>GAGGAGCGAAAGCGTGGGGAGCGAACAGGATTAGATACCCTGGTAGTCCACGCCGTAAACGGTGGATGCTGGATGTGGG<br>GCCCGTTCCACGGGTTCCGTGTCGGAGCTAACGCGTTAAGCATCCCGCCTGGGGAGTACGGCCGCAAGGCTAAAACTCA<br>AAGAAATTGACGGGGGCCCGCACAAGCGGCGGAGCATGCGGATTAATTCGATGCAACGCGAAGAACCTTACCTGGGCTTG<br>ACATGTTCCCGACAGCCGTAGAGATATGGCCTCCCTTCGGGGCGGGTTCACAGGTGGTGCATGGTCGTCGTCAGCTCGT<br>GTCGTGAGATGTTGGGTTAAGTCCCGCAACGAGCGCAACCCTTGCCCTATGTTGCCAGCACGTGATGGTGGGGACTCGT<br>GGGGGACTGCCGGGGTTAACTCGGAGGAAGGTGGGGATGACGTCAAATCATCATGCCCCTTATGTCTTGGGCTTCACGCA<br>TGCTACAATGGCTGGTACAGAGGGTTGCGATATCGTGAGGTGGAGCGAATCCCTTAAAGCCAGTCTCAGTTCGGATTGGG<br>GTCTGCAACTCGACCCCATGAAGGTGGAGTCGCTAGTAATCGCAGATCAGCAACGCTGCGGTGAATACGTTCTCGGGCCT<br>TGTACACACCGCCCGTCACGTCACGAAAGTTGGTAACACCCGAAGCCCATGGCCTAACCGTTTATCGGGGGGAGTGGTC<br>GAAGGTGGGATTGGCGATTGGGACG
146	Saccharibacteria sp. OTU_701	GATGAACGCTGGCGGCGTGCCTAACACATGCAAGTCGAGCGGCAGCGAGGGGTGCTTGCACCCTGTCGGCGAGCGGCG<br>GACGGCTGAGTAACGCGTGGGAACGTGCCCCAAAGTGAGGAATAACTGCCCGAAAGGGTAGCTAATACCGCATATTATCTT<br>CGGATCAAAGGATTTATCCGCTTTGGGAGCGGCTCGCGTCGGATTAGGTAGTTGGTGAGGTAATGGCTCACCAAGCCGAC<br>GATCCGTAGCTGGTCTGAGAGGATGACCAGCCAGACTGGAACTGAGACACGGTCCAGACTCCTACGGGAGGCAGCAGTG<br>AGGAATCTTCCACAATGGGCGAAAGCCTGATGGAGCAACGCCGCGTGCAGGACGAAGGCCTTCGGGTTGTAAACTGCTTT<br>TATAAGTGAGGAATATGACGGTAACTTATGAATAAGGATCGGCTAACTACGTGCCAGCAGCCGCGGTCATACGTAGGATCC<br>GAGCGTTATCCGGAGTGACTGGGCGTAAAGAGTTGCGTAGGCGGTTGTATAAGTGAATAGTGAAATCTGGTGGCTCAACCA<br>TACAGGCTATTGTTCAAACTGTACAACTTGAGAGTGGTAGAGGTCACTGGAATTTCTTGTGTAGGAGTGAAATCCGTAGATAT<br>AAGAAGGAACACCAATGGCGTAGGCAGGTGACTGGACCATTTCTGACGCTAAGGCACGAAAGCGTGGGGAGCGAACCGGA<br>TTAGATACCCGGGTAGTCCACGCCGTAAACGATGGATACTGGCTGTTGGAGGTATCGACCCCTTCAGTAGCGAAGCTAAC<br>GCGTTAAGTATCCCGCCTGTGGAGTACGGCCGCAAGGCTAAAACATAAAGGAATTGACGGGGACCCGCACAAGCGGTGGA<br>TCGTGTTCTTTAATTCGATGCTAAACGAAGAACCTTACCAGGGCTTGACATCCAGGGAATTTTTGGGAAACCAATTAGTGCC<br>GTTTGGAACCCTGTGACAGGTGATGCATGGCCGTCGTCAGCTCGTGTCGTGAGATGTTTGGTTAAGTCCATCAACGAGCG<br>CAACCCTTATTGTTAGTTGCCATCATTAAGTTGGGCACTCTAGCGAGACTGCCGGTAATAAACCGGAGGAAGGTGGGGATG<br>ACGTCAAATCATCATGCCCCTTATGACCTGGGCTACACACGTGCTACAATGGTCGGTACAACGAGTTGCGAGCCGGTGAC<br>GGCAAGCTAATCTCTGAAAGCCGATCTCAGTTCGGATTGGAGGCTGCAACTCGCCTCCATGAAGTCGGAATCGCTAGTAAT<br>CGCGGATCAGCACGCCGCGGTGAATACGTTCCCGGGCCTTGTACACACCGCCCGTCACACCACGAGAGTTTGTAACACC<br>CGAAGTCGGTGAGGTAACCTTTTAGGGGCCAGCCGCCTAAGGTGGGATGGATGATTGGGGTG
147	Streptococcus sp. OTU_702	GACGAACGCTGGCGGCGTGCCTAATACATGCAAGTGGGACGCAAGGAAACACACTGTGCTTGCACACCGTGTTTTCTTGA<br>GTCGCGAACGGGTGAGTAACGCGTAGGTAACCTGCCTATTAGCGGGGGATAACTATTGGAAACGATAGCTAATACCGCATA<br>ATATTAATTATTGCATGATAATTGATTGAAAGATGCAAGCGCATCACTAGTAGATGGACCTGCGTTGTATTAGCTAGTTGGTAA<br>GGTAAGAGCTTACCAAGGCGACGATACATAGCCGACCTGAGAGGGTGATCGGCCACACTGGGACTGAGACACGGCCCAG<br>ACTCCTACGGGAGGCAGCAGTAGGGAATCTTCGGCAATGGACGAAAGTCTGACCGAGCAACGCCGCGTGAGTGAAGAAGG<br>TTTTCGGATCGTAAAGCTCTGTTGTAAGTCAAGAACGTGTGTGAGAGTGGAAAGTTCACACAGTGACGGTAGCTTACCAGAA<br>AGGGACGGCTAACTACGTGCCAGCAGCCGCGGTAATACGTAGGTCCCGAGCGTTGTCCGGATTTATTGGGCGTAAAGGGA<br>GCGCAGGCGGTCAGGAAAGTCTGGAGTAAAAGGCTATGGCTCAACCATAGTGTGCTCTGGAAACTGTCTGACTTGAGTGCA<br>GAAGGGGAGAGTGGAATTCCATGTGTAGCGGTGAAATGCGTAGATATATGGAGGAACACCAGTGGCGAAAGCGGCTCTCTG<br>GTCTGTCACTGACGCTGAGGCTCGAAAGCGTGGGTAGCGAACAGGATTAGATACCCTGGTAGTCCACGCCGTAAACGATG<br>AGTGCTAGGTGTTAGGCCCTTTCCGGGGCTTAGTGCCGGAGCTAACGCAATAAGCACTCCGCCTGGGGAGTACGACCGC<br>AAGGTTGAAACTCAAAGGAATTGACGGGGGCCCGCACAAGCGGTGGAGCATGTGGTTTAATTCGAAGCAACGCGAAGAACC<br>TTACCAGGTCTTGACATCCCGATGCTATTCTTAGAGATAGGAAGTTACTTCGGTACATCGGAGACAGGTGGTGCATGGTTGT<br>CGTCAGCTCGTGTCGTGAGATGTTGGGTTAAGTCCCGCAACGAGCGCAACCCTTATCCTTACTTGCCAGCACTTAGGGTG<br>GGAACTGTAAGGAGACTGCCGGTGACAAGCCGGAGGAAGGTGGGGATGACGTCAAGTCATCATGGCCCTTACGGCCTGG<br>GCTACACACGTGCTACAATGGTTGGTACAGACGGTAGCGAAGCCGCGAGGTGGAGCCAATCTGAGAAAGCCGATCGTAGT<br>CCGGATTGCAGTCTGCAACTCGACTGCATGAAGTCGGAATCGCTAGTAATCGCGAATCAGCATGTCGCGGTGAATACGTTC<br>CCGGGTCTTGTACACACTGCCCGTCACACCATGGGAGTTTGTTGCACCAGAAGCAGGTAGCTTAACGAAAGAGGGCGCTT<br>GCCACGGTGTGGCCGATGACTGGGGTG
148	Cardiobacterium sp. OTU_706	GACGAACGCTGGCGGCGTGCCTAATACATGCAAGTGGGACGCAAGGAAACACACTGTGCTTGCACACCGTGTTTTCTTGA<br>GTCGCGAACGGGTGAGTAACGCGTAGGTAACCTGCCTATTAGCGGGGGATAACTATTGGAAACGATAGCTAATACCGCATA<br>ATATTAATTATTGCATGATAATTGATTGAAAGATGCAAGCGCATCACTAGTAGATGGACCTGCGTTGTATTAGCTAGTTGGTAA<br>GGTAAGAGCTTACCAAGGCGACGATACATAGCCGACCTGAGAGGGTGATCGGCCACACTGGGACTGAGACACGGCCCAG<br>ACTCCTACGGGAGGCAGCAGTAGGGAATCTTCGGCAATGGACGAAAGTCTGACCGAGCAACGCCGCGTGAGTGAAGAAGG<br>TTTTCGGATCGTAAAGCTCTGTTGTAAGTCAAGAACGTGTGTGAGAGTGGAAAGTTCACACAGTGACGGTAGCTTACCAGAA<br>AGGGACGGCTAACTACGTGCCAGCAGCCGCGGTAATACGTAGGTCCCGAGCGTTGTCCGGATTTATTGGGCGTAAAGGGA<br>GCGCAGGCGGTCAGGAAAGTCTGGAGTAAAAGGCTATGGCTCAACCATAGTGTGCTCTGGAAACTGTCTGACTTGAGTGCA<br>GAAGGGGAGAGTGGAATTCCATGTGTAGCGGTGAAATGCGTAGATATATGGAGGAACACCAGTGGCGAAAGCGGCTCTCTG<br>GTCTGTCACTGACGCTGAGGCTCGAAAGCGTGGGTAGCGAACAGGATTAGATACCCTGGTAGTCCACGCCCTAAACGATG<br>TCAACTAGGCGTCGGGTTGTATAGACTCGGTGCCGCAGCTAACGCATTAAGTTGACCGCCTGGGGAGTACGGCCGCAAG<br>GTTGAAACTCAAAGAAATTGACGGGGACCCGCACAAGCGGTGGAGCATGTGGTTTAATTCGATGCAACGCGAAGAACCTTA<br>CCAGGCCTTGACATCCAGAGAACTTAGCAGAGATGCTTTGGTGCCTTCGGGAACTCTGAGACAGGTGTTGCATGGCTGTC<br>GTCAGCTCGTGTCGTGAGATGTTGGGTTAAGTCCCGCAACGAGCGCAACCCTTATCCTTACTTGCCAGCACTTAGGGTGG<br>GAACTGTAAGGAGACTGCCGGTGACAAGCCGGAGGAAGGTGGGGATGACGTCAAGTCATCATGGCCCTTACGGCCTGGG<br>CTACACACGTGCTACAATGGTTGGTACAGACGGTAGCGAAGCCGCGAGGTGGAGCCAATCTGAGAAAGCCGATCGTAGTC<br>CGGATTGCAGTCTGCAACTCGACTGCATGAAGTCGGAATCGCTAGTAATCGCGAATCAGCATGTCGCGGTGAATACGTTCC<br>CGGGTCTTGTACACACTGCCCGTCACACCATGGGAGTTTGTTGCACCAGAAGCAGGTAGCTTAACGAAAGAGGGCGCTTG<br>CCACGGTGTGGCCGATGACTGGGGTG

Sr no.	Species_OTUs	16S reference sequence
149	Tannerella sp. OTU_716	GATGAACGCTAGCGATAGGCTTAACACATGCAAGTCGAGGGGCAGCATGATCTTAGCTTGCTAAGGTCGATGGCGACCGGCGCACGGGTGCGTAACGCGTATGCAACTTGCCTTACAGAGGGGGATAACCCGTTGAAAGACGGACTAAAACCGCATACACTTGTATTATTGCATGATATTACAAGGAAATATTTATAGCTGTAAGATAGGCATGCGTCCCATTAGCTGGTTGGTGAGGTAACGGCTCACCAAGGCGACGATGGGTAGGGGAACTGAGAGGTTTATCCCCCACACTGGTACTGAGACACGGACCAGACTCCTACGGGAGGCAGCAGTGAGGAATATTGGTCAATGGGCGAGAGCCTGAACCAGCCAAGTCGCGTGAAGGAAGACTGTCCTAAGGATTGTAAACTTCTTTTATACGGGAATAACGGGCGATACGAGTATTGCATTGAATGTACCGTAAGAATAAGCATCGGCTAACTCCGTGCCAGCAGCCGCGGTAATACGGAGGATGCGAGCGTTATCCGGATTTATTGGGTTTAAAGGGTGCGTAGGTGGGCTGTTAAGTCCGCGGTGAAAGTTTGTCGCTTAACGATAAAATTGCCGTTGAAACTGGTAGTCTTGAGTATAGATGAAGTAGGCGGAATGCGTGGTGTAGCGGTGAAATGCATAGAGATCACGCAGAACTCCGATTGCGAAGGCAGCTTACTAAGGTATAACTGACACTGAAGCACGAAAGCGTGGGTATCAAACAGGATTAGATACCCTGGTAGTCCACGCAGTAAACGATGATTACTAGGAGTTTGCGATATAGTGTAAGCTCTACAGCGAAAGCGTTAAGTAATCCACCTGGGGAGTACGCCGGCAACGGTGAAACTCAAAGGAATTGACGGGGGCCCGCACAAGCGGAGGAACATGTGGTTTAATTCGATGATACGCGAGGAACCTTACCCGGGATTGAAATGTAGACGACGGAGAGTGAGAGCTTTCTTCCCTTCGGGGCGTCTATGTAGGTGCTGCATGGTTGTCGTCAGCTCGTGCCGTGAGGTGTCGGCTTAAGTGCCATAACGAGCGCAACCCTGACTGTCAGTTGCTAACAGGTAAAGCTGAGGACTCTGGCGGGACTGCCGGCGTAAGCTGTGAGGAAGGTTGGGATGACGTCAAATCAGCACGGCCCTTACATCCGGGGCGACACACGTGTTACAATGGCAGGGACAAAGGGCAGCTACCGGGCGACCGGATGCCAATCTCCAAACCCTGTCTCAGTTCGGATCGGAGTCTGCAACTCGACTCCGTGAAGCTGGATTCGCTAGTAATCGCGCATCAGCCATGGCGCGGTGAATACGTTCCCGGGCCTTGTACACACCGCCCGTCAAGCCATGGGAGCCGGGGGTACCTGAAGTCCGTAACCGCGAGGATCGGCCTAGGGTAAAACTGGTGACTGGGGCT
150	Alloprevotella sp. OTU_722	GATGAACGCTAGCTACAGGCTTAACACATGCAAGTCGTGGGGCAGCGGATACTTAGCTTGCTAAGTATGCCGGCGACCGGCGCACGGGTGAGTAACGCGTACCGAACCTGCCCATCACACAGGGATAGGCTTGCGAAAGCAAGATTAATACCTGATGGTCTCAGTTGTATGCATGTATGATTGAGTAAAGCCTTCGGGCGGTGATGGATGGCGGTGCGTCCCATTAGGAAGTTGGCGGGGTAACGGCCCACCAATCCTTCGATGGGTAGGGGTTCTGAGAGGAAGGTCCCCCACATTGGAACTGAGATACGGTCCAAACTCCTACGGGAGGCAGCAGTGAGGAATATTGGTCAATGGGCGAGAGCCTGAACCAGCCAAGTAGCGTGCGGGATGAAGGCCCTTGTGGTCGTAAACCGCTTTTATCAGTGAATAAAGTGCACCACGTGTGGTGTCATTGCAGGTAGCTGAAGAAAAAGGACCGGCTAATTCCGTGCCAGCAGCCGCGGTAATACGGAAGGTCCGGGCGTTATCCGGAATTATTGGGTTTAAAGGGAGCGCAGGCGGGAGTGTAAGTCAGCTGTTAAATATCAGGGCCCAACTCTGTTATGCAGTTGAAACTATATTTCTTGAGTACGCACAGGGATGGTGGAATTCAGGGTGTAGCGGTGAAATGCTTAGATATCCTGAAGAACTCCGATCGCGAAGGCAGCCATCCGGAGCGTAACTGACGCTGAGGCTCGAAGGTGCGGGTATCGAACAGGATTAGATACCCTGGTAGTCCGCACGGTAAACGATGAATACTCGCAGTTCGGCCGTTTAGGTCGAGTTGTCCAGCGAAAGCGTTAAGTATTCCACCTGGGGAGTACGCCGGCAACGGTGAAACTCAAAGGAATTGACGGGGGCCCGCACAAGCGGAGGAACATGTGGTTTAATTCGATGATACGCGAGGAACCTTACCCGGGCTTGAATTGCAGTCGCTGCCCACAGAGACGTGGGCTTCCCTTCGGGGCGTCTGTGAAGGTGCTGCATGGTTGTCGTCAGCTCGTGCCGTGAGGTGTCGGCTTAAGTGCCATAACGAGCGCAACCCCCTTTCCCAGTTGCCATCGGGTGATGCCGGGCACTCTGGGGACACTGCCACCGCAAGGTGCGAGGAAGGTGGGGATGACGTCAAATCAGCACGGCCCTTACGTCCGGGGCTACACACGTGTTACAATGGCCGGTACAGAGGGACGGTGCAATGCAAGTTGCATCCAATCTTGAAATCCGGTCCCAGTTCGGACTGGGGTCTGCAACCCGACCCCACGAAGCTGGATTCGCTAGTAATCGCGCATCAGCCATGGCGCGGTGAATACGTTCCCGGGCCTTGTACACACCGCCCGTCAAGCCATGAAAGCCGGGGGTGCCTGAAGTTCGTGACCGCGAGGAGCGACCTAGGGCAAGACCGGTGATTGGGGCT
151	Porphyromonas sp. OTU_726	GATGAACGCTAGCGATAGGCTTAACACATGCAAGTCGAGGGGCAGCATGATCTTAGCTTGCTAAGGTTGATGGCGACCGGCGCACGGGTGCGTAACGCGTATGCAACTTGCCTTACAGAGGGGGATAACCCGTTGAAAGACGGACTAAAACCGCATACACTTGTATTATTGCATGATATTACAAGGAAATATTTATAGCTGTAAGATAGGCATGCGTCCCATTAGCTGGTTGGTGAGGTAACGGCTCACCAAGGCGACGATGGGTAGGGGAACTGAGAGGTTTATCCCCCACACTGGTACTGAGACACGGACCAGACTCCTACGGGAGGCAGCAGTGAGGAATATTGGTCAATGGGCGAGAGCCTGAACCAGCCAAGTCGCGTGAAGGAAGACAGTCCTAAGGATTGTAAACTTCTTTTATACGGGAATAACGGGCGATACGAGTATTGCATTGAATGTACCGTAAGAATAAGCATCGGCTAACTCCGTGCCAGCAGCCGCGGTAATACGGAGGATGCGAGCGTTATCCGGATTTATTGGGTTTAAAGGGTGCGTAGGTTGTTCGGTAAGTCAGCGGTGAAACCTGAGCGCTCAACGTTCAGCCTGCCGTTGAAACTGCCGGGCTTGAGTTCAGTGGCGGCAGGCGGAATTCGTGGTGTAGCGGTGAAATGCATAGATATCACGAGGAACTCCGATTGCGAAGGCAGCTTGCCATACTGCGACTGACACTGAAGCACGAAGGCGTGGGTATCAAACAGGATTAGATACCCTGGTAGTCCACGCAGTAAACGATGATTACTAGGAGTTTGCGATATACCGTCAAGCTTCCACAGCGAAAGCGTTAAGTAATCCACCTGGGGAGTACGCCGGCAACGGTGAAACTCAAAGGAATTGACGGGGGCCCGCACAAGCGGAGGAACATGTGGTTTAATTCGATGATACGCGAGGAACCTTACCCGGGCTTGAATTGCAGACGTACGATTCAGAGATGATGAGGCCCTTCGGGGCGTCTGCGAAGGTGCTGCATGGTTGTCGTCAGCTCGTGCCGTGAGGTGTCGGCTCAAGTGCCATAACGAGCGCAACCCCCCTCCCCAGTTGCCATCGGGTGATGCCGGGCACTCCGGGGACACTGCCGCCGCAAGGTGCGAGGAAGGTGGGGACGACGTCAAATCAGCACGGCCCTTACGTCCGGGGCTACACACGTGTTACAATGGCCGGTACAGAGCGTCGGCCGCGCGCAAGTGCGGTCCAATCCTCAAAGCCGGTCCCAGTTCGGACTGGGGTCTGCAACCCGACCCCACGAAGCTGGATTCGCTAGTAATCGCGCATCAGCCATGGCGCGGTGAATACGTTCCCGGGCCTTGTACACACCGCCCGTCAAGCCATGAAAGCCGGGGGCGCCTGAAGTCCGTGACCGCGAGGGTCGGCCTAGGGCGAAACCGGTGATTGGGGCT
152	Bacteroidales gen. sp. OTU_727	GATGAACGCTAGCGGCAGGCCTAACACATGCAAGTCGAGCGGTAGGCGCGCCCCTCGGGGCGCGCACGAGCGGCGGACGGGTGAGTAGCGCGTATGCAACCTGCCCGGCCCCGGGGTCCAGCCCGCCGAAAGGCGGAGTGATCCCCCGGGATCCGCGGGCGCCGCCTGGCGCCCGCGGGGTAGGCCCCCCCTACGGGGGGGCTGGGGCCGGATGGGCATGCGTGCCATTAGCTAGTAGGCGGGGCAACGGCCCACCTAGGCGACGATGGCTCGGGGGCCCGGGAGGGCGGCCCCCCACACTGGGACTGAGACACGGCCCAGACTCCTACGGGAGGCAGCAGTGGGGAATATTGGTCAATGGGCGGGAGCCTGAACCAGCCATGCCGCGTGCCGGATGGAGGCCCTAGGGGTCGTGAACGGCTTTTCCGGGGGAGGAATAGGCCCCACGTGTGGGGCGATGCCCGTACCCCGGGAATAAGGATCGGCTAACCCCGTGCCAGCAGCCGCGGTAATACGGGGGATCCGAGCGTTATCCGGATTTACTGGGTTTAAAGGGCGCGCAGGCGGGGGCGCGGGTCGGCGGTGAAACCCCGGGGCTCAACCCCGGGCATGCCGCCGAAACCTCGCCCCTTGGAGGGGGCCGGGGCCGGCGGAACGCGTGGTGTAGCGGTGAAATGCGTAGATATCACGTGGAAGGCCGATCGCGGAGGCAGCTGGCCAGGCCCCCCTCGACGCTGAGGCGCGACAGCGTGGGGATCGAACGGGATTAGATACCCCGGTAGTCCACGCCGTAAACGATGGGCACTGGCCCCCGGGGCCGCTGGGCCTCGGGGGCTGCGCGAAAGCATTAAGTGCCCCACCTGGGGAGTACGGCCGCAAGGCTGAAACTCAAAGGAATTGACGGGGGCCCGCACAAGCGGAGGAACATGTGGTTTAATTCGATGGTACGCGAGGAACCTTACCCGGGCTCGAACGCCGGCCGGAGGCCCCCGAGAGGGGGCCGCCCCACGGGGCGGCCGGCGAGGTGCTGCATGGTTGTCGTCAGCTCGTGCCGTGAGGTGTCGCGTTAAGTCGCATAACGAGCGCAACCCCCCGCCGCCAGTTGCCAGCGCGTGAGGGCGGGGACTCTGGCGGGACTGCCGGCGCAAGCCGCGAGGAGGGCGGGGACGACGTCAAATCAGCACGGCCCCCACGTCCGGGGCGACACACGTGTTACAGTGGCCGGCACAGCGGGCCGCCACCCCGCGAGGGGGCGCGAATCCCGAAAGCCGGCCCCGGTTCGGATCGGGGTCTGCAACCCGACCCCGTGAAGCTGGATTCGCTAGTAATCGCGCATCAGCCACGGCGCGGTGAATACGTTCCCGGGCCTTGTACACACCGCCCGTCAAGCCATGGGAGCCGGGGGCGCCTGAAGCCCGCGCCCGGGAGGGGCGGTCGAGGGCGAGCCCGGCGACTGGGGCT

Sr no.	Species_OTUs	16S reference sequence
153	Alloprevotella sp. OTU_728	GATGAACGCTAGCTACAGGCTTAACACATGCAAGTCGTGGGGCAGCGGATACTTAGCTTGCTAAGTATGCCGGCGACCGGCGCACGGGTGAGTAACGCGTACCGAACCTGCCCATCACACAGGGATAGGCTTGCGAAAGCAAGATTAATACCTGATGGTCTCAGTTGTATGCATGTATGATTGAGTAAAGCCTTCGGGCGGTGATGGATGGCGGTGCGTCCCATTAGGAAGTTGGCGGGGTAACGGCCCACCAATCCTTCGATGGGTAGGGGTTCTGAGAGGAAGGTCCCCCACATTGGAACTGAGATACGGTCCAAACTCCTACGGGAGGCAGCAGTGAGGAATATTGGTCAATGGGCGAGAGCCTGAACCAGCCAAGTAGCGTGCGGGATGAAGGCCCTTGTGGTCGTAAACCGCTTTTATCAGTGAATAAAGTGCATCACGTGTGGTGTCATTGCAGGTAGCTGAAGAAAAAGGACCGGCTAATTCCGTGCCAGCAGCCGCGGTAATACGGAAGGTCCGGGCGTTATCCGGAATTATTGGGTTTAAAGGGAGCGCAGGCGGGAGTATAAGTCAGCTGTTAAATATCAGAGCCCAACTCTGTTATGCAGTTGAAACTATATTTCTTGAGTACGCACAGGGATGGCGGAATTCAGGGTGTAGCGGTGAAATGCTTAGATATCCTGAAGAACTCCGATCGCGAAGGCAACCGCCGGGAACTCCGTGGATACTGCCGCCGCAAGGCGGAGGAAGGTGGGGATGACGTCAAATCAGCACGGCCCTTACGTCCGGGGCTACACACGTGTTACAATGGTCGGAGCAGCAGGAAGCTACCTGGCGACAGGATGCTGATCCCGAAACCCGGCCTCAGTTCGGACTGGAGTCTGCAACCCGACTCCACGAAGCCGGATTCGCTAGTAATCGCGCATCAGCCATGGCGCGGTGAATACGTTCCCGGGCCTTGTACACACCGCCCGTCAAGCCATGAAAGCCGGGGGTGCCTGAAAGCCGTGACCGCGAGGGTCGGCCTAGGGTAAAACCGGTGATTGGGGCT
154	Neisseria sp. OTU_735	ATTGAACGCTGGCGGCATGCTTTACACATGCAAGTCGGACGGCAGCGGGGTAGTGCTTGCACTACTGCCGGCGAGTGGCGAACGGGTGAGTAACATATCGGAACGTACCGAGCAGTGGGGGATAACTAATCGAAAGATTAGCTAATACCGCATATCTTCTGAGGAAGAAAGCAGGGGACCTTTGGGCCTTGCGCTGTTTGAGCGGCCGATATCTGATTAGCTGGTTGGTGGGGTAAAGGCCTACCAAGGCGACGATCAGTAGCGGGTCTGAGAGGATGATCCGCCACACTGGGACTGAGACACGGCCCAGACTCCTACGGGAGGCAGCAGTGGGGAATTTTGGACAATGGGGGCAACCCTGATCCAGCCATGCCGCGTGTCTGAAGAAGGCCTTCGGGTTGTAAAGGACTTTTGTCCGGGAAGAAAAGCGCGATGTTAATACCATTGCGTGCTGACGGTACCGGAAGAATAAGCACCGGCTAACTACGTGCCAGCAGCCGCGGTAATACGTAGGGTGCGAGCGTTAATCGGAATTACTGGGCGTAAAGCGGGCGCAGACGGTTACTTAAGCAGGATGTGAAATCCCCGGGCTCAACCTGGGAATTGCGTTCTGAACTGGGTGGCTAGAGTGTGTCAGAGGGGGGTAGAATTCCACGTGTAGCAGTGAAATGCGTAGAGATGTGGAGGAATACCGATGGCGAAGGCAGCCCCCTGGGATAGCACTGACGTTCATGCCCGAAAGCGTGGGTAGCAAACAGGATTAGATACCCTGGTAGTCCACGCCCTAAACGATGTCGATTAGCTGTTGGGCAACTTGATTGCTTGGTAGCGAAGCTAACGCGTGAAATCGACCGCCTGGGGAGTACGGTCGCAAGATTAAAACTCAAAGGAATTGACGGGGACCCGCACAAGCGGTGGATGATGTGGATTAATTCGATGCAACGCGAAGAACCTTACCTGGTCTTGACATGTACGGAACCTTCCGGAGACGGAAGGGTGCCTTCGGGAGCCGTAACACAGGTGCTGCATGGCTGTCGTCAGCTCGTGTCGTGAGATGTTGGGTTAAGTCCCGCAACGAGCGCAACCCCTATTGTTAGTTGCCATCATTGAGTTGGGCACTCTAGCGAGACTGCCGGTAATAAACCGGAGGAAGGTGGGGATGACGTCAAATCATCATGCCCCTTATGACCTGGGCTACACACGTGCTACAATGGCTGGTACAACGAGTCGCAAGCCGGTGACGGCAAGCTAATCTCTGAAAGCCAGTCTCAGTTCGGATTGTAGGCTGCAACTCGCCTACATGAAGTCGGAATCGCTAGTAATCGCGGATCAGCACGCCGCGGTGAATACGTTCCCGGGCCTTGTACACACCGCCCGTCACACCACGAGAGTTTGTAACACCCGAAGTCGGTGAGGTAACCGTAAGGAGCCAGCCGCCTAAGGTGGGATAGATGATTGGGGTG
155	Saccharibacteria sp. OTU_737	GATAAACGCTGGCGGCGTGCCTAATACATGCAAGTCGTGCGGAAAGGTTTCTCACTTTGGTTCGAAACATTTCACATTTCACATTGACCATTTCACATTTATTTAACAGGTATCATTTTTCATTTTTTTTGCTGAGAGGCATTTCAATGATAATTGGTAAATTAGAAATTAATGAGAATTGTAAAATGAAAAATGAGAAATACTATTTCGGATTAGTGTGAGGAATACTCGAGCGGCGGACGGCAGAGTAACGCGTAGGAATGTACCCCAAAGTGAGGAATAAGCACCGGAAACGGTGTCTAATACCGCATAATATCTTCGGATTAAAGCAGCAATGCGCTTTGGGAACAGCCTGCGTCCGATTAGCTTGTTGGTGAGGTAATGGCTCACCAAGGCTACGATCGGTAGCTGGTCTGAGAGGATGATCAGCCTGACTGGAACTGAGACACGGTCCAGACTCCTACGGGAGGCAGCAGTAGGGAATTTTCCACAATGGGCGCAAGCCTGATGGAGCAACGCCGCGTGCAGGATGAAGGCTTTAGGGTCGTAAACTGCTTTTATGTGCGATGAATTTGACAGTAACACATGAATAAGGATCGGCTAACTACGTGCCAGCAGCCGCGGTCATACGTAGGATCCAAGCGTTATCCGGAATTACTGGGCGTAAAGAGTTGCGTATGTGGCATTGTAAGCAAGACATGAAATCGTGTGGCTCAACCATACGGCTATGTCTTGAACTGCAAAGCTTTGAGAGCGAGAGGGGTAGATGGAATTACTAGTGTAGGAGTGAAATCCGTAGATATTAGTAGGAACACCGATAGCGTAGGCAGTCTACTGGCTCGTTTCTGACACTGAGGCACGAAAGCGTGGGGAGCAAATGGGATTAGATACCCCAGTAGTCCACGCCGTAAACTATGGATGCTAGCTGTGAACCGTATCGACCCGGTTCGTAGCGAAGCTAACGCGTTAAGCATCCCGCCTGTGGAGTACGGCCGCAAGGCTAAAACATAAAGGAATTGACGGGGACCCGCACAAGCGGTGGAGCGTGTTGTTTAATTCGATGCTAAACGAAGAACCTTACCCAGGCTTGACATCCCGAGAAGTTTGTCGAAAGACAAATGTGCTTTATTGAACTCGGTGACAGGTGTTGCATGGCCGTCGTCAGCTCGTGTCGTGAGATGTTAGGTTAAGTCCTTCAACGAGCGCAACCCTTATAGTTAGTTGAATTTTTCTAGCTAGACTGCCCTGGTAACAGGGAGGAAGGAGGGGATGACGCCAGGTCAGTATTACCCTTATGTCTGGGGCTACAAACACGCTACAATGGCCGGTACAAAGGGCAGCCAACCCGCGAGGGGGAGCTAATCCCATCAAAGCCGGTCTCAGTTCGGATTGCAGGCTGAAACTCGCCTGCATGAAGCTGGAATCGCTAGTAATGGTAGGTCAGCTATACTACCGTGAATACGTTCCCGGGTCTTGTACACACCGCCCGTCAAACCATGAAAGTGGCCAACACCCGAAGTCCAGATTTTTCTGGCCTAAGGTGGGGGACATGATTGGGGTT
156	Prevotellaceae gen. sp. OTU_751	GATGAACGCTAGCTACAGGCCTAACACATGCAAGTCGTGGGGCAGCGGGTATGTTTGTTTTCAAGCATATGCCGGCGACCGGCGCACGGGTGAGTAACGCGTATCCAACCTGCCCATCACAAGGGAATAACCCGTCGAAAGTCGGCCTAATGCCCTATGTATTCCGATGAAGTCCTCTGAATCGGAATAAAGCATTTGCGGTGATGGATGGGGATGCGTCTGATTAGCTTGTCGGCGGGGTAACGGCCCACCGAGGCAACGATCAGTAGGGGTTCTGAGAGGAAGGTCCCCCACATTGGAACTGAGACACGGTCCAAACTCCTACGGGAGGCAGCAGTGAGGAATATTGGTCAATGGGCGTGAGCCTGAACCAGCCAAGTAGCGTGCAGGATGACGGCCCTATGGGTTGTAAACTGCTTTTATACGGGGATAAAAGGGTGAACGTGTTCTCCTTTGCAGGTACCGTATGAATAAGGACCGGCTAATTCCGTGCCAGCAGCCGCGGTAATACGGAAGGTCCTGGCGTTATCCGGATTTATTGGGTTTAAAGGGAGCGCAGGCTGCACTTTAAGCGTGTTGTGAAATGTACCGGCTCAACCGGTAACGTGCAGCGCGAACTGGGGTGCTTGAGTACGAAGAGGGAAAGCGGAACTCGTGGTGTAGCGGTGAAATGCTTAGATATCACGAGGAACTCCGATCGCGAAGGCAGCTTTCCGTTTCGGTACTGACGCTGAGGCTCGAAAGTGCGGGTATCGAACAGGATTAGATACCCTGGTAGTCCGCACGGTAAACGATGGATGCTCGCTGTTGGATATTTTTATTCAGTGGCTAAGTGAAAACGTTAAGCATCCCACCTGGGGAGTACGCCGGCAACGGTGAAACTCAAAGGAATTGACGGGGGCCCGCACAAGCGGAGGAACATGTGGTTTAATTCGATGATACGCGAGGAACCTTACCCGGGCTTGAACCAGGAGTGACGAGTGCAGAGATGCGCTTATTCTTCGGACACTTTTGGAGGTGCTGCATGGTTGTCGTCAGCTCGTGCCGTGAGGTGTCGGCTTAAGTGCCATAACGAGCGCAACCCTTTTCTACGTTTGCCATCGGGTAATGCCGGGAACTCCGTGGATACTGCCGCCGCAAGGCGGAGGAAGGTGGGGATGACGTCAAATCAGCACGGCCCTTACGTCCGGGGCTACACACGTGTTACAATGGTCGGAGCAGCAGGAAGCTACCTGGCGACAGGATGCTGATCCCAAAACCCGGCCTCAGTTCGGACTGGAGTCTGCAACCCGACTCCACGAAGCCGGATTCGCTAGTAATCGCGCATCAGCCATGGCGCGGTGAATACGTTCCCGGGCCTTGTACACACCGCCCGTCAAGCCATGAAAGCCGGGGGTGCCTGAAAGCCGTGACCGCGAGGGTCGGCCTAGGGTAAAACCGGTGATTGGGGCT

Sr no.	Species_OTUs	16S reference sequence
157	Saccharibacteria sp. OTU_778	GATGAACGCTGGCGGCGTGCCTAATACATGCAAGTCGAGCGGCAGCACGGTCTTCGGACTGGTGGCGAGCGGCGGACG<br>GCAGAGTAACGCGTAGGAATATACCCCAAAGTGAGGGATAAGCTCCAGAAATGGAGTCTAATACCGCATATGGTCTTCGGAT<br>TAAAGCCTTCGGGCGCTATGGGAATAGCCTGCGTTCGATTAGCTAGTTGGTGAGGTAATGGCTCACCAAGGCTACGATCGA<br>TAGCTGGTTTGAGAGGATGATCAGCATGACTGGAACTGAGATACGGTCCAGACTCCTACGGGAGGCAGCAGTAGGGAATTT<br>TCCACAATGGACGAAAGTCTGATGGAGCAACGCCGCGTGCAGGATGAAGGCCTTCGGGTCGTAAACTGCTTTTATGTGCG<br>AAGAATATGACGGTAACACATGAATAAGGATCGGCTAACTACGTGCCAGCAGCCGCGGTCATACGTAGGATCCAAGCGTTA<br>TCCGGAATTACTGGGCGTAAAGAGTTGCGTAGGTGGCATAACAAGTGGAGCGTGAAAGCGTGTGGCTCAACCATACAGCTG<br>CGTTCCAAACTGTTAAGCTTGAGTATGAGAGGGGTAGATGGAATTTCTAGTGTAGGAGTGAAATCCGTAGATATTAGAAGGAA<br>CACCGATAGCGTAGGCAGTCTACTGGCTCATTACTGACACTAAGGCACGAAAGCGTGGGTAGCAAACGGGATTAGATACCC<br>CGGTAGTCCACGCCGTAAACTATGGATGCTAGCTGTTACTCACATCGACCTGAGTAGTAGCGAAGCTAACGCGTTAAGCAT<br>CCCGCCTGGGGAGTACGGTCGCAAGATTAAAACTCAAAGGAATTGACGGGGACCCGCACAAGCGGTGGAGCGTGTTGTTT<br>AATTCGATGGTAAGCGAAGAACCTTACCAAGGCTTGAAATCTCTGGAAGTCTAGCGAAAGCTGGATGTGCCTTCGGGAACC<br>AGATGACAGGTGCTGCATGGCCGTCGTCAGCTCGTGTCGTGAGATGTATGGTTAAGTCCATTAACGAGCGCAACCCTTGT<br>GTTTAGTTGAATTTTTCTAAACAGACTGCCCCGGCAACGGGGAGGAAGGAGGGGATGATGTCAGGTCAGTATTGCCCTTAC<br>GCCTTGGGCTACAAACACGCTACAATGGCCGGTACAAAGGGCTGCCAACCCGCGAGGGGGAGCAAATCCCATCAAAACC<br>GGTCTCAGTTCGGATTGCAGGCTGAAACTCGCCTGCATGAAGCTGGAATCGCTAGTAACGGTAGGTCAGCACACTACCGT<br>GAATACGTTCCCGGGTCTTGTACACACCGCCCGTCAAACCATGAAAGTCGCCAACACCCAAAGTCTGAGCTTTGCCTCGG<br>CCTAAGGTGGGGGAGATGATTGGGGTT
158	Lactobacillaceae gen. sp. OTU_789	GACGAACGCTGGCGGCGTGCCTAATACATGCAAGTCGAGCGAGCTTGCCTAGATGAATTTGGTGCTTGCACCAAATGAAAC<br>TAGATACAAGCGAGCGGCGGACGGGTGAGTAACACGTGGGTAACCTGCCCAAGAGACTGGGATAACACCTGGAAACAGAT<br>GCTAATACCGGATAACAACACTAGACGCATGTCTAGAGTTTAAAAGATGGTTCTGCTATCACTCTTGGATGGACCTGCGGTG<br>CATTAGCTAGTTGGTAAGGTAACGGCTTACCAAGGCAATGATGCATAGCCGAGTTGAGAGACTGATCGGCCACATTGGGAC<br>TGAGACACGGCCCAAACTCCTACGGGAGGCAGCAGTAGGGAATCTTCCACAATGGACGCAAGTCTGATGGAGCAACGCCG<br>CGTGAGTGAAGAAGGGTTTCGGCTCGTAAAGCTCTGTTGGTAGTGAAGAAAGATAGAGGTAGTAACTGGCCTTTATTTGACG<br>GTAATTACTTAGAAAGTCACGGCTAACTACGTGCCAGCAGCCGCGGTAATACGTAGGTGGCAAGCGTTGTCCGGATTTATT<br>GGGCGTAAAGCGAGTGCAGGCGGTTCAATAAGTCTGATGTGAAAGCCTTCGGCTCAACCGGAGAATTGCATCAGAAACTGT<br>TGAACTTGAGTGCAGAAGAGGAGAGTGGAACTCCATGTGTAGCGGTGGAATGCGTAGATATATGGAAGAACACCAGTGGCG<br>AAGGCGGCTCTCTGGTCTGCAACTGACGCTGAGGCTCGAAAGCATGGGTAGCGAACAGGATTAGATACCCTGGTAGTCCA<br>TGCCGTAAACGATGAGTGCTAAGTGTTGGGAGGTTTCCGCCTCTCAGTGCTGCAGCTAACGCATTAAGCACTCCGCCTGG<br>GGAGTACGACCGCAAGGTTGAAACTCAAAGGAATTGACGGGGGCCCGCACAAGCGGTGGAGCATGCGGATTAATTCGATG<br>CAACGCGAAGAACCTTACCAGGGCTTGACATAACCTGGATGATGCCAGAGATGGTGTGTCCCTTCGGGGCCAGGTTACAG<br>GTGGTGCATGGTCGTCGTCAGCTCGTGTCGTGAGATGTTGGGTTAAGTCCCGCAACGAGCGCAACCCTTGCCTTGTGTTA<br>CCAGCGGGTCGTGCCGGGGACTCACAAGGGACCGCCGGGGTTAACTCGGAGGAAGGTGGGGATGACGTCAGATCATCAT<br>GCCCCTTACGTCCTGGGCTTCACGCATGCTACAATGGCTGGTACAGCGGGATGCGATACTGTAAGGTGGAGCGGATCCT<br>GTAAAACCGGTCTCAGTTCGGATCGGGGCCTGCAACTCGGCCTCGTGAAGGTGGAGTCGCTAGTAATCGCGGATCAGCA<br>GTGCCGCGGTGAATGCGTTCCCGGGCCTTGTACACACCGCCCGTCAAGTCATGAAAGTGGGCAGCACCCGAAGCCGGT<br>GGCCTAACCTATGTGGGGGGAGCCGTCTAAGGTGAGGTTCGCGATTGGGACT
159	Clostridiales XIII gen. sp. OTU_798	GATGAACGCTGGCGGCGTGCCTAACACATGCAAGTCGAGCGGTATATACTTAAATGAAACTTCGGTCGAGTGAGAGTATAG<br>AGAGCGGCGGACGGGTGAGTAACGCGTAGGCAACCTGCCCCATACAGAGGGATAGCCTCGGGAAACCGGGATTAAAACC<br>TCATAACGCGAAAGAGTCACATGGCTTTTTCGCCAAAGATTTATCGGTATGGGATGGGCCTGCGTCTGATTAGCTAGTTGGT<br>GGGGTAACGGCCTACCAAGGCAACGATCAGTAGCCGACCTGAGAGGGTAATCGGCCACATTGGAACTGAGACACGGTCCA<br>AACTCCTACGGGAGGCAGCAGTGGGGAATATTGCACAATGGGCGAAAGCCTGATGCAGCAACGCCGCGTGAGCGATGAAG<br>GCCTTTGGGTCGTAAAGCTCTGTCCTTGGGGAAGAATAAATGACGGTACCCTTGGAGGAAGCCCCGGCTAACTACGTGCC<br>AGCAGCCGCGGTAATACGTAGGGGTCAAGCGTTATCCGGAATTATTGGGCGTAAAGAGTGCGTAGGTGGTTTTGTAAGCGC<br>GGGGTGAAAGGCAATGGCTCAACCATTGTAAGCCTTGCGAACTGCAAGACTTGAGTGCAGGAGAGGAAAGTGGAATTCCTA<br>GTGTAGCGGTGAAATGCGTAGATATTAGGAGGAACACCAGTGGCGAAGGCGACTTTCTGGACTGTAACTGACACTGAGGCA<br>CGAAAGCGTGGGGAGCAAACAGGATTAGATACCCTGGTAGTCCACGCCGTAAACGATGAGCACTAGGTGTCGGGGCCGCA<br>AGGTTTCGGTGCCGCAGTTAACGCATTAAGTGCTCCGCCTGGGGAGTACGCACGCAAGTGTGAAACTCAAAGGAATTGAC<br>GGGGACCCGCACAAGCAGCGGAGCATGTGGTTTAATTCGAAGCAACGCGAAGAACCTTACCAGGACTTGACATCCCTCTG<br>ACAGTCCCTTAACCGGGATCTCTCTTCGGAGCAGAGGAGACAGGTGGTGCATGGTTGTCGTCAGCTCGTGTCGTGAGATG<br>TTGGGTTAAGTCCCGCAACGAGCGCAACCCTTGTCATTAGTTGCCAGCAGTTCGGCTGGGCACTCTAGTGAGACTGCCGG<br>GGATAACTCGGAGGAAGGTGGGGATGACGTCAAATCATCATGCCCCTTATGTTCTGGGCTACACACGTGCTACAATGGCTG<br>GTACAAAGAGAAGCGAGACGGTGACGTGGAGCAAATCTCAAAAACCAGTCCCAGTTCGGATTGTAGGCTGCAACTCGCCTA<br>CATGAAGTCGGAGTTGCTAGTAATCGCGAATCAGAATGTCGCGGTGAATGCGTTCCCGGGTCTTGTACACACCGCCCGTC<br>ACACCATGGAAGTTGGGGGCGCCCGAAGTCGGTCAGTTAATAGGCTGCCTAAGGCGAAATCAATGACTGGGGTG
160	Capnocytophaga sp. OTU_862	GATGAATGCTGGCGGCGTGCCTAACACATGCAAGTCGAGCGGCAGCGAGGGGTGCTTGCACCCCGTCGGCGAGCGGCG<br>AACGGCTGAGTAACGCGTAGGAATTTGCCCCAAAGTGAGGAATAACTGCCCGAAAGGGTAGCTAATGCCGCATATGGTCTT<br>CGGATTAAAGGATTTATCCGCTTTGGGAGAAGCCTGCGTTGGATTAGGTTGTTGGTAGGGTAATGGCCTACCAAGCCGACG<br>ATCCATAGCTGGTCTGAGAGGATGATCAGCCAGACTGGAACTGAGACACGGTCCAGACTCCTACGGGAGGCAGCAGTGAG<br>GAATATTGGACAATGGTCGGAAGACTGATCCAGCCATGCCGCGTGCAGGAAGACGGCCTTATGGGTTGTAAACTGCTTTTG<br>CAGGGGAAGAATAAGGACTACGTGTAGTTTGATGACGGTACTCTGCGAATAAGCATCGGCTAACTCCGTGCCAGCAGCCG<br>CGGTAATACGGAGGATGCGAGCGTTATCCGGAATCATTGGGTTTAAAGGGTCCGTAGGCGGGCTAATAAGTCAGAGGTGAA<br>AGCGCTCAGCTCAACTGAGCAACTGCCTTTGAAACTGTTAGTCTTGAATGGTTGTGAAGTAGTTGGAATGTGTAGTGTAGCG<br>GTGAAATGCTTAGATATTACACAGAACACCGATAGCGAAGGCATATTACTAACAATTAATTGACGCTGATGGACGAAAGCGTG<br>GGGAGCGAACAGGATTAGATACCCTGGTAGTCCACGCTGTAAACGATGGATACTAGCTGTTTGGTTGTTAAGACTGAGTGG<br>CTAAGCGAAAGTGATAAGTATCCCACCTGGGGAGTACGCACGCAAGTGTGAAACTCAAAGGAATTGACGGGGGCCCGCAC<br>AAGCGGTGGAGCATGTGGTTTAATTCGATGATACGCGAGGAACCTTACCAAGGTTTAAATGGAGACTGACGTACTTAGAGAT<br>AGGTATTTCTTCGGACAGTTTTCAAGGTGCTGCATGGTTGTCGTCAGCTCGTGCCGTGAGGTGTCAGGTTAAGTCCTATAA<br>CGAGCGCAACCCCTGCCATTAGTTGCTAGCGAGTCAAGTCGAGCACTCTAGTGGGACTGCCGGTGCAAACCGTGAGGAA<br>GGTGGGGATGACGTCAAATCATCACGGCCCTTACATCTTGGGCTACACACGTGCTACAATGGTCGTTACAGAGAGCAGCC<br>ACTGCGTGAGCAGGCGCGAATCTATAAAGACGATCACAGTTCGGATCGGAGTCTGCAACTCGACTCCGTGAAGCTGGAAT<br>CGCTAATAATCGGATATCAGCCATGATCCGGTGAATACGTTCCCGGGCCTTGTACACACCGCCCGTCAAGCCATGGAAGC<br>TGGGGGTACCTGAAGACGGTCACCGCGAGGAGCTGTTTAGGGTAAAACTAGTGACTGGGGCT

Sr no.	Species_OTUs	16S reference sequence
161	Capnocytophaga sp. OTU_873	CGCAGTGGCTGCTCTCTGTAACGACCATTGTAGCACGTGTGTAGCCCAAGATGTAAGGGCCGTGATGATTTGACGTCATCCCCACCTTCCTCACGGTTTACACCGGCAGTCTTGCTAGAGTCCCCACCATCACGTGCTGGTAACTAACAATAGGGGTTGCGCTCGTTATAGGACTTAACCTGACACCTCACGGCACGAGCTGACGACAACCATGCAGCACCTTGAAAACTGTCCGAAGAAATACCTATCTCTAAGTACGTCAGTTCCCATTTAAACCTTGGTAAGGTTCCTCGCGTATCATCGAATTAAACCACATGCTCCACCGCTTGTGCGGGCCCCCGTCAATTCCTTTGAGTTTCACACTTGCGTGCGTACTCCCCAGGTGGGATACTTATCACTTTCGCTTAGCCACTCAGCTTACGCCAAACAGCTAGTATCCATCGTTTACAGCGTGGACTACCAGGGTATCTAATCCTGTTCGCTCCCCACGCTCTCGTCTCTCAGCGTCAATCATTTGTTAGTCTCCTGCCTTCGCAATCGGTGTTCTGTGTAATATCTAAGCATTTCACCGCTACACTACACATTCCAAAAACTTCACAAATATTCAAGACTACCAGTATCAAAGGCAATTTCACAGTTAAGCTGTAAGATTTCACCCCTGACTTAATAGCCCGCCTACAGACCCTTTAAACCCAATGATTCCGAATAACGCTCGCATCCTCCGTATTACCGCGGCTGCTGGCACGGAGTTAGCCGATGCTTATTCATATAGTACCGTCATCAGAGTACACGTACTCCTTATTCTCCCTATATAAAAGCAGTTTACAACCCATAAGGCATTCTTCCTGCACGCGGCATGGCTGGTTCAGTCTTCCGACCATTGACCAATATTCCTCACTGCTGCCTCCCGTAGGAGTCTGGTCCCTGTCTCAGTACCAGTGTGGGGGATCTCCCTCTCAGGACCCCTACCTATCGTAGCCTTGGGGTGCCGTTACCACTCCAACTAGCTAATAGGACGCATGCCCATCTTCTACCGCCTCAGCTTTAATTATCAAACG
162	Lancefieldella sp. OTU_889	GATGAACGCTGGCGGCGCGCCTAACACATGCAAGTCGAACGGTTAAAGCACCTTTTAGGTGTGTATAAAGTGGCGAACGGCTGAGTAACACGTGGGCAACCTGCCCTCCTCTTGGGGATAGCCTCGGGAAACCGAGGATAATACCCGATACTTCAATGTGGCCGCATGACGACATTGAGAAAGCTTTTGCGGAGAAGGATGGGCCCGCGGCCTGTTAGCTTGTTGGTGGGGTAGAGGCCTACCAAGGCAATGATGGGTAGCTGGGTTGAGAGACCGACCAGCCAGATTGGGACTGAGACACGGCCCAGACTCCTACGGGAGGCAGCAGTGGGGAATCTTGCACAATGGGCGAAAGCCTGATGCAGCGACGCCGCGTGCGGGACGAAGGCCTTCGGGTTGTAAACCGCTTTCAGCAGGGACGAGGCGAAAGTGACGGTACCTGCAGAAGAAGCCCCGGCTAACTACGTGCCAGCAGCCGCGGTAATACGTAGGGGGCAAGCGTTATCCGGATTCATTGGGCGTAAAGCGCTCGTAGGCGGTCTGTTAGGTCGGGAGTTAAATCCGGAGGCTCAACCTCCGCCCGCTCCCGATACCGGCAGACTTGAGTTTGGTAGGGGAAGGTGGAATTCCTAGTGTAGCGGTGGAATGCGCAGATATTAGGAAGAACACCAGTGGCGAAGGCGGCCTTCTGGGCCATAACTGACGCTGAGGAGCGAAAGCTAGGGGAGCAAACAGGATTAGATACCCTGGTAGTCCACGCTGTAAACGATGATTACTAGGTGTTGGGGGTCGAACCTCAGCGCCCAAGCAAACGCGATAAGTAATCCGCCTGGGGAGTACGTACGCAAGTATGAAACTCAAAGGAATTGACGGGGACCCGCACAAGCGGTGGAGCATGTGGTTTAATTCGACGCAACGCGAGGAACCTTACCAGCGTTTGACATCTTAGGAATGAGATAGAGATATTTCAGTGTCCCTTCGGGGAAACCTAAAGACAGGTGGTGCATGGCTGTCGTCAGCTCGTGTCGTGAGATGTTGGGTTAAGTCCCGCAACGAGCGCAACCCCTTTCGTATGTTACCATCATTAAGTTGGGGACTCATGCGATACTGCCTACGATGAGTAGGAGGAAGGTGGGGATGACGTCAAGTCATCATGCCCCTTATACGCTGGGCTACACACGTGCTACAATGGGTAGAACAGAGAGTTGCAAAGCCGTGAGGTGAAGCTAATCTCAGAAAACTATTCTTAGTTCGGATTGTACTCTGCAACTCGAGTACATGAAGTTGGAATCGCTAGTAATCGCGAATCAGCAATGTCGCGGTGAATACGTTCTCGGGTCTTGTACACACCGCCCGTCACACCACGAGAGTTGGTTGCACCTGAAGTAGCAGGCCTAACCGTAAGGAGGGATGCTCCGAGGGTGTGATTAGCGATTGGGGTG
163	Prevotella sp. OTU_892	GATGAACGCTGGCTACAGGCTTAACACATGCAAGTCGTGGGGAAACGGCATTATGTGCTTGCACATTCTGGACGTCGACCGGCGCACGGGTGAGTATCGCGTATCCAACCTGCCCCTTACTTGGGGATACCCCGTTGAAAGACGGTCTAATACCCGATGTGTTTCATTGACGGCATCCGATATGAAACAAAGGTTTTCCGGTAAGGGATGGGGATGCGTCTGATTAGCTTGTTGGCGGGGCAACGGCCCACCAAGGCGACGATCAGTAGGGGTTCTGAGAGGAAGGTCCCCCACATTGGAACTGAGACACGGTCCAAACTCCTACGGGAGGCAGCAGTGAGGAATATTGGTCAATGGACGCAAGTCTGAACCAGCCAAGTAGCGTGCAGGATGACGGCCCTATGGGTTGTAAACTGCTTTTATGTGGGAATAAAGTGGCGCACGTGTGCGCCATTGCATGTACCTCATGAATAAGGACCGGCTAATTCCGTGCCAGCAGCCGCGGTAATACGGAAGGTCCGGGCGTTATCCGGATTTATTGGGTTTAAAGGGAGCGTAGGCCGTGGATTAAGCGTGTTGTGAAATGCAGGTGCTCAACGTCTGCACTGCAGCGCGAACTGGTCCACTTGAGTGTGCGCAACGCAGGCGGAATTCGTCGTGTAGCGGTGAAATGCTTAGATATGACGAAGAACTCCGATTGCGAAGGCAGCTTGCGGGAGCACAACTGACGCTGAAGCTCGAAAGTGCGGGTATCGAACAGGATTAGATACCCTGGTAGTCCGCACGGTAAACGATGGATGCCCGTTGTCAGGCTGTTTCAGCCTGGTGACCAAGCGAAAGCATTAAGCATCCCACCTGGGGAGTACGCCGGCAACGGTGAAACTCAAAGGAATTGACGGGGGCCCGCACAAGCGGAGGAACATGTGGTTTAATTCGATGATACGCGAGGAACCTTACCCGGGCTTGAATTGCAGACGAACGATTCAGAGATGATGAGGCCCTTCGGGGCGTCTGTGAAGGTGCTGCATGGTTGTCGTCAGCTCGTGCCGTGAGGTGTCGGCTTAAGTGCCATAACGAGCGCAACCCCTTTCTTCAGTTGCCATCAGGTGATGCTGGGCACTCTGTAGATACTGCCACCGCAAGGTGTGAGGAAGGTGGGGATGACGTCAAATCAGCACGGCCCTTACGTCCGGGGCTACACACGTGTTACAATGGCCGGTACAGAGAGTTGATTTTGTGCAAACACGATCTAATCCTTAAATCCGGTCCCAGTTCGGACTGGGGTCTGCAACCCGACCCCACGAAGCTGGATTCGCTAGTAATCGCGCATCAGCCATGGCGCGGTGAATACGTTCCCGGGCCTTGTACACACCGCCCGTCAAGCCATGAAAGCCGGGGGTGCCTGAAGTCTGTGACCGCAAGGAACGGCCTAGGGCAAAACCGGTGATTGGGGCT
164	Prevotella sp. OTU_894	GATGAACGCTGGCTACAGGCTTAACACATGCAAGTCGTGGGGAAACGGCATTATGTGCTTGCACATTCTGGACGTCGACCGGCGCACGGGTGAGTATCGCGTATCCAACCTGCCCCTTACTTGGGGATACCCCGTTGAAAGACGGTCTAATACCCGATGTGTTTCATTGACGGCATCCGATATGAAACAAAGGTTTTCCGGTAAGGGATGGGGATGCGTCTGATTAGCTTGTTGGCGGGGCAACGGCCCACCAAGGCGACGATCAGTAGGGGTTCTGAGAGGAAGGTCCCCCACATTGGAACTGAGACACGGTCCAAACTCCTACGGGAGGCAGCAGTGAGGAATATTGGTCAATGGACGCAAGTCTGAACCAGCCAAGTAGCGTGCAGGATGACGGCCCTATGGGTTGTAAACTGCTTTTATGTGGGAATAAAGTGGCGCACGTGTGCGCCATTGCATGTACCTCATGAATAAGGACCGGCTAATTCCGTGCCAGCAGCCGCGGTAATACGGAAGGTCCGGGCGTTATCCGGATTTATTGGGTTTAAAGGGAGTGTAGGCGGTCTGTTAAGCGTGTTGTGAAATTTAGGTGCTCAACATTTAACTTGCAGCGCGAACTGTCAGACTTGAGTACACGCAGCGCAGGCGGAATTCATGGTGTAGCGGTGAAATGCTTAGATATCATGAGGAACTCCGATCGCGAAGGCAGCCTGCGGGAGTGTTACTGACGCTTAAGCTCGAAGGTGCGGGTATCGAACAGGATTAGATACCCTGGTAGTCCGCACAGTAAACGATGGATGCCCGCCGTTGGCGCCCTGCGCCTGCGGCCAAGCGAAAGCATTAAGCATCCCACCTGGGGAGTACGCCGGCAACGGTGAAACTCAAAGGAATTGACGGGGGCCCGCACAAGCGGAGGAACATGTGGTTTAATTCGATGATACGCGAGGAACCTTACCCGGGCTTGAATTGCAGACGCACGATTCAGAGATGATGAGGCCCTTCGGGGCGTCTGTGAAGGTGCTGCATGGTTGTCGTCAGCTCGTGCCGTGAGGTGTCGGCTTAAGTGCCATAACGAGCGCAACCCCTTTTTTCAGTTGCCATCAGGTGATGCTGGGCACTCTGGAGATACTGCCACCGTAAGGTGTGAGGAAGGTGGGGATGACGTCAAATCAGCACGGCCCTTACGTCCGGGGCTACACACGTGTTACAATGGCCGGTACAGAGCGTTGGTTGTGTGCAAATACGATCTAATCCTTAAAGCCGGTCCCAGTTCGGACTGGGGTCTGCAACCCGACCCCACGAAGCTGGATTCGCTAGTAATCGCGCATCAGCCATGGCGCGGTGAATACGTTCCCGGGCCTTGTACACACCGCCCGTCAAGCCATGAAAGCCGGGGGTGCCTGAAGTCTGTGACCGCAAGGAACGGCCTAGGGCAAAACTGGTGATTGGGGCT

Sr no.	Species_OTUs	16S reference sequence
165	Fusobacterium sp. OTU_895	GATGAACGCTGACAGAATGCTTAACACATGCAAGTCAACTTGAATTTGGGTTTTTAACTTAGGTTTGGGTGGCGGACGGGTG<br>AGTAACGCGTAAAGAACTTGCCTCACAGCTAGGGACAACATTTGGAAACGAATGCTAATACCTAATATTATGATAATAGGGCA<br>TCCTATAATTATGAAAGCTATAAGCGCTGTGAGAGAGCTTTGCGTCCCATTAGCTAGTTGGAGAGGTAACGGCTCACCAAGG<br>CGATGATGGGTAGCCGGCCTGAGAGGGTGATCGGCCACAAGGGGACTGAGACACGGCCCTTACTCCTACGGGAGGCAG<br>CAGTGGGGAATATTGGACAATGGACCAAGAGTCTGATCCAGCAATTCTGTGTGCACGATGAAGTTTTTCGGAATGTAAAGTG<br>CTTTCAGTTGGGAAGAAAGAAATGACGGTACCAACAGAAGAAGTGACGGCTAAATACGTGCCAGCAGCCGCGGTAATACGT<br>ATGTCACGAGCGTTATCCGGATTTATTGGGCGTAAAGCGCGTCTAGGTGGTTATGTAAGTCTGATGTGAAAATGCAGGGCT<br>CAACTCTGTATTGCGTTGGAAACTGTATAACTAGAGTACTGGAGAGGTAAGCGGAACTACAAGTGTAGAGGTGAAATTCGTA<br>GATATTTGTAGGAATGCCGATGGGGAAGCCAGCTTACTGGACAGATACTGACGCTGAAGCGCGAAAGCGTGGGTAGCAAAC<br>AGGATTAGATACCCTGGTAGTCCACGCCGTAAACGATGATTACTAGGTGTTGGGGGTCGAACCTCAGCGCCCAAGCAAAC<br>GCGATAAGTAATCCGCCTGGGGAGTACGTACGCAAGTATGAAACTCAAAGGAATTGACGGGGACCCGCACAAGCGGTGGA<br>GCATGTGGTTTAATTCGACGCAACGCGAGGAACCTTACCAGCGTTTGACATCTTAGGAGTGAGATAGAGATATTTCAGTGTC<br>CCTTCGGGGAAACCTAAAGACAGGTGGTGCATGGCTGTCGTCAGCTCGTGTCGTGAGATGTTGGGTTAAGTCCCGCAACG<br>AGCGCAACCCCTATTGTTAGTTGCCATCATTCAGTTGGGCACTCTAGCGAGACTGCCGGTAATAAACCGGAGGAAGGTGG<br>GGATGACGTCAAATCATCATGCCCCTTATGACCTGGGCTACACACGTGCTACAATGGCTGGTACAACGAGTCGCAAGCCG<br>GTGACGGCAAGCTAATCTCTTAAAGCCAGTCTCAGTTCGGATTGTAGGCTGCAACTCGCCTACATGAAGTCGGAATCGCTA<br>GTAATCGCGGATCAGCACGCCGCGGTGAATACGTTCCCGGGCCTTGTACACACCGCCCGTCACACCACGAGAGTTTGTA<br>ACACCCGAAGTCGGTGAGGTAACCTTTTAGGAGCCAGCCGCCTAAGGTGGGATAGATGATTGGGGTG
166	Eubacterium sp. OTU_896	GATGAACGCTGGCGGCGTGCTTAACACATGCAAGTCGAGCGAGAAGTTTTGAAAAGATTCTTCGGATGAAATTTAAAATGGA<br>AAGCGGCGGACGGGTGAGTAACGCGTAGGCAACCTGCCCCATACAAAGGGATAGCATTTGGAAACGAATATTAATACCTTA<br>TGAAACTTAACTAGTGCATGCTAGGTAGGTCAAAGATTTATCGGTATGGGATGGGCCTGCGTCTGATTAGCTAGTTGGTGAG<br>GTAACGGCTCACCAAGGCGACGATCAGTAGCCGACCTGAGAGGGTGAACGGCCACATTGGAACTGAGACACGGTCCAAAC<br>TCCTACGGGAGGCAGCAGTGGGGAATATTGCACAATGGGGGAAACCCTGATGCAGCAACGCCGCGTGAACGATGAAGGC<br>CTTTGGGTCGTAAAGTTCTGTTCTAGGTGATGAAAACTGACAGTAACCTAGGAGAAAGCCCCGGCTAACTCCGTGCCAGCA<br>GCCGCGGTAATACGGAGGGGGCAAGCGTTATCCGGAATTATTGGGCGTAAAGGGTACGTAGGTGGCCTTTTAAGCGTAGG<br>GTATAAGGCAATGGCTCAACCATTGTTCGCCCTATGAACTGGAAGGCTTGAGTGCAGGAGAGGAAAGCGGAATTCCTAGTG<br>TAGCGGTGGAATGCATAGATATTAGGAGGAACATCAGCGGCGAAGGCGGCTTTCTGGACTGCAACTGACACTGAGGTACGA<br>AAGCGTGGGGAGCAAACAGGATTAGATACCCTGGTAGTCCACGCCGTAAACGATGAGCACTAGGTGTCGGGGTCGTAAGG<br>CTTCGGTGCCGTAGTTAACGCATTAAGTGCTCCGCCTGGGGAGTACGCACGCAAGTGTGAAACTCAAAGGAATTGACGGG<br>GACCCGCACAAGCAGCGGAGCATGTGGTTTAATTCGAAGCAACGCGAAGAACCTTACCAGGACTTGACATCCTTCTGACC<br>GGTCTTTAATAGGACCTTTCTTTTGACAGAAGAGACAGGTGGTGCATGGTTGTCGTCAGCTCGTGTCGTGAGATGTTGGGT<br>TAAGTCCCGCAACGAGCGCAACCCTTGTCATTAGTTGCCATCATTAAGTTGGGCACTCTAGTGAGACTGCCGGGGACAACT<br>CGGAGGAAGGTGGGGATGACGTCAAATCATCATGCCCCTTATGTTCTGGGCTACACACGTGCTACAATGGCTGGTACAAAG<br>AGAAGCAAGACCGCAAGGTGGAGCAAAGCTCAAAAACCAGCCCCAGTTCGGATTGTAGGCTGAAACTCGCCTACATGAAGT<br>CGGAGTTGCTAGTAATCGCAGATCAGAATGCTGCGGTGAATGCGTTCCCGGGTCTTGTACACACCGCCCGTCACACCACG<br>AGAGTTGGTTGCACCTGAAGTAGCAGGCCTAACCGTAAGGAGGGATGCTCCGAGGGTGTGATTAGCGATTGGGGTG
167	Lactobacillales gen. sp. OTU_897	GACGAACGCTGGCGGCGTGCCTAATACATGCAAGTAGAACGCTGAAGAGAGGAGCTTGCTCTTCTTGGATGAGTTGCGAA<br>CGGGTGAGTAACGCGTAGGTAACCTGCCTGGTAGCGGGGGATAACTATTGGAAACGATAGCTAATACCGCATAAAATTGATT<br>ATTGCATGATAATTAATTGAAAGATGCAACTGCATCACTACCAGATGGACCTGCGTTGTATTAGCTAGTTGGTGAGGTAACGG<br>CTCACCAAGGCGACGATACATAGCCGACCTGAGAGGGTGATCGGCCACACTGGGACTGAGACACGGCCCAGACTCCTAC<br>GGGAGGCAGCAGTAGGGAATCTTCGGCAATGGGGGGAACCCTGACCGAGCAACGCCGCGTGAGTGAAGAAGGTTTTCGG<br>ATCGTAAAGCTCTGTTGTAAGAGAAGAACGGGTGTGAGAGTGGAAAGTTCACACTGTGACGGTATCTTACCAGAAAGGGACG<br>GCTAACTACGTGCCAGCAGCCGCGGTAATACGTAGGTCCCGAGCGTTGTCCGGATTTATTGGGCGTAAAGCGAGCGCAG<br>GCGGTTAGATAAGTCTGAAGTTAAAGGCTGTGGCTTAACCATAGTATGCTTTGGAAACTGTTTAACTTGAGTGCAGAAGGGG<br>AGAGTGGAATTCCATGTGTAGCGGTGAAATGCGTAGATATATGGAGGAACACCGGTGGCGAAAGCGGCTCTCTGGTCTGTA<br>ACTGACGCTGAGGCTCGAAAGCGTGGGGAGCAAACAGGATTAGATACCCTGGTAGTCCACGCCGTAAACGATGAGTGCTA<br>AGTGTTGGAGGGGTTCCACCCTTCAGTGCTGGAGTTAACGCAATAAGCACTCCGCCTGGGGAGTACGGCCGCAAGGCTG<br>AAACTCAAAGGAATTGACGGGGACCCGCACAAGCGGTGGAGCATGTGGTTTAATTCGAAGCAACGCGAAGAACCTTACCAG<br>GTCTTGACATCCCGACGACCGCTCTAGAGATAGAGTTTTTCTTCGGAACGTCGGTGACAGGTGGTGCATGGTTGTCGTCA<br>GCTCGTGTCGTGAGATGTTGGGTTAAGTCCCGCAACGAGCGCAACCCCTATAACTAGTTGCCAGCATTAAGATGGGGACT<br>CTAGTTAGACTGCCGGTGACAAACCGGAGGAAGGTGGGGATGACGTCAAATCATCATGCCCCTTATGACCTGGGCTACAC<br>ACGTGCTACAATGGATGGTACAACGAGCAGCGAACTCGCGAGGGTAAGCGAATCTCTAAAAGCCATTCTCAGTTCGGATTG<br>TAGTCTGCAACTCGACTACATGAAGCCGGAATCGCTAGTAATCGCGGATCAGCACGCCGCGGTGAATACGTTCCCGGGTC<br>TTGTACACACCGCCCGTCACACCACGAGAGTTTGTAACACCCGAAGCCGGTGGCCTAACCTTATAGGAGGGAGCCGTCGA<br>AGGTGGGATAGATGATTGGGGTG
168	Leptotrichia sp. OTU_898	GATGAACGCTGACAGAATGCTTAACACATGCAAGTCGATGGGGAAGTGGTGCTTGCACCATGGCAACCATGGCGGACGGG<br>TGAGTAACGCGTAAGGGACTTACCTGCAGGACTGGGATAACAGGGGGAAACTCCTGACAAGACCGGATAATGTCATGGAGC<br>TGCATGGCTTTATGATGAAAGGAGACGCCTGCGGAGAGCCTTGCGTCCTATTAGCTAGTTGGTGAGGTAATGGCTCACCAA<br>GGCGAAGATAGGTAGCCGGCCTGAGAGGGTGGACGGCCACAAGGGGACTGAGATACGGCCCTTACTCCTACGGGAGGCA<br>GCAGTGGGGAATATTGGACAATGGGGGCAACCCTGATCCAGCAATTCTGTGTGCACGAAGAAGGTTTTCGGATTGTAAAGT<br>GCTTTCAGCAGGGAAGAAGGAAGTGACGGTACCTGCAGAAGAAGCGACGGCTAAATACGTGCCAGCAGCCGCGGTAATAC<br>GTATGTCGCGAGCGTTATCCGGAATTATTGGGCATAAAGGGCATCTAGGCGGCCTGACAAGTCAGGGGTGAAAACCTGCG<br>GCTCAACCGCAGGCCTGCCTTTGAAACTGTGAGGCTGGAGTACCGGAGAGGTGGACGGAACTGCACGAGTAGAGGTGAAA<br>TTCGTAGATATGTGCAGGAATGCCGATGATGAAGATAGTTCACTGGACGGTAACTGACGCTGAAGTGCGAAAGCCGGGGGA<br>GCAAACAGGATTAGATACCCTGGTAGTCCCGGCTGTAAACGATGATTACTGGGTGTGGGCAGGAAGACTGTCTGTGCCGAA<br>GCGAATGCGATAAGTAATCCGCCTGGGGAGTACGGCCGCAAGGCTGAAACTCAAAGGAATTGACGGGGACCCGCACAAGC<br>GGTGGAGCATGTGGTTTAATTCGAAGCAACGCGAAGAACCTTACCAGGTCTTGACATCCCTCTGACCGCTCTAGAGATAGA<br>GTTTTCCTTCGGGACAGAGGTGACAGGTGGTGCATGGTTGTCGTCAGCTCGTGTCGTGAGATGTTGGGTTAAGTCCCGCA<br>ACGAGCGCAACCCCTATTGTTAGTTGCCATCATTCAGTTGGGCACTCTAGCGAGACTGCCGGTAATAAACCGGAGGAAGGT<br>GGGGATGACGTCAAATCATCATGCCCCTTATGACCTGGGCTACACACGTGCTACAATGGCTGGTACAACGAGTCGCAAGC<br>CGGTGACGGCAAGCTAATCTCTTAAAGCCAGTCTCAGTTCGGATTGTAGGCTGCAACTCGCCTACATGAAGTCGGAATCGC<br>TAGTAATCGCGGATCAGCACGCCGCGGTGAATACGTTCCCGGGCCTTGTACACACCGCCCGTCACACCACGAGAGTTTG<br>TAACACCCGAAGTCGGTGAGGTAACCTTTTAGGAGCCAGCCGCCTAAGGTGGGATAGATGATTGGGGTG

Sr no.	Species_OTUs	16S reference sequence
169	Campylobacter sp. OTU_901	AGTGAACGCTGGCGGCGTGCCTAATACATGCAAGTCGAACGGACAAGTAAGAGCTTGCTCTTATGAGTTAGTGGCGCACG<br>GGTGAGTAATGTATAGCTAATCTGCCCTACACTAGAGGACAACAGTTGGAAACGGCTGCTAATACTCTATACTCCTGTCTTA<br>CATAAGTTAGATAGGGAAAGTTTTTCGGTGTAGGATGAGGCTATATTGTATCAGCTAGTTGGTGAGGTAATGGCTCACCAAG<br>GCTATGACGCATAACTGGTCTGAGAGGATGATCAGTCACACTGGAACTGAGACACGGTCCAGACTCCTACGGGAGGCAGC<br>AGTAGGGAATATTGCTCAATGGGGGAAACCCTGAAGCAGCAACGCCGCGTGGAGGATGACACTTTTCGGAGCGTAAACTC<br>CTTTTGTTAGGGAAGAACAATGACGGTACCTAACGAATAAGCACCGGCTAACTCCGTGCCAGCAGCCGCGGTAATACGGAG<br>GGTGCAAGCGTTACTCGGAATCACTGGGCGTAAAGGACGCGTAGGCGGATTATCAAGTCTCTTGTGAAATCCTATGGCTTA<br>ACCATAGAACTGCTTGGGAAACTGGTAATCTAGAGTGAGGGAGAGGCAGATGGAATTGGTGGTGTAGGGGTAAAATCCGTA<br>GAGATCACCAGGAATACCCATTGCGAAGGCGATCTGCTGGAACTCAACTGACGCTAATGCGTGAAAGCGTGGGGAGCAAA<br>CAGGATTAGATACCCTGGTAGTCCACGCCCTAAACGATGTATACTAGTTGTTGCTAAGCTAGTCTTGGCAGTAATGCACCTA<br>ACGGATTAAGTATACCGCCTGGGGAGTACGGTCGCAAGATTAAAACTCAAAGGAATAGACGGGGACCCGCACAAGCGGTG<br>GAGCATGTGGTTTAATTCGAAGATACGCGAAGAACCTTACCCGGACTTGATATCTAACAAATCATCTAGAGATAGAAGAGTGT<br>CTGCTTGCAGAAATGTTAAGACAGGTGCTGCATGGCTGTCGTCAGCTCGTGTCGTGAGATGTTGGGTTAAGTCCCGCAAC<br>GAGCGCAACCCTTGTCATTAGTTGCCATCATTAAGTTGGGCACTCTAATGAGACTGCCGGTGACAAGCCGGAGGAAGGTG<br>GGGATGACGTCAAGTCCTCATGGCCCTTATGACCAGGGCTTCACACGTCATACAATGGTCGGTACAGAGGGTAGCCAAGC<br>CGCGAGGTGGAGCCAATCTCACAAAACCGATCGTAGTCCGGATTGCACTCTGCAACTCGAGTGCATGAAGTCGGAATCGC<br>TAGTAATCGCAGGTCAGCATACTGCGGTGAATACGTTCCCGGGTCTTGTACACACCGCCCGTCACACCATGGGAGTGGGG<br>GATACCAGAAGTAGGTAGGGTAACCGCAAGGAGCCCGCTTACCACGGTATGCTTCATGACTGGGGTG
170	Prevotella sp. OTU_903	GATGAACGCTGGCTACAGGCTTAACACATGCAAGTCGAGGGGAAACGACGGGGAAGCTTGCTTCCCCGGGCGTCGACCG<br>GCGCACGGGTGAGTAACGCGTATCCAACCTGCCTCTGACTGAGGGATAACCCGTCGAAAGTCGGCCTAATACCTCATGGT<br>ATCGTCTGCGGGCATCCAACGACGATTAAAGATTTCATCGGTCAGAGATGGGGATGCGTCTGATTAGCTGGTTGGCGGGG<br>TAACGGCCCACCAAGGCGACGATCAGTAGGGGTTCTGAGAGGAAGGTCCCCCACATTGGAACTGAGACACGGTCCAAACT<br>CCTACGGGAGGCAGCAGTGAGGAATATTGGTCAATGGGCGAGAGCCTGAACCAGCCAAGTAGCGTGCAGGATGACGGCC<br>CTATGGGTTGTAAACTGCTTTTATGCGGGGATAAAGTGAGGGACGTGTCCTTCATTGCAGGTACCGCATGAATAAGGACCG<br>GCTAATTCCGTGCCAGCAGCCGCGGTAATACGGAAGGTCCGGGCGTTATCCGGATTTATTGGGTTTAAAGGGAGTGTAGG<br>CGGTCTGTTAAGCGTGTTGTGAAATTTAGGTGCTCAACATTTAACTTGCAGCGCGAACTGTCAGACTTGAGTACACGCAGC<br>GCAGGCGGAATTCATGGTGTAGCGGTGAAATGCTTAGATATCATGAGGAACTCCGATCGCGAAGGCAGCCTGCGGGAGTG<br>TTACTGACGCTTAAGCTCGAAGGTGCGGGTATCGAACAGGATTAGATACCCTGGTAGTCCGCACAGTAAACGATGGATGCC<br>CGCCGTTGGCGCCCTGCGCCTGCGGCCAAGCGAAAGCATTAAGCATCCCACCTGGGGAGTACGCCGGCAACGGTGAAA<br>CTCAAAGGAATTGACGGGGGCCCGCACAAGCGGAGGAACATGTGGTTTAATTCGATGATACGCGAGGAACCTTACCCGGG<br>CTTGAATTGCAGTCGCTGCCCACAGAGACGTGGGCTTCCCTTCGGGGCGTCTGTGAAGGTGCTGCATGGTTGTCGTCAG<br>CTCGTGCCGTGAGGTGTCGGCTTAAGTGCCATAACGAGCGCAACCCCCTTTCCCAGTTGCCATCGGGTTATGCCGGGCA<br>CTCTGGGGACACTGCCACCGCAAGGTGCGAGGAAGGTGGGGATGACGTCAAATCAGCACGGCCCTTACGTCCGGGGCTA<br>CACACGTGTTACAATGGCCGGTACAGAGGGACGGTGCAATGCAAGTTGCATCCAATCTTGAAATCCGGTCCCAGTTCGGA<br>CTGGGGTCTGCAACCCGACCCCACGAAGCTGGATTCGCTAGTAATCGCGCATCAGCCATGGCGCGGTGAATACGTTCCC<br>GGGCCTTGTACACACCGCCCGTCAAGCCATGAAAGCCGGGGGTGCCTGAAGTTCGTGACCGCGAGGAGCGACCTAGGG<br>CAAGACCGGTGATTGGGGCT
171	Lactobacillales gen. sp. OTU_906	GACGAACGCTGGCGGCGTGCCTAATACATGCAAGTCGAACGAGAGCGACCGGTGCTTGCACTGGTCAATCTAGTGGCGAA<br>CGGGTGAGTAACACGTGGGTAACCTGCCCATCAGAGGGGGATAACATTCGGAAACGGATGCTAAAACCGCATAGGTCTTC<br>GAACCGCATGGTTTGAAGAGGAAAAGAGGCGCAAGCTTCTGCTGATGGATGGACCCGCGGTGCATTAGCTAGTTGGTGAG<br>GTAACGGCTCACCAAGGCCGTGATGCATAGCCGACCTGAGAGGGTGATCGGCCACATTGGGACTGAGACACGGCCCAAA<br>CTCCTACGGGAGGCAGCAGTAGGGAATCTTCCGCAATGGACGCAAGTCTGACGGAGCAACGCCGCGTGAGTGAAGAAGG<br>TTTTCGGATCGTAAAACTCTGTTGTTAGAGAAGAACAAGTGCTAGAGTAACTGTTAGCGCCTTGACGGTATCTAACCAGAAAG<br>CCACGGCTAACTACGTGCCAGCAGCCGCGGTAATACGTAGGTGGCGAGCGTTGTCCGGATTTATTGGGCGTAAAGGGAGT<br>GTAGGCGGTCTTTTAAGTCTGATGTGAAAGCCCACGGCTCAACCGTGGAGGGTCATTGGAAACTGGGAGACTTGAGTGCA<br>GAAGAGGAGAGCGGAATTCCATGTGTAGCGGTGAAATGCGTAGATATATGGAGGAACACCAGTGGCGAAGGCGGCTCTCT<br>GGTCTGTAACTGACGCTGAGGCTCGAAAGCGTGGGGAGCAAACAGGATTAGATACCCTGGTAGTCCACGCCGTAAACGAT<br>GAGTGCTAAGTGTTGGAGGGGTTCCACCCTTCAGTGCTGGAGTTAACGCAATAAGCACTCCGCCTGGGGAGTACGGCCG<br>CAAGGCTGAAACTCAAAGGAATTGACGGGGACCCGCACAAGCGGTGGAGCATGTGGTTTAATTCGAAGCAACGCGAAGAAC<br>CTTACCAGGTCTTGACATCCCGACGACCGCTCTAGAGATAGAGTTTTTCTTCGGAACGTCGGTGACAGGTGGTGCATGGTT<br>GTCGTCAGCTCGTGTCGTGAGATGTTGGGTTAAGTCCCGCAACGAGCGCAACCCCTATAACTAGTTGCCAGCATTAAGATG<br>GGGACTCTAGTTAGACTGCCGGTGACAAACCGGAGGAAGGTGGGGATGACGTCAAATCATCATGCCCCTTATGACCTGGG<br>CTACACACGTGCTACAATGGATGGTACAACGAGCAGCGAACTCGCGAGGGTAAGCGAATCTCTAAAAGCCATTCTCAGTTC<br>GGATTGTAGTCTGCAACTCGACTACATGAAGCCGGAATCGCTAGTAATCGCGGATCAGCACGCCGCGGTGAATACGTTCC<br>CGGGTCTTGTACACACCGCCCGTCACACCACGAGAGTTTGTAACACCCGAAGCCGGTGGCCTAACCTTTTAGGAGGGAG<br>CCGTCGAAGGTGGGATAGATGATTGGGGTG
172	Eggerthellaceae gen. sp. OTU_907	GATGAACGCTGGCGGCGTGCCTAACACATGCAAGTCGAACGGTTAAACCGCCTTCGGGCGGAGATACAGTGGCGAACGG<br>GTGAGTAACACGTGACCAACCTGCCCCCCGCACCGGGACAACCTCGGGAAACCGAGGCTGATACCGGATATTCCCTCCT<br>CCCCTCCTGGGGAGGCGGGGAAAGCTCCGGCGGCGGGGGATGGGGTCGCGGCCCATCAGGTAGTAGGCGGGGTAACG<br>GCCCACCTAGCTTTCGACGGGTAGCCGGGCTGAGAGGCCGGACGGCCACATTGGGACTGAGATACGGCCCAGACTCCT<br>ACGGGAGGCAGCAGTGGGGAATCTTGCGCAATGGGGGAAACCCTGACGCAGCAACGCCGCGTGCGGGATGACGGCCTT<br>CGGGTTGTAAACCGCTTTCAGCAGGGAAGAAGTTGACGGTACCTGCAGAAGAAGCCCCGGCTAACTACGTGCCAGCAGCC<br>GCGGTAACACGTAGGGGGCGAGCGTTATCCGGATTCATTGGGCGTAAAGCGCGCGTAGGCGGCCCGCCAAGCAGAACCT<br>CTAACCTCGGGGCCCAACCCCGAGCCGGGTTCTGGACTGGCGGGCTCGAGTCTGGTAGAGGAAGGCGGAATTCCCGGT<br>GTAGCGGTGAAATGCGCAGATATCGGGAAGAACACCGATGGCGAAGGCAGCCTTCTGGGCCACGACTGACGCTGAGGCG<br>CGAAAGCTGGGGGAGCGAACAGGATTAGATACCCTGGTATTCCCAGCCGTAAACGATGGACGCTAGGTGTGGGGGGATGA<br>CCCTCCGTGCCGAAGCCAACGCATTAAGCGTCCCGCCTGGGGAGTACGGCCGCAAGGCTAAAACTCAAAGGAATTGACG<br>GGGGCCCGCACAAGCAGCGGAGCATGTGGCTTAATTCGAAGCAACGCGAAGAACCTTACCAGGGCTTGACATGCGCATGA<br>AGCCGGGGAGACCCGGTGGCCGAGAGGAGTGCGCGCAGGTGGTGCATGGCTGTCGTCAGCTCGTGTCGTGAGATGTTG<br>GGTTAAGTCCCGCAACGAGCGCAACCCCTGCCCCGTGTTGCCAGCATTCAGTTGGGGACTCGCGGGGGACCGCCGGCG<br>TCAAGCCGGAGGAAGGCGGGGACGACGTCAAGTCATCATGCCCCTTATGCCCTGGGCCGCACACGTGCTACAATGGCCG<br>GCACAGAGGGCTGCGAACCCGCGAGGGCGAGCGAATCCCACAAAGCCGGCCCCAGTTCGGATCGGAGGCTGCAACCCG<br>CCTCCGTGAAGCCGGAGTTGCTAGTAATCGCGGATCAGCATGCCGCGGTGAATACGTTCCCGGGCCTTGTACACACCGC<br>CCGTCACACCACCCGAGTCGTCTGCACCCGAAGCCGCCGGCCGAACCCGCAAGGGACGGAGGCGTCGAAGGTGTGGA<br>GGGTAAGGGGGGTG

Sr no.	Species_OTUs	16S reference sequence
173	Streptococcus sp. OTU_908	GACGAACGCTGGCGGCGTGCCTAATACATGCAAGTAGAACGCTGAAGGAGGAGCTTGCTTCTCTGGATGAGTTGCGAACG<br>GGTGAGTAACGCGTAGGTAACCTGCCTGGTAGCGGGGGATAACTATTGGAAACGATAGCTAATACCGCATAAGAGTAGATG<br>TTGCATGACATTTGCTTAAAAGGTGCAATTGCATCACTACCAGATGGACCTGCGTTGTATTAGCTGGTTGGTGAGGTAACGG<br>CTCACCAAGGCAACGATACATAGCCGACCTGAGAGGGTGATCGGCCACACTGGGACTGAGACACGGCCCAGACTCCTAC<br>GGGAGGCAGCAGTAGGGAATCTTCGGCAATGGACGGAAGTCTGACCGAGCAACGCCGCGTGAGTGAAGAAGGTTTTCGGA<br>TCGTAAAGCTCTGTTGTAAGAGAAGAACGAGTGTGAGAGTGGAAAGTTCACACTGTGACGGTATCTTACCAGAAAGGGACGG<br>CTAACTACGTGCCAGCAGCCGCGGTAATACGTAGGTCCCGAGCGTTGTCCGGATTTATTGGGCGTAAAGCGAGCGCAGG<br>CGGTTAGATAAGTCTGAAGTTAAAGGCTGTGGCTTAACCATAGTACGCTTTGGAAACTGTTTAACTTGAGTGCAAGAGGGGA<br>GAGTGGAATTCCATGTGTAGCGGTGAAATGCGTAGATATATGGAGGAACACCGGTGGCGAAAGCGGCTCTCTGGCTTGTAA<br>CTGACGCTGAGGCTCGAAAGCGTGGGGAGCAAACAGGATTAGATACCCTGGTAGTCCACGCCGTAAACGATGAGTGCTAG<br>GTGTTAGACCCTTTCCGGGGTTTAGTGCCGCAGCTAACGCATTAAGCACTCCGCCTGGGGAGTACGACCGCAAGGTTGAA<br>ACTCAAAGGAATTGACGGGGGCCCGCACAAGCGGTGGAGCATGTGGTTTAATTCGAAGCAACGCGAAGAACCTTACCAGG<br>TCTTGACATCCCTCTGACCGCTCTAGAGATAGAGTTTTCCTTCGGGACAGAGGTGACAGGTGGTGCATGGTTGTCGTCAG<br>CTCGTGTCGTGAGATGTTGGGTTAAGTCCCGCAACGAGCGCAACCCCTATTGTTAGTTGCCATCATTCAGTTGGGCACTCT<br>AGCGAGACTGCCGGTAATAAACCGGAGGAAGGTGGGGATGACGTCAAATCATCATGCCCCTTATGTCTTGGGCTTCACGC<br>ATGCTACAATGGCCGGTACAGAGGGTTGCGATACTGTGAGGTGGAGCTAATCCCTAAAAGCCGGTCTCAGTTCGGATTGG<br>GGTCTGCAACTCGACCCCATGAAGTCGGAGTCGCTAGTAATCGCAGATCAGCAACGCTGCGGTGAATACGTTCCCGGGC<br>CTTGTACACACCGCCCGTCAAGTCACGAAAGTTGGTAACACCCAAAGCCGGTGGCCTAACCTTTTTGGAGGGAGCCGTCT<br>AAGGTGGGATTGGCGATTGGGACT
174	Porphyromonas sp. OTU_909	GATGAACGCTAGCGATAGGCTTAACACATGCAAGTCGAGGGGCAGCATGATCTTAGCTTGCTAAGGTTGATGGCGACCGG<br>CGCACGGGTGCGTAACGCGTATGCAACTTGCCTTACAGAGGGGGATAACCCGTTGAAAGACGGACTAAAACCGCATACAC<br>TTGTATTATTGCATGATATTACAAGGAAATATTTATAGCTGTAAGATAGGCATGCGTCCCATTAGCTTGTTGGTGAGGTAACG<br>GCTCACCAAGGCGACGATGGGTAGGGGAACTGAGAGGTTTATCCCCCACACTGGTACTGAGACACGGACCAGACTCCTAC<br>GGGAGGCAGCAGTGAGGAATATTGGTCAATGGGCGAGAGCCTGAACCAGCCAAGTCGCGTGAAGGAAGACTGTCCTAAGG<br>ATTGTAAACTTCTTTTATACGGGAATAACGGGCGATACGAGTATTGCATTGAATGTACCGTAAGAATAAGCATCGGCTAACTC<br>CGTGCCAGCAGCCGCGGTAATACGGAGGATGCGAGCGTTATCCGGATTTATTGGGTTTAAAGGGTGCGTAGGTTGTTCGG<br>TAAGTCAGCGGTGAAACCTGAGCGCTCAACGTTCAGCCTGCCGTTGAAACTGCCGGGCTTGAGTTCAGCGGCGGCAGGC<br>GGAATTCGTGGTGTAGCGGTGAAATGCATAGATATCACGAGGAACTCCGATTGCGAAGGCAGCTTGCCATACTGCGACTGA<br>CACTGAAGCACGAAGGCGTGGGTATCAAACAGGATTAGATACCCTGGTAGTCCACGCAGTAAACGATGATTACTAGGAGTTT<br>GCGATATACCGTCAAGCTTCCACAGCGAAAGCGTTAAGTAATCCACCTGGGGAGTACGCCGGCAACGGTGAAACTCAAAG<br>GAATTGACGGGGGCCCGCACAAGCGGAGGAACATGTGGTTTAATTCGATGATACGCGAGGAACCTTACCCGGGCTTGAAT<br>TGCAGACGCAGGATACAGAGATGTTGACTCCCTTCGGGGCGTCTGTGAAGGTGCTGCATGGTTGTCGTCAGCTCGTGCC<br>GTGAGGTGTCGGCTTAAGTGCCATAACGAGCGCAACCCCTTTCCTTAGTTGCCATCAGGTGATGCTGGGCACTCTGGGGA<br>TACTGCCACCGCAAGGTGTGAGGAAGGTGGGGATGACGTCAAATCAGCACGGCCCTTACGTCCGGGGCTACACACGTGT<br>TACAATGGCCGGTACAGAGGGACGGTGCGATGCAAATCGCATCCAATCTTGAAAGCCGGTCCCAGTTCGGACTGAGGTCT<br>GCAACCCGACCTCACGAAGCTGGATTCGCTAGTAATCGCGCATCAGCCATGGCGCGGTGAATACGTTCCCGGGCCTTGT<br>ACACACCGCCCGTCAAGCCATGAAAGCCGGGGGTGCCTGAAGTTCGTAACCGCGAGGAGCGACCTAGGGCAAAACTGGT<br>GATTGGGGCT
175	Capnocytophaga sp. OTU_910	ATTGAACGCTGGCGGCATGCTTTACACATGCAAGTCGGACGGCAGCACAGAGAAGCTTGCTTCTTGGGTGGCGAGTGGCG<br>AACGGGTGAGTAACATATCGGAACGTACCGAGCAGTGGGGGATAACTAATCGAAAGATTAGCTAATACCGCATATATTCTGA<br>GGAAGAAAGCAGGGGACCATTTGGCCTTGCGCTGTTTGAGCGGCCGATATCTGATTAGCTAGTTGGTGGGGTAAAGGCCT<br>ACCAAGGCGACGATCAGTAGCGGGTCTGAGAGGATGATCCGCCACACTGGGACTGAGACACGGCCCAGACTCCTACGGG<br>AGGCAGCAGTGAGGAATATTGGACAATGGTCGGAAGACTGATCCAGCCATGCCGCGTGCAGGATGAAGGTCCTATGGATT<br>GTAAACTGCTTTTGTAAGGGAAGAATAAGAGCTACGTGTAGTTTGATGACGGTACCTTACGAATAAGCATCGGCTAACTCCG<br>TGCCAGCAGCCGCGGTAATACGGAGGATGCGAGCGTTATCCGGAATCATTGGGTTTAAAGGGTCCGTAGGCGGGCTGATA<br>AGTCAGAGGTGAAAGCGCTTAGCTCAACTAAGCAACTGCCTTTGAAACTGTCAGTCTTGAATGATTGTGAAGTAGTTGGAAT<br>GTGTAGTGTAGCGGTGAAATGCTTAGATATTACACAGAACACCGATAGCGAAGGCATATTACTAACAATTTATTGACGCTGAT<br>GGACGAAAGCGTGGGGAGCGAACAGGATTAGATACCCTGGTAGTCCACGCTGTAAACGATGGATACTAGCTGTTTGGAGCA<br>ATCTGAGTGGCTAAGCGAAAGTGATAAGTATCCCACCTGGGGAGTACGCACGCAAGTGTGAAACTCAAAGGAATTGACGGG<br>GGCCCGCACAAGCGGTGGAGCATGTGGTTTAATTCGATGATACGCGAGGAACCTTACCAAGGTTTAAATGGGAACTGACAG<br>AGGTAGAGATACCTTCTTCTTCGGACAGTTTTCAAGGTGCTGCATGGTTGTCGTCAGCTCGTGCCGTGAGGTGTCAGGTTA<br>AGTCCTATAACGAGCGCAACCCCTGCCATTAGTTGCTAACGAGTAAGGTCGAGCCCTCTAGTGGGACTGCCGGTGCAAAC<br>CGTGAGGAAGGTGGGGATGACGTCAAATCATCACGGCCCTTACATCTTGGGCTACACACGTGCTACAATGGCCGTTACAG<br>AGAGCAGCCACTGCGCGAGCAGGAGCGAATCTATAAAGACGGTCACAGTTCGGATCGGAGTCTGCAACTCGACTCCGTGA<br>AGCTGGAATCGCTAGTAATCGGATATCAGCCATGATCCGGTGAATACGTTCCCGGGCCTTGTACACACCGCCCGTCAAGC<br>CATGGAAGCTGGGGGTACCTGAAGACGGTCACCGCGAGGAGCTGTTTAGGGTAAAACTAGTGACTGGGGCT
176	Lancefieldella sp. OTU_936	GATGAACGCTGGCGGCGCGCCTAACACATGCAAGTCGAACGGTTAAAGCCCCTTCGGGGGTGCATAGAGTGGCGAACGG<br>CTGAGTAACACGTGGGCAACCTGCCCCCCGCACCGGGACAGCCTCGGGAAACCGTGGTTAATACCGGATACTCCGCCAC<br>CCCCGCATGGGGGCGGCGGGAAAGCCCAGGCGGAGGGGGATGGGCCCGCGGCCTGTTAGCTCGTTGGCGGGGTAACG<br>GCCCACCAAGGCGATGATGGGTAGCCGGGTTGAGAGACCGACCGGCCAGATTGGGACTGAGACACGGCCCAGACTCCTA<br>CGGGAGGCAGCAGTGGGGAATCTTGCACAATGGGCGAAAGCCTGATGCAGCGACGCCGCGTGCGGGACGAAGGCCTTC<br>GGGTTGTAAACCGCTTTCAGCAGGGACGAGGCGAAAGTGACGGTACCTGCAGAAGAAGCCCCGGCTAACTACGTGCCAG<br>CAGCCGCGGTAATACGTAGGGGGCAAGCGTTATCCGGATTCATTGGGCGTAAAGCGCTCGTAGGCGGTCTGTTAGGTCG<br>GGAGTTAAATCCGGAGGCTCAACCTCCGTTCGCTCCCGATACCGGCAGACTTGAGTTTGGTAGGGGAAGGTGGAATTCCT<br>AGTGTAGCGGTGGAATGCGCAGATATTAGGAAGAACACCAGTGGCGAAGGCGGCCTTCTGGGCCATAACTGACGCTGAGG<br>AGCGAAAGCTAGGGGAGCAAACAGGATTAGATACCCTGGTAGTCCTAGCTGTAAACGATGGACACTAGGTGTGGGAGAATA<br>CGTCTTCCGTGCCGCAGCTAACGCATTAAGTGTCCCGCCTGGGGAGTACGGCCGCAAGGCTAAAACTCAAAGGAATTGAC<br>GGGGACCCGCACAAGCAGCGGAGCATGTGGTTTAATTCGAAGCAACGCGAAGAACCTTACCAGGGCTTGACATGTAGGTG<br>AAGCGGCGGAAACGTCGTGGCCGAAAGGAGCCTACACAGGTGGTGCATGGCTGTCGTCAGCTCGTGTCGTGAGATGTTA<br>GGTTAAGTCCTGCAACGAGCGCAACCCTCGTCGTATATTGCCAGCGGTTCGGCCGGGCACCTATACGAGACCGCCGGCG<br>TCAAGCCGGAGGAAGGTGGGGACGACGTCAAGTCATCATGCCCCTTATGTCCTGGGCTACACACGTGCTACAATGGCCG<br>GCACAATGAGCTGCCAACCCGCAAGGGCGAGCGAATCTCTAAAGCCGGTCCCAGTTCGGATTGGAGGCTGCAACCCGCC<br>TCCATGAAGTCGGAGTTGCTAGTAATCGCGGATCAGCACGCCGCGGTGAATGCGTTCCCGGGTCTTGTACACACCGCCC<br>GTCACACCACCCGAGTCGATTGCACCCGAAGTCGTCGGCCTAACCTTTTAGGAGGGAGATGCCGAAGGTGTGGTTGGTAA<br>GGGGGGTG

Sr no.	Species_OTUs	16S reference sequence
177	Capnocytophaga sp. OTU_1010	GATGAACGCTAGCGGCAGGCCTAACACATGCAAGTCGAGGGGGAGGTTACTTTCGGGTGACTGAGACCGGCGTACGGGT<br>GCGTAACGCGTATACAATCTGCCTTTCACTGGGGGATAGCCCGAAGAAATTTGGATTAATACCCCATAGTATATAGGGACGG<br>CATCGTTTTTATATTAAAGCTACGGTGGTGAAAGATGAGTATGCGTTCTATTAGCTAGTTGGAGAGGTAACGGCTCCCCAAG<br>GCGATGATAGATAGGGGTTCTGAGAGGGATGTCCCCCACACTGGTACTGAGATACGGACCAGACTCCTACGGGAGGCAGC<br>AGTGAGGAATATTGGACAATGGTCGGAAGACTGATCCAGCCATGCCGCGTGCAGGATGAAGGTCTTATGGATTGTAAACTG<br>CTTTTGTAAGGGAAGAATAAGGACTACGTGTAGTTTGATGACGGTACCTTATGAATAAGCATCGGCTAACTCCGTGCCAGCA<br>GCCGCGGTAATACGGAGGATGCGAGCGTTATCCGGAATCATTGGGTTTAAAGGGTCTGTAGGCGGGCTTGTAAGTCAGAG<br>GTGAAAGCGCTCAGCTCAACTGAGCAACTGCCTTTGAAACTGTTGGTCTTGAATGGTTGTGAAGTAGTTGGAATGTGTAGTG<br>TAGCGGTGAAATGCTTAGATATTACACAGAACACCGATAGCGAAGGCATATTACTAACAATTAATTGACGCTGATGGACGAAA<br>GCGTGGGGAGCGAACAGGATTAGATACCCTGGTAGTCCACGCTGTAAACGATGGATACTAGCTGTTTGGACGCGAGTTTGA<br>GTGGCTAAGCGAAAGTGATAAGTATCCCACCTGGGGAGTACGTTCGCAAGAATGAAACTCAAAGGAATTGACGGGGGCCCG<br>CACAAGCGGTGGAGCATGTGGTTTAATTCGATGATACGCGAGGAACCTTACCCGGGCTTGAATTGCAGAGGAAGAATTTAG<br>AGATAATGACGCCCTTCGGGGTCTCTGTGAAGGTGCTGCATGGTTGTCGTCAGCTCGTGCCGTGAGGTGTCGGCTTAAGT<br>GCCATAACGAGCGCAACCCCTCTCTTCAGTTGCCATCAGGTGATGCTGGGCACTCTGGAGACACTGCCACCGTAAGGTGT<br>GAGGAAGGTGGGGATGACGTCAAATCAGCACGGCCCTTACGTCCGGGGCTACACACGTGTTACAATGGCCGGTACAGAG<br>GGAGGGTGTAATGTAAATTGCATCAAATCTTGAAAGCCGGTCCCAGTTCGGACTGGGGTCTGCAACCCGACCCCACGAAG<br>CTGGATTCGCTAGTAATCGCGCATCAGCCATGGCGCGGTGAATACGTTCCCGGGCCTTGTACACACCGCCCGTCAAGCC<br>ATGAAAGCCGGGGGTGCCTGAAGTCCGTGACCGCAAGGATCGGCCTAGGGCAAAACTGGTGATTGGGGCT
178	Granulicatella sp. OTU_1014	GACGAACGCTGGCGGCGTGCCTAATACATGCAAGTCGAACGAGAGCGACCGGTGCTTGCACTGGTCAATCTAGTGGCGAA<br>CGGGTGAGTAACACGTGGGTAACCTGCCCATCAGAGGGGGATAACATCCGGAAACGGATGCTAAAACCGCATAGGTCTTC<br>GAGCCGCATGGCTTGAAGAGGAAAAGAGGCGCAAGCTTCTGCTGATGGATGGACCCGCGGTGCATTAGCTAGTTGGTGAG<br>GTAACGGCTCACCAAGGCCGTGATGCATAGCCGACCTGAGAGGGTGATCGGCCACATTGGGACTGAGACACGGCCCAAA<br>CTCCTACGGGAGGCAGCAGTAGGGAATCTTCCGCAATGGACGCAAGTCTGACGGAGCAACGCCGCGTGAGTGAAGAAGG<br>TTTTCGGATCGTAAAACTCTGTTGTTAGAGAAGAACAAGTGCTAGAGTAACTGTTAGCGCCTTGACGGTATCTAACCAGAAAG<br>CCACGGCTAACTACGTGCCAGCAGCCGCGGTAATACGTAGGTGGCAAGCGTTGTCCGGATTTATTGGGCGTAAAGCGAGC<br>GCAGGCGGTTCCTTAAGTCTGATGTGAAAGCCCCCGGCTCAACCGGGGAGGGTCATTGGAAACTGGGGAACTTGAGTGCA<br>GAAGAGGAGAGTGGAATTCCATGTGTAGCGGTGAAATGCGTAGATATATGGAGGAACACCAGTGGCGAAGGCGACTCTCTG<br>GTCTGTAACTGACGCTGAGGCTCGAAAGCGTGGGGAGCAAACAGGATTAGATACCCTGGTAGTCCACGCCGTAAACGATG<br>AGTGCTAGGTGTTAGACCCTTTCCGGGGTTTAGTGCCGCAGCTAACGCATTAAGCACTCCGCCTGGGGAGTACGACCGCA<br>AGGTTGAAACTCAAAGGAATTGACGGGGGCCCGCACAAGCGGTGGAGCATGTGGTTTAATTCGAAGCAACGCGAAGAACCT<br>TACCAGGTCTTGACATCCCTCTGACCGCTCTAGAGATAGAGCTTTCCTTCGGGACAGAGGTGACAGGTGGTGCATGGTTG<br>TCGTCAGCTCGTGTCGTGAGATGTTGGGTTAAGTCCCGCAACGAGCGCAACCCCTATTGTTAGTTGCCATCATTCAGTTGG<br>GCACTCTAGCGAGACTGCCGGTAATAAACCGGAGGAAGGTGGGGATGACGTCAAATCATCATGCCCCTTATGACCTGGGC<br>TACACACGTGCTACAATGGCTGGTACAACGAGTCGCAAGCCGGTGACGGCAAGCTAATCTCTTAAAGCCAGTCTCAGTTCG<br>GATTGTAGGCTGCAACTCGCCTACATGAAGTCGGAATCGCTAGTAATCGCGGATCAGCACGCCGCGGTGAATACGTTCCC<br>GGGCCTTGTACACACCGCCCGTCACACCACGAGAGTTTGTAACACCCGAAGTCGGTGAGGTAACCTTTTAGGAGCCAGCC<br>GCCTAAGGTGGGATAGATGATTGGGGTG
179	Peptostreptococcus sp. OTU_1016	GATGAACGCTGGCGGCGTGCCTAACACATGCAAGTCGAGCGAGGGTTTGCTCAGTATTGAGTATTCTAAGTCTAGAATGTT<br>CAATTCTGAGCAAAACCAAGCGGCGGACGGGTGAGTAACGCGTGGGTAACCTGCCCTATACACATGGATAACATACTGAAA<br>AGTTTACTAATACATGATAATATATATTTGCGGCATCGCAGATATATCAAAGTGTTAGCGGTATAGGATGGACCCGCGTCTGA<br>TTAGCTAGTTGGTGAGATAACTGCCCACCAAGGCGACGATCAGTAGCCGACCTGAGAGGGTGATCGGCCACATTGGAACT<br>GAGACACGGTCCAAACTCCTACGGGAGGCAGCAGTGGGGAATATTGCACAATGGGCGAAAGCCTGATGCAGCAACGCCG<br>CGTGAACGATGAAGGTCTTCGGATCGTAAAGTTCTGTTGCAGGGGAAGATAATGACGGTACCCTGTGAGGAAGCCCCGGC<br>TAACTACGTGCCAGCAGCCGCGGTAATACGTAGGGGGCTAGCGTTATCCGGATTTACTGGGCGTAAAGGGTGCGTAGGTG<br>GTCCTTCAAGTCGGTGGTTAAAGGCTACGGCTCAACCGTAGTAAGCCGCCGAAACTGGAGGACTTGAGTGCAGGAGAGGA<br>AAGTGGAATTCCCAGTGTAGCGGTGAAATGCGTAGATATTGGGAGGAACACCAGTAGCGAAGGCGGCTTTCTGGACTGCAA<br>CTGACACTGAGGCACGAAAGCGTGGGTAGCAAACAGGATTAGATACCCTGGTAGTCCACGCTGTAAACGATGAGTACTAGG<br>TGTCGGGGGTTACCCCCCTCGGTGCCGCAGCTAACGCATTAAGTACTCCGCCTGGGGAGTACGCACGCAAGTGTGAAAC<br>TCAAAGGAATTGACGGGGACCCGCACAAGTAGCGGAGCATGTGGTTTAATTCGAAGCAACGCGAAGAACCTTACCTAAGCT<br>TGACATCCCTCGGACAGGTGTTTAATCACACCCTTCCTTCGGGACTGAGGTGACAGGTGGTGCATGGTTGTCGTCAGCTC<br>GTGTCGTGAGATGTTGGGTTAAGTCCCGCAACGAGCGCAACCCCTATTGTTAGTTGCCATCATTCAGTTGGGCACTCTAGC<br>GAGACTGCCGGTAATAAACCGGAGGAAGGTGGGGATGACGTCAAATCATCATGCCCCTTATGACCTGGGCTACACACGTG<br>CTACAATGGCTGGTACAACGAGTCGCAAGCCGGTGACGGCAAGCTAATCTCTTAAAGCCAGTCTCAGTTCGGATTGTAGGC<br>TGCAACTCGCCTACATGAAGTCGGAATCGCTAGTAATCGCGGATCAGCACGCCGCGGTGAATACGTTCCCGGGCCTTGTA<br>CACACCGCCCGTCACACCACGAGAGTTTGTAACACCCGAAGTCGGTGAGGTAACCTTTTAGGAGCCAGCCGCCTAAGGTG<br>GGATAGATGATTGGGGTG
180	Porphyromonas sp. OTU_1037	GATGAACGCTAGCGATAGGCTTAACACATGCAAGTCGAGGGGCAGCATGATCTTAGCTTGCTAAGGTCGATGGCGACCGG<br>CGCACGGGTGCGTAACGCGTATGCAACTTGCCTTACAGAGGGGGATAACCCGTTGAAAGACGGACTAAAACCGCATACAC<br>TTGTATTATTGCATGATATTACAAGGAAATATTTATAGCTGTAAGATAGGCATGCGTCCCATTAGCTGGTTGGTGAGGTAACG<br>GCTCACCAAGGCGACGATGGGTAGGGGAACTGAGAGGTTTATCCCCCACACTGGTACTGAGACACGGACCAGACTCCTAC<br>GGGAGGCAGCAGTGAGGAATATTGGTCAATGGGCGAGAGCCTGAACCAGCCAAGTCGCGTGAAGGAAGACAGTCCTAAGG<br>ATTGTAAACTTCTTTTATACGGGAATAACGGGCGATACGAGTATTGCATTGAATGTACCGTAAGAATAAGCATCGGCTAACTC<br>CGTGCCAGCAGCCGCGGTAATACGGAGGATGCGAGCGTTATCCGGATTTATTGGGTTTAAAGGGTGCGTAGGTTGTTCGG<br>TAAGTCAGCGGTGAAACCTGAGCGCTCAACGTTCAGCCTGCCGTTGAAACTGCCGGGCTTGAGTTCAGTGGCGGCAGGC<br>GGAATTCGTGGTGTAGCGGTGAAATGCATAGATATCACGAGGAACTCCGATTGCGAAGGCAGCTTGCCATACTGCGACTGA<br>CACTGAAGCACGAAGGCGTGGGTATCAAACAGGATTAGATACCCTGGTAGTCCACGCAGTAAACGATGATTACTAGGAGTTT<br>GCGATATACCGTCAAGCTTCCACAGCGAAAGCGTTAAGTAATCCACCTGGGGAGTACGCCGGCAACGGTGAAACTCAAAG<br>GAATTGACGGGGGCCCGCACAAGCGGAGGAACATGTGGTTTAATTCGATGATACGCGAGGAACCTTACCCGGGATTGAAAT<br>TTAGATGGCGGCAGATGAGAGTTTGCTTTCCTTCGGGACATCTAAGTAGGTGCTGCATGGTTGTCGTCAGCTCGTGCCGT<br>GAGGTGTCGGCTTAAGTGCCATAACGAGCGCAACCCGCGTCGATAGTTACTAACAGGTCATGCTGAGGACTCTATCGAGA<br>CAGCCGTCGTAAGACGTGAGGAAGGGGCGGATGACGTCAAATCAGCACGGCCCTTACATCCGGGGCGACACACGTGTTA<br>CAATGGCAGGGACAAAGGGAAGCGACATGGTGACATGAAGCGGATCTCCAAACCCTGTCCCAGTTCGGATCGGAGTCTGC<br>AACTCGACTCCGTGAAGCTGGATTCGCTAGTAATCGCGCATCAGCCATGGCGCGGTGAATACGTTCCCGGGCCTTGTACA<br>CACCGCCCGTCAAGCCATGGAAGTTGGGAGTACCTGAAGAGCGTAACCGCAAGGGGCGCTTAAGGGTAATACCAGTGACT<br>GGGGCT

Sr no.	Species_OTUs	16S reference sequence
181	Gemella sp. OTU_1053	AGTGAACGCTGGCGGCGTGCCTAATACATGCAAGTCGAACGGAGATTAAGTAGCTTGCTATTTAATCTTAGTGGCGCACGGGTGAGTAATGTATAGTTAATCTGCCCCATAGTGGAGGACAACAGTTAGAAATGACTGCTAATACTCCATACTCCTTCTTATCATAAGTTAAGTCGGGAAAGTTTTTCGCTATGGGATGAGACTATATCGTATCAGCTAGTTGGTAAGGTAATGGCTTACCAAGGCTATGACGCGTAACTGGTCTGAGAGGATGATCAGTCACATTGGAACTGAGACACGGTCCAAACTCCTACGGGAGGCAGCAGTAGGGAATATTGCGCAATGGGGGAAACCCTGACGCAGCAACGCCGCGTGGAGGATGACACTTTTCGGAGCGTAAACTCCTTTTGTTAGGGAAGAATAATGACGGTACCTAACCAGAAAGCCACGGCTAACTACGTGCCAGCAGCCGCGGTAATACGTAGGTGGCAAGCGTTGTCCGGAATTATTGGGCGTAAAGCGCGCGCAGGTGGTTTAATAAGTCTGATGTGAAAGCCCACGGCTCAACCGTGGAGGGTCATTGGAAACTGTTAAACTTGAGTGCAGGAGAGAAAAGTGGAATTCCTAGTGTAGCGGTGAAATGCGTAGAGATTAGGAGGAACACCAGTGGCGAAGGCGGCTTTTTGGCCTGTAACTGACACTGAGGCGCGAAAGCGTGGGGAGCAAACAGGATTAGATACCCTGGTAGTCCACGCCGTAAACGATGAGTGCTAAGTGTTGGTCTCATAAGAGATCAGTGCTGCAGCTAACGCATTAAGCACTCCGCCTGGGGAGTACGACCGCAAGGTTGAAACTCAAAGGAATTGACGGGGACCCGCACAAGCGGTGGAGCATGTGGTTTAATTCGAAGCAACGCGAAGAACCTTACCAAGTCTTGACATACTGTGAGGACAGAAGAGATTTTGTTGTTCTGACCTATGGTTAGACACAGATACAGGTGGTGCATGGTTGTCGTCAGCTCGTGTCGTGAGATGTTGGGTTAAGTCCCGCAACGAGCGCAACCCTTATATCTAGTTGCCAGCAGTAAGATGGGGACTCTAGATAGACTGCCAGTGATAAACTGGAGGAAGGTGGGGATGACGTCAAATCATCATGCCCCTTATGACTTGGGCTACACACGTGCTACAATGGATAGGAACAAAGAGAAGCGAGCTCGCGAGAGTCAGCCAACCTCATAAAACTATTCTCAGTTCGGATTGTAGTCTGCAACTCGACTACATGAAGCTGGAATCGCTAGTAATCGCGAATCAGAATGTCGCGGTGAATACGTTCCCGGGTCTTGTACACACCGCCCGTCACACCACGAGAGTTTGTAACACCCGAAGACGGTGGCCTAACCTATAAGGAGGGAGCCGGTCACGGTGGGACAGATGATTGGGGTG
182	Streptococcus sp. OTU_1061	GACGAACGCTGGCGGCGTGCCTAATACATGCAAGTAGAACGCTGAAGAGAGGAGCTTGCTCTTCTTGGATGAGTTGCGAACGGGTGAGTAACGCGTAGGTAACCTGCCTGGTAGCGGGGGATAACTATTGGAAACGATAGCTAATACCGCATAATAGTAGATGTTGCATGACATTTGCTTAAAAGGTGCAATTGCATCACTACCAGATGGACCTGCGTTGTATTAGCTAGTTGGTGAGGTAACGGCTCACCAAGGCAACGATACATAGCCGACCTGAGAGGGTGATCGGCCACACTGGGACTGAGACACGGCCCAGACTCCTACGGGAGGCAGCAGTAGGGAATCTTCGGCAATGGACGGAAGTCTGACCGAGCAACGCCGCGTGAGTGAAGAAGGTTTTCGGATCGTAAAGCTCTGTTGTAAGAGAAGAACGAGTGTGAGAGTGGAAAGTTCACACTGTGACGGTATCTTACCAGAAAGGGACGGCTAACTACGTGCCAGCAGCCGCGGTAATACGTAGGTCCCGAGCGTTGTCCGGATTTATTGGGCGTAAAGCGAGCGCAGGCGGTTAGATAAGTCTGAAGTTAAAGGCTGTGGCTTAACCATAGTACGCTTTGGAAACTGTTTAACTTGAGTGCAAGAGGGGAGAGTGGAATTCCATGTGTAGCGGTGAAATGCGTAGATATATGGAGGAACACCGGTGGCGAAAGCGGCTCTCTGGCTTGTAACTGACGCTGAGGCTCGAAAGCGTGGGGAGCAAACAGGATTAGATACCCTGGTAGTCCACGCCGTAAACGATGAGTGCTAGGTGTTAGACCCTTTCCGGGGTTTAGTGCCGCAGCTAACGCATTAAGCACTCCGCCTGGGGAGTACGACCGCAAGGTTGAAACTCAAAGGAATTGACGGGGGCCCGCACAAGCGGTGGAGCATGTGGTTTAATTCGAAGCAACGCGAAGAACCTTACCAGGTCTTGACATCCCTCTGACCGCTCTAGAGATAGAGCTTTCCTTCGGGACAGAGGTGACAGGTGGTGCATGGTTGTCGTCAGCTCGTGTCGTGAGATGTTGGGTTAAGTCCCGCAACGAGCGCAACCCTTGTCCCGTGTTGCCAGCACGTTGTGGTGGGGACTCGCGGGAGACTGCCGGGGTCAACTCGGAGGAAGGTGGGGATGACGTCAAATCATCATGCCCCTTATGTCTTGGGCTTCACGCATGCTACAATGGCCGGTACAGAGGGCTGCGATACCGTGAGGTGGAGCGAATCCCTTAAAGCCGGTCTCAGTTCGGATCGGTGTCTGCAACTCGACACCGTGAAGTTGGAGTCGCTAGTAATCGCAGATCAGCAACGCTGCGGTGAATACGTTCTCGGGCCTTGTACACACCGCCCGTCACGTCATGAAAGTCGGCAACACCCGAAGCCCGTGGCCCTACGGGGAGCGGTCGAAGGTGGGGCTGGTGATTGGGACG
183	Streptococcus sp. OTU_1063	GACGAACGCTGGCGGCGTGCCTAATACATGCAAGTAGAACGCTGAAGGAGGAGCTTGCTCTTCTGGATGAGTTGCGAACGGGTGAGTAACGCGTAGGTAACCTGCCTGGTAGCGGGGGATAACTATTGGAAACGATAGCTAATACCGCATAATAGTAGATGTTGCATGACATTTGCTTAAAAGGTGCAATTGCATCACTACCAGATGGACCTGCGTTGTATTAGCTAGTTGGTGAGGTAACGGCTCACCAAGGCAACGATACATAGCCGACCTGAGAGGGTGATCGGCCACACTGGGACTGAGACACGGCCCAGACTCCTACGGGAGGCAGCAGTAGGGAATCTTCGGCAATGGACGGAAGTCTGACCGAGCAACGCCGCGTGAGTGAAGAAGGTTTTCGGATCGTAAAGCTCTGTTGTAAGAGAAGAACGAGTGTGAGAGTGGAAAGTTCACACTGTGACGGTATCTTACCAGAAAGGGACGGCTAACTACGTGCCAGCAGCCGCGGTAATACGTAGGTCCCGAGCGTTGTCCGGATTTATTGGGCGTAAAGCGAGCGCAGGCGGTTAGATAAGTCTGAAGTTAAAGGCTGTGGCTTAACCATAGTACGCTTTGGAAACTGTTTAACTTGAGTGCAAGAGGGGAGAGTGGAATTCCATGTGTAGCGGTGAAATGCGTAGATATATGGAGGAACACCGGTGGCGAAAGCGGCTCTCTGGCTTGTAACTGACGCTGAGGCTCGAAAGCGTGGGGAGCAAACAGGATTAGATACCCTGGTAGTCCACGCCGTAAACGATGAGTGCTAGGTGTTAGACCCTTTCCGGGGTTTAGTGCCGCAGCTAACGCATTAAGCACTCCGCCTGGGGAGTACGACCGCAAGGTTGAAACTCAAAGGAATTGACGGGGGCCCGCACAAGCGGTGGAGCATGTGGTTTAATTCGAAGCAACGCGAAGAACCTTACCAGGTCTTGACATCCCTCTGACCGCTCTAGAGATAGAGCTTTCCTTCGGGACAGAGGTGACAGGTGGTGCATGGTTGTCGTCAGCTCGTGTCGTGAGATGTTGGGTTAAGTCCCGCAACGAGCGCAACCCCTATTGTTAGTTGCCATCATTCAGTTGGGCACTCTAGCGAGACTGCCGGTAATAAACCGGAGGAAGGTGGGGATGACGTCAAATCATCATGCCCCTTATGACCTGGGCTACACACGTGCTACAATGGCTGGTACAACGAGTCGCAAGCCGGTGACGGCAAGCTAATCTCTTAAAGCCAGTCTCAGTTCGGATTGTAGGCTGCAACTCGCCTACATGAAGTCGGAATCGCTAGTAATCGCGAATCAGCAACGTCGCGGTGAATGCGTTCCCGGGCCTTGTACACACCGCCCGTCAAGTCATGAAAGTGGGCAGCACCCGAAGCCGGTGGCCTAACCCCTTGTGGGATGGAGCCGTCTAAGGTGAGGCTCGTGATTGGGACT
184	Bifidobacterium sp. OTU_1065	GACGAACGCTGGCGGCGTGCTTAACACATGCAAGTCGAACGCTGAAGCTCAGCTTGCTGGGTGGATGAGTGGCGAACGGGTGAGTAACACGTGAGTAACCTGCCCCCTTCTTTGGGATAACGCCCGGAAACGGGTGCTAATACTGGATATTCACTGATCTTCGCATGGGGGTTGGTGGAAAGGTTTTTTCTGGTGGGGGATGGGCTCGCGGCCTATCAGCTTGTTGGTGGGGTGATGGCCTACCAAGGCTTTGACGGGTAGCCGGCCTGAGAGGGTGACCGGTCACATTGGGACTGAGATACGGCCCAGACTCCTACGGGAGGCAGCAGTGGGGAATATTGCACAATGGGCGCAAGCCTGATGCAGCGACGCCGCGTGCGGGATGGAGGCCTTCGGGTTGTAAACCGCTTTTGATCGGGAGCAAGCCCTTCGGGGTGAGTGTACCTTTCGAATAAGCACCGGCTAACTACGTGCCAGCAGCCGCGGTAATACGTAGGGTGCAAGCGTTATCCGGAATTATTGGGCGTAAAGGGCTCGTAGGCGGTTCGTCGCGTCCGGTGTGAAAGCCCATCGCTTAACGGTGGGTCTGCGCCGGGTACGGGCGGGCTGGAGTGCGGTAGGGGAGACTGGAATTCCCGGTGTAACGGTGGAATGTGTAGATATCGGGAAGAACACCAATGGCGAAGGCAGGTCTCTGGGCCGTCACTGACGCTGAGGAGCGAAAGCGTGGGGAGCGAACAGGATTAGATACCCTGGTAGTCCACGCCGTAAACGGTGGATGCTGGATGTGGGGCCCGTTCCACGGGTTCCGTGTCGGAGCTAACGCGTTAAGCATCCCGCCTGGGGAGTACGGCCGCAAGGCTAAAACTCAAAGAAATTGACGGGGGCCCGCACAAGCGGCGGAGCATGCGGATTAATTCGATGCAACGCGAAGAACCTTACCTGGGCTTGACATGTTCCCGACAGCCGTAGAGATATGGCCTCCCTTCGGGGCGGGTTCACAGGTGGTGCATGGTCGTCGTCAGCTCGTGTCGTGAGATGTTGGGTTAAGTCCCGCAACGAGCGCAACCCTCGCCCTGTGTTGCCAGCACGTCATGGTGGGAACTCACGGGGGACCGCCGGGGTCAACTCGGAGGAAGGTGGGGATGACGTCAGATCATCATGCCCCTTACGTCCAGGGCTTCACGCATGCTACAATGGCCGGTACAGCGGGATGCGACATGGCGACATGGAGCGGATCCCTGAAAACCGGTCTCAGTTCGGATTGGAGTCTGCAACCCGACTCCATGAAGGCGGAGTCGCTAGTAATCGCGGATCAGCAACGCCGCGGTGAATGCGTTCCCGGGCCTTGTACACACCGCCCGTCAAGTCATGAAAGTGGGCAGCACCCGAAGCCGGTGGCCTAACCCCTTGTGGGATGGAGCCGTCTAAGGTGAGGCTCGTGATTGGGACT

Sr no.	Species_OTUs	16S reference sequence
185	Streptococcus sp. OTU_1070	ATTGAACGCTGGCGGCAGGCCTAACACATGCAAGTCGAGCGGTAGAGAGAAGCTTGCTTCTCTTGAGAGCGGCGGACGG<br>GTGAGTAATGCCTAGGAATCTGCCTGGTAGTGGGGGATAACTATTGGAAACGATAGCTAATACCGCATAAGAGTAGATGTTG<br>CATGACATTTGCTTAAAAGGTGCAATTGCATCACTACCAGATGGACCTGCGTTGTATTAGCTAGTTGGTGAGGTAACGGCTC<br>ACCAAGGCAACGATACATAGCCGACCTGAGAGGGTGATCGGCCACACTGGGACTGAGACACGGCCCAGACTCCTACGGG<br>AGGCAGCAGTAGGGAATCTTCGGCAATGGACGGAAGTCTGACCGAGCAACGCCGCGTGAGTGAAGAAGGTTTTCGGATCG<br>TAAAGCTCTGTTGTAAGAGAAGAACGAGTGTGAGAGTGGAAAGTTCACACTGTGACGGTATCTTACCAGAAAGGGACGGCTA<br>ACTACGTGCCAGCAGCCGCGGTAATACGTAGGTCCCGAGCGTTGTCCGGATTTATTGGGCGTAAAGCGAGCGCAGGCGG<br>TTAGATAAGTCTGAAGTTAAAGGCTGTGGCTTAACCATAGTACGCTTTGGAAACTGTTTAACTTGAGTGCAAGAGGGGAGAGT<br>GGAATTCCATGTGTAGCGGTGAAATGCGTAGATATATGGAGGAACACCGGTGGCGAAAGCGGCTCTCTGGCTTGTAACTGA<br>CGCTGAGGCTCGAAAGCGTGGGGAGCAAACAGGATTAGATACCCTGGTAGTCCACGCCGTAAACGATGAGTGCTAGGTGT<br>TAGACCCTTTCCGGGGTTTAGTGCCGCAGCTAACGCATTAAGCACTCCGCCTGGGGAGTACGACCGCAAGGTTGAAACTC<br>AAAGGAATTGACGGGGGCCCGCACAAGCGGTGGAGCATGTGGTTTAATTCGAAGCAACGCGAAGAACCTTACCAGGTCTT<br>GACATCCCTCTGACCGCTCTAGAGATAGAGTTTTCCTTCGGGACAGAGGTGACAGGTGGTGCATGGTTGTCGTCAGCTCG<br>TGTCGTGAGATGTTGGGTTAAGTCCCGCAACGAGCGCAACCCCTATTGTTAGTTGCCATCATTCAGTTGGGCACTCTAGCG<br>AGACTGCCGGTAATAAACCGGAGGAAGGTGGGGATGACGTCAAATCATCATGCCCCTTATGACCTGGGCTACACACGTGC<br>TACAATGGCTGGTACAACGAGTCGCAAGCCGGTGACGGCAAGCTAATCTCTTAAAGCCAGTCTCAGTTCGGATTGTAGGCT<br>GCAACTCGCCTACATGAAGTCGGAATCGCTAGTAATCGCGGATCAGCACGCCGCGGTGAATACGTTCCCGGGCCTTGTAC<br>ACACCGCCCGTCACACCACGAGAGTTTGTAACACCCGAAGTCGGTGAGGTAACCTTTTAGGAGCCAGCCGCCTAAGGTGG<br>GATAGATGATTGGGGTG
186	Limosilactobacillus sp. OTU_1071	GATGAACGCCGGCGGTGTGCCTAATACATGCAAGTCGAGCGCACTGGCCCAACTGATATGACGTGCTTGCACTGAATTGA<br>CGTTGGATTCCCAGTGAGCGGCGGACGGGTGAGTAACACGTGGGCAACCTGCCCTGAAGCGGGGGATAACATCTGGAAA<br>CAGGTGCTAATACCGCATAACAACGAAAACCACATGGTTTTCGTTTCAAAGATGGCTTCGGCTATCACTTCAGGATGGGCC<br>CGCGGTGCATTAGCTAGTTGGTAAGGTAACGGCTTACCAAGGCGATGATGCATAGCCGAGTTGAGAGACTGATCGGCCAC<br>AATGGAACTGAGACACGGTCCATACTCCTACGGGAGGCAGCAGTAGGGAATCTTCCACAATGGGCGCAAGCCTGATGGAG<br>CAACACCGCGTGAGTGAAGAAGGGTTTCGGCTCGTAAAGCTCTGTTGTTAAAGAAGAACACGTATGAGAGTAACTGTTCATA<br>CGTTGACGGTATTTAACCAGAAAGTCACGGCTAACTACGTGCCAGCAGCCGCGGTAATACGTAGGTGGCAAGCGTTATCC<br>GGATTTATTGGGCGTAAAGAGAGTGCAGGCGGTTTTCTAAGTCTGATGTGAAAGCCTTCGGCTTAACCGGAGAAGTGCATC<br>GGAAACTGGATAACTTGAGTGCAGAAGAGGGTAGTGGAACTCCATGTGTAGCGGTGGAATGCGTAGATATATGGAAGAACAC<br>CAGTGGCGAAGGCGGCTACCTGGTCTGCAACTGACGCTGAGACTCGAAAGCATGGGTAGCGAACAGGATTAGATACCCTG<br>GTAGTCCATGCCGTAAACGATGAGTGCTAGGTGTTGGAGGGTTTCCGCCCTTCAGTGCCGGAGCTAACGCATTAAGCACT<br>CCGCCTGGGGAGTACGACCGCAAGGTTGAAACTCAAAGGAATTGACGGGGGCCCGCACAAGCGGTGGAGCATGTGGTTT<br>AATTCGAAGCTACGCGAAGAACCTTACCAGGTCTTGACATCTTGCGCCAACCCTAGAGATAGGGCGTTTCCTTCGGGAACG<br>CAATGACAGGTGGTGCATGGTCGTCGTCAGCTCGTGTCGTGAGATGTTGGGTTAAGTCCCGCAACGAGCGCAACCCTTGT<br>TACTAGTTGCCAGCATTAAGTTGGGCACTCTAGTGAGACTGCCGGTGACAAACCGGAGGAAGGTGGGGACGACGTCAGAT<br>CATCATGCCCCTTATGACCTGGGCTACACACGTGCTACAATGGACGGTACAACGAGTCGCGAACTCGCGAGGGCAAGCAA<br>ATCTCTTAAAACCGTTCTCAGTTCGGACTGCAGGCTGCAACTCGCCTGCACGAAGTCGGAATCGCTAGTAATCGCGGATCA<br>GCATGCCGCGGTGAATACGTTCCCGGGCCTTGTACACACCGCCCGTCACACCATGAGAGTTTGTAACACCCAAAGTCGGT<br>GGGGTAACCTTTTAGGAGCCAGCCGCCTAAGGTGGGACAGATGATTAGGGTG
187	Neisseriaceae gen. sp. OTU_1079	GACGAACGCTGGCGGCGTGCCTAATACATGCAAGTAGAACGCTGAAGAGAGGAGCTTGCTCTTCTTGGATGAGTTGCGAA<br>CGGGTGAGTAACGCGTAGGTAACCTGCCTGGTAGCGGGGGATAACTATTGGAAACGATAGCTAATACCGCATGATATTAATT<br>GTCGCATGATAATTAATTGAAAGATGCAATTGCATCACTACCAGATGGACCTGCGTTGTATTAGCTAGTTGGTGAGGTAACG<br>GCTCACCAAGGCGACGATACATAGCCGACCTGAGAGGGTGATCGGCCACACTGGGACTGAGACACGGCCCAGACTCCTA<br>CGGGAGGCAGCAGTAGGGAATCTTCGGCAATGGGGGGAACCCTGACCGAGCAACGCCGCGTGAGTGAAGAAGGTTTTCG<br>GATCGTAAAGCTCTGTTGTAAGAGAAGAACGGGTGTGAGAGTGGAAAGTTCACACTGTGACGGTATCTTACCAGAAAGGGAC<br>GGCTAACTACGTGCCAGCAGCCGCGGTAATACGTAGGGTGCGAGCGTTAATCGGAATTACTGGGCGTAAAGCGGGCGCA<br>GACGGTTACTTAAGCAGGATGTGAAATCCCCGGGCTCAACCTGGGAATTGCGTTCTGAACTGGGTGGCTAGAGTGTGTCA<br>GAGGGGGGTAGAATTCCACGTGTAGCAGTGAAATGCGTAGAGATGTGGAGGAATACCGATGGCGAAGGCAGCCCCCTGGG<br>ATGGCACTGACGTTCATGCCCGAAAGCGTGGGTAGCAAACAGGATTAGATACCCTGGTAGTCCACGCCCTAAACGATGTC<br>GATTAGCTGTTGGGCAACTTGATTGCTTGGTAGCGAAGCTAACGCGTGAAATCGACCGCCTGGGGAGTACGGTCGCAAGA<br>TTAAAACTCAAAGGAATTGACGGGGACCCGCACAAGCGGTGGATGATGTGGATTAATTCGATGCAACGCGAAGAACCTTAC<br>CTGGTCTTGACATGTACGGAACCTTCCAGAGACGGAAGGGTGCCTTCGGGAGCCGTAACACAGGTGCTGCATGGCTGTC<br>GTCAGCTCGTGTCGTGAGATGTTGGGTTAAGTCCCGCAACGAGCGCAACCCTTGTCATTAGTTGCCATCATTTGGTTGGG<br>CACTCTAATGAGACTGCCGGTGACAAGCCGGAGGAAGGTGGGGATGACGTCAAGTCCTCATGGCCCTTATGACCAGGGCT<br>TCACACGTCATACAATGGTCGGTACAGAGGGTAGCCAAGCCGCGAGGTGGAGCCAATCCCAAAAAACCGATCGTAGTCCG<br>GATTGCACTCTGCAACTCGAGTGCATGAAGTCGGAATCGCTAGTAATCGCAGGTCAGCATACTGCGGTGAATACGTTCCCG<br>GGTCTTGTACACACCGCCCGTCACACCATGGGAGTGGGGGATACCAGAAGCAGGTAGGCTAACCGCAAGGGGGCCGCTT<br>GCCACGGTATGCTTCATGACTGGGGTG
188	Prevotella sp. OTU_1082	GATGAACGCTAGCTATAGGCTTAACACATGCAAGTCGAGGGGAAACGGCATTATGTGCTTGCACATTTTGGACGTCGACCG<br>GCGCACGGGTGAGTATCGCGTATCCAACCTTCCCTCCACTCGGGGATACCCCGTTGAAAGACGGCCTAATACCCGATGTT<br>GTCCACATATGGCATCTGACGTGGACCAAAGATTCATCGGTGGAGGATGGGGATGCGTCTGATTAGCTTGTTGGTGCGGG<br>TAACGGCCCACCAAGGCTACGATCAGTAGGGGTTCTGAGAGGAAGGTCCCCCACATTGGAACTGAGACACGGTCCAAACT<br>CCTACGGGAGGCAGCAGTGAGGAATATTGGTCAATGGACGTAAGTCTGAACCAGCCAAGTAGCGTGCAGGATTGACGGCC<br>CTATGGGTTGTAAACTGCTTTTGTTGGGGAGTAAAGTGAGGCACGCGTGCCTTTTTGCATTTACCCTTCGAATAAGGACCG<br>GCTAATTCCGTGCCAGCAGCCGCGGTAATACGGAAGGTCCAGGCGTTATCCGGATTTATTGGGTTTAAAGGGAGTGTAGG<br>CGGTCTGTTAAGCGTGTTGTGAAATTTAGGTGCTCAACATCTACCTTGCAGCGCGAACTGGCGGACTTGAGTGCACGCAAC<br>GTATGCGGAATTCATGGTGTAGCGGTGAAATGCTTAGATATCATGACGAACTCCGATTGCGAAGGCAGCGTACGGGAGTGT<br>TACTGACGCTTAAGCTCGAAGGTGCGGGTATCGAACAGGATTAGATACCCTGGTAGTCCGCACGGTAAACGATGGATGCC<br>CGCTGTTAGCGCCTGGCGTTAGCGGCTAAGCGAAAGCATTAAGCATCCCACCTGGGGAGTACGCCGGCAACGGTGAAAC<br>TCAAAGGAATTGACGGGGGCCCGCACAAGCGGAGGAACATGTGGTTTAATTCGATGATACGCGAGGAACCTTACCCGGGA<br>TTGAAATGTAGATGACGGATGGTGAAAACCGTCTTCCCTTCGGGGCTTCTATGTAGGTGCTGCATGGTTGTCGTCAGCTCG<br>TGCCGTGAGGTGTCGGCTTAAGTGCCATAACGAGCGCAACCCACATCGGTAGTTGCTAACAGTTTTCGCTGAGGACTCTA<br>CCGAGACTGCCGTCGTAAGGCGCGAGGAAGGTGTGGATGACGTCAAATCAGCACGGCCCTTACATCCGGGGCGACACAC<br>GTGTTACAATGGGAGGGACAAAGGGCAGCTACCGGGCGACCGGATGCGAATCTCTAAACCCTTCCCCAGTTCGGATCGGA<br>GTCTGCAACTCGACTCCGTGAAGCTGGATTCGCTAGTAATCGCGCATCAGCCATGGCGCGGTGAATACGTTCCCGGGCC<br>TTGTACACACCGCCCGTCAAGCCATGGGAGTCGGGGGTACCTGAAGGGCGTAACCGCAAGGGGCGCACTAGGGTAATAC<br>CGGTGACTGGGGCT

Sr no.	Species_OTUs	16S reference sequence
189	Saccharibacteria sp. OTU_1088	GACGAACGCTGGCGGCGTGCTTAACACATGCAAGTCGAACGGGCCTGCCCTTTTTTGGGTGGGTTAGTGGCGAACGGGT<br>GAGTATCACGTGAGTAACCTGCCCCCTGCTCCTGGATAACGCTCTGAAAGGGGTGCTAATACGGGGTGTGCTGGTGCTGC<br>CGCATGGTGGTGCCGGGATAGGTTCCCCTTTTTTGGGGTTCTGGTGGGGGATGGGCTCGCGGCCTATCAGCTTGTTGGT<br>GGGGTGATGGCCTACCAAGGCGGTGACGGGTAGCCGGCCTGAGAGGGTGGACGGCCACACTGGGACTGAGACACGGCC<br>CAGACTCCTGCGGGAGGCAGCAGTGGGGGGTATTGCGCAATGGGCGGAAGCCTGACGCAGCGACGCCGCGTGAGGGAC<br>GGAGGCCTTCGGGTTGTGAACCTCTTTCGCCGGTGGAGAAGGCGCCCCCGTGTGGGGTGTTGACTGTAGCCGGGTTAAG<br>AAGCGCCGGCTAACTACGTGCCAGCAGCCGCGGTAATACGTAGGGCGCGAGCGTTGTCCGGAGTTACTGGGCGTAAAGA<br>GTTGCGTAGGCGGTTGTATAAGTGAATAGTGAAATCTGGTGGCTCAACCATACAGGCTATTGTTCAAACTGTACAACTTGAG<br>AGTGGTAGAGGTCACTGGAATTTCTTGTGTAGGAGTGAAATCCGTAGATATAAGAAGGAACACCAATGGCGTAGGCAGGTGA<br>CTGGACCATTTCTGACGCTAAGGCACGAAAGCGTGGGGAGCGAACCGGATTAGATACCCGGGTAGTCCACGCCGTAAACG<br>ATGGATACTGGCTGTTGGAGGTATCGACCCCTTCAGTAGCGAAGCTAACGCGTTAAGTATCCCGCCTGTGGAGTACGGCC<br>GCAAGGCTAAAACATAAAGGAATTGACGGGGACCCGCACAAGCGGTGGATCGTGTTCTTTAATTCGATGCTAAACGAAGAAC<br>CTTACCAGGGCTTGACATCCAGGGAATTTTTGGGAAACCAATTAGTGCCGTTTGGAACCCTGTGACAGGTGATGCATGGCC<br>GTCGTCAGCTCGTGTCGTGAGATGTTTGGTTAAGTCCATCAACGAGCGCAACCCTTGTGTCTAGTTGTATTTTTCTAGACAG<br>ACTGCTTCGGTAACGGAGAGGAAGGAGGGGATGATGTCAGGTCAGTATTTCCCTTACGTCCTGGGCTAGAAACACGATACA<br>ATGGCTAGTACAATGCGCAGCGAAGCCGCGAGGTGAAGCAAATCGCACCAAAGCTAGTCCCAGTTCGGATTGGAGGCTGA<br>AACTCGCCTCCATGAAGTCGGAATCGCTAGTAATCGCAAATCAGCAAGTTGCGGTGAATACGTTCCCGGGTCTTGTACACA<br>CCGCCCGTCAAACCATGAAAGTGACCAACACCCGAAGTCCGATTCGTCGGCCTAAGGTGGGGGGCATGATTGGGGTT
190	Leptotrichia sp. OTU_1089	GATGAACGCTGACAGAATGCTTAACACATGCAAGTCTTTGGCAAATCTGTGCTTGCACAGCATAGCCAAGGCGGACGGGTG<br>AGTAACGCGTAAAGAACTTGCCCTGCAGTCTGGGATAACAGACGGAAACGACTGATAAAACCTGATATAATCAAGCCCACGC<br>ATGTGGTGCTTGATGAAAAGAAATGCTGCAGGAGAGCTTTGCGTCCTATTAGCTTGTTGGTGGGGTAATGGCCCACCAAGG<br>CAATGATAGGTAGCCGGCCTGAGAGGGTGACCGGCCACAAGGGGACTGAGATACGGCCCTTACTCCTACGGGAGGCAGC<br>AGTGGGGAATATTGGACAATGGGGGCAACCCTGATCCAGCAATTCTGTGTGCACGAAGAAGGTTTTCGGATTGTAAAGTGC<br>TTTCAGCAGGGAAGAAGAAAGTGACGGTACCTGCAGAAGAAGCGACGGCTAAATACGTGCCAGCAGCCGCGGTAATACGTA<br>TGTCGCAAGCGTTATCCGGAATTATTGGGCATAAAGGGCATCTAGGCGGCCAGACAAGTCTGGGGTGAAAACTTGCGGCT<br>CAACCGCAAGCCTGCCCTGGAAACTGTTTGGCTAGAGTGCTGGAGAGGTGGACGGAACTGCACGAGTAGAGGTGAAATTC<br>GTAGATATGTGCAGGAATGCCGATGATGAAGATAGTTCACTGGACGGTAACTGACGCTGAAGTGCGAAAGCTGGGGGAGCG<br>AACAGGATTAGATACCCTGGTAGTCCCAGCCGTAAACGATGATTACTGGGTGTGGGCATGAAGAGTGTCCGTGCCGAAGCT<br>AATGCGATAAGTAATCCGCCTGGGGAGTACGGCCGCAAGGCTGAAACTCAAAGGAATTGACGGGGACCCGCACAAGCGGT<br>GGAGCATGTGGTTTAATTCGACGCAACGCGAGGAACCTTACCAGATCTTGACATCCCACGTATGCCTGCGAGAGCAGGCA<br>GTGCCTTCGGGAACGTGGAGACAGGTGGTGCATGGCTGTCGACAGCTCGTGTCGTGAGATGTTGGGTTAAGTCCCGCAA<br>CGAGCGCAACCCCTATCGCTAGTTGCCATCATTAAGTTGGGGACTCTAGCGAGACTGCCTGCGAAGAGCAGGAGGAAGGT<br>GGGGATGACGTCAAGTCATCATGCCCCTTATGATCTGGGCTACACACGTGCTACAATGGCCGGTACAAAGAGCTGCAAAAC<br>GGTAACGTTTAGCCAATCTTTAAAGCCGGTCCAAGTTCGGATTGAAGTCTGCAACTCGACTTCATGAAGCCGGAATCGCTA<br>GTAATCGCAGATCAGCAATGCTGCGGTGAATACGTTCTCGGGTCTTGTACACACCGCCCGTCACACCACGAGAGTTGTCT<br>GCACCTGAAGCCGCCGGTCCAACCGCAAGGAGGAAGGCGTCTAAGGTGTGGATAGTGATTGGGGTG
191	Leptotrichia sp. OTU_1090	GATGAACGCTGACAGAATGCTTAACACATGCAAGTCTTTGGCGAATCTGTGCTTGCACAGGCTAGCCAAGGCGGACGGGT<br>GAGTAACGCGTAAAGAACTTGCCCTGCAGACAGGGATAACAGACGGAAACGACTGATAATACCTGATACAATTGCCAGCAC<br>GCATGTGCCCGGCAATGAAAAGTGATGCTGCAGGAGAGCTTTGCGTCCTATTAGCTTGTTGGTGAGGTAATGGCTCACCAA<br>GGCGATGATAGGTAGCCGGCCTGAGAGGGTGAACGGCCACAAGGGGACTGAGATACGGCCCTTACTCCTACGGGAGGCA<br>GCAGTGGGGAATATTGGACAATGGGGGCAACCCTGATCCAGCAATTCTGTGTGCACGAAGAAGGTTTTCGGATTGTAAAGT<br>GCTTTCAGCAGGGAAGAAGAAAGTGACGGTACCTGCAGAAGAAGCGACGGCTAAATACGTGCCAGCAGCCGCGGTAATAC<br>GTATGTCGCAAGCGTTATCCGGAATTATTGGGCATAAAGGGCATCTAGGCGGCCAGACAAGTCTGGGGTGAAAACTTGCGG<br>CTCAACCGCAAGCCTGCCCTGGAAACTGTTTGGCTAGAGTGCTGGAGAGGTGGACGGAACTGCACGAGTAGAGGTGAAAT<br>TCGTAGATATGTGCAGGAATGCCGATGATGAAGATAGTTCACTGGACGGTAACTGACGCTGAAGTGCGAAAGCTGGGGGAG<br>CGAACAGGATTAGATACCCTGGTAGTCCACGCCGTAAACGATGAGTGCTAGGTGTTAGGCCCTTTCCGGGGCTTAGTGCC<br>GGAGCTAACGCAATAAGCACTCCGCCTGGGGAGTACGACCGCAAGGTTGAAACTCAAAGGAATTGACGGGGGCCCGCACA<br>AGCGGTGGAGCATGTGGTTTAATTCGAAGCAACGCGAAGAACCTTACCAGGTCTTGACATCCCGATGCTATTCTTAGAGATA<br>GGAAGTTACTTCGGTACATCGGAGACAGGTGGTGCATGGTTGTCGTCAGCTCGTGTCGTGAGATGTTGGGTTAAGTCCCG<br>CAACGAGCGCAACCCTTATTGTTAGTTGCCATCATTAAGTTGGGCACTCTAGCGAGACTGCCGGTAATAAACCGGAGGAAG<br>GTGGGGATGACGTCAAATCATCATGCCCCTTATGACCTGGGCTACACACGTGCTACAATGGTCGGTACAACGAGTTGCGA<br>GCCGGTGACGGCAAGCTAATCTCTGAAAGCCGATCTCAGTTCGGATTGGAGGCTGCAACTCGCCTCCATGAAGTCGGAAT<br>CGCTAGTAATCGCGGATCAGCACGCCGCGGTGAATACGTTCCCGGGCCTTGTACACACCGCCCGTCACACCACGAGAGT<br>TTGTAACACCCGAAGTCGGTGAGGTAACCTTTTAGGGGCCAGCCGCCTAAGGTGGGATGGATGATTGGGGTG
192	Leptotrichia sp. OTU_1091	GATGAACGCTGACAGAATGCTTAACACATGCAAGTCTTTGGCGAATCTGTGCTTGCACAGCCTAGCCAAGGCGGACGGGT<br>GAGTAACGCGTAAAGAACTTGCCCTGCAGACAGGGATAACAGACGGAAACGACTGATAATACCTGATACAATTGCCAGCAC<br>GCATGTGCCCGGCAATGAAAAGTGATGCTGCAGGAGAGCTTTGCGTCCTATTAGCTTGTTGGTGAGGTAACGGCTCACCAA<br>GGCGATGATAGGTAGCCGGCCTGAGAGGGTGAACGGCCACAAGGGGACTGAGATACGGCCCTTACTCCTACGGGAGGCA<br>GCAGTGGGGAATATTGGACAATGGGGGCAACCCTGATCCAGCAATTCTGTGTGCACGAAGAAGGTTTTCGGATTGTAAAGT<br>GCTTTCAGCAGGGAAGAAGAAAGTGACGGTACCTGCAGAAGAAGCGACGGCTAAATACGTGCCAGCAGCCGCGGTAATAC<br>GTATGTCGCAAGCGTTATCCGGAATTATTGGGCATAAAGGGCATCTAGGCGGCCAGACAAGTCTGGGGTGAAAACTTGCGG<br>CTCAACCGCAAGCCTGCCCTGGAAACTGTTTGGCTAGAGTGCTGGAGAGGTGGACGGAACTGCACGAGTAGAGGTGAAAT<br>TCGTAGATATGTGCAGGAATGCCGATGATGAAGATAGTTCACTGGACGGTAACTGACGCTGAAGTGCGAAAGCTGGGGGAG<br>CGAACAGGATTAGATACCCTGGTAGTCCCAGCCGTAAACGATGATTACTGGGTGTGGGCATGAAGAGTGTCTGTGCCGAAG<br>CTAATGCGATAAGTAATCCGCCTGGGGAGTACGGCCGCAAGGCTGAAACTCAAAGGAATTGACGGGGACCCGCACAAGCG<br>GTGGAGCATGTGGTTTAATTCGACGCAACGCGAGGAACCTTACCAGGGCTTGACATGGGCCGGTCGTCCGCGGAGACGC<br>GGGCTCCCCTTGCGGGGCCGGTCCGCAGGTGGTGCATGGTTGTCGTCAGCTCGTGTCGTGAGATGTTGGGTTAAGTCC<br>CGCAACGAGCGCAACCCCTGTCCCGTGTTGCCAGCACGTCGTGGTGGGGACTCGCGGGAGACTGCCGGGGTCAACTCG<br>GAGGAGGGTGGGGACGACGTCAAATCATCATGCCCCTTATGTCCTGGGCTTCACGCATGCTACAATGGCCGGTACAGAGG<br>GTTGCGATGCCGTGAGGCGGGGCGAATCCCTTAAAGCCGGTCTCAGTTCGGATCGGTGTCTGCAACTCGACACCGTGAA<br>GCTGGAGTCGCTAGTAATCGCGGATCAGCAACGCCGCGGTGAATACGTTCTCGGGCCTTGTACACACCGCCCGTCACGT<br>CATGAAAGCCGGTGACGCCCGAAGCCCGTGGCCCTACGGGGAGCGGTCGAAGGCGGGGCTGGTGATTGGGACG

Sr no.	Species_OTUs	16S reference sequence
193	Cardiobacterium sp. OTU_1092	GACGAACGCTGGCGGCGTGCCTAATACATGCAAGTGGGACGCAAGGAAACACACTGTGCTTGCACACCGTGTTTTCTTGA<br>GTCGCGAACGGGTGAGTAACGCGTAGGTAACCTGCCTATTAGCGGGGGATAACTATTGGAAACGATAGCTAATACCGCATA<br>ATATTAATTATTGCATGATAATTGATTGAAAGATGCAAGCGCATCACTAGTAGATGGACCTGCGTTGTATTAGCTAGTTGGTAA<br>GGTAAGAGCTTACCAAGGCGACGATCCGTAGCTGGTCTGAGAGGATGATCAGCCACACTGGGACTGAGACACGGCCCAGA<br>CTCCTACGGGAGGCAGCAGTGGGGAATATTGGACAATGGGGGAAACCCTGATCCAGCAATGCCGCGTGTGTGAAGAAGGC<br>CTTCGGGTTGTAAAGCACTTTCAGCAGGGAGGAAAGCATCATGGTTAATACCCATGGTAGTGACGTTACCTGCAGAAGAAG<br>CACCGGCTAACTCCGTGCCAGCAGCCGCGGTAATACGGAGGGTGCAAGCGTTATTCGGAATTACTGGGCGTAAAGCGCA<br>CGCAGGCGGCTATCTAAGTCAGATGTGAAAGCCCCGGGCTTAACCTGGGAACTGCATTTGAAACTGGGTGGCTAGAGTAT<br>GAAAGAGGAAAGCGGAATTTCCAGTGTAGCAGTGAAATGCGTAGATATTGGAAGGAACACCGATGGCGAAGGCAGCTTTCT<br>GGGTCGATACTGACGCTCATGTGCGAAAGCGTGGGGAGCAAACAGGATTAGATACCCTGGTAGTCCACGCCCTAAACGAT<br>GTCAACTAGGCGTCGGGTTGTATAGACTCGGTGCCGCAGCTAACGCATTAAGTTGACCGCCTGGGGAGTACGGCCGCAA<br>GGTTGAAACTCAAAGAAATTGACGGGGACCCGCACAAGCGGTGGAGCATGTGGTTTAATTCGATGCAACGCGAAGAACCTT<br>ACCAGGCCTTGACATCCAGAGAACTTAGCAGAGATGCTTTGGTGCCTTCGGGAACTCTGAGACAGGTGTTGCATGGCTGT<br>CGTCAGCTCGTGTCGTGAGATGTTGGGTTAAGTCCCGCAACGAGCGCAACCCTTATCCTTACTTGCCAGCACTTAGGGTG<br>GGAACTGTAAGGAGACTGCCGGTGACAAGCCGGAGGAAGGTGGGGATGACGTCAAGTCATCATGGCCCTTACGGCCTGG<br>GCTACACACGTGCTACAATGGTTGGTACAGACGGTAGCGAAGCCGCGAGGTGGAGCCAATCTGAGAAAGCCGATCGTAGT<br>CCGGATTGCAGTCTGCAACTCGACTGCATGAAGTCGGAATCGCTAGTAATCGCGAATCAGCATGTCGCGGTGAATACGTTC<br>CCGGGTCTTGTACACACTGCCCGTCACACCATGGGAGTTTGTTGCACCAGAAGCAGGTAGCTTAACGAAAGAGGGCGCTT<br>GCCACGGTGTGGCCGATGACTGGGGTG
194	Fusobacterium sp. OTU_1093	GATGAACGCTGACAGAATGCTTAACACATGCAAGTCTACTTGAATTTGGGTTTTTAACTTAGGTTTGGGTGGCGGACGGGTG<br>AGTAACGCGTAAAGAACTTGCCTCACAGCTAGGGACAACATTTAGAAATGAATGCTAATACCTAATATTATGATAATAGGGCAT<br>CCTATAATTATGAAAGCTATATGCGCTGTGAGAGAGCTTTGCGTCCCATTAGCTAGTTGGAGAGGTAACGGCTCACCAAGG<br>CGATGATGGGTAGCCGGCCTGAGAGGGTGATCGGCCACAAGGGGACTGAGACACGGCCCTTACTCCTACGGGAGGCAG<br>CAGTGGGGAATATTGGACAATGGACCGAGAGTCTGATCCAGCAATTCTGTGTGCACGATGAAGTTTTTCGGAATGTAAAGTG<br>CTTTCAGTTGGGAAGAAAGAAATGACGGTACCAACAGAAGAAGTGACGGCTAAATACGTGCCAGCAGCCGCGGTAATACGT<br>ATGTCACGAGCGTTATCCGGATTTATTGGGCGTAAAGCGCGTCTAGGTGGTTATATAAGTCTGATGTGAAAATGCAGGGCTC<br>AACTCTGTATTGCGTTGGAAACTGTATAACTAGAGTACTGGAGAGGTAAGCGGAACTACAAGTGTAGAGGTGAAATTCGTAGA<br>TATTTGTAGGAATGCCGATGGGGAAGCCAGCTTACTGGACAGATACTGACGCTGAAGCGCGAAAGCGTGGGTAGCAAACAG<br>GATTAGATACCCTGGTAGTCCACGCTGTAAACGATGATTACTAGGTGTTGGGGGTCGAACCTCAGCGCCCAAGCAAACGC<br>GATAAGTAATCCGCCTGGGGAGTACGTACGCAAGTATGAAACTCAAAGGAATTGACGGGGACCCGCACAAGCGGTGGAGC<br>ATGTGGTTTAATTCGACGCAACGCGAGGAACCTTACCAGCGTTTGACATCTTAGGAATGAGATAGAGATATTTCAGTGTCCC<br>TTCGGGGAAACCTAAAGACAGGTGGTGCATGGCTGTCGTCAGCTCGTGTCGTGAGATGTTGGGTTAAGTCCCGCAACGAG<br>CGCAACCCCTTTCGTATGTTACCATCATTAAGTTGGGGACTCATGCGATACTGCCTACGATGAGTAGGAGGAAGGTGGGGA<br>TGACGTCAAGTCATCATGCCCCTTATGTTTAGGGCTACACACGTGCTACAATGGTCATTACAGAGAGAAGCAAAACTGCGAA<br>GTCAAGCAAACCTCAAAAAGATGATCCAAGTTCGGATTGTAGGCTGAAACTCGCCTACATGAAGTTGGAGTTGCTAGTAATC<br>GCGAATCAGAATGTCGCGGTGAATGCGTTCCCGGGTCTTGTACACACCGCCCGTCACACCATGGGAGTTTGGGGGGCCC<br>AAAGTCAGCGACTCAACCGCAAGGAGAGAGCTGCCCAAGGCAAAACAAATGACTGGGGTG
195	Lautropia sp. OTU_1094	ATTGAACGCTGGCGGCATGCTTTACACATGCAAGTCGAACGGCAGCGCGGGCTTCGGCCTGGCGGCGAGTGGCGAACG<br>GGTGAGTAATACATCGGAACGTGCCCAGTAGTGGGGGATAGCTCGGCGAAAGCCGGATTAATACCGCATACGACCTGAGG<br>GTGAAAGCGGGGGATCTTCGGACCTCGCGCTATTGGAGCGGCCGATGGCAGATTAGCTAGTTGGTGGGGTAAAGGCCTA<br>CCAAGGCGACGATCTGTAGCTGGTCTGAGAGGACGACCAGCCACACTGGGACTGAGACACGGCCCAGACTCCTACGGGA<br>GGCAGCAGTGGGGAATTTTGGACAATGGGCGAAAGCCTGATCCAGCAATGCCGCGTGTGTGATGAAGGCCTTCGGGTTGT<br>AAAGCACTTTTGGCGGGAACGAAAAGGACTGTGCCAATACCACGGTTCGATGACGGTACCCGCAGAATAAGCACCGGCTAA<br>CTACGTGCCAGCAGCCGCGGTAATACGTAGGGTGCAAGCGTTAATCGGAATTACTGGGCGTAAAGAGTGCGCAGGCGGTT<br>TTGCAAGACCGATGTGAAATCCCCGGGCTTAACCTGGGAACTGCATTGGTGACTGCAAGGCTAGAGTGTGTCAGAGGGAG<br>GTGGAATTCCGCATGTAGCAGTGAAATGCGTAGATATGCGGAGGAACACCGATGGCGAAGGCAGCCTCCTGGGATAACAC<br>TGACGCTCATGCACGAAAGCGTGGGGAGCAAACAGGATTAGATACCCTGGTAGTCCACGCCCTAAACGATGTCTACTAGTT<br>GTCGGGGATTAATTTCCTTGGTAACGCAGCTAACGCGTGAAGTAGACCGCCTGGGGAGTACGGTCGCAAGATTAAAACTCA<br>AAGGAATTGACGGGGACCCGCACAAGCGGTGGATGATGTGGATTAATTCGATGCAACGCGAAGAACCTTACCTGGTCTTGA<br>CATGTACGGAACCTTCCAGAGACGGAAGGGTGCCTTCGGGAGCCGTAACACAGGTGCTGCATGGCTGTCGTCAGCTCGT<br>GTCGTGAGATGTTGGGTTAAGTCCCGCAACGAGCGCAACCCTTGTCATTAGTTGCCATCATTCAGTTGGGCACTCTAATGA<br>GACTGCCGGTGACAAACCGGAGGAAGGTGGGGATGACGTCAAGTCCTCATGGCCCTTATGACCAGGGCTTCACACGTCAT<br>ACAATGGTCGGTACAAAGGGTAGCCAAGCCGCGAGGCGGAGCCAATCTCATAAAACCGATCGTAGTCCGGATTGCACTCT<br>GCAACTCGAGTGCATGAAGTCGGAATCGCTAGTAATCGCAGGTCAGCATACTGCGGTGAATACGTTCCCGGGTCTTGTACA<br>CACCGCCCGTCACACCATGGGAGTGGGGGATACCAGAAGTAGGTAGGGTAACCGCAAGGAGCCCGCTTACCACGGTATG<br>CTTCATGACTGGGGTG
196	Capnocytophaga sp. OTU_1095	GATGAACGCTAGCGGCAGGCCTAACACATGCAAGTCGAGGGAGAGGTTACCTTCGGGTAACCAAAACCGGCGCACGGGT<br>GCGTAACGCGTATACAATCTGCCTTTCACATAGGGATAGCCCGAAGAAATTTGGATTAATACCTAATGGTATATAAGAGCGG<br>CATCGTTTTTATATTAAAGCTACGGTGGTGAAAGATGAGTATGCGTTCTATTAGCTAGTTGGAGAGGTAACGGCTCCCCAAG<br>GCGACGATAGATAGGGGTTCTGAGAGGGATGTCCCCCACACTGGTACTGAGATACGGACCAGACTCCTACGGGAGGCAG<br>CAGTGAGGAATATTGGACAATGGTCGGAAGACTGATCCAGCCATGCCGCGTGCAGGATGAAGGTCCTATGGATTGTAAACT<br>GCTTTTGTAAGGGAAGAATAAGGAGTACGTGTACTTTGATGACGGTACCTTACGAATAAGCATCGGCTAACTCCGTGCCAGC<br>AGCCGCGGTAATACGGAGGATGCGAGCGTTATCCGGAATCATTGGGTTTAAAGGGTCCGTAGGCGGGCTGATAAGTCAGA<br>GGTGAAAGCGCTTAGCTCAACTAAGCAACTGCCTTTGAAACTGTCAGTCTTGAATGATTGTGAAGTAGTTGGAATGTGTAGT<br>GTAGCGGTGAAATGCTTAGATATTACACAGAACACCGATAGCGAAGGCATATTACTAACAATTTATTGACGCTGATGGACGAA<br>AGCGTGGGGAGCGAACAGGATTAGATACCCTGGTAGTCCACGCTGTAAACGATGGATACTAGCTGTTTGGAGCAATCTGAG<br>TGGCTAAGCGAAAGTGATAAGTATCCCACCTGGGGAGTACGCACGCAAGTGTGAAACTCAAAGGAATTGACGGGGGCCCG<br>CACAAGCGGTGGAGCATGTGGTTTAATTCGATGATACGCGAGGAACCTTACCAAGGTTTAAATGGGGACTGACAGAGGTAG<br>AGATACCTCCTTCTTCGGACAGTTTTCAAGGTGCTGCATGGTTGTCGTCAGCTCGTGCCGTGAGGTGTCAGGTTAAGTCCT<br>ATAACGAGCGCAACCCCTATTGTTAGTTGCCATCATTCAGTTGGGCACTCTAGCGAGACTGCCGGTAATAAACCGGAGGAA<br>GGTGGGGATGACGTCAAATCATCATGCCCCTTATGACCTGGGCTACACACGTGCTACAATGGCTGGTACAACGAGTCGCA<br>AGCCGGTGACGGCAAGCTAATCTCTGAAAGCCAGTCTCAGTTCGGATTGTAGGCTGCAACTCGCCTACATGAAGTCGGAAT<br>CGCTAGTAATCGCGGATCAGCACGCCGCGGTGAATACGTTCCCGGGCCTTGTACACACCGCCCGTCACACCACGAGAGT<br>TTGTAACACCCGAAGTCGGTGAGGTAACCGTAAGGAGCCAGCCGCCTAAGGTGGGATAGATGATTGGGGTG

Sr no.	Species_OTUs	16S reference sequence
197	Gemella sp. OTU_1097	GACGAACGCTGGCGGCGTGCCTAATACATGCAAGTAGAACGCTGAAGCTTGGTGCTTGCACCGAGCGGATGAGTTGCGAACGGGTGAGTAACGCGTAGGTAACCTGCCTGGTAGCGGGGGATAACTATTGGAAACGATAGCTAATACCGCATAAGAGTAGATGTTGCATGACATTTGCTTAAAAGGTGCAATTGCATCACTACCAGATGGACCTGCGTTGTATTAGCTAGTTGGTGAGGTAACGGCTCACCAAGGCGACGATACATAGCCGACCTGAGAGGGTGATCGGCCACACTGGGACTGAGACACGGCCCAGACTCCTACGGGAGGCAGCAGTAGGGAATCTTCCGCAATGGGCGAAAGCCTGACGGAGCAACGCCGCGTGAGTGAAGAAGGATTTCGGTTCGTAAAGCTCTGTTGTTAGGGAAGAATGGATATGTAGTAACTATACATGTAAGAGACGGTACCTAACCAGAAAGCCACGGCTAACTACGTGCCAGCAGCCGCGGTAATACGTAGGTGGCAAGCGTTGTCCGGAATTATTGGGCGTAAAGCGCGCGCAGGTGGTTTAATAAGTCTGATGTGAAAGCCCACGGCTCAACCGTGGAGGGTCATTGGAAACTGTTAAACTTGAGTGCAGGAGAGAAAAGTGGAATTCCTAGTGTAGCGGTGAAATGCGTAGAGATTAGGAGGAACACCAGTGGCGAAGGCGGCTTTTTGGCCTGTAACTGACACTGAGGCGCGAAAGCGTGGGGAGCAAACAGGATTAGATACCCTGGTAGTCCACGCCGTAAACGATGAGTGCTAAGTGTTGGTCTCATAAGAGATCAGTGCTGCAGCTAACGCATTAAGCACTCCGCCTGGGGAGTACGACCGCAAGGTTGAAACTCAAAGGAATTGACGGGGACCCGCACAAGCGGTGGAGCATGTGGTTTAATTCGAAGCAACGCGAAGAACCTTACCAAGTCTTGACATACTGTGAGGACACAAGAGATTGTGTTGTTCTGACCTATGGTTAGACACAGATACAGGTGGTGCATGGTTGTCGTCAGCTCGTGTCGTGAGATGTTGGGTTAAGTCCCGCAACGAGCGCAACCCTTATATCTAGTTGCCAGCAGTAAGATGGGGACTCTAGATAGACTGCCAGTGATAAACTGGAGGAAGGTGGGGATGACGTCAAATCATCATGCCCCTTATGACTTGGGCTACACACGTGCTACAATGGATAGGAACAAAGAGAAGCGAGCTCGCGAGAGTCAGCCAACCTCATAAAACTATTCTCAGTTCGGATTGTAGTCTGCAACTCGACTACATGAAGCTGGAATCGCTAGTAATCGCGAATCAGAATGTCGCGGTGAATACGTTCCCGGGTCTTGTACACACCGCCCGTCACACCACGAGAGTTTGTAACACCCGAAGACGGTGGCCTAACCTTTAAGGAGGGAGCCGGTCACGGTGGGACAGATGATTGGGGTG
198	Peptostreptococcus sp. OTU_1098	GATGAACGCTGACAGAATGCTTAACACATGCAAGTCTACTTGAATTTGGGTTTTTAACTTAGGTTTGGGTGGCGGACGGGTGAGTAACGCGTAAAGAACTTGCCTCACAGCTAGGGACAACATTTAGAAATGAATGCTAATACCTAATATTATGATAATAGGGCATCCTATAATTATGAAAGCTATATGCGCTGTGAGAGAGCTTTGCGTCCCATTAGCTAGTTGGAGAGGTAACGGCTCACCAAGGCGATGATGGGTAGCCGGCCTGAGAGGGTGATCGGCCACAAGGGGACTGAGACACGGCCCTTACTCCTACGGGAGGCAGCAGTGGGGAATATTGCACAATGGGCGAAAGCCTGATGCAGCAACGCCGCGTGAACGATGAAGGTCTTCGGATCGTAAAGTTCTGTTGCAGGGGAAGATAATGACGGTACCCTGTGAGGAAGCCCCGGCTAACTACGTGCCAGCAGCCGCGGTAATACGTAGGGGGCTAGCGTTATCCGGATTTACTGGGCGTAAAGGGTGCGTAGGTGGTCCTTCAAGTCGGTGGTTAAAGGCTACGGCTCAACCGTAGTAAGCCGCCGAAACTGGAGGACTTGAGTGCAGGAGAGGAAAGTGGAATTCCCAGTGTAGCGGTGAAATGCGTAGATATTGGGAGGAACACCAGTAGCGAAGGCGGCTTTCTGGACTGCAACTGACACTGAGGCACGAAAGCGTGGGTAGCAAACAGGATTAGATACCCTGGTAGTCCACGCTGTAAACGATGAGTACTAGGTGTCGGGGGTTACCCCCCTCGGTGCCGCAGCTAACGCATTAAGTACTCCGCCTGGGGAGTACGCACGCAAGTGTGAAACTCAAAGGAATTGACGGGGACCCGCACAAGTAGCGGAGCATGTGGTTTAATTCGAAGCAACGCGAAGAACCTTACCTAAGCTTGACATCCCTCGGACAGGTGTTTAATCACACCCTTCCTTCGGGACTGAGGTGACAGGTGGTGCATGGTTGTCGTCAGCTCGTGTCGTGAGATGTTGGGTTAAGTCCCGCAACGAGCGCAACCCTTGTCTTTAGTTGCCAGCATTCAGTTGGGCACTCTAGAGAGACTGCCAGGGATAACCTGGAGGAAGGTGGGGATGACGTCAAATCATCATGCCCCTTATGCTTAGGGCTACACACGTGCTACAATGGGTGGTACAGAGGGTTGCCAAACCGTGAGGTGGAGCCAATCCCTTAAAGCCATTCTCAGTTCGGATTGTAGGCTGAAACTCGCCTACATGAAGCTGGAGTTACTAGTAATCGCAGATCAGAATGCTGCGGTGAATGCGTTCCCGGGTCTTGTACACACCGCCCGTCACACCACGGGAGTCGGAAACACCCGAAGCCGATTATCCAACCGCAAGGAGGAAGTCGTCGAAGGTGGCGTCGATAACTGGGGTG
199	Cardiobacterium sp. OTU_1099	ATTGAACGCTGGCGGCATGCTTAACACATGCAAGTCGAACGGAAACGACGGAGCTTGCTCCGGGCGTCGAGTGGCGGACGGGTGAGTAATGCATGGGAATCTGCCTTTTGCTGGGGGATAACGTAGGGAAACTTACGCTAATACCGCATAAGACCTGAGGGTGAAAGTGGGGGACCGTAAGGCCTCACGGCAGAAGATGAGCCCATGTCGGATTAGCTAGTTGGTGGGGTAAAGGCCTACCAAGGCGACGATCCGTAGCTGGTCTGAGAGGATGATCAGCCACACTGGGACTGAGACACGGCCCAGACTCCTACGGGAGGCAGCAGTGGGGAATATTGGACAATGGGGGAAACCCTGATCCAGCAATGCCGCGTGTGTGAAGAAGGCCTTCGGGTTGTAAAGCACTTTCAGCAGGGAGGAAAGCATCATGGTTAATACCCATGGTAGTGACGTTACCTGCAGAAGAAGCACCGGCTAACTCCGTGCCAGCAGCCGCGGTAATACGGAGGGTGCAAGCGTTATTCGGAATTACTGGGCGTAAAGCGCACGCAGGCGGCTATCTAAGTCAGATGTGAAAGCCCCGGGCTTAACCTGGGAACTGCATTTGAAACTGGGTGGCTAGAGTATGAAAGAGGAAAGCGGAATTTCCAGTGTAGCAGTGAAATGCGTAGATATATGGAGGAACACCAGTGGCGAAAGCGGCTCTCTGGTCTGTCACTGACGCTGAGGCTCGAAAGCGTGGGTAGCGAACAGGATTAGATACCCTGGTAGTCCACGCCGTAAACGATGAGTGCTAGGTGTTAGGCCCTTTCCGGGGCTTAGTGCCGGAGCTAACGCAATAAGCACTCCGCCTGGGGAGTACGACCGCAAGGTTGAAACTCAAAGGAATTGACGGGGGCCCGCACAAGCGGTGGAGCATGTGGTTTAATTCGAAGCAACGCGAAGAACCTTACCAGGTCTTGACATCCCGATGCTATTCTTAGAGATAGGAAGTTACTTCGGTACATCGGAGACAGGTGGTGCATGGTTGTCGTCAGCTCGTGTCGTGAGATGTTGGGTTAAGTCCCGCAACGAGCGCAACCCTTATTGTTAGTTGCCATCATTAAGTTGGGCACTCTAGCGAGACTGCCGGTAATAAACCGGAGGAAGGTGGGGATGACGTCAAATCATCATGCCCCTTATGACCTGGGCTACACACGTGCTACAATGGTCGGTACAACGAGTTGCGAGCCGGTGACGGCAAGCTAATCTCTGAAAGCCGATCTCAGTTCGGATTGGAGGCTGCAACTCGCCTCCATGAAGTCGGAATCGCTAGTAATCGCGGATCAGCACGCCGCGGTGAATACGTTCCCGGGCCTTGTACACACCGCCCGTCACACCACGAGAGTTTGTAACACCCGAAGTCGGTGAGGTAACCTTTTAGGGGCCAGCCGCCTAAGGTGGGATGGATGATTGGGGTG
200	Porphyromonas sp. OTU_1101	GATGAACGCTGGCTACAGGCTTAACACATGCAAGTCGTGGGGAAACGGCATTATGTGCTTGCACATTCTGGACGTCGACCGGCGCACGGGTGAGTATCGCGTATCCAACCTGCCCCTTACTTGGGGATACCCCGTTGAAAGACGGCCTAATACCCGATGTGTTTCATTGACGGCATCCGATATGAAACAAAGGTTTTCCGGTAAGGGATGGGGATGCGTCTGATTAGCTTGTTGGCGGGGCAACGGCCCACCAAGGCGACGATCAGTAGGGGTTCTGAGAGGAAGGTCCCCCACATTGGAACTGAGACACGGTCCAAACTCCTACGGGAGGCAGCAGTGAGGAATATTGGTCAATGGGCGAGAGCCTGAACCAGCCAAGTCGCGTGAAGGACGACGGTTCTATGGATTGTAAACTTCTTTTGTAGAGGAATAATGGCAGCTACGTGTAGCTGAGATGCATGTACTCTACGAATAAGTATCGGCTAACTCCGTGCCAGCAGCCGCGGTAATACGGAGGATACGAGCGTTATCCGGAATTATTGGGTTTAAAGGGTGCGTAGGTGGCGTATTAAGTCAGTGGTGAAAAGCTGCAGCTCAACTGTAGTCTTGCCGTTGAAACTGATATGCTAGAGAGGAGACGAGGTATGCGGAATGTGTGGTGTAGCGGTGAAATGCATAGATATCACACAGAACGCCGATTGCGAAGGCAGCGTACCAGGCTCCGTCTGACACTGAAGCACGAAAGCGTGGGGATCAAACAGGATTAGATACCCTGGTAGTCCACGCAGTAAACGATGAATACTAGATTTTTGCGATATAGTGTAAGAGTCTAAGCGAAAGCGATAAGTATTCCACCTGGGGAGTACGCCGGCAACGGTGAAACTCAAAGGAATTGACGGGGGCCCGCACAAGCGGAGGAACATGTGGTTTAATTCGATGATACGCGAGGAACCTTACCCGGGATTGAAATTTAGATGGTGGCAGATGAGAGTTTGCTTTCCTTCGGGACATCTAAGTAGGTGCTGCATGGTTGTCGTCAGCTCGTGCCGTGAGGTGTCGGCTTAAGTGCCATAACGAGCGCAACCCGCGTCGATAGTTACTAACAGGTCATGCTGAGGACTCTATCGAGACAGCCGTCGTAAGACGCGAGGAAGGGGCGGATGACGTCAAATCAGCACGGCCCTTACATCCGGGGCGACACACGTGTTACAATGGCAGGGACAAAGGGAAGCGACATGGTGACATGAAGCGGATCTCCAAACCCTGTCCCAGTTCGGATCGGAGTCTGCAACTCGACTCCGTGAAGCTGGATTCGCTAGTAATCGCGCATCAGCCATGGCGCGGTGAATACGTTCCCGGGCCTTGTACACACCGCCCGTCAAGCCATGGAAGTTGGGAGTACCTGAAGAGCGTAACCGGAAGGGGCGCTTAAGGGTAATACCAGTGACTGGGGCT

Sr no.	Species_OTUs	16S reference sequence
201	Fusobacterium sp. OTU_1122	GATGAACGCTGACAGAATGCTTAACACATGCAAGTCAACTTGAATTTGGGTTTTTAACTTAGGTTTGGGTGGCGGACGGGTG<br>AGTAACGCGTAAAGAACTTGCCTCACAGTTAGGGACAACATTTGGAAACGAATGCTAATACCTGATATTATGATAATAGGGCA<br>TCCCATAATTATGAAAGCTATAAGCGCTGTGAGAGAGCTTTGCGTCCCATTAGCTAGTTGGAGAGGTAACGGCTCACCAAG<br>GCGATGATGGGTAGCCGGCCTGAGAGGGTGATCGGCCACAAGGGGACTGAGACACGGCCCTTACTCCTACGGGAGGCA<br>GCAGTGGGGAATATTGGACAATGGACCAAGAGTCTGATCCAGCAATTCTGTGTGCACGATGAAGTTTTTCGGAATGTAAAGT<br>GCTTTCAGTTGGGAAGAAATAAATGACGGTACCAACAGAAGAAGTGACGGCTAAATACGTGCCAGCAGCCGCGGTAATACG<br>TATGTCACGAGCGTTATCCGGATTTATTGGGCGTAAAGCGCGTCTAGGTGGTTATGTAAGTCTGATGTGAAAATGCAGGGCT<br>CAACTCTGTATTGCGTTGGAAACTGTATAACTAGAGTACTGGAGAGGTAAGCGGAACTACAAGTGTAGAGGTGAAATTCGTA<br>GATATTTGTAGGAATGCCGATGGGGAAGCCAGCTTACTGGACAGATACTGACGCTGAAGCGCGAAAGCGTGGGTAGCAAAC<br>AGGATTAGATACCCTGGTAGTCCACGCCGTAAACGATGATTACTAGGTGTTGGGGGTCGAACCTCAGCGCCCAAGCAAAC<br>GCGATAAGTAATCCGCCTGGGGAGTACGTACGCAAGTATGAAACTCAAAGGAATTGACGGGGACCCGCACAAGCGGTGGA<br>GCATGTGGTTTAATTCGATGCAACGCGAAGAACCTTACCTACTCTTGACATCCAGAGAACATTCCAGAGATGGATTGGTGCC<br>TTCGGGAACTCTGAGACAGGTGCTGCATGGCTGTCGTCAGCTCGTGTTGTGAAATGTTGGGTTAAGTCCCGCAACGAGCG<br>CAACCCTTATCCTTTGTTGCCAGCGATTAGGTCGGGAACTCAAAGGAGACTGCCGGTGATAAACCGGAGGAAGGTGGGGA<br>TGACGTCAAGTCATCATGGCCCTTACGAGTAGGGCTACACACGTGCTACAATGGCGTATACAGAGGGAAGCGAGAGTGCG<br>AGCTGGAGCGAATCTCACAAAGTACGTCTAAGTCCGGATTGGAGTCTGCAACTCGACTCCATGAAGTCGGAATCGCTAGTA<br>ATCGCAAATCAGAATGTTGCGGTGAATACGTTCCCGGGCCTTGTACACACCGCCCGTCACACCATGGGAGTGGGTTGTAC<br>CAGAAGTAGATAGCTTAACCTTCGGGGGGGCGTTTACCACGGTATGATTCATGACTGGGGTG
202	Abiotrophia sp. OTU_1127	GACGAACGCTGGCGGCGTGCCTAATACATGCAAGTAGAACGCTGAAGAGAGGAGCTTGCTCTTCTTGGATGAGTTGCGAA<br>CGGGTGAGTAACGCGTAGGTAACCTGCCTGGTAGCGGGGGATAACTATTGGAAACGATAGCTAATACCGCATAAAATTGATT<br>ATCGCATGATAATTAATTGAAAGATGCAATTGCATCACTACCAGATGGACCTGCGTTGTATTAGCTAGTTGGTGAGGTAACGG<br>CTCACCAAGGCGACGATACATAGCCGACCTGAGAGGGTGATCGGCCACATTGGGACTGAGACACGGCCCAAACTCCTAC<br>GGGAGGCAGCAGTAGGGAATCTTCCGCAATGGACGCAAGTCTGACGGAGCAACGCCGCGTGAGTGAAGAAGGTCTTCGG<br>ATCGTAAAGCTCTGTTGTTAGAGAAGAACAGCGCATAGAGTAACTGCTATGCGTGTGACGGTATCTAACCAGAAAGCCACGG<br>CTAACTACGTGCCAGCAGCCGCGGTAATACGTAGGTGGCGAGCGTTGTCCGGATTTATTGGGCGTAAAGGGAGTGTAGGC<br>GGTCTTTTAAGTCTGATGTGAAAGCCCACGGCTCAACCGTGGAGGGTCATTGGAAACTGGGAGACTTGAGTGCAGAAGAGG<br>AGAGCGGAATTCCATGTGTAGCGGTGAAATGCGTAGATATATGGAGGAACACCAGTGGCGAAGGCGGCTCTCTGGTCTGTA<br>ACTGACGCTGAGGCTCGAAAGCGTGGGGAGCAAACAGGATTAGATACCCTGGTAGTCCACGCCGTAAACGATGAGTGCTA<br>AGTGTTGGAGGGGTTCCACCCTTCAGTGCTGGAGTTAACGCAATAAGCACTCCGCCTGGGGAGTACGGCCGCAAGGCTG<br>AAACTCAAAGGAATTGACGGGGACCCGCACAAGCGGTGGAGCATGTGGTTTAATTCGAAGCAACGCGAAGAACCTTACCAG<br>GTCTTGACATCCCGACGACCGCTCTAGAGATAGAGTTTTTCTTCGGAACGTCGGTGACAGGTGGTGCATGGTTGTCGTCA<br>GCTCGTGTCGTGAGATGTTGGGTTAAGTCCCGCAACGAGCGCAACCCCTATAACTAGTTGCCAGCATTGAGATGGGGACT<br>CTAGTTAGACTGCCGGTGACAAACCGGAGGAAGGTGGGGATGACGTCAAATCATCATGCCCCTTATGACCTGGGCTACAC<br>ACGTGCTACAATGGATGGTACAACGAGCAGCGAACTCGCGAGGGTAAGCGAATCTCTAAAAGCCATTCTCAGTTCGGATTG<br>TAGTCTGCAACTCGACTACATGAAGCCGGAATCGCTAGTAATCGCGGATCAGCACGCCGCGGTGAATACGTTCCCGGGTC<br>TTGTACACACCGCCCGTCACACCACGAGAGTTTGTAACACCCGAAGCCGGTGGCCTAACCTTTTAGGAGGGAGCCGTCGA<br>AGGTGGGATAGATGATTGGGGTG
203	Neisseria sp. OTU_1132	ATTGAACGCTGGCGGCATGCTTTACACATGCAAGTCGGACGGCAGCACAGAGAAGCTTGCTTCTTGGGTGGCGAGTGGCG<br>AACGGGTGAGTAATATATCGGAACGTACCGAGTAATGGGGGATAACTAATCGAAAGATTAGCTAATACCGCATATTCTCTGAG<br>GAGGAAAGCAGGGGACCTTCGGGCCTTGCGTTATTCGAGCGGCCGATATCTGATTAGCTAGTTGGTGGGGTAAAGGCCTA<br>CCAAGGCGACGATCAGTAGCGGGTCTGAGAGGATGATCCGCCACACTGGGACTGAGACACGGCCCAGACTCCTACGGGA<br>GGCAGCAGTGGGGAATTTTGGACAATGGGCGCAAGCCTGATCCAGCCATGCCGCGTGTCTGAAGAAGGCCTTCGGGTTG<br>TAAAGGACTTTTGTCAGGGAAGAAAAGGCTGTTGCTAATATCGACAGCTGATGACGGTACCTGAAGAATAAGCACCGGCTAA<br>CTACGTGCCAGCAGCCGCGGTAATACGTAGGGTGCGAGCGTTAATCGGAATTACTGGGCGTAAAGCGAGCGCAGACGGTT<br>ACTTAAGCAGGATGTGAAATCCCCGGGCTCAACCTGGGAACTGCGTTCTGAACTGGGTGACTAGAGTGTGTCAGAGGGAG<br>GTAGAATTCCACGTGTAGCAGTGAAATGCGTAGAGATGTGGAGGAATACCGATGGCGAAGGCAGCCTCCTGGGATAACACT<br>GACGTTCATGCTCGAAAGCGTGGGTAGCAAACAGGATTAGATACCCTGGTAGTCCACGCCCTAAACGATGTCAATTAGCTG<br>TTGGGCAACTTGATTGCTTAGTAGCGTAGCTAACGCGTGAAATTGACCGCCTGGGGAGTACGGTCGCAAGATTAAAACTCA<br>AAGGAATTGACGGGGACCCGCACAAGCGGTGGATGATGTGGATTAATTCGATGCAACGCGAAGAACCTTACCTGGTCTTGA<br>CATGTACGGAATCCTCCAGAGACGGAGGAGTGCCTTCGGGAGCCGTAACACAGGTGCTGCATGGCTGTCGTCAGCTCGT<br>GTCGTGAGATGTTGGGTTAAGTCCCGCAACGAGCGCAACCCTTGTCATTAGTTGCCATCATTTAGTTGGGCACTCTAATGA<br>GACTGCCGGTGACAAGCCGGAGGAAGGTGGGGATGACGTCAAGTCCTCATGGCCCTTATGACCAGGGCTTCACACGTCA<br>TACAATGGTCGGTACAGAGGGTAGCCAAGCCGCGAGGTGGAGCCAATCTCACAAAACCGATCGTAGTCCGGATTGCACTC<br>TGCAACTCGAGTGCATGAAGTCGGAATCGCTAGTAATCGCAGGTCAGCATACTGCGGTGAATACGTTCCCGGGCCTTGTA<br>CACACCGCCCGTCACACCACGAGAGTTTGTAACACCCGAAGTCGGTGAGGTAACCTTTTAGGAGCCAGCCGCCTAAGGTG<br>GGATAGATGATTGGGGTG
204	Streptococcus sp. OTU_1133	GACGAACGCTGGCGGCGTGCCTAATACATGCAAGTAGAACGCTGAAGGAGGAGCTTGCTTCTCTGGATGAGTTGCGAACG<br>GGTGAGTAACGCGTAGGTAACCTGCCTGGTAGCGGGGGATAACTATTGGAAACGATAGCTAATACCGCATAATAGTAGATGT<br>TGCATGACATTTGCTTAAAAGGTGCAATTGCATCACTACCAGATGGACCTGCGTTGTATTAGCTAGTTGGTGAGGTAACGGC<br>TCACCAAGGCGACGATACATAGCCGACCTGAGAGGGTGATCGGCCACACTGGGACTGAGACACGGCCCAGACTCCTACG<br>GGAGGCAGCAGTAGGGAATCTTCGGCAATGGACGGAAGTCTGACCGAGCAACGCCGCGTGAGTGAAGAAGGTTTTCGGAT<br>CGTAAAGCTCTGTTGTAAGAGAAGAACGAGTGTGAGAGTGGAAAGTTCACACTGTGACGGTATCTTACCAGAAAGGGACGG<br>CTAACTACGTGCCAGCAGCCGCGGTAATACGTAGGTCCCGAGCGTTGTCCGGATTTATTGGGCGTAAAGCGAGCGCAGG<br>CGGTTAGATAAGTCTGAAGTTAAAGGCTGTGGCTTAACCATAGTACGCTTTGGAAACTGTTTAACTTGAGTGCAAGAGGGGA<br>GAGTGGAATTCCATGTGTAGCGGTGAAATGCGTAGATATATGGAGGAACACCGGTGGCGAAAGCGGCTCTCTGGCTTGTAA<br>CTGACGCTGAGGCTCGAAAGCGTGGGGAGCAAACAGGATTAGATACCCTGGTAGTCCACGCCGTAAACGATGAGTGCTAG<br>GTGTTAGACCCTTTCCGGGGTTTAGTGCCGCAGCTAACGCATTAAGCACTCCGCCTGGGGAGTACGACCGCAAGGTTGAA<br>ACTCAAAGGAATTGACGGGGGCCCGCACAAGCGGTGGAGCATGTGGTTTAATTCGAAGCAACGCGAAGAACCTTACCAGG<br>TCTTGACATCCCTCTGACCGCTCTAGAGATAGAGTTTTCCTTCGGGACAGAGGTGACAGGTGGTGCATGGTTGTCGTCAG<br>CTCGTGTCGTGAGATGTTGGGTTAAGTCCCGCAACGAGCGCAACCCCTATTGTTAGTTGCCATCATTCAGTTGGGCACTCT<br>AGCGAGACTGCCGGTAATAAACCGGAGGAAGGTGGGGATGACGTCAAATCATCATGCCCCTTATGACCTGGGCTACACAC<br>GTGCTACAATGGCTGGTACAACGAGTCGCAAGCCGGTGACGGCAAGCTAATCTCTTAAAGCCAGTCTCAGTTCGGATTGTA<br>GGCTGCAACTCGCCTACATGAAGTCGGAATCGCTAGTAATCGCGGATCAGCACGCCGCGGTGAATACGTTCCCGGGCCT<br>TGTACACACCGCCCGTCACACCATGGGAGTGGGGGATACCAGAAGTAGGTAGGGTAACCGCAAGGAGCCCGCTTACCAC<br>GGTATGCTTCATGACTGGGGTG

Sr no.	Species_OTUs	16S reference sequence
205	Gemella sp. OTU_1135	GACGAACGCTGGCGGCGTGCCTAATACATGCAAGTCGAGCGAAGTTTTTCTGGTGCTTGCACTAGAAAAACTTAGCGGCGA<br>ACGGGTGAGTAACACGTAAAGAACCTGCCTCATAGACTGGGACAACTATTGGAAACGATAGCTAATACCGGATAACAGTATT<br>TCTCGCATGAGAGATATTTAAAAGTTGGTTATGCTAACACTATGAGATGGCTTTGCGGTGCATTAGCTAGTTGGTGGGGTAAA<br>GGCCCACCAAGGCGACGATGCATAGCCGACCTGAGAGGGTGATCGGCCACACTGGGACTGAGACACGGCCCAGACTCC<br>TACGGGAGGCAGCAGTAGGGAATCTTCCGCAATGGGCGAAAGCCTGACGGAGCAACGCCGCGTGAGTGAAGAAGGATTTC<br>GGTTCGTAAAGCTCTGTTGTTAGGGAAGAATGGATATGTAGTAACTATACATGTAAGAGACGGTACCTAACCAGAAAGCCAC<br>GGCTAACTACGTGCCAGCAGCCGCGGTAATACGTAGGTGGCAAGCGTTGTCCGGAATTATTGGGCGTAAAGCGCGCGCA<br>GGTGGTTTAATAAGTCTGATGTGAAAGCCCACGGCTCAACCGTGGAGGGTCATTGGAAACTGTTAAACTTGAGTGCAGGAG<br>AGAAAAGTGGAATTCCTAGTGTAGCGGTGAAATGCGTAGAGATTAGGAGGAACACCAGTGGCGAAGGCGGCTTTTTGGCCT<br>GTAACTGACACTGAGGCGCGAAAGCGTGGGGAGCAAACAGGATTAGATACCCTGGTAGTCCACGCCGTAAACGATGAGTG<br>CTAAGTGTTGGTCTCATAAGAGATCAGTGCTGCAGCTAACGCATTAAGCACTCCGCCTGGGGAGTACGACCGCAAGGTTGA<br>AACTCAAAGGAATTGACGGGGACCCGCACAAGCGGTGGAGCATGTGGTTTAATTCGAAGCAACGCGAAGAACCTTACCAAG<br>TCTTGACATACTGTGAGGACACAAGAGATTGTGTTGTTCTGACCTATGGTTAGACACAGATACAGGTGGTGCATGGTTGTCG<br>TCAGCTCGTGTCGTGAGATGTTGGGTTAAGTCCCGCAACGAGCGCAACCCTTATATCTAGTTGCCAGCAGTAAGATGGGGA<br>CTCTAGATAGACTGCCAGTGATAAACTGGAGGAAGGTGGGGATGACGTCAAATCATCATGCCCCTTATGACCTGGGCTACA<br>CACGTACTACAATGGGAGTTAATAGACGGAAGCGAGATCGCGAGATGGAGCAAACCCGAGAAACACTCTCTCAGTTCGGAT<br>CGTAGGCTGCAACTCGCCTACGTGAAGTCGGAATCGCTAGTAATCGCAGGTCAGCATACTGCGGTGAATACGTTCCCGGG<br>CCTTGTACACACCGCCCGTCACACCACGAAAGTCGGAAGTGCCCAAAGCCGGTGGGGTAACCTTCGGGAGCCAGCCGTC<br>TAAGGTAAAGTCGATGATTGGGGTG
206	Porphyromonas sp. OTU_1136	GATGAACGCTAGCGATAGGCTTAACACATGCAAGTCGAGGGGCAGCATGATCTTAGCTTGCTAAGGTCGATGGCGACCGG<br>CGCACGGGTGCGTAACGCGTATGCAACTTGCCTTACAGAGGGGGATAACCCGTTGAAAGACGGACTAAAACCGCATACAC<br>TTGTATTATTGCATGATATTACAAGGAAATATTTATAGCTGTAAGATAGGCATGCGTCCCATTAGCTGGTTGGTGAGGTAACG<br>GCTCACCAAGGCGACGATGGGTAGGGGAACTGAGAGGTTTATCCCCCACACTGGTACTGAGACACGGACCAGACTCCTAC<br>GGGAGGCAGCAGTGAGGAATATTGGTCAATGGGCGAGAGCCTGAACCAGCCAAGTCGCGTGAAGGAAGACTGTCCTAAGG<br>ATTGTAAACTTCTTTTATACGGGAATAACGGGCGATACGAGTATTGCATTGAATGTACCGTAAGAATAAGCATCGGCTAACTC<br>CGTGCCAGCAGCCGCGGTAATACGGAGGATGCGAGCGTTATCCGGATTTATTGGGTTTAAAGGGTGCGTAGGTTGTTCGG<br>TAAGTCAGCGGTGAAACCTGAGCGCTCAACGTTCAGCCTGCCGTTGAAACTGCCGGGCTTGAGTTCAGTGGCGGCAGGC<br>GGAATTCGTGGTGTAGCGGTGAAATGCATAGATATCACGAGGAACTCCGATTGCGAAGGCAGCTTGCCATACTGCGACTGA<br>CACTGAAGCACGAAGGCGTGGGTATCAAACAGGATTAGATACCCTGGTAGTCCACGCAGTAAACGATGATTACTAGGAGTTT<br>GCGATATACCGTCAAGCTTCCACAGCGAAAGCGTTAAGTAATCCACCTGGGGAGTACGCCGGCAACGGTGAAACTCAAAG<br>GAATTGACGGGGGCCCGCACAAGCGGAGGAACATGTGGTTTAATTCGATGATACGCGAGGAACCTTACCCGGGATTGAAAT<br>GTAGATGACGGATGGTGAAAACCGTCTTCCCTTCGGGGCTTCTATGTAGGTGCTGCATGGTTGTCGTCAGCTCGTGCCGT<br>GAGGTGTCGGCTTAAGTGCCATAACGAGCGCAACCCACATCGGTAGTTGCTAACAGTTTTCGCTGAGGACTCTACCGAGA<br>CTGCCGTCGTAAGGCGTGAGGAAGGTGTGGATGACGTCAAATCAGCACGGCCCTTACATCCGGGGCGACACACGTGTTA<br>CAATGGGAGGGACAAAGGGCAGCTACCGGGCGACCGGATGCGAATCTCTAAACCCTTCCCCAGTTCGGATCGGAGTCTG<br>CAACTCGAGTACATGAAGTTGGAATCGCTAGTAATCGCGAATCAGCAATGTCGCGGTGAATACGTTCTCGGGTCTTGTACA<br>CACCGCCCGTCACACCACGAGAGTTGGTTGCACCTGAAGTAGCAGGCCTAACCGTAAGGAGGGATGCTCCGAGGGTGTG<br>ATTAGCGATTGGGGTG
207	Streptococcus sp. OTU_1137	GACGAACGCTGGCGGCGTGCCTAATACATGCAAGTAGAACGCACAGGATGCACCGTAGTTTACTACACCGTATTCTGTGAG<br>TTGCGAACGGGTGAGTAACGCGTAGGTAACCTGCCTGGTAGCGGGGGATAACTATTGGAAACGATAGCTAATACCGCATAA<br>GAACATTTACTGCATGGTAGATGTTTAAAAGGTGCAAATGCATCACTACCAGATGGACCTGCGTTGTATTAGCTAGTAGGTGA<br>GGTAACGGCTCACCTAGGCGACGATACATAGCCGACCTGAGAGGGTGATCGGCCACACTGGGACTGAGACACGGCCCAG<br>ACTCCTACGGGAGGCAGCAGTAGGGAATCTTCGGCAATGGGGGGAACCCTGACCGAGCAACGCCGCGTGAGTGAAGAAG<br>GTTTTCGGATCGTAAAGCTCTGTTGTTAAGGAAGAACGAGTGTGAGAATGGAAAGTTCATACTGTGACGGTACTTAACCAGAA<br>AGGGACGGCTAACTACGTGCCAGCAGCCGCGGTAATACGTAGGTCCCGAGCGTTGTCCGGATTTATTGGGCGTAAAGCGA<br>GCGCAGGCGGTTAGATAAGTCTGAAGTTAAAGGCAGTGGCTCAACCATTGTAGGCTTTGGAAACTGTTTAACTTGAGTGCAG<br>AAGGGGAGAGTGGAATTCCATGTGTAGCGGTGAAATGCGTAGATATATGGAGGAACACCGGTGGCGAAAGCGGCTCTCTG<br>GTCTGTAACTGACGCTGAGGCTCGAAAGCGTGGGGAGCGAACAGGATTAGATACCCTGGTAGTCCACGCCGTAAACGATG<br>AGTGCTAGGTGTTAGGTCCTTTCCGGGACTTAGTGCCGCAGCTAACGCATTAAGCACTCCGCCTGGGGAGTACGACCGCA<br>AGGTTGAAACTCAAAGGAATTGACGGGGGCCCGCACAAGCGGTGGAGCATGTGGTTTAATTCGAAGCAACGCGAAGAACCT<br>TACCAGGTCTTGACATCCCGATGCCCGCTCTAGAGATAGAGCTTTACTTCGGTACATCGGTGACAGGTGGTGCATGGTTGT<br>CGTCAGCTCGTGTCGTGAGATGTTGGGTTAAGTCCCGCAACGAGCGCAACCCTTATTGTTAGTTGCCATCATTCAGTTGGG<br>CACTCTAGCGAGACTGCCGGTAATAAACCGGAGGAAGGTGGGGATGACGTCAAATCATCATGCCCCTTATGTCTTGGGCTT<br>CACGCATGCTACAATGGCCGGTACAGAGGGCTGCGATACCGTGAGGTGGAGCGAATCCCTTAAAGCCGGTCTCAGTTCG<br>GATCGGTGTCTGCAACTCGACACCGTGAAGTTGGAGTCGCTAGTAATCGCAGATCAGCAACGCTGCGGTGAATACGTTCT<br>CGGGCCTTGTACACACCGCCCGTCACGTCATGAAAGTCGGCAACACCCGAAGCCCGTGGCCTTATGGGGAGCGGTCGAA<br>GGTGGGGCTGGTGATTGGGACG
208	Prevotella sp. OTU_1139	GATGAACGCTGGCTACAGGCTTAACACATGCAAGTCGAGGGGAAACGACGGGGAAGCTTGCTTCCCCGGGCGTCGACCG<br>GCGCACGGGTGAGTAACGCGTATCCAACCTGCCTCTGACTGAGGGATAACCCGTCGAAAGTCGGCCTAATACCTCATGGC<br>ATCGTCTGCGGGCATCCAACGACGATTAAAGATTTCATCGGTCAGGGATGGGGATGCGTCTGATTAGCTTGTTGGCGGGG<br>TAACGGCCCACCAAGGCGACGATCAGTAGGGGTTCTGAGAGGAAGGTCCCCCACATTGGAACTGAGACACGGTCCAAACT<br>CCTACGGGAGGCAGCAGTGAGGAATATTGGTCAATGGGCGAGAGCCTGAACCAGCCAAGTAGCGTGCAGGAAGACGGCC<br>CTATGGGTTGTAAACTGCTTTTATGCGGGGATAAAGTGAGGGACGTGTCCTTCATTGCAGGTACCGCATGAATAAGGACCG<br>GCTAATTCCGTGCCAGCAGCCGCGGTAATACGGAAGGTCCGGGCGTTATCCGGATTTATTGGGTTTAAAGGGAGCGTAGG<br>CCGTGGATTAAGCGTGTTGTGAAATGCAGGTGCTCAACGTCTGCACTGCAGCGCGAACTGGTCCACTTGAGTGTGCGCAA<br>CGCAGGCGGAATTCGTCGTGTAGCGGTGAAATGCTTAGATATGACGAAGAACTCCGATTGCGAAGGCAGCTTGCGGGAGC<br>ACAACTGACGCTGAAGCTCGAAAGTGCGGGTATCGAACAGGATTAGATACCCTGGTAGTCCGCACGGTAAACGATGGATGC<br>CCGTTGTCAGGCTGTTTCAGCCTGGTGACCAAGCGAAAGCATTAAGCATCCCACCTGGGGAGTACGCCGGCAACGGTGAA<br>ACTCAAAGGAATTGACGGGGGCCCGCACAAGCGGAGGAACATGTGGTTTAATTCGATGATACGCGAGGAACCTTACCCGG<br>GATTGAAATTTAGATGGCGGCAGATGAGAGTTTGCTTTCCTTCGGGACATCTAAGTAGGTGCTGCATGGTTGTCGTCAGCT<br>CGTGCCGTGAGGTGTCGGCTTAAGTGCCATAACGAGCGCAACCCGCGTCGATAGTTACTAACAGGTTATGCTGAGGACTC<br>TATCGAGACAGCCGTCGTAAGACGTGAGGAAGGGGCGGATGACGTCAAATCAGCACGGCCCTTACATCCGGGGCGACAC<br>ACGTGTTACAATGGCAGGGACAAAGGGAAGCGACATGGTGACATGAAGCGGATCTCCAAACCCTGTCCCAGTTCGGATCG<br>GAGTCTGCAACTCGACTCCGTGAAGCTGGATTCGCTAGTAATCGCGCATCAGCCATGGCGCGGTGAATACGTTCCCGGG<br>CCTTGTACACACCGCCCGTCAAGCCATGGAAGTTGGGAGTACCTGAAGAGCGTAACCGGAAGGGGCGCTTAAGGGTAATA<br>CCAGTGACTGGGGCT

Sr no.	Species_OTUs	16S reference sequence
209	Neisseria sp. OTU_1144	ATTGAACGCTGGCGGCATGCTTTACACATGCAAGTCGGACGGCAGCGGGGTAGTGCTTGCACTACTGCCGGCGAGTGGCGAACGGGTGAGTAACATATCGGAACGTACCGAGCAGTGGGGGATAACTAATCGAAAGATTAGCTAATACCGCATATCTTCTGAGGAAGAAAGCAGGGGACCTTTGGGCCTTGCGCTGTTTGAGCGGCCGATATCTGATTAGCTGGTTGGTGGGGTAAAGGCCTACCAAGGCGACGATCAGTAGCGGGTCTGAGAGGATGATCCGCCACACTGGGACTGAGACACGGCCCAGACTCCTACGGGAGGCAGCAGTGGGGAATTTTGGACAATGGGGGCAACCCTGATCCAGCCATGCCGCGTGTCTGAAGAAGGCCTTCGGGTTGTAAAGGACTTTTGTCCGGGAAGAAAAGCGCGATGTTAATACCATTGCGTGCTGACGGTACCGGAAGAATAAGCACCGGCTAACTACGTGCCAGCAGCCGCGGTAATACGTAGGGTGCGAGCGTTAATCGGAATTACTGGGCGTAAAGCGGGCGCAGACGGTTACTTAAGCAGGATGTGAAATCCCCGGGCTCAACCTGGGAATTGCGTTCTGAACTGGGTGGCTAGAGTGTGTCAGAGGGGGGTAGAATTCCACGTGTAGCAGTGAAATGCGTAGAGATGTGGAGGAATACCGATGGCGAAGGCAGCCCCCTGGGATAGCACTGACGTTCATGCCCGAAAGCGTGGGTAGCAAACAGGATTAGATACCCTGGTAGTCCACGCCCTAAACGATGTCGATTAGCTGTTGGGCAACTTGATTGCTTGGTAGCGAAGCTAACGCGTGAAATCGACCGCCTGGGGAGTACGGTCGCAAGATTAAAACTCAAAGGAATTGACGGGGACCCGCACAAGCGGTGGATGATGTGGATTAATTCGATGCAACGCGAAGAACCTTACCTGGTCTTGACATGTACGGAACCTTCCGGAGACGGAAGGGTGCCTTCGGGAGCCGTAACACAGGTGCTGCATGGCTGTCGTCAGCTCGTGTCGTGAGATGTTGGGTTAAGTCCCGCAACGAGCGCAACCCTTGTCATTAGTTGCCATCATTTGGTTGGGCACTCTAATGAGACTGCCGGTGACAAGCCGGAGGAAGGTGGGGATGACGTCAAGTCCTCATGGCCCTTATGACCAGGGCTTCACACGTCATACAATGGTCGGTACAGAGGGTAGCCAAGCCGCGAGGTGGAGCCAATCCCAAAAAACCGATCGTAGTCCGGATTGCACTCTGCAACTCGAGTGCATGAAGTCGGAATCGCTAGTAATCGCAGGTCAGCATACTGCGGTGAATACGTTCCCGGGTCTTGTACACACCGCCCGTCACACCACGAGAGTTTGTAACACCCGAAGTCGGTGAGGTAACCGTAAGGAGCCAGCCGCCTAAGGTGGGATAGATGATTGGGGTG
210	Streptococcus sanguinis OTU_1147	GACGAACGCTGGCGGCGTGCTTAACACATGCAAGTCGAACGGGCTCTGTCTGGCTGTGTGCTGGGTGGGGTTAGTGGCGAACGGGTGAGTAATACGTGAGTAACCTGCCCTCTTCTTTGGGATAAGCCTTGGAAACGGGGTCTAATACTGGATATTCCTGTCTGGTCGCATGGCTGGGTGGGGAAAGTTTTTTCGGTGGGGGATGGGCTCGCGGCCTATCAGCTTGTTGGTGGGGTGATGGCCTACCAAGGCGTCGACGGGTAGCCGGCCTGAGAGGGTGACCGGCCACACTGGGACTGAGATACGGCCCAGACTCCTACGGGAGGCAGCAGTGGGGGATATTGCACAATGGGCGCAAGCCTGATGCAGCGACGCCGCGTGGGGGATGACGGCTTTCGGGTTGTAAACCTCTTTCGTCGGGGGACAAGGCTCTGCTTGGTGGTGGGGTTGAGGGTACCCGGTGAAGAAGCGCCGGCTAACTACGTGCCAGCAGCCGCGGTAATACGTAGGGCGCGAGCGTTGTCCGGAATTATTGGGCGTAAAGCGAGCGCAGGCGGTTAGATAAGTCTGAAGTTAAAGGCTGTGGCTTAACCATAGTATGCTTTGGAAACTGTTTAACTTGAGTGCAGAAGGGGAGAGTGGAATTCCATGTGTAGCGGTGAAATGCGTAGATATATGGAGGAACACCGGTGGCGAAAGCGGCTCTCTGGTCTGTAACTGACGCTGAGGCTCGAAAGCGTGGGGAGCAAACAGGATTAGATACCCTGGTAGTCCACGCCGTAAACGATGAGTGCTAGGTGTTAGGCCCTTTCCGGGGCTTAGTGCCGCAGCTAACGCATTAAGCACTCCGCCTGGGGAGTACGACCGCAAGGTTGAAACTCAAAGGAATTGACGGGGGCCCGCACAAGCGGTGGAGCATGTGGTTTAATTCGAAGCAACGCGAAGAACCTTACCAGGTCTTGACATCCCTCTGACCGCTCTAGAGATAGAGTTTTCCTTCGGGACAGAGGTGACAGGTGGTGCATGGTTGTCGTCAGCTCGTGTCGTGAGATGTTGGGTTAAGTCCCGCAACGAGCGCAACCCCTATTGTTAGTTGCCATCATTCAGTTGGGCACTCTAGCGAGACTGCCGGTAATAAACCGGAGGAAGGTGGGGATGACGTCAAATCATCATGCCCCTTATGACCTGGGCTACACACGTGCTACAATGGCTGGTACAACGAGTCGCAAGCCGGTGACGGCAAGCTAATCTCTGAAAGCCAGTCTCAGTTCGGATTGTAGGCTGCAACTCGCCTACATGAAGTCGGAATCGCTAGTAATCGCGGATCAGCACGCCGCGGTGAATACGTTCCCGGGCCTTGTACACACCGCCCGTCACACCACGAGAGTTTGTAACACCCGAAGTCGGTGAGGTAACCGTAAGGAGCCAGCCGCCTAAGGTGGGATAGATGATTGGGGTG
211	Neisseriaceae gen. sp. OTU_1149	ATTGAACGCTGGCGGCATGCTTTACACATGCAAGTCGAACGGCAGCGGGGGAGTGCTTGCACTTCTGTCCGGCGAGTGGCGAACGGGTGAGTAATATATCGGAACGTACCGAGTAGTGGGGGATAACCAATCGAAAGATTGGCTAATACCGCATACGTCTTGAGAGAGAAAGCAGGGGACCATTTGGCCTTGCGCTATTCGAGCGGCCGATAACTGATTAGCTAGTTGGTGGGGTAAAGGCCTACCAAGGCGACGATCAGTAGCGGGTCTGAGAGGACGATCCGCCACACTGGGACTGAGACACGGCCCAGACTCCTACGGGAGGCAGCAGTGGGGAATTTTGGACAATGGGGGCAACCCTGATCCAGCCATGCCGCGTGTCTGAAGAAGGCCTTCGGGTTGTAAAGGACTTTTGTTCGGGAAGAAAAGCGCAGTGTTAATACCATTGCGTACTGACGGTACCGAAAGAATAAGCACCGGCTAACTACGTGCCAGCAGCCGCGGTAATACGTAGGGTGCGAGCGTTAATCGGAATTACTGGGCGTAAAGCGAGCGCAGACGGTTATTTAAGCAGGATGTGAAATCCCCGGGCTTAACCTGGGAACTGCGTTCTGAACTGGATAGCTAGAGTATGTCAGAGGGGGGTAGAATTCCACGTGTAGCAGTGAAATGCGTAGAGATGTGGAGGAATACCGATGGCGAAGGCAGCCCCCTGGGATAATACTGACGTTCATGCTCGAAAGCGTGGGTAGCAAACAGGATTAGATACCCTGGTAGTCCACGCCCTAAACGATGTCGATTAGCTGTTGGGCAACTTGATTGCTTAGTAGCGTAGCTAACGCGTGAAATCGACCGCCTGGGGAGTACGGTCGCAAGATTAAAACTCAAAGGAATTGACGGGGACCCGCACAAGCGGTGGATGATGTGGATTAATTCGATGCAACGCGAAGAACCTTACCTGGTCTTGACATGTACGGAACCTTCCAGAGACGGAAGGGTGCCTTCGGGAACCGTAACACAGGTGCTGCATGGCTGTCGTCAGCTCGTGTCGTGAGATGTTGGGTTAAGTCCCGCAACGAGCGCAACCCTTGTCATTAGTTGCCATCATTTAGTTGGGCACTCTAATGAGACTGCCGGTGACAAACCGGAGGAAGGTGGGGATGACGTCAAGTCCTCATGGCCCTTATGACCAGGGCTTCACACGTCATACAATGGTCGGTACAGAGGGTAGCCAAGCCGCGAGGTGGAGCCAATCCCAGAAAACCGATCGTAGTCCGGATTGCACTCTGCAACTCGAGTGCATGAAGTCGGAATCGCTAGTAATCGCAGGTCAGCATACTGCGGTGAATACGTTCCCGGGTCTTGTACACACCGCCCGTCACACCATGGGAGTGGGGGATACCAGAAGCAGGTAGGATAACCGCAAGGGGTCCGCTTGCCACGGTATGCTTCATGACTGGGGTG
212	Prevotella sp. OTU_1152	GATGAACGCTAGCTACAGGCTTAACACATGCAAGTCGAGGGGAAACGGCATTAAGTGCTTGCACTTTTTGGACGTCGACCGGCGCACGGGTGAGTAACGCGTATCCAACCTTCCCATGACTAAGTGATAACCTGCCGAAAGGCAGACTAATACCTTATGGTCTTCACTGACGGCATCAGATGTGAAGTAAAGATTTATCGGTTATGGATGGGGATGCGTCTGATTAGTTAGTTGGCGGGGTAACGGCCCACCAAGGCAACGATCAGTAGGGGTTCTGAGAGGAAGGTCCCCCACATTGGAACTGAGACACGGTCCAAACTCCTACGGGAGGCAGCAGTGAGGAATATTGGTCAATGGGCGAGAGCCTGAACCAGCCAAGTAGCGTGCAGGAAGACGGCCCTATGGGTTGTAAACTGCTTTTATACGAGAATAATTTGATGCACGTGTGCGTTATTGCATGTATCGTATGAATAAGGACCGGCTAATTCCGTGCCAGCAGCCGCGGTAATACGGAAGGTCCAGGCGTTATCCGGATTTATTGGGTTTAAAGGGAGTGTAGGCGGTTTGTTAAGCGTGTTGTGAAATTTAGGTGCTCAACATTTAACTTGCAGCGCGAACTGTCAGACTTGAGTACACGCAACGTATGCGGAATTCATGGTGTAGCGGTGAAATGCTTAGATATCATGAAGAACTCCGATTGCGAAGGCAGCATACGGGAGTGTAACTGACGCTTAAGCTCGAAGGTGCGGGTATCGAACAGGATTAGATACCCTGGTAGTCCGCACAGTAAACGATGGATGCCCGCTGTTAGCGCCTGGCGTTAGCGGCTAAGCGAAAGCATTAAGCATCCCACCTGGGGAGTACGCCGGCAACGGTGAAACTCAAAGGAATTGACGGGGGCCCGCACAAGCGGAGGAACATGTGGTTTAATTCGATGATACGCGAGGAACCTTACCCGGGCTTGAATTGCAGAGGAATTATTTAGAGATAAATATGCCCTTCGGGGTCTCTGTGAAGGTGCTGCATGGTTGTCGTCAGCTCGTGCCGTGAGGTGTCGGCTTAAGTGCCATAACGAGCGCAACCCCTTTTCTTAGTTGCCATCAGGTAATGCTGGGCACTCTGGGAACACTGCCACCGCAAGGTGTGAGGAAGGTGGGGATGACGTCAAATCAGCACGGCCCTTACGTCCGGGGCTACACACGTGTTACAATGGCCGGTACAGAGGGATGGTGTAATGTAAATTGCATCAAATCTTAAAAGCCGGTCCCAGTTCGGACTGAGGTCTGCAACCCGACCTCACGAAGCTGGATTCGCTAGTAATCGCGCATCAGCCATGGCGCGGTGAATACGTTCCCGGGCCTTGTACACACCGCCCGTCAAGCCATGAAAGCCGGGGGTGCCTGAAGTTCGTAACCGTAAGGAGCGACCTAGGGCAAAACTGGTAATTGGGGCT

Sr no.	Species_OTUs	16S reference sequence
213	Fudania sp. OTU_1153	GACGAACGCTGGCGGCGTGCCTAATACATGCAAGTAGAACGCACAGGATACACCGTAGTTTACTACACCGTATTCTGTGAG<br>TTGCGAACGGGTGAGTAACGCGTAGGTAACCTGCCTGGTAGCGGGGGATAACTATTGGAAACGATAGCTAATACCGCATAA<br>GAACATTTACTGCATGGTAGATGTTTAAAAGGTGCAAATGCATCACTACCAGATGGACCTGCGTTGTATTAGCTAGTAGGTGA<br>GGTAACGGCTCACCTAGGCGACGATACATAGCCGACCTGAGAGGGTGATCGGCCACACTGGGACTGAGACACGGCCCAG<br>ACTCCTACGGGAGGCAGCAGTGGGGGATATTGCACAATGGGCGCAAGCCTGATGCAGCGACGCCGCGTGGGGGATGAC<br>GGCTTTCGGGTTGTAAACCTCTTTCGTCGGGGGACAAGGCTCTGCTTGGTGGTGGGGTTGAGGGTACCCGGTGAAGAAG<br>CGCCGGCTAACTACGTGCCAGCAGCCGCGGTAATACGTAGGGCGCGAGCGTTGTCCGGAATTATTGGGCGTAAAGGGCT<br>CGTAGGCGGCTTGTCGCGTCTGCTGTGAAAATGCGGGGCTTAACTCCGTACGTGCAGTGGGTACGGGCAGGCTAGAGTG<br>CGGTAGGGGTGACTGGAATTCCTGGTGTAGCGGTGGAATGCGCAGATATCAGGAGGAACACCGATGGCGAAGGCAGGTCA<br>CTGGGCTGTTACTGACGCTGAGGAGCGAAAGCGTGGGGAGCGAACAGGATTAGATACCCTGGTAGTCCATGCTGTAAACG<br>TTGGGAACTAGGTGTGGGGTTCTTTCCACGGATTCTGCGCCGGAGCTAACGCATTAAGTTCCCCGCCTGGGGAGTACGG<br>CCGCAAGGCTAAAACTCAAAGGAATTGACGGGGGCCCGCACAAGCGGCGGAGCATGCGGATTAATTCGATGCAACGCGAA<br>GAACCTTACCAAGGCTTGACATACGCTGGGACGGTGCAGAGATGTGCTGGCTTTTGCTGGTGTACAGGTGGTGCATGGTT<br>GTCGTCAGCTCGTGTCGTGAGATGTTGGGTTAAGTCCCGCAACGAGCGCAACCCTTGTCTCGTGTTGCCAGCAGGTTGG<br>GCTGGGGACTCACGGGAGACTGCCGGGGTTAACTCGGAGGAAGGTGGGGATGACGTCAAATCATCATGCCCCTTATGTCT<br>TGGGCTTCACGCATGCTACAATGGCCGGTACAGAGGGTTGCGATATCGTGAGGTGGAGCGAATCCCTTAAAGTCGGTCTC<br>AGTTCGGATCGGGGTCTGCAACTCGACCCCGTGAAGTCGGAGTCGCTAGTAATCGCGGATCAGCAACGCCGCGGTGAAT<br>ACGTTCTCGGGCCTTGTACACACCGCCCGTCACGTCACGAAAGTTGGCAACACCCGAAGCTCGTGGCCTAACCCGTGTG<br>GGGGGGGAGCGGTCGAAGGTGGGGTTGGCGATTGGGACG
214	Streptococcus sp. OTU_1155	GACGAACGCTGGCGGCGTGCCTAATACATGCAAGTAGAACGCTGAAGGAGGAGCTTGCTCTTCTGGATGAGTTGCGAACG<br>GGTGAGTAACGCGTAGGTAACCTGCCTGGTAGCGGGGGATAACTATTGGAAACGATAGCTAATACCGCATAACAACAGTTG<br>TTGCATGACAGCTGTTTAAAAGGTGCAATTGCATCACTACCAGATGGACCTGCGTTGTATTAGCTAGTTGGTGGGGTAACGG<br>CTCACCAAGGCAACGATACATAGCCGACCTGAGAGGGTGATCGGCCACACTGGGACTGAGACACGGCCCAGACTCCTAC<br>GGGAGGCAGCAGTAGGGAATCTTCGGCAATGGACGGAAGTCTGACCGAGCAACGCCGCGTGAGTGAAGAAGGTTTTCGGA<br>TCGTAAAGCTCTGTTGTAAGAGAAGAACGAGTGTGAGAGTGGAAAGTTCACACTGTGACGGTATCTTACCAGAAAGGGACGG<br>CTAACTACGTGCCAGCAGCCGCGGTAATACGTAGGTCCCGAGCGTTGTCCGGATTTATTGGGCGTAAAGCGAGCGCAGG<br>CGGTTAGATAAGTCTGAAGTTAAAGGCTGTGGCTTAACCATAGTACGCTTTGGAAACTGTTTAACTTGAGTGCAAGAGGGGA<br>GAGTGGAATTCCATGTGTAGCGGTGAAATGCGTAGATATATGGAGGAACACCGGTGGCGAAAGCGGCTCTCTGGCTTGTAA<br>CTGACGCTGAGGCTCGAAAGCGTGGGGAGCAAACAGGATTAGATACCCTGGTAGTCCACGCCGTAAACGATGAGTGCTAG<br>GTGTTAGACCCTTTCCGGGGTTTAGTGCCGCAGCTAACGCATTAAGCACTCCGCCTGGGGAGTACGACCGCAAGGTTGAA<br>ACTCAAAGGAATTGACGGGGGCCCGCACAAGCGGTGGAGCATGTGGTTTAATTCGAAGCAACGCGAAGAACCTTACCAGG<br>ACTTGACATCCTTCTGACCGGTCTTTAATAGGACCTTTCTTTTGACAGAAGAGACAGGTGGTGCATGGTTGTCGTCAGCTC<br>GTGTCGTGAGATGTTGGGTTAAGTCCCGCAACGAGCGCAACCCTTGTCATTAGTTGCCATCATTAAGTTGGGCACTCTAGT<br>GAGACTGCCGGGGACAACTCGGAGGAAGGTGGGGATGACGTCAAATCATCATGCCCCTTATGTTCTGGGCTACACACGTG<br>CTACAATGGCTGGTACAAAGAGAAGCAAGACCGCAAGGTGGAGCAAAGCTCAAAAACCAGCCCCAGTTCGGATTGTAGGCT<br>GAAACTCGCCTACATGAAGTCGGAGTTGCTAGTAATCGCAGATCAGAATGCTGCGGTGAATGCGTTCCCGGGTCTTGTACA<br>CACCGCCCGTCACACCATGGAAGTTGGGGGCGCCCAAAGTTGGCAGATGAATATGCTACCTAAGGTGAAACCAATGACTG<br>GGGTG
215	Pseudoramibacter sp. OTU_1156	GATGAACGCTAGCTATAGGCTTAACACATGCAAGTCGAGGGGAAACGGCATTATGTGCTTGCACATTTTGGACGTCGACCG<br>GCGCACGGGTGAGTATCGCGTATCCAACCTTCCCTCCACTCGGGGATACCCCGTTGAAAGACGGCCTAATACCCGATGTT<br>GTCCACATATGGCATCTGACGTGGACCAAAGATTCATCGGTGGAGGATGGGGATGCGTCTGATTAGCTTGTTGGTGCGGG<br>TAACGGCCCACCAAGGCTACGATCAGTAGGGGTTCTGAGAGGAAGGTCCCCCACATTGGAACTGAGACACGGTCCAAACT<br>CCTACGGGAGGCAGCAGTGGGGAATATTGCGCAATGGGGGCAACCCTGACGCAGCAATACCGCGTGAGTGAAGAAGGTTT<br>TCGGATCGTAAAGCTCTGTTATTGGGGAAGAAGCAGTGACGGTACCCAATGAGGAAGTCCCGGCTAACTACGTGCCAGCA<br>GCCGCGGTAATACGTAGGGGACGAGCGTTGTCCGGAATCACTGGGCGTAAAGGGCGCGTAGGCGGTTTTATAAGTCAGAT<br>GTGAAAGGTACCGGCTCAACCGGTGACGTGCATTTGAAACTGTAAGACTTGAGTACTGAAGAGGCAAGCGGAATTCCTAGT<br>GTAGCGGTGAAATGCGTAGATATTAGGAAGAACACCGGTGGCGAAGGCGGCTTGCTGGGCAGATACTGACGCTGAGGTGC<br>GAAAGCGTGGGGAGCGAACAGGATTAGATACCCTGGTAGTCCACGCCGTAAACGATGAATACTAGGTGTTGGCGAATAAGT<br>CAGTGCCGGAGTTAACACAATAAGTATTCCGCCTGGGGAGTACGACCGCAAGGTTGAAACTCAAAGGAATTGACGGGGACC<br>CGCACAAGCAGCGGAGCATGTGGTTTAATTCGAAGCAACGCGAAGAACCTTACCAGGCCTTGACATCCTCTGAGCGCAATA<br>GAGATATTGCTTTCTCTTCGGAGACAGAGAGACAGGTGGTGCATGGTTGTCGTCAGCTCGTGTCGTGAGATGTTGGGTTAA<br>GTCCCGCAACGAGCGCAACCCCTGTCATTAGTTGCCATCATTAAGTTGGGCACTCTAATGAGACTGCCGTAGACAATACGG<br>AGGAAGGTGGGGACGACGTCAAATCATCATGCCCCTTATGGCCTGGGCTACACACGTGCTACAATGGTCTGAACAAAGGG<br>AAGCGAAGGAGCGATCCGGAGCGAATCTCATAAAACAGATCCCAGTTCGGATTGCAGGCTGCAACTCGCCTGCATGAAGAT<br>GGAGTTGCTAGTAATCGCGGATCAGAATGTCGCGGTGAATGCGTTCCCGGGTCTTGTACACACCGCCCGTCACACCACGA<br>GAGTCGGTAACACCCGAAGCCAGTGAGACAACCGGAAGGAGTCAGCTGTCGAAGGTGGGATCGGTAATTGGGGTG
216	Saccharibacteria sp. OTU_1157	GATGAACGCTGGCGGCGTGCCTAACACATGCAAGTCGAGCGGCAGCGAAGGGTGCTTGCACCCTGTCGGCGAGCGGCG<br>GACGGCTGAGTAACGCGTGGGAACGTGCCCCAAAGTGAGGAATAACTGCCCGAAAGGGTAGCTAATACCGCATATTATCTT<br>CGGATCAAAGGATTATCCGCTTTGGGAGCGGCTCGCGTCGGATTAGGTAGTTGGTGAGGTAATGGCTCACCAAGCCGACG<br>ATCCGTAGCTGGTCTGAGAGGATGACCAGCCAGACTGGAACTGAGACACGGTCCAGACTCCTACGGGAGGCAGCAGTGA<br>GGAATCTTCCACAATGGGCGAAAGCCTGATGGAGCAACGCCGCGTGCAGGACGAAGGCCTTCGGGTTGTAAACTGCTTTT<br>ATAAGTGAGGAATATGACGGTAACTTATGAATAAGGATCGGCTAACTACGTGCCAGCAGCCGCGGTCATACGTAGGATCCG<br>AGCGTTATCCGGAGTGACTGGGCGTAAAGAGTTGCGTAGGCGGTTGTATAAGTGAATAGTGAAATCTGATGGCTCAACCATA<br>CAGGCTATTATTCAAACTGTACAACTCGAGAGTGGTAGAGGTCACTGGAATTTCTTGTGTAGGAGTGAAATCCGTAGATATAA<br>GAAGGAACACCGATGGCGTAGGCAGGTGACTGGACCATTTCTGACGCTAAGGCACGAAAGCGTGGGGAGCGAACCGGATT<br>AGATACCCGGGTAGTCCACGCCGTAAACGATGGATACTGGCTGTTGGAGGTATCGACCCCTTCAGTAGCGAAGCTAACGC<br>GTTAAGTATCCCGCCTGTGGAGTACGGCCGCAAGGCTAAAACATAAAGGAATTGACGGGGACCCGCACAAGCGGTGGATC<br>GTGTTCTTTAATTCGATGCTAAACGAAGAACCTTACCAGGGCTTGACATCCAGGGAAGGTCTGCGAAAGTGGACTGTGCCT<br>TTTGGAACCCTGTGACAGGTGATGCATGGCCGTCGTCAGCTCGTGTCGTGAGATGTTTGGTTAAGTCCATCAACGAGCGC<br>AACCCTTGTGTCTAGTTGTATTTTTCTAGACAGACTGCTTCGGTAACGGAGAGGAAGGAGGGGATGATGTCAGGTCAGTATT<br>TCCCTTACGTCCTGGGCTAGAAACACGATACAATGGCTAGTACAATGCGCAGCGAAGCCGCGAGGTGAAGCAAATCGCAC<br>CAAAGCTAGTCCCAGTTCGGATTGGAGGCTGAAACTCGCCTCCATGAAGTCGGAATCGCTAGTAATCGCAAATCAGCAAGT<br>TGCGGTGAATACGTTCTCGGGTCTTGTACACACCGCCCGTCACACCACGAGAGTTGGTTGCACCTGAAGTAGCAGGCCTA<br>ACCGTAAGGAGGGATGCTCCGAGGGTGTGATTAGCGATTGGGGTG

Sr no.	Species_OTUs	16S reference sequence
217	Prevotella sp. OTU_1158	GATGAACGCTAGCTATAGGCTTAACACATGCAAGTCGAGGGGAAACGGCATTATGTGCTTGCACATTTTGGACGTCGACCG<br>GCGCACGGGTGAGTATCGCGTATCCAACCTTCCCTCCACTCGGGGATACCCCGTTGAAAGACGGCCTAATACCCGATGTT<br>GTCCACATATGGCATCTGACGTGGACCAAAGATTCATCGGTGGAGGATGGGGATGCGTCTGATTAGCTTGTTGGTGCGGG<br>TAACGGCCCACCAAGGCTACGATCAGTAGGGGTTCTGAGAGGAAGGTCCCCCACATTGGAACTGAGACACGGTCCAAACT<br>CCTACGGGAGGCAGCAGTGAGGAATATTGGTCAATGGACGTAAGTCTGAACCAGCCAAGTAGCGTGCAGGATTGACGGCC<br>CTATGGGTTGTAAACTGCTTTTGTTGGGGAGTAAAGTGGGGCACGCGTGCCTTTTTGCATTTACCCTTCGAATAAGGACCG<br>GCTAATTCCGTGCCAGCAGCCGCGGTAATACGGAAGGTCCAGGCGTTATCCGGATTTATTGGGTTTAAAGGGAGTGTAGG<br>CGGTCTGTTAAGCGTGTTGTGAAATTTAGGTGCTCAACATTTAACTTGCAGCGCGAACTGGCGGACTTGAGTGCACGCAAC<br>GTATGCGGAATTCATGGTGTAGCGGTGAAATGCTTAGATATCATGACGAACTCCGATTGCGAAGGCAGCGTACGGGAGTGT<br>TACTGACGCTTAAGCTCGAAGGTGCGGGTATCGAACAGGATTAGATACCCTGGTAGTCCGCACGGTAAACGATGGATGCC<br>CGCTGTTAGCGCCTGGCGTTAGCGGCTAAGCGAAAGCATTAAGCATCCCACCTGGGGAGTACGCCGGCAACGGTGAAAC<br>TCAAAGGAATTGACGGGGGCCCGCACAAGCGGAGGAACATGTGGTTTAATTCGATGATACGCGAGGAACCTTACCCGGGC<br>TTGAATTGCAGACGCAGGATACAGAGATGTTGACTCCCTTCGGGGCGTCTGTGAAGGTGCTGCATGGTTGTCGTCAGCTC<br>GTGCCGTGAGGTGTCGGCTTAAGTGCCATAACGAGCGCAACCCCTTTCCTTAGTTGCCATCAGGTGATGCTGGGCACTCT<br>GGGGATACTGCCACCGCAAGGTGTGAGGAAGGTGGGGATGACGTCAAATCAGCACGGCCCTTACGTCCGGGGCTACACA<br>CGTGTTACAATGGTCGGAGCAGCAGGAAGCTACCTGGCGACAGGATGCTGATCCCAAAACCCGGCCTCAGTTCGGACTG<br>GAGTCTGCAACCCGACTCCACGAAGCCGGATTCGCTAGTAATCGCGCATCAGCCATGGCGCGGTGAATACGTTCCCGGG<br>CCTTGTACACACCGCCCGTCAAGCCATGAAAGCCGGGGGTGCCTGAAAGCCGTGACCGCGAGGGTCGGCCTAGGGTAAA<br>ACCGGTGATTGGGGCT
218	Streptococcus sp. OTU_1159	ATTGAACGCTGGCGGCAGGCTTAACACATGCAAGTCGAACGGTAACATAAAGAAGCTTGCTTCTTTGATGACGAGTGGCGG<br>ACGGGTGAGTAATGCTTGGGAATCTAGCTTATGGAGGGGGATAACTACGGGAAACTGTAGCTAATACCGCGTAATATCGGAA<br>GATTAAAGTGTGGGACCTTCGGGCCACATGCCATAGGATGAGCCCAAGTGGGATTAGGTAGTTGGTGAGGTAAAGGCTCAC<br>CAAGCCGACGATCTCTAGCTGGTCTGAGAGGATGACCAGCCACACTGGGACTGAGACACGGCCCAGACTCCTACGGGAG<br>GCAGCAGTAGGGAATCTTCGGCAATGGACGGAAGTCTGACCGAGCAACGCCGCGTGAGTGAAGAAGGTTTTCGGATCGTA<br>AAGCTCTGTTGTAAGAGAAGAACGAGTGTGAGAGTGGAAAGTTCACACTGTGACGGTATCTTACCAGAAAGGGACGGCTAAC<br>TACGTGCCAGCAGCCGCGGTAATACGTAGGTCCCGAGCGTTGTCCGGATTTATTGGGCGTAAAGCGAGCGCAGGCGGTT<br>AGATAAGTCTGAAGTTAAAGGCTGTGGCTTAACCATAGTACGCTTTGGAAACTGTTTAACTTGAGTGCAAGAGGGGAGAGTG<br>GAATTCCATGTGTAGCGGTGAAATGCGTAGATATATGGAGGAACACCGGTGGCGAAAGCGGCTCTCTGGCTTGTAACTGAC<br>GCTGAGGCTCGAAAGCGTGGGGAGCAAACAGGATTAGATACCCTGGTAGTCCACGCCGTAAACGATGAGTGCTAGGTGTT<br>AGACCCTTTCCGGGGTTTAGTGCCGCAGCTAACGCATTAAGCACTCCGCCTGGGGAGTACGACCGCAAGGTTGAAACTCA<br>AAGGAATTGACGGGGGCCCGCACAAGCGGTGGAGCATGTGGTTTAATTCGAAGCAACGCGAAGAACCTTACCAGGTCTTG<br>ACATCCCTCTGACCGCTCTAGAGATAGAGTTTTCCTTCGGGACAGAGGTGACAGGTGGTGCATGGTTGTCGTCAGCTCGT<br>GTCGTGAGATGTTGGGTTAAGTCCCGCAACGAGCGCAACCCCTATTGTTAGTTGCCATCATTTAGTTGGGCACTCTAGCGA<br>GACTGCCGGTAATAAACCGGAGGAAGGTGGGGATGACGTCAAATCATCATGCCCCTTATGACCTGGGCTACACACGTGCT<br>ACAATGGCTGGTACAACGAGTCGCAAGCCGGTGACGGCAAGCTAATCTCTTAAAGCCAGTCTCAGTTCGGATTGTAGGCTG<br>CAACTCGCCTACATGAAGTCGGAATCGCTAGTAATCGCGGATCAGCACGCCGCGGTGAATACGTTCCCGGGCCTTGTACA<br>CACCGCCCGTCACACCACGAGAGTTTGTAACACCCGAAGTCGGTGAGGTAACCGTAAGGAGCCAGCCGCCTAAGGTGGG<br>ATAGATGATTGGGGTG
219	Neisseriaceae gen. sp. OTU_1160	ATTGAACGCTGGCGGCATGCTTTACACATGCAAGTCGGACGGCAGCGGGGTAGTGCTTGCACTACTGCCGGCGAGTGGC<br>GAACGGGTGAGTAATATATTGGAACGTACCGAGTAATGGGGGATAACCAATCGAAAGATTGGCTAATACCGCATACGCTCTG<br>AGGAGGAAAGCAGGGGACCTTCGGGCCTTGCGTTATTCGAGCGGCCAATATCTGATTAGCTAGTTGGTGGGGTAAAGGCC<br>TACCAAGGCGACGATCAGTAGCGGGTCTGAGAGGATGATCCGCCACACTGGGACTGAGACACGGCCCAGACTCCTACGG<br>GAGGCAGCAGTGGGGAATTTTGGACAATGGGCGCAAGCCTGATCCAGCCATGCCGCGTGTCTGAAGAAGGCCTTCGGGT<br>TGTAAAGGACTTTTGTTAGGGAAGAAAAAGGAGCGGTTAATACCCGTTTCTGCTGACGGTACCTAAAGAATAAGCACCGGCT<br>AACTACGTGCCAGCAGCCGCGGTAATACGTAGGGTGCGAGCGTTAATCGGAATTACTGGGCGTAAAGCGAGCGCAGACGG<br>TTACTTAAGCAGGATGTGAAATCCCCGGGCTCAACCTGGGAACTGCGTTCTGAACTGGGTAGCTAGAGTATGTCAGAGGGG<br>GGTAGAATTCCACGTGTAGCAGTGAAATGCGTAGAGATGTGGAGGAATACCGATGGCGAAGGCAGCCCCCTGGGATAATAC<br>TGACGTTCATGCTCGAAAGCGTGGGTAGCAAACAGGATTAGATACCCTGGTAGTCCACGCCCTAAACGATGTCGATTAGCT<br>GTTGGGCAGCTTGACTGCTTAGTAGCGTAGCTAACGCGTGAAATCGACCGCCTGGGGAGTACGGTCGCAAGATTAAAACT<br>CAAAGGAATTGACGGGGACCCGCACAAGCGGTGGAGCATGTGGTTTAATTCGAAGCAACGCGAAGAACCTTACCAGGTCTT<br>GACATCCCTCTGACCGCTCTAGAGATAGAGTTTTCCTTCGGGACAGAGGTGACAGGTGGTGCATGGTTGTCGTCAGCTCG<br>TGTCGTGAGATGTTGGGTTAAGTCCCGCAACGAGCGCAACCCCTATTGTTAGTTGCCATCATTCAGTTGGGCACTCTAGCG<br>AGACTGCCGGTAATAAACCGGAGGAAGGTGGGGATGACGTCAAATCATCATGCCCCTTATGACCTGGGCTACACACGTGC<br>TACAATGGCTGGTACAACGAGTCGCAAGCCGGTGACGGCAAGCTAATCTCTTAAAGCCAGTCTCAGTTCGGATTGTAGGCT<br>GCAACTCGCCTACATGAAGTCGGAATCGCTAGTAATCGCGGATCAGCACGCCGCGGTGAATACGTTCCCGGGCCTTGTAC<br>ACACCGCCCGTCACACCACGAGAGTTTGTAACACCCGAAGTCGGTGAGGTAACCTTTTAGGAGCCAGCCGCCTAAGGTGG<br>GATAGATGATTGGGGTG
220	Porphyromonas sp. OTU_1164	GACGAACGCTGGCGGCGTGCTTAACACATGCAAGTCGAACGTGATTTTTGTGGAAATTCTTTCGGGAATGGAAATGAAATGA<br>AAGTGGCGAACGGGTGAGTAACACGTGAGCAACCTACCTTACACAGGGGGATAGCCGTTGGAAACGACGATTAATACCGCA<br>TGAGACCACAGAATCGCATGATATAGGGGTCAAAGATTTATCGGTGTAAGAAGGGCTCGCGTCTGATTAGCTAGTTGGAAGG<br>GTAAAGGCCTACCAAGGCGACGATCAGTAGCCGGTCTGAGAGGATGAACGGCCACATTGGAACTGAGACACGGTCCAAAC<br>TCCTACGGGAGGCAGCAGTGGGGAATATTGCACAATGGGGGGAACCCTGATGCAGCGACGCCGCGTGAGCGAAGAAGGT<br>TTTCGAATCGTAAAGCTCTGTCCTATGAGAAGATAATGACGGTATCATAGGAGGAAGCCCCGGCTAAATACGTGCCAGCAG<br>CCGCGGTAATACGTATGGGGCGAGCGTTGTCCGGAATTATTGGGTTTAAAGGGTGCGTAGGTGGCGTATTAAGTCAGTGGT<br>GAAAAGCTGCAGCTCAACTGTAGTCTTGCCGTTGAAACTGATATGCTAGAGAGGAGACGAGGTATGCGGAATGTGTGGTGT<br>AGCGGTGAAATGCATAGATATCACACAGAACGCCGATTGCGAAGGCAGCGTACCAGGCTCCGTCTGACACTGAAGCACGA<br>AAGCGTGGGGATCAAACAGGATTAGATACCCTGGTAGTCCACGCAGTAAACGATGAATACTAGATTTTTGCGATATACTGTAA<br>GAGTCTAAGCGAAAGCGATAAGTATTCCACCTGGGGAGTACGCCGGCAACGGTGAAACTCAAAGGAATTGACGGGGGCCC<br>GCACAAGCGGAGGAACATGTGGTTTAATTCGATGATACGCGAGGAACCTTACCCGGGATTGAAATTTAGATGGCGGCAGAT<br>GAGAGTTTGCTTTCCTTCGGGACATCTAAGTAGGTGCTGCATGGTTGTCGTCAGCTCGTGCCGTGAGGTGTCGGCTTAAG<br>TGCCATAACGAGCGCAACCCGCGTCGATAGTTACTAACAGGTTATGCTGAGGACTCTATCGAGACAGCCGTCGTAAGACGT<br>GAGGAAGGGGCGGATGACGTCAAATCAGCACGGCCCTTACATCCGGGGCGACACACGTGTTACAATGGCAGGGACAAAG<br>GGAAGCGACATGGTGACATGAAGCGGATCTCCAAACCCTGTCCCAGTTCGGATCGGAGTCTGCAACTCGACTCCGTGAAG<br>CTGGATTCGCTAGTAATCGCGCATCAGCCATGGCGCGGTGAATACGTTCCCGGGCCTTGTACACACCGCCCGTCAAGCC<br>ATGGAAGTTGGGAGTACCTGAAGAGCGTAACCGGAAGGGGCGCTTAAGGGTAATACCAGTGACTGGGGCT

Sr no.	Species_OTUs	16S reference sequence
221	Dialister sp. OTU_1165	GACGAACGCTGGCGGCGTGCTTAACACATGCAAGTCGAACGAAAAGAGGGAAAGAGCTTGCTCTTTCCGGAATTGAGTGG<br>CAAACGGGTGAGTAACACGTAAACAACCTGCCTTCAGGATGGGGACAACAGACGGAAACGACTGCTAATACCGAATAAGTT<br>CCAAGAGCCGCATGGCCCATGGAAGAAAAGGTGGCCTCTACCTGTAAGCTATCGCCTGAAGAGGGGTTTGCGTCTGATTA<br>GCTGGTTGGAGGGGTAACGGCCCACCAAGGCGACGATCAGTAGCCGGTCTGAGAGGATGAACGGCCACACTGGAACTGA<br>GACACGGTCCAGACTCCTACGGGAGGCAGCAGTGGGGAATCTTCCGCAATGGGCGAAAGCCTGACGGAGCAACGCCGC<br>GTGAGTGATGACGGCCTTCGGGTTGTAAAACTCTGTGATCCGGGACGAAAAGGCAGAGTGCGAAGAACAAACTGCATTGAC<br>GGTACCGGAAAAGCAAGCCACGGCTAACTACGTGCCAGCAGCCGCGGTAATACGTAGGTGGCAAGCGTTGTCCGGAATTA<br>TTGGGCGTAAAGCGCGCGCAGGCGGCTTCCCAAGTCCCTCTTAAAAGTGCGGGGCTTAACCCCGTGATGGGAAGGAAAC<br>TGGGAAGCTGGAGTATCGGAGAGGAAAGTGGAATTCCTAGTGTAGCGGTGAAATGCGTAGAGATTAGGAAGAACACCGGTG<br>GCGAAGGCGACTTTCTGGACGAAAACTGACGCTGAGGCGCGAAAGCGTGGGGAGCAAACAGGATTAGATACCCTGGTAGT<br>CCACGCCGTAAACGATGGATACTAGGTGTAGGAGGTATCGACCCCTCCTGTGCCGGAGTTAACGCAATAAGTATCCCGCC<br>TGGGAAGTACGATCGCAAGATTAAAACTCAAAGGAATTGACGGGGGCCCGCACAAGCGGTGGAGTATGTGGTTTAATTCGA<br>CGCAACGCGAAGAACCTTACCAGGTCTTGACATTGATGGACAGAACTAGAGATAGTTCCTCTTCTTCGGAAGCCAGAAAACA<br>GGTGGTGCACGGTTGTCGTCAGCTCGTGTCGTGAGATGTTGGGTTAAGTCCCGCAACGAGCGCAACCCCTATCTTATGTT<br>GCCAGCACGTAATGGTGGGAACTCATGAGAGACTGCCGCAGACAATGCGGAGGAAGGCGGGGATGACGTCAAATCATCAT<br>GCCCCTTATGACCTGGGCTACACACGTACTACAATGGGAGTTAATAGACGGAAGCGAGATCGCGAGATGGAGCAAACCCG<br>AGAAACACTCTCTCAGTTCGGATCGTAGGCTGCAACTCGCCTACGTGAAGTCGGAATCGCTAGTAATCGCAGGTCAGCATA<br>CTGCGGTGAATACGTTCCCGGGCCTTGTACACACCGCCCGTCACACCACGAAAGTCGGAAGTGCCCAAAGCCGGTGGGG<br>TAACCTTCGGGAGCCAGCCGTCTAAGGTAAAGTCGATGATTGGGGTG
222	Asteroleplasma sp. OTU_1166	GATAAACGCCGGCGGCGTGCCTAATACATGCAAGTCGAACGAGTGCAGCGATGCACGAGTGGCGTACGGGTGAGTAACAC<br>GTTGGTAACCTGCCCCGCAGACCGGGATACCCTAGTGAAAACTGGGCTAATACCGGATGGCGTCCCCAGGGCGGAAGCC<br>CGGGGGACCAAAGGCGGCCTCGGCCGCGCTGCGGGATGGACCTGCGTCGCATTAGCTAGTTGGCGGGATAAAAGCCCA<br>CCAAGGCCGGGATGCGTAGCCGAGCTGAGAGGCTAAACGGCCACATTGGGACTGAGACACGGCCCAGACTCCTACGGGA<br>GGCAGCAGTAGGGAGTATTTGGCAATGGGGGGAACCCTGACCGAGCAACGCCGCGTGAAGGATTGAGGCCTTCGGGTCG<br>TAAACTTCTGTGGAGGGCTGGGAAACGCGCGGGGAGGCAATGCCCCGCGCCTGACCGGCCCTCTAGAAAGCAACTGCTA<br>ACTACGTGCCAGCAGCAGCGGTAATACGTAGGTTGCAAGCGTTATCCGGAATTATTGGGCGTAAAGAGTGAGCAGGCGGC<br>CCGGCAAGCGGAAGGTCAAATGCGGCGGCTCAACCGTCGTACGCTTTCCGAACTGCGGGGCTGGAGTGCTGGAGAGGC<br>AAGCGGAACTCCACATGTAGCGGTGAAATGCGCAGATATGTGGAAGAACACCGGCGGCGAAGGCGGCTTGCTAGACAGCG<br>ACTGACGCTCAGTCACGAAAGCGTGGGGAGCAAATAGGCTTAGATACCCTAGTAGTCCACGCCGTAAACGTTGAGGACCA<br>GGTGTCGGGGGCATACCCCGGCGCCCAAGCTAACGCAGTGAGTCCTCCGCCTGGGTACTACGTTCGCAAGAATAAAACT<br>TAAAGGAATTGACGGGGGCCCGCACAAGCAGTGGAGCATGTGGTTTAATTCGTGGCTACGCGGAGAACCTTACCTGGGTT<br>TGACATCCCGCTAAAGGCCCGGGAGACCGGGAGATAGCTATAGCGGTGAACGGTGGTGCATGGCTGTCGTCAGCTCGTG<br>TCGTGAGATGTTGGGTTAAGTCCCGCAACGAGCGCAACCCCTGCCTTCAGTTGCCATCATCAAGTTGGGCACTCTGAAGG<br>GACTGCCGGCGCAAGCCGGAGGAAGATGGGGATGACGTCAAGTCATCACGCCCCCTATGCCCAGGGCTACACACGTGCT<br>ACAATGGCCGTCACAGCGCGTTGCGACACCGCGAGGTGGAGCGGATCGCTCAAAGACGGTCTCAGTTCGGATCGGGGTC<br>TGCAATTCGACCCCGTGAAGCCGGAATTGCTAGTAATCGCGGATCAGCACTGCCGCGGTGAATACGTTCCCGGGCCTTGT<br>ACACACTGCCCGTCACACCATGAGAGCCGGCAAGGCCCGAAAGCGTTTGCCCAACCCGCAAGGGGGGGCGGCGTCTAA<br>GGCAGGGCCGGTGATTGGGGTT
223	Porphyromonas sp. OTU_1168	ATTGAACGCTGGCGGCATGCTTTACACATGCAAGTCGGACGGCAGCGGGGTAGTGCTTGCACTACTGCCGGCGAGTGGC<br>GAACGGGTGAGTAACATATCGGAACGTACCGAGCAGTGGGGGATAACTAATCGAAAGATTAGCTAATACCGCATATATTCTG<br>AGGAAGAAAGCAGGGGACCTTTGGGCCTTGCGCTGTTTGAGCGGCCGATATCTGATTAGCTGGTTGGTGGGGTAAAGGCC<br>TACCAAGGCGACGATCAGTAGCGGGTCTGAGAGGATGATCCGCCACACTGGGACTGAGACACGGACCAGACTCCTACGG<br>GAGGCAGCAGTGAGGAATATTGGTCAATGGGCGAGAGCCTGAACCAGCCAAGTCGCGTGAAGGAAGACAGTCCTAAGGAT<br>TGTAAACTTCTTTTATACGGGAATAACGGGCGATACGAGTATTGCATTGAATGTACCGTAAGAATAAGCATCGGCTAACTCCG<br>TGCCAGCAGCCGCGGTAATACGGAGGATGCGAGCGTTATCCGGATTTATTGGGTTTAAAGGGTGCGTAGGTTGTTCGGTAA<br>GTCAGCGGTGAAACCTGAGCGCTCAACGTTCAGCCTGCCGTTGAAACTGCCGGGCTTGAGTTCAGTGGCGGCAGGCGGA<br>ATTCGTGGTGTAGCGGTGAAATGCATAGATATCACGAGGAACTCCGATTGCGAAGGCAGCTTGCCATACTGCGACTGACAC<br>TGAAGCACGAAGGCGTGGGTATCAAACAGGATTAGATACCCTGGTAGTCCACGCAGTAAACGATGATTACTAGGAGTTTGC<br>GATATACCGTCAAGCTTCCACAGCGAAAGCGTTAAGTAATCCACCTGGGGAGTACGCCGGCAACGGTGAAACTCAAAGGAA<br>TTGACGGGGGCCCGCACAAGCGGAGGAACATGTGGTTTAATTCGATGATACGCGAGGAACCTTACCCGGGATTGAAATGTA<br>GATGACGGATGGTGAAAACCGTCTTCCCTTCGGGGCTTCTATGTAGGTGCTGCATGGTTGTCGTCAGCTCGTGCCGTGAG<br>GTGTCGGCTTAAGTGCCATAACGAGCGCAACCCACATCGGTAGTTGCTAACAGTTTTCGCTGAGGACTCTACCGAGACTG<br>CCGTCGTAAGGCGTGAGGAAGGTGTGGATGACGTCAAATCAGCACGGCCCTTACATCCGGGGCGACACACGTGTTACAAT<br>GGGAGGGACAAAGGGCAGCTACCGGGCGACCGGATGCGAATCTCTAAACCCTTCCCCAGTTCGGATCGGAGTCTGCAAC<br>TCGACTCCGTGAAGCTGGATTCGCTAGTAATCGCGCATCAGCCATGGCGCGGTGAATACGTTCCCGGGCCTTGTACACAC<br>CGCCCGTCAAGCCATGGGAGTCGGGGGTACCTGAAGGGCGTAACCGCAAGGGGCGCACTAGGGTAATACCGGTGACTG<br>GGGCT

J. Full-16S and metatranscriptomic sequencing reads per sample

Sr no	Sample_ID	16S filtered reads	RNAseq Raw reads	RNAseq humanreads (%)	RNAseq rRNA (%)
1	2	13811	33251415	11.0	4.6
2	7	16157	29392766	28.8	3.6
3	10	17703	18485656	46.6	4.4
4	11P	9432	39053259	74.2	6.3
5	13	30322	18880899	15.2	5.1
6	16	21088	33684297	43.5	15.0
7	17	21159	28763949	18.7	3.3
8	18P	15089	25011394	29.7	8.0
9	25P	14344	25757693	43.4	3.8
10	27	4051	51758540	79.2	6.6
11	37	18653	32410865	23.5	5.9
12	45	18489	45199479	56.7	6.8
13	36P	12947	20229006	27.2	5.9
14	52P	17786	17282425	31.8	4.1
15	56	19508	28286457	33.0	10.3
16	58	19266	32600982	19.0	3.2
17	65P	13965	18847691	32.2	5.8
18	67	24793	32793229	14.9	8.4
19	69	26731	23059318	47.0	9.9
20	70	27286	26804683	17.6	5.0
21	72	2595	13738261	26.8	6.5
22	74	17992	35115739	62.4	9.0
23	76	22276	58264795	56.0	13.3
24	77	603	28139287	51.9	11.3
25	80	8883	52602981	28.1	3.9
26	121P	16179	23967392	56.4	6.0
27	127	9677	59010900	89.0	3.4
28	125P	15292	24261930	40.7	10.2
29	131	12817	21188487	26.8	11.4
30	133	7310	20627413	26.5	25.8
31	137	11664	20951049	71.9	9.7
32	140	10237	32122054	2.6	3.5
33	144	20035	25480379	34.6	6.7
34	134P	19708	19920079	42.4	7.1
35	148	19356	23454865	11.0	3.9
36	150	12176	19070849	8.4	3.4
37	186	21211	73860813	82.6	5.7
38	198	11857	24696911	43.4	10.1
39	213	1844	20602676	71.4	21.6
40	221	12079	19030621	25.2	8.6
41	222	19689	19031381	27.9	10.8
42	223	3929	28142019	24.6	7.0
43	230	13180	18152999	8.1	5.8
44	235	13643	88547026	90.7	3.6
45	234P	7007	48304862	29.8	35.7
46	240P	11104	39568643	24.9	4.8
47	238P	8732	46699226	58.7	3.3
48	253	9214	19365608	53.8	6.7
